# Bioinformatic prediction, qCLASH, and AlphaFold3 reveal non-overlapping layers of the miR-214-3p–mRNA interactome

**DOI:** 10.3389/fgene.2026.1924314

**Published:** 2026-09-30

**Authors:** Jia Yao, Bingqiao Huang, Chenyu Chang, Ziqi Lin, Jinyue Lei, Weifeng Zhu, Mo Lisha, Tielong Xu

**Affiliations:** 1 School of Pharmacy, Jiangxi University of Chinese Medicine, Nanchang, Jiangxi, China; 2 Evidence-Based Medicine Research Center, Jiangxi University of Chinese Medicine, Nanchang, Jiangxi, China; 3 The Affiliated Hospital of Jiangxi University of Chinese Medicine, Nanchang, Jiangxi, China

**Keywords:** AlphaFold3, bioinformatic prediction, cross-method concordance, miRISC, miRNA–mRNA interaction, qCLASH

## Abstract

**Introduction:**

MicroRNAs (miRNAs) regulate gene expression post-transcriptionally through sequence-specific targeting of messenger RNAs. Reliable mapping of miRNA-mRNA interactions remains challenging because available methods differ widely and systematic cross-method comparisons are lacking.

**Method:**

Using mouse miR-214-3p as a model, we systematically compared three approaches: bioinformatic prediction (TargetScan, miRDB, miRWalk), Argonaute-dependent experimental detection (qCLASH on mouse lung tissue), and artificial intelligence-driven structural modeling (AlphaFold3). Gene lists were harmonized to Ensembl release 116 (GRCm39) Gene IDs; overlaps were evaluated against a transcriptome background using Fisher’s exact tests with Benjamini–Hochberg correction, and the three-tool consensus was assessed by 1,000,000 Monte Carlo simulations.

**Results:**

Only 95 target genes were commonly predicted by all three bioinformatic tools, and at most 6 of the 15 Ensembl-mappable qCLASH genes were captured by any prediction algorithm. Nevertheless, all observed overlaps were significantly enriched relative to random expectation (O/E ratios 24.52–80.6; adjusted q < 0.05), and the 95-gene consensus fell far outside the simulated null distribution (P ∼ MC∼ < 10^−6^). AlphaFold3 produced high-confidence models (ipTM ≥0.6) for 80.95% of qCLASH-derived interactions, but a substantial fraction of bioinformatically predicted sites failed to form structurally plausible complexes.

**Discussion:**

Bioinformatic prediction, qCLASH, and AlphaFold3 thus capture distinct and complementary layers of miRNA targeting (sequence-defined potential, context-dependent occupancy, and spatial feasibility) rather than converging on a single landscape. High AlphaFold3 confidence scores, for instance, did not guarantee biologically functional conformations; even models meeting the thresholds often displayed non-canonical architectures incompatible with silencing. In our mouse lung miR-214-3p model system, our findings support an integrative approach: method selection should align with the specific research question, and multi-layer integration is key to constructing reliable miRNA-target maps in a given biological context.

## 1 Introduction

MicroRNAs (miRNAs) are ∼20-nt non-coding RNAs that regulate gene expression at the post-transcriptional level by targeting mRNAs. The seed sequence (nucleotides 2–8 at the 5′end) guides Argonaute (AGO) proteins to form a silencing complex (miRISC) with target mRNAs. Nevertheless, defining a reliable miRNA–mRNA interaction landscape remains challenging due to the intrinsic complexity, thermodynamic constraints, and strong context dependency of miRNA targeting *in vivo*. In recent years, miRNA-based targeted drugs have become an important direction in drug development. However, a single miRNA can act on multiple mRNAs, making it a significant challenge to comprehensively understand and mitigate off-target effects. Mapping miRNA–mRNA interactions remains a central challenge for understanding regulatory networks and enabling therapeutic applications.

Current approaches for identifying miRNA–mRNA interactions can be broadly categorized into three types. sequence-based bioinformatic prediction, AGO-based experimental detection (qCLASH) (Ule et al., 2003; Wang et al., 2009; Helwak et al., 2013; Gay et al., 2021) and AI-driven structural modeling (AlphaFold3). Each approach is grounded in fundamentally different principles (detailed in Methods), and therefore may computational predictions and experimental interactomes capture distinct aspects of miRNA targeting. Although low concordance among is widely acknowledged, quantitative evidence directly comparing sequence-defined prediction, context-dependent occupancy, and structural feasibility within a unified dataset has been lacking. Several integrative frameworks, such as MIRit (Ronchi and Foti, 2026), have recently been developed to combine multi-omic miRNA–target data. However, these frameworks have not yet incorporated structure-based modeling, leaving the structural dimension of miRNA targeting unexplored in systematic cross-method comparisons. To address this question, we performed a systematic and quantitative comparison of three representative strategies—bioinformatic prediction (TargetScan, miRDB, miRWalk), AGO-dependent experimental detection (qCLASH), and AI-driven structural modeling (AlphaFold3) —using mouse miR-214-3p as a model in a focused, high-resolution case analysis that minimizes biological variability while enabling precise cross-method evaluation. We explicitly tested agreement across methods at both gene and target-site levels, and evaluated the structural plausibility of identified interactions within AGO2-mediated complexes. While previous comparisons have largely been restricted to pairwise evaluations, no study has systematically integrated structural modeling into a three-layer comparative framework. Here, we introduce AlphaFold3 into this landscape for the first time, enabling us to test not only whether predicted and captured interactions agree at the sequence level, but whether they are structurally compatible with AGO2-mediated silencing. This three-layer integration provides a structured framework for interpreting miRNA–mRNA interaction data across complementary methodological dimensions.

## 2 Methods and materials

The overall workflow of this study is shown in Figure 1.

**FIGURE 1:**
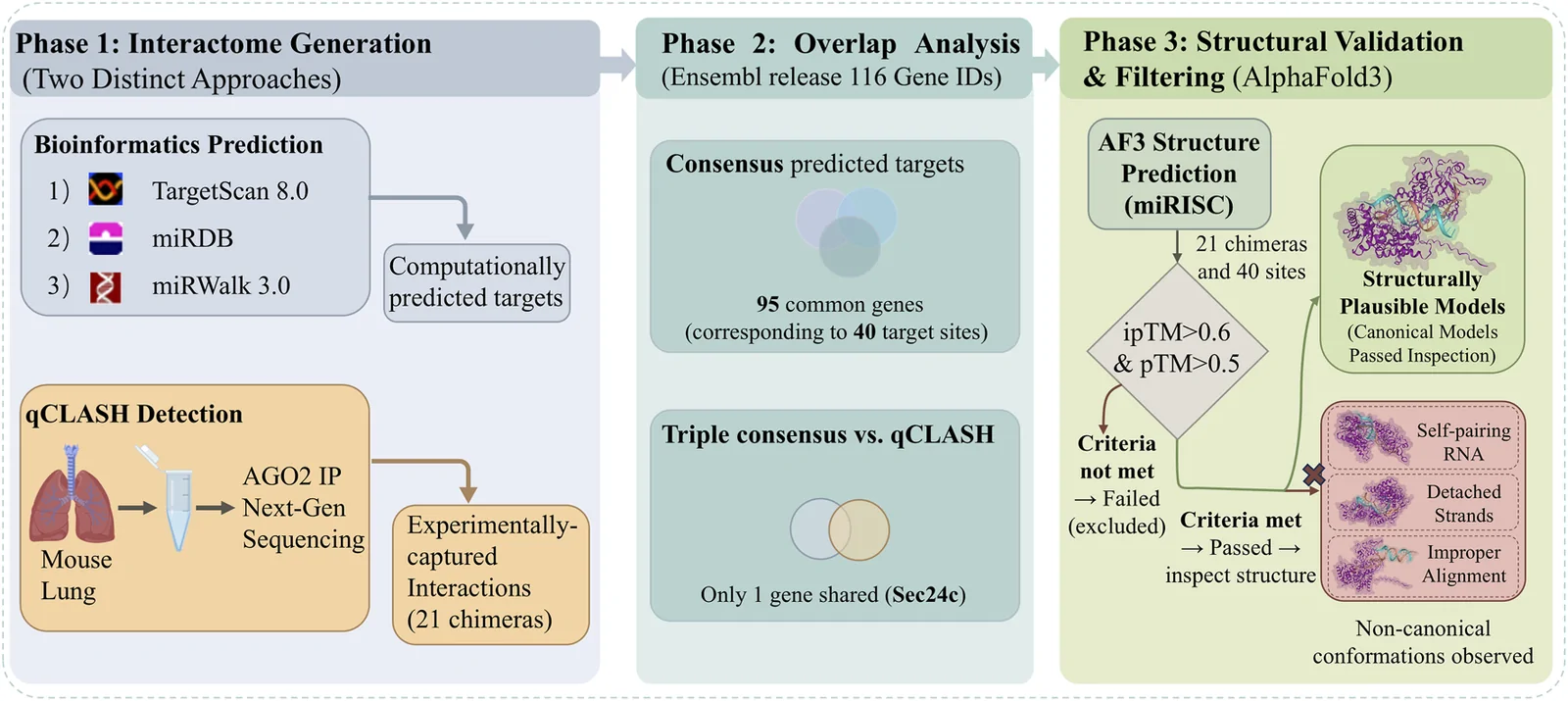
Three-phase workflow for cross-method comparison of the miR-214-3p--mRNA interactome in mouse lung tissue. Phase 1: sequence-based bioinformatic prediction was performed using TargetScan 8.0, miRDB, and miRWalk 3.0; AGO-dependent experimental detection was performed using qCLASH, yielding 21 chimerics. Phase 2: gene symbols from all four sets were harmonized to Ensembl release 116 (GRCm39) genes; the three prediction tools shared 95 common genes (corresponding to 40 unique target sites), of which only one gene (Sec24 c) was also detected by qCLASH. Phase 3: AlphaFold3 (AF3) was used to predict three-dimensional structures of the miRNA-induced silencing complex (miRISC) for both the 40 consensus target sites and the 21 qCLASH-derived chimeric events. Candidate models were screened using interface predicted TM-score (ipTM ≥0.6) and predicted TM-score (pTM >0.5). Models meeting these thresholds were subsequently inspected for structural plausibility and classified as either canonical (structurally plausible) or non-canonical conformations. See Methods for detailed parameters.

### 2.1 Identification of miR-214-3p–mRNA interactomes using different methods

#### 2.1.1 Bioinformatics prediction

The interaction profile of miR-214-3p–mRNA in mice was predicted using three methods: TargetScan 8.0, miRDB, and miRWalk 3.0. The principles and prediction threshold settings for each method are as follows: TargetScan (Lewis et al., 2005; Grimson et al., 2007): This method predicts target sites located in the 3′untranslated region (UTR) of mRNAs based on the principle of strict complementary pairing of the miRNA seed region and the evolutionary conservation of target sites. The cumulative weighted context^++^ score was set to ≤ −0.3. miRDB (Liu and Wang, 2019): This method uses the support vector machine-based MirTarget model to specifically predict functional target sites located in the 3′UTR of mRNAs. The target prediction score was typically set to ≥80. miRWalk 3.0 (Sticht et al., 2018): This is an integrated prediction platform that uses the TarPmiR algorithm based on random forest machine learning to predict target sites for the miRNA of interest. It can predict target sites across the entire mRNA transcript, including the 3′UTR, 5′UTR, and coding sequence (CDS).

#### 2.1.2 qCLASH detection

The qCLASH technique (Helwak et al., 2013; Gay et al., 2021) was employed to detect the miR-214-3p–mRNA interaction profile in mouse lung tissue. Firstly, mouse lung tissue samples were collected. Six female Kunming mice, approximately 25 g and 6–8 weeks old, were used in this experiment. The mice were housed under a 12-h light/dark cycle at 25 °C and 60% relative humidity, with free access to food and water. After 1 week of acclimatization, mice were euthanized by cervical dislocation under deep anesthesia, and lung tissues were immediately dissected for subsequent analysis. This animal experiment was approved by the Animal Medical Ethics Committee of Jiangxi University of Chinese Medicine (Approval No. JZLLSC20241017). Then, qCLASH detection of mouse lung tissue samples was performed according to references (Helwak et al., 2013; Gay et al., 2021). The detection process is briefly described as follows: (i) UV Cross-linking: Lung tissue samples were ground to a fine powder in liquid nitrogen. The frozen powder was then resuspended in pre-cooled DPBS (Corning, 21-030-CV) and transferred to a pre-cooled culture dish inside a UV crosslinker (CL-3000, AnalytikJena). The samples were exposed to 254 nm UV light at a total dose of 400 mJ/cm^2^ over 24 min. To maintain a cryogenic environment during crosslinking and ensure uniform exposure, liquid nitrogen was periodically added to the crosslinker chamber, and the suspension was gently resuspended every 4 min using a pre-chilled pipette. This procedure was adapted from the standard qCLASH protocol to accommodate tissue powder processing while preserving RNA–protein crosslinking efficiency. (ii) Specific Antibody Binding: The tissue lysate was prepared, DNA contamination was removed, and target mRNAs were fragmented by RNase treatment to obtain pre-processed samples. An anti-AGO2 antibody was used to immunoprecipitate AGO2 protein from the pre-processed samples, thereby capturing the miRNA–AGO–target mRNA silencing complexes (i.e., miRISCs). (iii) Generation of Head-to-Tail miRNA–Target mRNA Hybrids: Using Ligase 1, the 5′end of the target mRNA and the 3′end of the miRNA within the captured miRISC were ligated to generate head-to-tail miRNA–target mRNA hybrid chimeras. After processing the ends of the hybrid chimeras, specific adapters were ligated, followed by purification and ethanol precipitation to extract the chimeric nucleic acids. (iv) Labeling, Library Preparation, and Sequencing: The chimeric nucleic acids were reverse transcribed, PCR-amplified using labeled primers, size-selected via agarose gel electrophoresis, and then subjected to next-generation sequencing. (v) Analysis of Sequencing Results: The sequencing data were generated with a target depth of 10 Gb per sample. The hyb pipeline was used to identify chimeric reads containing both miRNA and mRNA sequences (Helwak et al., 2013; Travis et al., 2014). Briefly, adapter sequences were trimmed and low-quality bases were filtered (Phred quality threshold ≥30; minimum read length ≥17 nt) using Flexbar. Identical reads were collapsed to remove PCR duplicates. Clean reads were aligned to the mouse reference transcript database using Bowtie2 with local alignment mode. Chimeric reads containing both miRNA and mRNA sequences were identified by requiring two non-contiguous matches to the reference database with a maximum gap/overlap of 4 nucleotides (gmax = 4) and an e-value threshold of 0.1 (hval = 0.1), with transcript preference enabled to prioritize miRNA–mRNA chimeras (pref = mim). From these chimeric reads, reads containing miR-214-3p were selected as candidate chimeras. Candidate chimeras were further filtered by requiring: i) miR-214-3p at the 5′end of the read, ii) no perfect full-length alignment to a single gene locus, and iii) BLAST of the mRNA-derived portion using the NCBI BLAST online service with default parameters (Standard databases, organism set to *Mus musculus*, taxid:10090), allowing up to 5 mismatches. Raw-read-level QC figures were not retained after the deduplication step. The qCLASH sequencing data are publicly available via Zenodo (https://zenodo.org/records/16885174). The dataset comprises six files (1_10_18, 2_10_18, 3_10_18, 1_10_25, 2_10_25, and 3_10_25) corresponding to the six individual mice used in this study. These data were originally generated from mouse lung tissue as part of a previous study from our group (Wu et al., 2026) and were re-analyzed here for miR-214-3p targets.

#### 2.1.3 AlphaFold3-based 3D structure prediction of miRISCs

To benchmark the reliability of our AlphaFold3 pipeline for AGO2–RNA complexes, we first performed a positive control using the experimentally determined crystal structure of human AGO2 bound to a guide RNA (PDB: 4W5N; resolution 2.9 Å). The protein and RNA sequences were extracted from this PDB entry and submitted to AlphaFold3 using the identical parameter settings as described below. The top-ranked predicted model was compared with the experimental structure using US-align (Zhang et al., 2022) to quantify structural similarity via TM-score and RMSD.

After validating the pipeline, we applied AlphaFold3 to predict the three-dimensional structures of miR-214-3p–containing miRISCs. The AGO2 protein sequence was derived from the mouse reference sequence. The miRISCs predicted here included: (i) miRISCs formed by miR-214-3p interacting with target mRNAs commonly predicted by all three bioinformatics methods; and (ii) miRISCs formed by miR-214-3p interacting with each target mRNA identified by qCLASH.

Qualified miRISCs were screened using interface predicted TM-score (ipTM) ≥ 0.6 and predicted TM-score (pTM) > 0.5, permissive confidence cut-offs recommended in the AlphaFold3 Server guidelines (Abramson et al., 2024). These thresholds were adopted because, in a recent independent benchmark on protein–nucleic acid and RNA complexes, Peng(Peng et al., 2025) showed that AF3’s ipTM correlates with observed interface fidelity (FNAT) and that pTM tracks global fold accuracy on such assemblies, supporting the cautious transfer of these cut-offs to AGO2–miRNA–mRNA ternary complexes.

### 2.2 Consistency analysis of results from different identification methods

#### 2.2.1 Statistical analysis of overlaps among the three bioinformatics prediction methods

To enable rigorous comparison, gene symbols from TargetScan, miRDB and miRWalk were mapped to Ensembl release 116 (GRCm39) Gene IDs, and only uniquely mapped genes were retained for statistical analysis; unmapped or ambiguously mapped symbols were excluded. The mouse transcriptome background was defined as all genes with at least one annotated transcript in the Ensembl GRCm39 annotation file (N = 78,348).

For each pair of prediction tools, we calculated the observed intersection k, the expected intersection under independent sampling E = |A||B|/N, the observed-to-expected ratio O/E = k/E, and the Jaccard similarity coefficient J = k/(|A| + |B| − k). Statistical enrichment was evaluated using Fisher’s exact test based on the 2 × 2 contingency table. The three pairwise comparisons were corrected for multiple testing using the Benjamini–Hochberg procedure. For the three-way intersection among all three tools, we performed 1,000,000 fixed-size simulations and calculated a Monte Carlo P value with the plus-one correction.

#### 2.2.2 Statistical analysis of qCLASH enrichment across prediction-support tiers

qCLASH genes were similarly mapped to Ensembl release 116 (GRCm39) Gene IDs using the same filtering criteria (only uniquely mapped genes retained; unmapped or ambiguously mapped symbols excluded). To evaluate whether qCLASH-supported genes were enriched among computational predictions, three prespecified nested prediction sets were constructed: genes supported by at least one, at least two, or all three prediction tools. Each tier was compared with the qCLASH set using the same transcriptome background (N = 78,348) and Fisher’s exact test framework. The three Fisher tests were corrected together using the Benjamini–Hochberg procedure. In addition, mutually exclusive strata supported by exactly zero, one, two or three tools were summarized to assess whether qCLASH frequency changed monotonically with predictor count.

#### 2.2.3 Structural modeling and assessment of miRISC candidates by AlphaFold3

Given that bioinformatics prediction methods typically yield a large number of potential miRNA target mRNAs, it is impractical to perform 3D structure prediction for all candidate miRNA–mRNA interaction complexes (miRISCs). This study focused on the target mRNA sites commonly predicted by all three bioinformatics methods. AlphaFold3 was used to individually simulate the 3D structure of miRISCs formed by miR-214-3p, the target site sequence, and AGO2. The qualified miRISCs were then screened to assess the structural feasibility of bioinformatically predicted targets. Similarly, for the miRNA–mRNA interaction complexes (miRISCs) directly captured by qCLASH experiments, we performed structural prediction analysis using AlphaFold3 and screened for qualified miRISCs to assess the structural feasibility of qCLASH-identified targets. In this workflow, AlphaFold3 was applied after candidate prioritization (i.e., the consensus bioinformatics targets and qCLASH-identified targets) for structural feasibility assessment, rather than as an independent transcriptome-wide screening method. Therefore, AlphaFold3 outputs are reported as candidate-level structural evidence and are not treated as an additional gene set in the gene-overlap statistics presented in Sections 2.2.1 and 2.2.2.

For the qualified miRISC models (those meeting the ipTM and pTM thresholds described in Section 2.1.3), we further assessed whether their three-dimensional conformations were compatible with the established miRNA action mechanism. The 3D structural data predicted by AlphaFold3 were imported into the PAE Viewer platform (https://pae-viewer.uni-goettingen.de/) for visualization. Using this view, we examined whether the miRNA–mRNA interacting duplex was located within the RNA-binding functional domains of AGO2 and whether the spatial arrangement among the three components conformed to the characteristics of a functional miRISC. This assessment served to evaluate whether AlphaFold3 models that pass the confidence thresholds nevertheless exhibit conformational features incompatible with miRNA-mediated silencing.

## 3 Results

### 3.1 miR-214-3p–mRNA interactomes identified by different methods

#### 3.1.1 Bioinformatics prediction results

Prediction analyses using TargetScan 8.0, miRDB, and miRWalk 3.0 identified 606, 213, and 904 potential target mRNAs for miR-214-3p in the mouse transcriptome, respectively, after harmonizing gene symbols to Ensembl release 116 (GRCm39) Gene IDs (raw outputs are provided in Supplementary Appendix Excels 1–3). The intersection of target mRNAs common to all three methods comprised 95 mRNAs (Supplementary Appendix Excel 4).

#### 3.1.2 qCLASH experimental detection results

Using qCLASH technology and applying the filtering criteria described in Methods 2.1.2, we identified 200 supporting reads containing miR-214-3p, which were collapsed to 21 high-confidence chimeric hybrids. Among these 21 chimeric sites, 10 fell within coding sequences (CDS), 5 in 3′untranslated regions (3′UTRs), 1 in 5′UTR, and 5 mapped to unannotated genomic regions (Supplementary Appendix Table 1). Of the 16 sites assignable to annotated transcript regions, 11 (68.75%) lay outside 3′UTRs. We further analyzed the base complementarity between miR-214-3p and these target mRNAs, as well as their Minimum Free Energy (MFE). The results showed that the reverse complementary pairing structures conformed to the classical miRNA action mechanism, and their MFEs were all well below the threshold for stable duplex formation, ranging from −33.8 to −20.3 kcal/mol. These parameters support the reliability of the qCLASH detection results (Supplementary Appendix Table 1 provides the sequences, read counts, complementarity details, site region, and MFE values).

#### 3.1.3 AlphaFold3 structure prediction results

Throughout this section, structural confidence refers to AlphaFold3-derived metrics (ipTM/pTM) assessing the reliability of the predicted spatial arrangement, not the biological functionality of the miRNA–mRNA interaction. Notably, the “classical pairing”confirmed here refers to the base-pairing geometry at the miRNA–mRNA interface, whereas the “non-canonical” features discussed below refer to target site locations and seed-matching patterns—two distinct aspects that can coexist in the same interaction.

In the positive control using the human AGO2–guide RNA complex (PDB: 4W5N), AlphaFold3 predicted the structure with ipTM = 0.95 and pTM = 0.93. Comparison with the crystal structure using US-align yielded a TM-score of 0.956 and an RMSD of 2.19 Å over 806 aligned residues, confirming that our pipeline can reliably recapitulate AGO2–RNA complex architecture (Supplementary Appendix Figure 2).

From the 95 consensus target genes identified by all three bioinformatics methods (Section 3.1.1), we extracted the specific target sites predicted by each tool; after deduplication, this yielded 40 unique target sites (Supplementary Appendix Excel 5). AlphaFold3 was used to individually model the miRISC formed by miR-214-3p, each target site sequence, and AGO2. The results, as shown in Table 1, are as follows: 24 (60%) miRISCs had ipTM values between 0.6 and 0.8, indicating that their 3D structural models were predicted with relatively high confidence; 3 (7.50%) miRISCs had ipTM >0.8, indicating high-confidence 3D structural models; and 13 (32.50%) miRISCs had ipTM <0.6, indicating unreliable 3D structural models. The pTM values for all miRISCs were greater than 0.5, falling within the range of 0.71–0.90 (Table 1), suggesting that the backbone folding state of the AGO2 protein in the predicted models remained globally well-folded at the overall structural level (Supplementary Appendix Table 2). Furthermore, for the 21 target sites identified by qCLASH, AlphaFold3 structural predictions indicated that 80.95% of the models (17/21) met the reliability threshold (ipTM ≥0.6), with 23.81% (5/21) being high confidence (ipTM >0.8). The pTM values for all models remained consistently within the high-confidence range of 0.86–0.91 (Table 2; Supplementary Appendix Table 3).

**TABLE 1:** Summary of AlphaFold3 prediction confidence for miRISCs obtained from bioinformatics methods.

ipTM score*	Number of miRISC	Percentage (%)
>0.8	3	7.50
0.6–0.8	24	60.00
<0.6	13	32.50
Total	40	100

* ipTM, range was divided according to AlphaFold3.

**TABLE 2:** Summary of AlphaFold3 prediction confidence for miRISCs obtained from qCLASH.

ipTM score*	Number of miRISC	Percentage (%)
>0.8	5	23.81
0.6–0.8	12	57.14
<0.6	4	19.05
Total	21	100

* ipTM, range was divided according to AlphaFold3.

### 3.2 Consistency analysis of miR-214-3p–mRNA interactomes identified by different methods

To evaluate overlaps at the gene level, we mapped the gene symbols from all four methods to Ensembl release 116 (GRCm39) Gene IDs. For the three prediction tools, the mapped set sizes were 606 (TargetScan), 213 (miRDB), and 904 (miRWalk), as described in Section 3.1.1. For qCLASH, the 21 chimeric events corresponded to 15 uniquely mapped genes, which were used in the following gene-set analyses.

#### 3.2.1 Overlap among the four gene sets

The UpSet plot in Figure 2a visualizes all non-zero exact intersections among the four gene sets. Among the 12 non-zero intersection combinations, the largest subset was unique to miRWalk (519 genes), followed by genes unique to TargetScan (316 genes), and genes shared by miRWalk and TargetScan (183 genes). Pairwise Jaccard coefficients ranged from 0.00441 to 0.227, indicating limited absolute overlap. TargetScan shared 105 genes with miRDB (expected 1.65; O/E = 63.73; q = 1.02 × 10^−163^) and 279 genes with miRWalk (expected 6.99; O/E = 39.90; q = 2.47 × 10^−382^). miRDB and miRWalk shared 198 genes (expected 2.46; O/E = 80.56; q = 1.92 × 10^−371^) (Figure 2b). qCLASH overlapped with TargetScan, miRDB and miRWalk by 3, 1 and 5 genes, respectively, corresponding to O/E ratios of 25.9, 24.5 and 28.9. The three prediction tools shared 95 genes in total, of which 94 were not detected by qCLASH, while one gene (Sec24 c) was present in all four sets (Figure 2a). The expected three-way overlap under independent random sampling was 0.0190 (O/E = 4,997.6). Across 1,000,000 fixed-size simulations, none reached the observed overlap of 95 (P ∼ MC∼ < 10^−6^; maximum simulated overlap = 3) (Figure 3). This 95-gene consensus, despite representing only 7.69% of the three-set union, was substantially larger than expected by chance. Thus, although absolute overlap was limited, all observed intersections were significantly enriched relative to random expectation.

**FIGURE 2:**
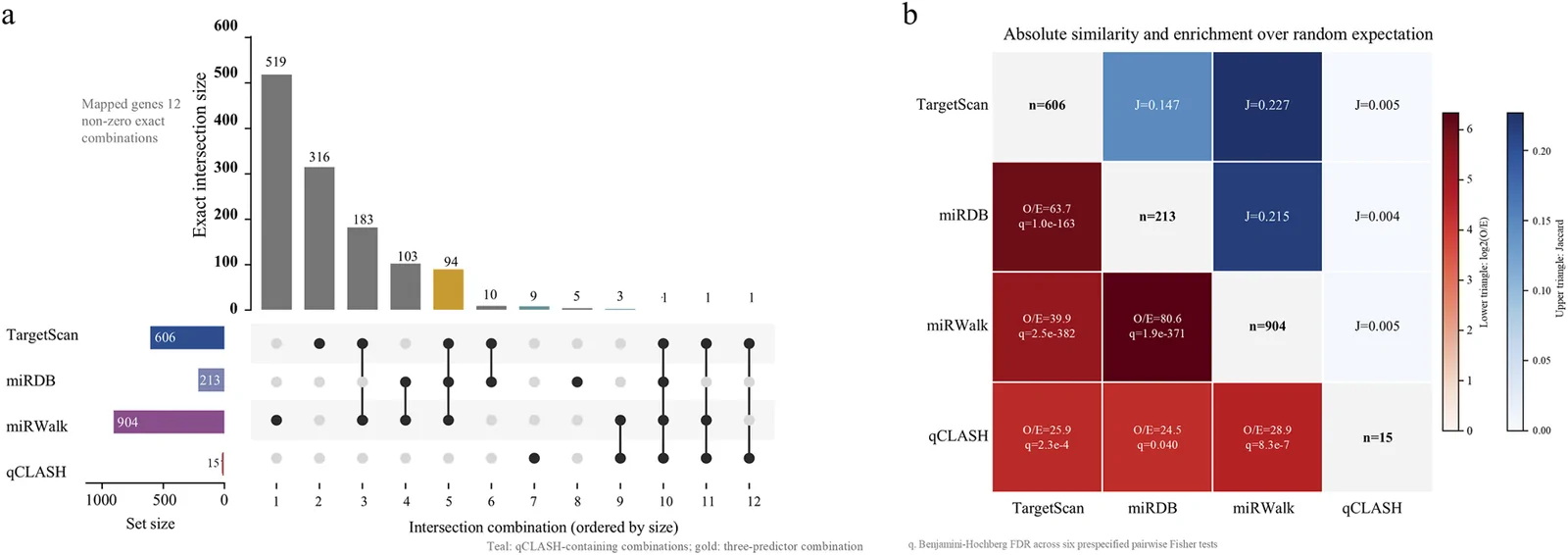
Quantitative visualization of overlaps among TargetScan, miRDB, miRWalk, and qCLASH. **(a)** UpSet plot showing all non-zero exact intersections among the four gene sets. Horizontal bars indicate total set sizes (TargetScan, n = 606; miRDB, n = 213; miRWalk, n = 904; qCLASH, n = 15). Vertical bars show the 12 non-zero intersection combinations, with the dot matrix indicating set membership. Teal bars denote combinations containing qCLASH; the gold bar highlights the 94 genes shared by all three prediction tools but not by qCLASH. The three prediction tools shared 95 genes in total, of which 94 were not detected by qCLASH; the remaining gene, Sec24 c, was present in all four sets. **(b)** Pairwise comparison heatmap. Upper triangle: Jaccard similarity coefficients. Lower triangle: log_2_-transformed observed-to-expected enrichment (O/E), with the untransformed O/E ratio and Benjamini–Hochberg-adjusted q value annotated in each cell. Diagonal cells show mapped set sizes. All overlaps were evaluated against a mouse transcriptome background of N = 78,348 Ensembl release 116 (GRCm39) genes.

**FIGURE 3:**
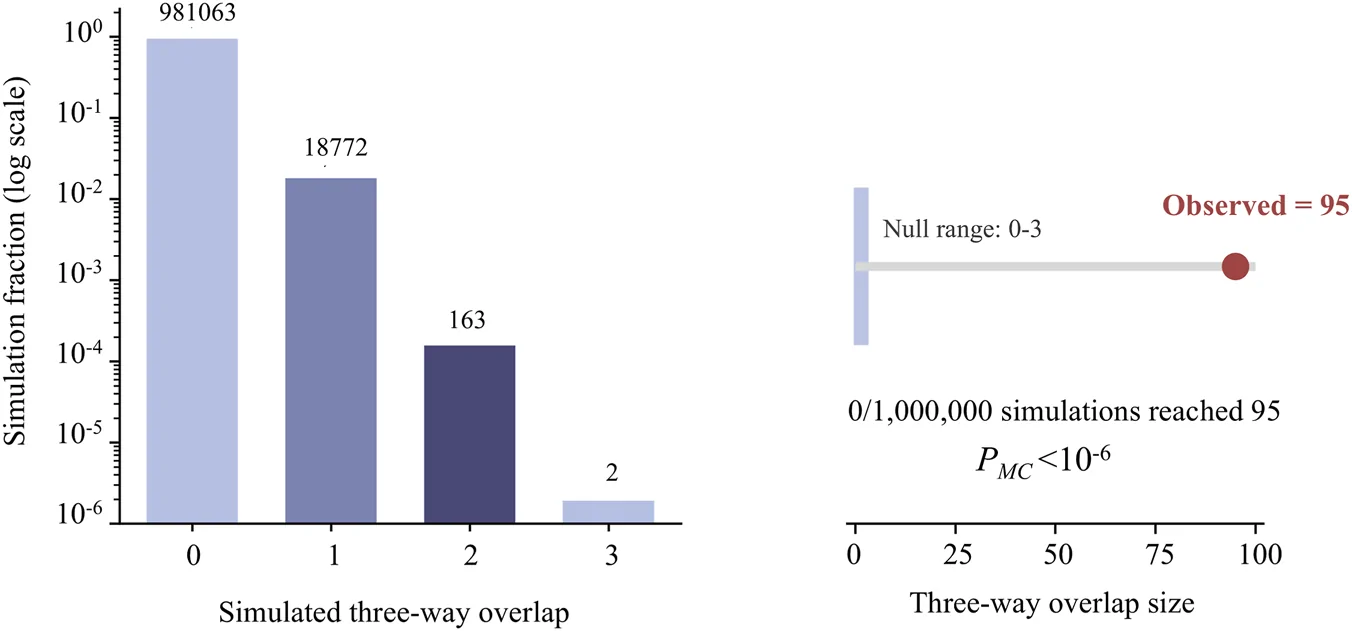
Null distribution of the three-prediction-tool intersection. Fixed-set-size Monte Carlo null distribution for the common intersection of TargetScan, miRDB, and miRWalk in a background of N = 78,348 mouse Ensembl release 116 (GRCm39) genes. Among 1,000,000 simulations, the three-way overlap was 0 in 981,063 simulations, 1 in 18,772 simulations, 2 in 163 simulations, and 3 in 2 simulations; the observed overlap of 95 genes (red dashed line) was not reached in any simulation (P ∼ MC∼ < 10^−6^). Bar labels indicate simulation counts; the left ordinate is logarithmic.

#### 3.2.2 qCLASH-detected genes are enriched in computationally predicted sets

Having established the overall overlap landscape among the four gene sets (Section 3.2.1), we next asked whether qCLASH-supported genes were enriched within computationally predicted sets. As shown in Table 3, six of the 15 qCLASH genes were supported by at least one prediction tool (expected 0.2366; O/E = 25.36; odds ratio = 41.78, 95% CI 12.22–131.66; q = 2.02 × 10^−7^). Two qCLASH genes were supported by at least two predictors (expected 0.0750; O/E = 26.65; odds ratio = 30.74, 95% CI 3.36–136.46; q = 3.77 × 10^−3^), and one gene, Sec24 c, was shared by qCLASH and all three prediction tools (expected 0.01819; O/E = 54.98; odds ratio = 59.41, 95% CI 1.39–398.11; q = 1.80 × 10^−2^). Among the qCLASH genes, Sec24 c and Sun2 were supported by at least two tools; Sec24 c was the only gene jointly identified by all three prediction methods and qCLASH.

**TABLE 3:** Enrichment of qCLASH-supported genes across prediction-support tiers.

Prediction support tier	Gene set size	qCLASH overlap	Expected overlap	O/E	Odds ratio (95% CI)	BH-adjusted q
≥1 tool	1,236	6	0.2366	25.36	41.78 (12.22–131.66)	2.02 × 10^−7^
≥2 tools	392	2	0.0750	26.65	30.74 (3.36–136.46)	3.77 × 10^−3^
3 tools	95	1	0.01819	54.98	59.41 (1.39–398.11)	1.80 × 10^−2^

O/E, observed-to-expected ratio; BH, Benjamini–Hochberg. The expected overlap was calculated as E = |A||B|/N, where N = 78,348 (mouse transcriptome background, Ensembl release 116 GRCm39). The three nested prediction tiers are cumulative: “≥ 1 tool” includes genes supported by at least one of TargetScan, miRDB, or miRWalk; “≥ 2 tools” includes genes supported by at least two; “3 tools” includes genes supported by all three. Odds ratios are conditional maximum-likelihood estimates with exact 95% confidence intervals. q values are Benjamini–Hochberg adjusted Fisher’s exact P values, corrected across the three prespecified nested comparisons.

To further examine whether qCLASH support increases monotonically with the number of supporting predictors, we stratified the transcriptome into mutually exclusive groups supported by exactly one, two, or three prediction tools. The qCLASH proportions in these strata were 4/844 (0.47%), 1/297 (0.34%), and 1/95 (1.05%), respectively (Table 3). This pattern did not show a strictly monotonic increase. Together, these results support enrichment of qCLASH evidence within predicted targets but do not establish a stable dose–response relationship with predictor count.

#### 3.2.3 Structural modeling outcomes and conformational assessment of candidate targets by AlphaFold3

The 40 consensus target sites used for AlphaFold3 structural modeling represent the core set subjected to structural prediction; the subsequent Ensembl-based identifier harmonization (Section 2.2.1) did not affect this structural modeling input. It should be noted that the ‘pass rate’ reported below refers to the proportion of candidate models that met the AlphaFold3 confidence thresholds (ipTM ≥0.6 and pTM >0.5), rather than a measure of method concordance in gene-set overlap.

Based on these criteria, the structural modeling pass rate for bioinformatics-predicted candidates was 67.50%: 27 of the 40 consensus target site models met the thresholds (Table 1; Supplementary Appendix Table 2). For qCLASH-identified targets, the pass rate was 80.95%: 17 of the 21 models met the thresholds (Table 2; Supplementary Appendix Table 3). Combining both sets, the overall pass rate was 72.13% (44 of 61 models).

Among the 44 qualified models (those meeting the ipTM and pTM thresholds described in Section 2.1.3), we further assessed whether their three-dimensional conformations were compatible with the established miRNA action mechanism. Using PAE Viewer for visual analysis of the predicted structures (Supplementary Appendix Figure 1), we found significant diversity among different miRISCs in the spatial conformations of the miRNA and/or target mRNA and their relative arrangement. There was no fixed conformational pattern; instead, structural disorder was observed, which is inconsistent with the established scientific understanding that miRISC spatial structures are relatively conserved. Furthermore, for some miRISCs, despite exhibiting qualified or even high interface scores (ipTM ≥0.6 and pTM >0.5), their spatial configurations did not conform to the conformational patterns expected for miRNA action mechanisms. These mainly manifested as the following unreasonable conformational patterns: (1) Intramolecular self-pairing within the miRNA or target mRNA, failing to form the miRNA–mRNA complementary pairing structure consistent with the classical recognition mode (Figures 4a,b). (2) Within the AGO2 protein, the miRNA and mRNA did not run in an anti-parallel, complementary manner; their spatial orientations were independent and separated from each other (Figure 4c). (3) The miRNA–mRNA interaction duplex was clearly not located within the known RNA-binding spatial region of AGO2. Although contact existed, it was not effectively accommodated by AGO2 (Figures 4d–f). (4) High conformational uncertainty in the predicted-structures, manifested as “broken-chain-like” features in the target mRNA or miRNA (Figures 4g,h), These observations indicate that AlphaFold3 models meeting confidence thresholds do not necessarily represent biologically functional miRISC conformations, and interface confidence scores should be interpreted with caution.

**FIGURE 4:**
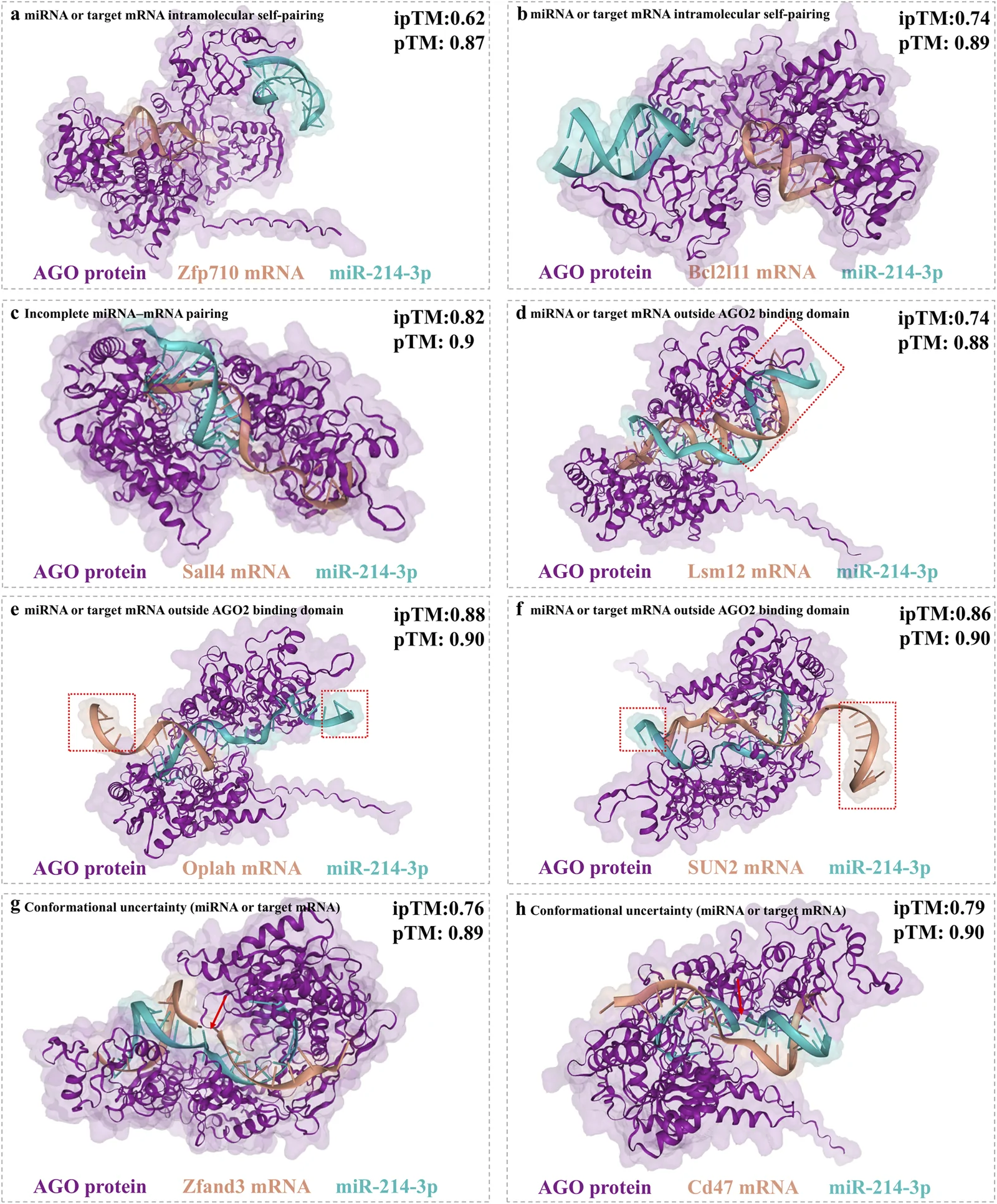
Representative non-canonical spatial features observed in AlphaFold3-predicted miRISC structures. All models shown were selected from those meeting the high-confidence criteria (ipTM ≥0.6 and pTM >0.5; see Methods 2.1.3). **(a,b)** Intramolecular self-pairing within the miRNA or target mRNA, precluding functional duplex formation. **(c)** Incomplete miRNA–mRNA pairing. **(d–f)** Improper placement of the miRNA–mRNA duplex outside the AGO2 RNA-binding domain. **(g,h)** High conformational uncertainty (‘broken-chain-like’ features) in the target mRNA **(g)** or both the miRNA and target mRNA **(h)**. Color code: AGO2, purple; miRNA, turquoise; target mRNA, orange. ipTM, interface predicted TM-score; pTM, predicted TM-score. See also Supplementary Appendix Figure 1 for PAE Viewer visualizations of additional models. Notably, these examples demonstrate that AlphaFold3 models meeting the ipTM and pTM confidence thresholds do not necessarily represent biologically functional miRISC conformations.

## 4 Discussion

MicroRNAs (miRNAs) regulate gene expression post-transcriptionally by pairing with messenger RNAs, and miR-214-3p in particular has been implicated in diverse processes including skeletal development (Wang et al., 2019), immune regulation (Eroglu et al., 2021), inflammatory responses (Yan et al., 2020), multi-organ fibrosis (Elemeery et al., 2017; Yang et al., 2018; Savary et al., 2019; Zhao et al., 2025), cell proliferation (Zhang et al., 2018), tumor progression (Penna et al., 2011; Dettori et al., 2018; Liu et al., 2018; Guo et al., 2021), and viral infections (Santhakumar et al., 2010; Yanagawa-Matsuda et al., 2012; Zhang et al., 2017; Zhang et al., 2019). Understanding how different methodological approaches capture miRNA–mRNA interactions is essential for interpreting such functions, because the functional output of a given miRNA depends heavily on which target mRNAs it engages in a specific context (Bartel, 2009; Jonas and Izaurralde, 2015). Using mouse miR-214-3p as a model, we systematically compared three distinct methodological layers: sequence-based bioinformatic prediction, AGO-dependent experimental mapping (qCLASH), and AI-driven structural modeling (AlphaFold3)—focusing on their consistency and bias rather than on functional validation of individual targets. Our results show that these approaches do not converge on a single interaction landscape; instead, they capture distinct and complementary layers: bioinformatic prediction defines sequence-defined binding potential; qCLASH captures context-dependent occupancy *in vivo*; and AlphaFold3 evaluates spatial feasibility within the AGO2-mediated complex. This layered divergence does not indicate methodological failure, but rather reflects the inherent separation between sequence-based permissibility, real-time occupancy, and structural compatibility.

Bioinformatic tools such as TargetScan and miRDB systematically predict potential targets based on sequence complementarity and evolutionary conservation, thereby delineating a broad, theoretical “bindable space.” In contrast, qCLASH captures stable miRNA–mRNA–AGO ternary complexes formed in a specific cellular context (here, normal mouse lung tissue) (Helwak et al., 2013). The discrepancy between these two layers arises because the formation of such complexes depends not only on sequence complementarity but also on dynamic factors including AGO protein abundance, competitive RNA-binding proteins, target transcript expression levels, and subcellular localization (Akhtar et al., 2019; Boerneke et al., 2019; Xu et al., 2022). Consequently, many computationally predicted high-scoring sites may not be effectively occupied under experimental conditions. Moreover, the AGO2-centric design of qCLASH introduces a potential selection bias, as interactions mediated by other AGO paralogs are not captured. Thus, the experimentally observed interactome represents a subset of the broader miRNA target space, and the tissue-specific context further limits overlap with general computational predictions. This dataset, generated from a single miRNA and a single tissue type, provides a high-resolution case study rather than a comprehensive survey; our conclusions should therefore be interpreted within this specific context.

It should be acknowledged that the bioinformatic prediction layer in this study was represented by three widely used tools—TargetScan, miRDB, and miRWalk—selected for their established status and distinct methodological principles (seed-based conservation, SVM-based scoring, and random forest-based transcript-wide prediction, respectively). However, the field of miRNA target prediction has recently witnessed a surge of machine learning and deep learning-based approaches, including TEC-miTarget (Transformer + CNN) (Yang et al., 2024), microT-CNN (Zacharopoulou et al., 2024), DPAT (Xu et al., 2025), and PAIR-Former, among others. Recent benchmarks have demonstrated that performance varies substantially across tools and biological contexts. Our study did not aim to exhaustively benchmark all available algorithms; rather, we selected three representative tools for cross-method comparison. Among these, miRWalk generated the largest candidate set (904 genes), substantially exceeding TargetScan (606 genes) and miRDB (213 genes). This is partly attributable to its transcript-wide search strategy (including 5′UTR, CDS, and 3′UTR), which, while offering broader coverage, may also increase false positives—consistent with the finding that miRWalk underperformed relative to DIANA-microT and miRmap in recent benchmarks (Faiza et al., 2019). Nonetheless, miRWalk was selected because it provides the specific target site sequences required for AlphaFold3 structural modeling, whereas TargetScan and miRDB do not offer direct sequence access. Thus, its inclusion represents a trade-off between prediction specificity and the practical requirements of structural modeling. We acknowledge that alternative tools might yield different quantitative patterns, but this does not undermine our primary conclusion, as the layered divergence we observed stems from fundamental differences in methodological principles rather than from the performance of any single tool.

The qCLASH technique used here is a derivative of chimera-generating CLIP approaches that directly link miRNAs to their mRNA targets within AGO complexes. This class of methods—exemplified by CLASH and CLEAR-CLIP—bypasses the bioinformatic inference required by first-generation approaches such as HITS-CLIP and PAR-CLIP. However, as with all ligation-based methods, qCLASH is subject to technical constraints that shape the resulting interactome. The intramolecular ligation step joining miRNA and mRNA fragments depends on favorable spatial orientation and RNA end accessibility; genuine interactions that do not present suitable termini or are sterically hindered may fail to ligate, contributing to false negatives. Additionally, library construction and PCR amplification can introduce sequence-dependent biases, potentially under-representing chimeras with extreme GC content, and UV crosslinking captures only interactions that are stably assembled at the moment of crosslinking, thereby missing transient or rapidly exchanging binding events. It should also be acknowledged that our qCLASH experiment was conducted as an application study following the established protocol (Helwak et al., 2013; Gay et al., 2021), and did not include parallel negative controls such as IgG isotype, beads-only, non-crosslinked, or no-ligase conditions. Such controls were essential for establishing the method in the original CLIP framework (Ule et al., 2003) and for demonstrating the *in vivo* origin of chimeric duplexes via spike-in assays (Helwak et al., 2013). However, the qCLASH Basic Protocol does not include these controls in its routine application settings (Gay et al., 2021), and many published application studies rely on the foundational protocol validation rather than repeating all control conditions. Nevertheless, we acknowledge that the absence of these controls limits our ability to formally exclude potential background ligation events and crosslinking-independent RNA associations.

While CLEAR-CLIP has been applied to map ∼130,000 endogenous miRNA–target interactions in mouse brain, analogous tissue-level datasets remain limited. Our qCLASH dataset from mouse lung thus complements existing resources and provides a tissue-specific reference for cross-method comparison. To further assess the reliability of the qCLASH-identified interactions and partially mitigate the aforementioned limitations, we cross-referenced the 21 detected chimeric reads (corresponding to 15 uniquely mapped genes) against the public database ENCORI/starBase, which integrates AGO CLIP-seq data for miRNA–target interactions (Zhou et al., 2026). Among these 15 genes, 5 (33.3%) were found to be supported by ENCORI/starBase: Rhob, Sec24 c, Sun2, Cd47, and Adgra2. This level of external corroboration supports the biological relevance of the qCLASH-derived interactome and partially mitigates the limitation of both the absence of experimental controls and the lack of biological replicates, as the supported interactions are corroborated by externally generated CLIP-seq datasets. Future studies incorporating matched negative controls and biological replicates would be valuable to further strengthen the specificity assessment of qCLASH-derived interactions in tissue settings.

Conversely, a substantial fraction of qCLASH-identified interactions was not captured by any bioinformatic tool. This reflects known constraints of classical prediction algorithms, which emphasize strict seed region complementarity (nucleotides 2–8) and focus predominantly on 3′UTRs. In contrast, experimental methods such as qCLASH have revealed the *in vivo* existence of non-canonical pairing modes, including seed mismatches compensated by 3′-end pairing, G:U wobbles, and functional target sites within CDS or 5′UTR regions (Yao et al., 2024). These patterns are often underrepresented or excluded by conventional algorithms. Furthermore, prediction tools rely on static sequence and thermodynamic features, whereas actual target accessibility is dynamically modulated by RNA secondary structure, RNA-binding protein competition, and post-transcriptional modifications such as m6A (Xu et al., 2022). qCLASH captures the stable interactions that prevail in this complex molecular environment, which are difficult to infer from sequence alone. Among the 21 qCLASH-derived chimeric events, three corresponded to intergenic or clone-derived sequences that could not be assigned to annotated protein-coding genes (re-annotated as unannotated genes 1–3 in Supplementary Appendix Table 1). These entries were therefore not included in the gene-level enrichment analyses presented in the Results section, although their structural models were retained for the AlphaFold3 assessment (Supplementary Appendix Table 3). Their presence highlights the importance of distinguishing between chimeric events and annotated gene targets when interpreting qCLASH data, and also reflects the ability of the approach to capture RNA fragments originating from unannotated or non-genic regions. The genomic context of the 80-read chimeric event (unannotated gene 3) is shown in Supplementary Appendix Figure 3.

When AlphaFold3 was applied to candidate targets derived from bioinformatic predictions, interface confidence metrics (ipTM) did not consistently align with structural plausibility. Even among models with ipTM ≥0.6, we observed non-canonical conformations, including miRNA or mRNA strands deviating from known AGO2 RNA-binding domains, abnormal intramolecular RNA folding, and regions of high local conformational uncertainty. AlphaFold3 was not specifically developed for ternary RNA–protein–RNA complexes such as the miRISC, and its performance on such assemblies has not been systematically benchmarked. The non-canonical conformations observed in some high-ipTM models may therefore reflect current limitations of the model when applied to this complex class, rather than definitive evidence against biological feasibility. A positive control using the human AGO2–guide RNA complex (PDB: 4W5N) confirmed that our pipeline can reliably model known AGO2–RNA binary complexes (TM-score = 0.956, RMSD = 2.19 Å), but this does not guarantee that all high-ipTM ternary models represent biologically relevant conformations. For qCLASH-confirmed interactions, AlphaFold3 produced structurally plausible models in over 80% of cases (Table 1; Supplementary Appendix Table 3), indicating that when interactions are experimentally supported, structural modeling can recapitulate general miRISC features; however, such structural plausibility does not necessarily reflect accurate functional pairing. A model meeting ipTM and pTM thresholds may still represent a non-functional conformation, while a model with lower confidence scores might capture biologically relevant but structurally dynamic interactions. We therefore view AlphaFold3 as a structural plausibility filter rather than a functional validator. It is best used as an auxiliary tool for spatial feasibility assessment rather than as an independent method for high-throughput miRNA target identification (Terwilliger et al., 2024). Its integration with experimentally validated interactions, as demonstrated here, offers a more balanced approach to interpreting miRNA targeting at structural resolution.

Beyond these methodological considerations, the limited overlap among the three layers carries biological implications. The fact that only a small fraction of sequence-defined binding sites are occupied in vivo—and that even fewer are structurally compatible with AGO2—suggests that miRNA targeting is subject to multiple filters operating at different levels: sequence complementarity defines a permissive space of potential interactions, cellular context narrows this space through transcript availability, competition, and AGO loading, and structural compatibility further restricts the space to conformations permissive for silencing. Viewed through this lens, the low concordance observed here does not imply that any single approach is ‘wrong,’ but rather that miRNA-target interactions are inherently multimodal: sequence-defined potential, context-dependent occupancy, and structural feasibility each capture a distinct facet of the same regulatory system. This perspective reframes the question from ‘which method is more accurate’ to ‘which layer of the system is relevant to a given biological question’—a shift that may inform both the design of future experiments and the interpretation of existing miRNA-target datasets. No single approach is sufficient to fully define the interaction landscape, as each method inherently captures only a subset of the regulatory space. The recent development of integrative frameworks such as MIRit(Ronchi and Foti, 2026) echoes this perspective, providing standardized workflows for multi-omic miRNA–mRNA integration. Within the scope of this miR-214-3p case study, these findings support an integrative framework in which method selection should align with research objectives in a context-dependent manner: bioinformatic prediction for high-throughput candidate screening, AGO-based experimental methods for capturing *bona fide in vivo* interactions, and structural modeling for spatial feasibility assessment. Such multi-level integration represents a promising direction for constructing more reliable miRNA-target maps in future studies across broader biological contexts.

This study focused on methodological comparison rather than functional validation of individual targets, and several limitations should be acknowledged. First, the qCLASH experiment was performed on pooled samples without biological replicates, precluding statistical assessment of reproducibility. Additionally, only female mice were used, which is a limitation given that lung is a sex-dimorphic tissue; future studies incorporating both sexes are needed to assess the generalizability of our findings. Second, AlphaFold3 predictions are inherently computational and may not fully capture the dynamic conformational flexibility of the miRISC complex; the presence of non-canonical conformations in high-ipTM models suggests that interface confidence scores should be interpreted with caution. Third, bioinformatic prediction thresholds were selected based on standard tool recommendations, but alternative thresholds would yield different sets of predicted targets and potentially alter overlap metrics. Future studies incorporating multiple miRNAs, diverse tissues, and experimental replicates are needed to validate and extend these findings.

## Data Availability

The qCLASH sequencing data are publicly available via Zenodo (https://zenodo.org/records/16885174), which provides a valid and persistent identifier (DOI: 10.5281/zenodo.16885174) for the dataset. The AlphaFold3 input sequences, output structural models (including confidence metrics and PAE data), positive control files (PDB: 4W5N and US-align results), and associated processing scripts have been deposited in Figshare and are publicly available under the CC-BY license. The dataset can be accessed at the following DOI: 10.6084/m9.figshare.33142508.
