# Supplementary material for: Bioinformatic prediction, qCLASH, and AlphaFold3 reveal non-overlapping layers of the miR-214-3p–mRNA interactome
Source: Front Genet. 2026 Sep 30;17:1924314. doi: 10.3389/fgene.2026.1924314 (PMC13661022; doi:10.3389/fgene.2026.1924314)
Supplement: Supplementary file 1 [file DataSheet1.pdf]

Note: The number of target genes listed in this sheet reflects the raw prediction output. For statistical analysis, gene symbols were mapped to Ensembl release 116 (GRCm39) Gene IDs; only uniquely mapped genes were retained (see Methods 2.2.1 of the main text).

Target gene	Representative transcript	Gene name	3P-seq tags + 5	Total sites	8mer sites	7mer-m8 sites	7mer-A1 sites	6mer sites	Representative miRNA	Predicted occupancy (low miRNA)	Predicted occupancy (high miRNA)	Predicted occupancy (transfected miRNA)	Cumulative weighted context++ score	Total context++ score	Aggregate PCT
Usp20	ENSMUST00000102849.5	ubiquitin specific peptidase 20	1147	4	3	1	0	3	mmu-miR-214-3p	0.0942	0.5914	2.2565	-0.64	-0.65	< 0.1
Tmem245	ENSMUST00000068792.7	transmembrane protein 245	1295	2	0	1	1	9	mmu-miR-214-3p	0.0714	0.5079	3.0257	-1.53	-1.75	< 0.1
Gpd1l	ENSMUST00000146623.2	glycerol-3-phosphate dehydrogenase 1-like	1645	3	2	1	0	1	mmu-miR-214-3p	0.0798	0.4973	1.8194	-0.76	-0.78	< 0.1
Cnr2	ENSMUST00000097843.3	cannabinoid receptor 2 (macrophage)	7	3	1	1	1	2	mmu-miR-214-3p	0.0811	0.4961	1.9501	-0.83	-0.83	< 0.1
Eif4ebp2	ENSMUST00000020288.8	eukaryotic translation initiation factor 4E binding	13138	5	1	1	3	5	mmu-miR-214-3p	0.0792	0.4884	2.0582	-0.99	-1.08	< 0.1
Cacng4	ENSMUST00000021066.3	calcium channel, voltage-dependent, gamma subunit 4	8	5	2	3	0	3	mmu-miR-214-3p	0.0734	0.4823	2.1512	-0.7	-0.7	< 0.1
Gas7	ENSMUST00000108682.3	growth arrest specific 7	469	5	3	1	1	2	mmu-miR-214-3p	0.0666	0.4574	2.2981	-0.44	-0.45	< 0.1
Bcl2l11	ENSMUST00000110341.3	BCL2-like 11 (apoptosis facilitator)	219	3	0	2	1	1	mmu-miR-214-3p	0.069	0.4487	2.0052	-0.71	-1.41	< 0.1
B3gnt3	ENSMUST00000034260.7	UDP-GlcNAc:betaGal beta-1,3-N-acetylglucosaminylt	58	2	1	1	0	1	mmu-miR-214-3p	0.0762	0.421	1.373	-0.57	-0.66	< 0.1

Scn2b	ENSMUST00000170998.2	sodium channel, voltage-gated, type II, beta	51	2	1	0	1	2	mmu-miR-214-3p	0.0671	0.4043	1.5627	-0.34	-0.34	< 0.1
Luzp1	ENSMUST00000105849.3	leucine zipper protein 1	845	5	0	3	2	6	mmu-miR-214-3p	0.0564	0.3961	2.2202	-0.38	-0.39	< 0.1
Ermn	ENSMUST00000090940.5	ermin, ERM-like protein	5	2	1	1	0	2	mmu-miR-214-3p	0.0608	0.3893	1.6058	-0.55	-0.55	< 0.1
Peak1	ENSMUST00000188142.1	pseudopodium-enriched atypical kinase 1	241	3	1	2	0	0	mmu-miR-214-3p	0.0642	0.3838	1.3715	-0.34	-0.39	< 0.1
Vsx2	ENSMUST00000021665.6	visual system homeobox 2	5	3	1	2	0	3	mmu-miR-214-3p	0.0573	0.3834	1.8466	-0.3	-0.3	< 0.1
Creb1	ENSMUST00000087366.5	cAMP responsive element binding protein 1	1211	1	1	0	0	2	mmu-miR-214-3p	0.0607	0.382	1.5461	-0.52	-0.58	< 0.1
Afmid	ENSMUST00000073388.7	arylformamidase	185	5	3	1	1	0	mmu-miR-214-3p	0.0567	0.3715	1.559	-0.84	-0.97	< 0.1
Peli3	ENSMUST00000120475.2	pellino 3	65	6	0	5	1	2	mmu-miR-214-3p	0.0534	0.3712	1.9288	-0.5	-0.52	< 0.1
Abcc5	ENSMUST00000079158.7	ATP-binding cassette, sub-family C (CFTR/MRP), member 5	144	1	1	0	0	5	mmu-miR-214-3p	0.0687	0.3681	0.9999	-0.34	-0.35	< 0.1
Nabp2	ENSMUST00000164199.2	nucleic acid binding protein 2	116	1	1	0	0	1	mmu-miR-214-3p	0.0711	0.3629	0.8412	-0.53	-0.54	< 0.1
Hykk	ENSMUST00000039742.7	hydroxylysine kinase 1	2556	3	1	2	0	0	mmu-miR-214-3p	0.0559	0.3588	1.4123	-0.49	-0.51	< 0.1
Naa15	ENSMUST00000029303.7	N(alpha)-acetyltransferase 15, NatA auxiliary subunit	2921	1	0	1	0	2	mmu-miR-214-3p	0.0603	0.3514	1.1247	-0.61	-0.64	< 0.1

Traf3	ENSMUST00000117269.2	TNF receptor-associated factor 3	465	2	0	2	0	5	mmu-miR-214-3p	0.0497	0.3506	1.9956	-0.77	-0.93	< 0.1
Ralgapb	ENSMUST00000109486.3	Ral GTPase activating protein, beta subunit (non-	957	4	1	2	1	3	mmu-miR-214-3p	0.0528	0.347	1.5754	-0.3	-0.32	< 0.1
1600014C10Rik	ENSMUST00000165308.2	RIKEN cDNA 1600014C10 gene	1191	4	1	2	1	1	mmu-miR-214-3p	0.051	0.346	1.6466	-0.74	-0.74	< 0.1
Csf1	ENSMUST00000118593.2	colony stimulating factor 1 (macrophage)	1560	2	0	0	2	0	mmu-miR-214-3p	0.0544	0.3386	1.3278	-0.45	-0.45	< 0.1
Tfcp2l1	ENSMUST00000027629.9	transcription factor CP2-like 1	272	3	0	2	1	5	mmu-miR-214-3p	0.0482	0.3381	1.8874	-0.31	-0.31	< 0.1
Ammecr1l	ENSMUST00000115808.2	AMME chromosomal region gene 1-like	2444	3	2	1	0	1	mmu-miR-214-3p	0.0503	0.3369	1.4924	-0.46	-0.46	< 0.1
Celf1	ENSMUST00000068726.7	CUGBP, Elav-like family member 1	4386	4	0	3	1	1	mmu-miR-214-3p	0.0473	0.3345	1.923	-0.66	-0.68	< 0.1
Dcun1d1	ENSMUST00000178098.2	DCN1, defective in cullin neddylation 1, domain containing 1 (S. cerevisiae)	649	4	1	1	2	5	mmu-miR-214-3p	0.0551	0.3337	1.217	-0.37	-0.45	< 0.1
Zfand3	ENSMUST00000057897.7	zinc finger, AN1-type domain 3	2092	1	0	0	1	3	mmu-miR-214-3p	0.056	0.3315	1.1659	-0.57	-0.64	< 0.1
Hmg20a	ENSMUST00000034879.3	high mobility group 20A	416	3	1	1	1	2	mmu-miR-214-3p	0.049	0.3291	1.6065	-0.47	-0.47	< 0.1
1700052N19Rik	ENSMUST00000118544.2	RIKEN cDNA 1700052N19 gene	87	4	1	1	2	1	mmu-miR-214-3p	0.0493	0.3274	1.5019	-0.36	-0.38	< 0.1
Rapgefl1	ENSMUST00000107479.2	Rap guanine nucleotide exchange factor	18	1	0	1	0	3	mmu-miR-214-3p	0.051	0.3257	1.3537	-0.33	-0.34	< 0.1

Rab14	ENSMUST00000113025.1	RAB14, member RAS oncogene family	6563	3	1	1	1	4	mmu-miR-214-3p	0.0474	0.325	1.6857	-0.59	-0.67	< 0.1
Mfap3	ENSMUST00000108849.2	microfibrillar-associated protein 3	118	3	1	1	1	0	mmu-miR-214-3p	0.0533	0.3249	1.2021	-0.31	-0.35	< 0.1
Taf8	ENSMUST00000067103.2	TAF8 RNA polymerase II, TATA box binding protein (TBP)-associated factorq	146	4	2	2	0	3	mmu-miR-214-3p	0.0471	0.3247	1.6639	-0.93	-0.93	< 0.1
Glyctk	ENSMUST00000036382.7	glycerate kinase	249	2	0	1	1	1	mmu-miR-214-3p	0.0492	0.3225	1.3816	-0.3	-0.33	< 0.1
Mrrf	ENSMUST00000028250.3	mitochondrial ribosome recycling factor	242	3	1	1	1	3	mmu-miR-214-3p	0.0472	0.3224	1.6026	-0.35	-0.43	< 0.1
Wfikkn1	ENSMUST00000095487.5	WAP, FS, Ig, KU, and NTR-containing protein 1	9	0	0	0	0	0	mmu-miR-214-3p	0.057	0.3221	0.9569	-0.69	-0.69	< 0.1
Gpr156	ENSMUST00000061274.7	G protein-coupled receptor 156	12	4	0	2	2	2	mmu-miR-214-3p	0.0456	0.3209	1.8012	-0.47	-0.47	< 0.1
Qsox1	ENSMUST00000035325.9	quiescin Q6 sulfhydryl oxidase 1	1530	3	1	1	1	1	mmu-miR-214-3p	0.0512	0.3196	1.293	-0.49	-0.51	< 0.1
Ackr2	ENSMUST00000050327.3	atypical chemokine receptor 2	62	3	1	1	1	3	mmu-miR-214-3p	0.0495	0.3187	1.3658	-0.76	-0.92	< 0.1
Ndc1	ENSMUST00000139560.2	NDC1 transmembrane nucleoporin	1548	3	1	2	0	1	mmu-miR-214-3p	0.0462	0.3187	1.6338	-0.46	-0.49	< 0.1
Map6d1	ENSMUST00000040880.7	MAP6 domain containing 1	7	4	1	3	0	3	mmu-miR-214-3p	0.0451	0.317	1.7544	-0.53	-0.53	< 0.1
C2cd4c	ENSMUST00000178228.1	C2 calcium-dependent domain containing 4C	11	3	1	2	0	8	mmu-miR-214-3p	0.0479	0.3158	1.4869	-0.64	-0.88	< 0.1

Fam163a	ENSMUST00000015628.3	family with sequence similarity 163, member A	11	3	1	1	1	1	mmu-miR-214-3p	0.0483	0.3152	1.3648	-0.66	-0.66	< 0.1
Tgoln1	ENSMUST00000070524.4	trans-golgi network protein	3978	2	0	1	1	0	mmu-miR-214-3p	0.0473	0.3142	1.4393	-0.77	-0.83	< 0.1
Htr1a	ENSMUST00000022235.4	5-hydroxytryptamine (serotonin) receptor	5	3	0	2	1	2	mmu-miR-214-3p	0.0452	0.3126	1.6098	-0.52	-0.52	< 0.1
Lonrf2	ENSMUST00000147695.2	LON peptidase N-terminal domain and ring finger 2	29	3	2	1	0	0	mmu-miR-214-3p	0.0466	0.3126	1.4155	-0.42	-0.5	< 0.1
Fbln5	ENSMUST00000021603.8	fibulin 5	2038	1	0	1	0	0	mmu-miR-214-3p	0.0502	0.3115	1.1806	-0.69	-0.73	< 0.1
Ankrd52	ENSMUST00000014642.4	ankyrin repeat domain 52	1083	4	0	3	1	2	mmu-miR-214-3p	0.0431	0.3103	1.9457	-0.43	-0.44	< 0.1
6430573F11Rik	ENSMUST00000135373.2	RIKEN cDNA 6430573F11 gene	43	3	1	2	0	2	mmu-miR-214-3p	0.0469	0.3082	1.3774	-0.65	-0.65	< 0.1
Wnt2b	ENSMUST00000029429.4	wingless related MMTV integration site 2b	74	2	1	0	1	3	mmu-miR-214-3p	0.0459	0.3082	1.4973	-0.38	-0.41	< 0.1
Sec24c	ENSMUST00000048657.8	Sec24 related gene family, member C (S. cerevisiae)	3852	1	1	0	0	0	mmu-miR-214-3p	0.0502	0.3078	1.1576	-0.35	-0.35	< 0.1
6330416G13Rik	ENSMUST00000077709.5	RIKEN cDNA 6330416G13 gene	252	3	1	2	0	1	mmu-miR-214-3p	0.0479	0.307	1.2367	-0.67	-1.03	< 0.1
Snhg11	ENSMUST00000109488.2	small nucleolar RNA host gene 11	826	4	0	2	2	3	mmu-miR-214-3p	0.043	0.301	1.63	-0.32	-0.33	< 0.1
Gm17296	ENSMUST00000170518.1	predicted gene, 17296	125	2	1	1	0	0	mmu-miR-214-3p	0.0443	0.2994	1.5088	-0.35	-0.4	< 0.1

Cpsf4	ENSMUST00000160629.2	cleavage and polyadenylation specific factor 4	474	3	1	1	1	1	mmu-miR-214-3p	0.0454	0.2989	1.3089	-0.88	-0.92	< 0.1
Slc16a1	ENSMUST00000046212.1	solute carrier family 16 (monocarboxylic acid transporters), member 1	1864	3	0	2	1	1	mmu-miR-214-3p	0.0439	0.2981	1.4135	-0.45	-0.45	< 0.1
Mylk	ENSMUST00000023538.8	myosin, light polypeptide kinase	4565	1	0	1	0	2	mmu-miR-214-3p	0.042	0.2975	1.7488	-0.49	-0.49	< 0.1
Gm20708	ENSMUST00000132298.1	predicted gene 20708	185	4	2	1	1	0	mmu-miR-214-3p	0.0458	0.2974	1.2292	-0.72	-0.89	< 0.1
Zfp174	ENSMUST00000041778.7	zinc finger protein 174	81	1	1	0	0	3	mmu-miR-214-3p	0.0474	0.2933	1.1502	-0.37	-0.43	< 0.1
Cbx2	ENSMUST00000026662.7	chromobox 2	84	3	1	2	0	0	mmu-miR-214-3p	0.0438	0.2918	1.3397	-0.53	-0.53	< 0.1
Ercc2	ENSMUST00000062831.10	excision repair cross-complementing rodent repair deficiency, complementation	196	4	1	2	1	0	mmu-miR-214-3p	0.0418	0.2915	1.5548	-0.45	-0.46	< 0.1
Zfp710	ENSMUST00000166250.2	zinc finger protein 710	107	1	0	1	0	0	mmu-miR-214-3p	0.0457	0.2841	1.105	-0.33	-0.35	< 0.1
Acvr1b	ENSMUST00000000544.10	activin A receptor, type 1B	815	4	1	2	1	3	mmu-miR-214-3p	0.04	0.2841	1.6431	-0.3	-0.33	< 0.1
Cma1	ENSMUST00000022834.6	chymase 1, mast cell	20	1	1	0	0	0	mmu-miR-214-3p	0.0493	0.2835	0.8422	-0.6	-0.6	< 0.1
Cmah	ENSMUST00000167746.2	cytidine monophospho-N-acetylneuraminic acid hydroxylase	408	3	0	0	3	0	mmu-miR-214-3p	0.0401	0.2833	1.6102	-0.5	-0.51	< 0.1

Rassf5	ENSMUST00000112442.1	Ras association (RalGDS/AF-6) domain family member 5	26	3	0	2	1	3	mmu-miR-214-3p	0.0404	0.283	1.5529	-0.4	-0.41	< 0.1
Gm7932	ENSMUST00000101425.3	predicted gene 7932	23	1	1	0	0	0	mmu-miR-214-3p	0.0487	0.2813	0.8453	-0.62	-0.62	< 0.1
Gm15737	ENSMUST00000113434.1	predicted gene 15737	10	2	1	1	0	2	mmu-miR-214-3p	0.0412	0.28	1.4057	-0.46	-0.46	< 0.1
Adat3	ENSMUST00000178231.1	adenosine deaminase, tRNA-specific 3	3472	2	1	1	0	3	mmu-miR-214-3p	0.0415	0.2785	1.3491	-0.5	-0.51	< 0.1
Fkbp15	ENSMUST00000084527.4	FK506 binding protein 15	1383	1	1	0	0	2	mmu-miR-214-3p	0.0428	0.2782	1.2268	-0.34	-0.34	< 0.1
Kmt2d	ENSMUST00000178486.3	lysine (K)-specific methyltransferase 2D	273	2	0	2	0	2	mmu-miR-214-3p	0.0381	0.2781	1.8783	-0.56	-0.62	< 0.1
Cdk5r1	ENSMUST00000053413.11	cyclin-dependent kinase 5, regulatory subunit 1 (p35)	71	4	0	2	2	0	mmu-miR-214-3p	0.0396	0.2781	1.5198	-0.31	-0.31	< 0.1
Wnt2	ENSMUST00000010941.3	wingless-related MMTV integration site 2	86	2	1	1	0	0	mmu-miR-214-3p	0.0446	0.2779	1.0537	-0.49	-0.49	< 0.1
Tmem130	ENSMUST00000061446.7	transmembrane protein 130	9	1	1	0	0	0	mmu-miR-214-3p	0.0462	0.2777	0.9554	-0.44	-0.44	< 0.1
Lipg	ENSMUST00000066532.4	lipase, endothelial	16	3	0	1	2	2	mmu-miR-214-3p	0.0407	0.2775	1.347	-0.34	-0.34	< 0.1
Nos1ap	ENSMUST00000179801.1	nitric oxide synthase 1 (neuronal) adaptor protein	89	2	1	0	1	3	mmu-miR-214-3p	0.043	0.2772	1.1806	-0.53	-0.53	< 0.1
Tbc1d5	ENSMUST00000024717.8	TBC1 domain family, member 5	569	4	1	1	2	1	mmu-miR-214-3p	0.0393	0.2761	1.5199	-0.36	-0.46	< 0.1

Wdr82	ENSMUST00000020490.6	WD repeat domain containing 82	2078	5	2	2	1	1	mmu-miR-214-3p	0.0402	0.2744	1.3511	-0.4	-0.53	< 0.1
Kif13b	ENSMUST00000100473.4	kinesin family member 13B	841	2	0	1	1	1	mmu-miR-214-3p	0.0387	0.2738	1.5961	-0.31	-0.31	< 0.1
Psmd11	ENSMUST00000017572.8	proteasome (prosome, macropain) 26S subunit, non-ATPase, 11	847	4	0	4	0	3	mmu-miR-214-3p	0.0389	0.2734	1.5237	-0.38	-0.4	< 0.1
Dpy19l4	ENSMUST00000084892.6	dpy-19-like 4 (C. elegans)	673	2	1	1	0	2	mmu-miR-214-3p	0.0406	0.2725	1.2951	-0.3	-0.31	< 0.1
Fuca2	ENSMUST00000121465.2	fucosidase, alpha-L- 2, plasma	8314	2	1	0	1	0	mmu-miR-214-3p	0.0476	0.2722	0.7739	-0.46	-0.47	< 0.1
Cox17	ENSMUST00000120495.1	cytochrome c oxidase assembly protein 17	280	3	1	1	1	0	mmu-miR-214-3p	0.0398	0.2719	1.3613	-0.59	-0.59	< 0.1
Pbx4	ENSMUST00000081503.7	pre B cell leukemia homeobox 4	76	1	1	0	0	0	mmu-miR-214-3p	0.0476	0.2719	0.769	-0.51	-0.51	< 0.1
D430019H16Rik	ENSMUST00000178224.1	RIKEN cDNA D430019H16 gene	154	5	0	4	1	2	mmu-miR-214-3p	0.039	0.2713	1.4543	-0.39	-0.57	< 0.1
Cmtm6	ENSMUST00000035007.8	CKLF-like MARVEL transmembrane domain containing	1857	3	0	1	2	4	mmu-miR-214-3p	0.0379	0.2706	1.6181	-0.57	-0.69	< 0.1
Ppp2r4	ENSMUST00000113601.4	protein phosphatase 2A activator, regulatory	214	2	2	0	0	1	mmu-miR-214-3p	0.0415	0.2697	1.131	-0.7	-0.71	< 0.1
Rgag4	ENSMUST00000113631.1	retrotransposon gag domain containing 4	29	4	0	3	1	3	mmu-miR-214-3p	0.0383	0.2694	1.5054	-0.3	-0.3	< 0.1
Jup	ENSMUST00000001592.9	junction plakoglobin	208	4	1	2	1	2	mmu-miR-214-3p	0.0419	0.2691	1.1162	-0.39	-0.51	< 0.1

H2-Ke6	ENSMUST00000045467.8	H2-K region expressed gene 6	390	1	1	0	0	0	mmu-miR-214-3p	0.0462	0.2675	0.7897	-0.55	-0.55	< 0.1
BC026585	ENSMUST00000119891.1	cDNA sequence BC026585	127	3	1	1	1	3	mmu-miR-214-3p	0.0408	0.2669	1.1627	-0.65	-0.97	< 0.1
Sv2a	ENSMUST00000035371.8	synaptic vesicle glycoprotein 2 a	46	3	0	2	1	0	mmu-miR-214-3p	0.0399	0.2669	1.2332	-0.46	-0.47	< 0.1
Kpna1	ENSMUST00000004054.7	karyopherin (importin) alpha 1	5043	2	0	1	1	2	mmu-miR-214-3p	0.0415	0.2655	1.1158	-0.32	-0.39	< 0.1
St8sia1	ENSMUST00000032421.3	ST8 alpha-N-acetyl-neuraminide alpha-2,8-	150	1	1	0	0	4	mmu-miR-214-3p	0.0424	0.2642	1.0568	-0.32	-0.4	< 0.1
Myoz3	ENSMUST00000056533.7	myozenin 3	2514	2	1	0	1	0	mmu-miR-214-3p	0.0409	0.2633	1.0271	-0.39	-0.39	< 0.1
Foxp2	ENSMUST00000115477.2	forkhead box P2	105	2	0	1	1	2	mmu-miR-214-3p	0.0372	0.2605	1.407	-0.46	-0.59	< 0.1
Dynlt1c	ENSMUST00000092966.4	dynein light chain Tctex-type 1C	5	3	1	2	0	2	mmu-miR-214-3p	0.0376	0.2595	1.3535	-0.62	-0.62	< 0.1
Amfr	ENSMUST00000053766.7	autocrine motility factor receptor	7076	5	1	2	2	2	mmu-miR-214-3p	0.0368	0.2595	1.4774	-0.52	-0.91	< 0.1
Fgd5	ENSMUST00000089334.3	FYVE, RhoGEF and PH domain containing 5	364	2	1	1	0	0	mmu-miR-214-3p	0.0411	0.2592	1.0216	-0.36	-0.36	< 0.1
Pml	ENSMUST00000085673.5	promyelocytic leukemia	812	0	0	0	0	4	mmu-miR-214-3p	0.0408	0.2583	1.0335	-0.31	-0.32	< 0.1
E130309D14Rik	ENSMUST00000100866.2	RIKEN cDNA E130309D14 gene	39	2	1	0	1	1	mmu-miR-214-3p	0.0417	0.2563	0.9699	-0.37	-0.43	< 0.1

Atp6v0a1	ENSMUST00000044721.7	ATPase, H+ transporting, lysosomal V0 subunit A1	837	3	0	3	0	1	mmu-miR-214-3p	0.0365	0.2557	1.3886	-0.52	-0.52	< 0.1
Grin1	ENSMUST00000114310.4	glutamate receptor, ionotropic, NMDA1 (zeta 1)	7	2	0	2	0	1	mmu-miR-214-3p	0.0367	0.2557	1.3918	-0.39	-0.4	< 0.1
Map3k4	ENSMUST00000089058.5	mitogen-activated protein kinase kinase kinase 4	390	1	1	0	0	1	mmu-miR-214-3p	0.038	0.2525	1.1689	-0.35	-0.35	< 0.1
Tmem248	ENSMUST00000065329.7	transmembrane protein 248	1139	0	0	0	0	0	mmu-miR-214-3p	0.0423	0.2524	0.8114	-0.55	-0.55	< 0.1
Rbfox1	ENSMUST00000056416.7	RNA binding protein, fox-1 homolog (C.	709	3	0	1	2	0	mmu-miR-214-3p	0.0371	0.2522	1.2053	-0.36	-0.39	< 0.1
Stx12	ENSMUST00000030698.4	syntaxin 12	1629	1	1	0	0	1	mmu-miR-214-3p	0.0414	0.2516	0.8594	-0.45	-0.46	< 0.1
Nras	ENSMUST00000029445.7	neuroblastoma ras oncogene	1567	4	0	3	1	4	mmu-miR-214-3p	0.0361	0.2509	1.3468	-0.49	-0.87	< 0.1
Tubgcp4	ENSMUST00000110658.2	tubulin, gamma complex associated protein 4	1090	4	1	1	2	0	mmu-miR-214-3p	0.0367	0.2509	1.2478	-0.42	-0.49	< 0.1
Carns1	ENSMUST00000167055.1	carnosine synthase 1	275	1	1	0	0	0	mmu-miR-214-3p	0.0392	0.2508	1.0468	-0.33	-0.33	< 0.1
Slc9a8	ENSMUST00000047815.7	solute carrier family 9 (sodium/hydrogen exchanger),	247	2	0	0	2	5	mmu-miR-214-3p	0.0355	0.2489	1.3866	-0.49	-1.06	< 0.1
Fam198a	ENSMUST00000043011.7	family with sequence similarity 198, member A	81	2	1	0	1	0	mmu-miR-214-3p	0.0366	0.2471	1.2089	-0.43	-0.46	< 0.1
Btbd11	ENSMUST00000105307.2	BTB (POZ) domain containing 11	120	2	1	1	0	0	mmu-miR-214-3p	0.0395	0.2471	0.9458	-0.3	-0.31	< 0.1

Def8	ENSMUST00000065534.4	differentially expressed in FDCP 8	1168	8	3	3	2	4	mmu-miR-214-3p	0.038	0.2471	1.0597	-0.3	-0.74	< 0.1
Cxcr2	ENSMUST00000106899.3	chemokine (C-X-C motif) receptor 2	10	1	1	0	0	0	mmu-miR-214-3p	0.0372	0.2467	1.083	-0.37	-0.37	< 0.1
Igf1r	ENSMUST00000005671.8	insulin-like growth factor I receptor	2095	2	0	1	1	4	mmu-miR-214-3p	0.0339	0.2449	1.5638	-0.4	-0.43	< 0.1
Cmklr1	ENSMUST00000047936.7	chemokine-like receptor 1	1165	2	1	0	1	7	mmu-miR-214-3p	0.0346	0.2436	1.3934	-0.44	-0.65	< 0.1
Zfp276	ENSMUST00000001092.9	zinc finger protein (C2H2 type) 276	297	4	0	3	1	3	mmu-miR-214-3p	0.0346	0.2427	1.3245	-0.37	-0.38	< 0.1
Best3	ENSMUST00000020378.4	bestrophin 3	174	1	0	0	1	2	mmu-miR-214-3p	0.0332	0.2422	1.6315	-0.98	-1.27	< 0.1
Rnf185	ENSMUST00000077078.6	ring finger protein 185	336	3	1	1	1	1	mmu-miR-214-3p	0.0347	0.2418	1.2979	-0.53	-0.57	< 0.1
Krtap10-4	ENSMUST00000092369.3	keratin associated protein 10-4	5	1	1	0	0	0	mmu-miR-214-3p	0.0333	0.2417	1.6003	-0.4	-0.4	< 0.1
Zc2hc1b	ENSMUST00000019954.4	zinc finger, C2HC-type containing 1B	5	2	1	1	0	2	mmu-miR-214-3p	0.0375	0.2415	1.0031	-0.69	-0.69	< 0.1
Ikbkb	ENSMUST00000063401.4	inhibitor of kappaB kinase beta	4750	2	0	2	0	2	mmu-miR-214-3p	0.0342	0.2405	1.3537	-0.54	-0.54	< 0.1
Tor4a	ENSMUST00000081869.6	torsin family 4, member A	141	3	1	1	1	1	mmu-miR-214-3p	0.0353	0.2404	1.1719	-0.44	-0.53	< 0.1
9130011E15Rik	ENSMUST00000045396.7	RIKEN cDNA 9130011E15 gene	285	3	0	3	0	3	mmu-miR-214-3p	0.0345	0.2397	1.285	-0.32	-0.34	< 0.1

Arhgef10l	ENSMUST00000069623.6	Rho guanine nucleotide exchange factor	313	2	1	1	0	2	mmu-miR-214-3p	0.0384	0.2393	0.9085	-0.31	-0.37	< 0.1
Olfr60	ENSMUST00000075470.1	olfactory receptor 60	1	1	1	0	0	0	mmu-miR-214-3p	0.0398	0.2392	0.775	-0.39	-0.39	< 0.1
Bsnd	ENSMUST00000054472.3	Bartter syndrome, infantile, with sensorineural deafness (Barttin)	23	4	0	0	4	3	mmu-miR-214-3p	0.0332	0.2389	1.4791	-0.65	-0.67	< 0.1
Slc38a3	ENSMUST00000167868.2	solute carrier family 38, member 3	2700	4	1	2	1	1	mmu-miR-214-3p	0.036	0.2375	1.1369	-0.45	-0.9	< 0.1
Med22	ENSMUST00000102899.4	mediator complex subunit 22	351	4	0	3	1	2	mmu-miR-214-3p	0.0337	0.2375	1.3344	-0.31	-0.32	< 0.1
Med19	ENSMUST00000102645.3	mediator complex subunit 19	642	2	0	2	0	0	mmu-miR-214-3p	0.0397	0.2373	0.7515	-0.6	-0.84	< 0.1
Sec16b	ENSMUST00000086130.3	SEC16 homolog B (S. cerevisiae)	833	2	0	2	0	0	mmu-miR-214-3p	0.0341	0.2366	1.2276	-0.38	-0.38	< 0.1
2010109I03Rik	ENSMUST00000077004.5	RIKEN cDNA 2010109I03 gene	6	2	1	0	1	1	mmu-miR-214-3p	0.0353	0.236	1.1066	-0.49	-0.49	< 0.1
Prrg3	ENSMUST00000048790.6	proline rich Gla (G-carboxyglutamic acid) 3 (transmembrane)	304	2	0	1	1	3	mmu-miR-214-3p	0.0338	0.2355	1.2591	-0.39	-0.43	< 0.1
Mfn2	ENSMUST00000105716.3	mitofusin 2	10277	3	0	2	1	0	mmu-miR-214-3p	0.0335	0.2336	1.224	-0.31	-0.32	< 0.1
Wscd1	ENSMUST00000021168.8	WSC domain containing 1	716	1	1	0	0	2	mmu-miR-214-3p	0.0362	0.2326	0.9488	-0.49	-0.59	< 0.1

Ogfod3	ENSMUST00000026169.6	2-oxoglutarate and iron-dependent oxygenase domain containing 3	649	1	1	0	0	1	mmu-miR-214-3p	0.0405	0.2315	0.6573	-0.62	-0.75	< 0.1
Tph1	ENSMUST00000107669.3	tryptophan hydroxylase 1	17	1	1	0	0	1	mmu-miR-214-3p	0.036	0.2314	0.9578	-0.55	-0.58	< 0.1
Myrip	ENSMUST00000048121.7	myosin VIIA and Rab interacting protein	95	2	1	0	1	2	mmu-miR-214-3p	0.0346	0.2314	1.1039	-0.44	-0.47	< 0.1
Rnf224	ENSMUST00000091318.4	ring finger protein 224	5	3	0	2	1	1	mmu-miR-214-3p	0.0333	0.2313	1.2434	-0.44	-0.44	< 0.1
Cd247	ENSMUST00000005907.6	CD247 antigen	44	2	1	1	0	0	mmu-miR-214-3p	0.0344	0.2298	1.0134	-0.69	-0.69	< 0.1
Vps11	ENSMUST00000034644.8	vacuolar protein sorting 11 (yeast)	325	2	1	1	0	0	mmu-miR-214-3p	0.0349	0.2295	1.0184	-0.8	-0.81	< 0.1
Lsm12	ENSMUST00000107156.3	LSM12 homolog (S. cerevisiae)	3995	2	1	0	1	0	mmu-miR-214-3p	0.0353	0.2294	0.9413	-0.84	-0.99	< 0.1
1700021K19Rik	ENSMUST00000115105.3	RIKEN cDNA 1700021K19 gene	816	3	0	1	2	1	mmu-miR-214-3p	0.0327	0.2294	1.2666	-0.4	-0.41	< 0.1
Tgfa	ENSMUST00000032066.9	transforming growth factor alpha	454	4	0	4	0	2	mmu-miR-214-3p	0.0322	0.2287	1.3352	-0.31	-0.31	< 0.1
Cgn	ENSMUST00000107273.2	cingulin	157	1	1	0	0	0	mmu-miR-214-3p	0.0368	0.2286	0.8473	-0.31	-0.33	< 0.1
Rnf152	ENSMUST00000058688.6	ring finger protein 152	313	3	0	3	0	2	mmu-miR-214-3p	0.032	0.2285	1.3892	-0.33	-0.34	< 0.1
Pla2g2f	ENSMUST00000030526.6	phospholipase A2, group IIF	5	2	1	1	0	1	mmu-miR-214-3p	0.0351	0.2284	0.9707	-0.5	-0.5	< 0.1

Amigo3	ENSMUST00000085060.2	adhesion molecule with Ig like domain 3	49	3	0	3	0	2	mmu-miR-214-3p	0.0324	0.2277	1.2746	-0.58	-0.58	< 0.1
Sfxn2	ENSMUST00000026011.6	sideroflexin 2	29	2	1	1	0	0	mmu-miR-214-3p	0.0331	0.227	1.1398	-0.43	-0.43	< 0.1
Rnaset2b	ENSMUST00000179728.1	ribonuclease T2B	5	2	2	0	0	0	mmu-miR-214-3p	0.0335	0.2265	1.0757	-0.56	-0.56	< 0.1
Rcsd1	ENSMUST00000040357.9	RCSD domain containing 1	194	1	0	0	1	0	mmu-miR-214-3p	0.0388	0.2264	0.6947	-0.38	-0.56	< 0.1
Fam216b	ENSMUST00000066437.4	family with sequence similarity 216, member B	42	3	1	1	1	2	mmu-miR-214-3p	0.0323	0.2247	1.1778	-0.6	-0.65	< 0.1
Lypd1	ENSMUST00000159417.1	Ly6/Plaur domain containing 1	6	2	0	1	1	3	mmu-miR-214-3p	0.0311	0.2242	1.4086	-0.36	-0.38	< 0.1
Map2k4	ENSMUST00000046963.4	mitogen-activated protein kinase kinase 4	1573	1	1	0	0	3	mmu-miR-214-3p	0.0341	0.2238	0.9988	-0.3	-0.32	< 0.1
Ccdc96	ENSMUST00000060100.2	coiled-coil domain containing 96	70	2	1	0	1	0	mmu-miR-214-3p	0.0359	0.2235	0.8007	-0.46	-0.5	< 0.1
Ldlrap1	ENSMUST00000037828.6	low density lipoprotein receptor adaptor protein 1	567	5	0	3	2	5	mmu-miR-214-3p	0.0324	0.2229	1.1241	-0.33	-0.55	< 0.1
Pdk1	ENSMUST00000006669.5	pyruvate dehydrogenase kinase, isoenzyme 1	2787	3	0	1	2	1	mmu-miR-214-3p	0.0315	0.2223	1.2372	-0.37	-0.39	< 0.1
Rexo2	ENSMUST00000034524.3	REX2, RNA exonuclease 2 homolog (S. cerevisiae)	280	2	1	1	0	0	mmu-miR-214-3p	0.0337	0.2207	0.9024	-0.9	-0.95	< 0.1

Smarcd1	ENSMUST00000023759.4	SWI/SNF related, matrix associated, actin dependent regulator of chromatin, subfamily d,	1422	2	0	2	0	0	mmu-miR-214-3p	0.0318	0.22	1.1125	-0.45	-0.45	< 0.1
Fbxo41	ENSMUST00000159062.2	F-box protein 41	5	4	0	4	0	0	mmu-miR-214-3p	0.0304	0.2194	1.4025	-0.45	-0.45	< 0.1
Mapk9	ENSMUST00000109179.3	mitogen-activated protein kinase 9	1683	2	1	0	1	3	mmu-miR-214-3p	0.0325	0.2185	1.0506	-0.31	-0.38	< 0.1
Ldoc1	ENSMUST00000075983.5	leucine zipper, down-regulated in cancer 1	13	3	0	3	0	0	mmu-miR-214-3p	0.0315	0.2175	1.0934	-0.7	-0.7	< 0.1
Nr1h5	ENSMUST00000058899.8	nuclear receptor subfamily 1, group H, member 5	209	1	1	0	0	0	mmu-miR-214-3p	0.0358	0.2172	0.7197	-0.4	-0.42	< 0.1
Usp46	ENSMUST00000119154.2	ubiquitin specific peptidase 46	244	2	2	0	0	0	mmu-miR-214-3p	0.0337	0.2171	0.836	-0.34	-0.46	< 0.1
2900026A02Rik	ENSMUST00000050125.8	RIKEN cDNA 2900026A02 gene	671	3	0	2	1	1	mmu-miR-214-3p	0.0309	0.2169	1.2048	-0.35	-0.47	< 0.1
Wdtc1	ENSMUST00000105906.1	WD and tetratricopeptide repeats 1	108	5	1	3	1	2	mmu-miR-214-3p	0.0307	0.2168	1.2358	-0.3	-0.4	< 0.1
Ptpn9	ENSMUST00000034832.6	protein tyrosine phosphatase, non-receptor type 9	253	3	1	1	1	2	mmu-miR-214-3p	0.0307	0.2165	1.213	-0.58	-0.94	< 0.1
Plagl2	ENSMUST00000056924.8	pleiomorphic adenoma gene-like 2	205	3	0	2	1	1	mmu-miR-214-3p	0.0307	0.2164	1.2161	-0.31	-0.32	< 0.1
Plekhj1	ENSMUST00000036805.5	pleckstrin homology domain containing, family J	148	0	0	0	0	1	mmu-miR-214-3p	0.0362	0.2162	0.6746	-0.45	-0.49	< 0.1

Lrrc61	ENSMUST00000114545.2	leucine rich repeat containing 61	318	3	0	3	0	0	mmu-miR-214-3p	0.0302	0.2161	1.3086	-0.46	-0.47	< 0.1
Klhl18	ENSMUST00000111933.2	kelch-like 18	238	4	1	3	0	2	mmu-miR-214-3p	0.0303	0.2161	1.2901	-0.3	-0.3	< 0.1
Pcbp4	ENSMUST00000024260.7	poly(rC) binding protein 4	700	3	0	3	0	0	mmu-miR-214-3p	0.0313	0.2159	1.0871	-0.62	-0.62	< 0.1
Med20	ENSMUST00000024778.2	mediator complex subunit 20	1858	3	1	1	1	0	mmu-miR-214-3p	0.0326	0.2152	0.9346	-0.47	-0.51	< 0.1
Nipal4	ENSMUST00000020679.2	NIPA-like domain containing 4	18	1	1	0	0	1	mmu-miR-214-3p	0.0329	0.2151	0.9268	-0.34	-0.34	< 0.1
Slc11a2	ENSMUST00000138843.2	solute carrier family 11 (proton-coupled divalent metal ion transporters), member 2	799	2	1	1	0	3	mmu-miR-214-3p	0.0305	0.2144	1.2178	-0.36	-0.45	< 0.1
Lims1	ENSMUST00000020077.5	LIM and senescent cell antigen-like domains 1	8422	1	0	0	1	3	mmu-miR-214-3p	0.0309	0.2142	1.1573	-0.34	-0.34	< 0.1
Dolpp1	ENSMUST00000028209.9	dolichyl pyrophosphate phosphatase 1	745	4	0	4	0	2	mmu-miR-214-3p	0.0303	0.214	1.2246	-0.38	-0.38	< 0.1
Naa30	ENSMUST00000037362.5	N(alpha)-acetyltransferase 30, NatC catalytic subunit	980	4	0	1	3	1	mmu-miR-214-3p	0.0309	0.214	1.1093	-0.33	-0.41	< 0.1
Esrrb	ENSMUST00000116402.4	estrogen related receptor, beta	130	2	0	1	1	1	mmu-miR-214-3p	0.0302	0.214	1.2573	-0.33	-0.52	< 0.1
Glis2	ENSMUST00000014447.7	GLIS family zinc finger 2	406	3	0	2	1	2	mmu-miR-214-3p	0.0298	0.2133	1.2961	-0.46	-0.46	< 0.1

Hdgf	ENSMUST00000005017.9	hepatoma-derived growth factor	18948	4	0	3	1	2	mmu-miR-214-3p	0.0299	0.2127	1.2377	-0.59	-0.59	< 0.1
Nomo1	ENSMUST00000033121.6	nodal modulator 1	294	1	1	0	0	1	mmu-miR-214-3p	0.034	0.2122	0.7696	-0.39	-0.4	< 0.1
Zfyve1	ENSMUST00000048319.4	zinc finger, FYVE domain containing 1	283	2	1	1	0	1	mmu-miR-214-3p	0.0345	0.2121	0.8054	-0.4	-0.64	< 0.1
Vgll1	ENSMUST00000033465.4	vestigial like 1 homolog (Drosophila)	5	3	0	3	0	1	mmu-miR-214-3p	0.0302	0.2119	1.1567	-0.74	-0.74	< 0.1
Adap1	ENSMUST00000110865.1	ArfGAP with dual PH domains 1	324	3	0	3	0	4	mmu-miR-214-3p	0.0302	0.2114	1.1509	-0.44	-0.44	< 0.1
Gdpd4	ENSMUST00000041860.7	glycerophosphodiester phosphodiesterase	5	1	1	0	0	0	mmu-miR-214-3p	0.0338	0.2113	0.7684	-0.31	-0.31	< 0.1
Vwde	ENSMUST00000054530.5	von Willebrand factor D and EGF domains	5	1	1	0	0	0	mmu-miR-214-3p	0.0338	0.2101	0.7543	-0.34	-0.34	< 0.1
Cldn18	ENSMUST00000136429.2	claudin 18	2574	2	1	0	1	0	mmu-miR-214-3p	0.0335	0.21	0.7697	-0.38	-0.38	< 0.1
Mpzl3	ENSMUST00000114664.2	myelin protein zero-like 3	453	1	1	0	0	0	mmu-miR-214-3p	0.0317	0.2097	0.9378	-0.31	-0.31	< 0.1
Crtac1	ENSMUST00000048630.6	cartilage acidic protein 1	9	2	1	1	0	2	mmu-miR-214-3p	0.0326	0.2094	0.8317	-0.34	-0.59	< 0.1
Hdac9	ENSMUST00000110819.2	histone deacetylase 9	21	2	0	1	1	1	mmu-miR-214-3p	0.0291	0.209	1.3002	-0.31	-0.31	< 0.1
Uhmk1	ENSMUST00000123399.1	U2AF homology motif (UHM) kinase 1	267	4	0	2	2	1	mmu-miR-214-3p	0.029	0.2088	1.316	-0.47	-0.59	< 0.1

Fam155a	ENSMUST00000110969.3	family with sequence similarity 155, member A	5	3	1	2	0	0	mmu-miR-214-3p	0.0317	0.2076	0.8851	-0.35	-0.35	< 0.1
Lmf2	ENSMUST00000023283.5	lipase maturation factor 2	859	1	1	0	0	0	mmu-miR-214-3p	0.0303	0.2072	1.063	-0.47	-0.47	< 0.1
Lmo3	ENSMUST00000162772.2	LIM domain only 3	6	1	1	0	0	1	mmu-miR-214-3p	0.031	0.2066	0.9529	-0.38	-0.39	< 0.1
Zbtb8b	ENSMUST00000106046.2	zinc finger and BTB domain containing 8b	17	1	1	0	0	0	mmu-miR-214-3p	0.0335	0.2065	0.6993	-0.33	-0.33	< 0.1
Gpr6	ENSMUST00000061796.6	G protein-coupled receptor 6	5	2	1	0	1	1	mmu-miR-214-3p	0.0309	0.2064	0.9593	-0.56	-0.56	< 0.1
Rasgef1c	ENSMUST00000063444.2	RasGEF domain family, member 1C	14	3	0	2	1	0	mmu-miR-214-3p	0.0293	0.206	1.1247	-0.55	-0.55	< 0.1
Gm17567	ENSMUST00000164429.1	predicted gene, 17567	42	2	0	1	1	3	mmu-miR-214-3p	0.0286	0.2059	1.2917	-0.35	-0.45	< 0.1
Ezh1	ENSMUST00000107284.2	enhancer of zeste homolog 1 (Drosophila)	37	2	0	2	0	2	mmu-miR-214-3p	0.0296	0.2059	1.0953	-0.33	-0.34	< 0.1
5830411N06Rik	ENSMUST00000164583.2	RIKEN cDNA 5830411N06 gene	8	3	0	1	2	0	mmu-miR-214-3p	0.0292	0.2055	1.1597	-0.32	-0.32	< 0.1
RP23-180L12.5	ENSMUST00000189185.1		6	2	2	0	0	2	mmu-miR-214-3p	0.0297	0.2052	1.0664	-0.47	-0.51	< 0.1
Mcoln2	ENSMUST00000011152.8	mucolipin 2	17	2	1	0	1	0	mmu-miR-214-3p	0.0321	0.2048	0.8013	-0.5	-0.5	< 0.1
Cd300ld	ENSMUST00000045075.2	CD300 molecule-like family member d	11	1	1	0	0	0	mmu-miR-214-3p	0.0301	0.2047	0.9974	-0.57	-0.57	< 0.1

1700010I14Rik	ENSMUST00000151609.1	RIKEN cDNA 1700010I14 gene	24	1	1	0	0	0	mmu-miR-214-3p	0.0343	0.2047	0.6235	-0.51	-0.57	< 0.1
S100pbp	ENSMUST00000106061.3	S100P binding protein	727	4	2	1	1	2	mmu-miR-214-3p	0.0293	0.2044	1.1007	-0.3	-0.59	< 0.1
Gprc5a	ENSMUST00000050104.7	G protein-coupled receptor, family C, group 5, member A	642	1	1	0	0	0	mmu-miR-214-3p	0.0334	0.2039	0.6677	-0.37	-0.37	< 0.1
Me1	ENSMUST00000034989.9	malic enzyme 1, NADP(+)-dependent,	11509	2	1	1	0	1	mmu-miR-214-3p	0.0292	0.2038	1.091	-0.57	-0.57	< 0.1
Nckipsd	ENSMUST00000035218.7	NCK interacting protein with SH3 domain	67	4	0	3	1	3	mmu-miR-214-3p	0.0294	0.2038	1.0664	-0.31	-0.41	< 0.1
Aar2	ENSMUST00000029158.3	AAR2 splicing factor homolog (S. cerevisiae)	2676	2	0	2	0	3	mmu-miR-214-3p	0.0287	0.2035	1.1758	-0.68	-0.75	< 0.1
Sall4	ENSMUST00000075044.4	sal-like 4 (Drosophila)	337	1	0	1	0	0	mmu-miR-214-3p	0.03	0.2032	0.9461	-0.63	-0.63	< 0.1
Tmie	ENSMUST00000050958.4	transmembrane inner ear	43	0	0	0	0	0	mmu-miR-214-3p	0.0316	0.2032	0.8084	-0.36	-0.36	< 0.1
Pgf	ENSMUST00000004913.6	placental growth factor	33	1	0	1	0	0	mmu-miR-214-3p	0.0313	0.2029	0.8346	-0.58	-0.58	< 0.1
Ern1	ENSMUST00000001059.3	endoplasmic reticulum (ER) to nucleus signalling 1	832	1	0	0	1	4	mmu-miR-214-3p	0.028	0.2026	1.306	-0.33	-0.34	< 0.1
0610030E20Rik	ENSMUST00000077783.3	RIKEN cDNA 0610030E20 gene	1246	2	0	1	1	3	mmu-miR-214-3p	0.0287	0.2022	1.1351	-0.31	-0.33	< 0.1
Elfn2	ENSMUST00000088592.4	leucine rich repeat and fibronectin type III, extracellular 2	5	2	0	2	0	0	mmu-miR-214-3p	0.0289	0.2021	1.0902	-0.32	-0.32	< 0.1

Cpped1	ENSMUST00000096272.5	calcineurin-like phosphoesterase domain containing	139	2	1	0	1	1	mmu-miR-214-3p	0.0293	0.2017	1.0018	-0.32	-0.35	< 0.1
Epb4.1l1	ENSMUST00000103137.4	erythrocyte protein band 4.1-like 1	1725	2	1	0	1	1	mmu-miR-214-3p	0.0313	0.2016	0.8144	-0.31	-0.42	< 0.1
H2-Q6	ENSMUST00000174699.2	histocompatibility 2, Q region locus 6	5	3	0	2	1	0	mmu-miR-214-3p	0.0283	0.2014	1.1905	-0.59	-0.59	< 0.1
Haao	ENSMUST00000000687.7	3-hydroxyanthranilate 3,4-dioxygenase	1346	2	1	0	1	0	mmu-miR-214-3p	0.0325	0.2004	0.6938	-0.49	-0.71	< 0.1
Kctd15	ENSMUST00000108069.2	potassium channel tetramerisation domain containing 15	292	3	1	2	0	2	mmu-miR-214-3p	0.0281	0.2004	1.1898	-0.36	-0.36	< 0.1
Cdc42se2	ENSMUST00000064104.7	CDC42 small effector 2	5132	2	1	1	0	2	mmu-miR-214-3p	0.029	0.1999	1.0514	-0.37	-0.38	< 0.1
Shh	ENSMUST00000002708.3	sonic hedgehog	28	1	1	0	0	1	mmu-miR-214-3p	0.0296	0.1996	0.9549	-0.43	-0.47	< 0.1
Birc5	ENSMUST00000093906.4	baculoviral IAP repeat-containing 5	2096	4	1	2	1	0	mmu-miR-214-3p	0.0281	0.1986	1.1299	-0.37	-0.37	< 0.1
P2rx6	ENSMUST00000023441.5	purinergic receptor P2X, ligand-gated ion channel, 6	207	2	0	1	1	3	mmu-miR-214-3p	0.0286	0.1985	1.0666	-0.36	-0.37	< 0.1
Gm16401	ENSMUST00000072800.4	predicted gene 16401	6	1	1	0	0	0	mmu-miR-214-3p	0.0317	0.1984	0.7194	-0.43	-0.43	< 0.1
Zbtb42	ENSMUST00000174780.1	zinc finger and BTB domain containing 42	75	2	0	1	1	4	mmu-miR-214-3p	0.028	0.1982	1.141	-0.31	-0.31	< 0.1
Sidt1	ENSMUST00000127567.2	SID1 transmembrane family, member 1	6	2	0	2	0	0	mmu-miR-214-3p	0.0278	0.1981	1.1729	-0.36	-0.36	< 0.1

Fam134a	ENSMUST00000097694.5	family with sequence similarity 134, member A	2091	2	0	2	0	1	mmu-miR-214-3p	0.0279	0.1971	1.1182	-0.45	-0.52	< 0.1
5830454E08Rik	ENSMUST00000064646.4	RIKEN cDNA 5830454E08 gene	64	1	1	0	0	0	mmu-miR-214-3p	0.0318	0.197	0.6793	-0.62	-0.62	< 0.1
Pycr1	ENSMUST00000170556.2	pyrroline-5-carboxylate reductase 1	11	2	1	0	1	0	mmu-miR-214-3p	0.0302	0.1969	0.8383	-0.36	-0.49	< 0.1
Fam57a	ENSMUST00000094014.4	family with sequence similarity 57, member A	275	3	2	1	0	0	mmu-miR-214-3p	0.0293	0.1967	0.9369	-0.39	-0.55	< 0.1
Traf3ip1	ENSMUST00000047242.8	TRAF3 interacting protein 1	419	4	0	3	1	2	mmu-miR-214-3p	0.028	0.1963	1.0762	-0.41	-0.47	< 0.1
Prpsap1	ENSMUST00000106391.2	phosphoribosyl pyrophosphate synthetase-associated protein 1	2350	4	0	1	3	2	mmu-miR-214-3p	0.0279	0.1956	1.0745	-0.47	-0.56	< 0.1
Tmem234	ENSMUST00000102591.4	transmembrane protein 234	2084	2	0	2	0	1	mmu-miR-214-3p	0.0285	0.1949	0.9444	-0.52	-0.53	< 0.1
Tmem161b	ENSMUST00000057495.8	transmembrane protein 161B	1510	0	0	0	0	0	mmu-miR-214-3p	0.0315	0.1948	0.6592	-0.42	-0.42	< 0.1
Dhx35	ENSMUST00000029186.8	DEAH (Asp-Glu-Ala-His) box polypeptide 35	558	4	0	2	2	3	mmu-miR-214-3p	0.0285	0.1948	0.9947	-0.31	-0.5	< 0.1
Soga1	ENSMUST00000069098.6	suppressor of glucose, autophagy associated 1	473	4	1	2	1	3	mmu-miR-214-3p	0.0269	0.1946	1.2529	-0.42	-0.67	< 0.1
Gm12216	ENSMUST00000120776.2	predicted gene 12216	28	2	1	0	1	2	mmu-miR-214-3p	0.0304	0.1945	0.7592	-0.51	-0.56	< 0.1
Scara3	ENSMUST00000042046.3	scavenger receptor class A, member 3	33	4	0	3	1	0	mmu-miR-214-3p	0.0276	0.1944	1.0807	-0.38	-0.39	< 0.1

Atrip	ENSMUST00000045011.3	ATR interacting protein	55	1	1	0	0	0	mmu-miR-214-3p	0.0318	0.1936	0.6374	-0.55	-0.7	< 0.1
Fgfr1op	ENSMUST00000097419.4	Fgfr1 oncogene partner	776	3	0	2	1	0	mmu-miR-214-3p	0.0279	0.1935	1.0009	-0.37	-0.39	< 0.1
Slc30a10	ENSMUST00000061093.6	solute carrier family 30, member 10	291	2	1	1	0	1	mmu-miR-214-3p	0.0291	0.193	0.8519	-0.42	-0.46	< 0.1
Kif7	ENSMUST00000183846.2	kinesin family member 7	66	0	0	0	0	0	mmu-miR-214-3p	0.0295	0.1927	0.823	-0.35	-0.35	< 0.1
Ubap2l	ENSMUST00000090908.6	ubiquitin-associated protein 2-like	1835	1	0	1	0	0	mmu-miR-214-3p	0.0285	0.1921	0.8953	-0.51	-0.51	< 0.1
Smim5	ENSMUST00000152171.2	small integral membrane protein 5	110	2	1	0	1	1	mmu-miR-214-3p	0.0293	0.192	0.8195	-0.65	-0.69	< 0.1
Slc13a3	ENSMUST00000029208.9	solute carrier family 13 (sodium-dependent dicarboxylate transporter), member 3	116	2	0	1	1	2	mmu-miR-214-3p	0.0269	0.192	1.1652	-0.53	-0.55	< 0.1
Fezf1	ENSMUST00000031709.5	Fez family zinc finger 1	7	1	1	0	0	1	mmu-miR-214-3p	0.0283	0.1915	0.9123	-0.44	-0.44	< 0.1
Htt	ENSMUST00000080036.2	huntingtin	604	1	0	0	1	7	mmu-miR-214-3p	0.0262	0.1914	1.3046	-0.67	-1.12	< 0.1
Ttll5	ENSMUST00000040179.8	tubulin tyrosine ligase-like family, member 5	139	2	0	2	0	0	mmu-miR-214-3p	0.0274	0.1911	1.0184	-0.43	-0.43	< 0.1
Capn13	ENSMUST00000095208.3	calpain 13	5	1	1	0	0	0	mmu-miR-214-3p	0.0298	0.1905	0.7416	-0.38	-0.38	< 0.1

Zbtb39	ENSMUST00000054287.7	zinc finger and BTB domain containing 39	95	3	0	2	1	2	mmu-miR-214-3p	0.0267	0.1897	1.1112	-0.32	-0.41	< 0.1
Serping1	ENSMUST00000111641.1	serine (or cysteine) peptidase inhibitor, clade G, member 1	11522	1	1	0	0	0	mmu-miR-214-3p	0.0291	0.1895	0.7871	-0.46	-0.46	< 0.1
Lmna	ENSMUST00000029699.7	lamin A	221	2	0	2	0	0	mmu-miR-214-3p	0.0279	0.189	0.9196	-0.33	-0.33	< 0.1
Rrp7a	ENSMUST00000018184.4	ribosomal RNA processing 7 homolog A (S. cerevisiae)	22	5	1	2	2	3	mmu-miR-214-3p	0.0272	0.1889	0.9893	-0.32	-0.93	< 0.1
Psmg2	ENSMUST00000025418.3	proteasome (prosome, macropain) assembly	5850	1	1	0	0	0	mmu-miR-214-3p	0.0303	0.188	0.6401	-0.65	-0.65	< 0.1
Scp2d1	ENSMUST00000028918.3	SCP2 sterol-binding domain containing 1	5	0	0	0	0	0	mmu-miR-214-3p	0.0299	0.1855	0.6273	-0.69	-0.69	< 0.1
Rexo1	ENSMUST00000057910.10	REX1, RNA exonuclease 1 homolog (S. cerevisiae)	2073	2	1	1	0	0	mmu-miR-214-3p	0.0275	0.1855	0.882	-0.46	-0.47	< 0.1
Dok4	ENSMUST00000046461.7	docking protein 4	66	2	1	1	0	2	mmu-miR-214-3p	0.0264	0.1846	1.0058	-0.38	-0.47	< 0.1
Lat2	ENSMUST00000036362.7	linker for activation of T cells family, member 2	107	1	1	0	0	0	mmu-miR-214-3p	0.0291	0.1843	0.6855	-0.44	-0.44	< 0.1
Layn	ENSMUST00000098782.3	layilin	167	1	1	0	0	3	mmu-miR-214-3p	0.0284	0.1833	0.7383	-0.45	-0.48	< 0.1
Nxph3	ENSMUST00000058866.7	neurexophilin 3	43	1	1	0	0	0	mmu-miR-214-3p	0.0282	0.1832	0.7579	-0.33	-0.33	< 0.1

Tmem209	ENSMUST00000115160.4	transmembrane protein 209	692	1	1	0	0	0	mmu-miR-214-3p	0.0292	0.1826	0.6331	-0.44	-0.44	< 0.1
BC023829	ENSMUST00000101506.4	cDNA sequence BC023829	483	1	0	1	0	4	mmu-miR-214-3p	0.0253	0.1816	1.125	-0.42	-0.42	< 0.1
Chaf1a	ENSMUST00000002914.8	chromatin assembly factor 1, subunit A (p150)	535	2	1	0	1	1	mmu-miR-214-3p	0.0262	0.1814	0.9626	-0.77	-0.77	< 0.1
Kctd12	ENSMUST00000184744.1	potassium channel tetramerisation domain containing 12	904	3	0	2	1	2	mmu-miR-214-3p	0.0258	0.1812	0.994	-0.3	-0.33	< 0.1
Lrat	ENSMUST00000029632.6	lecithin-retinol acyltransferase (phosphatidylcholine-retinol-O-acyltransferase)	316	2	0	1	1	2	mmu-miR-214-3p	0.0275	0.1808	0.7798	-0.32	-0.32	< 0.1
Cyb5rl	ENSMUST00000106758.2	cytochrome b5 reductase-like	552	3	0	1	2	1	mmu-miR-214-3p	0.0259	0.1807	0.9785	-0.57	-0.61	< 0.1
Igdcc3	ENSMUST00000034961.4	immunoglobulin superfamily, DCC subclass, member 3	158	2	0	2	0	0	mmu-miR-214-3p	0.0258	0.1797	0.9591	-0.31	-0.31	< 0.1
Ube3c	ENSMUST00000049453.4	ubiquitin protein ligase E3C	2175	1	0	1	0	2	mmu-miR-214-3p	0.025	0.1791	1.0959	-0.31	-0.43	< 0.1
Stard4	ENSMUST00000119991.2	StAR-related lipid transfer (START) domain containing	2326	3	0	1	2	0	mmu-miR-214-3p	0.0256	0.179	0.9642	-0.31	-0.33	< 0.1
Dctd	ENSMUST00000170263.3	dCMP deaminase	22	3	0	3	0	0	mmu-miR-214-3p	0.0264	0.1788	0.8571	-0.4	-0.49	< 0.1
L1cam	ENSMUST00000114430.2	L1 cell adhesion molecule	20	2	1	1	0	1	mmu-miR-214-3p	0.025	0.1787	1.0629	-0.54	-0.55	< 0.1

Rhou	ENSMUST00000045487.3	ras homolog gene family, member U	659	2	0	2	0	2	mmu-miR-214-3p	0.0254	0.1783	0.9888	-0.31	-0.31	< 0.1
Cfhr1	ENSMUST00000023965.6	complement factor H-related 1	262	1	1	0	0	0	mmu-miR-214-3p	0.0284	0.1775	0.6162	-0.44	-0.45	< 0.1
Emx1	ENSMUST00000045942.8	empty spiracles homeobox 1	125	4	1	3	0	2	mmu-miR-214-3p	0.0259	0.177	0.8594	-0.49	-0.76	< 0.1
Ttl	ENSMUST00000035812.8	tubulin tyrosine ligase	519	2	1	1	0	0	mmu-miR-214-3p	0.0261	0.1768	0.8444	-0.32	-0.33	< 0.1
Plekhb2	ENSMUST00000027297.5	pleckstrin homology domain containing, family B (evectins) member 2	2312	3	0	1	2	1	mmu-miR-214-3p	0.0256	0.1762	0.9027	-0.31	-0.31	< 0.1
Ovch2	ENSMUST00000106755.2	ovochymase 2	5	1	1	0	0	0	mmu-miR-214-3p	0.0278	0.1758	0.641	-0.62	-0.62	< 0.1
1810043G02Rik	ENSMUST00000105397.4	RIKEN cDNA 1810043G02 gene	365	2	1	1	0	0	mmu-miR-214-3p	0.026	0.1755	0.829	-0.61	-0.74	< 0.1
Cdipt	ENSMUST00000032920.3	CDP-diacylglycerol--inositol 3-phosphatidyltransferase (phosphatidylinositol synthase)	1678	2	0	2	0	0	mmu-miR-214-3p	0.0251	0.1753	0.9472	-0.46	-0.46	< 0.1
Rmi2	ENSMUST00000037913.8	RMI2, RecQ mediated genome instability 2, homolog (S. cerevisiae)	105	4	0	2	2	1	mmu-miR-214-3p	0.0253	0.175	0.8951	-0.35	-0.6	< 0.1
Hs2st1	ENSMUST00000043325.7	heparan sulfate 2-O-sulfotransferase 1	972	2	1	1	0	7	mmu-miR-214-3p	0.0251	0.1747	0.9357	-0.3	-0.36	< 0.1

Cdx2	ENSMUST00000031650.3	caudal type homeobox 2	5	1	1	0	0	2	mmu-miR-214-3p	0.0265	0.1746	0.7602	-0.37	-0.37	< 0.1
Nadk2	ENSMUST00000067760.5	NAD kinase 2, mitochondrial	306	1	1	0	0	0	mmu-miR-214-3p	0.0274	0.1746	0.6518	-0.36	-0.36	< 0.1
Psca	ENSMUST00000023265.4	prostate stem cell antigen	100	1	1	0	0	1	mmu-miR-214-3p	0.026	0.1744	0.807	-0.76	-0.77	< 0.1
Nol10	ENSMUST00000046011.10	nucleolar protein 10	358	2	1	0	1	0	mmu-miR-214-3p	0.0254	0.1744	0.8595	-0.41	-0.41	< 0.1
Slc24a3	ENSMUST00000110007.2	solute carrier family 24 (sodium/potassium/calcium exchanger), member 3	677	1	1	0	0	1	mmu-miR-214-3p	0.0249	0.1742	0.9673	-0.39	-0.39	< 0.1
Urgcp	ENSMUST00000093362.6	upregulator of cell proliferation	505	4	0	2	2	4	mmu-miR-214-3p	0.0242	0.1742	1.0886	-0.3	-0.59	< 0.1
Gpr85	ENSMUST00000060442.8	G protein-coupled receptor 85	46	0	0	0	0	1	mmu-miR-214-3p	0.0267	0.1735	0.7093	-0.3	-0.3	< 0.1
Pde6g	ENSMUST00000026452.2	phosphodiesterase 6G, cGMP-specific, rod, gamma	17	1	1	0	0	0	mmu-miR-214-3p	0.0274	0.1729	0.6144	-0.63	-0.63	< 0.1
Gm10518	ENSMUST00000097454.2	predicted gene 10518	5	2	1	1	0	2	mmu-miR-214-3p	0.0253	0.1727	0.8611	-0.53	-0.53	< 0.1
Tpp1	ENSMUST00000033184.4	tripeptidyl peptidase I	1064	3	1	1	1	0	mmu-miR-214-3p	0.025	0.1719	0.8537	-0.49	-0.54	< 0.1
Tubgcp3	ENSMUST00000000776.9	tubulin, gamma complex associated protein 3	860	1	0	0	1	2	mmu-miR-214-3p	0.024	0.1719	1.0587	-0.4	-0.4	< 0.1
Ipo11	ENSMUST00000186033.1	importin 11	179	1	1	0	0	0	mmu-miR-214-3p	0.0262	0.1711	0.7184	-0.31	-0.31	< 0.1

Mcf2l	ENSMUST00000173099.2	mcf.2 transforming sequence-like	30	3	0	2	1	0	mmu-miR-214-3p	0.0239	0.1709	1.0289	-0.33	-0.33	< 0.1
Sun2	ENSMUST00000089311.5	Sad1 and UNC84 domain containing 2	589	3	0	2	1	0	mmu-miR-214-3p	0.0244	0.1704	0.9391	-0.3	-0.3	< 0.1
Arl10	ENSMUST00000026988.5	ADP-ribosylation factor-like 10	193	2	0	2	0	1	mmu-miR-214-3p	0.0242	0.1695	0.9285	-0.39	-0.4	< 0.1
Lrrc1	ENSMUST00000183873.2	leucine rich repeat containing 1	556	2	0	2	0	1	mmu-miR-214-3p	0.0244	0.1692	0.8608	-0.53	-0.54	< 0.1
Eda	ENSMUST00000113776.2	ectodysplasin-A	60	2	1	1	0	1	mmu-miR-214-3p	0.0237	0.1692	1.0079	-0.31	-0.31	< 0.1
Lysmd3	ENSMUST00000049055.6	LysM, putative peptidoglycan-binding, domain containing 3	719	1	1	0	0	1	mmu-miR-214-3p	0.0257	0.1687	0.7235	-0.3	-0.31	< 0.1
Vipas39	ENSMUST00000072744.8	VPS33B interacting protein, apical-basolateral polarity regulator, spe-39 homolog	249	6	0	5	1	0	mmu-miR-214-3p	0.0236	0.1684	0.9932	-0.39	-0.52	< 0.1
Spag11b	ENSMUST00000110767.1	sperm associated antigen 11B	5	3	0	2	1	0	mmu-miR-214-3p	0.0243	0.1683	0.868	-0.5	-0.5	< 0.1
Cdc14a	ENSMUST00000090464.5	CDC14 cell division cycle 14A	36	3	0	1	2	0	mmu-miR-214-3p	0.0241	0.1672	0.8701	-0.31	-0.32	< 0.1
Eps8l1	ENSMUST00000171445.2	EPS8-like 1	186	4	0	4	0	1	mmu-miR-214-3p	0.0242	0.1668	0.8395	-0.3	-0.46	< 0.1
Ccdc120	ENSMUST00000033490.7	coiled-coil domain containing 120	22	1	0	1	0	1	mmu-miR-214-3p	0.0236	0.1663	0.9374	-0.35	-0.35	< 0.1

Gm16039	ENSMUST00000162034.2	predicted gene 16039	1286	2	1	0	1	0	mmu-miR-214-3p	0.024	0.1657	0.8373	-0.52	-0.53	< 0.1
Tnfaip8l1	ENSMUST00000077788.6	tumor necrosis factor, alpha-induced protein 8-	102	1	0	1	0	0	mmu-miR-214-3p	0.026	0.1656	0.6058	-0.37	-0.37	< 0.1
March3	ENSMUST00000035278.6	membrane-associated ring finger (C3HC4) 3	123	3	1	1	1	3	mmu-miR-214-3p	0.0262	0.1649	0.5856	-0.35	-0.53	< 0.1
Nodal	ENSMUST00000049339.6	nodal	146	2	0	2	0	0	mmu-miR-214-3p	0.0233	0.1644	0.9248	-0.45	-0.45	< 0.1
Tbc1d10b	ENSMUST00000120705.1	TBC1 domain family, member 10b	1010	2	0	1	1	2	mmu-miR-214-3p	0.0236	0.1643	0.8876	-0.52	-0.58	< 0.1
Cdip1	ENSMUST00000004173.6	cell death inducing Trp53 target 1	2796	3	0	3	0	1	mmu-miR-214-3p	0.0237	0.1639	0.8616	-0.39	-0.57	< 0.1
S100b	ENSMUST00000036387.7	S100 protein, beta polypeptide, neural	27	2	1	1	0	4	mmu-miR-214-3p	0.0237	0.1637	0.8515	-0.38	-0.58	< 0.1
Slc16a3	ENSMUST00000168579.2	solute carrier family 16 (monocarboxylic acid transporters), member 3	2295	5	1	1	3	2	mmu-miR-214-3p	0.0232	0.1627	0.898	-0.34	-0.82	< 0.1
Sema4d	ENSMUST00000110040.3	sema domain, immunoglobulin domain (Ig), transmembrane domain (TM) and short cytoplasmic domain, (semaphorin) 4D	53	2	0	2	0	0	mmu-miR-214-3p	0.0233	0.1622	0.8583	-0.3	-0.3	< 0.1
Hexdc	ENSMUST00000038831.9	hexosaminidase (glycosyl hydrolase family 20, catalytic domain) containing	357	1	1	0	0	0	mmu-miR-214-3p	0.0252	0.1621	0.623	-0.46	-0.46	< 0.1

3110043O21Rik	ENSMUST00000084724.4	RIKEN cDNA 3110043O21 gene	809	3	0	2	1	0	mmu-miR-214-3p	0.0228	0.1608	0.9066	-0.35	-0.36	< 0.1
Gal3st2	ENSMUST00000094663.3	galactose-3-O-sulfotransferase 2	5	1	1	0	0	0	mmu-miR-214-3p	0.0249	0.1604	0.6262	-0.32	-0.32	< 0.1
Plekha6	ENSMUST00000105082.3	pleckstrin homology domain containing, family A	725	2	0	1	1	2	mmu-miR-214-3p	0.0222	0.1598	0.9911	-0.48	-0.53	< 0.1
Cd81	ENSMUST00000037941.9	CD81 antigen	4861	1	1	0	0	0	mmu-miR-214-3p	0.0245	0.1594	0.6537	-0.34	-0.36	< 0.1
Arhgap17	ENSMUST00000098060.3	Rho GTPase activating protein 17	997	2	0	1	1	1	mmu-miR-214-3p	0.0228	0.1594	0.8507	-0.31	-0.31	< 0.1
Calm4	ENSMUST00000042219.4	calmodulin 4	5	1	1	0	0	0	mmu-miR-214-3p	0.0247	0.1591	0.6146	-0.46	-0.46	< 0.1
Ltbp3	ENSMUST00000081496.5	latent transforming growth factor beta binding protein 3	4793	1	1	0	0	0	mmu-miR-214-3p	0.0232	0.1591	0.8131	-0.34	-0.35	< 0.1
Shisa3	ENSMUST00000087241.5	shisa homolog 3 (Xenopus laevis)	77	2	0	2	0	1	mmu-miR-214-3p	0.0224	0.1585	0.91	-0.36	-0.39	< 0.1
Chmp7	ENSMUST00000036381.8	charged multivesicular body protein 7	101	2	0	1	1	4	mmu-miR-214-3p	0.0221	0.1581	0.9614	-0.31	-0.48	< 0.1
Cdk11b	ENSMUST00000067081.4	cyclin-dependent kinase 11B	1196	1	1	0	0	0	mmu-miR-214-3p	0.0228	0.158	0.8169	-0.54	-0.54	< 0.1
Hsd3b7	ENSMUST00000106271.1	hydroxy-delta-5-steroid dehydrogenase, 3 beta- and steroid delta-isomerase 7	6003	2	1	1	0	2	mmu-miR-214-3p	0.023	0.1578	0.7974	-0.6	-0.6	< 0.1
Mad2l1bp	ENSMUST00000171172.1	MAD2L1 binding protein	294	1	1	0	0	0	mmu-miR-214-3p	0.0239	0.1577	0.682	-0.46	-0.46	< 0.1

Cluh	ENSMUST00000092915.6	clustered mitochondria (cluA/CLU1) homolog	6514	1	0	0	1	2	mmu-miR-214-3p	0.0218	0.1575	0.9966	-0.33	-0.33	< 0.1
Slc2a1	ENSMUST00000030398.4	solute carrier family 2 (facilitated glucose transporter),	4281	2	0	1	1	1	mmu-miR-214-3p	0.0223	0.1574	0.8977	-0.38	-0.39	< 0.1
Frmd8	ENSMUST00000025728.7	FERM domain containing 8	1448	3	0	2	1	5	mmu-miR-214-3p	0.0219	0.1573	0.972	-0.56	-0.77	< 0.1
Mapkapk3	ENSMUST00000035194.2	mitogen-activated protein kinase-activated protein kinase 3	538	4	0	1	3	1	mmu-miR-214-3p	0.022	0.157	0.9486	-0.35	-0.35	< 0.1
Tgif2	ENSMUST00000081335.7	TGFB-induced factor homeobox 2	10	3	0	0	3	3	mmu-miR-214-3p	0.0222	0.1567	0.9	-0.59	-0.67	< 0.1
Scgn	ENSMUST00000021770.7	secretagogin, EF-hand calcium binding protein	7	1	0	1	0	0	mmu-miR-214-3p	0.0239	0.1562	0.6465	-0.43	-0.43	< 0.1
Ctu1	ENSMUST00000038332.8	cytosolic thiouridylase subunit 1 homolog (S. pombe)	119	2	1	0	1	0	mmu-miR-214-3p	0.0231	0.1558	0.7407	-0.36	-0.36	< 0.1
Galnt7	ENSMUST00000034021.6	UDP-N-acetyl-alpha-D-galactosamine: polypeptide N-acetylgalactosaminyltransferase 7	218	2	0	2	0	1	mmu-miR-214-3p	0.0224	0.1558	0.8154	-0.31	-0.32	< 0.1
Tchp	ENSMUST00000094441.5	trichoplein, keratin filament binding	332	1	1	0	0	1	mmu-miR-214-3p	0.0234	0.1551	0.6811	-0.37	-0.51	< 0.1
1700066B19Rik	ENSMUST00000097617.2	RIKEN cDNA 1700066B19 gene	11	2	0	1	1	1	mmu-miR-214-3p	0.0217	0.1549	0.9282	-0.36	-0.37	< 0.1

Sclt1	ENSMUST00000026866.9	sodium channel and clathrin linker 1	363	1	1	0	0	0	mmu-miR-214-3p	0.024	0.1548	0.5951	-0.42	-0.42	< 0.1
Pak6	ENSMUST00000099557.4	p21 protein (Cdc42/Rac)-activated kinase 6	111	1	0	1	0	1	mmu-miR-214-3p	0.0218	0.1548	0.8966	-0.36	-0.38	< 0.1
Zfp691	ENSMUST00000052715.5	zinc finger protein 691	21	2	0	2	0	0	mmu-miR-214-3p	0.0223	0.1545	0.7917	-0.54	-0.54	< 0.1
Sema6d	ENSMUST00000103239.4	sema domain, transmembrane domain (TM), and cytoplasmic domain,	274	2	0	2	0	0	mmu-miR-214-3p	0.0218	0.1537	0.8769	-0.35	-0.35	< 0.1
Prps2	ENSMUST00000026839.4	phosphoribosyl pyrophosphate synthetase 2	310	1	0	1	0	0	mmu-miR-214-3p	0.0238	0.1537	0.6036	-0.32	-0.35	< 0.1
Tmprss6	ENSMUST00000017086.3	transmembrane serine protease 6	6342	1	1	0	0	0	mmu-miR-214-3p	0.0225	0.1537	0.7722	-0.3	-0.3	< 0.1
Skint6	ENSMUST00000171224.1	selection and upkeep of intraepithelial T cells	2	1	1	0	0	0	mmu-miR-214-3p	0.0234	0.1533	0.6387	-0.41	-0.41	< 0.1
Farp2	ENSMUST00000120301.2	FERM, RhoGEF and pleckstrin domain protein 2	403	1	1	0	0	1	mmu-miR-214-3p	0.0216	0.1529	0.8673	-0.45	-0.45	< 0.1
Calm5	ENSMUST00000183198.2	calmodulin 5	5	1	1	0	0	0	mmu-miR-214-3p	0.0238	0.1524	0.5706	-0.5	-0.5	< 0.1
Tspan11	ENSMUST00000032501.4	tetraspanin 11	2495	3	2	0	1	0	mmu-miR-214-3p	0.0219	0.1504	0.7622	-0.51	-0.71	< 0.1
Oraov1	ENSMUST00000033388.6	oral cancer overexpressed 1	313	2	1	1	0	3	mmu-miR-214-3p	0.0222	0.1502	0.7101	-0.3	-0.51	< 0.1
Map1lc3b	ENSMUST00000034270.11	microtubule-associated protein 1 light chain 3 beta	15047	3	0	2	1	3	mmu-miR-214-3p	0.0211	0.15	0.8773	-0.38	-0.42	< 0.1

Bpnt1	ENSMUST00000027916.7	bisphosphate 3'-nucleotidase 1	6548	1	1	0	0	3	mmu-miR-214-3p	0.0232	0.1497	0.5762	-0.43	-0.49	< 0.1
Abcc4	ENSMUST00000036554.7	ATP-binding cassette, sub-family C (CFTR/MRP), member 4	329	2	0	2	0	1	mmu-miR-214-3p	0.0214	0.1494	0.8051	-0.3	-0.31	< 0.1
Klk4	ENSMUST00000007161.6	kallikrein related-peptidase 4 (prostase, enamel matrix, prostate)	5	1	1	0	0	0	mmu-miR-214-3p	0.0231	0.1493	0.5788	-0.4	-0.4	< 0.1
Mak	ENSMUST00000165087.2	male germ cell-associated kinase	109	1	1	0	0	1	mmu-miR-214-3p	0.0226	0.1489	0.6333	-0.31	-0.33	< 0.1
Lman2l	ENSMUST00000125304.2	lectin, mannose-binding 2-like	787	3	0	3	0	2	mmu-miR-214-3p	0.0211	0.1487	0.8281	-0.43	-0.48	< 0.1
Map7	ENSMUST00000116259.3	microtubule-associated protein 7	1064	3	0	3	0	1	mmu-miR-214-3p	0.0209	0.1486	0.863	-0.31	-0.32	< 0.1
Mtm1	ENSMUST00000171933.2	X-linked myotubular myopathy gene 1	394	2	0	1	1	1	mmu-miR-214-3p	0.0212	0.1485	0.8003	-0.35	-0.36	< 0.1
Glo1	ENSMUST00000024823.7	glyoxalase 1	19773	2	1	1	0	1	mmu-miR-214-3p	0.0215	0.1481	0.7641	-0.37	-0.37	< 0.1
Ramp3	ENSMUST00000045374.7	receptor (calcitonin) activity modifying protein 3	205	2	0	1	1	0	mmu-miR-214-3p	0.0213	0.148	0.7889	-0.35	-0.35	< 0.1
Dio3	ENSMUST00000173014.1	deiodinase, iodothyronine type III	21	4	0	3	1	0	mmu-miR-214-3p	0.0207	0.1475	0.8752	-0.43	-0.43	< 0.1
Asb16	ENSMUST00000036467.4	ankyrin repeat and SOCS box-containing 16	756	0	0	0	0	0	mmu-miR-214-3p	0.0219	0.1475	0.6956	-0.38	-0.38	< 0.1
Apon	ENSMUST00000060782.3	apolipoprotein N	3884	1	1	0	0	0	mmu-miR-214-3p	0.0228	0.1469	0.5639	-0.45	-0.45	< 0.1

Pim1	ENSMUST00000024811.7	proviral integration site 1	5	3	0	1	2	1	mmu-miR-214-3p	0.0209	0.1467	0.8088	-0.35	-0.35	< 0.1
Faah	ENSMUST00000049095.5	fatty acid amide hydrolase	396	2	1	0	1	0	mmu-miR-214-3p	0.0207	0.1453	0.8097	-0.3	-0.49	< 0.1
4833423E24Rik	ENSMUST00000099923.1	RIKEN cDNA 4833423E24 gene	6	1	1	0	0	0	mmu-miR-214-3p	0.0224	0.1446	0.5554	-0.48	-0.48	< 0.1
A4gnt	ENSMUST00000042553.7	alpha-1,4-N-acetylglucosaminyltransferase	23	1	0	1	0	1	mmu-miR-214-3p	0.0214	0.1436	0.659	-0.3	-0.3	< 0.1
Mblac1	ENSMUST00000057773.4	metallo-beta-lactamase domain containing 1	77	1	1	0	0	1	mmu-miR-214-3p	0.0218	0.1429	0.5823	-0.5	-0.51	< 0.1
Cisd1	ENSMUST00000045887.8	CDGSH iron sulfur domain 1	10205	2	1	1	0	0	mmu-miR-214-3p	0.0219	0.1423	0.5583	-0.63	-0.79	< 0.1
Ovca2	ENSMUST00000071562.2	candidate tumor suppressor in ovarian cancer 2	1090	2	1	1	0	0	mmu-miR-214-3p	0.0214	0.1423	0.6198	-0.31	-0.55	< 0.1
Ifitm7	ENSMUST00000117803.1	interferon induced transmembrane protein 7	5	1	1	0	0	0	mmu-miR-214-3p	0.0215	0.1411	0.5822	-0.53	-0.53	< 0.1
Icam1	ENSMUST00000086399.4	intercellular adhesion molecule 1	1864	2	1	1	0	2	mmu-miR-214-3p	0.0198	0.1408	0.8204	-0.33	-0.33	< 0.1
Glb1	ENSMUST00000063042.9	galactosidase, beta 1	193	2	0	1	1	0	mmu-miR-214-3p	0.0201	0.1405	0.7555	-0.41	-0.41	< 0.1
A730049H05Rik	ENSMUST00000057977.3	RIKEN cDNA A730049H05 gene	10	1	1	0	0	1	mmu-miR-214-3p	0.0198	0.1404	0.8095	-0.4	-0.4	< 0.1
Pid1	ENSMUST00000168574.3	phosphotyrosine interaction domain containing 1	1213	2	0	2	0	0	mmu-miR-214-3p	0.0202	0.1401	0.7123	-0.34	-0.34	< 0.1

Olfr283	ENSMUST00000057386.5	olfactory receptor 283	5	1	1	0	0	0	mmu-miR-214-3p	0.0207	0.1398	0.657	-0.58	-0.58	< 0.1
Aktip	ENSMUST00000120349.2	thymoma viral proto-oncogene 1 interacting protein	2485	2	1	1	0	0	mmu-miR-214-3p	0.0202	0.1392	0.6953	-0.46	-0.48	< 0.1
Katnb1	ENSMUST00000034239.7	katanin p80 (WD40-containing) subunit B 1	636	2	1	1	0	1	mmu-miR-214-3p	0.0202	0.1386	0.7031	-0.41	-0.41	< 0.1
Ube2g2	ENSMUST00000174510.2	ubiquitin-conjugating enzyme E2G 2	1548	2	1	1	0	2	mmu-miR-214-3p	0.0196	0.1385	0.7739	-0.33	-0.33	< 0.1
Prr15l	ENSMUST00000107636.3	proline rich 15-like	14	5	0	2	3	2	mmu-miR-214-3p	0.0196	0.138	0.7775	-0.37	-0.61	< 0.1
Hoxa4	ENSMUST00000101395.2	homeobox A4	40	1	1	0	0	0	mmu-miR-214-3p	0.0206	0.1379	0.6166	-0.42	-0.42	< 0.1
Gm7347	ENSMUST00000095004.3	predicted gene 7347	5	2	0	1	1	2	mmu-miR-214-3p	0.019	0.1375	0.8826	-0.54	-0.54	< 0.1
Gm5464	ENSMUST00000100453.1	predicted gene 5464	6	1	1	0	0	1	mmu-miR-214-3p	0.0207	0.137	0.5812	-0.36	-0.36	< 0.1
Myoz1	ENSMUST00000090469.6	myozenin 1	22070	1	0	1	0	1	mmu-miR-214-3p	0.0206	0.1369	0.5976	-0.35	-0.37	< 0.1
3110018I06Rik	ENSMUST00000082269.2	RIKEN cDNA 3110018I06 gene	5	1	1	0	0	0	mmu-miR-214-3p	0.0209	0.1362	0.5382	-0.48	-0.48	< 0.1
Dpy19l2	ENSMUST00000133010.2	dpy-19-like 2 (C. elegans)	5	1	0	1	0	0	mmu-miR-214-3p	0.0202	0.1362	0.6396	-0.31	-0.31	< 0.1
Nes	ENSMUST00000090973.6	nestin	852	2	1	1	0	0	mmu-miR-214-3p	0.0191	0.136	0.8069	-0.49	-0.5	< 0.1

4731417B20Rik	ENSMUST00000053319.2	RIKEN cDNA 4731417B20 gene	5	2	1	1	0	0	mmu-miR-214-3p	0.0197	0.1357	0.6796	-0.46	-0.46	< 0.1
Lce1a1	ENSMUST00000074142.5	late cornified envelope 1A1	5	1	0	1	0	0	mmu-miR-214-3p	0.0195	0.1352	0.6979	-0.37	-0.37	< 0.1
Gm11733	ENSMUST00000092394.3	predicted gene 11733	5	1	1	0	0	0	mmu-miR-214-3p	0.0207	0.1351	0.5357	-0.33	-0.33	< 0.1
Pdlim3	ENSMUST00000034053.5	PDZ and LIM domain 3	17949	1	1	0	0	0	mmu-miR-214-3p	0.0205	0.1343	0.5447	-0.37	-0.37	< 0.1
Cmc2	ENSMUST00000148235.2	COX assembly mitochondrial protein 2	3529	1	1	0	0	2	mmu-miR-214-3p	0.0197	0.1342	0.6595	-0.53	-0.55	< 0.1
Tor1a	ENSMUST00000028200.8	torsin family 1, member A (torsin A)	518	3	2	1	0	1	mmu-miR-214-3p	0.0203	0.1341	0.5678	-0.42	-0.62	< 0.1
Vat1	ENSMUST00000040430.7	vesicle amine transport protein 1 homolog (T californica)	1543	2	0	1	1	2	mmu-miR-214-3p	0.0187	0.134	0.8125	-0.42	-0.42	< 0.1
B230104I21Rik	ENSMUST00000181143.1	RIKEN cDNA B230104I21 gene	5	1	0	1	0	0	mmu-miR-214-3p	0.0204	0.1339	0.5441	-0.49	-0.49	< 0.1
Polr3g	ENSMUST00000048993.6	polymerase (RNA) III (DNA directed) polypeptide G	63	2	1	0	1	0	mmu-miR-214-3p	0.0193	0.1339	0.6866	-0.3	-0.36	< 0.1
Hnf1a	ENSMUST00000031535.6	HNF1 homeobox A	38	2	0	2	0	2	mmu-miR-214-3p	0.0185	0.1337	0.8615	-0.36	-0.36	< 0.1
Arhgef5	ENSMUST00000031750.8	Rho guanine nucleotide exchange factor	178	2	1	1	0	0	mmu-miR-214-3p	0.0193	0.1335	0.6842	-0.33	-0.33	< 0.1
D11Wsu47e	ENSMUST00000042227.9	DNA segment, Chr 11, Wayne State University 47, expressed	154	1	0	0	1	0	mmu-miR-214-3p	0.0195	0.1333	0.656	-0.33	-0.33	< 0.1

Gga1	ENSMUST00000041587.7	golgi associated, gamma adaptin ear containing, ARF binding protein 1	648	1	1	0	0	2	mmu-miR-214-3p	0.0186	0.133	0.7947	-0.47	-0.47	< 0.1
Sdf2l1	ENSMUST00000023453.7	stromal cell-derived factor 2-like 1	103	1	1	0	0	0	mmu-miR-214-3p	0.0216	0.1329	0.4417	-0.39	-0.63	< 0.1
5031410I06Rik	ENSMUST00000063524.2	RIKEN cDNA 5031410I06 gene	5	2	0	1	1	1	mmu-miR-214-3p	0.0184	0.1328	0.843	-0.49	-0.49	< 0.1
Hspb1	ENSMUST00000111155.1	heat shock protein 1	171	2	0	2	0	0	mmu-miR-214-3p	0.0188	0.1304	0.6733	-0.56	-0.61	< 0.1
Grtp1	ENSMUST00000165605.2	GH regulated TBC protein 1	515	1	0	1	0	0	mmu-miR-214-3p	0.0196	0.13	0.5635	-0.4	-0.4	< 0.1
Tmem8c	ENSMUST00000009358.3	transmembrane protein 8C	25	3	0	2	1	0	mmu-miR-214-3p	0.0183	0.1297	0.7509	-0.4	-0.4	< 0.1
Copz1	ENSMUST00000100162.3	coatomer protein complex, subunit zeta 1	5113	1	0	1	0	3	mmu-miR-214-3p	0.0183	0.1297	0.7516	-0.31	-0.31	< 0.1
Krt39	ENSMUST00000107445.2	keratin 39	5	1	1	0	0	0	mmu-miR-214-3p	0.0194	0.1278	0.5323	-0.61	-0.61	< 0.1
4930524B15Rik	ENSMUST00000102829.3	RIKEN cDNA 4930524B15 gene	7	3	0	1	2	0	mmu-miR-214-3p	0.0182	0.1262	0.663	-0.34	-0.36	< 0.1
Il7r	ENSMUST00000003981.4	interleukin 7 receptor	34	2	0	2	0	0	mmu-miR-214-3p	0.0178	0.1255	0.6942	-0.38	-0.38	< 0.1
Lce1k	ENSMUST00000179917.1	late cornified envelope 1K	5	1	0	1	0	0	mmu-miR-214-3p	0.0179	0.1251	0.6837	-0.34	-0.34	< 0.1
Mesdc2	ENSMUST00000094215.4	mesoderm development candidate 2	2852	3	1	2	0	5	mmu-miR-214-3p	0.0174	0.1243	0.7407	-0.39	-0.66	< 0.1

Ecscr	ENSMUST00000097618.4	endothelial cell surface expressed chemotaxis and apoptosis regulator	110	1	0	1	0	0	mmu-miR-214-3p	0.0175	0.1236	0.702	-0.32	-0.33	< 0.1
H2-Bl	ENSMUST00000173080.4	histocompatibility 2, blastocyst	101	1	1	0	0	5	mmu-miR-214-3p	0.0178	0.1235	0.6501	-0.4	-0.41	< 0.1
Rnf5	ENSMUST00000015622.7	ring finger protein 5	220	2	0	1	1	1	mmu-miR-214-3p	0.0174	0.1231	0.7004	-0.36	-0.36	< 0.1
Trip10	ENSMUST00000019631.9	thyroid hormone receptor interactor 10	587	1	0	1	0	1	mmu-miR-214-3p	0.0179	0.1219	0.583	-0.37	-0.37	< 0.1
Asprv1	ENSMUST00000043400.6	aspartic peptidase, retroviral-like 1	5	1	1	0	0	0	mmu-miR-214-3p	0.0178	0.1214	0.5886	-0.31	-0.31	< 0.1
Cxx1b	ENSMUST00000088778.4	CAAX box 1B	9	3	0	3	0	2	mmu-miR-214-3p	0.0172	0.1209	0.6759	-0.5	-0.5	< 0.1
Gm21698	ENSMUST00000191203.1	predicted gene, 21698	5	2	0	1	1	1	mmu-miR-214-3p	0.0167	0.1208	0.7674	-0.48	-0.48	< 0.1
Cxx1a	ENSMUST00000088779.4	CAAX box 1A	5	3	0	3	0	2	mmu-miR-214-3p	0.0172	0.1206	0.6742	-0.51	-0.51	< 0.1
A630091E08Rik	ENSMUST00000069035.4	RIKEN cDNA A630091E08 gene	5	1	0	1	0	1	mmu-miR-214-3p	0.0174	0.1206	0.6249	-0.42	-0.42	< 0.1
Lrrc6	ENSMUST00000023006.6	leucine rich repeat containing 6 (testis)	22	1	1	0	0	0	mmu-miR-214-3p	0.0191	0.1205	0.4327	-0.33	-0.53	< 0.1
Gm10610	ENSMUST00000177929.1	predicted gene 10610	5	2	0	1	1	2	mmu-miR-214-3p	0.0168	0.1202	0.7309	-0.41	-0.41	< 0.1
C130074G19Rik	ENSMUST00000048308.5	RIKEN cDNA C130074G19 gene	1411	2	1	1	0	1	mmu-miR-214-3p	0.0172	0.1201	0.638	-0.33	-0.38	< 0.1

Zfp526	ENSMUST00000055604.4	zinc finger protein 526	45	2	0	1	1	0	mmu-miR-214-3p	0.0171	0.1199	0.6626	-0.32	-0.32	< 0.1
Reep1	ENSMUST00000121469.1	receptor accessory protein 1	124	2	0	1	1	2	mmu-miR-214-3p	0.0169	0.1195	0.693	-0.3	-0.33	< 0.1
Cops7a	ENSMUST00000112439.3	COP9 (constitutive photomorphogenic) homolog, subunit 7a (Arabidopsis	366	1	1	0	0	0	mmu-miR-214-3p	0.0178	0.1193	0.5345	-0.45	-0.45	< 0.1
Gm7120	ENSMUST00000177361.1	predicted gene 7120	7	1	1	0	0	1	mmu-miR-214-3p	0.017	0.1187	0.6326	-0.45	-0.45	< 0.1
Ngp	ENSMUST00000035061.6	neutrophilic granule protein	31	2	0	2	0	1	mmu-miR-214-3p	0.0166	0.1183	0.7021	-0.53	-0.53	< 0.1
Fxyd6	ENSMUST00000085939.6	FXYD domain-containing ion transport regulator	1033	4	0	2	2	3	mmu-miR-214-3p	0.0168	0.1182	0.6597	-0.44	-0.64	< 0.1
Tmem109	ENSMUST00000038128.9	transmembrane protein 109	653	4	0	3	1	0	mmu-miR-214-3p	0.0168	0.1182	0.6646	-0.31	-0.54	< 0.1
Atf7ip2	ENSMUST00000044005.8	activating transcription factor 7 interacting protein	179	1	0	1	0	1	mmu-miR-214-3p	0.0169	0.1176	0.6301	-0.3	-0.3	< 0.1
Micu1	ENSMUST00000165563.2	mitochondrial calcium uptake 1	773	3	0	2	1	1	mmu-miR-214-3p	0.0168	0.1173	0.6305	-0.32	-0.34	< 0.1
Tax1bp3	ENSMUST00000040687.6	Tax1 (human T cell leukemia virus type I) binding protein 3	45	1	0	1	0	2	mmu-miR-214-3p	0.0163	0.117	0.7264	-0.37	-0.45	< 0.1
2900055J20Rik	ENSMUST00000096572.1	RIKEN cDNA 2900055J20 gene	5	1	0	1	0	0	mmu-miR-214-3p	0.0175	0.1165	0.4988	-0.46	-0.46	< 0.1
Pttg1ip	ENSMUST00000009435.6	pituitary tumor-transforming 1 interacting protein	9041	3	0	2	1	2	mmu-miR-214-3p	0.0162	0.1163	0.7279	-0.31	-0.58	< 0.1

Marveld2	ENSMUST00000022137.8	MARVEL (membrane-associating) domain containing 2	77	2	0	2	0	4	mmu-miR-214-3p	0.0162	0.1161	0.7119	-0.39	-0.69	< 0.1
Hpd	ENSMUST00000031398.8	4-hydroxyphenylpyruvic acid	6563	1	1	0	0	0	mmu-miR-214-3p	0.0174	0.1159	0.4943	-0.54	-0.55	< 0.1
Ncmap	ENSMUST00000105858.2	noncompact myelin associated protein	24	1	0	1	0	2	mmu-miR-214-3p	0.0162	0.1159	0.7006	-0.44	-0.44	< 0.1
Trim16	ENSMUST00000055006.6	tripartite motif-containing 16	426	1	1	0	0	0	mmu-miR-214-3p	0.0168	0.1157	0.581	-0.36	-0.36	< 0.1
Pdss2	ENSMUST00000095725.5	prenyl (solanesyl) diphosphate synthase, subunit 2	1192	1	0	1	0	0	mmu-miR-214-3p	0.0166	0.1154	0.6081	-0.3	-0.3	< 0.1
Gm11541	ENSMUST00000069852.1	predicted gene 11541	5	1	1	0	0	0	mmu-miR-214-3p	0.0169	0.1153	0.5517	-0.4	-0.4	< 0.1
1810013L24Rik	ENSMUST00000023150.5	RIKEN cDNA 1810013L24 gene	1935	4	0	1	3	0	mmu-miR-214-3p	0.0171	0.1152	0.5216	-0.31	-0.4	< 0.1
Ino80b	ENSMUST00000113935.2	INO80 complex subunit B	285	1	0	1	0	0	mmu-miR-214-3p	0.0169	0.1145	0.5339	-0.41	-0.41	< 0.1
Clec12a	ENSMUST00000065289.4	C-type lectin domain family 12, member a	87	1	0	1	0	1	mmu-miR-214-3p	0.0164	0.1141	0.5999	-0.3	-0.3	< 0.1
Esp8	ENSMUST00000171813.2	exocrine gland secreted peptide 8	5	1	0	1	0	0	mmu-miR-214-3p	0.0166	0.114	0.5633	-0.38	-0.38	< 0.1
Sprr2b	ENSMUST00000061038.2	small proline-rich protein 2B	5	1	0	1	0	1	mmu-miR-214-3p	0.0163	0.1139	0.6018	-0.41	-0.41	< 0.1
Ppih	ENSMUST00000106321.3	peptidyl prolyl isomerase H	80	2	1	1	0	3	mmu-miR-214-3p	0.016	0.1137	0.6709	-0.33	-0.58	< 0.1

BC029214	ENSMUST00000114261.3	cDNA sequence BC029214	479	1	0	1	0	0	mmu-miR-214-3p	0.0169	0.1134	0.4954	-0.36	-0.36	< 0.1
Tcn2	ENSMUST00000109993.3	transcobalamin 2	355	2	0	2	0	1	mmu-miR-214-3p	0.0157	0.1132	0.7043	-0.44	-0.44	< 0.1
Adi1	ENSMUST00000020957.7	acireductone dioxygenase 1	119	1	0	1	0	0	mmu-miR-214-3p	0.0168	0.1131	0.5143	-0.32	-0.32	< 0.1
Nptn	ENSMUST00000176250.1	neuroplastin	4527	2	0	0	2	1	mmu-miR-214-3p	0.0158	0.1131	0.6843	-0.32	-0.32	< 0.1
Cxcl16	ENSMUST00000019064.3	chemokine (C-X-C motif) ligand 16	1127	2	1	0	1	0	mmu-miR-214-3p	0.0168	0.1128	0.5047	-0.49	-0.61	< 0.1
Arl2	ENSMUST00000025893.5	ADP-ribosylation factor-like 2	4938	2	0	2	0	0	mmu-miR-214-3p	0.016	0.1125	0.6249	-0.49	-0.49	< 0.1
Grm4	ENSMUST00000118161.1	glutamate receptor, metabotropic 4	11	1	1	0	0	1	mmu-miR-214-3p	0.0158	0.1124	0.6787	-0.32	-0.32	< 0.1
Espn	ENSMUST00000030785.9	espin	77	4	0	3	1	0	mmu-miR-214-3p	0.0155	0.1123	0.7269	-0.43	-0.44	< 0.1
Lce1a2	ENSMUST00000029530.5	late cornified envelope 1A2	5	1	0	1	0	0	mmu-miR-214-3p	0.0158	0.1119	0.6388	-0.34	-0.34	< 0.1
Gm10471	ENSMUST00000094946.4	predicted gene 10471	5	2	0	1	1	1	mmu-miR-214-3p	0.0155	0.1116	0.703	-0.45	-0.45	< 0.1
Fgf22	ENSMUST00000020577.2	fibroblast growth factor 22	1	2	0	1	1	2	mmu-miR-214-3p	0.0153	0.1105	0.6953	-0.31	-0.31	< 0.1
9430015G10Rik	ENSMUST00000072554.7	RIKEN cDNA 9430015G10 gene	153	3	0	1	2	0	mmu-miR-214-3p	0.0155	0.1103	0.6455	-0.36	-0.38	< 0.1

Guca1a	ENSMUST00000059348.7	guanylate cyclase activator 1a (retina)	20	1	1	0	0	0	mmu-miR-214-3p	0.0165	0.1102	0.4779	-0.53	-0.53	< 0.1
Il24	ENSMUST00000187650.1	interleukin 24	5	1	0	1	0	0	mmu-miR-214-3p	0.0154	0.1099	0.6519	-0.34	-0.34	< 0.1
Pcbp2	ENSMUST00000077037.6	poly(rC) binding protein 2	8069	1	0	1	0	0	mmu-miR-214-3p	0.0163	0.1098	0.4995	-0.39	-0.39	< 0.1
Ebp	ENSMUST00000033509.9	phenylalkylamine Ca2+ antagonist (emopamil) binding protein	178	2	1	0	1	2	mmu-miR-214-3p	0.0162	0.1097	0.5105	-0.48	-0.61	< 0.1
Saysd1	ENSMUST00000059666.4	SAYSVFN motif domain containing 1	121	2	0	1	1	0	mmu-miR-214-3p	0.0157	0.1092	0.578	-0.35	-0.46	< 0.1
1110012L19Rik	ENSMUST00000053981.5	RIKEN cDNA 1110012L19 gene	142	1	0	1	0	0	mmu-miR-214-3p	0.0159	0.1091	0.5409	-0.41	-0.41	< 0.1
Pdcl2	ENSMUST00000122213.2	phosducin-like 2	17	1	1	0	0	0	mmu-miR-214-3p	0.0176	0.1084	0.3588	-0.33	-0.66	< 0.1
Eif1ad	ENSMUST00000025759.7	eukaryotic translation initiation factor 1A domain	2231	3	1	2	0	1	mmu-miR-214-3p	0.0152	0.1083	0.6339	-0.46	-0.91	< 0.1
Pter	ENSMUST00000028063.5	phosphotriesterase related	1482	1	0	1	0	0	mmu-miR-214-3p	0.0161	0.1079	0.4752	-0.33	-0.33	< 0.1
1110051M20Rik	ENSMUST00000102594.5	RIKEN cDNA 1110051M20 gene	712	3	0	2	1	0	mmu-miR-214-3p	0.0148	0.1076	0.7037	-0.56	-0.56	< 0.1
Vmac	ENSMUST00000164907.1	vimentin-type intermediate filament associated coiled-coil protein	43	1	1	0	0	0	mmu-miR-214-3p	0.0154	0.1071	0.575	-0.32	-0.33	< 0.1
Hmgn3	ENSMUST00000185315.1	high mobility group nucleosomal binding domain 3	119	0	0	0	0	0	mmu-miR-214-3p	0.0159	0.1066	0.4685	-0.53	-0.53	< 0.1

Gpx4	ENSMUST00000105372.2	glutathione peroxidase 4	13	2	1	1	0	1	mmu-miR-214-3p	0.0151	0.1061	0.5782	-0.37	-0.64	< 0.1
Slc27a1	ENSMUST00000034267.4	solute carrier family 27 (fatty acid transporter), member 1	552	1	1	0	0	2	mmu-miR-214-3p	0.0151	0.1061	0.597	-0.35	-0.35	< 0.1
Ly6c2	ENSMUST00000100542.4	lymphocyte antigen 6 complex, locus C2	55	2	1	1	0	0	mmu-miR-214-3p	0.0152	0.106	0.5734	-0.71	-0.71	< 0.1
Cxxc5	ENSMUST00000060722.6	CXXC finger 5	1271	2	1	1	0	0	mmu-miR-214-3p	0.0148	0.1057	0.6287	-0.43	-0.43	< 0.1
A230083G16Rik	ENSMUST00000069553.2	RIKEN cDNA A230083G16 gene	5	1	1	0	0	0	mmu-miR-214-3p	0.0155	0.1057	0.5094	-0.37	-0.37	< 0.1
Esp1	ENSMUST00000161110.1	exocrine gland secreted peptide 1	5	1	0	1	0	0	mmu-miR-214-3p	0.0151	0.1057	0.5718	-0.34	-0.34	< 0.1
Rps6kl1	ENSMUST00000019379.7	ribosomal protein S6 kinase-like 1	84	1	1	0	0	1	mmu-miR-214-3p	0.0151	0.1046	0.5349	-0.34	-0.34	< 0.1
Slc6a12	ENSMUST00000032200.10	solute carrier family 6 (neurotransmitter transporter, betaine/GABA), member 12	927	2	0	2	0	1	mmu-miR-214-3p	0.0148	0.1045	0.5897	-0.47	-0.49	< 0.1
Rhob	ENSMUST00000067384.4	ras homolog gene family, member B	4893	1	1	0	0	1	mmu-miR-214-3p	0.0146	0.1043	0.6212	-0.38	-0.38	< 0.1
Dlk2	ENSMUST00000170271.1	delta-like 2 homolog (Drosophila)	7	2	0	1	1	1	mmu-miR-214-3p	0.0143	0.1036	0.6712	-0.32	-0.32	< 0.1
Rasd2	ENSMUST00000139848.2	RASD family, member 2	319	2	0	2	0	0	mmu-miR-214-3p	0.0145	0.1034	0.6224	-0.31	-0.32	< 0.1

Uck2	ENSMUST00000053686.3	uridine-cytidine kinase 2	208	1	0	1	0	1	mmu-miR-214-3p	0.0147	0.1018	0.5175	-0.31	-0.31	< 0.1
Pabpc1l	ENSMUST00000067715.4	poly(A) binding protein, cytoplasmic 1-like	9	1	1	0	0	2	mmu-miR-214-3p	0.0143	0.1016	0.5869	-0.62	-0.62	< 0.1
Gimap1	ENSMUST00000054368.4	GTPase, IMAP family member 1	8	3	0	3	0	1	mmu-miR-214-3p	0.0141	0.1014	0.6407	-0.31	-0.43	< 0.1
Sept4	ENSMUST00000107960.2	septin 4	753	1	1	0	0	0	mmu-miR-214-3p	0.0149	0.1013	0.4831	-0.52	-0.53	< 0.1
Lair1	ENSMUST00000068865.6	leukocyte-associated Ig-like receptor 1	25	1	1	0	0	1	mmu-miR-214-3p	0.0146	0.1013	0.5325	-0.38	-0.38	< 0.1
Slc9a3r2	ENSMUST00000019684.6	solute carrier family 9 (sodium/hydrogen exchanger),	436	0	0	0	0	0	mmu-miR-214-3p	0.0147	0.101	0.496	-0.33	-0.33	< 0.1
Gm5900	ENSMUST00000179862.1	predicted pseudogene 5900	6	1	1	0	0	1	mmu-miR-214-3p	0.0147	0.1007	0.4906	-0.39	-0.39	< 0.1
Speer4a	ENSMUST00000079447.2	spermatogenesis associated glutamate (E)-rich	5	2	0	1	1	1	mmu-miR-214-3p	0.0139	0.1004	0.6478	-0.42	-0.42	< 0.1
Lamtor3	ENSMUST00000168345.1	late endosomal/lysosomal adaptor, MAPK and MTOR activator	17	1	1	0	0	1	mmu-miR-214-3p	0.0147	0.1002	0.4822	-0.36	-0.37	< 0.1
Sigmar1	ENSMUST00000059354.9	sigma non-opioid intracellular receptor 1	216	2	0	2	0	0	mmu-miR-214-3p	0.0141	0.0999	0.5631	-0.34	-0.34	< 0.1
Slc41a3	ENSMUST00000044019.10	solute carrier family 41, member 3	1405	1	1	0	0	0	mmu-miR-214-3p	0.014	0.0999	0.592	-0.3	-0.3	< 0.1
Gm16223	ENSMUST00000087332.4	predicted gene 16223	56	1	0	1	0	1	mmu-miR-214-3p	0.014	0.099	0.5608	-0.3	-0.3	< 0.1

Gimap 7	ENSMUST00000052503.7	GTPase, IMAP family member 7	26	1	1	0	0	0	mmu-miR-214-3p	0.0146	0.0989	0.4589	-0.49	-0.49	< 0.1
Nsmf	ENSMUST00000100334.5	NMDA receptor synaptonuclear signaling and neuronal migration factor	3429	2	0	1	1	5	mmu-miR-214-3p	0.0137	0.0989	0.627	-0.32	-0.35	< 0.1
G6pc2	ENSMUST00000112317.2	glucose-6-phosphatase, catalytic, 2	7	2	0	1	1	0	mmu-miR-214-3p	0.0138	0.0983	0.586	-0.47	-0.47	< 0.1
Apoc3	ENSMUST00000121916.2	apolipoprotein C-III	30960	1	0	1	0	2	mmu-miR-214-3p	0.0145	0.0981	0.4522	-0.34	-0.45	< 0.1
Cetn2	ENSMUST00000114551.4	centrin 2	1010	0	0	0	0	0	mmu-miR-214-3p	0.0144	0.0977	0.4517	-0.57	-0.58	< 0.1
Rhog	ENSMUST00000098230.5	ras homolog gene family, member G	115	1	1	0	0	2	mmu-miR-214-3p	0.0141	0.0974	0.4954	-0.35	-0.41	< 0.1
Nmb	ENSMUST00000026817.5	neuromedin B	60	1	0	1	0	0	mmu-miR-214-3p	0.0143	0.097	0.452	-0.4	-0.4	< 0.1
Gm10654	ENSMUST00000098653.2	predicted gene 10654	5	2	0	2	0	2	mmu-miR-214-3p	0.0134	0.0969	0.6188	-0.34	-0.34	< 0.1
Mrpl17	ENSMUST00000124482.1	mitochondrial ribosomal protein L17	2376	3	1	2	0	1	mmu-miR-214-3p	0.014	0.0959	0.4638	-0.49	-0.75	< 0.1
Atg10	ENSMUST00000022119.4	autophagy related 10	264	2	0	2	0	0	mmu-miR-214-3p	0.0138	0.0955	0.4877	-0.3	-0.6	< 0.1
Sprr2a3	ENSMUST00000090872.5	small proline-rich protein 2A3	7	1	0	1	0	1	mmu-miR-214-3p	0.0136	0.0952	0.521	-0.36	-0.36	< 0.1
Ldha	ENSMUST00000048209.10	lactate dehydrogenase A	147830	2	0	2	0	0	mmu-miR-214-3p	0.0134	0.0951	0.5403	-0.38	-0.38	< 0.1

Trim31	ENSMUST00000078438.4	tripartite motif-containing 31	6	1	1	0	0	1	mmu-miR-214-3p	0.0132	0.0934	0.5304	-0.44	-0.44	< 0.1
Eef1d	ENSMUST00000154584.2	eukaryotic translation elongation factor 1 delta (guanine nucleotide	3254	2	1	1	0	0	mmu-miR-214-3p	0.0136	0.0928	0.4446	-0.56	-0.83	< 0.1
Ccndbp1	ENSMUST00000060455.9	cyclin D-type binding-protein 1	2633	1	0	1	0	0	mmu-miR-214-3p	0.0136	0.0924	0.4323	-0.42	-0.42	< 0.1
Gm648	ENSMUST00000081133.4	predicted gene 648	7	1	0	1	0	0	mmu-miR-214-3p	0.0135	0.0924	0.4518	-0.33	-0.33	< 0.1
Speer4b	ENSMUST00000053257.4	spermatogenesis associated glutamate (E)-rich	5	2	0	1	1	1	mmu-miR-214-3p	0.0128	0.0922	0.5893	-0.53	-0.53	< 0.1
Polr2l	ENSMUST00000043870.8	polymerase (RNA) II (DNA directed) polypeptide L	4695	1	0	1	0	1	mmu-miR-214-3p	0.0132	0.092	0.4805	-0.35	-0.4	< 0.1
Pcbp1	ENSMUST00000053015.6	poly(rC) binding protein 1	4070	1	0	1	0	0	mmu-miR-214-3p	0.0134	0.0918	0.4357	-0.34	-0.34	< 0.1
Gm12830	ENSMUST00000137570.1	predicted gene 12830	5	1	1	0	0	0	mmu-miR-214-3p	0.0133	0.0915	0.4506	-0.32	-0.32	< 0.1
Eppin	ENSMUST00000103100.1	epididymal peptidase inhibitor	6	1	0	1	0	1	mmu-miR-214-3p	0.0134	0.0913	0.4337	-0.32	-0.35	< 0.1
Tspan17	ENSMUST00000026993.8	tetraspanin 17	858	2	0	2	0	0	mmu-miR-214-3p	0.0127	0.091	0.5624	-0.33	-0.33	< 0.1
Timp4	ENSMUST00000032462.6	tissue inhibitor of metalloproteinase 4	8075	1	0	1	0	2	mmu-miR-214-3p	0.0128	0.0909	0.524	-0.4	-0.48	< 0.1
Ly6i	ENSMUST00000023250.5	lymphocyte antigen 6 complex, locus I	111	2	0	1	1	0	mmu-miR-214-3p	0.0128	0.0903	0.5076	-0.45	-0.45	< 0.1

Tex101	ENSMUST00000078001.5	testis expressed gene 101	5	1	1	0	0	0	mmu-miR-214-3p	0.013	0.0902	0.4657	-0.46	-0.46	< 0.1
Pdrg1	ENSMUST00000028972.8	p53 and DNA damage regulated 1	3002	2	0	2	0	0	mmu-miR-214-3p	0.0127	0.09	0.5088	-0.44	-0.45	< 0.1
Tmem258	ENSMUST00000040372.8	transmembrane protein 258	110	2	0	1	1	0	mmu-miR-214-3p	0.0131	0.0894	0.4229	-0.45	-0.61	< 0.1
Aldh3a1	ENSMUST00000108716.2	aldehyde dehydrogenase family 3, subfamily A1	62	1	0	1	0	1	mmu-miR-214-3p	0.0126	0.089	0.5067	-0.33	-0.34	< 0.1
Tff3	ENSMUST00000024827.4	trefoil factor 3, intestinal	72	2	0	2	0	0	mmu-miR-214-3p	0.0125	0.0883	0.4965	-0.54	-0.54	< 0.1
Ndufab1	ENSMUST00000033157.4	NADH dehydrogenase (ubiquinone) 1, alpha/beta subcomplex, 1	572	2	0	0	2	1	mmu-miR-214-3p	0.0122	0.088	0.5432	-0.3	-0.32	< 0.1
Tppp3	ENSMUST00000014990.7	tubulin polymerization-promoting protein family member 3	1291	1	0	1	0	2	mmu-miR-214-3p	0.0121	0.0875	0.5508	-0.38	-0.38	< 0.1
Sprr2i	ENSMUST00000047264.2	small proline-rich protein 2I	5	1	0	1	0	0	mmu-miR-214-3p	0.0128	0.0872	0.4139	-0.34	-0.34	< 0.1
Gm21761	ENSMUST00000187530.1	predicted gene, 21761	5	1	0	1	0	0	mmu-miR-214-3p	0.0122	0.0861	0.4892	-0.37	-0.37	< 0.1
Pla2g1b	ENSMUST00000145785.2	phospholipase A2, group IB, pancreas	238	1	0	1	0	0	mmu-miR-214-3p	0.0125	0.0859	0.423	-0.45	-0.47	< 0.1
Lin28a	ENSMUST00000051674.2	lin-28 homolog A (C. elegans)	1253	3	0	2	1	1	mmu-miR-214-3p	0.0119	0.0853	0.5233	-0.3	-0.3	< 0.1

Trim61	ENSMUST00000124790.2	tripartite motif-containing 61	161	1	0	1	0	0	mmu-miR-214-3p	0.0122	0.0836	0.4002	-0.36	-0.37	< 0.1
Gm20878	ENSMUST00000153997.2	predicted gene, 20878	5	3	0	1	2	2	mmu-miR-214-3p	0.0118	0.0835	0.472	-0.38	-0.51	< 0.1
Rps2	ENSMUST00000054289.7	ribosomal protein S2	105	1	0	0	1	0	mmu-miR-214-3p	0.012	0.0822	0.3981	-0.3	-0.3	< 0.1
BC147527	ENSMUST00000081558.7	cDNA sequence BC147527	0	1	0	1	0	0	mmu-miR-214-3p	0.0115	0.0818	0.4662	-0.35	-0.35	< 0.1
Crygf	ENSMUST00000114027.2	crystallin, gamma F	5	0	0	0	0	0	mmu-miR-214-3p	0.0118	0.0814	0.3996	-0.48	-0.48	< 0.1
1500009L16Rik	ENSMUST00000150459.1	RIKEN cDNA 1500009L16 gene	265	1	0	1	0	0	mmu-miR-214-3p	0.0117	0.0806	0.3932	-0.35	-0.35	< 0.1
AF067061	ENSMUST00000181748.1	cDNA sequence AF067061	5	1	0	1	0	0	mmu-miR-214-3p	0.0113	0.0803	0.4658	-0.33	-0.33	< 0.1
Ppap2a	ENSMUST00000016144.5	phosphatidic acid phosphatase type 2A	3831	1	0	1	0	1	mmu-miR-214-3p	0.0114	0.08	0.4316	-0.38	-0.38	< 0.1
Hmga1-rs1	ENSMUST00000105046.3	high mobility group AT-hook I, related sequence 1	6	3	0	3	0	2	mmu-miR-214-3p	0.011	0.0799	0.5105	-0.32	-0.43	< 0.1
Hmga1	ENSMUST00000117600.2	high mobility group AT-hook 1	8	2	0	2	0	1	mmu-miR-214-3p	0.0111	0.0798	0.4954	-0.37	-0.38	< 0.1
Gm7361	ENSMUST00000074148.6	predicted gene 7361	5	1	0	1	0	2	mmu-miR-214-3p	0.011	0.0797	0.5158	-0.33	-0.33	< 0.1
Safb2	ENSMUST00000075510.6	scaffold attachment factor B2	1343	1	0	1	0	2	mmu-miR-214-3p	0.0111	0.0792	0.4793	-0.31	-0.36	< 0.1

Gm20767	ENSMUST00000179071.1	predicted gene, 20767	0	1	0	1	0	0	mmu-miR-214-3p	0.0112	0.079	0.446	-0.35	-0.35	< 0.1
Urm1	ENSMUST00000091142.3	ubiquitin related modifier 1 homolog (S. cerevisiae)	556	2	1	1	0	0	mmu-miR-214-3p	0.0114	0.0789	0.4072	-0.3	-0.6	< 0.1
Ccdc130	ENSMUST00000005120.6	coiled-coil domain containing 130	714	1	1	0	0	2	mmu-miR-214-3p	0.0113	0.0778	0.389	-0.32	-0.32	< 0.1
Rnf13	ENSMUST00000041826.9	ring finger protein 13	4649	1	1	0	0	0	mmu-miR-214-3p	0.0109	0.0771	0.433	-0.3	-0.3	< 0.1
AK010878	ENSMUST00000173969.1	cDNA sequence AK010878	73	1	0	1	0	1	mmu-miR-214-3p	0.0108	0.0765	0.4398	-0.35	-0.35	< 0.1
Sprr2e	ENSMUST00000068399.1	small proline-rich protein 2E	5	2	0	1	1	0	mmu-miR-214-3p	0.0107	0.0754	0.4274	-0.53	-0.53	< 0.1
Znhit3	ENSMUST00000103195.4	zinc finger, HIT type 3	970	1	0	1	0	0	mmu-miR-214-3p	0.0107	0.0751	0.4144	-0.31	-0.31	< 0.1
Trappc3	ENSMUST00000030660.8	trafficking protein particle complex 3	148	2	0	2	0	0	mmu-miR-214-3p	0.0105	0.0746	0.4419	-0.38	-0.4	< 0.1
Fgf2	ENSMUST00000138563.3	fibroblast growth factor 2	5	2	0	2	0	0	mmu-miR-214-3p	0.0104	0.0744	0.4505	-0.42	-0.42	< 0.1
Tubb4a	ENSMUST00000071135.5	tubulin, beta 4A class IVA	19	1	1	0	0	0	mmu-miR-214-3p	0.0105	0.0738	0.4156	-0.36	-0.36	< 0.1
Fgf20	ENSMUST00000034014.8	fibroblast growth factor 20	7	2	0	1	1	0	mmu-miR-214-3p	0.0102	0.0727	0.4346	-0.46	-0.46	< 0.1
Cyp2c29	ENSMUST00000003137.9	cytochrome P450, family 2, subfamily c, polypeptide 29	8041	1	0	1	0	0	mmu-miR-214-3p	0.0101	0.0721	0.4321	-0.32	-0.32	< 0.1

1110007C09Rik	ENSMUST00000048946.6	RIKEN cDNA 1110007C09 gene	97	1	0	1	0	0	mmu-miR-214-3p	0.01	0.071	0.4062	-0.33	-0.34	< 0.1
Il25	ENSMUST00000037863.5	interleukin 25	8	1	1	0	0	0	mmu-miR-214-3p	0.01	0.0698	0.3597	-0.37	-0.37	< 0.1
Iqcf3	ENSMUST00000062917.9	IQ motif containing F3	5	1	1	0	0	2	mmu-miR-214-3p	0.0098	0.0695	0.4067	-0.35	-0.35	< 0.1
Sgca	ENSMUST00000103162.2	sarcoglycan, alpha (dystrophin-associated glycoprotein)	213	1	0	1	0	2	mmu-miR-214-3p	0.0096	0.0681	0.4014	-0.3	-0.32	< 0.1
Ly6k	ENSMUST00000168815.2	lymphocyte antigen 6 complex, locus K	20	1	0	1	0	0	mmu-miR-214-3p	0.0095	0.068	0.4066	-0.39	-0.39	< 0.1
Vps45	ENSMUST00000015891.5	vacuolar protein sorting 45 (yeast)	588	1	1	0	0	1	mmu-miR-214-3p	0.0096	0.0678	0.389	-0.33	-0.33	< 0.1
Hook2	ENSMUST00000064495.6	hook homolog 2 (Drosophila)	618	2	0	2	0	0	mmu-miR-214-3p	0.0096	0.0677	0.3797	-0.4	-0.4	< 0.1
Fbxw4	ENSMUST00000160018.2	F-box and WD-40 domain protein 4	39	1	1	0	0	0	mmu-miR-214-3p	0.0094	0.0674	0.4172	-0.45	-0.45	< 0.1
Uqcrc1	ENSMUST00000026743.8	ubiquinol-cytochrome c reductase core protein 1	10630	1	0	1	0	0	mmu-miR-214-3p	0.0093	0.0656	0.3742	-0.32	-0.32	< 0.1
Tnnt1	ENSMUST00000071798.7	troponin T1, skeletal, slow	179	1	0	1	0	0	mmu-miR-214-3p	0.0094	0.0655	0.3412	-0.33	-0.33	< 0.1
Nbl1	ENSMUST00000042844.6	neuroblastoma, suppression of tumorigenicity 1	104	2	0	1	1	0	mmu-miR-214-3p	0.009	0.0651	0.423	-0.35	-0.36	< 0.1
Cox14	ENSMUST00000023761.2	cytochrome c oxidase assembly protein 14	1099	1	0	1	0	0	mmu-miR-214-3p	0.0093	0.0648	0.3383	-0.36	-0.36	< 0.1

Olfr653	ENSMUST00000098174.2	olfactory receptor 653	5	1	0	0	1	0	mmu-miR-214-3p	0.009	0.0638	0.3584	-0.3	-0.3	< 0.1
Lyl1	ENSMUST00000037165.4	lymphoblastomic leukemia 1	434	2	0	2	0	0	mmu-miR-214-3p	0.0087	0.0623	0.3757	-0.31	-0.32	< 0.1
Tmem191c	ENSMUST00000159242.2	transmembrane protein 191C	49	1	1	0	0	0	mmu-miR-214-3p	0.0087	0.062	0.3681	-0.42	-0.47	< 0.1
Pxdc1	ENSMUST00000053459.8	PX domain containing 1	383	1	1	0	0	0	mmu-miR-214-3p	0.0086	0.0616	0.3666	-0.33	-0.33	< 0.1
Idh1	ENSMUST00000097709.5	isocitrate dehydrogenase 1 (NADP+), soluble	16304	2	0	1	1	0	mmu-miR-214-3p	0.0088	0.0614	0.3275	-0.4	-0.41	< 0.1
Pkig	ENSMUST00000164399.2	protein kinase inhibitor, gamma	9	1	0	1	0	0	mmu-miR-214-3p	0.0087	0.0611	0.3247	-0.37	-0.37	< 0.1
Eif3g	ENSMUST00000004206.8	eukaryotic translation initiation factor 3, subunit G	87	1	0	1	0	0	mmu-miR-214-3p	0.0087	0.0611	0.3303	-0.37	-0.37	< 0.1
Ypel4	ENSMUST00000090729.3	yippee-like 4 (Drosophila)	7	1	1	0	0	0	mmu-miR-214-3p	0.0087	0.0608	0.3271	-0.31	-0.31	< 0.1
Krtap6-3	ENSMUST00000185581.1	keratin associated protein 6-3	5	1	0	1	0	0	mmu-miR-214-3p	0.0086	0.0602	0.3212	-0.38	-0.38	< 0.1
Slamf1	ENSMUST00000015460.4	signaling lymphocytic activation molecule family member 1	9	1	0	0	1	4	mmu-miR-214-3p	0.0082	0.0597	0.3972	-0.32	-0.4	< 0.1
B020031M17Rik	ENSMUST00000180768.1	RIKEN cDNA B020031M17 gene	5	1	0	1	0	0	mmu-miR-214-3p	0.0084	0.0592	0.3217	-0.33	-0.33	< 0.1

Acaa2	ENSMUST00000041053.9	acetyl-Coenzyme A acyltransferase 2 (mitochondrial 3-oxoacyl-Coenzyme A thiolase)	38987	1	1	0	0	0	mmu-miR-214-3p	0.0081	0.0577	0.3335	-0.4	-0.4	< 0.1
Atp6v0e2	ENSMUST00000040361.5	ATPase, H+ transporting, lysosomal V0 subunit E2	563	5	0	3	2	2	mmu-miR-214-3p	0.008	0.0576	0.3721	-0.4	-0.74	< 0.1
Dppa5a	ENSMUST00000071991.5	developmental pluripotency associated 5A	5	1	0	1	0	0	mmu-miR-214-3p	0.0082	0.0575	0.3179	-0.33	-0.33	< 0.1
Scgb2b27	ENSMUST00000185399.1	secretoglobin, family 2B, member 27	5	1	1	0	0	0	mmu-miR-214-3p	0.0079	0.0567	0.3463	-0.3	-0.3	< 0.1
Lce3e	ENSMUST00000098886.4	late cornified envelope 3E	5	1	1	0	0	0	mmu-miR-214-3p	0.0078	0.0561	0.3425	-0.49	-0.49	< 0.1
Gm8879	ENSMUST00000170260.2	predicted gene 8879	5	1	1	0	0	0	mmu-miR-214-3p	0.0077	0.055	0.3372	-0.36	-0.36	< 0.1
Smim22	ENSMUST00000184256.2	small integral membrane protein 22	709	1	0	1	0	0	mmu-miR-214-3p	0.0077	0.0546	0.3062	-0.3	-0.3	< 0.1
Gm5861	ENSMUST00000168407.2	predicted gene 5861	5	1	1	0	0	0	mmu-miR-214-3p	0.0076	0.0545	0.334	-0.36	-0.36	< 0.1
4933402N22Rik	ENSMUST00000170301.1	RIKEN cDNA 4933402N22 gene	5	1	1	0	0	0	mmu-miR-214-3p	0.0075	0.0536	0.3269	-0.37	-0.37	< 0.1
Gm6465	ENSMUST00000168154.1	predicted gene 6465	5	1	1	0	0	0	mmu-miR-214-3p	0.0075	0.0535	0.3262	-0.35	-0.35	< 0.1
Gm6455	ENSMUST00000168057.2	predicted gene 6455	5	1	1	0	0	0	mmu-miR-214-3p	0.0075	0.0534	0.326	-0.43	-0.43	< 0.1

Speer1-ps1	ENSMUST00000172106.3	spermatogenesis associated glutamate (E)-rich protein 1,	5	1	1	0	0	0	mmu-miR-214-3p	0.0074	0.0534	0.3261	-0.36	-0.36	< 0.1
Gm8857	ENSMUST00000170064.1	predicted gene 8857	5	1	1	0	0	0	mmu-miR-214-3p	0.0075	0.0534	0.326	-0.34	-0.34	< 0.1
Gm8890	ENSMUST00000164262.2	predicted gene 8890	5	1	1	0	0	0	mmu-miR-214-3p	0.0074	0.0531	0.3246	-0.36	-0.36	< 0.1
Proca1	ENSMUST00000060539.7	protein interacting with cyclin A1	104	1	1	0	0	0	mmu-miR-214-3p	0.0074	0.0519	0.288	-0.47	-0.47	< 0.1
1810043H04Rik	ENSMUST00000106223.3	RIKEN cDNA 1810043H04 gene	1746	3	1	2	0	0	mmu-miR-214-3p	0.0073	0.0515	0.2856	-0.33	-0.85	< 0.1
2810004N23Rik	ENSMUST00000034465.7	RIKEN cDNA 2810004N23 gene	124	2	2	0	0	0	mmu-miR-214-3p	0.0068	0.0491	0.3136	-0.31	-0.31	< 0.1
Ndufa2	ENSMUST00000014438.4	NADH dehydrogenase (ubiquinone) 1 alpha subcomplex,	32591	1	0	1	0	0	mmu-miR-214-3p	0.0069	0.0489	0.28	-0.34	-0.34	< 0.1
Prr13	ENSMUST00000023810.6	proline rich 13	3553	1	0	1	0	1	mmu-miR-214-3p	0.0068	0.0485	0.2913	-0.35	-0.35	< 0.1
Bpifa2	ENSMUST00000048103.3	BPI fold containing family A, member 2	9	1	0	1	0	0	mmu-miR-214-3p	0.0067	0.0473	0.2731	-0.3	-0.3	< 0.1
1700042G07Rik	ENSMUST00000106492.2	RIKEN cDNA 1700042G07 gene	5	1	0	1	0	0	mmu-miR-214-3p	0.0063	0.0448	0.2572	-0.41	-0.41	< 0.1
Gadd45gip1	ENSMUST00000036734.5	growth arrest and DNA-damage-inducible, gamma interacting protein 1	1290	1	0	0	1	0	mmu-miR-214-3p	0.006	0.0436	0.2896	-0.35	-0.35	< 0.1

Speer4f	ENSMUST00000076099.3	spermatogenesis associated glutamate (E)-rich	5	2	2	0	0	0	mmu-miR-214-3p	0.0059	0.0434	0.2978	-1.05	-1.05	< 0.1
Stub1	ENSMUST00000044911.8	STIP1 homology and U-Box containing protein 1	92	1	1	0	0	1	mmu-miR-214-3p	0.006	0.0432	0.2731	-0.33	-0.35	< 0.1
Ly6g	ENSMUST00000190262.1	lymphocyte antigen 6 complex, locus G	8	1	0	1	0	0	mmu-miR-214-3p	0.0061	0.0432	0.2504	-0.3	-0.3	< 0.1
Olfr59	ENSMUST00000119717.1	olfactory receptor 59	0	1	0	0	1	0	mmu-miR-214-3p	0.0058	0.0422	0.2741	-0.37	-0.37	< 0.1
Gm6460	ENSMUST00000163787.2	predicted gene 6460	5	1	1	0	0	0	mmu-miR-214-3p	0.0059	0.0417	0.2433	-0.45	-0.45	< 0.1
Ndufa4	ENSMUST00000031637.5	NADH dehydrogenase (ubiquinone) 1 alpha subcomplex,	1662	1	0	0	1	0	mmu-miR-214-3p	0.0056	0.0398	0.2363	-0.31	-0.31	< 0.1
Cstl1	ENSMUST00000109955.4	cystatin-like 1	5	1	0	0	1	0	mmu-miR-214-3p	0.004	0.029	0.1842	-0.3	-0.3	< 0.1
Scgb3a1	ENSMUST00000043873.4	secretoglobin, family 3A, member 1	791	1	0	1	0	0	mmu-miR-214-3p	0.0035	0.0256	0.1708	-0.32	-0.32	< 0.1
Mrgpra9	ENSMUST00000179005.2	MAS-related GPR, member A9	5	1	0	1	0	0	mmu-miR-214-3p	0.0032	0.0231	0.1533	-0.31	-0.31	< 0.1
