# Supplementary material for: Bioinformatic prediction, qCLASH, and AlphaFold3 reveal non-overlapping layers of the miR-214-3p–mRNA interactome
Source: Front Genet. 2026 Sep 30;17:1924314. doi: 10.3389/fgene.2026.1924314 (PMC13661022; doi:10.3389/fgene.2026.1924314)
Supplement: Supplementary file 2 [file DataSheet10.pdf]

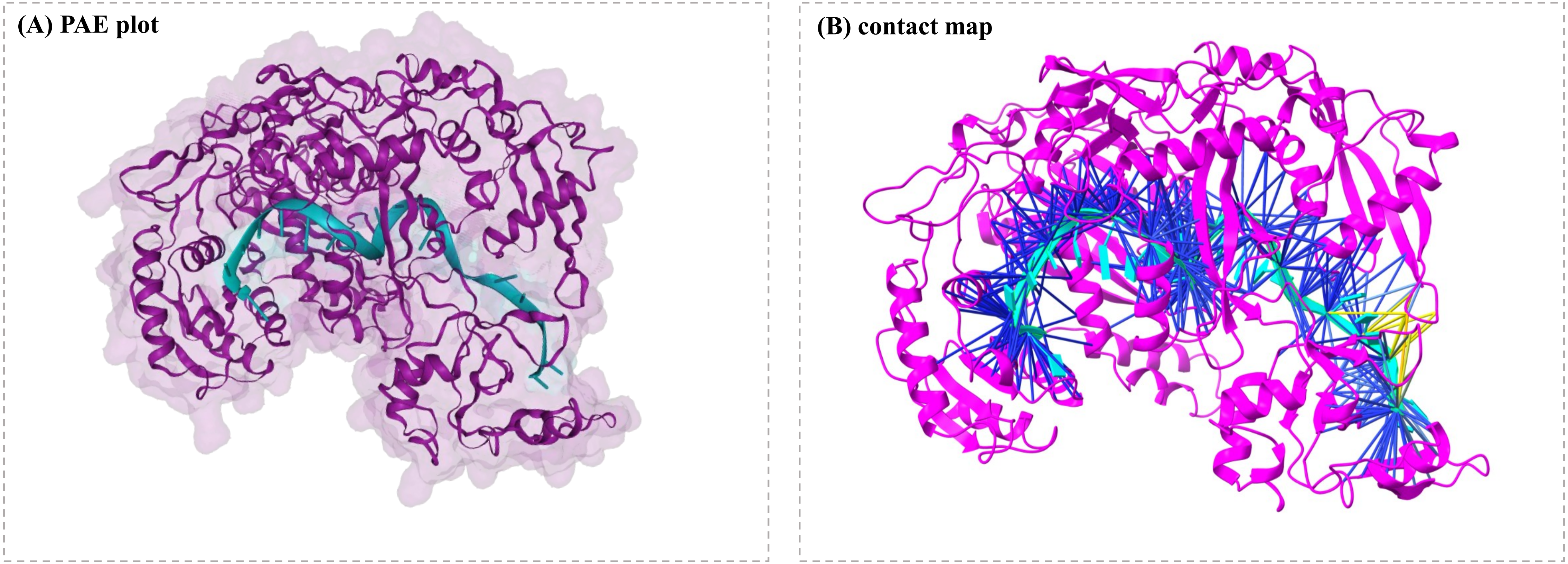

Appendix Figure 2. Positive control for AlphaFold3 structural prediction.

(A) PAE plot for the AlphaFold3 prediction of the human AGO2–guide RNA complex (PDB: 4W5N). The prediction achieved ipTM = 0.95 and pTM = 0.93. Purple represents AGO protein, turquoise represents RNA.

(B) contact map showing spatial contacts between AGO2 (chain A, purple) and guide RNA (chain B, turquoise) within 8 Å (blue lines). Comparison with the crystal structure using US-align gave a TM-score of 0.956 and an RMSD of 2.19 Å, confirming that AlphaFold3 can reliably recapitulate the architecture of an AGO2–nucleic acid complex under our parameter settings.
