# Supplementary material for: Bioinformatic prediction, qCLASH, and AlphaFold3 reveal non-overlapping layers of the miR-214-3p–mRNA interactome
Source: Front Genet. 2026 Sep 30;17:1924314. doi: 10.3389/fgene.2026.1924314 (PMC13661022; doi:10.3389/fgene.2026.1924314)
Supplement: Supplementary file 3 [file DataSheet11.pdf]

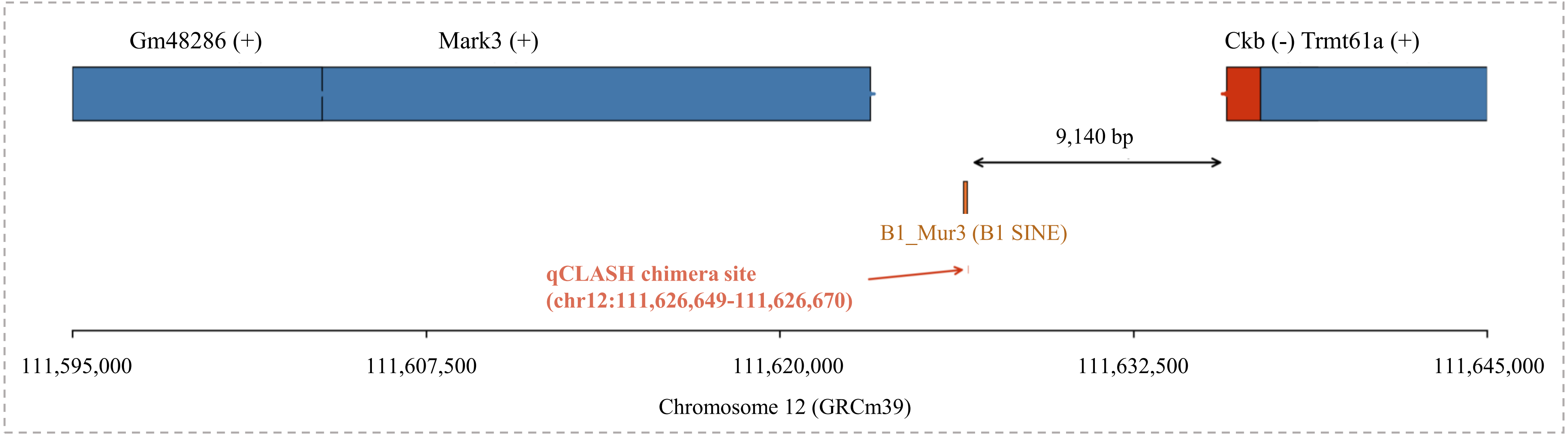

Appendix Figure 3. Genomic context of the qCLASH chimeric entry designated as unannotated gene 3.
Schematic illustration of the genomic context of the 80-read chimeric entry designated as unannotated gene 3 (chr12:111,626,649–111,626,670, GRCm39). This entry is one of three that could not be assigned to annotated protein-coding genes; the other two correspond to clone-derived sequences listed in Appendix Table 1.
