# Supplementary material for: Bioinformatic prediction, qCLASH, and AlphaFold3 reveal non-overlapping layers of the miR-214-3p–mRNA interactome
Source: Front Genet. 2026 Sep 30;17:1924314. doi: 10.3389/fgene.2026.1924314 (PMC13661022; doi:10.3389/fgene.2026.1924314)
Supplement: Supplementary file 4 [file DataSheet2.pdf]

Target Rank	Target Score	miRNA Name	Gene ID	Gene Symbol	Transcript Accession	Gene Description
1	99	mmu-miR-214-3p	269593	Luzp1	NM_024452	leucine zipper protein 1
2	99	mmu-miR-214-3p	57170	Dolpp1	NM_001290508	dolichyl pyrophosphate phosphatase 1
3	99	mmu-miR-214-3p	55988	Snx12	NM_001110310	sorting nexin 12
4	99	mmu-miR-214-3p	54188	Cpsf4	NM_001291248	cleavage and polyadenylation specific factor 4
5	99	mmu-miR-214-3p	74270	Usp20	NM_028846	ubiquitin specific peptidase 20
6	98	mmu-miR-214-3p	20354	Sema4d	NM_001281880	sema domain, immunoglobulin domain (Ig), transmembrane domain (TM) and short cytoplasmic domain, (semaphorin) 4D
7	98	mmu-miR-214-3p	74838	Naa15	NM_053089	N(alpha)-acetyltransferase 15, NatA auxiliary subunit
8	98	mmu-miR-214-3p	99377	Sall4	NM_175303	spalt like transcription factor 4
9	98	mmu-miR-214-3p	20541	Slc8a1	NM_001112798	solute carrier family 8 (sodium/calcium exchanger), member 1
10	98	mmu-miR-214-3p	55943	Stx8	NM_018768	syntaxin 8
11	98	mmu-miR-214-3p	171463	Il17rd	NM_134437	interleukin 17 receptor D
12	97	mmu-miR-214-3p	1.01E+08	Rubcn	NM_001200038	RUN domain and cysteine-rich domain containing, Beclin 1-interacting protein
13	97	mmu-miR-214-3p	225339	Ammecr1l	NM_001242430	AMME chromosomal region gene 1-like
14	97	mmu-miR-214-3p	210973	Kbtbd2	NM_145958	kelch repeat and BTB (POZ) domain containing 2
15	96	mmu-miR-214-3p	14182	Fgfr1	NM_001079908	fibroblast growth factor receptor 1
16	96	mmu-miR-214-3p	268480	Rapgefl1	NM_001080925	Rap guanine nucleotide exchange factor (GEF)-like 1
17	96	mmu-miR-214-3p	23876	Fbln5	NM_011812	fibulin 5
18	96	mmu-miR-214-3p	72745	Tmem161b	NM_175187	transmembrane protein 161B

19	96	mmu-miR-214-3p	21769	Zfand3	NM_148926	zinc finger, AN1-type domain 3
20	96	mmu-miR-214-3p	208104	Mlxip	NM_133917	MLX interacting protein
21	96	mmu-miR-214-3p	12912	Creb1	NM_001037726	cAMP responsive element binding protein 1
22	96	mmu-miR-214-3p	104444	Rexo2	NM_024233	RNA exonuclease 2
23	95	mmu-miR-214-3p	77097	Tanc2	NM_181071	tetratricopeptide repeat, ankyrin repeat and coiled-coil containing 2
24	95	mmu-miR-214-3p	240518	Peli3	NM_172835	pellino 3
25	95	mmu-miR-214-3p	69053	1810013L24Rik	NM_001081400	RIKEN cDNA 1810013L24 gene
26	95	mmu-miR-214-3p	170947	Myoz3	NM_133363	myozenin 3
27	95	mmu-miR-214-3p	67878	Tmem33	NM_001285452	transmembrane protein 33
28	95	mmu-miR-214-3p	68564	Nufip2	NM_001024205	nuclear fragile X mental retardation protein interacting protein 2
29	95	mmu-miR-214-3p	230848	Zbtb40	NM_198248	zinc finger and BTB domain containing 40
30	95	mmu-miR-214-3p	116873	Stim2	NM_001081103	stromal interaction molecule 2
31	95	mmu-miR-214-3p	66867	Hmg20a	NM_025812	high mobility group 20A
32	94	mmu-miR-214-3p	76217	Jakmip2	NM_001163637	janus kinase and microtubule interacting protein 2
33	94	mmu-miR-214-3p	229055	Zbtb10	NM_177660	zinc finger and BTB domain containing 10
34	94	mmu-miR-214-3p	66931	1700010I14Rik	NM_025851	RIKEN cDNA 1700010I14 gene
35	94	mmu-miR-214-3p	232237	Fgd5	NM_172731	FYVE, RhoGEF and PH domain containing 5
36	94	mmu-miR-214-3p	116848	Baz2a	NM_054078	bromodomain adjacent to zinc finger domain, 2A
37	94	mmu-miR-214-3p	268490	Lsm12	NM_172947	LSM12 homolog

38	94	mmu-miR-214-3p	218811	Sec24c	NM_001168273	Sec24 related gene family, member C (S. cerevisiae)
39	93	mmu-miR-214-3p	14169	Fgf14	NM_010201	fibroblast growth factor 14
40	93	mmu-miR-214-3p	319262	Fchsd1	NM_175684	FCH and double SH3 domains 1
41	93	mmu-miR-214-3p	360013	Myo18a	NM_001291212	myosin XVIIIA
42	93	mmu-miR-214-3p	215476	Prr14l	NM_194340	proline rich 14-like
43	93	mmu-miR-214-3p	209225	Zfp710	NM_001145999	zinc finger protein 710
44	93	mmu-miR-214-3p	17354	Mllt10	NM_010804	myeloid/lymphoid or mixed-lineage leukemia; translocated to, 10
45	93	mmu-miR-214-3p	209224	Enox2	NM_001271449	ecto-NOX disulfide-thiol exchanger 2
46	92	mmu-miR-214-3p	72832	Crtac1	NM_145123	cartilage acidic protein 1
47	92	mmu-miR-214-3p	16576	Kif7	NM_001291222	kinesin family member 7
48	92	mmu-miR-214-3p	68559	Pdrg1	NM_178939	p53 and DNA damage regulated 1
49	92	mmu-miR-214-3p	214345	Lrrc1	NM_001146048	leucine rich repeat containing 1
50	92	mmu-miR-214-3p	79455	Pdcl2	NM_001310637	phosducin-like 2
51	92	mmu-miR-214-3p	56327	Arl2	NM_019722	ADP-ribosylation factor-like 2
52	92	mmu-miR-214-3p	20501	Slc16a1	NM_009196	solute carrier family 16 (monocarboxylic acid transporters), member 1
53	92	mmu-miR-214-3p	14055	Ezh1	NM_007970	enhancer of zeste 1 polycomb repressive complex 2 subunit
54	92	mmu-miR-214-3p	74563	Rasgef1c	NM_029004	RasGEF domain family, member 1C
55	92	mmu-miR-214-3p	241589	D430041D05Rik	NM_001033347	RIKEN cDNA D430041D05 gene
56	92	mmu-miR-214-3p	26370	Cetn2	NM_019405	centrin 2

57	91	mmu-miR-214-3p	18712	Pim1	NM_008842	proviral integration site 1
58	91	mmu-miR-214-3p	68618	1110012L19Rik	NM_026787	RIKEN cDNA 1110012L19 gene
59	91	mmu-miR-214-3p	211446	Exoc3	NM_177333	exocyst complex component 3
60	91	mmu-miR-214-3p	66932	Rexo1	NM_025852	REX1, RNA exonuclease 1
61	91	mmu-miR-214-3p	80879	Slc16a3	NM_001038653	solute carrier family 16 (monocarboxylic acid transporters), member 3
62	91	mmu-miR-214-3p	71562	Afmid	NM_027827	arylformamidase
63	91	mmu-miR-214-3p	170719	Oxr1	NM_001130163	oxidation resistance 1
64	91	mmu-miR-214-3p	14457	Gas7	NM_008088	growth arrest specific 7
65	91	mmu-miR-214-3p	18654	Pgf	NM_001271705	placental growth factor
66	91	mmu-miR-214-3p	108150	Galnt7	NM_001167981	polypeptide N-acetylgalactosaminyltransferase 7
67	91	mmu-miR-214-3p	72117	Naa50	NM_028108	N(alpha)-acetyltransferase 50, NatE catalytic subunit
68	91	mmu-miR-214-3p	66328	Scp2d1	NM_025490	SCP2 sterol-binding domain containing 1
69	91	mmu-miR-214-3p	18521	Pcbp2	NM_001103165	poly(rC) binding protein 2
70	91	mmu-miR-214-3p	18019	Nfatc2	NM_001291171	nuclear factor of activated T cells, cytoplasmic, calcineurin dependent 2
71	91	mmu-miR-214-3p	381379	Med19	NM_025885	mediator complex subunit 19
72	91	mmu-miR-214-3p	104457	0610010K14Rik	NM_145758	RIKEN cDNA 0610010K14 gene
73	90	mmu-miR-214-3p	13589	Mapre1	NM_007896	microtubule-associated protein, RP/EB family, member 1
74	90	mmu-miR-214-3p	26419	Mapk8	NM_001310452	mitogen-activated protein kinase 8
75	90	mmu-miR-214-3p	51810	Hnrnpu	NM_016805	heterogeneous nuclear ribonucleoprotein U

76	90	mmu-miR-214-3p	330627	Trim66	NM_001170912	tripartite motif-containing 66
77	90	mmu-miR-214-3p	226594	Rcsd1	NM_178593	RCSD domain containing 1
78	90	mmu-miR-214-3p	66724	Tab3	NM_025729	TGF-beta activated kinase 1/MAP3K7 binding protein 3
79	90	mmu-miR-214-3p	68498	Tspan11	NM_026743	tetraspanin 11
80	90	mmu-miR-214-3p	216705	Clint1	NM_001045520	clathrin interactor 1
81	90	mmu-miR-214-3p	19317	Qk	NM_021881	quaking
82	90	mmu-miR-214-3p	226470	Zbtb41	NM_172643	zinc finger and BTB domain containing 41
83	90	mmu-miR-214-3p	12402	Cbl	NM_007619	Casitas B-lineage lymphoma
84	90	mmu-miR-214-3p	68597	Ccdc167	NM_026782	coiled-coil domain containing 167
85	90	mmu-miR-214-3p	71667	Tmem248	NM_001081394	transmembrane protein 248
86	90	mmu-miR-214-3p	214812	Zfp609	NM_172536	zinc finger protein 609
87	89	mmu-miR-214-3p	12802	Cnr2	NM_009924	cannabinoid receptor 2 (macrophage)
88	89	mmu-miR-214-3p	217431	Nol10	NM_001008421	nucleolar protein 10
89	89	mmu-miR-214-3p	81004	Tbl1xr1	NM_030732	transducin (beta)-like 1X-linked receptor 1
90	89	mmu-miR-214-3p	72649	Tmem209	NM_001311093	transmembrane protein 209
91	89	mmu-miR-214-3p	70297	Gcc2	NM_027375	GRIP and coiled-coil domain containing 2
92	89	mmu-miR-214-3p	54711	Plagl2	NM_018807	pleiomorphic adenoma gene-like 2
93	89	mmu-miR-214-3p	68318	Aph1c	NM_026674	aph1 homolog C, gamma secretase subunit
94	89	mmu-miR-214-3p	74254	Gpn1	NM_133756	GPN-loop GTPase 1

95	89	mmu-miR-214-3p	66717	Ccdc96	NM_025725	coiled-coil domain containing 96
96	89	mmu-miR-214-3p	78428	Pym1	NM_001253705	PYM homolog 1, exon junction complex associated factor
97	88	mmu-miR-214-3p	19210	Ptdss1	NM_008959	phosphatidylserine synthase 1
98	88	mmu-miR-214-3p	19271	Ptprj	NM_008982	protein tyrosine phosphatase, receptor type, J
99	88	mmu-miR-214-3p	66914	Vps28	NM_001305668	vacuolar protein sorting 28
100	88	mmu-miR-214-3p	13388	Dll1	NM_007865	delta like canonical Notch ligand 1
101	88	mmu-miR-214-3p	22413	Wnt2	NM_023653	wingless-type MMTV integration site family, member 2
102	88	mmu-miR-214-3p	16646	Kpna1	NM_008465	karyopherin (importin) alpha 1
103	88	mmu-miR-214-3p	114604	Prdm15	NM_144789	PR domain containing 15
104	88	mmu-miR-214-3p	230796	Wdtc1	NM_199306	WD and tetratricopeptide repeats 1
105	88	mmu-miR-214-3p	17534	Mrc2	NM_008626	mannose receptor, C type 2
106	87	mmu-miR-214-3p	76582	Ipo11	NM_029665	importin 11
107	87	mmu-miR-214-3p	12125	Bcl2l11	NM_207681	BCL2-like 11 (apoptosis facilitator)
108	87	mmu-miR-214-3p	67425	Eps8l1	NM_026146	EPS8-like 1
109	87	mmu-miR-214-3p	72787	Ndc1	NM_028355	NDC1 transmembrane nucleoporin
110	87	mmu-miR-214-3p	320080	Zbtb39	NM_198035	zinc finger and BTB domain containing 39
111	87	mmu-miR-214-3p	207393	Elfn2	NM_183141	leucine rich repeat and fibronectin type III, extracellular 2
112	87	mmu-miR-214-3p	22415	Wnt3	NM_009521	wingless-type MMTV integration site family, member 3
113	87	mmu-miR-214-3p	83797	Smarcd1	NM_031842	SWI/SNF related, matrix associated, actin dependent regulator of chromatin, subfamily d, member 1

114	87	mmu-miR-214-3p	15460	Hr	NM_021877	lysine demethylase and nuclear receptor corepressor
115	87	mmu-miR-214-3p	59289	Ackr2	NM_001276719	atypical chemokine receptor 2
116	87	mmu-miR-214-3p	19230	Twf1	NM_008971	twinfilin actin binding protein 1
117	87	mmu-miR-214-3p	68039	Nmb	NM_026523	neuromedin B
118	87	mmu-miR-214-3p	110006	Gusb	NM_010368	glucuronidase, beta
119	87	mmu-miR-214-3p	15507	Hspb1	NM_013560	heat shock protein 1
120	87	mmu-miR-214-3p	235472	Prtg	NM_175485	protogenin
121	87	mmu-miR-214-3p	53321	Cntnap1	NM_016782	contactin associated protein-like 1
122	87	mmu-miR-214-3p	12503	Cd247	NM_001113391	CD247 antigen
123	86	mmu-miR-214-3p	14573	Gdnf	NM_001301332	glial cell line derived neurotrophic factor
124	86	mmu-miR-214-3p	235386	Hykk	NM_177351	hydroxylysine kinase 1
125	86	mmu-miR-214-3p	434784	Ldoc1	NM_001018087	regulator of NFKB signaling
126	86	mmu-miR-214-3p	11877	Arvcf	NM_001272028	armadillo repeat gene deleted in velocardiofacial syndrome
127	86	mmu-miR-214-3p	230279	Tmem268	NM_144905	transmembrane protein 268
128	86	mmu-miR-214-3p	22134	Tgoln1	NM_009443	trans-golgi network protein
129	86	mmu-miR-214-3p	16874	Lhx6	NM_001083126	LIM homeobox protein 6
130	86	mmu-miR-214-3p	67040	Ddx17	NM_199079	DEAD (Asp-Glu-Ala-Asp) box polypeptide 17
131	86	mmu-miR-214-3p	107047	Psmg2	NM_134138	proteasome (prosome, macropain) assembly chaperone 2
132	86	mmu-miR-214-3p	223881	Rnd1	NM_172612	Rho family GTPase 1

133	86	mmu-miR-214-3p	245049	Myrip	NM_144557	myosin VIIA and Rab interacting protein
134	86	mmu-miR-214-3p	19212	Pter	NM_008961	phosphotriesterase related
135	85	mmu-miR-214-3p	18007	Neo1	NM_001042752	neogenin
136	85	mmu-miR-214-3p	228850	Ralgapb	NM_001291137	Ral GTPase activating protein, beta subunit (non-catalytic)
137	85	mmu-miR-214-3p	27979	Eif3b	NM_133916	eukaryotic translation initiation factor 3, subunit B
138	85	mmu-miR-214-3p	76707	Clasp1	NM_029709	CLIP associating protein 1
139	85	mmu-miR-214-3p	381305	Rc3h1	NM_001024952	RING CCCH (C3H) domains 1
140	85	mmu-miR-214-3p	70646	Naa30	NM_027492	N(alpha)-acetyltransferase 30, NatC catalytic subunit
141	85	mmu-miR-214-3p	97487	Cmtm4	NM_153582	CKLF-like MARVEL transmembrane domain containing 4
142	85	mmu-miR-214-3p	77832	Tchp	NM_029992	trichoplein, keratin filament binding
143	85	mmu-miR-214-3p	118451	Mrps2	NM_080452	mitochondrial ribosomal protein S2
144	85	mmu-miR-214-3p	270110	Irf2bp2	NM_001164598	interferon regulatory factor 2 binding protein 2
145	85	mmu-miR-214-3p	19342	Rab4b	NM_029391	RAB4B, member RAS oncogene family
146	84	mmu-miR-214-3p	52858	Cdipt	NM_026638	CDP-diacylglycerol--inositol 3-phosphatidyltransferase (phosphatidylinositol synthase)
147	84	mmu-miR-214-3p	270028	Fam155a	NM_173446	family with sequence similarity 155, member A
148	84	mmu-miR-214-3p	72590	Ppme1	NM_028292	protein phosphatase methylesterase 1
149	84	mmu-miR-214-3p	68365	Rab14	NM_026697	RAB14, member RAS oncogene family
150	84	mmu-miR-214-3p	104175	Sbk1	NM_145587	SH3-binding kinase 1
151	84	mmu-miR-214-3p	18854	Pml	NM_008884	promyelocytic leukemia

152	84	mmu-miR-214-3p	70998	Phf6	NM_027642	PHD finger protein 6
153	84	mmu-miR-214-3p	384061	Fndc5	NM_027402	fibronectin type III domain containing 5
154	84	mmu-miR-214-3p	15401	Hoxa4	NM_008265	homeobox A4
155	83	mmu-miR-214-3p	243339	Tmem130	NM_177735	transmembrane protein 130
156	83	mmu-miR-214-3p	59092	Pcbp4	NM_021567	poly(rC) binding protein 4
157	83	mmu-miR-214-3p	14025	Bcl11a	NM_001242934	B cell CLL/lymphoma 11A (zinc finger protein)
158	83	mmu-miR-214-3p	68192	Leprotl1	NM_026609	leptin receptor overlapping transcript-like 1
159	83	mmu-miR-214-3p	14555	Gpd1	NM_010271	glycerol-3-phosphate dehydrogenase 1 (soluble)
160	83	mmu-miR-214-3p	16565	Kif21b	NM_001039472	kinesin family member 21B
161	83	mmu-miR-214-3p	68646	Nadk2	NM_001286255	NAD kinase 2, mitochondrial
162	83	mmu-miR-214-3p	19291	Purb	NM_011221	purine rich element binding protein B
163	83	mmu-miR-214-3p	70078	Nol7	NM_023554	nucleolar protein 7
164	83	mmu-miR-214-3p	66593	Diablo	NM_023232	diablo, IAP-binding mitochondrial protein
165	83	mmu-miR-214-3p	75547	Akap13	NM_029332	A kinase (PRKA) anchor protein 13
166	83	mmu-miR-214-3p	14013	Mecom	NM_007963	MDS1 and EVI1 complex locus
167	83	mmu-miR-214-3p	60344	Fign	NM_001267846	fidgetin
168	82	mmu-miR-214-3p	209027	Pycr1	NM_144795	pyrroline-5-carboxylate reductase 1
169	82	mmu-miR-214-3p	27416	Abcc5	NM_013790	ATP-binding cassette, sub-family C (CFTR/MRP), member 5
170	82	mmu-miR-214-3p	74007	Btbd11	NM_028709	BTB (POZ) domain containing 11

171	82	mmu-miR-214-3p	30938	Fgd3	NM_015759	FYVE, RhoGEF and PH domain containing 3
172	82	mmu-miR-214-3p	15191	Hdgf	NM_008231	hepatoma-derived growth factor
173	82	mmu-miR-214-3p	72238	Tbc1d5	NM_001285993	TBC1 domain family, member 5
174	82	mmu-miR-214-3p	76246	Rtf1	NM_030112	RTF1, Paf1/RNA polymerase II complex component
175	82	mmu-miR-214-3p	11980	Atp8a1	NM_001284345	ATPase, aminophospholipid transporter (APLT), class I, type 8A, member 1
176	82	mmu-miR-214-3p	54725	Cadm1	NM_001310841	cell adhesion molecule 1
177	82	mmu-miR-214-3p	20681	Sox8	NM_011447	SRY (sex determining region Y)-box 8
178	82	mmu-miR-214-3p	114893	Dcun1d1	NM_001205361	DCN1, defective in cullin neddylation 1, domain containing 1 (S. cerevisiae)
179	82	mmu-miR-214-3p	81006	Gpr63	NM_030733	G protein-coupled receptor 63
180	82	mmu-miR-214-3p	19012	Plpp1	NM_008903	phospholipid phosphatase 1
181	82	mmu-miR-214-3p	320910	Itgb8	NM_177290	integrin beta 8
182	81	mmu-miR-214-3p	67857	Ppp6c	NM_024209	protein phosphatase 6, catalytic subunit
183	81	mmu-miR-214-3p	73205	C9orf72	NM_001081343	C9orf72, member of C9orf72-SMCR8 complex
184	81	mmu-miR-214-3p	239719	Mrtfb	NM_153588	myocardin related transcription factor B
185	81	mmu-miR-214-3p	234797	6430548M08Rik	NM_001163760	RIKEN cDNA 6430548M08 gene
186	81	mmu-miR-214-3p	327655	Ppip5k1	NM_178795	diphosphoinositol pentakisphosphate kinase 1
187	81	mmu-miR-214-3p	320706	Soga1	NM_001164663	suppressor of glucose, autophagy associated 1
188	81	mmu-miR-214-3p	235582	Glyctk	NM_174846	glycerate kinase
189	81	mmu-miR-214-3p	68279	Mcoln2	NM_001005846	mucolipin 2

190	81	mmu-miR-214-3p	74015	Fcho1	NM_028715	FCH domain only 1
191	81	mmu-miR-214-3p	69707	Iqcg	NM_178378	IQ motif containing G
192	81	mmu-miR-214-3p	12416	Cbx2	NM_007623	chromobox 2
193	81	mmu-miR-214-3p	219257	Pcdh20	NM_178685	protocadherin 20
194	81	mmu-miR-214-3p	232585	Vwde	NM_001013757	von Willebrand factor D and EGF domains
195	81	mmu-miR-214-3p	29806	Limd1	NM_013860	LIM domains containing 1
196	81	mmu-miR-214-3p	109246	Tspan9	NM_175414	tetraspanin 9
197	81	mmu-miR-214-3p	14299	Ncs1	NM_019681	neuronal calcium sensor 1
198	80	mmu-miR-214-3p	18391	Sigmar1	NM_001286540	sigma non-opioid intracellular receptor 1
199	80	mmu-miR-214-3p	16536	Kcnq2	NM_010611	potassium voltage-gated channel, subfamily Q, member 2
200	80	mmu-miR-214-3p	381290	Atp2b4	NM_001167949	ATPase, Ca++ transporting, plasma membrane 4
201	80	mmu-miR-214-3p	74478	Snx29	NM_028964	sorting nexin 29
202	80	mmu-miR-214-3p	12571	Cdk6	NM_009873	cyclin-dependent kinase 6
203	80	mmu-miR-214-3p	80857	Fgf20	NM_030610	fibroblast growth factor 20
204	80	mmu-miR-214-3p	14924	Magi1	NM_001286785	membrane associated guanylate kinase, WW and PDZ domain containing 1
205	80	mmu-miR-214-3p	21887	Tle3	NM_001083927	transducin-like enhancer of split 3
206	80	mmu-miR-214-3p	381338	Lonrf2	NM_001029878	LON peptidase N-terminal domain and ring finger 2
207	80	mmu-miR-214-3p	170828	Vgll1	NM_001313763	vestigial like family member 1
208	80	mmu-miR-214-3p	12273	C5ar1	NM_001173550	complement component 5a receptor 1

209	80	mmu-miR-214-3p	54004	Diaph2	NM_172493	diaphanous related formin 2
210	80	mmu-miR-214-3p	215627	Zbtb8b	NM_153541	zinc finger and BTB domain containing 8b
211	80	mmu-miR-214-3p	231821	Adap1	NM_172723	ArfGAP with dual PH domains 1
212	80	mmu-miR-214-3p	15200	Hbegf	NM_010415	heparin-binding EGF-like growth factor
213	80	mmu-miR-214-3p	56226	Espn	NM_207689	espin
