# Supplementary material for: Bioinformatic prediction, qCLASH, and AlphaFold3 reveal non-overlapping layers of the miR-214-3p–mRNA interactome
Source: Front Genet. 2026 Sep 30;17:1924314. doi: 10.3389/fgene.2026.1924314 (PMC13661022; doi:10.3389/fgene.2026.1924314)
Supplement: Supplementary file 5 [file DataSheet3.pdf]

mirnaid	refseqid	genesymbol	duplex	start	end	bindingp	energy	seed	accessibility	au	phylopstem	phyloflank	me	number_of_pairings	binding_region_length	longest_consecutive_pairing	position
mmu-miR-214-3p	NM_001001160	Fbxo41	ACAGCAGGCACAGACAGGCAGT#ACGCGGGGTCTGTGCCTCTGC#.(((.((((((((((..(((.((#))))...))))))))))))).	216	237	0.84615	-24	0	1.64E-06	0.2	0.7	1.3	-8	17	21	13	5UTR
mmu-miR-214-3p	NM_001001333	Hexdc	ACAGCAGGCACAGACAGGCAGT#GCTGGCAGAGGTACGACCACTTCTCTGTGCTGTGTGAGCTGC#.((((..(((((((((((.(((#.))).).)))).))))))..)))).	1352	1394	0.84615	-22	0	0.0002972	0.4	3.9	1.81	-7	18	25	6	CDS
mmu-miR-214-3p	NM_001001333	Hexdc	ACAGCAGGCACAGACAGGCAGT#GCTGCCTGCCTACAGTCACTTGTACATGGAGGCTTTGCTGA#.((((((((..((.((((((((#)))))))).))..................)))).)))).	1417	1458	0.84615	-21	1	9.27E-05	0.5	2.7	1.67	-9	18	41	8	CDS
mmu-miR-214-3p	NM_001001565	Chpf	ACAGCAGGCACAGACAGGCAGT#GCTGTCCAAGAAGCACCCACTAGACACTCTGTTCCTGCTGG#.(((((((.(((((..((((((#))))))....................))))).))))))).	2305	2346	1	-23	1	0.0047337	0.4	2.7	3.35	-6	18	41	7	CDS
mmu-miR-214-3p	NM_001001565	Chpf	ACAGCAGGCACAGACAGGCAGT#TTGCCTGTACCCTATGTCACCGAGGCTTCTCGGCTCACTGTGCTACTGCC#...((((((((((((((((((.#))))))))............................))))))..	1997	2047	0.92308	-23	0	0.0015873	0.4	3	2.55	-7	18	50	8	CDS
mmu-miR-214-3p	NM_001001565	Chpf	ACAGCAGGCACAGACAGGCAGT#GCTCCTGTCCTGGGAGCTGGGCTGTA#(((((.(((.((((((((.(((#))))))))))).))...)))..))))).	3093	3119	1	-22	0	6.78E-05	0.4	0.4	0.19	-7	19	26	9	3UTR
mmu-miR-214-3p	NM_001001566	Chpf	ACAGCAGGCACAGACAGGCAGT#GCTGTCCAAGAAGCACCCACTAGACACTCTGTTCCTGCTGG#.(((((((.(((((..((((((#))))))....................))))).))))))).	2162	2203	1	-23	1	0.0047337	0.4	3.3	2.84	-6	18	41	7	CDS
mmu-miR-214-3p	NM_001001566	Chpf	ACAGCAGGCACAGACAGGCAGT#TTGCCTGTACCCTATGTCACCGAGGCTTCTCGGCTCACTGTGCTACTGCC#...((((((((((((((((((.#))))))))............................))))))..	1854	1904	0.92308	-23	0	0.0015873	0.4	3	2.96	-7	18	50	8	CDS

mmu-miR-214-3p	NM_001001566	Chpf	ACAGCAGGCACAGACAGGCAGT#GCTCCTGTCCTGGGAGCTGGGCTGTA#(((((.(((.((((((((.(((#))))))))).))...)))..))))).	2950	2976	1	-22	0	6.78E-05	0.4	0.4	0.19	-7	19	26	9	3UTR
mmu-miR-214-3p	NM_001001735	Nsd3	ACAGCAGGCACAGACAGGCAGT#ACCGGGGCTGGGTTGCCGGGTGTCTGCTGG#.((((((((((.....(((((.#.)))))..)))))))))).	38	68	0.84615	-25	1	1.18E-05	0.3	-0	0.42	-10	15	19	10	5UTR
mmu-miR-214-3p	NM_001001735	Nsd3	ACAGCAGGCACAGACAGGCAGT#GCTGGGCTGATCTAGATGTCTGTCTGTT#((((((((((((((((.((((#))))..))).))).).)))))).)))).	3396	3424	0.92308	-21	1	0.0046075	0.5	2.3	1.66	-4	21	28	6	3UTR
mmu-miR-214-3p	NM_001001737	Zfp950	ACAGCAGGCACAGACAGGCAGT#GCTGCTGGTCTGGCTACCTCCCCAGCTGTGTACCCCAGATACCTGCTGG#.((((((((.(((((.((((((#)))))).)))))))..........................))))	548	597	0.92308	-26	1	7.98E-06	0.4	0	0.13	-5	19	49	7	3UTR
mmu-miR-214-3p	NM_001001737	Zfp950	ACAGCAGGCACAGACAGGCAGT#CTGCCCAGCTCATGTGCTGTTTAAGGCTTGTTGG#.(((((((((((((((((((.#)))))).......))).))))).....))))))).	4275	4309	1	-22	1	0.0024751	0.6	-0	0.06	-4	20	34	8	3UTR
mmu-miR-214-3p	NM_001001984	Kdm2a	ACAGCAGGCACAGACAGGCAGT#ACTGCCTGTCTGATGAGAAACTGA#.(((....(((((((((((((#))))))))))))).)).....))).	4271	4295	0.92308	-25	0	0.0001653	0.5	2.3	2.02	-14	17	24	12	CDS
mmu-miR-214-3p	NM_001001984	Kdm2a	ACAGCAGGCACAGACAGGCAGT#CCTGTAAGGCCTGCTGC#.((((((((.(..(((((....#)))))..))))))))).	2476	2493	0.92308	-24	1	0.0001166	0.4	2	1.07	-10	14	17	9	CDS
mmu-miR-214-3p	NM_001003824	Kcnq2	ACAGCAGGCACAGACAGGCAGT#GCCGTTCTGTGTGATTGATATCATGGTGCTGATTGCCTCCATTGCTGTG#((((((((((((((..(((...#)))..))))))).........................))))))).	694	743	0.92308	-22	1	0.0004369	0.5	1.5	2.03	-5	17	49	7	CDS
mmu-miR-214-3p	NM_001003824	Kcnq2	ACAGCAGGCACAGACAGGCAGT#CTGCCTTGTGCTTTCTGTG#((((.(((((((...((((((.#))))))))))))).)))).	511	530	0.84615	-23	0	0.0546885	0.5	-0	-0.5	-11	17	19	13	CDS
mmu-miR-214-3p	NM_001003824	Kcnq2	ACAGCAGGCACAGACAGGCAGT#CTGCGCTGTTGCCTTTGGCTGTC#(((((((((...((((((((.#)))).)))))))))...))))).	2635	2658	1	-22	1	0.0001194	0.5	2.3	2.24	-11	18	23	9	3UTR
mmu-miR-214-3p	NM_001003825	Kcnq2	ACAGCAGGCACAGACAGGCAGT#ATTGCCTCCATTGCTGTGCTGGCTGCTGG#.((((((((((((..(((((((#)))))))......))))))...)))))).	725	754	1	-26	1	0.0001418	0.4	2.2	1.42	-5	19	29	7	CDS

mmu-miR-214-3p	NM_001003825	Kcnq2	ACAGCAGGCACAGACAGGCAGT#CTGCCTTGTGCTTTCTGTG#((((.(((((((...((((((.#))))))))))))).)))).	511	530	0.84615	-23	0	0.0546885	0.5	-0	-0.5	-11	17	19	13	CDS
mmu-miR-214-3p	NM_001003825	Kcnq2	ACAGCAGGCACAGACAGGCAGT#CTGCCTGTCCAGTCTGG#....(((((...(((((((((.#)))))))))..))))).	1889	1906	0.84615	-23	0	2.90E-06	0.4	4.5	3.82	-17	14	17	9	CDS
mmu-miR-214-3p	NM_001003825	Kcnq2	ACAGCAGGCACAGACAGGCAGT#CTGCGCTGTTGCCTTTGGCTGTC#((((((((((...((((((((.#)))).)))))))))...))))).	2620	2643	1	-22	1	0.0001194	0.5	2.3	2.24	-11	18	23	9	3UTR
mmu-miR-214-3p	NM_001003948	Pid1	ACAGCAGGCACAGACAGGCAGT#TGTGTGAGTGTGTGTGTATGTG#((((((.(((((..(((.(((..#))).))..))))).))).))).	2379	2401	1	-19	0	0.3381358	0.5	1.4	1.89	-8	16	22	5	3UTR
mmu-miR-214-3p	NM_001004062	Crtc1	ACAGCAGGCACAGACAGGCAGT#ACTGTTTCTCTGTGCCGTTGCCCACTGCCCATGCTGG#.(((((((((((((.(((((((#))))))).))))))))...............))))).	3239	3276	0.92308	-27	1	5.52E-05	0.4	0.2	0.32	-5	20	37	8	3UTR
mmu-miR-214-3p	NM_001004062	Crtc1	ACAGCAGGCACAGACAGGCAGT#GCTATCTGTCTGTCCATCCATCTACCTGGGGTCAGGCTGATGGCCGAGGCTGTG#(((((.((((((((((((.(((#))).))))))))))...........................)))...))))).	2114	2168	0.84615	-23	0	4.10E-05	0.4	0.2	-0.2	-6	20	54	9	3UTR
mmu-miR-214-3p	NM_001004180	Fam222a	ACAGCAGGCACAGACAGGCAGT#ACTGCCTGATACGGATTCCTGCTGC#.(((((((....(.((((((((#))))))))...).....))))))).	161	186	0.84615	-25	1	0.0001828	0.3	0.4	0.48	-13	16	25	8	5UTR
mmu-miR-214-3p	NM_001004180	Fam222a	ACAGCAGGCACAGACAGGCAGT#GCTCAGCCATGCTGGCCTGTCTGC#.((((((((.(((.((((((((#)))..))).)))))))))).))).	211	235	0.84615	-23	1	5.90E-06	0.3	2.4	1.5	-6	19	24	10	5UTR
mmu-miR-214-3p	NM_001005846	Mcoln2	ACAGCAGGCACAGACAGGCAGT#ACTGCCCAAAGTTCTCCGGTTCTGTGCTTGCG#...((((((((((..((((((#))))))).............))))	1308	1340	0.92308	-26	0	5.13E-05	0.4	4.3	4.01	-7	17	32	11	CDS
mmu-miR-214-3p	NM_001005846	Mcoln2	ACAGCAGGCACAGACAGGCAGT#GCTGTCCTGCCTCTGCTGTCTGAGGA#.(..((((((((((((((((((#)))).)))))..)))).))))))..).	1707	1733	0.92308	-23	0	7.46E-06	0.5	1.5	0.98	-8	19	26	6	CDS
mmu-miR-214-3p	NM_001006668	Kcnq2	ACAGCAGGCACAGACAGGCAGT#GCCGTTCTGTGTGATTGATATCATGGTGCTGATTGCCTCCATTGCTGTG#(((((((((((((((..(((...#)))..)))))))..........................))))))).	694	743	0.92308	-22	1	0.0004369	0.5	1.5	2.03	-5	17	49	7	CDS

mmu-miR-214-3p	NM_001006668	Kcnq2	ACAGCAGGCACAGACAGGCAGT#CTGCCTTGTGCTTTCTGTG#((((.(((((((...((((((.#))))))))))))).)))).	511	530	0.84615	-23	0	0.0546885	0.5	-0	-0.5	-11	17	19	13	CDS
mmu-miR-214-3p	NM_001006668	Kcnq2	ACAGCAGGCACAGACAGGCAGT#CTGCGCTGTTGCCTTTGGCTGTC#(((((((((...((((((((.#)))).)))))))))...))))).	2599	2622	1	-22	1	0.0001194	0.5	2.3	2.24	-11	18	23	9	3UTR
mmu-miR-214-3p	NM_001006669	Kcnq2	ACAGCAGGCACAGACAGGCAGT#GCCGTTCTGTGTGATTGATATCATGGTGCTGATTGCCTCCATTGCTGTG#((((((((((((((..(((...#)))..)))))))..........................))))))).	694	743	0.92308	-22	1	0.0004369	0.5	1.5	2.03	-5	17	49	7	CDS
mmu-miR-214-3p	NM_001006669	Kcnq2	ACAGCAGGCACAGACAGGCAGT#CTGCCTTGTGCTTTCTGTG#((((.(((((((...((((((.#))))))))))))).)))).	511	530	0.84615	-23	0	0.0546885	0.5	-0	-0.5	-11	17	19	13	CDS
mmu-miR-214-3p	NM_001006669	Kcnq2	ACAGCAGGCACAGACAGGCAGT#CTGCGCTGTTGCCTTTGGCTGTC#(((((((((...((((((((.#)))).)))))))))...))))).	2527	2550	1	-22	1	0.0001194	0.5	2.3	2.24	-11	18	23	9	3UTR
mmu-miR-214-3p	NM_001006674	Kcnq2	ACAGCAGGCACAGACAGGCAGT#GCCGTTCTGTGTGATTGATATCATGGTGCTGATTGCCTCCATTGCTGTG#((((((((((((((..(((...#)))..)))))))..........................))))))).	694	743	0.84615	-22	1	0.0004369	0.5	3.8	4.05	-5	17	49	7	CDS
mmu-miR-214-3p	NM_001006674	Kcnq2	ACAGCAGGCACAGACAGGCAGT#CTGCCTTGTGCTTTCTGTG#((((.(((((((...((((((.#))))))))))))).)))).	511	530	0.84615	-23	0	0.0546885	0.5	-0	-0.5	-11	17	19	13	CDS
mmu-miR-214-3p	NM_001006674	Kcnq2	ACAGCAGGCACAGACAGGCAGT#CTGCGCTGTTGCCTTTGGCTGTC#(((((((((...((((((((.#)))).)))))))))...))))).	2082	2105	1	-22	1	0.0001194	0.5	2.3	2.24	-11	18	23	9	3UTR
mmu-miR-214-3p	NM_001007570	Slc25a42	ACAGCAGGCACAGACAGGCAGT#ACTGCCTTCTGTGGCTGTC#...((((.((((((.(((((((#))))))))))))).)))).	1786	1805	0.84615	-28	0	7.24E-06	0.5	-0	-0.1	-10	17	19	13	3UTR
mmu-miR-214-3p	NM_001007570	Slc25a42	ACAGCAGGCACAGACAGGCAGT#CTGCTGTCTGGAGCCCTGGCTGG#.((((.(((.((((((.((((.#))))))))))..)))...)))).	194	217	0.92308	-26	0	3.57E-06	0.4	0.4	-0.2	-7	17	23	10	CDS
mmu-miR-214-3p	NM_001008421	Nol10	ACAGCAGGCACAGACAGGCAGT#GCAGGCTGACTGGCTTGCTT#..(((((((.((((.(((.(.((#)).).))).)))))))))).	2894	2914	0.84615	-20	1	6.96E-05	0.6	-0	0.06	-7	16	20	10	3UTR
mmu-miR-214-3p	NM_001008421	Nol10	ACAGCAGGCACAGACAGGCAGT#GCCCCCCTTAAATCTTGTCTTCTCCCCTGCTGA#.(((((((...(((((((....#.))))))).....))))))).	2320	2353	0.92308	-20	1	0.0196631	0.5	-0	-0.2	-9	14	21	7	3UTR

mmu-miR-214-3p	NM_001013360	Npcd	ACAGCAGGCACAGACAGGCAGT#ACTGCCTGTCCTTGAGCCCTGCTT#..(((((((.((((((((((((#))))))))))..)).))).)))).	2436	2460	0.92308	-28	1	0.0013231	0.4	-0	-0.1	-6	19	24	10	3UTR
mmu-miR-214-3p	NM_001013362	Npcd	ACAGCAGGCACAGACAGGCAGT#ACTGCCTGTCCTTGAGCCCTGCTT#..(((((((.((((((((((((#))))))))))..)).))).)))).	2727	2751	0.84615	-28	1	0.0013231	0.4	0.1	0.37	-6	19	24	10	3UTR
mmu-miR-214-3p	NM_001013370	Sesn1	ACAGCAGGCACAGACAGGCAGT#ACTGCCTGTCTAGCAGTGAGCACTCGGCAGCTTTGGTGTTAGAGCTGG#.((((.((((((((((((((((#)))))))))))........................)))))...)))).	1690	1738	0.92308	-27	0	0.0007138	0.4	1.1	1.66	-6	20	48	11	3UTR
mmu-miR-214-3p	NM_001013370	Sesn1	ACAGCAGGCACAGACAGGCAGT#CTGCCCCATGCCCTGCTGA#.(((((((..((....(((((.#)))))...)).))))))).	2356	2375	0.92308	-21	1	0.000326	0.4	1.2	0.98	-9	14	19	7	3UTR
mmu-miR-214-3p	NM_001013390	Scn4b	ACAGCAGGCACAGACAGGCAGT#GCTGCCTGCCTAGTCTGTCTTTGCTCTCTGGGCTGCAGCTGCTGA#.(((((((((((((((((((((#))))))))).................))))...)))..)))))).	1700	1745	0.92308	-26	1	0.0003349	0.4	-1	-0.3	-4	21	45	8	3UTR
mmu-miR-214-3p	NM_001013390	Scn4b	ACAGCAGGCACAGACAGGCAGT#ATTGCCCGTCTCCTGCTT#..((((((...((((.((((((#)))))).)))))))))).	2869	2887	0.92308	-22	1	0.1627948	0.6	0	-0.1	-10	16	18	10	3UTR
mmu-miR-214-3p	NM_001013757	Vwde	ACAGCAGGCACAGACAGGCAGT#ACTGCTTGTCTGATACCATACAACTTGATGGTCTCTTCCTGGCAGCTGTTAATCCTTTCCTGCTGA#.(((((((((((((((((((((#))))))))))))...............))...........................))))))).	3019	3085	0.84615	-25	1	0.0166042	0.6	2	2.41	-4	21	66	12	3UTR
mmu-miR-214-3p	NM_001013757	Vwde	ACAGCAGGCACAGACAGGCAGT#GCTGCCTCTTTCAAATCCCTGTGTCTGTA#...((((((((((..(((((((#))))))).........)))))))))).	490	519	0.84615	-24	0	0.0378905	0.6	0.1	-0.1	-8	17	29	10	CDS
mmu-miR-214-3p	NM_001015046	Rap1gap2	ACAGCAGGCACAGACAGGCAGT#ACTGTCTGTCTTGGGTCGCAGTGCAGTGCCTTCCTGG#.(((.(((((((((((((((((#)))))))))))).............))))))..))).	2786	2823	0.89744	-27	0	1.20E-07	0.5	-0	-0.1	-8	20	37	11	3UTR
mmu-miR-214-3p	NM_001015046	Rap1gap2	ACAGCAGGCACAGACAGGCAGT#CTGACCGTTTAGGCCTGCTGTC#(((((((((..((((.(((((.#))).))))))..))))))))).	2475	2497	0.92308	-26	1	0.0017136	0.4	0.4	0.48	-8	18	22	9	3UTR
mmu-miR-214-3p	NM_001015046	Rap1gap2	ACAGCAGGCACAGACAGGCAGT#ACTGCTAGGGAAGATGTGCCTGCC#...(((((((((..(.((((((#))))))..).....))))))))).	1392	1416	1	-25	0	0.0006894	0.4	-0	-0.1	-9	16	24	9	CDS

mmu-miR-214-3p	NM_001015046	Rap1gap2	ACAGCAGGCACAGACAGGCAGT#CTGCCTGCAGGCATTACCTCTGGGCTGGATGACTGTGTGCTGA#.(((((.((((((((((((((.#)))))))...........))))..........))).))))).	4291	4334	0.92308	-23	0	0.0076445	0.4	2.2	2.92	-5	19	43	7	3UTR
mmu-miR-214-3p	NM_001015876	Tyw1	ACAGCAGGCACAGACAGGCAGT#GCTGGGGCATTCCCTCATGACAGTGCTCCTGCCTCAGCCTGCTGA#.((((((((..((.((((.(((#.))))))).))..)))))))).	2699	2744	1	-26	1	1.20E-06	0.4	0.1	0.11	-9	17	22	8	3UTR
mmu-miR-214-3p	NM_001015876	Tyw1	ACAGCAGGCACAGACAGGCAGT#GCTGTCACCTCTCTGGACCTGCCTGTG#(((((((..((((.(((((((#))))...))).))))..))))).))).	426	453	0.92308	-24	1	0.0016521	0.5	2.1	2.14	-7	19	27	5	CDS
mmu-miR-214-3p	NM_001015876	Tyw1	ACAGCAGGCACAGACAGGCAGT#CCGTCTGTGCCTTCCTGG#.(((.((((((((((.(((....#))))))))))))..))).	517	535	1	-23	0	1.94E-05	0.5	2.6	2.62	-8	15	18	12	CDS
mmu-miR-214-3p	NM_001017525	Btbd11	ACAGCAGGCACAGACAGGCAGT#CTGTCTGTCTGTACTGGGCCAAGGAGCTGC#.((((.((((((((((((((.#))))))))))))).....))).....))	2082	2112	0.92308	-26	0	3.40E-05	0.4	0.7	0.41	-6	19	30	12	3UTR
mmu-miR-214-3p	NM_001018063	Rtl8b	ACAGCAGGCACAGACAGGCAGT#CTGCTGCTGTCCCTGCTGG#.(((((((.((((.((.((((.#)))))))))).)))))))).	708	727	0.84615	-26	1	7.86E-06	0.3	-0	0.01	-6	17	19	10	3UTR
mmu-miR-214-3p	NM_001018063	Rtl8b	ACAGCAGGCACAGACAGGCAGT#CTGCCCATAAGGCCTGTGCTGACGCTGC#.((((.(((((((.(.(((((.#))))).....)..)))))))...)))).	913	941	0.84615	-22	0	1.58E-05	0.4	1.9	1.08	-7	17	28	7	3UTR
mmu-miR-214-3p	NM_001018087	Ldoc1	ACAGCAGGCACAGACAGGCAGT#CCTGATGTGCCTGCCGG#.(.(((((((((..((((....#)))).))))))))).).	1113	1130	0.92308	-24	0	0.0001757	0.5	0.6	0.27	-7	14	17	9	3UTR
mmu-miR-214-3p	NM_001018087	Ldoc1	ACAGCAGGCACAGACAGGCAGT#GCTCAGCCTCTCTGTGCAGCTC#..(((..((((((((.(((((((#)))..)))).))))))).))).	17	39	0.84615	-22	0	4.21E-05	0.4	0.8	0.28	-9	17	22	7	5UTR
mmu-miR-214-3p	NM_001024205	Nufip2	ACAGCAGGCACAGACAGGCAGT#CTGTGTGTCTGTGTCTGTGTGTG#((((((((((((((((.((((.#)))).))))))))))))).))).	3517	3540	1	-31	1	0.1975398	0.4	0.5	1.01	-4	20	23	13	3UTR
mmu-miR-214-3p	NM_001024539	Shc2	ACAGCAGGCACAGACAGGCAGT#CTGCCTGGACCTTCAACTGTAATTAAACACAGGGTTGAGCGTGAGCCTGCTGTG#(((((((((((((.(((((((.#)))))))).........))))........................))))))))).	2349	2403	0.92308	-27	1	0.0001835	0.5	3.4	2.59	-5	20	54	9	3UTR

mmu-miR-214-3p	NM_001024539	Shc2	ACAGCAGGCACAGACAGGCAGT#GCTGCCTATCGTGGCTGCTGA#.((((((.(((.((.(((((((#))))))).))))).)))))).	1726	1747	0.84615	-30	1	9.41E-06	0.4	0.6	0.04	-10	18	21	7	CDS
mmu-miR-214-3p	NM_001024617	Inpp4b	ACAGCAGGCACAGACAGGCAGT#ATTGTCTTCAGTCAGGCGCTTGCTGG#.((((((((.(.((((((((((#))))))))...)))..).))))))))).	2308	2334	1	-23	1	4.19E-05	0.6	3.5	3.13	-8	19	26	8	CDS
mmu-miR-214-3p	NM_001024617	Inpp4b	ACAGCAGGCACAGACAGGCAGT#GCTGATCGAAATGTGTCTGCTT#..((((((((((....((((((#)))).))....)))))))))).	3700	3722	0.92308	-22	1	0.00675	0.6	0.3	0.77	-8	16	22	10	3UTR
mmu-miR-214-3p	NM_001024910	Septin10	ACAGCAGGCACAGACAGGCAGT#GCTCCTTGTCTATGTGTGGCCATGTTGG#.((((((((((((((((.(((#))).))))))))....))).)).))))).	126	154	0.84615	-21	1	0.002807	0.5	1.8	0.94	-4	20	28	7	CDS
mmu-miR-214-3p	NM_001024910	Septin10	ACAGCAGGCACAGACAGGCAGT#GCAGATGTTTGTGCAGCG#...((..((((((((((.((...#))...))))))))).)).	1111	1129	0.84615	-18	0	0.0021023	0.5	3	3.24	-10	13	18	9	CDS
mmu-miR-214-3p	NM_001024910	Septin10	ACAGCAGGCACAGACAGGCAGT#CTGTGTTTGCG#...((((((((((.........#)))))))))).	40	51	1	-16	0	1.84E-05	0.4	-0	0.1	-8	10	11	10	5UTR
mmu-miR-214-3p	NM_001024911	Septin10	ACAGCAGGCACAGACAGGCAGT#TGCCTTATTGTGTATTGCTGTG#(((((((.((((((..(((((..#)))))..))))))..))))))).	1858	1880	0.92308	-23	0	7.03E-05	0.7	3	3.08	-7	17	22	6	3UTR
mmu-miR-214-3p	NM_001024911	Septin10	ACAGCAGGCACAGACAGGCAGT#GCTCCTTGTCTATGTGTGGCCATGTTGG#.((((((((((((((((.(((#))).))))))))....))).)).))))).	126	154	0.92308	-21	1	0.002807	0.5	0.2	0.36	-4	20	28	7	CDS
mmu-miR-214-3p	NM_001024911	Septin10	ACAGCAGGCACAGACAGGCAGT#CTTGCTGTGGATCTTGGTCACTTTCCTGCTGC#.(((((((((((.((((....#)))))))))).............)))	1595	1627	0.92308	-20	1	0.0015781	0.6	3.3	3.68	-5	16	32	9	3UTR
mmu-miR-214-3p	NM_001024945	Qsox1	ACAGCAGGCACAGACAGGCAGT#GCTGTCCTGGACTGTGCTGAGGAGACCAACAGTGCTGTC#((((((((((((.((((((((#)))).)))))..)))))))).............)))))).	397	436	0.84615	-27	1	0.0001913	0.4	0.1	0.09	-5	21	39	7	CDS
mmu-miR-214-3p	NM_001024945	Qsox1	ACAGCAGGCACAGACAGGCAGT#GCTGCTTCGGAATGGCTCTGTCTCCCGAGTGCCTGTGCTGG#.((((((((((((((((((((#)))))............))))))).....))))))..)))).	825	866	0.92308	-26	1	9.80E-06	0.5	0.2	-0.1	-4	21	41	6	CDS
mmu-miR-214-3p	NM_001024952	Rc3h1	ACAGCAGGCACAGACAGGCAGT#GCTGTGGCCATACTGTCTGCAAAATGTGCCTGA#....((((((((..((((((((#.)))))))).....)))))))	533	566	1	-25	0	0.0008189	0.5	4.8	4.67	-12	16	23	8	CDS

mmu-miR-214-3p	NM_001024952	Rc3h1	ACAGCAGGCACAGACAGGCAGT#TGTGTGTGTGTGCGTGTTAGTG#((((((.(((((.(((.(((..#))).))).))))).)))).)).	398	420	0.92308	-24	0	0.0189697	0.6	1.2	4.08	-7	17	22	5	5UTR
mmu-miR-214-3p	NM_001024954	Pbx4	ACAGCAGGCACAGACAGGCAGT#GCTGCATCTAATTAAGTTTCTGGCACCTGGCTGAGTGCCATAGGTGACTGGCCCTGTGTCCTGCTGA#.((((((((((((...(.((((#.)))).).))))).))))))).	1207	1274	0.84615	-27	1	0.00265	0.4	1.1	0.3	-6	17	22	7	CDS
mmu-miR-214-3p	NM_001025192	Cxadr	ACAGCAGGCACAGACAGGCAGT#GTCGGGGTAGGGGGAGGATCGATGAGGACAGTGTCTGTGCCTCGCTC#..((((((((((((((......#.))))))))))).))).	2196	2243	1	-24	1	3.30E-07	0.4	0.1	-0.3	-5	14	17	11	3UTR
mmu-miR-214-3p	NM_001025192	Cxadr	ACAGCAGGCACAGACAGGCAGT#CTGGTCCTGAATTCCAGACTTGAGCAATGTGCTTGCTGG#.((((((((((..(((((((.#)))..))))..................)))))))))).	2617	2656	0.92308	-23	1	4.26E-05	0.4	0.7	0.56	-6	18	39	11	3UTR
mmu-miR-214-3p	NM_001025379	Sema3g	ACAGCAGGCACAGACAGGCAGT#GCTGGTGTGCTCTGTGCCTGG#....((((((((((((.(((((#)))).).)).)))))))))).	910	931	0.84615	-25	0	2.76E-05	0.4	2.5	3.24	-10	17	21	10	CDS
mmu-miR-214-3p	NM_001025379	Sema3g	ACAGCAGGCACAGACAGGCAGT#AGTGCCTGTGCTGAGTGCTGCCTGG#....((((((((((((((((.(#).))))))).))).....)))))).	1616	1641	0.92308	-24	0	0.0002347	0.4	3.8	2.74	-10	17	25	7	CDS
mmu-miR-214-3p	NM_001025581	Kcnc2	ACAGCAGGCACAGACAGGCAGT#GCCATGCCTGTGCCTGTC#...((((((((((.(((((...#))).)).)))))))))).	1471	1489	0.92308	-26	0	0.0003931	0.5	4.1	4.7	-7	15	18	10	CDS
mmu-miR-214-3p	NM_001025581	Kcnc2	ACAGCAGGCACAGACAGGCAGT#GCTGTCTGGTGCTGCTGTT#((((((.((((...((((((((#)))))))))))))))))).	4994	5013	1	-25	0	4.48E-05	0.6	2.1	1.97	-11	18	19	18	3UTR
mmu-miR-214-3p	NM_001025600	Cadm1	ACAGCAGGCACAGACAGGCAGT#GCTGCCCATGTCTTAAATACGTAGAGCAGCGAAGACTAGAAACGTAGACTGTGACCCAGACGCTGTCGTCTGCTGTG#((((((((...((((((((((#.)))))..........))))))))))))).	1977	2054	0.84615	-23	1	0.0002798	0.5	1.5	1.26	-11	19	31	14	3UTR
mmu-miR-214-3p	NM_001029850	Magi1	ACAGCAGGCACAGACAGGCAGT#ACCGCCATGCTGTGCCTTGCA#...((((((((((((((.((#)).))).))))))))).)).	1007	1028	0.92308	-24	0	7.70E-05	0.4	3.4	2.48	-7	17	21	10	CDS

mmu-miR-214-3p	NM_001029850	Magi1	ACAGCAGGCACAGACAGGCAGT#GTCTTGTGCCTGGTGTA#(((.(((((((((((.......#)))).))))))).))).	5178	5195	1	-24	0	0.1128847	0.8	3.9	3.86	-7	14	17	7	3UTR
mmu-miR-214-3p	NM_001029850	Magi1	ACAGCAGGCACAGACAGGCAGT#ACTGCCTAGCTGAGCCAAAGTGTTTGCTTGTA#((((((((.(((..(((((((#)))))))..))).))).......	6271	6303	0.84615	-23	1	0.0073733	0.5	5.5	3.95	-7	19	32	7	3UTR
mmu-miR-214-3p	NM_001029877	Nova2	ACAGCAGGCACAGACAGGCAGT#CTGCCTTTTGTGTGCATGCCTGTG#((((((.(((((.(.((((((.#))))))..).))))).))).))).	3017	3041	0.84615	-23	0	0.0016564	0.5	0.5	0.74	-8	18	24	6	3UTR
mmu-miR-214-3p	NM_001029877	Nova2	ACAGCAGGCACAGACAGGCAGT#CTCCCTCCCTGTGCTTGACTGG#.(((((((((((..(((.((.#)).)))..))))))))).))).	1898	1920	0.92308	-23	1	0.0005611	0.4	1.1	1.25	-5	17	22	9	3UTR
mmu-miR-214-3p	NM_001029877	Nova2	ACAGCAGGCACAGACAGGCAGT#CTGCCTGCTGCCACTCTGCCGCA#...((.(((((((.((((((((.#))))))))))......)))))).	7318	7341	0.84615	-23	0	0.0001621	0.3	-0	-0	-9	16	23	10	3UTR
mmu-miR-214-3p	NM_001029878	Lonrf2	ACAGCAGGCACAGACAGGCAGT#GCAGAGCCGTGTTTGTGTTTCGTGTCCTGCTGTA#((((((((((((((((((.((#))...))).))))))))........))))))).	3041	3075	0.92308	-26	1	0.0121562	0.5	0	0	-4	21	34	8	3UTR
mmu-miR-214-3p	NM_001029912	Zswim5	ACAGCAGGCACAGACAGGCAGT#GCTGCCTGTTTCTCACTTTGACTGAAGCCTGCC#...((((((.((((((((((((#)))))))))))...........)	1895	1928	1	-27	0	0.0094095	0.5	2.9	3.37	-8	18	33	9	CDS
mmu-miR-214-3p	NM_001029912	Zswim5	ACAGCAGGCACAGACAGGCAGT#ACATGTTTGTCTCTGAAGGTATGCCTGCTGA#.(((((((((.(((((((((((#)).))))))))).........))))	4646	4677	1	-25	1	0.0008895	0.6	0.2	0.1	-7	20	31	9	3UTR
mmu-miR-214-3p	NM_001033163	Ephx3	ACAGCAGGCACAGACAGGCAGT#AGTGTGTGTGTGTGTGTGTGTGTG#((((((.(((((.(((.(((.(#).))).))).))))).))).))).	2210	2234	1	-22	0	0.7081606	0.5	1.7	0.47	-7	18	24	5	3UTR
mmu-miR-214-3p	NM_001033163	Ephx3	ACAGCAGGCACAGACAGGCAGT#TGTGTGTGTGTGTGTGTGTGTG#((((((.(((((.(((.(((..#))).))).))))).))).))).	1880	1902	0.84615	-22	0	0.2209187	0.5	2.7	1.53	-7	17	22	5	3UTR
mmu-miR-214-3p	NM_001033167	Slc22a23	ACAGCAGGCACAGACAGGCAGT#ACTGGTTGTGTTGGCCTGCA#...((((((.(((((((.((((#)))).)))).))))))))).	4481	4501	1	-24	0	0.0003211	0.5	4	2.86	-8	17	20	9	3UTR
mmu-miR-214-3p	NM_001033219	Slc45a4	ACAGCAGGCACAGACAGGCAGT#GCATGCCTGTTTGTTGCTGTC#((((((...(((((((((((((((#)).))))))))))))))))).	5464	5485	1	-25	0	0.0001058	0.5	0.3	1.1	-9	19	21	17	3UTR

mmu-miR-214-3p	NM_001033219	Slc45a4	ACAGCAGGCACAGACAGGCAGT#GCGCCTGCTGTGGCTGTC#...((((.(((((.(((((.((#)))))))))))).)))).	2342	2360	1	-25	0	2.36E-05	0.4	0.2	-0.5	-10	16	18	12	CDS
mmu-miR-214-3p	NM_001033269	Eif4e1b	ACAGCAGGCACAGACAGGCAGT#ACTGCCACAGCTCATCACTGTGCCTGCA#...((((((((((...((((((#))))))...........)))))))))).	1075	1103	0.92308	-27	0	0.0130548	0.5	0	-0.2	-8	16	28	10	3UTR
mmu-miR-214-3p	NM_001033269	Eif4e1b	ACAGCAGGCACAGACAGGCAGT#ACTGCGCCACTTCGAACTGGACCGTCTGTGGCTGGAGACGTTGCTGTG#(((((((.((((((((.((((((#.))))..))))))))).))........))))).	547	595	1	-23	1	0.0064849	0.5	1	3.37	-6	20	34	9	CDS
mmu-miR-214-3p	NM_001033272	Spata13	ACAGCAGGCACAGACAGGCAGT#GCCAGGCCTCCTGTGCCTGCA#...((((((((((..((((.((#))...)))).)))))))))).	2227	2248	0.92308	-26	0	1.09E-05	0.4	2.8	2.15	-8	16	21	10	CDS
mmu-miR-214-3p	NM_001033272	Spata13	ACAGCAGGCACAGACAGGCAGT#ACTGTCTGACTATGGCCAGCTGG#.((((.(((.((..((((((((#))))))))....))))).)))).	1971	1994	0.92308	-24	0	7.29E-06	0.4	2.4	1.79	-9	17	23	8	CDS
mmu-miR-214-3p	NM_001033286	Slc30a10	ACAGCAGGCACAGACAGGCAGT#TTGTCTGTCTTGTGGTGCTGG#.(((((..((((((((((((.#))))))))))).))).))))).	1926	1947	0.84615	-26	0	0.0019929	0.5	-0	-0	-8	18	21	10	3UTR
mmu-miR-214-3p	NM_001033286	Slc30a10	ACAGCAGGCACAGACAGGCAGT#GCTGTTTGAGATGTTAGTGGAACAAGCCTGCTGA#.(((((((((((..((((((((#))))))))...)))...........)))))))).	1731	1765	0.92308	-24	1	0.0024564	0.5	-0	0.12	-6	19	34	8	3UTR
mmu-miR-214-3p	NM_001033286	Slc30a10	ACAGCAGGCACAGACAGGCAGT#GCTGCCTGGTGTTTTTGTCTGGGGTTTCCTGCA#...(((((..((((((((((((#))))))).........)))))...	3909	3942	0.92308	-23	0	0.0003236	0.5	0.1	-0.1	-9	17	33	7	3UTR
mmu-miR-214-3p	NM_001033322	Gucy1a2	ACAGCAGGCACAGACAGGCAGT#ACTCAGGCTCTGTGCTGGCTGG#.((((.(((((((((..(.(((#))))..).)))))))).)))).	2300	2322	0.92308	-22	0	2.27E-05	0.5	3.8	3.87	-6	17	22	8	CDS
mmu-miR-214-3p	NM_001033336	Abcc4	ACAGCAGGCACAGACAGGCAGT#ATGGCCTGTCGTTTTATTTTAAAAGTTCCTGCTGTT#((((((((.((.((((((((.((#)).)))))))).............)).)))))))).	4272	4308	1	-23	1	0.0409318	0.6	1.4	2.34	-8	19	36	8	3UTR
mmu-miR-214-3p	NM_001033336	Abcc4	ACAGCAGGCACAGACAGGCAGT#TTGCCAGTTCGTCTCCCTGCTGTC#((((((((.((.((((.(((((.#))))).))).))...)))))))).	4497	4521	1	-23	1	0.0112708	0.5	4	2.47	-9	18	24	8	3UTR

mmu-miR-214-3p	NM_001033347	D430041D05Rik	ACAGCAGGCACAGACAGGCAGT#GCTGGTGTATTTGCCTGCTGTG#((((((((((...(((..((((#)))).)))...)))))))))).	1541	1563	1	-25	1	7.00E-05	0.5	0.7	-0	-11	17	22	10	CDS
mmu-miR-214-3p	NM_001033347	D430041D05Rik	ACAGCAGGCACAGACAGGCAGT#GCTGCTCTGTCTTCAGTGACCTCTGC#.((((.((((((((((((((((#))))).))))))...))).))))))).	4783	4809	1	-25	0	0.0044315	0.5	0.8	0.4	-8	20	26	6	3UTR
mmu-miR-214-3p	NM_001033375	Washc4	ACAGCAGGCACAGACAGGCAGT#ACTGTTACTGCCTGTGCACAGCGTGGCCAGCTGG#.((((.(((((..(((((((((#.)))))))))).......)	4082	4116	0.84615	-26	0	5.89E-06	0.4	0.1	0.01	-10	18	29	9	3UTR
mmu-miR-214-3p	NM_001033375	Washc4	ACAGCAGGCACAGACAGGCAGT#GCTGCTCGGGGCTGTGTCTGTGGG#.(.((((((((((.(.((((((#))))))..)..)))))))))).).	64	88	0.84615	-24	0	1.60E-05	0.3	0.8	1.66	-6	18	24	10	5UTR
mmu-miR-214-3p	NM_001033375	Washc4	ACAGCAGGCACAGACAGGCAGT#CTGTACTCTGTGTGTGTTTGTA#...(((((((((.((((((((.#))))...)))).))))))))).	3903	3925	0.84615	-22	0	0.2216543	0.6	0.8	0.48	-8	17	22	9	3UTR
mmu-miR-214-3p	NM_001033405	Treml2	ACAGCAGGCACAGACAGGCAGT#GCGAGACCCTGTCTGTGCAGTGCTC#..((((.(((((((((((....#.)))))))))))..)))).	604	629	1	-26	0	0.0026474	0.4	1.7	0.6	-7	15	19	11	CDS
mmu-miR-214-3p	NM_001033405	Treml2	ACAGCAGGCACAGACAGGCAGT#GCTGCTGCTGCTGTGGCTACAGGGCTGTG#(((((((.(((((.((((((((#)))))..)))))))).)).....))))).	524	553	0.92308	-25	1	8.69E-05	0.4	1.9	0.35	-7	20	29	8	CDS
mmu-miR-214-3p	NM_001033405	Treml2	ACAGCAGGCACAGACAGGCAGT#GCTGACGAAGGCCATGTGTCTTCTGATATTCCCTGACTGTGACTGCTGG#.(((((((.(((((.((((.(((#.))).)))).))))).)))))).	1750	1799	0.92308	-25	1	0.0089237	0.5	0.4	-0.2	-7	18	23	6	3UTR
mmu-miR-214-3p	NM_001033410	Rnf224	ACAGCAGGCACAGACAGGCAGT#CTGCTGTCTATGCCTCTTGTA#((((.((((((.((((((.((((.#))))))))).))))).)))).	913	934	0.92308	-23	0	0.0134334	0.5	0.8	-0.4	-11	18	21	9	3UTR
mmu-miR-214-3p	NM_001033410	Rnf224	ACAGCAGGCACAGACAGGCAGT#CTGCCGTCTTGCCCGTGTA#(((.(.((((.((((.(((((.#))))))))))))).)))).	31	50	0.92308	-21	0	0.0003946	0.4	-1	-0.6	-12	17	19	13	5UTR
mmu-miR-214-3p	NM_001033444	Capn13	ACAGCAGGCACAGACAGGCAGT#ATTGCCTGAGCAGGGGTCAGCTGTTTATAGATGATACATTCCCTGCTGC#.((((((((.((((.((((((((#))))))))............))))................)))))	227	276	0.84615	-20	1	0.0041299	0.5	-0	-0.2	-6	19	49	8	CDS
mmu-miR-214-3p	NM_001033444	Capn13	ACAGCAGGCACAGACAGGCAGT#ACTCTTGTCTGCTTCCTGATACGGCTGG#.(((((((..((((((((.(((#)))))))))))...)))......)))).	2035	2063	0.84615	-19	1	0.0002902	0.5	3.3	2.47	-6	18	28	11	CDS

mmu-miR-214-3p	NM_001033475	Tmed8	ACAGCAGGCACAGACAGGCAGT#ACTGCCATATTCCTGCCTGCCTGCTGG#.(((((((((((...((((((#))))))......)))..))))))))).	1488	1515	0.92308	-27	1	0.0002423	0.4	0.2	0.16	-6	18	27	9	3UTR
mmu-miR-214-3p	NM_001033475	Tmed8	ACAGCAGGCACAGACAGGCAGT#GCTGCAGTTATTGCTTGCTGA#.(((((((((..(((..(((((#))))).)))..))))))))).	3224	3245	1	-25	1	0.0001754	0.5	0.1	0.05	-10	17	21	9	3UTR
mmu-miR-214-3p	NM_001033534	Layn	ACAGCAGGCACAGACAGGCAGT#TGCCTCAGGCTGGCCTGCA#...((((((.(((..(((((..#)))))....))))))))).	1687	1706	1	-24	0	8.52E-05	0.5	-0	0.18	-9	14	19	9	3UTR
mmu-miR-214-3p	NM_001033536	Rfx7	ACAGCAGGCACAGACAGGCAGT#ACTGGCATATGTGTGTGTTTGCTGTT#((((((((((((.((((.((((#)))).)...))).))))))))))))).	6650	6676	0.92308	-27	1	0.0268614	0.7	2.8	1.66	-5	20	26	12	3UTR
mmu-miR-214-3p	NM_001033536	Rfx7	ACAGCAGGCACAGACAGGCAGT#TTGTCTGTTGTACCTGCTGA#.(((((((.(((.((((((((.#))))))))))).))))))).	7743	7763	1	-26	1	0.0010003	0.5	5.5	3.32	-6	18	20	11	3UTR
mmu-miR-214-3p	NM_001033536	Rfx7	ACAGCAGGCACAGACAGGCAGT#ACTGTCTGCTACATTGCAAGAGTCTCAGGTGCCTCCTGTA#((((.((((((((.((((((((#))))))))))).................)))))).)))).	2559	2599	0.92308	-24	0	0.0007388	0.5	1.8	2.06	-9	20	40	10	CDS
mmu-miR-214-3p	NM_001033536	Rfx7	ACAGCAGGCACAGACAGGCAGT#GGTGCCGGAGTGTGAGCCCTGCTGTC#(((((((((((..(.((((.(#).)))).)..))))...)))))))).	4064	4090	0.92308	-24	1	0.0004453	0.4	4.4	2.55	-6	18	26	8	CDS
mmu-miR-214-3p	NM_001033769	B020031M17Rik	ACAGCAGGCACAGACAGGCAGT#ACCTTCTGTCAGCTCCTGCTGTT#((((((((..(.((((((....#.)))))).)..)))))))).	798	821	0.92308	-21	1	3.48E-05	0.5	-0	0.02	-10	15	20	8	3UTR
mmu-miR-214-3p	NM_001033790	Pramel17	ACAGCAGGCACAGACAGGCAGT#GCTGCAAGCTGTGCTGGATGGCATAGATATACTGCTGA#.((((((((((((.(..(((((#)))))..)))))))).................))))))).	372	410	0.84615	-24	1	8.44E-05	0.5	-0	-0.2	-5	18	38	7	CDS
mmu-miR-214-3p	NM_001033790	Pramel17	ACAGCAGGCACAGACAGGCAGT#TTGCTACTGTCTGCTGCCCTAATTGTTTGAGACGCTGTG#((((((.(((((((((((((((.#))))..))))))).)))).............))))).	1490	1529	0.92308	-24	0	0.0091561	0.5	0.1	0.03	-6	20	39	7	CDS
mmu-miR-214-3p	NM_001033790	Pramel17	ACAGCAGGCACAGACAGGCAGT#GCTGCCGACTGTTCCTTCTGA#.(((.(((.((((...((((((#))))))..)))).))).))).	105	126	1	-22	0	0.0031264	0.5	-0	-0	-11	16	21	6	5UTR

mmu-miR-214-3p	NM_001033908	Med22	ACAGCAGGCACAGACAGGCAGT#CCTGTCCACTGCCTGCTGC#.(((((((((..((((((....#))))))...))))))))).	1209	1228	1	-27	1	0.004589	0.5	-0	-0.6	-10	15	19	9	3UTR
mmu-miR-214-3p	NM_001033908	Med22	ACAGCAGGCACAGACAGGCAGT#CTCGCCGTAAGGGCCTGCTGC#.((((((((....((.(((((.#)).)))))....)))))))).	102	123	0.84615	-23	1	4.97E-05	0.3	-0	-0.4	-13	15	21	8	5UTR
mmu-miR-214-3p	NM_001033966	Ak2	ACAGCAGGCACAGACAGGCAGT#CTGCCTGTCTCTGCCGCCTGA#.(((..((((.(((((((((((.#))))))))))).)))))..))).	1234	1255	1	-30	0	0.0006202	0.5	-0	-0.4	-13	17	21	10	3UTR
mmu-miR-214-3p	NM_001034030	Limk2	ACAGCAGGCACAGACAGGCAGT#CTGCACGCTGTCCTTGCTGTG#((((((((.((((.(..((((.#))))..))))).)))))))).	1506	1527	0.82051	-23	1	1.50E-05	0.4	0.3	0.11	-6	17	21	8	3UTR
mmu-miR-214-3p	NM_001034030	Limk2	ACAGCAGGCACAGACAGGCAGT#TGATCTGGGCCTGCTGG#.((((((((.((((((......#)).)))).)))))))).	1800	1817	0.92308	-23	1	2.80E-05	0.5	0.2	0.03	-5	14	17	8	3UTR
mmu-miR-214-3p	NM_001034085	Ppp2r1b	ACAGCAGGCACAGACAGGCAGT#TGCACTGTCTGAAGCCTGTGGTA#((.((((((.((((((((((..#))).)))))))..)))))).)).	1614	1637	1	-26	0	4.26E-05	0.5	3.5	3.95	-7	18	23	7	CDS
mmu-miR-214-3p	NM_001034085	Ppp2r1b	ACAGCAGGCACAGACAGGCAGT#GCTGCTGGCTGGCCAGCTGG#.((((.(((.(((.(.((((((#)))))).))))))).))))).	1380	1400	0.84615	-25	0	8.14E-07	0.5	2.4	4.37	-8	17	20	7	CDS
mmu-miR-214-3p	NM_001034085	Ppp2r1b	ACAGCAGGCACAGACAGGCAGT#TGCCTAGTCTTCCCTTGCCTGCCTGG#.(((((((((.(((((((((..#))))).))))).....))))))).)).	3341	3367	1	-25	1	0.0001749	0.5	0.1	0.45	-7	18	26	7	3UTR
mmu-miR-214-3p	NM_001034905	Spag11b	ACAGCAGGCACAGACAGGCAGT#GCACTGTCTGGACTTGTG#...(((((..(((((((((...#)).))))))))..))))).	1181	1199	0.84615	-22	0	0.0008136	0.6	0	-0.2	-9	14	18	7	3UTR
mmu-miR-214-3p	NM_001034905	Spag11b	ACAGCAGGCACAGACAGGCAGT#ACTGCCTGAAGTCCCCTCTGCAGCCATGA#....(((((.((((((((((((#))))))))).......))))..))).)).	14	43	0.84615	-20	0	0.0552143	0.5	-1	0.03	-11	17	29	8	5UTR
mmu-miR-214-3p	NM_001037098	Nacc2	ACAGCAGGCACAGACAGGCAGT#GCTGCCTGTCTGCCCGGCTGTC#(((((.((..(((((((((((((#))))))))))))).)).))))).	5490	5512	1	-32	0	0.0039843	0.5	1.9	2.28	-8	19	22	12	3UTR
mmu-miR-214-3p	NM_001037098	Nacc2	ACAGCAGGCACAGACAGGCAGT#GCTGCCTGGATGTTCGTCTGCA#...(((((((((..((((((((#))))))))..)))..))))))).	383	405	0.92308	-28	0	5.37E-05	0.4	0.5	1.03	-9	17	22	8	5UTR

mmu-miR-214-3p	NM_001037177	Nfatc2	ACAGCAGGCACAGACAGGCAGT#CTGTCTGTCTGCTCTCTCTTGGGTCCTCTGG#.(((.(((..((((((((((.#))))))))))).............))))	2363	2394	1	-22	0	0.000333	0.5	1.7	1.52	-10	17	31	11	3UTR
mmu-miR-214-3p	NM_001037177	Nfatc2	ACAGCAGGCACAGACAGGCAGT#GCTGCAGAGAAACCCTCCTGGCTCTGTCTTCAGATGCTCCGTGCTCCTTCCTGCCCCAGGATCTTCGCTTGTGCTGCTGG#.(((((.((((((...(((((.#.)))))............))))))))))).	2108	2188	0.92308	-21	0	0.0014377	0.4	2.1	2.18	-7	16	31	11	CDS
mmu-miR-214-3p	NM_001037178	Nfatc2	ACAGCAGGCACAGACAGGCAGT#CTGTCTGTCTGCTCTCTCTTGGGTCCTCTGG#.(((.(((..((((((((((.#))))))))))).............))))	1728	1759	0.84615	-22	0	0.000333	0.5	2.5	3.87	-10	17	31	11	3UTR
mmu-miR-214-3p	NM_001037711	Cgn	ACAGCAGGCACAGACAGGCAGT#CTGCTCTGTCTTGCCTGCTT#..((((((((.((((((((((.#)))).)))))))))))))).	3755	3775	1	-30	1	0.0109007	0.5	2.1	1.28	-8	18	20	14	3UTR
mmu-miR-214-3p	NM_001037726	Creb1	ACAGCAGGCACAGACAGGCAGT#TTGTCCTGCTGAGCTTGCTGG#.((((((((.(((.(((((((.#))).))))))).)))))))).	2794	2815	0.92308	-26	1	8.12E-05	0.5	1	1.29	-6	18	21	8	3UTR
mmu-miR-214-3p	NM_001037726	Creb1	ACAGCAGGCACAGACAGGCAGT#GCTGCCTGTGGCCCGGGCGGCTGG#.((((.(.(....(((((((((#)))))))))......).).)))).	61	85	1	-24	0	5.03E-07	0.3	2.5	2.76	-17	15	24	9	5UTR
mmu-miR-214-3p	NM_001037726	Creb1	ACAGCAGGCACAGACAGGCAGT#CTGTCTGTGCTAGCA#...((.((((((((((((.....#))))))))))).)).	2834	2849	0.92308	-23	0	0.0003018	0.6	0.6	1.05	-8	13	15	11	3UTR
mmu-miR-214-3p	NM_001037736	Arhgef10	ACAGCAGGCACAGACAGGCAGT#GCTGCGTGTGCCCAGTGGTTACTCTGTGCCTGTGCCCTGTG#(((((((((((((((.(((((#)))))).)).............))))))))))....)))).	591	632	0.92308	-28	1	0.0001095	0.4	2.1	1.17	-4	21	41	10	CDS
mmu-miR-214-3p	NM_001037736	Arhgef10	ACAGCAGGCACAGACAGGCAGT#CTGCCATTGCTGCTGCCTGCC#...((((((((((.(((((((.#)))))..))))).)))))))).	652	673	1	-26	0	0.0159207	0.4	1.5	2.44	-7	17	21	7	CDS
mmu-miR-214-3p	NM_001037736	Arhgef10	ACAGCAGGCACAGACAGGCAGT#ACAGCAGTCTGTGTGACCTGCTGA#.((((((((((((((..((.((#)).)).)))))))...))))))).	3950	3974	0.84615	-25	1	1.50E-06	0.4	1.7	1.13	-4	18	24	7	CDS
mmu-miR-214-3p	NM_001037810	Spn	ACAGCAGGCACAGACAGGCAGT#ACTCCTGCTCCTGCCTGTC#...(((((((.((.((((.(((#)))))))))..))))))).	83	102	0.92308	-20	0	0.0289166	0.5	-0	-0	-10	16	19	9	5UTR

mmu-miR-214-3p	NM_001037846	Cnot2	ACAGCAGGCACAGACAGGCAGT#TTGGAAAATGTTTGCCCTGCTGTA#(((((((..((((((......#.)))))).)))))))).	2503	2527	1	-20	1	0.0123811	0.6	5.4	4.34	-6	14	17	8	3UTR
mmu-miR-214-3p	NM_001037848	Cnot2	ACAGCAGGCACAGACAGGCAGT#CTGTTTGTAGTGTGTATGCTC#..((((.(((((.(((((((.#))))))))..))))).)))).	735	756	0.92308	-21	0	0.0071247	0.6	4.8	4.42	-8	17	21	8	3UTR
mmu-miR-214-3p	NM_001037925	BC147527	ACAGCAGGCACAGACAGGCAGT#ACTGTTTGGAGCCCAGATGCCTGCTA#..(((((((((...((((((((#))))))))).......).)))))))).	1479	1505	0.92308	-21	1	0.0091835	0.5	0.1	0.05	-10	17	26	8	3UTR
mmu-miR-214-3p	NM_001037925	BC147527	ACAGCAGGCACAGACAGGCAGT#ATTTTCTGTCAGCTCCTGCTGTT#((((((((..(.((((((.(((#))).)))))).)..)))))))).	1522	1545	0.84615	-21	1	1.53E-05	0.6	0.1	0.09	-10	18	23	8	3UTR
mmu-miR-214-3p	NM_001038999	Atp8a1	ACAGCAGGCACAGACAGGCAGT#ATTGCCATGTCTCTTGTGTGTTCTGTG#(((((.(.((((((((((((((#)))))).)))))...)))).).)))).	1136	1163	0.84615	-23	0	0.0133841	0.5	0.6	0.6	-10	20	27	6	CDS
mmu-miR-214-3p	NM_001038999	Atp8a1	ACAGCAGGCACAGACAGGCAGT#GCTGCGTGGATAGTGTCTAATCTGTG#((((.((((((((..((.(((((#))))).))..).)))))))...)))).	7944	7970	0.84615	-22	0	0.0006857	0.5	1	1.15	-10	18	26	6	3UTR
mmu-miR-214-3p	NM_001038999	Atp8a1	ACAGCAGGCACAGACAGGCAGT#ACTGCTTTTCTGTCGTTTGTC#...(((((((((((.(((((((#))))))).))))).)))))).	4413	4434	0.92308	-22	0	0.1259908	0.6	-0	1.19	-7	18	21	7	3UTR
mmu-miR-214-3p	NM_001039154	Cdh8	ACAGCAGGCACAGACAGGCAGT#GTCTGTCTGGAACCACAACACTCACAGTGACCCTGACTGA#.((((((((..(((((((((...#))))))))).....................)))).))).	1353	1393	0.84615	-22	1	0.0019579	0.6	4.1	3.93	-6	16	40	9	CDS
mmu-miR-214-3p	NM_001039154	Cdh8	ACAGCAGGCACAGACAGGCAGT#AGTGCCTGTTGCTATTAAAGTGCTGG#.((((((((((...((((((((.(#).)))))))))))).......))))).	2026	2052	0.84615	-20	1	0.0063455	0.5	2.7	3.15	-11	17	26	11	CDS
mmu-miR-214-3p	NM_001039169	Eif4e2	ACAGCAGGCACAGACAGGCAGT#CTGTCTGTCTGTGGCCGCA#...((.(.(((((((((((((.#))))))))))))).).)).	1870	1889	1	-29	0	0.000202	0.5	0.6	0.58	-11	16	19	13	3UTR
mmu-miR-214-3p	NM_001039170	Eif4e2	ACAGCAGGCACAGACAGGCAGT#CTGTCTGTCTGTGGCCGCA#...((.(.(((((((((((((.#))))))))))))).).)).	1419	1438	1	-29	0	0.000202	0.5	0.6	0.58	-11	16	19	13	3UTR
mmu-miR-214-3p	NM_001039365	Mobp	ACAGCAGGCACAGACAGGCAGT#TTGCTTTTACCAGAAGAAGGAGGAGGACTGGATCTGCTGTGCCTGCC#...((((((((((.((((((((#.)))))..)))))))))))))).	288	335	0.89744	-28	0	0.0002019	0.4	2.5	2.57	-7	18	22	14	CDS

mmu-miR-214-3p	NM_001039365	Mobp	ACAGCAGGCACAGACAGGCAGT#GCTGTGTCTGTGCCA#......(((((((((..(((((#))))))))))))))).	837	852	0.96154	-23	0	4.48E-05	0.5	0.3	0.09	-16	14	15	14	3UTR
mmu-miR-214-3p	NM_001039466	Trim46	ACAGCAGGCACAGACAGGCAGT#GCTAGGCCTCTGGTGCCTGCTA#..(((((((((....(((((((#)))..))))...))))))))).	2607	2629	1	-24	1	7.74E-06	0.4	2.2	2.53	-11	16	22	9	3UTR
mmu-miR-214-3p	NM_001039466	Trim46	ACAGCAGGCACAGACAGGCAGT#GCCATCCTGTGCCAGTTGTG#(((((.((((((((...(((...#)))...))))))).))))).	521	541	0.92308	-24	0	0.000168	0.4	0.6	0.21	-8	15	20	7	CDS
mmu-miR-214-3p	NM_001039472	Kif21b	ACAGCAGGCACAGACAGGCAGT#CTGCCCTGCCCTGGCCTGCC#...((((((.(((.((((((((.#))))).))..))))))))).	8863	8883	1	-25	0	0.0016127	0.4	0	0.2	-9	16	20	9	3UTR
mmu-miR-214-3p	NM_001039563	Spag11b	ACAGCAGGCACAGACAGGCAGT#ACTGCCTGAAGTCCCCTCTGCAGCCATGA#....(((((.((((((((((((#))))))))).........)))))..))).)).	14	43	0.84615	-20	0	0.0534944	0.5	-1	0.03	-11	17	29	8	5UTR
mmu-miR-214-3p	NM_001039563	Spag11b	ACAGCAGGCACAGACAGGCAGT#GCTGTTTCCAGGCCTGTCCAATGCCTCGTC#...(((((((..(((((((...#.)))))))...))))).)).	89	119	0.92308	-21	0	0.0393975	0.5	0.3	-0.2	-13	14	20	7	CDS
mmu-miR-214-3p	NM_001039586	Glyctk	ACAGCAGGCACAGACAGGCAGT#CTGATCATGGTTTCTCCTCTCCTGCTGTTGCCTGACTGG#.((((((((((((.((((.((.#.))))))))))).))))).))).	2322	2361	0.84615	-25	1	0.0009536	0.4	-0	0.12	-5	18	22	10	3UTR
mmu-miR-214-3p	NM_001039586	Glyctk	ACAGCAGGCACAGACAGGCAGT#ACTGTCACCATTGCCTGCTGA#.(((((((((......((((((#))))))).....))))))))).	3349	3370	1	-24	1	0.0032604	0.5	2.1	3.31	-13	15	21	9	3UTR
mmu-miR-214-3p	NM_001039648	Gm20767	ACAGCAGGCACAGACAGGCAGT#TTGCAATTAGGGAATCTTCTGTCAGCTCCTGCTGTT#((((((((..(.((((((....#.)))))).)..))))	1282	1318	1	-21	1	5.18E-05	0.5	0	0	-10	15	20	8	3UTR
mmu-miR-214-3p	NM_001039648	Gm20767	ACAGCAGGCACAGACAGGCAGT#GCTCCTTCTCCTGCTTGCTGC#.(((((((((.((((.(((.(((#)))))))))..))))))))).	7	28	0.92308	-21	1	4.05E-06	0.5	0	0	-7	18	21	9	5UTR
mmu-miR-214-3p	NM_001039683	Eif4e1b	ACAGCAGGCACAGACAGGCAGT#ACTGCCACAGCTCATCACTGTGCCTGCA#...((((((((((...((((((#))))))...........)))))))))).	1037	1065	0.92308	-27	0	0.0130548	0.5	0	-0.2	-8	16	28	10	3UTR
mmu-miR-214-3p	NM_001039939	Asxl1	ACAGCAGGCACAGACAGGCAGT#GCTGCCTGTTGAAGAGGGTCCCAGGTGTGCTTACAAGTGGGCTGG#.(((((((((((((((((((((#)))))))))))...............)))))))........)))).	2680	2725	0.92308	-27	1	1.89E-05	0.4	0	0.43	-4	21	45	10	CDS

mmu-miR-214-3p	NM_001040187	Ddx17	ACAGCAGGCACAGACAGGCAGT#ACTGCATTCTTTGGCTGTGCCCTGAGGGTGCCTGCTC#..(((((((((...((((((((#.))))...))))...))))))))).	3779	3816	0.84615	-26	1	3.63E-06	0.5	4.2	3.9	-10	17	25	9	3UTR
mmu-miR-214-3p	NM_001040187	Ddx17	ACAGCAGGCACAGACAGGCAGT#GCCGCCATTTTGTGCAGTTGCTGG#.(((((((((((((..(((.((#)).)))..)))))))..)))))).	179	203	0.92308	-23	1	0.0021992	0.3	2.3	1.88	-5	18	24	7	5UTR
mmu-miR-214-3p	NM_001040187	Ddx17	ACAGCAGGCACAGACAGGCAGT#TGTCCTGTCTTGTCTGTC#...(((((((.(((((((((..#)).))))))))))))))).	4036	4054	0.92308	-22	0	0.2279617	0.5	3	3.05	-9	16	18	14	3UTR
mmu-miR-214-3p	NM_001040426	Thsd4	ACAGCAGGCACAGACAGGCAGT#CTGCCTGTCCCTCTCACTGTGCCTCTGC#.(((.((((((((((((((((.#)))))))))........)))))))))).	5080	5108	0.84615	-30	0	0.0770473	0.4	0.1	0.1	-8	19	28	10	3UTR
mmu-miR-214-3p	NM_001042499	Rabl3	ACAGCAGGCACAGACAGGCAGT#GCTCCTGTCTGCAGCGGCGCCCCGCTTCCTGCTC#..((((((..((((((((.(((#)))))))))))).........))))))).	1933	1967	0.92308	-22	1	0.0016711	0.3	0.1	0.27	-7	17	34	11	3UTR
mmu-miR-214-3p	NM_001042499	Rabl3	ACAGCAGGCACAGACAGGCAGT#ACTGCTTTCCTGGCTGAGGACTTCAATGCTGA#.((((((((.(((..(((((((#)))))))..))))))))........	510	542	0.84615	-20	1	0.0004093	0.6	4	3.66	-7	18	32	7	CDS
mmu-miR-214-3p	NM_001042593	Hbs1l	ACAGCAGGCACAGACAGGCAGT#CCTGTGCTGTTACACTACCAAACCGTCAGTGAGCCTGCTGTT#((((((((((((((((((....#))))).))))......................))))))))).	1934	1976	0.84615	-25	1	0.0015975	0.6	4.4	3.82	-4	18	42	9	CDS
mmu-miR-214-3p	NM_001042593	Hbs1l	ACAGCAGGCACAGACAGGCAGT#GCTGTTCTGTTTCCATTCTCCTGCTGTG#((((((((...((((((((((((#))))).))))))).......)))))))).	2507	2535	1	-23	1	0.0598518	0.6	0.7	0.74	-9	19	28	8	3UTR
mmu-miR-214-3p	NM_001042752	Neo1	ACAGCAGGCACAGACAGGCAGT#TTGCCTGCTGAGAGCCTGCA#...((((((.(((.(((((((.#))))))))))))...)))))).	4922	4942	0.87179	-27	0	1.68E-05	0.4	2.3	3.19	-9	16	20	10	3UTR
mmu-miR-214-3p	NM_001044740	Slc7a2	ACAGCAGGCACAGACAGGCAGT#GCTGCACTGTGTGGGTGTCTGCTGTT#((((((((((((..(((((((((#))))).))))....))))))))))))).	7052	7078	0.84615	-31	1	0.0001555	0.4	-0	0.14	-9	20	26	11	3UTR
mmu-miR-214-3p	NM_001044740	Slc7a2	ACAGCAGGCACAGACAGGCAGT#GCTGGGCTGTCGTGGGTCTGCTGTG#(((((((((.((((((((.((((#))))..))))).)).))))))))).	2251	2276	1	-28	1	1.72E-05	0.4	0.7	0.1	-5	20	25	9	3UTR

mmu-miR-214-3p	NM_001045520	Clint1	ACAGCAGGCACAGACAGGCAGT#ACAGTCGGAGAAGGAAGACTCGTGTGTTGTCTGTCTGTAGCGGTTGTT#(((((..((((((((((((((.#.))))))))))))).)).))))).	2750	2798	1	-26	0	0.0014846	0.5	0.4	1.15	-7	19	23	12	3UTR
mmu-miR-214-3p	NM_001045520	Clint1	ACAGCAGGCACAGACAGGCAGT#ACTGGATGTGTGTGCTTTTGCCAAGACTCCAGCAGTGCTGC#.(((((((((((.(((..((((#))))..))).))))))..................))))).	2567	2608	0.92308	-22	1	0.0002479	0.5	2.6	2.31	-5	18	41	6	3UTR
mmu-miR-214-3p	NM_001045528	Fkbp15	ACAGCAGGCACAGACAGGCAGT#CTGCTGAAGAGGATCATGAGGCTGTCTGCAGAACTGCTGTT#(((((((...(((((((.(...#.).))))))).....))))))).	3982	4023	0.84615	-24	1	0.0007157	0.5	3.3	2.23	-9	15	23	7	3UTR
mmu-miR-214-3p	NM_001045528	Fkbp15	ACAGCAGGCACAGACAGGCAGT#GGCTGTCTGCAGAACTGCTGTT#(((((((...(((((((.(...#).))))))).....))))))).	4001	4023	0.84615	-24	1	0.0007157	0.5	1.5	2.08	-9	15	22	7	3UTR
mmu-miR-214-3p	NM_001045528	Fkbp15	ACAGCAGGCACAGACAGGCAGT#ACAGCCTCTCTGCTGATCCTGTTGTG#((((((((((((((.((((.((#)).)))).)))).))..)))))))).	1120	1146	1	-23	1	0.0039879	0.4	0.5	0.52	-5	20	26	8	CDS
mmu-miR-214-3p	NM_001045959	Mink1	ACAGCAGGCACAGACAGGCAGT#GCTGCTTGCTGATAGCAATGGCTACACAAACCTGCCTGATGTG#(((.(((((((((.(((((((((#))))))))))).....................)))))).))).	2897	2940	1	-24	0	0.0011015	0.5	3.5	2.98	-8	20	43	11	CDS
mmu-miR-214-3p	NM_001045959	Mink1	ACAGCAGGCACAGACAGGCAGT#GCTGCCTTCCCAGCCTCCCACGACCCTGACCCTGCTGC#.(((((((..(((..(((((((#)))))))..................)))..))))))).	2025	2063	0.84615	-23	1	0.1190285	0.3	2.9	2.39	-8	17	38	7	CDS
mmu-miR-214-3p	NM_001045964	Mink1	ACAGCAGGCACAGACAGGCAGT#GCTGCTTGCTGATAGCAATGGCTACACAAACCTGCCTGATGTG#(((.(((((((((.(((((((((#))))))))))).....................)))))).))).	2918	2961	1	-24	0	0.0011015	0.5	3.5	2.98	-8	20	43	11	CDS
mmu-miR-214-3p	NM_001045964	Mink1	ACAGCAGGCACAGACAGGCAGT#GCTGCCTTCCCAGCCTCCCACGACCCTGACCCTGCTGC#.(((((((..(((..(((((((#)))))))..................)))..))))))).	2025	2063	0.84615	-23	1	0.1190285	0.3	2.9	2.39	-8	17	38	7	CDS
mmu-miR-214-3p	NM_001048192	Agbl5	ACAGCAGGCACAGACAGGCAGT#GCTTGCCTGTTTCCTGCTC#..((((((...((((((((((((#))).))))))))))))))).	2085	2104	0.92308	-23	1	0.0002095	0.5	0.8	1.63	-10	17	19	14	CDS

mmu-miR-214-3p	NM_001048192	Agbl5	ACAGCAGGCACAGACAGGCAGT#GCCTGACTGTGCTG#......(((((((.(((((...#))))).))))))).	298	312	1	-22	0	0.0003434	0.5	4.1	3.33	-16	12	14	7	CDS
mmu-miR-214-3p	NM_001077424	A4gnt	ACAGCAGGCACAGACAGGCAGT#GCTGAAGGAAATTTATCTCTCCCTGTCCCTGGTCCTGGTGTTTGCCTGTGGCCTGCTGTA#((((((((((((.((((((.(#.).)))))).))))).)	180	240	0.92308	-29	1	1.45E-05	0.4	1.2	0.75	-5	20	25	8	CDS
mmu-miR-214-3p	NM_001077424	A4gnt	ACAGCAGGCACAGACAGGCAGT#GCTGTCTATAGTCCATGCTCATGTGGCTGCTGG#.((((((.((((((((((((((#)))))))...))).......	1496	1529	0.92308	-26	1	2.94E-05	0.5	0.1	0.11	-6	20	33	7	3UTR
mmu-miR-214-3p	NM_001079830	Trim33	ACAGCAGGCACAGACAGGCAGT#ACTGCTTCGAGTCTGTTGCCTAGTA#...((((((((((((((((((#))))))))...))))))).)))).)).	7669	7694	0.92308	-24	0	0.0060507	0.6	2.2	1.36	-7	19	25	7	3UTR
mmu-miR-214-3p	NM_001079830	Trim33	ACAGCAGGCACAGACAGGCAGT#CTGCTTGACTGAGTGAACAAACTGCTGTA#((((((((.(((.(((((((.#))))))).))).)).......))))))).	6627	6656	1	-23	1	0.0005271	0.5	0.5	0.23	-6	19	29	7	3UTR
mmu-miR-214-3p	NM_001079883	Bcl11b	ACAGCAGGCACAGACAGGCAGT#ATTGCTTGGTATTTGTGCTCTGCTT#..(((((((((((((((((((#))))))))...))))))).))))).	7632	7657	1	-25	1	0.0005827	0.7	4.9	3.29	-5	20	25	8	3UTR
mmu-miR-214-3p	NM_001079883	Bcl11b	ACAGCAGGCACAGACAGGCAGT#GGTGCTTTCTGTGTCTGCC#...(((((((((((.(((((.(#).)))))))))))))))).	7464	7483	0.84615	-25	0	0.013824	0.5	5.7	3.58	-7	17	19	16	3UTR
mmu-miR-214-3p	NM_001079883	Bcl11b	ACAGCAGGCACAGACAGGCAGT#GCCTAACCTGTGTCTGCG#...((((((((((..(((((...#)))))...)))))))))).	3049	3067	1	-25	0	0.000411	0.4	3	1.91	-8	14	18	10	3UTR
mmu-miR-214-3p	NM_001080127	Rnps1	ACAGCAGGCACAGACAGGCAGT#ACTGCTCTTCTGTTCGGCCGACCATGCCCTGCTGA#.(((((((((((((..((((((#))))))..)))))).............))).))))).	1432	1467	0.84615	-24	1	0.0006179	0.5	-0	-0.6	-5	19	35	6	3UTR
mmu-miR-214-3p	NM_001080127	Rnps1	ACAGCAGGCACAGACAGGCAGT#ACTGCTACTGCTGTGTTGGCA#...((.(((((((.((((((((#))))))..)))))))))).)).	869	890	0.84615	-21	0	7.37E-05	0.4	2.5	3.49	-9	17	21	10	CDS
mmu-miR-214-3p	NM_001080128	Rnps1	ACAGCAGGCACAGACAGGCAGT#ACTGCTCTTCTGTTCGGCCGACCATGCCCTGCTGA#.(((((((((((((..((((((#))))))..)))))).............))).))))).	1251	1286	0.84615	-24	1	0.0006179	0.5	-0	-0.6	-5	19	35	6	3UTR

mmu-miR-214-3p	NM_001080128	Rnps1	ACAGCAGGCACAGACAGGCAGT#ACTGCTACTGCTGTGTTGGCA#...((.(((((((.((((((((#)))))..)))))))))).)).	688	709	0.84615	-21	0	7.37E-05	0.4	2.5	3.49	-9	17	21	10	CDS
mmu-miR-214-3p	NM_001080550	Wfdc8	ACAGCAGGCACAGACAGGCAGT#GCTCTGCTGCTGCTGCTGCTGTT#((((((((((((((.(((((...#)).)))))).))))..))))))).	559	582	0.92308	-23	1	0.0007537	0.4	0.1	0.1	-5	18	23	7	CDS
mmu-miR-214-3p	NM_001080706	Btaf1	ACAGCAGGCACAGACAGGCAGT#GCTGCGTGTGCTGTTGTGAGCTTGCA#...(((((((((((((.(((((#))))).))).))))).....)))))).	2883	2909	0.92308	-25	0	0.0001949	0.5	3.6	4.03	-7	18	26	6	CDS
mmu-miR-214-3p	NM_001080755	Zzz3	ACAGCAGGCACAGACAGGCAGT#GCTGCTTGCCTGCATGTCTGCTGC#.((((((((((((.((((((((#)))))))).)))..))))))))).	7131	7155	0.92308	-30	1	2.88E-07	0.4	0.1	0.25	-5	20	24	9	3UTR
mmu-miR-214-3p	NM_001080755	Zzz3	ACAGCAGGCACAGACAGGCAGT#GCTGCCAGTGTGTGCTGTGTGTTGG#.(((((((((((.((.((((((#)))))).)).))))))...))))).	7179	7204	0.84615	-27	1	0.0003369	0.5	0.6	0.17	-5	19	25	6	3UTR
mmu-miR-214-3p	NM_001080773	Pdpk1	ACAGCAGGCACAGACAGGCAGT#CCTGTACCCGGTTTTACACGGCTGAGATTGTGTCTGCTT#..((((((((((((.(((.(.((#.))).)))...))))))))))).	681	720	0.88462	-21	1	0.0035305	0.5	4.2	4.04	-6	17	23	11	CDS
mmu-miR-214-3p	NM_001080814	Fat3	ACAGCAGGCACAGACAGGCAGT#CTGTCTGTTTTTGCCACCAGCAAAGATATTGGTACAAATGCTGA#.(((((((((.((((((((((.#))))))))))).))))......................))))).	9961	####	0.84615	-24	1	0.0102657	0.6	2.3	2.9	-7	19	44	10	CDS
mmu-miR-214-3p	NM_001080814	Fat3	ACAGCAGGCACAGACAGGCAGT#GCCTCTGTACAGTCTCAACGTGTCTGTGTCTGATGG#.((.((((((((((((.(....#.).)))))))))))	7581	7617	1	-24	0	3.61E-05	0.5	2.2	2.91	-7	15	19	12	CDS
mmu-miR-214-3p	NM_001080814	Fat3	ACAGCAGGCACAGACAGGCAGT#GCTGCCAAAACTGGGCCCTGTTGG#.((((((((.(((...((((((#))))))....))).))).))))).	14641	####	1	-24	1	0.0009797	0.5	4.1	3.45	-7	17	24	6	3UTR
mmu-miR-214-3p	NM_001080925	Rapgefl1	ACAGCAGGCACAGACAGGCAGT#GCGTGTCTCGCCTGCTGC#.((((((((..(((((.((...#)).))))).)))))))).	541	559	0.84615	-28	1	0.0002025	0.3	1.7	1.3	-8	15	18	8	CDS
mmu-miR-214-3p	NM_001080930	Atxn1l	ACAGCAGGCACAGACAGGCAGT#ATTGTCTGTCTATGTTGTGCCTTAGTGCCTGCA#...(((((((((((((((((((#)))))))))))))............	3370	3403	1	-29	0	0.0002849	0.4	2.4	1.51	-7	19	33	11	3UTR

mmu-miR-214-3p	NM_001080930	Atxn1l	ACAGCAGGCACAGACAGGCAGT#ACTGCTGTTGTCTGCTCATTGTCCTGCCTGTTGC#.((((((((((((((((((((#))))))..))))))..........)))))))).	5397	5431	1	-28	1	0.0541656	0.5	1.8	2.42	-4	21	34	9	3UTR
mmu-miR-214-3p	NM_001080930	Atxn1l	ACAGCAGGCACAGACAGGCAGT#TTGTTTGCTCTGTGCTGCTGTG#((((((.((((((((((((((.#))))))).))))))))))))).	5373	5395	0.92308	-28	0	0.000273	0.5	1.3	1.59	-5	20	22	13	3UTR
mmu-miR-214-3p	NM_001081022	Katnip	ACAGCAGGCACAGACAGGCAGT#GCTGCTGGGAGCCAGGGATACCTGATTCCTCTCTGTGCCTCCCCTGTG#((((.(((((((((.((((((.#.)))...))).)))))))))...)))).	5119	5167	0.92308	-25	0	0.0002576	0.4	0.1	-0.2	-9	19	28	9	3UTR
mmu-miR-214-3p	NM_001081039	Dock9	ACAGCAGGCACAGACAGGCAGT#TGAACCCGGGCAGTATCGTGAAGGACGAAGCCCTGGCTGTGCCTGCTGG#.((((((((((((.((((....#.)))).)))))))))))).	3657	3706	1	-32	1	4.96E-05	0.4	4.4	4.62	-5	16	19	12	CDS
mmu-miR-214-3p	NM_001081102	Nsd2	ACAGCAGGCACAGACAGGCAGT#ACTGTCTGTCCTTTGGCTTGCTT#..((((((((.((((((((((((#))))))))))...))))))))).	5152	5175	0.84615	-26	1	0.0001266	0.4	-1	-0.5	-6	19	23	10	3UTR
mmu-miR-214-3p	NM_001081102	Nsd2	ACAGCAGGCACAGACAGGCAGT#GCTGCCTTTGTTTTGCCTGTTT#..((((((((.((((((((((((#)))))))..))))))))))))).	4769	4791	0.92308	-25	1	0.0579734	0.5	-0	-0.3	-8	19	22	12	3UTR
mmu-miR-214-3p	NM_001081102	Nsd2	ACAGCAGGCACAGACAGGCAGT#TGCCTGTCTGGCAGCA#...((..((.((((((((((..#))))))))))).)).	2419	2435	1	-25	0	2.21E-06	0.5	2.9	3.11	-11	14	16	12	CDS
mmu-miR-214-3p	NM_001081102	Nsd2	ACAGCAGGCACAGACAGGCAGT#ACTCACTGCTGTCCTGCTGC#.(((((((.((((.(((..(((#)))..)))))))))))))).	5329	5349	0.98077	-24	1	0.0002404	0.5	-1	-0.1	-6	17	20	14	3UTR
mmu-miR-214-3p	NM_001081103	Stim2	ACAGCAGGCACAGACAGGCAGT#TTGCCTTCCCCAGATCCAGACATCCTGTCTGTGTCAAGTTGCCCTGCTC#..(((((((((((((((((..((#.))..)))))))))).........)))))).	2020	2069	1	-26	1	0.0003609	0.5	3.3	1.77	-5	18	31	10	CDS
mmu-miR-214-3p	NM_001081103	Stim2	ACAGCAGGCACAGACAGGCAGT#GCCTGTCGGCTGCGGGAGCTGA#.((((..((((.((((((((...#))))))).).)))....)))).	1261	1283	0.84615	-23	0	4.23E-07	0.3	2.5	3.66	-11	15	22	7	CDS
mmu-miR-214-3p	NM_001081110	Cd8a	ACAGCAGGCACAGACAGGCAGT#GCTTTCTGTCGCTGAACCTGCTGC#.((((((((((..((((((.(((#))).))))))..))..)))))))).	145	169	0.84615	-23	1	0.0034098	0.4	0.7	0.06	-10	18	24	7	CDS

mmu-miR-214-3p	NM_001081110	Cd8a	ACAGCAGGCACAGACAGGCAGT#GCTGACTGTCAAAACTGCTGC#.((((((.....(((((.((((#)))).)))))....)))))).	2796	2817	0.84615	-21	1	0.0002509	0.5	0.4	-0	-15	15	21	6	3UTR
mmu-miR-214-3p	NM_001081110	Cd8a	ACAGCAGGCACAGACAGGCAGT#CTGGGTCCCTGTGTCTGCGTGG#.((((((((((((.....(((.#))).....)))))))))).)).	1581	1603	0.84615	-21	1	1.24E-06	0.4	0	0.03	-6	15	22	10	3UTR
mmu-miR-214-3p	NM_001081144	Zfp518b	ACAGCAGGCACAGACAGGCAGT#AGTGCCTGCTGCTCCACCAGTCACTGGATGTGGGAGGATGCCTGCA#...(((((((((.((((((.(#).)))))))))).........................)))))))).	609	655	0.92308	-24	0	4.88E-05	0.5	0	0.11	-7	17	46	9	5UTR
mmu-miR-214-3p	NM_001081144	Zfp518b	ACAGCAGGCACAGACAGGCAGT#CTGCCTGCTGCTCTGTGGCCATGTG#...(((((((((((((((((.#)))))))....)))))).)).))).	5815	5840	0.92308	-24	0	0.0002458	0.5	0.2	1.21	-7	18	25	7	3UTR
mmu-miR-214-3p	NM_001081144	Zfp518b	ACAGCAGGCACAGACAGGCAGT#TTGTCCTGTCTGAGAGGACTAGGTGTCAGCTGG#.((((.((((((((((((((((.#))).)))))))).........)))))).)))).	3646	3679	1	-24	0	4.37E-06	0.4	0.1	0.38	-6	19	33	7	CDS
mmu-miR-214-3p	NM_001081176	Polr3g	ACAGCAGGCACAGACAGGCAGT#ACTCTTGTGTGTTCACAGTTTAAAAAGCTCACTCTGGCTCACCTGCTGA#.(((((((.(((.(((((.(((#)))))))).)))...........................))))))	1109	1158	0.84615	-19	1	0.0001104	0.5	0.3	0.21	-6	18	49	8	3UTR
mmu-miR-214-3p	NM_001081183	Virma	ACAGCAGGCACAGACAGGCAGT#GCTGATGACTGCAATCTGCGACTGTCTGCTGG#.((((((((((((...(((((#.)))))..))))....))))))	4194	4226	1	-25	1	7.47E-05	0.5	2.5	2.65	-5	18	26	9	CDS
mmu-miR-214-3p	NM_001081183	Virma	ACAGCAGGCACAGACAGGCAGT#TTGCCTTGTGGCTGCA#...((((.((((...((((((.#)))))))))).))))).	2501	2517	1	-21	0	1.58E-05	0.5	3.4	3.06	-12	14	16	10	CDS
mmu-miR-214-3p	NM_001081201	Dpy19l4	ACAGCAGGCACAGACAGGCAGT#TGCTTGTTAAGTGCCTTCAGCTGA#.(((((((((.((((((((..#))))))))..))))))...)))).	1101	1125	0.92308	-25	1	0.0005843	0.6	0.6	0.47	-7	18	24	8	CDS
mmu-miR-214-3p	NM_001081201	Dpy19l4	ACAGCAGGCACAGACAGGCAGT#GCTGTTCCTGTCACCTGCTGC#.(((((((....((((((((((#))))..)))))).))))))).	2797	2818	0.84615	-24	1	0.0043664	0.5	-0	0.54	-12	17	21	7	3UTR
mmu-miR-214-3p	NM_001081201	Dpy19l4	ACAGCAGGCACAGACAGGCAGT#GCTGCTGTTCCTCCTGCACCTGCTGA#.((((((..((((((.(((((#))))))))).....)))..))))))).	2393	2419	0.92308	-23	1	0.0006607	0.5	0.4	-0.1	-6	18	26	8	3UTR

mmu-miR-214-3p	NM_001081204	B3glct	ACAGCAGGCACAGACAGGCAGT#GCTGCATCTGGAGCTGGCCTGTTACTCCTGCTGTT#((((((((....((((((((((#.)))).))))))...))	2503	2538	0.92308	-27	1	0.0097421	0.5	0	-0	-12	18	24	8	3UTR
mmu-miR-214-3p	NM_001081217	Zfp174	ACAGCAGGCACAGACAGGCAGT#GCTGCCTTGCCTGCTTGTC#((((((((((.....(((((((#))))))))))))))).)).	3791	3810	1	-25	1	0.0030828	0.5	0.2	0.17	-13	17	19	15	3UTR
mmu-miR-214-3p	NM_001081217	Zfp174	ACAGCAGGCACAGACAGGCAGT#TGTGTGTGTGTGTGTGTCTGA#.(((((.(((((.(((.(((..#))).))).))))).)).))).	2481	2502	0.92308	-23	0	0.1924282	0.6	-0	-0	-7	16	21	5	3UTR
mmu-miR-214-3p	NM_001081252	Uggt2	ACAGCAGGCACAGACAGGCAGT#ACTGGCTGTGGTGTGAAACCTGGTGTG#(((.((((((((.((((.((((#)))).)))))..))))...)))).))).	4393	4420	0.92308	-25	0	0.0187104	0.5	2.6	1.81	-8	19	27	4	CDS
mmu-miR-214-3p	NM_001081252	Uggt2	ACAGCAGGCACAGACAGGCAGT#GCCCCTTGGCTACATGTGTGTGCTGGGCTGTA#(((((.((((((.((((((...#)))...........))).))))))	5404	5436	0.84615	-24	0	1.89E-05	0.5	2.8	2.51	-7	17	32	6	3UTR
mmu-miR-214-3p	NM_001081269	Nsd3	ACAGCAGGCACAGACAGGCAGT#ATTGCCTGTAACATTTGAGCTTCTTACTGATGTGTGCTGG#.(((((.(((((((((((((((#)))))))))................))).))).))))).	9432	9472	0.84615	-26	0	0.0090161	0.6	2.3	2.19	-5	20	40	9	3UTR
mmu-miR-214-3p	NM_001081269	Nsd3	ACAGCAGGCACAGACAGGCAGT#CCAGGTCTGTGCCGCTGA#.((((.(((((((((.((....#))..))))))))))))).	3015	3033	1	-26	0	8.27E-06	0.4	3.8	4.74	-6	15	18	13	CDS
mmu-miR-214-3p	NM_001081293	Rprd2	ACAGCAGGCACAGACAGGCAGT#GCCTGTGGAGAAACCTGCTGTC#((((((((.....((((((...#))))))).......)))))))).	1428	1450	1	-24	1	0.0001424	0.5	0.7	0.89	-14	14	22	8	CDS
mmu-miR-214-3p	NM_001081293	Rprd2	ACAGCAGGCACAGACAGGCAGT#GCTTGTCGTCCTGGTGTA#(((.((((.((.(((((((...#))))))))))))).)))).	329	347	1	-23	0	5.83E-05	0.6	0.5	0.59	-11	16	18	13	CDS
mmu-miR-214-3p	NM_001081293	Rprd2	ACAGCAGGCACAGACAGGCAGT#GCTTGTCTCAGTCTACTGCTGG#.((((((..((((((((((...#))))))))..))...)))))).	3095	3117	0.92308	-23	1	1.87E-07	0.5	-0	0.21	-7	16	22	8	CDS
mmu-miR-214-3p	NM_001081293	Rprd2	ACAGCAGGCACAGACAGGCAGT#ACTGCGAACCCTGTGCCAGCC#...((.(((((((....((((((#)))))).....))))))).)).	1897	1918	1	-22	0	0.008102	0.5	0.5	0.52	-10	14	21	7	CDS
mmu-miR-214-3p	NM_001081304	Atf6	ACAGCAGGCACAGACAGGCAGT#GCTTGCCTTCTCTGCTCCTGCTGA#.((((((((..((((.(((((((#))).))))..))))..)))))))).	4090	4114	0.84615	-25	1	0.000752	0.5	-0	-0.3	-7	18	24	7	3UTR

mmu-miR-214-3p	NM_001081304	Atf6	ACAGCAGGCACAGACAGGCAGT#CTGCCTGTGAAGCAGCTCAACACGAGGATGTGACTGTGGCTGTG#...((((.(((((((((((((.#))))))))).......................))))).)))).	3791	3835	0.84615	-25	0	5.26E-05	0.4	0.1	-0.1	-9	17	44	8	3UTR
mmu-miR-214-3p	NM_001081304	Atf6	ACAGCAGGCACAGACAGGCAGT#CTGCGGTTCTGTCGTCTGCTC#..((((((((((((...((((.#))))...))))).))))))).	767	788	0.92308	-23	1	0.0049556	0.4	-0	0.04	-6	16	21	7	CDS
mmu-miR-214-3p	NM_001081343	C9orf72	ACAGCAGGCACAGACAGGCAGT#CTGCCCCTCTGTGTTCTGTGGTA#((.((((((((((..(((((.#)))))..))))))).)))).)).	1945	1968	0.92308	-25	0	0.0022271	0.5	4.3	3.12	-6	18	23	7	3UTR
mmu-miR-214-3p	NM_001081343	C9orf72	ACAGCAGGCACAGACAGGCAGT#GCTGCCGTGTGCCTGGCTT#..((((((((((....((((((#)))))).))))))))).)).	2407	2426	0.84615	-23	1	3.58E-05	0.5	4	3.85	-8	16	19	8	3UTR
mmu-miR-214-3p	NM_001081343	C9orf72	ACAGCAGGCACAGACAGGCAGT#CTGCCTGTACTCTGGTGTTTCCCGTGTCACCTGCA#...((((((((((((((((((.#)))))))).))...))).............))))).	2517	2552	0.84615	-22	0	0.0004624	0.5	3.5	3.31	-7	18	35	8	3UTR
mmu-miR-214-3p	NM_001081345	Chd2	ACAGCAGGCACAGACAGGCAGT#CTGGCTAGAGTCTGATGCCTGGTGG#.((.(((((((((((((.(((.#))).))...))))).)))))).)).	7834	7859	0.92308	-27	0	1.93E-06	0.5	3.6	3.23	-7	18	25	6	3UTR
mmu-miR-214-3p	NM_001081345	Chd2	ACAGCAGGCACAGACAGGCAGT#ATTGACACTGTCTGTGTTTGATTTGCTGA#.((((((((((((((((((((#)))).).)))))))))).....)))))).	7946	7975	0.84615	-25	1	0.0004973	0.5	2.2	2.31	-4	21	29	10	3UTR
mmu-miR-214-3p	NM_001081376	Chd5	ACAGCAGGCACAGACAGGCAGT#GCTGCAACCATCCGTACCTCTTCCCTGTGGCTGCTGTG#(((((((.(((((....(((((#)))))..................))))).))))))).	3281	3319	0.84615	-25	1	0.0008816	0.4	2.6	3.72	-8	17	38	7	CDS
mmu-miR-214-3p	NM_001081376	Chd5	ACAGCAGGCACAGACAGGCAGT#CTGCCAATGCTGTCCTGCA#...(((((.((((.(((((((.#)))))..))))))))))).	5849	5868	0.84615	-23	0	0.0009532	0.4	4.5	4.43	-8	16	19	11	CDS
mmu-miR-214-3p	NM_001081394	Tmem248	ACAGCAGGCACAGACAGGCAGT#CTGTCTCAGTGTGCTTGCA#...(((((((((...((((((.#))))))...))))))))).	1629	1648	1	-24	0	0.0050568	0.7	3.1	0.98	-9	15	19	9	3UTR
mmu-miR-214-3p	NM_001081400	1810013L24Rik	ACAGCAGGCACAGACAGGCAGT#ACTGCCGCTCCTGTCCCTTCCTCCCTGGCCTGCC#...((((((((((...((((((#))))))....)))).............)))))).	3423	3457	0.84615	-23	0	0.0009242	0.4	0.1	-0.1	-8	16	34	6	3UTR

mmu-miR-214-3p	NM_001081423	Ttll5	ACAGCAGGCACAGACAGGCAGT#CTGCCCTCCCCCTGCCCTGCTAGGTCCCTCTGTCTCTCCTCCTGCTGC#.(((((((...((((((((((.#.)))))).............))))).....))))))).	4554	4602	0.84615	-23	1	0.0260191	0.4	0.2	0.74	-9	17	38	7	3UTR
mmu-miR-214-3p	NM_001081423	Ttll5	ACAGCAGGCACAGACAGGCAGT#CCTGCTGTGCGCTCCTGG#.(((.((((((((.((((....#))))))))))).)).))).	4301	4319	1	-22	0	0.0001139	0.4	-1	0.35	-9	15	18	10	3UTR
mmu-miR-214-3p	NM_001081436	Ino80d	ACAGCAGGCACAGACAGGCAGT#ACTGCCTGTGCCTGCTTGTTCACCCTGTGTGTGTTGTG#((((((.((((((((((((((#)))))))))..............)))))).)))))).	3811	3849	1	-30	0	0.0100131	0.5	1.6	1.58	-5	21	38	9	3UTR
mmu-miR-214-3p	NM_001081557	Camta1	ACAGCAGGCACAGACAGGCAGT#GCAAGAAGCCAGCAATAATTACAGTTGCCTGTTTGACCAGATCTCCGTGCCTGCA#...(((((((((((((((((.#.))))))))))............)))))	2789	2844	1	-28	0	0.0194984	0.5	2.2	1.7	-7	18	32	10	CDS
mmu-miR-214-3p	NM_001081557	Camta1	ACAGCAGGCACAGACAGGCAGT#GCTGCTTCGAGAGCCGTGTGGTTGTCGTGTGTGAGAAGATGATGAGCCGGGCCTGCTGG#.((((((((((((((((((((#))))))...............)))).)))...................)))))))).	3199	3258	0.81538	-24	1	1.30E-07	0.5	3.1	2.44	-4	21	59	8	CDS
mmu-miR-214-3p	NM_001081633	Ess2	ACAGCAGGCACAGACAGGCAGT#GCTACGCCTGTTAGCCTGTGCCTACAGAGAGCTGA#.(((((((((((((((((((((#)))..))))))....)))))))).......)))).	2236	2271	0.84615	-27	1	0.0001486	0.4	2.7	3.16	-4	21	35	8	3UTR
mmu-miR-214-3p	NM_001081633	Ess2	ACAGCAGGCACAGACAGGCAGT#GCTGCCTCCCTGTCCTCCTCTCTGCTTGACCCACTGTTCTTCAGCTCTGCCTGCTGG#.(((((((((((((.(((((((#)))))))............)))).......................))))))))).	2045	2102	0.92308	-26	1	9.70E-06	0.5	3.5	3.13	-5	20	57	9	3UTR
mmu-miR-214-3p	NM_001081633	Ess2	ACAGCAGGCACAGACAGGCAGT#CTGGAACTGTCTGGCCAGGCTGC#.((((.(((.(((((((.(((.#)))...))))))))))..)))).	1751	1774	1	-25	0	0.0007536	0.4	2.8	3.44	-7	17	23	10	3UTR
mmu-miR-214-3p	NM_001083126	Lhx6	ACAGCAGGCACAGACAGGCAGT#GCTGCCTCTTCCGTGCCGTCTGC#.(((..(((((.(((.(((((((#)))))))..)).)))))..))).	269	292	0.84615	-25	0	0.0051612	0.4	1.9	1.06	-14	17	23	7	CDS
mmu-miR-214-3p	NM_001083127	Lhx6	ACAGCAGGCACAGACAGGCAGT#GCTGCCTCTTCCGTGCCGTCTGC#.(((..(((((.(((.(((((((#)))))))..)).)))))..))).	269	292	0.84615	-25	0	0.0051612	0.4	1.9	1.06	-14	17	23	7	CDS

mmu-miR-214-3p	NM_001083312	Gbp7	ACAGCAGGCACAGACAGGCAGT#CCTGGCTGTGCTTCTGA#.(((.((((((((.((((....#)))).))))))))))).	460	477	1	-22	0	1.50E-05	0.5	1.7	1.75	-9	15	17	11	CDS
mmu-miR-214-3p	NM_001083312	Gbp7	ACAGCAGGCACAGACAGGCAGT#GCTCCTGTGTGCCTAGTGG#.((..((((((..(((((.(((#))))))))))))))..)).	134	153	0.92308	-21	0	0.0004662	0.5	1.5	0.32	-16	16	19	14	CDS
mmu-miR-214-3p	NM_001083317	Slc35a4	ACAGCAGGCACAGACAGGCAGT#GCCTGTCCTTGAAGATCCCTGCCTTGTCCTGCATGTA#((((((((.((((((((((...#))))))).....…………)))))))).))).	2221	2258	0.92308	-24	1	0.0108874	0.5	-0	-0	-5	18	37	8	3UTR
mmu-miR-214-3p	NM_001083320	Magi1	ACAGCAGGCACAGACAGGCAGT#TGAAAAGTCTTGTGCCTGGTGTA#(((.((((((((((((.......#.)))).))))))).))).	4547	4570	1	-24	0	0.1128847	0.8	4.7	3.8	-7	14	18	7	3UTR
mmu-miR-214-3p	NM_001083320	Magi1	ACAGCAGGCACAGACAGGCAGT#ACTGCCTAGCTGAGCCAAAGTGTTTGCTTGTA#(((((((((.(((..(((((((#))))))).))).))).……	5646	5678	0.84615	-23	1	0.0073733	0.5	4.5	4.24	-7	19	32	7	3UTR
mmu-miR-214-3p	NM_001083320	Magi1	ACAGCAGGCACAGACAGGCAGT#GTGTGGTTGTGTCGGCTGTA#(((((.((((((..((.((...#)).)).))))))).))))).	5600	5620	1	-23	0	0.0006008	0.5	4.8	4.91	-8	15	20	6	3UTR
mmu-miR-214-3p	NM_001083321	Magi1	ACAGCAGGCACAGACAGGCAGT#GCTGCACACTGTGCTTGACTT#..(((((((((((....(((((#)))))...))))))))).)).	4862	4883	0.92308	-22	1	0.0015537	0.5	1	1.52	-7	16	21	9	3UTR
mmu-miR-214-3p	NM_001083321	Magi1	ACAGCAGGCACAGACAGGCAGT#TGCATCTGTTGTGCCTCCTGTT#((((.((((((((.(((((((..#))))..)))))))))))).)))).	1199	1221	1	-22	0	0.0005807	0.4	1.8	2.54	-9	18	22	11	CDS
mmu-miR-214-3p	NM_001083321	Magi1	ACAGCAGGCACAGACAGGCAGT#ACAACCCGGTCTCCCAGAGAAGGAGAAGTGCCTGGTGTG#(((.(((((((((((.((..((#))..))..))))).…………..))))))).))).	369	408	0.92308	-21	0	0.0030831	0.5	0.9	0.9	-7	18	39	7	5UTR
mmu-miR-214-3p	NM_001083880	1600012H06Rik	ACAGCAGGCACAGACAGGCAGT#GCTGCAGTCTGTCACTTGCG#...(((((.((((((..(((((#))))).))))))..))))).	437	457	0.84615	-22	0	0.0005046	0.4	2	2.11	-8	16	20	6	CDS
mmu-miR-214-3p	NM_001083881	1600012H06Rik	ACAGCAGGCACAGACAGGCAGT#GCTGCAGTCTGTCACTTGCG#...(((((.((((((..(((((#))))).))))))..))))).	507	527	0.84615	-22	0	0.0005046	0.4	2	2.11	-8	16	20	6	CDS
mmu-miR-214-3p	NM_001083927	Tle3	ACAGCAGGCACAGACAGGCAGT#GCTCGGCCCCCTTCGCCTGTCTGTGTGTGGTA#..(.((.((((((((((((...#.))))))))))))).)).).	42	74	1	-28	0	7.50E-06	0.4	1.4	2.46	-10	15	19	12	5UTR

mmu-miR-214-3p	NM_001083927	Tle3	ACAGCAGGCACAGACAGGCAGT#GCTCGCCGTGGATTTTGCCTGCTGTA#((((((((((.(..((((((((#))).))).))..)..)))))))))).	3893	3919	0.92308	-26	1	0.0093044	0.5	2.7	0.75	-9	19	26	10	3UTR
mmu-miR-214-3p	NM_001083927	Tle3	ACAGCAGGCACAGACAGGCAGT#TGCCAAAGTCTGTTTTTCCTGCTGC#.((((((.((((((.((((..#))))...))))))....))))))).	2862	2887	0.80769	-25	1	0.0167808	0.5	4.8	4.37	-5	17	25	7	CDS
mmu-miR-214-3p	NM_001083928	Tle3	ACAGCAGGCACAGACAGGCAGT#GCTCGGCCCCCTTCGCCTGTCTGTGTGTGGTA#..(.((.((((((((((((...#.)))))))))))).)).).	42	74	1	-28	0	7.50E-06	0.4	1.4	2.46	-10	15	19	12	5UTR
mmu-miR-214-3p	NM_001083928	Tle3	ACAGCAGGCACAGACAGGCAGT#GCTCGCCGTGGATTTTGCCTGCTGTA#((((((((((.(..((((((((#))).))).))..)..)))))))))).	3839	3865	0.92308	-26	1	0.0093044	0.5	2.7	0.75	-9	19	26	10	3UTR
mmu-miR-214-3p	NM_001083928	Tle3	ACAGCAGGCACAGACAGGCAGT#TGCCAAAGTCTGTTTTTCCTGCTGC#.((((((.((((((.((((..#))))...))))))....))))))).	2808	2833	0.80769	-25	1	0.0167808	0.5	4.8	4.37	-5	17	25	7	CDS
mmu-miR-214-3p	NM_001085385	1600014C10Rik	ACAGCAGGCACAGACAGGCAGT#GCTGTGTGTGCTATGGAAGGGTACAGGCGTGCCTGCTGTA#((((((((((((((((.(((((#)))))).)))).))...............))))))))))).	926	966	0.92308	-30	1	6.31E-07	0.4	0	0	-4	21	40	11	3UTR
mmu-miR-214-3p	NM_001085385	1600014C10Rik	ACAGCAGGCACAGACAGGCAGT#GCCTGTTAGGTGCCTGG#....(((((((.(((((((...#)))))))..))))))).	491	508	0.89744	-27	0	1.84E-05	0.4	0	0	-13	14	17	7	CDS
mmu-miR-214-3p	NM_001085385	1600014C10Rik	ACAGCAGGCACAGACAGGCAGT#CTCCTGTCTGCTGGCTGCA#...((((.((((((((((.((.#)))))))))))).)).)))).	1583	1602	0.92308	-25	0	0.0031527	0.6	0	0	-9	16	19	10	3UTR
mmu-miR-214-3p	NM_001085409	Steap3	ACAGCAGGCACAGACAGGCAGT#CTGCCTGCCGCTGCGCCGCTC#..(((.(((.(((.(((((((.#)))))))...))).)))))).	1390	1411	0.84615	-25	0	0.0030076	0.3	2	2.44	-9	16	21	7	CDS
mmu-miR-214-3p	NM_001085409	Steap3	ACAGCAGGCACAGACAGGCAGT#CTGTCCCTGGGTGTGCTGGCCCTGGGCATGTTGTCGCTGCTGG#.(((((((((((..(((((((.#)))..)))))..)))))...................)))))).	1508	1551	0.84615	-23	1	0.0014271	0.4	3.6	3.03	-6	18	43	6	CDS
mmu-miR-214-3p	NM_001085410	Nadk2	ACAGCAGGCACAGACAGGCAGT#ACGCGTGTGCTCTCCTGCTGA#.(((((((.....(((.(((.((#)))).)))).....))))))).	1730	1751	1	-19	1	0.0006188	0.5	0.2	0.56	-14	14	21	7	3UTR
mmu-miR-214-3p	NM_001085549	Trabd2b	ACAGCAGGCACAGACAGGCAGT#CTGCCAAAAGCCTGCTGTG#(((((((((.......(((((.#)))))....))))))))).	2620	2639	0.92308	-24	1	0.0073892	0.5	0.8	-0.1	-14	14	19	9	3UTR

mmu-miR-214-3p	NM_001098836	Atxn7l3	ACAGCAGGCACAGACAGGCAGT#ACTGCCTCTGGCCTGG#....(((((.((((..((((((#))))))))))))))).	1265	1281	1	-24	0	8.59E-05	0.5	1.3	1.02	-12	15	16	15	CDS
mmu-miR-214-3p	NM_001098837	Atxn7l3	ACAGCAGGCACAGACAGGCAGT#ACTGCCTCTGGCCTGG#....(((((.((((..((((((#))))))))))))))).	1244	1260	1	-24	0	8.59E-05	0.5	0.6	0.75	-12	15	16	15	CDS
mmu-miR-214-3p	NM_001099785	D3Ertd751e	ACAGCAGGCACAGACAGGCAGT#GGTGCCGGTCTAGGTGTCTGCC#...((((((((((((.((((.(#).)))).)))).))))..)))))))).	2927	2949	0.92308	-25	0	2.04E-05	0.5	0	0.01	-7	17	22	8	3UTR
mmu-miR-214-3p	NM_001099785	D3Ertd751e	ACAGCAGGCACAGACAGGCAGT#GCCAGTGTGTGTCAGGCTGA#.((((.((((((.((.(((...#))).)).))))))..)))).	122	142	1	-24	0	0.0001776	0.4	0.9	2.18	-7	15	20	6	CDS
mmu-miR-214-3p	NM_001100454	Wfikkn1	ACAGCAGGCACAGACAGGCAGT#CTGTACCACGCACACCACCCTGTCGTGCCTGCC#...((((((((.((((((....#.)))))))))))))).	2055	2088	1	-27	0	0.0040855	0.4	0.1	0.32	-10	14	16	14	CDS
mmu-miR-214-3p	NM_001100454	Wfikkn1	ACAGCAGGCACAGACAGGCAGT#GCATGCCAGCAGGCCTGTGTTCGTGGCCCCGGGGATGTCTGTGCACTGCCTGC#.((((((((((((((((((...#.)))......))))))))).)))).)).	1862	1915	0.84615	-26	1	0.0002763	0.3	-0	-0.6	-4	18	29	9	CDS
mmu-miR-214-3p	NM_001100458	Fam169a	ACAGCAGGCACAGACAGGCAGT#GCTTGCCTGTTATGCCCTGTA#((((..((((..(((((((((#))).)))))).)))))))).	2959	2980	0.89744	-22	0	0.0064014	0.5	0.1	-0.1	-16	18	21	8	3UTR
mmu-miR-214-3p	NM_001100458	Fam169a	ACAGCAGGCACAGACAGGCAGT#GCTGTTTGAGTGCTCTGTCCCGTTGTG#(((((.((.((((((((((((#))))))))).....))))))).))))).	4264	4291	0.92308	-21	0	0.0523754	0.5	0.4	0.52	-7	20	27	8	3UTR
mmu-miR-214-3p	NM_001102430	Arfgef1	ACAGCAGGCACAGACAGGCAGT#ATTGCTCTGCTCTGTGAAAGGATCCTGCA#...(((((((((((((((((#))))).))).))))))).......))))).	5748	5777	0.84615	-24	0	0.0010414	0.5	3.9	2.86	-7	19	29	6	3UTR
mmu-miR-214-3p	NM_001109657	Gas7	ACAGCAGGCACAGACAGGCAGT#GCCTGCATGGGCTGTTGA#.((((((.(.((..(((((...#)))))..))).)))))).	19	37	0.92308	-22	1	1.25E-05	0.5	0.8	1.64	-9	14	18	6	5UTR
mmu-miR-214-3p	NM_001109764	Ctnna2	ACAGCAGGCACAGACAGGCAGT#GCTGACTTGTCTGCCTCTGG#.(((.((((..((((((((((((#)))).))))))))))))))).	3303	3323	1	-23	0	0.0017291	0.6	0	0	-12	18	20	14	3UTR
mmu-miR-214-3p	NM_001110129	Ppih	ACAGCAGGCACAGACAGGCAGT#ACTACCTTGTCTAGCCTGTCTCCAACTCATGGAGGTGCCCTGTCTGCCTCGTGCCTGCTGA#.((((((((((((.(((((((.#.))))))).)).)))	1329	1390	0.92308	-33	1	0.0004693	0.4	0	-0	-5	19	23	10	3UTR

mmu-miR-214-3p	NM_001110130	Ppih	ACAGCAGGCACAGACAGGCAGT#CTGCTCCAGGCCTGCTT#..(((((((.......(((((.#)))))....))))))).	362	379	0.84615	-18	1	0.0087168	0.6	5	3.89	-15	12	17	7	CDS
mmu-miR-214-3p	NM_001110140	Atp2a2	ACAGCAGGCACAGACAGGCAGT#ACTGCTCTCACGTGCCTGGTGG#.((.(((((((.....((((((#)))))).....))))))).)).	1866	1888	0.84615	-24	0	4.73E-05	0.5	4.9	4.28	-13	15	22	7	CDS
mmu-miR-214-3p	NM_001110160	Lrrc61	ACAGCAGGCACAGACAGGCAGT#CTGTTTACGAGGAAGGCTGTGAGCTCTCAGCTTCCTGCTCTGGCCACCATGCCTGCTGG#.((((((((((((((((.(((#.))).)))).))))......	2540	2599	1	-29	1	7.01E-06	0.4	0	0.13	-4	20	31	9	3UTR
mmu-miR-214-3p	NM_001110160	Lrrc61	ACAGCAGGCACAGACAGGCAGT#GCTGCAGTGTCTGGCTGGGTTGC#.((((.(((.((((((.(((((#)))))..)))))))))..)))).	544	567	1	-24	0	0.0001608	0.3	2.8	1.29	-7	18	23	9	CDS
mmu-miR-214-3p	NM_001110209	Lnpk	ACAGCAGGCACAGACAGGCAGT#CTGACCTTTGTGTGCCTGCTGTG#((((((((((((.(.((((((.#))).))).).)))))))))))).	6814	6837	1	-32	1	0.0030709	0.4	1.1	0.96	-6	19	23	12	3UTR
mmu-miR-214-3p	NM_001110209	Lnpk	ACAGCAGGCACAGACAGGCAGT#CTGTCTGTATGTGGAGTGCCTGCTC#..(((((((((..((((((((.#)))))))).......))))))))).	4359	4384	1	-27	1	0.0001787	0.6	-0	0.02	-10	17	25	9	3UTR
mmu-miR-214-3p	NM_001110209	Lnpk	ACAGCAGGCACAGACAGGCAGT#GCCTGAGTTTGTGTCTGCA#...((((((((((((((((...#))))..)))))))))))).	6949	6968	1	-27	0	0.000724	0.5	0.7	0.62	-7	16	19	12	3UTR
mmu-miR-214-3p	NM_001110300	Ap1m2	ACAGCAGGCACAGACAGGCAGT#GCTGCAGACTTTTAAGAGCTGTGTGTGTGTGTTTGTG#...(((((((((.(((.(((((#.))))).))).)	1402	1439	1	-25	0	0.0027594	0.5	3.2	3.34	-8	17	21	9	3UTR
mmu-miR-214-3p	NM_001110300	Ap1m2	ACAGCAGGCACAGACAGGCAGT#GGTGTTTCTGTCTGGGATGCCAGAGCTGC#.((((.(((((((((((((....#.))))))))...))))...)))).	687	716	0.84615	-22	0	1.10E-05	0.4	3.5	3.07	-6	16	24	8	CDS
mmu-miR-214-3p	NM_001110310	Snx12	ACAGCAGGCACAGACAGGCAGT#GCTGTTGTTTGGGAGGTGGTGGTTTGTTTGCTGGGGCCTGCTGTC#(((((((((.(((.((((((.#.))))))))))..))))))))).	1892	1937	0.96154	-27	1	0.0001884	0.5	4.1	4.25	-6	19	23	10	3UTR
mmu-miR-214-3p	NM_001110310	Snx12	ACAGCAGGCACAGACAGGCAGT#ACTGCCTGTACTTGTTTCCTGTTGA#.((((((((.(((((((((((((#)))))))))))).)).))..))))))).	1764	1789	0.84615	-27	1	0.0149073	0.6	2.2	2.69	-5	20	25	9	3UTR
mmu-miR-214-3p	NM_001110315	Kif1a	ACAGCAGGCACAGACAGGCAGT#CTGACCTGTACATATGTGGTTGCTGTG#(((((((.(((((((((((((.#))).)))))).)...)))).))))))).	8060	8087	0.82051	-25	1	0.0012501	0.4	3.7	1.83	-6	20	27	7	3UTR

mmu-miR-214-3p	NM_001110315	Kif1a	ACAGCAGGCACAGACAGGCAGT#GCTGATGAAGTTAACAACAACACCTGTTCAGCTGTGCCTCCTGA#.(((.(((((((((((((...#.)))))....)))))))).))).	3039	3083	0.84615	-24	0	0.0001367	0.5	3.8	2.99	-8	16	23	8	CDS
mmu-miR-214-3p	NM_001110843	Cacna2d1	ACAGCAGGCACAGACAGGCAGT#GCAGGGAGGGCACTGATCTTCGATCGCGAAGATGGCTGCTGGCTGCCTGCTGG#.((((((((((((.(((.((..#.)).))))))..))))))))).	292	345	0.90385	-28	1	4.67E-06	0.4	0.9	0.9	-5	17	22	9	5UTR
mmu-miR-214-3p	NM_001110844	Cacna2d1	ACAGCAGGCACAGACAGGCAGT#GCAGGGAGGGCACTGATCTTCGATCGCGAAGATGGCTGCTGGCTGCCTGCTGG#.((((((((((((.(((.((..#.)).))))))..))))))))).	292	345	0.90385	-28	1	4.67E-06	0.4	0.9	0.9	-5	17	22	9	5UTR
mmu-miR-214-3p	NM_001110845	Cacna2d1	ACAGCAGGCACAGACAGGCAGT#GCAGGGAGGGCACTGATCTTCGATCGCGAAGATGGCTGCTGGCTGCCTGCTGG#.((((((((((((.(((.((..#.)).))))))..))))))))).	292	345	0.90385	-28	1	4.67E-06	0.4	0.9	0.9	-5	17	22	9	5UTR
mmu-miR-214-3p	NM_001110846	Cacna2d1	ACAGCAGGCACAGACAGGCAGT#GCAGGGAGGGCACTGATCTTCGATCGCGAAGATGGCTGCTGGCTGCCTGCTGG#.((((((((((((.(((.((..#.)).))))))..))))))))).	292	345	0.90385	-28	1	4.67E-06	0.4	0.9	0.9	-5	17	22	9	5UTR
mmu-miR-214-3p	NM_001111051	Dclk1	ACAGCAGGCACAGACAGGCAGT#ATTGTCTCTCTGTGTCTGTCTGTC#((((((((((((((.(((((((#))))))).)))))))))).)))).	5275	5299	0.84615	-28	1	0.1847508	0.6	0.1	-0.1	-5	21	24	10	3UTR
mmu-miR-214-3p	NM_001111051	Dclk1	ACAGCAGGCACAGACAGGCAGT#GCTGCTTGCCCATTACCTGCTGC#.(((((((......((((((((#))))))))........))))))).	2362	2385	0.92308	-23	1	0.0017245	0.4	2.4	0.39	-14	15	23	8	3UTR
mmu-miR-214-3p	NM_001111052	Dclk1	ACAGCAGGCACAGACAGGCAGT#ATTGTCTCTCTGTGTCTGTCTGTC#((((((((((((((.(((((((#))))))).)))))))))).)))).	5349	5373	0.84615	-28	1	0.1847508	0.6	0.1	-0.1	-5	21	24	10	3UTR
mmu-miR-214-3p	NM_001111052	Dclk1	ACAGCAGGCACAGACAGGCAGT#GCTGCTTGCCCATTACCTGCTGC#.(((((((......((((((((#))))))))........))))))).	2436	2459	0.92308	-23	1	0.0017245	0.4	2.4	0.39	-14	15	23	8	3UTR
mmu-miR-214-3p	NM_001111053	Dclk1	ACAGCAGGCACAGACAGGCAGT#GCTGCCTTGTCTTTAGCTGAAACAGTAAACATCTGTGAGGATGCTCTGCTGG#.((((((((((((((((((((#))))))))).))))..........................	3246	3298	0.80769	-26	1	0.0001099	0.6	1.8	1.88	-4	21	52	7	3UTR

mmu-miR-214-3p	NM_001111053	Dclk1	ACAGCAGGCACAGACAGGCAGT#ACTGTCTGTGTACATTTGTGCATTTGTGCCTATCTGC#.(((.(((((((((((((((((#))))))))))..............)))))))..))).	5292	5329	0.92308	-24	0	0.0020753	0.5	0.4	0.64	-8	20	37	9	3UTR
mmu-miR-214-3p	NM_001111060	Cd59a	ACAGCAGGCACAGACAGGCAGT#GCTGCTTCTGGCTGTGTTCTGTTC#..(((((((((((.((((((((#)))))..))).)))))).))))).	297	321	0.92308	-23	1	0.0023364	0.5	0	-0	-6	19	24	6	CDS
mmu-miR-214-3p	NM_001111060	Cd59a	ACAGCAGGCACAGACAGGCAGT#GGTGTCTGTGAAGCCTGTGGAAACTGCTGC#.((((((.((((((((((((.(#).))))))))))).....)))))....))	134	164	0.92308	-22	1	0.0015751	0.5	-0	-0.1	-6	19	30	7	5UTR
mmu-miR-214-3p	NM_001112722	Arhgef10l	ACAGCAGGCACAGACAGGCAGT#GGTGCCCCATCCTGGCCTGCTGC#.((((((((.(((...((((.(#).)))).....))))))))))).	2405	2428	0.92308	-25	1	0.0002516	0.3	2	2.07	-7	16	23	11	CDS
mmu-miR-214-3p	NM_001112723	Arhgef10l	ACAGCAGGCACAGACAGGCAGT#GGTGCCCCATCCTGGCCTGCTGC#.((((((((.(((...((((.(#).)))).....))))))))))).	2363	2386	0.92308	-25	1	0.0002516	0.3	3.2	2.8	-7	16	23	11	CDS
mmu-miR-214-3p	NM_001112796	Bicd1	ACAGCAGGCACAGACAGGCAGT#TGTGTGTGTGTGTGTGTGTGTG#((((((.(((((.(((.(((..#))).))).))))).))).))).	3814	3836	0.84615	-22	0	0.4884675	0.5	0.2	0.89	-7	17	22	5	3UTR
mmu-miR-214-3p	NM_001112798	Slc8a1	ACAGCAGGCACAGACAGGCAGT#TGTCTGTCTGTCCTCTGG#.(((.(((.(((((((((((..#))))))))))))))))).	15101	####	1	-26	0	0.0009438	0.6	0.6	0.37	-9	17	18	17	3UTR
mmu-miR-214-3p	NM_001112798	Slc8a1	ACAGCAGGCACAGACAGGCAGT#ACTGCCGTTGTGTTTGTTGC#.(((((((((((.((.((((((#))))))))))))))))))).	2570	2590	1	-25	1	0.0018778	0.5	0.3	0.26	-5	19	20	19	CDS
mmu-miR-214-3p	NM_001112798	Slc8a1	ACAGCAGGCACAGACAGGCAGT#TTGTCTGTTGTGTGATGCTGTG#((((((.(((((.((((((((.#)))))))))))))..)))))).	6203	6225	0.96154	-25	0	0.0037416	0.5	-0	-0	-7	19	22	13	3UTR
mmu-miR-214-3p	NM_001113360	Plch2	ACAGCAGGCACAGACAGGCAGT#GCTGCCGCTGTGTGGAGGTGGACTGCTGG#.((((((((((((.(.((((((#)))))))))))))))..........))))	1359	1388	1	-26	1	0.0021455	0.3	2.5	2.37	-5	19	29	13	CDS
mmu-miR-214-3p	NM_001113360	Plch2	ACAGCAGGCACAGACAGGCAGT#GCTGCCCGTCCTGACCTGCCTCCTGTG#((((.((((((((((.((((((#)))))).))).))...))))).)))).	3860	3887	0.92308	-26	0	0.0287406	0.4	2.7	2.94	-8	20	27	6	CDS
mmu-miR-214-3p	NM_001113391	Cd247	ACAGCAGGCACAGACAGGCAGT#GCTGCCTCTGCCTCTGCCTCTGG#.(((.(((((((((..((((((#))))))))))....)))))))).	102	125	0.84615	-23	0	1.38E-05	0.4	0.2	0.12	-9	18	23	10	5UTR

mmu-miR-214-3p	NM_001113391	Cd247	ACAGCAGGCACAGACAGGCAGT#AGTGGAAAGTGTCTGTTCTCGCCTGCA#...((((((...((((((((.(#.).))))))))...)))))).	152	179	0.84615	-23	0	0.0001108	0.4	0.2	0.36	-14	15	21	8	CDS
mmu-miR-214-3p	NM_001113392	Cd247	ACAGCAGGCACAGACAGGCAGT#GCTGCCTCTGCCTCTGCCTCTGG#.(((.(((((((((..((((((#))))))))))....)))))))).	102	125	0.84615	-23	0	1.38E-05	0.4	0.2	0.12	-9	18	23	10	5UTR
mmu-miR-214-3p	NM_001113392	Cd247	ACAGCAGGCACAGACAGGCAGT#GGTTGTAACCTGTGCCTGG#....((((((((((((.(...#).))))...))))))))).	1650	1669	0.84615	-21	0	7.95E-06	0.4	0.1	0.44	-10	14	19	9	3UTR
mmu-miR-214-3p	NM_001113393	Cd247	ACAGCAGGCACAGACAGGCAGT#GCTGCCTCTGCCTCTGCCTCTGG#.(((.(((((((((..((((((#))))))))))....)))))))).	102	125	0.84615	-23	0	1.38E-05	0.4	0.2	0.12	-9	18	23	10	5UTR
mmu-miR-214-3p	NM_001113393	Cd247	ACAGCAGGCACAGACAGGCAGT#GGTTGTAACCTGTGCCTGG#....((((((((((((.(...#).))))...))))))))).	1741	1760	0.84615	-21	0	7.95E-06	0.4	0.1	0.44	-10	14	19	9	3UTR
mmu-miR-214-3p	NM_001113417	Thrb	ACAGCAGGCACAGACAGGCAGT#GCTGCCTGAGTTCTACTTTGTACTCTGCAGCCTGG#....(((((.((((((((((((#))))))))).............)))).))))).	3201	3236	0.92308	-24	0	4.30E-05	0.6	0.2	0.3	-11	17	35	8	3UTR
mmu-miR-214-3p	NM_001113417	Thrb	ACAGCAGGCACAGACAGGCAGT#GCCTGTACCTGGCCTCCTGC#.(((.((((.((((((((...#))))))..))))))).))).	3636	3656	0.92308	-24	0	4.63E-05	0.5	0.3	-0.2	-9	16	20	7	3UTR
mmu-miR-214-3p	NM_001113417	Thrb	ACAGCAGGCACAGACAGGCAGT#GCTGGTAGGAATGTCTGAAGCCTGCCTGCA#...((((((((((((((.((((#)))).).....))))))).....)))))	272	302	0.92308	-23	0	0.0001919	0.4	1.6	1.26	-7	18	30	7	CDS
mmu-miR-214-3p	NM_001113529	Csf1	ACAGCAGGCACAGACAGGCAGT#CTGCCTGCTGAACAGCCTGTC#...((((((.(((.(((((((.#))))))))))....)))))).	1986	2007	0.92308	-25	0	0.0183691	0.5	0.9	0.63	-9	16	21	10	3UTR
mmu-miR-214-3p	NM_001113529	Csf1	ACAGCAGGCACAGACAGGCAGT#TGTCTGTGTGTGTCTGGCGG#.(..((((((((.(((((((..#))))))).))))))))..).	129	149	0.92308	-26	0	0.0003268	0.5	1.5	1.59	-10	16	20	8	5UTR
mmu-miR-214-3p	NM_001113529	Csf1	ACAGCAGGCACAGACAGGCAGT#GTCTGTCTTCCACCTGCTGG#.(((((((...((((((((...#))))))))....))))))).	996	1016	0.92308	-26	1	0.0013958	0.4	-0	0.56	-9	15	20	8	CDS
mmu-miR-214-3p	NM_001113530	Csf1	ACAGCAGGCACAGACAGGCAGT#AGTGCCTGTGAGTGTGTTTGTGTGTGTGTATGTCTGTGTGTGTCTGG#....((((((((.((((((((..#.)))))))).)))))))).	99	146	0.92308	-25	0	0.0134762	0.5	0.6	0.54	-11	15	18	8	5UTR

mmu-miR-214-3p	NM_001113530	Csf1	ACAGCAGGCACAGACAGGCAGT#GTCTGTCTTCCACCTGCTGG#.(((((((...((((((((...#))))))))....))))))).	1881	1901	0.92308	-26	1	0.0002574	0.4	0.6	1.14	-9	15	20	8	CDS
mmu-miR-214-3p	NM_001113560	Glo1	ACAGCAGGCACAGACAGGCAGT#ATTGCCGTTCCTGATGTCTACAGTGCCTGTA#...((((((((((((((((((#))))))........)))))...)))	495	526	1	-24	0	0.0002171	0.6	3.4	3.26	-7	19	31	8	CDS
mmu-miR-214-3p	NM_001113569	Stxbp1	ACAGCAGGCACAGACAGGCAGT#CCTGATCTGTGTCCATACTGTTGTA#((((((((((((((((....#)))).))))))).....))))))).	2674	2699	0.92308	-23	1	0.0176683	0.5	4.4	4.2	-4	18	25	7	3UTR
mmu-miR-214-3p	NM_001114339	Pank1	ACAGCAGGCACAGACAGGCAGT#CTGGCTGTCTCGTGCCTA#.....(((((((((((.(((.#))).)))))).)))))).	167	185	0.92308	-26	0	5.35E-05	0.4	0.2	0.37	-14	15	18	6	5UTR
mmu-miR-214-3p	NM_001114339	Pank1	ACAGCAGGCACAGACAGGCAGT#TGTCATCTGTGTTTGTTGTT#(((((((((((((..((((..#)))).))))))))))))).	4951	4971	1	-23	1	0.178895	0.8	3.3	2.64	-5	18	20	14	3UTR
mmu-miR-214-3p	NM_001114339	Pank1	ACAGCAGGCACAGACAGGCAGT#GCCTATGTTGCTTGCTGA#.((((((((((...((((...#)))).))).)))))))).	1562	1580	0.94872	-22	1	0.0095672	0.5	0	-0.1	-7	15	18	8	CDS
mmu-miR-214-3p	NM_001114339	Pank1	ACAGCAGGCACAGACAGGCAGT#GCCACTATGGTGGATGAACCTGCTGTA#((((((((..((..(((((...#)))).......))..))..)))))))).	2103	2130	0.84615	-21	1	0.0093265	0.6	-0	-0.2	-8	15	27	8	3UTR
mmu-miR-214-3p	NM_001114609	Ino80d	ACAGCAGGCACAGACAGGCAGT#ACTGCCTGTGCCTGCTTGTTCACCCTGTGTGTGTTGTG#((((((.((((((((((((((#))))))))))..............)))))).)))))).	3902	3940	1	-30	0	0.0100131	0.5	1.4	1.98	-5	21	38	9	3UTR
mmu-miR-214-3p	NM_001122667	Mrtfb	ACAGCAGGCACAGACAGGCAGT#GCTTGTAACTGCCTGCTC#..((((((((...((((((...#))))))...)))))))).	4407	4425	1	-24	1	0.0056026	0.6	1.3	0.44	-12	14	18	8	3UTR
mmu-miR-214-3p	NM_001122667	Mrtfb	ACAGCAGGCACAGACAGGCAGT#ACTGTGTCCTCAGCGAAGTCTGGCCCAATGCTGG#.((((((((.((((((.(((((#))))).)..........))))))))...))))).	963	997	0.84615	-22	1	6.44E-05	0.5	2.7	2.34	-5	19	34	8	CDS
mmu-miR-214-3p	NM_001123327	Qser1	ACAGCAGGCACAGACAGGCAGT#CTGCCTGTGGCAACTCCTGGTGCATCTGCTGA#.((((((((((..((((((((.#))))))))...........)))).	3893	3925	0.92308	-27	1	0.000227	0.4	2.3	3.12	-8	18	32	8	CDS
mmu-miR-214-3p	NM_001123327	Qser1	ACAGCAGGCACAGACAGGCAGT#CTGCTCAGCCCCAAGAGTTTCTGCCTGCTGTG#((((((((((.((((.(((((.#)))))...........)))).)))	1632	1664	0.84615	-25	1	9.65E-06	0.5	0.1	0.34	-7	19	32	10	CDS

mmu-miR-214-3p	NM_001123327	Qser1	ACAGCAGGCACAGACAGGCAGT#TTGTGTGTTTGTGTCCATCGCTGA#.(((((.(((((((((((.((((.#)))).)))))))).))...))))).	8499	8523	0.84615	-24	0	0.0017969	0.6	2.4	3.37	-6	18	24	8	3UTR
mmu-miR-214-3p	NM_001127324	Ercc1	ACAGCAGGCACAGACAGGCAGT#AGTGCCATCCTGATCCTGCTGTG#((((((((..(((...((((.(#).))))...)))..)))))))).	1133	1156	0.84615	-23	1	0.0039642	0.4	-0	-0.5	-8	16	23	8	3UTR
mmu-miR-214-3p	NM_001127324	Ercc1	ACAGCAGGCACAGACAGGCAGT#ACTTCGCCCTTCGTGTGCTGCTGG#.(((((.(((((((..((((((#)))..)))..)).))))))))))).	620	644	1	-22	0	0.0007437	0.4	2.1	3.19	-6	18	24	10	CDS
mmu-miR-214-3p	NM_001127324	Ercc1	ACAGCAGGCACAGACAGGCAGT#GCTGGCTGTGCTGGCGTTGTGTCGCCTCTGTT#((((.((((.(((((((.((((#)))).)))).)))..........)	53	85	0.92308	-21	0	0.0268387	0.4	-0	0.07	-9	19	32	8	5UTR
mmu-miR-214-3p	NM_001128080	Tspan32	ACAGCAGGCACAGACAGGCAGT#GCTTCCATCCTGACCTGCACCAGCCTGGCCCTCACGGTATATGCTATGATGCTCTGTGCCTTCCTGTG#((((.((((((((((((((...#.))))).......................)))))))))..)))).	655	723	0.80769	-25	0	0.0034939	0.5	0.4	0.75	-8	18	47	9	CDS
mmu-miR-214-3p	NM_001128080	Tspan32	ACAGCAGGCACAGACAGGCAGT#GCTGCCTGCTTGCCC#......((((.(((.((((((((#)))))))))))))).	942	957	0.92308	-22	0	0.0020227	0.5	0	-0.2	-19	14	15	14	3UTR
mmu-miR-214-3p	NM_001128081	Tspan32	ACAGCAGGCACAGACAGGCAGT#CTGTGTGTAGTACACTATCTGTGAGTGCCTGCC#...((((((((..(((((.(((#.))).)))))..))))))))	51	84	0.84615	-25	0	0.0012969	0.5	-0	0.11	-11	16	21	8	5UTR
mmu-miR-214-3p	NM_001128081	Tspan32	ACAGCAGGCACAGACAGGCAGT#TGCTATGATGCTCTGTGCCTTCCTGTG#((((.(((((((((((((((..#))))....)).)))))))))..)))).	727	754	0.92308	-23	0	0.0034189	0.5	0.4	0.25	-8	19	27	9	CDS
mmu-miR-214-3p	NM_001128082	Tspan32	ACAGCAGGCACAGACAGGCAGT#CTGTGTGTAGTACACTATCTGTGAGTGCCTGCC#...((((((((..(((((.(((#.))).)))))..))))))))	51	84	0.84615	-25	0	0.0012969	0.5	-0	0.11	-11	16	21	8	5UTR
mmu-miR-214-3p	NM_001128082	Tspan32	ACAGCAGGCACAGACAGGCAGT#TGCTATGATGCTCTGTGCCTTCCTGTG#((((.(((((((((((((((..#))))....)).)))))))))..)))).	625	652	0.92308	-23	0	0.0034189	0.5	0.4	0.25	-8	19	27	9	CDS
mmu-miR-214-3p	NM_001128180	Dlgap1	ACAGCAGGCACAGACAGGCAGT#CTGCATCTGTGCCCTCCCTCGCAGCTGTA#(((((.((((((((...((((.#)))).))))))))..........))))).	2704	2733	1	-23	0	0.0030793	0.4	0.3	1	-7	17	29	8	3UTR
mmu-miR-214-3p	NM_001128181	Dlgap1	ACAGCAGGCACAGACAGGCAGT#GCTGCTGCATTCAGTCCTGCTGTT#((((((((.((.((..((((((#))))))....)).)))))))))))).	1985	2009	0.92308	-23	1	0.0018917	0.5	1.5	2.67	-9	18	24	10	CDS

mmu-miR-214-3p	NM_001128307	Dock9	ACAGCAGGCACAGACAGGCAGT#TGAACCCGGGCAGTATCGTGAAGGACGAAGCCCTGGCTGTGCCTGCTGG#.((((((((((((.((((....#.)))).)))))))))))).	4062	4111	1	-32	1	4.96E-05	0.4	0	0	-5	16	19	12	CDS
mmu-miR-214-3p	NM_001128308	Dock9	ACAGCAGGCACAGACAGGCAGT#TGAACCCGGGCAGTATCGTGAAGGACGAAGCCCTGGCTGTGCCTGCTGG#.((((((((((((.((((....#.)))).)))))))))))).	4062	4111	0.92308	-32	1	4.96E-05	0.4	1.3	2.16	-5	16	19	12	CDS
mmu-miR-214-3p	NM_001130163	Oxr1	ACAGCAGGCACAGACAGGCAGT#GTCTGTTTGGCCATTTACTGCA#...((((((.(((((((((...#))))))))))))......))).	2517	2539	0.92308	-20	0	0.0177324	0.7	0.7	1.67	-8	15	22	12	3UTR
mmu-miR-214-3p	NM_001130163	Oxr1	ACAGCAGGCACAGACAGGCAGT#CCTCGTCTGTGTAAAGCTC#..(((..(((((((((((....#))).))))))))...))).	3924	3943	0.97436	-18	0	0.0046943	0.6	1.4	1.54	-8	14	19	8	3UTR
mmu-miR-214-3p	NM_001130164	Oxr1	ACAGCAGGCACAGACAGGCAGT#GCTTTAAAGTCAGTCTGTTTGGCCATTTACTGCA#...((((((.(((((((((...#.))))))))))))......))	858	892	0.92308	-20	0	0.0177324	0.7	1.2	1.76	-8	15	23	12	3UTR
mmu-miR-214-3p	NM_001130164	Oxr1	ACAGCAGGCACAGACAGGCAGT#CCTCGTCTGTGTAAAGCTC#..(((..(((((((((((....#))).))))))))...))).	2277	2296	0.97436	-18	0	0.0046943	0.6	1.4	1.54	-8	14	19	8	3UTR
mmu-miR-214-3p	NM_001130165	Oxr1	ACAGCAGGCACAGACAGGCAGT#GTCTGTTTGGCCATTTACTGCA#...((((((.(((((((((...#))))))))))))......))).	2705	2727	0.92308	-20	0	0.0177324	0.7	0.7	1.67	-8	15	22	12	3UTR
mmu-miR-214-3p	NM_001130165	Oxr1	ACAGCAGGCACAGACAGGCAGT#CCTCGTCTGTGTAAAGCTC#..(((..(((((((((((....#))).))))))))...))).	4112	4131	0.97436	-18	0	0.0046943	0.6	1.4	1.54	-8	14	19	8	3UTR
mmu-miR-214-3p	NM_001130166	Oxr1	ACAGCAGGCACAGACAGGCAGT#CTGCCTGTTCCAAGGGCGGAGCAGAAGCAGGCCGTGGTCCTGCC#...(((((..(((((((((((.#)))))))))).......................))..))))).	139	183	0.92308	-22	0	8.90E-06	0.4	0.1	0.42	-9	16	44	9	5UTR
mmu-miR-214-3p	NM_001130166	Oxr1	ACAGCAGGCACAGACAGGCAGT#GTCTGTTTGGCCATTTACTGCA#...((((((.(((((((((...#))))))))))))......))).	2957	2979	0.92308	-20	0	0.0177324	0.7	0.7	1.67	-8	15	22	12	3UTR
mmu-miR-214-3p	NM_001130458	Tcn2	ACAGCAGGCACAGACAGGCAGT#ACTGCCAGGCATCCAGGGTCATGTTGGTGCCAGCTGTT#(((((.(((((.((((((((((#))))))...............)))).))))).))))).	1128	1166	0.84615	-27	0	0.0006268	0.4	1.8	0.94	-9	20	38	6	CDS

mmu-miR-214-3p	NM_001130458	Tcn2	ACAGCAGGCACAGACAGGCAGT#ATGGCCTGCTGTGGCTCTGA#.(((.((.(((((.(((((.((#)).)))))))))).))))).	1812	1832	0.84615	-23	0	1.90E-05	0.4	1	0.84	-11	17	20	10	3UTR
mmu-miR-214-3p	NM_001130459	Tcn2	ACAGCAGGCACAGACAGGCAGT#GTCATGTTGGTGCCAGCTGTT#(((((.(((((.(((((((...#))).)))).))))).))))).	1114	1135	0.84615	-24	0	0.0006268	0.4	2.2	1.05	-9	17	21	5	CDS
mmu-miR-214-3p	NM_001130459	Tcn2	ACAGCAGGCACAGACAGGCAGT#ATGGCCTGCTGTGGCTCTGA#.(((.((.(((((.(((((.((#)).)))))))))).))))).	1781	1801	0.84615	-23	0	1.90E-05	0.4	0.9	0.88	-11	17	20	10	3UTR
mmu-miR-214-3p	NM_001134717	2810006K23Rik	ACAGCAGGCACAGACAGGCAGT#AGTGCCACTGTCTGGCCTTCA#...(.((((.((((((((((.(#).))))..))))))))).).	1042	1063	0.92308	-21	0	0.0002334	0.5	0	-0	-12	16	21	10	3UTR
mmu-miR-214-3p	NM_001135559	Sos2	ACAGCAGGCACAGACAGGCAGT#CTGCACTATCGCAGCACTCTAGACAGAATGCTGGACTCTGTGCTGCTGA#.(((((.(((((((....((((#.))))...)))))))))))).	1874	1923	1	-23	0	1.97E-06	0.5	1.7	3.12	-6	16	21	12	CDS
mmu-miR-214-3p	NM_001135657	Ptprj	ACAGCAGGCACAGACAGGCAGT#GCTGAACGATTTTGGATGTTGGGTTCTAGGTCCTGGCTGTTGCTGGTCTGCTA#..((((((.((((((.(((.#.))).))))..)))))))))).	3825	3878	1	-24	1	4.86E-05	0.5	-1	-0.5	-6	17	22	10	CDS
mmu-miR-214-3p	NM_001135657	Ptprj	ACAGCAGGCACAGACAGGCAGT#GCTGGCCCAGGGTGCAGCTGCCTCTGCTGTT#(((((((........(((((((#.)))))))))))))).	102	133	0.88462	-20	1	3.14E-06	0.5	0.4	0.06	-18	14	16	14	CDS
mmu-miR-214-3p	NM_001136073	Nfatc2	ACAGCAGGCACAGACAGGCAGT#GCTGCCCTCTACCAGAGAAGCAAGAGCCTGAGTCCCGGCCTGCTGG#.((((((((...(((((((...#.))))..)))...)))))))).	2424	2470	1	-26	1	2.90E-06	0.4	0.5	0.02	-11	15	22	8	CDS
mmu-miR-214-3p	NM_001136104	Abl2	ACAGCAGGCACAGACAGGCAGT#ACTGCTGAGTCTCTTGGCTGCCTGCTT#..((((((((((((.((((((#))))))..))))....).)))))))).	5682	5709	1	-25	1	0.0025318	0.5	1	0.64	-5	19	27	8	3UTR
mmu-miR-214-3p	NM_001136104	Abl2	ACAGCAGGCACAGACAGGCAGT#GCTTGTTGGTGGCTGTCTGTA#(((((((.(((.(((((((...#)))))))).))).))).))))).	4239	4260	0.92308	-25	1	9.60E-06	0.4	0.6	0.21	-10	17	21	7	3UTR
mmu-miR-214-3p	NM_001136104	Abl2	ACAGCAGGCACAGACAGGCAGT#GCTGCTTGTACCTTTCCCTCCTTGCAGCCCTCTGGCTGGCTGTA#(((((.(((.(((((((((((((#))))))))...................)))))).))))).	4460	4504	0.92308	-24	0	8.40E-05	0.4	-0	-0.3	-7	20	44	8	3UTR

mmu-miR-214-3p	NM_001136104	Abl2	ACAGCAGGCACAGACAGGCAGT#GCTGCTGGTGTGCCTGG#....((((((((....((((((#))))))..)))))))).	3415	3432	0.84615	-24	0	2.99E-06	0.5	1	3.56	-13	14	17	8	CDS
mmu-miR-214-3p	NM_001136484	Gcnt1	ACAGCAGGCACAGACAGGCAGT#TGCCGAGGCTGTTGGAAAGCCTGCTGG#.((((((((((((...((((..#))))....))))......)))))))).	31	58	1	-26	1	0.0001269	0.4	0.2	0.49	-6	16	27	8	5UTR
mmu-miR-214-3p	NM_001142580	Vipas39	ACAGCAGGCACAGACAGGCAGT#CTTAGGAGAGTCACAGCATTCTACACGTGAGCCTGTCATTCACAGGCAGTGCCTGCTGG#.((((((((((.(((((((...#.)))))))..........))	1683	1742	0.92308	-32	1	8.74E-08	0.5	2.6	2.79	-7	17	30	10	3UTR
mmu-miR-214-3p	NM_001142581	Vipas39	ACAGCAGGCACAGACAGGCAGT#CTTAGGAGAGTCACAGCATTCTACACGTGAGCCTGTCATTCACAGGCAGTGCCTGCTGG#.((((((((((.(((((((...#.)))))))..........))	1626	1685	0.92308	-32	1	8.74E-08	0.5	3.1	2.67	-7	17	30	10	3UTR
mmu-miR-214-3p	NM_001142681	Chid1	ACAGCAGGCACAGACAGGCAGT#ACTACTCACTGCTGCCTGCTGTG#(((((((((((((..((..(((#))).))..))).)))))))))).	1437	1460	0.92308	-23	1	6.57E-06	0.4	0.2	0.03	-6	18	23	10	3UTR
mmu-miR-214-3p	NM_001142681	Chid1	ACAGCAGGCACAGACAGGCAGT#GCCTGGATTACTTCTATGACCTGCTGTA#((((((((((.((((((((...#)))))).......))).)).)))))))).	1253	1281	0.92308	-25	1	0.0032276	0.4	0.2	0.09	-7	18	28	8	CDS
mmu-miR-214-3p	NM_001142681	Chid1	ACAGCAGGCACAGACAGGCAGT#GCTTGCTCTGGTCTGTGGCTCTGTT#((((.((.((((((((((((((#))).)).))).)))))).)))))).	126	151	0.84615	-24	0	0.0008687	0.5	-1	-0.3	-10	20	25	6	CDS
mmu-miR-214-3p	NM_001145198	1500009L16Rik	ACAGCAGGCACAGACAGGCAGT#ATTCCTGCTGTGCAACCTCGCCCGAATCCTAGCTGTG#(((((((((((((.((((.(((#))))))))))))))).............)).)))))).	425	462	0.92308	-20	1	0.0136856	0.5	-0	0.42	-5	20	37	13	3UTR
mmu-miR-214-3p	NM_001145198	1500009L16Rik	ACAGCAGGCACAGACAGGCAGT#AGTGTATGTTGGCCTGCC#...((((((.((.((((.(((.(#).))).)))))))))))).	243	261	0.84615	-17	0	0.000211	0.5	1.4	1.63	-9	15	18	11	CDS
mmu-miR-214-3p	NM_001145209	Hbs1l	ACAGCAGGCACAGACAGGCAGT#GCTCCTAACCTGTCTGGCCTGTG#...((((((.((((((((....#.)))))))))))))))).	3964	3987	1	-26	0	0.0007631	0.5	-0	0.14	-8	14	16	14	3UTR
mmu-miR-214-3p	NM_001145209	Hbs1l	ACAGCAGGCACAGACAGGCAGT#TGTGTGTGTGTGCGTGCA#...(((.(((((.(((.((((..#))).))).))))).))).	3247	3265	0.92308	-25	0	0.0002757	0.5	0.4	0.34	-9	14	18	5	3UTR

mmu-miR-214-3p	NM_001145209	Hbs1l	ACAGCAGGCACAGACAGGCAGT#TGTGTGTGTGTGTCTCTGTG#((((.(((((((.(((.(((..#))).))).))))))))))).	3267	3287	1	-24	0	0.0028442	0.6	0.2	0.18	-9	17	20	11	3UTR
mmu-miR-214-3p	NM_001145802	Mak	ACAGCAGGCACAGACAGGCAGT#CTGCCCGTCTCGTTTTAGTCCTGTTGTT#((((((((((((((.(((((.#))))).)))).))....).)))))))).	2581	2609	1	-24	1	0.0220696	0.5	4	4.37	-4	20	28	8	3UTR
mmu-miR-214-3p	NM_001145802	Mak	ACAGCAGGCACAGACAGGCAGT#GCCTGTCTCCAGTGTAGCA#...((..(((((((((((...#))))))))...)))).)).	208	227	1	-21	0	0.0001661	0.6	0.5	0.85	-10	14	19	8	5UTR
mmu-miR-214-3p	NM_001145803	Mak	ACAGCAGGCACAGACAGGCAGT#CTGCCCGTCTCGTTTTAGTCCTGTTGTT#((((((((((((((.(((((.#))))).)))).))....).)))))))).	2869	2897	1	-24	1	0.0220696	0.5	4.1	4.23	-4	20	28	8	3UTR
mmu-miR-214-3p	NM_001145803	Mak	ACAGCAGGCACAGACAGGCAGT#GCCTGTCTCCAGTGTAGCA#...((..(((((((((((...#))))))))...)))).)).	208	227	1	-21	0	0.0001661	0.6	0.5	0.85	-10	14	19	8	5UTR
mmu-miR-214-3p	NM_001145835	Ralgds	ACAGCAGGCACAGACAGGCAGT#ACACCCTGTCGTGTGAGCTGG#.((((..((((.((((((..((#))..))))))))))..)))).	1981	2002	0.84615	-23	0	2.80E-05	0.4	1.8	2.59	-11	16	21	10	CDS
mmu-miR-214-3p	NM_001145836	Ralgds	ACAGCAGGCACAGACAGGCAGT#ACACCCTGTCGTGTGAGCTGG#.((((..((((.((((((..((#))..))))))))))..)))).	1945	1966	0.84615	-23	0	2.80E-05	0.4	1.8	2.59	-11	16	21	10	CDS
mmu-miR-214-3p	NM_001145935	Zfp691	ACAGCAGGCACAGACAGGCAGT#GCCTTTAGTACCTGCTGTG#((((((((.((....((((...#))))...)).)))))))).	1115	1134	1	-22	1	9.30E-05	0.5	2.4	2.46	-11	14	19	8	3UTR
mmu-miR-214-3p	NM_001145936	Zfp691	ACAGCAGGCACAGACAGGCAGT#GCCTTTAGTACCTGCTGTG#((((((((.((....((((...#))))...)).)))))))).	979	998	0.92308	-22	1	9.30E-05	0.5	4.5	1.75	-11	14	19	8	3UTR
mmu-miR-214-3p	NM_001145952	Lpp	ACAGCAGGCACAGACAGGCAGT#CTGCCTCCCTTTAACCTCTCTCTCTACTGTGCCAACTAATGCTGC#.((((((((((((..((((((.#))))))....................)))))))......))))).	6747	6792	0.84615	-24	1	0.058855	0.6	-0	-0.3	-5	18	45	7	3UTR
mmu-miR-214-3p	NM_001145952	Lpp	ACAGCAGGCACAGACAGGCAGT#ACTTTGGCCGTTGTGCCCGCTGTG#(((((.((((((.(((.(((...#.)))))))))))).))))).	1580	1604	1	-25	0	0.000118	0.5	4	3.66	-7	16	19	11	CDS
mmu-miR-214-3p	NM_001145954	Lpp	ACAGCAGGCACAGACAGGCAGT#CTGCCTCCCTTTAACCTCTCTCTCTACTGTGCCAACTAATGCTGC#.((((((((((((..((((((.#))))))....................)))))))......))))).	6161	6206	0.84615	-24	1	0.058855	0.6	-0	-0.3	-5	18	45	7	3UTR

mmu-miR-214-3p	NM_001145954	Lpp	ACAGCAGGCACAGACAGGCAGT#ACTTTGGCCGTTGTGCCCGCTGTG#(((((.((((((.((.(((...#.))))))))))).))))))).	994	1018	1	-25	0	0.000118	0.5	4	3.66	-7	16	19	11	CDS
mmu-miR-214-3p	NM_001145999	Zfp710	ACAGCAGGCACAGACAGGCAGT#GCTGCCATGTCTGTGTCGAATGCG#...(((((((((((((((((#)))))).))))))))))...))).	1420	1444	0.84615	-30	0	4.49E-06	0.4	3.5	3.89	-7	19	24	10	CDS
mmu-miR-214-3p	NM_001145999	Zfp710	ACAGCAGGCACAGACAGGCAGT#ATGAGGTGTCTGTGCCTGGTGA#.((.((((((((((((......#.)))))))))))).)).	568	590	1	-27	0	3.92E-06	0.4	0.4	0.62	-7	14	17	12	CDS
mmu-miR-214-3p	NM_001146000	Zfp710	ACAGCAGGCACAGACAGGCAGT#GCTGCCATGTCTGTGTCGAATGCG#...(((((((((((((((((#)))))).))))))))))...))).	1420	1444	0.84615	-30	0	4.49E-06	0.4	3.5	3.89	-7	19	24	10	CDS
mmu-miR-214-3p	NM_001146000	Zfp710	ACAGCAGGCACAGACAGGCAGT#ATGAGGTGTCTGTGCCTGGTGA#.((.((((((((((((......#.)))))))))))).)).	568	590	1	-27	0	3.92E-06	0.4	0.4	0.62	-7	14	17	12	CDS
mmu-miR-214-3p	NM_001146045	Fam169a	ACAGCAGGCACAGACAGGCAGT#GCTTGCCTGTTATGCCCTGTA#((((..((((..((((((((((#))).))))))).)))))))).	2926	2947	0.89744	-22	0	0.0064014	0.5	0.1	-0.1	-16	18	21	8	3UTR
mmu-miR-214-3p	NM_001146045	Fam169a	ACAGCAGGCACAGACAGGCAGT#GCTGTTTGAGTGCTCTGTCCCGTTGTG#(((((.((.(((((((((((((#))))))))).....))))))).))))).	4231	4258	0.92308	-21	0	0.0523754	0.5	0.4	0.52	-7	20	27	8	3UTR
mmu-miR-214-3p	NM_001146048	Lrrc1	ACAGCAGGCACAGACAGGCAGT#ATTGACCTCCGAGTTTTACCTGCTGTGTCTTCCCTGCTGTA#((((((((...((((((((((.#.)))))).))))))..)))))))).	1805	1846	1	-26	1	0.0067684	0.5	2.8	2.94	-9	18	23	8	3UTR
mmu-miR-214-3p	NM_001146048	Lrrc1	ACAGCAGGCACAGACAGGCAGT#GCAGCATCAGGCTGTGCCTGCTC#..(((((((((((....((.((#)).))......))))))))))).	2638	2661	0.92308	-23	1	4.87E-08	0.4	3.3	4.02	-7	15	23	11	3UTR
mmu-miR-214-3p	NM_001146073	Hexdc	ACAGCAGGCACAGACAGGCAGT#GCTGGCAGAGGTACGACCACTTCTCTGTGCTGTGTGAGCTGC#.((((..((((((((((.(((#.))).).)))).))))))..)))).	938	980	0.84615	-22	0	0.000366	0.4	3.9	1.81	-7	18	25	6	CDS
mmu-miR-214-3p	NM_001159289	Bcl11a	ACAGCAGGCACAGACAGGCAGT#CTGCCGAGTCCTCCCAGCCTGGGCCTGCAGTG#((.((((((.(((((.(((((.#)))))..)))).........)).)))))).)).	1176	1208	0.84615	-23	0	4.79E-05	0.6	2.5	2.25	-7	18	32	6	3UTR

mmu-miR-214-3p	NM_001159290	Bcl11a	ACAGCAGGCACAGACAGGCAGT#CTGCCGAGTCCTCCCAGCCTGGGCCTGCAGTG#((.((((((.(((((.(((((.#)))))..)))..........)).))))))).)).	831	863	0.84615	-23	0	4.79E-05	0.6	2.5	2.25	-7	18	32	6	3UTR
mmu-miR-214-3p	NM_001159317	Il1rap	ACAGCAGGCACAGACAGGCAGT#GCTGTCGCCGAGCCTGCTGG#.((((((((.......((((((#)))))).....)))))))).	148	168	0.84615	-23	1	4.09E-06	0.4	-0	0.48	-14	14	20	8	5UTR
mmu-miR-214-3p	NM_001159317	Il1rap	ACAGCAGGCACAGACAGGCAGT#GCTGAGAATCCTGTCTCCTCTTTCAGTTCCATATGTTGCCTGCTT#..((((((((((((((((((#)))).....)))))))..................)).)))))))).	3422	3467	0.84615	-23	1	0.032035	0.6	0.1	0.11	-5	20	45	7	3UTR
mmu-miR-214-3p	NM_001159318	Il1rap	ACAGCAGGCACAGACAGGCAGT#GCTGTCGCCGAGCCTGCTGG#.((((((((.......((((((#)))))).....)))))))).	148	168	0.84615	-23	1	4.09E-06	0.4	-0	0.48	-14	14	20	8	5UTR
mmu-miR-214-3p	NM_001159318	Il1rap	ACAGCAGGCACAGACAGGCAGT#GCTGAGAATCCTGTCTCCTCTTTCAGTTCCATATGTTGCCTGCTT#..((((((((((((((((((#)))).....)))))))..................)).)))))))).	3711	3756	0.84615	-23	1	0.032035	0.6	0.1	0.11	-5	20	45	7	3UTR
mmu-miR-214-3p	NM_001159485	Mcf2l	ACAGCAGGCACAGACAGGCAGT#GCCTGGAGCCCTGTGCCTGA#....(((((((((.(((((...#))))).....))))))))).	128	148	0.92308	-25	0	5.39E-05	0.4	0.1	0.08	-10	14	20	9	5UTR
mmu-miR-214-3p	NM_001159485	Mcf2l	ACAGCAGGCACAGACAGGCAGT#CCGCTCTGTGCCGCTGA#.((((.((((((((..((....#))..)))))))))))).	280	297	1	-23	0	0.0001901	0.4	2.9	2.26	-7	14	17	12	CDS
mmu-miR-214-3p	NM_001159485	Mcf2l	ACAGCAGGCACAGACAGGCAGT#CTCCCTGTCTCCTCACCCTGCTGC#.(((((((...((((((((.((.#)).))))))))......))))))).	5013	5037	1	-23	1	0.1218334	0.4	-0	0.23	-9	16	24	7	3UTR
mmu-miR-214-3p	NM_001159486	Mcf2l	ACAGCAGGCACAGACAGGCAGT#GCCTGGAGCCCTGTGCCTGA#....(((((((((.(((((...#))))).....))))))))).	128	148	0.92308	-25	0	5.39E-05	0.4	0.1	0.08	-10	14	20	9	5UTR
mmu-miR-214-3p	NM_001159486	Mcf2l	ACAGCAGGCACAGACAGGCAGT#CCGCTCTGTGCCGCTGA#.((((.((((((((..((....#))..)))))))))))).	280	297	1	-23	0	0.0001901	0.4	2.9	2.26	-7	14	17	12	CDS
mmu-miR-214-3p	NM_001159486	Mcf2l	ACAGCAGGCACAGACAGGCAGT#CTCCCTGTCTCCTCACCCTGCTGC#.(((((((...((((((((.((.#)).))))))))......))))))).	4927	4951	1	-23	1	0.1218334	0.4	-0	0.23	-9	16	24	7	3UTR

mmu-miR-214-3p	NM_001159517	Qk	ACAGCAGGCACAGACAGGCAGT#TGACCTGACCATGCCTGCCTGCTGA#.(((((((((((..((((((..#)).))))....))..))))))))).	1552	1577	1	-23	1	0.0054208	0.5	0.6	0.49	-6	17	25	9	3UTR
mmu-miR-214-3p	NM_001159533	Cacna1c	ACAGCAGGCACAGACAGGCAGT#CCTGTCATGTGCCATGCCTGTT#(((((((((((((((((....#)))))).)))))).))).))).	13216	####	0.96154	-26	1	0.0031596	0.5	1.7	1.67	-4	18	22	6	3UTR
mmu-miR-214-3p	NM_001159533	Cacna1c	ACAGCAGGCACAGACAGGCAGT#CCTGCTCTGTGTCTGCC#...(((((((((((((((....#)))).))))))))))).	7763	7780	0.92308	-26	0	0.0055044	0.6	4.6	2.59	-7	15	17	11	3UTR
mmu-miR-214-3p	NM_001159533	Cacna1c	ACAGCAGGCACAGACAGGCAGT#CTGCCTGCTGCGCCTTCTC#..((.((((.(((.((((((.#)))))))))).)))).)).	13390	####	0.92308	-26	0	1.86E-05	0.4	0.7	0.84	-11	16	19	10	3UTR
mmu-miR-214-3p	NM_001159534	Cacna1c	ACAGCAGGCACAGACAGGCAGT#CCTGTCATGTGCCATGCCTGTT#(((((((((((((((((....#)))))).)))))).))).))).	13216	####	0.96154	-26	1	0.0031596	0.5	1.7	1.67	-4	18	22	6	3UTR
mmu-miR-214-3p	NM_001159534	Cacna1c	ACAGCAGGCACAGACAGGCAGT#CCTGCTCTGTGTCTGCC#...(((((((((((((((....#)))).))))))))))).	7763	7780	0.92308	-26	0	0.0055044	0.6	4.6	2.59	-7	15	17	11	3UTR
mmu-miR-214-3p	NM_001159534	Cacna1c	ACAGCAGGCACAGACAGGCAGT#CTGCCTGCTGCGCCTTCTC#..((.((((.(((.((((((.#)))))))))).)))).)).	13390	####	0.92308	-26	0	1.86E-05	0.4	0.7	0.84	-11	16	19	10	3UTR
mmu-miR-214-3p	NM_001159561	P2rx6	ACAGCAGGCACAGACAGGCAGT#CTGCCCCATGTCTGCTGCTGCTGG#.(((((.((((((((((((((.#)))))...)))))).)))))))).	1279	1303	0.92308	-28	0	0.0001158	0.4	-0	-0.3	-5	19	24	8	3UTR
mmu-miR-214-3p	NM_001159561	P2rx6	ACAGCAGGCACAGACAGGCAGT#CCTTCCTCTGTGACCTGCTGC#.((((((((((((.(((....#)))...)))))).))))))).	1108	1129	1	-25	1	9.83E-08	0.4	2.5	1.07	-5	16	21	7	CDS
mmu-miR-214-3p	NM_001159602	Mymk	ACAGCAGGCACAGACAGGCAGT#CTGCCTGGCAGTCCTGCTGTC#((((((((.((...(((((((.#)))))))...)))))))))).	703	724	0.92308	-28	1	6.88E-06	0.4	3.2	3.82	-10	17	21	10	3UTR
mmu-miR-214-3p	NM_001159602	Mymk	ACAGCAGGCACAGACAGGCAGT#CTGCCTGTCAGAATCGTCTGCTT#..(((((((...(((((((((.#)))))))))......))))))).	988	1011	1	-26	1	0.0099818	0.5	2.6	2.93	-12	16	23	9	3UTR
mmu-miR-214-3p	NM_001159602	Mymk	ACAGCAGGCACAGACAGGCAGT#CTGGCCATGTCCTTTGTCCTGCTGC#.(((((((.((((((((((((.#))).)).))))...)))))))))).	552	577	1	-24	1	0.0251558	0.4	2.3	1.03	-5	19	25	10	CDS

mmu-miR-214-3p	NM_001159696	Tfap2c	ACAGCAGGCACAGACAGGCAGT#ACTGGACCTCTAAGCCTGTCCGTGCTCCTTAAATAATTGTTGTG#((((((((((.(((((((...#.))))))).))))).........)))))).	2689	2733	1	-26	1	0.1536174	0.7	0.7	1	-7	18	32	7	3UTR
mmu-miR-214-3p	NM_001159774	Ikbkb	ACAGCAGGCACAGACAGGCAGT#ACTCCTGGGTGCCTGTCAGGTGTGTGTGGATATTGCTGC#.((((((((((.((((((((.(#.).))))))))..)))).........)))))).	3059	3098	1	-25	1	0.0011001	0.6	2.8	3.83	-7	19	33	8	3UTR
mmu-miR-214-3p	NM_001159774	Ikbkb	ACAGCAGGCACAGACAGGCAGT#ACAGCCTGTCTGCTTTGGACTCCTGGGGGAGGAAGCTCTGCCCTGCC#...(((((((((((((((.((#)).)))))))))).........................)))).))).	2662	2709	0.84615	-24	0	0.0024968	0.4	3.1	2.8	-7	18	47	9	3UTR
mmu-miR-214-3p	NM_001159941	Kctd10	ACAGCAGGCACAGACAGGCAGT#GCTGCTAGCCGAGGCCAAGTACTACCTGGTGCAGGGCCTGCTGG#.((((((((.....((((.(((#.))).)))).......)))))))).	397	441	0.84615	-24	1	1.70E-10	0.4	0.1	0.24	-13	15	25	8	CDS
mmu-miR-214-3p	NM_001159986	Sec16b	ACAGCAGGCACAGACAGGCAGT#CTGCTTCTGTGTCTGG#....((((((((((..(((((.#)))))))))))))))).	662	678	0.92308	-23	0	3.35E-07	0.5	0.4	0.46	-11	15	16	15	CDS
mmu-miR-214-3p	NM_001159986	Sec16b	ACAGCAGGCACAGACAGGCAGT#GCTGCTTTCTACCTCTCAGTGTCCACCTCACCGCATCATATCCACACCCTTGTTGTGAGCCTGCTGC#.((((((((.((((((((....#.)))))).)).)))))))).	3366	3433	1	-23	1	0.0003458	0.4	0	-0.3	-5	16	20	8	3UTR
mmu-miR-214-3p	NM_001160404	Galnt1	ACAGCAGGCACAGACAGGCAGT#CTGCTGAATGTGCCTTGTGA#.((..((((((((....(((((.#)))))...))))))))..)).	2274	2294	1	-22	0	0.0073382	0.5	1.2	1.71	-14	14	20	7	3UTR
mmu-miR-214-3p	NM_001160404	Galnt1	ACAGCAGGCACAGACAGGCAGT#CTGTCTGTCAGGTGATACTTGATGG#.((.((((((((.(((((((((.#)))))))))..)))...)))).)).	3359	3384	0.84615	-21	0	3.65E-06	0.6	0.4	0.76	-10	18	25	9	3UTR
mmu-miR-214-3p	NM_001160404	Galnt1	ACAGCAGGCACAGACAGGCAGT#ACTCTTGGATATGTTCCTGCTGC#.((((((((.(((..((((.(((#)))))))....))).)))))))).	300	323	0.92308	-21	1	0.0007721	0.6	3.9	4.92	-7	17	23	7	CDS
mmu-miR-214-3p	NM_001161420	Vldlr	ACAGCAGGCACAGACAGGCAGT#TGTCTGTCTTTGCTCCTGCTA#..((((((((.(((((((((..#))))))))).)))..))))).	7178	7199	1	-24	1	0.0004164	0.6	0	0.11	-8	17	21	9	3UTR

mmu-miR-214-3p	NM_001161420	Vldlr	ACAGCAGGCACAGACAGGCAGT#GCTGTCTTAGTACTGTCACTGTTGTA#((((((..((((((((((((#)))))))..)).))))..))))))).	8089	8115	0.92308	-23	1	0.0338413	0.5	0.2	0.24	-7	20	26	7	3UTR
mmu-miR-214-3p	NM_001161420	Vldlr	ACAGCAGGCACAGACAGGCAGT#GCTGCCATGGTCTAGGGTGAGGGTGCTGTC#((((((..(((((((.((((((#))))))...))))...)))....))	7421	7451	0.92308	-23	0	0.0004878	0.5	0.2	0.02	-8	19	30	6	3UTR
mmu-miR-214-3p	NM_001161515	Dctd	ACAGCAGGCACAGACAGGCAGT#CCTGTGTCTCCTGCTGTG#((((((((.(.(.(((((....#))))).).))))))))).	1655	1673	0.84615	-21	1	0.0060064	0.4	0.4	-0.4	-9	15	18	9	3UTR
mmu-miR-214-3p	NM_001161516	Dctd	ACAGCAGGCACAGACAGGCAGT#CCTGTGTCTCCTGCTGTG#((((((((.(.(.(((((....#))))).).))))))))).	1436	1454	0.84615	-21	1	0.0060064	0.4	0.4	-0.4	-9	15	18	9	3UTR
mmu-miR-214-3p	NM_001161842	Il18r1	ACAGCAGGCACAGACAGGCAGT#GCTGCTGTGACAGGGGCACCCTAGCTCATGTTTTTGTGCCTGCA#...(((((((((((((((((#))))))......................))))..))))))))).	1564	1608	0.84615	-25	0	0.0022519	0.5	0	0.84	-7	19	44	9	CDS
mmu-miR-214-3p	NM_001161842	Il18r1	ACAGCAGGCACAGACAGGCAGT#ACTGTCTTCTGCTGTCTGGAGC#.(..((((((((((.(((((((#))))))))))).))))))..).	368	390	0.96154	-22	0	0.0001239	0.5	-0	0.06	-9	18	22	11	5UTR
mmu-miR-214-3p	NM_001161843	Il18r1	ACAGCAGGCACAGACAGGCAGT#GCTGCTGTGACAGGGGCACCCTAGCTCATGTTTTTGTGCCTGCA#...(((((((((((((((((#))))))......................))))..))))))))).	1436	1480	0.84615	-25	0	0.0022519	0.5	0	0.84	-7	19	44	9	CDS
mmu-miR-214-3p	NM_001161843	Il18r1	ACAGCAGGCACAGACAGGCAGT#TGCTTGTTCTCGCCTCTGTG#((((.((((...((((((((..#))))))))...)))))))).	1315	1335	0.92308	-21	0	7.41E-05	0.5	0	0.73	-15	16	20	8	CDS
mmu-miR-214-3p	NM_001162908	Sesn1	ACAGCAGGCACAGACAGGCAGT#ACTGCCTGTCTAGCAGTGAGCACTCGGCAGCTTTGGTGTTAGAGCTGG#.((((.((((((((((((((((#)))))))))))).........................)))))...)))).	2260	2308	0.92308	-27	0	0.0007138	0.4	1.1	1.66	-6	20	48	11	3UTR
mmu-miR-214-3p	NM_001162908	Sesn1	ACAGCAGGCACAGACAGGCAGT#CTGCCCCATGCCCTGCTGA#.(((((((..((....(((((.#)))))...)).))))))).	2926	2945	0.92308	-21	1	0.000326	0.4	1.2	0.98	-9	14	19	7	3UTR
mmu-miR-214-3p	NM_001162932	Rmi2	ACAGCAGGCACAGACAGGCAGT#CTGCCTTCCTGCTGCTGCTGC#.((((((.(((((((..((((((.#))))))..))).)))))))).	620	641	1	-25	0	0.0006984	0.4	0.1	0.04	-7	17	21	8	3UTR

mmu-miR-214-3p	NM_001162932	Rmi2	ACAGCAGGCACAGACAGGCAGT#TGTGTGTGTGTGTGTGCG#...(((.(((((.(((.(((..#))).))).))))).))).	704	722	0.84615	-23	0	0.0053482	0.4	0.2	-0.2	-9	14	18	5	3UTR
mmu-miR-214-3p	NM_001163144	Pcsk5	ACAGCAGGCACAGACAGGCAGT#GCTGCAGCCGCCCGGGACGGCGGGACCTGCTGTGCGTGCTGG#.(((((.((((((.((((....#.)))))))))).))))).	502	544	0.92308	-29	0	3.46E-06	0.3	0.1	0.36	-6	15	18	10	CDS
mmu-miR-214-3p	NM_001163144	Pcsk5	ACAGCAGGCACAGACAGGCAGT#GCTGCCCTCCTGGCCACTACCATGCTGA#.((((((((.(((...((((((#))))))...))))))).......))))).	2503	2531	0.92308	-23	1	0.0027369	0.4	2.6	2.41	-7	17	28	6	CDS
mmu-miR-214-3p	NM_001163170	Lix1l	ACAGCAGGCACAGACAGGCAGT#GCTTCATGCTTGTGGTTGTATCCTGCTGA#.(((((((.((((((((((((..#.))))))))..)))))..))))))).	1762	1791	0.92308	-23	1	0.0112484	0.5	-0	-0	-5	18	24	7	3UTR
mmu-miR-214-3p	NM_001163170	Lix1l	ACAGCAGGCACAGACAGGCAGT#GCTGGCTGCTGGGCAGTTGG#.((((..((.(((.(((.((((#)))).))))))).)).))))).	1017	1037	0.92308	-21	0	2.97E-07	0.5	4	2.92	-9	16	20	6	CDS
mmu-miR-214-3p	NM_001163283	Zbtb5	ACAGCAGGCACAGACAGGCAGT#GCCTGTCCCTGGCGCTCAACAGAGCCTGCCTCTCTGCTGCTGG#.((((((...((((.((((((.#.)))))).))))))))))).	4342	4385	1	-25	1	0.0040209	0.4	1	0.88	-9	16	19	10	3UTR
mmu-miR-214-3p	NM_001163283	Zbtb5	ACAGCAGGCACAGACAGGCAGT#TGCCTGCAAAATCTGCTGTA#((((((((....(.((((((..#)))))))....)))))))).	2387	2407	1	-22	1	0.0037742	0.5	3.7	3.21	-13	15	20	8	CDS
mmu-miR-214-3p	NM_001163284	Zbtb5	ACAGCAGGCACAGACAGGCAGT#GCCTGTCCCTGGCGCTCAACAGAGCCTGCCTCTCTGCTGCTGG#.((((((...((((.((((((.#.)))))).))))))))))).	5237	5280	1	-25	1	0.0040209	0.4	1	0.88	-9	16	19	10	3UTR
mmu-miR-214-3p	NM_001163284	Zbtb5	ACAGCAGGCACAGACAGGCAGT#TGCCTGCAAAATCTGCTGTA#((((((((....(.((((((..#)))))))....)))))))).	3282	3302	0.92308	-22	1	0.0037742	0.5	1.6	2.48	-13	15	20	8	CDS
mmu-miR-214-3p	NM_001163336	Atp2a3	ACAGCAGGCACAGACAGGCAGT#GCTGTGGCCCTGGCTGTGGCTGCTA#..((((((.(((((.((((((((#))))....)))).))))).)))))).	1079	1104	0.84615	-26	0	4.46E-06	0.4	3.3	3.94	-8	18	25	5	CDS
mmu-miR-214-3p	NM_001163336	Atp2a3	ACAGCAGGCACAGACAGGCAGT#CTGCCTGTCATCCTGCTC#..((((((....(((((((((.#)))))))))..)))))).	3107	3125	0.92308	-26	1	0.0004631	0.4	3.4	2.93	-13	15	18	9	CDS

mmu-miR-214-3p	NM_001163337	Atp2a3	ACAGCAGGCACAGACAGGCAGT#GCTGTGGCCCTGGCTGTGGCTGCTA#..(((((.(((((.((((((((#))))....)))).))))).))))).	1079	1104	0.84615	-26	0	4.46E-06	0.4	3.3	3.94	-8	18	25	5	CDS
mmu-miR-214-3p	NM_001163337	Atp2a3	ACAGCAGGCACAGACAGGCAGT#CTGCCTGTCATCCTGCTC#..((((((....(((((((((.#)))))))))..)))))).	3107	3125	0.92308	-26	1	0.0004777	0.4	3.4	2.93	-13	15	18	9	CDS
mmu-miR-214-3p	NM_001163356	Inka2	ACAGCAGGCACAGACAGGCAGT#CTGAGTGCCCTCGCTGGCAGGGTAGTGGAGGGCCTGCTGGGCCTGCTGC#.((((((((.(((.(((((...#.)))))))).)))))))).	243	292	0.97436	-31	1	3.64E-05	0.4	0.2	0.27	-6	16	19	8	CDS
mmu-miR-214-3p	NM_001163356	Inka2	ACAGCAGGCACAGACAGGCAGT#GCCTGTCTGTGGAACTGGGGCCTGTCTGC#.(((((((((((((((((...#))))))))))).........))))).))).	4185	4214	0.96154	-30	1	0.0004283	0.4	-0	-0.3	-4	18	29	10	3UTR
mmu-miR-214-3p	NM_001163387	Nlgn1	ACAGCAGGCACAGACAGGCAGT#TGCTTGGTGGCTGTGCTGCTGG#.(((((.((((((.((((((..#))))))....))))))))))).	4150	4172	1	-24	0	2.00E-05	0.6	0.1	0.14	-6	17	22	11	3UTR
mmu-miR-214-3p	NM_001163387	Nlgn1	ACAGCAGGCACAGACAGGCAGT#CTGCTTGTTGGGATGTTTGCTA#..(((((((((.(((((((((.#)))))))))..).)))))))).	1132	1154	0.84615	-23	1	0.0004424	0.5	-0	-0	-8	18	22	9	CDS
mmu-miR-214-3p	NM_001163387	Nlgn1	ACAGCAGGCACAGACAGGCAGT#GCGGCATCTGTCTGTGTGAAATCAATGGGAGCTACTTACTGCATGA#.(((((((((((((((((.((#)).))..))))))))))....................)))).)).	857	903	0.92308	-21	1	0.0065081	0.6	-0	-0	-4	20	46	10	5UTR
mmu-miR-214-3p	NM_001163610	Nhsl2	ACAGCAGGCACAGACAGGCAGT#TTGCCTGTACTCCTGGCCTGCATGG#.((((((((.(((((((((((.#))))))))....))))))))).)).	6854	6879	0.92308	-28	1	0.0002534	0.4	-0	0.25	-5	19	25	9	3UTR
mmu-miR-214-3p	NM_001163610	Nhsl2	ACAGCAGGCACAGACAGGCAGT#AGTGTCTGTCAACATAGGTAGCCAGGATCTGTGCCCAGCTCATGCTGTA#(((((((((((((((.(((...#.)))..).)))))))).......)))))).	4561	4610	0.92308	-26	1	8.71E-05	0.5	0.3	0.55	-4	18	30	8	3UTR
mmu-miR-214-3p	NM_001163610	Nhsl2	ACAGCAGGCACAGACAGGCAGT#GCCTGTCTACTGCCAACAAGCAAAGTGCCTGG#....((((((((((((((((...#)))))))))..............	817	849	1	-26	0	0.0011226	0.5	1.6	1.72	-10	15	32	8	CDS
mmu-miR-214-3p	NM_001163616	Tmem273	ACAGCAGGCACAGACAGGCAGT#ATTGCCACACACAACTTGGTGCTGATGGTTGCCTCGTGTCTGCATGA#.((((((((((.((..(((((.#.)))))))))))))))).)).	1007	1054	0.92308	-21	1	0.0002468	0.5	-0	-0.2	-8	17	20	15	3UTR

mmu-miR-214-3p	NM_001163617	Tars2	ACAGCAGGCACAGACAGGCAGT#ACTGGCTGATACTGCAGTGGCTGCTGA#.((((((.(((((.(((.((((#)))).)))...))...))).)))))).	423	450	0.84615	-24	1	0.0002132	0.5	0.5	1.69	-7	18	27	6	CDS
mmu-miR-214-3p	NM_001163617	Tars2	ACAGCAGGCACAGACAGGCAGT#ACTGCCTGTGCGACTGGCTGATTTCGGAGCCCTGCA#...(((((..((((((((((((#))))))))))))....)))..............))))).	1428	1464	0.92308	-22	0	0.0001458	0.4	2.6	1.64	-9	17	36	9	CDS
mmu-miR-214-3p	NM_001163618	Tars2	ACAGCAGGCACAGACAGGCAGT#ACTGGCTGATACTGCAGTGGCTGCTGA#.((((((.(((((.(((.((((#)))).)))...))...))).)))))).	423	450	0.84615	-24	1	0.0002132	0.5	0.5	1.69	-7	18	27	6	CDS
mmu-miR-214-3p	NM_001163618	Tars2	ACAGCAGGCACAGACAGGCAGT#ACTGCCTGTGCGACTGGCTGATTTCGGAGCCCTGCA#...(((((..((((((((((((#))))))))))))....)))..............))))).	1185	1221	0.92308	-22	0	0.0001458	0.4	0	2.35	-9	17	36	9	CDS
mmu-miR-214-3p	NM_001163621	Apol6	ACAGCAGGCACAGACAGGCAGT#ACTGGTCACAGTGCCTGCTGG#.((((((((((.(((...((((#)))))))...)))))))))).	52	73	0.84615	-25	1	1.54E-05	0.4	-0	0	-7	17	21	10	5UTR
mmu-miR-214-3p	NM_001163637	Jakmip 2	ACAGCAGGCACAGACAGGCAGT#GCCTGAGGAGGTGTCTGCTGC#.((((((((((...(((((...#))))).....)))))))))).	22	43	1	-27	1	1.66E-05	0.4	0.7	1.47	-9	15	21	10	5UTR
mmu-miR-214-3p	NM_001163637	Jakmip 2	ACAGCAGGCACAGACAGGCAGT#GCTGCTGCTGTTGCCGCCTCTACTGCTGG#.(((((((((((.((.(((((#))))))))))).)))........))))).	102	131	1	-23	1	1.84E-05	0.4	1.2	1.03	-5	19	29	11	5UTR
mmu-miR-214-3p	NM_001163671	Mapk9	ACAGCAGGCACAGACAGGCAGT#CTGTGCCTGTTGG#.((((((((((((.........#)))))))))))).	2662	2675	0.92308	-23	1	0.0004919	0.5	-0	-0.1	-6	12	13	12	3UTR
mmu-miR-214-3p	NM_001163672	Mapk9	ACAGCAGGCACAGACAGGCAGT#CTGTGCCTGTTGG#.((((((((((((.........#)))))))))))).	2667	2680	0.92308	-23	1	0.0004919	0.5	-0	-0.1	-6	12	13	12	3UTR
mmu-miR-214-3p	NM_001163675	Abcc4	ACAGCAGGCACAGACAGGCAGT#ATGGCCTGTCGTTTTATTTTAAAAGTTCCTGCTGTT#((((((((.((.(((((((.((#)).))))))).............)).)))))))).	4254	4290	0.92308	-23	1	0.0409318	0.6	2.5	2.34	-8	19	36	8	3UTR
mmu-miR-214-3p	NM_001163675	Abcc4	ACAGCAGGCACAGACAGGCAGT#TTGCCAGTTCGTCTCCCTGCTGTC#((((((((.((.(((.(((((.#))))).))).))...)))))))).	4479	4503	1	-23	1	0.0112708	0.5	2.3	2.76	-9	18	24	8	3UTR

mmu-miR-214-3p	NM_001163676	Abcc4	ACAGCAGGCACAGACAGGCAGT#TTGCCAGTTCGTCTCCCTGCTGTC#((((((((.((.(((.(((((.#))))).))).))...)))))))).	4272	4296	1	-23	1	0.0112708	0.5	2.5	2.41	-9	18	24	8	3UTR
mmu-miR-214-3p	NM_001163703	Dcun1d3	ACAGCAGGCACAGACAGGCAGT#CTGCCTGCTGCCCTGCA#...(((((..(((.(((((((.#)))))))))).))))).	1238	1255	0.92308	-26	0	8.07E-06	0.5	0.8	0.25	-10	15	17	10	3UTR
mmu-miR-214-3p	NM_001163703	Dcun1d3	ACAGCAGGCACAGACAGGCAGT#GCTGCCACTGAGGCCTGCC#...((((((.(((...((((((#)))))).)))..)))))).	473	492	0.84615	-25	0	3.89E-07	0.4	2	0.16	-10	15	19	6	CDS
mmu-miR-214-3p	NM_001163703	Dcun1d3	ACAGCAGGCACAGACAGGCAGT#GCAGGTCTGTACTGTGTGTAGTGCTGTT#((((((.((((((((((((.((#))..)))))).))))))).)...))))).	1643	1671	0.84615	-23	0	0.0009451	0.6	0.5	0.37	-5	20	28	6	3UTR
mmu-miR-214-3p	NM_001163741	Ccdc167	ACAGCAGGCACAGACAGGCAGT#GCCGTCTGTGAACAGAAACCCTGCTGA#.((((((((((((((.(((...#))))))))))).........))))))).	2485	2512	0.89744	-27	1	0.0050089	0.4	-0	0.03	-4	17	27	10	3UTR
mmu-miR-214-3p	NM_001163741	Ccdc167	ACAGCAGGCACAGACAGGCAGT#CTGCCTACCCTGTGTGCCATGCTGG#.(((((((((((.(.((((((.#))))))....).))))))).))))).	1999	2024	0.92308	-28	1	0.0008572	0.4	4.4	3.84	-6	18	25	6	3UTR
mmu-miR-214-3p	NM_001163741	Ccdc167	ACAGCAGGCACAGACAGGCAGT#GCTGTCTGCTGTGCAGGTGCA#...(((.((((((.((((((((#))))))))))))))...))).	1963	1984	0.84615	-26	0	6.71E-07	0.4	0.1	2.25	-9	17	21	14	3UTR
mmu-miR-214-3p	NM_001163760	6430548M08Rik	ACAGCAGGCACAGACAGGCAGT#CCTGCCTGTGTCTGCTC#..(((((((((((.((((....#)))).))))))))))).	1630	1647	1	-26	1	0.0651706	0.4	0.6	0.26	-6	15	17	11	3UTR
mmu-miR-214-3p	NM_001163761	6430548M08Rik	ACAGCAGGCACAGACAGGCAGT#CCTGCCTGTGTCTGCTC#..(((((((((((.((((....#)))).))))))))))).	1621	1638	1	-26	1	0.0651706	0.4	0.6	0.26	-6	15	17	11	3UTR
mmu-miR-214-3p	NM_001163762	6430548M08Rik	ACAGCAGGCACAGACAGGCAGT#GCCCGAGTCATCCGATCCTGTTTGTGCTGCAGCTGA#.((((.((((((((((((....#.))))))))))))	1618	1654	0.84615	-26	0	0.0006382	0.4	-1	-0	-6	16	21	12	3UTR
mmu-miR-214-3p	NM_001164036	Ly6e	ACAGCAGGCACAGACAGGCAGT#GCTGGTCACACATTCCCAAGCTTTGTCTGCCCCAGAGACTTCAGAGGCCTGCTGTT#(((((((((.(((((((((...#.)))).)))))).................	2076	2132	0.84615	-25	1	0.0003999	0.5	0.1	0.11	-5	18	38	9	3UTR
mmu-miR-214-3p	NM_001164037	Ly6e	ACAGCAGGCACAGACAGGCAGT#GCTGGTCACACATTCCCAAGCTTTGTCTGCCCCAGAGACTTCAGAGGCCTGCTGTT#(((((((((.(((((((((...#.)))).)))))).................	1929	1985	0.84615	-25	1	0.0003999	0.5	0.1	0.11	-5	18	38	9	3UTR

mmu-miR-214-3p	NM_001164038	Ly6e	ACAGCAGGCACAGACAGGCAGT#GCTGGTCACACATTCCCAAGCTTTGTCTGCCCCAGAGACTTCAGAGGCCTGCTGTT#(((((((((.(((((((((...#.)))).)))))..................	1845	1901	0.84615	-25	1	0.0003999	0.5	0.1	0.11	-5	18	38	9	3UTR
mmu-miR-214-3p	NM_001164040	Ly6e	ACAGCAGGCACAGACAGGCAGT#GCTGGTCACACATTCCCAAGCTTTGTCTGCCCCAGAGACTTCAGAGGCCTGCTGTT#(((((((((.(((((((((...#.)))).)))))..................	1694	1750	0.84615	-25	1	0.0003999	0.5	0.1	0.11	-5	18	38	9	3UTR
mmu-miR-214-3p	NM_001164159	Ppp6r3	ACAGCAGGCACAGACAGGCAGT#ATTGCCCTGTTTGAAGCATGTTGTA#((((((.((.((((((((((((#)))))).))))))..)).)))))).	1768	1793	0.92308	-22	0	0.0026079	0.6	1.7	2.28	-7	20	25	6	CDS
mmu-miR-214-3p	NM_001164190	Mtm1	ACAGCAGGCACAGACAGGCAGT#ACTGTGCGAGGAGCTGGCTGGCTTCTGCTGC#.(((((((...((.(((.((((#.)))).))).))))))))).	2759	2790	0.92308	-21	1	2.26E-05	0.5	-0	0.14	-10	16	20	9	3UTR
mmu-miR-214-3p	NM_001164191	Mtm1	ACAGCAGGCACAGACAGGCAGT#ACTGTGCGAGGAGCTGGCTGGCTTCTGCTGC#.(((((((...((.(((.((((#.)))).))).))))))))).	2650	2681	0.92308	-21	1	2.26E-05	0.5	-0	0.14	-10	16	20	9	3UTR
mmu-miR-214-3p	NM_001164192	Mtm1	ACAGCAGGCACAGACAGGCAGT#ACTGTGCGAGGAGCTGGCTGGCTTCTGCTGC#.(((((((...((.(((.((((#.)))).))).))))))))).	2557	2588	0.92308	-21	1	2.26E-05	0.5	-0	0.14	-10	16	20	9	3UTR
mmu-miR-214-3p	NM_001164222	Cert1	ACAGCAGGCACAGACAGGCAGT#GCTTCTTTTTACAGGCCTACAGTCTGTGTCTGTG#...((((((((((((((((.((#.))..))))...))))))))	2672	2706	1	-26	0	0.0001878	0.6	1.2	1.57	-7	18	25	12	3UTR
mmu-miR-214-3p	NM_001164357	Slc25a25	ACAGCAGGCACAGACAGGCAGT#GCTGCCCATGCTCAATGAGTGGCCTGCTGG#.((((((((.((..((((((((#))))))..........))..))))))))	3115	3145	0.84615	-27	1	5.25E-09	0.4	1.7	1.25	-7	18	30	10	3UTR
mmu-miR-214-3p	NM_001164357	Slc25a25	ACAGCAGGCACAGACAGGCAGT#GCTGCCTGCATGGGCCTAACGTCCTCTGAGCTGC#.((((((((.((..((((((((#))))))))..)).))))) ....)))).	2324	2358	0.84615	-26	1	0.0011327	0.4	3.8	3.67	-7	18	34	8	3UTR
mmu-miR-214-3p	NM_001164357	Slc25a25	ACAGCAGGCACAGACAGGCAGT#GCTGCCTGACAGCGTGGTGCATGGCTTTGCTGA#.((((((((((...((((((((#))))))))........)))). .....)))))).	2255	2288	0.84615	-22	1	2.47E-05	0.4	5.5	3.35	-9	18	33	8	3UTR
mmu-miR-214-3p	NM_001164358	Slc25a25	ACAGCAGGCACAGACAGGCAGT#GCTGCCCATGCTCAATGAGTGGCCTGCTGG#.((((((((.((..((((((((#))))))..........))..))))))))	3370	3400	0.84615	-27	1	5.25E-09	0.4	0.7	0.72	-7	18	30	10	3UTR

mmu-miR-214-3p	NM_001164358	Slc25a25	ACAGCAGGCACAGACAGGCAGT#GCTGCCTGCATGGGCCTAACGTCCTCTGAGCTGC#.((((((((.((..((((((((#))))))))..)).)))))............)))).	2579	2613	0.84615	-26	1	0.0011327	0.4	4.4	3.42	-7	18	34	8	3UTR
mmu-miR-214-3p	NM_001164358	Slc25a25	ACAGCAGGCACAGACAGGCAGT#GCTGCCTGACAGCGTGGTGCATGGCTTTGCTGA#.((((((((((...((((((((#))))))))........)))).....)))))).	2510	2543	0.92308	-22	1	2.47E-05	0.4	4	4.14	-9	18	33	8	3UTR
mmu-miR-214-3p	NM_001164401	Ptpro	ACAGCAGGCACAGACAGGCAGT#CTGCCTCCAGGAGATATCTACAATCTGTCTGTCACGGCCTGCA#...((((((...(((((((((.#.)))))))))...)))))).	2493	2536	0.98077	-28	0	0.0031247	0.4	2.8	3.73	-14	15	20	9	CDS
mmu-miR-214-3p	NM_001164401	Ptpro	ACAGCAGGCACAGACAGGCAGT#ACTGCTGTCGTGCTGAGCTGG#.((((.((((((.((((.(((((#))))))))))))))..)))).	2046	2067	0.92308	-26	0	8.52E-05	0.4	2	3.58	-9	18	21	14	CDS
mmu-miR-214-3p	NM_001164402	Ptpro	ACAGCAGGCACAGACAGGCAGT#GTCAGTGACCCTGCTGTG#(((((((((((.(((.......#))).)))..)))))))).	173	191	0.92308	-20	1	6.39E-07	0.5	2.9	3.1	-8	14	18	8	5UTR
mmu-miR-214-3p	NM_001164403	Ptpro	ACAGCAGGCACAGACAGGCAGT#GTCAGTGACCCTGCTGTG#(((((((((((.(((.......#))).)))..)))))))).	173	191	0.92308	-20	1	6.39E-07	0.5	2.9	3.1	-8	14	18	8	5UTR
mmu-miR-214-3p	NM_001164441	Ankrd33b	ACAGCAGGCACAGACAGGCAGT#ACTGCCTGGATCATGGTCTGTGCTGCC#...(((.((((((((((((((#)))))))........))))))))))).	5609	5636	0.80769	-27	0	0.0026289	0.4	-0	0.01	-8	18	27	11	3UTR
mmu-miR-214-3p	NM_001164441	Ankrd33b	ACAGCAGGCACAGACAGGCAGT#CTGGCTGTGGTGTGATTCCTGTTGTT#((((((((((((.((((.(((.#))).))))..))))...)))))))).	6596	6622	1	-25	1	0.0126794	0.6	3.8	3.47	-5	19	26	8	3UTR
mmu-miR-214-3p	NM_001164441	Ankrd33b	ACAGCAGGCACAGACAGGCAGT#ACTTGGCCCATCATGTCCTTGATGGGCAGTGTGCTTGCTGC#.((((((((((((((((((((#)))..))).....)))).............)))))))))).	5103	5144	0.84615	-23	1	4.08E-07	0.4	-1	-0.1	-4	21	41	11	3UTR
mmu-miR-214-3p	NM_001164504	Rnf165	ACAGCAGGCACAGACAGGCAGT#CTGCCGTTCTGTTCTGTTGTGTGCTGTG#((((((.(((((((((((((((.#)))))....))).)))).)).)))))).	7083	7111	0.92308	-26	0	0.0036574	0.6	3	3.4	-5	20	28	6	3UTR
mmu-miR-214-3p	NM_001164504	Rnf165	ACAGCAGGCACAGACAGGCAGT#ACTGCCTCCCGCAGAGCTCCTCGCGGGCTCGCTGTGCTGTTGTG#((((((.((((((..(((((((#)))))))........................)))))))))))).	6845	6889	0.84615	-24	0	0.0022366	0.3	3.9	3.45	-7	19	44	12	3UTR

mmu-miR-214-3p	NM_001164504	Rnf165	ACAGCAGGCACAGACAGGCAGT#GCTGCAGACAGAGAGGGCCTGCTGTG#(((((((((........(((((#)))))...........))))))))).	6348	6374	0.92308	-23	1	7.62E-06	0.5	3.8	3.9	-14	14	26	9	3UTR
mmu-miR-214-3p	NM_001164528	Ildr2	ACAGCAGGCACAGACAGGCAGT#GCTGCTTGCTGATCTCTTGCCCAGTTTTGCTGTG#(((((((((((((.((((((((#))))))))))))......))))))......)))))).	791	825	1	-25	1	0.0001338	0.5	4	3.39	-5	21	34	11	CDS
mmu-miR-214-3p	NM_001164528	Ildr2	ACAGCAGGCACAGACAGGCAGT#GCTGCCCTCACAGTTGCTGCTGCTATGTCCGCTGCCCATGCTGC#.((((((((((.((((((((((#.)))))).)))).).)))))..))))).	913	957	0.84615	-26	1	0.0017727	0.4	3.9	3.17	-7	20	27	6	CDS
mmu-miR-214-3p	NM_001164528	Ildr2	ACAGCAGGCACAGACAGGCAGT#CTGCCCAGTTGTTTTTGTGCCTTGTCTGTG#((((((((((((((((((((.#)))))....))))..))))))).).)))	8108	8138	0.92308	-25	1	0.0006165	0.6	0.8	0.15	-4	21	30	7	3UTR
mmu-miR-214-3p	NM_001164598	Irf2bp2	ACAGCAGGCACAGACAGGCAGT#GGCTGTCTGTGTTTGACTCTTTGGGTGACAGTTGCTGTT#((((((((((((((((.(...#).))))))))))).................))))))).	2505	2544	0.92308	-25	1	0.000654	0.5	4.2	1.9	-4	18	39	10	3UTR
mmu-miR-214-3p	NM_001164598	Irf2bp2	ACAGCAGGCACAGACAGGCAGT#ACTGTCTGTCATTTCTCCTCGCTGG#.((((((((....((((((((((#))))))))))......))).)))).	2729	2754	0.92308	-24	1	0.0124328	0.5	0.4	3.49	-12	17	25	10	3UTR
mmu-miR-214-3p	NM_001164663	Soga1	ACAGCAGGCACAGACAGGCAGT#CTGGAGCTCTGGAGACCCCTGTCTGTGCCTACATGTA#((((.(((((((((((((....#.))))))))	5905	5942	0.92308	-28	0	0.0007391	0.4	0.6	0.12	-8	17	21	13	3UTR
mmu-miR-214-3p	NM_001164797	Hephl1	ACAGCAGGCACAGACAGGCAGT#CTGTCTGTCTGGAGTCACACTGG#.(((..(((.(((((((((((.#)))))))))))..)))...))).	3871	3894	1	-24	0	0.0003758	0.5	3.3	2.23	-11	17	23	11	3UTR
mmu-miR-214-3p	NM_001164797	Hephl1	ACAGCAGGCACAGACAGGCAGT#GCTGCTGACCCTCAGTCTGCTGTG#(((((((((..(((...((((((#))))))....))..))))))))).	3726	3750	0.92308	-24	1	8.00E-05	0.5	2.6	2.41	-10	17	24	9	3UTR
mmu-miR-214-3p	NM_001164797	Hephl1	ACAGCAGGCACAGACAGGCAGT#GCCTGAGATGTGCGTTGGCGAATCTGTGTCCTGGCACCTGTT#((((((((((((((((((...#)))))).................)))))).))))....)))).	843	885	0.84615	-23	1	0.0013898	0.5	0.3	0.13	-4	19	42	6	CDS
mmu-miR-214-3p	NM_001164818	Aar2	ACAGCAGGCACAGACAGGCAGT#TTGCCTTTGTGTGCTTCTTGCTGG#.((((((((((((.(.((((((.#)))))).).))))))...))))).	1264	1288	1	-25	1	4.55E-05	0.5	3.7	3.32	-6	18	24	6	CDS

mmu-miR-214-3p	NM_001164818	Aar2	ACAGCAGGCACAGACAGGCAGT#CTGCCTGCTCCTGGTTGGATGGAAGGAAACCTGCTGG#.(((((((..(((.(((((((.#)))))))...)))...............))))))).	2077	2114	1	-25	1	0.0024715	0.4	0.4	0.02	-7	17	37	7	3UTR
mmu-miR-214-3p	NM_001165902	Ctnnb1	ACAGCAGGCACAGACAGGCAGT#ACTGCCTGTTGTGGTTAAACTCCTGCA#...(((((((((.((((((((((#)))))))))))))))))........))))).	1556	1583	0.80769	-26	0	0.003937	0.5	3.7	4.23	-8	18	27	13	CDS
mmu-miR-214-3p	NM_001165902	Ctnnb1	ACAGCAGGCACAGACAGGCAGT#GCTGTCTGTCTGCTCTAGCAACAAGCCGGCC#...((.(((.(((((((((((((#))))))))))))))))............))	1124	1155	1	-24	0	0.0002893	0.4	3.9	4.36	-10	17	31	12	CDS
mmu-miR-214-3p	NM_001165902	Ctnnb1	ACAGCAGGCACAGACAGGCAGT#GCCTGTTTGTAAATCTGCC#...(((((.((((((((((...#))))))))))...))))).	2535	2554	1	-23	0	0.0017889	0.6	1.5	1.71	-8	15	19	10	3UTR
mmu-miR-214-3p	NM_001165930	Glt8d1	ACAGCAGGCACAGACAGGCAGT#ACTGCATCTAGCTGCTGTAGGTAGGAACTGCTGTT#(((((((..((..(((.(((((#.)))))))))..))....	1411	1446	0.84615	-21	1	8.10E-05	0.6	0.6	0.29	-12	17	26	8	3UTR
mmu-miR-214-3p	NM_001165983	Ubap2l	ACAGCAGGCACAGACAGGCAGT#CTTCCAGTATGGGCCTGCTGTG#(((((((((.((.((.((.((.#)).)).)).)).))))))))).	2974	2996	0.92308	-23	1	5.14E-06	0.4	2.5	3.37	-7	17	22	9	CDS
mmu-miR-214-3p	NM_001165984	Ubap2l	ACAGCAGGCACAGACAGGCAGT#CTTCCAGTATGGGCCTGCTGTG#(((((((((.((.((.((.((.#)).)).)).)).))))))))).	2929	2951	1	-23	1	5.14E-06	0.4	2.5	2.9	-7	17	22	9	CDS
mmu-miR-214-3p	NM_001165985	Ubap2l	ACAGCAGGCACAGACAGGCAGT#CTTCCAGTATGGGCCTGCTGTG#(((((((((.((.((.((.((.#)).)).)).)).))))))))).	2992	3014	0.82692	-23	1	5.14E-06	0.4	4	3.54	-7	17	22	9	CDS
mmu-miR-214-3p	NM_001165986	Ubap2l	ACAGCAGGCACAGACAGGCAGT#CTTCCAGTATGGGCCTGCTGTG#(((((((((.((.((.((.((.#)).)).)).)).))))))))).	2962	2984	0.92308	-23	1	5.14E-06	0.4	4.2	3.35	-7	17	22	9	CDS
mmu-miR-214-3p	NM_001165987	Ubap2l	ACAGCAGGCACAGACAGGCAGT#CTTCCAGTATGGGCCTGCTGTG#(((((((((.((.((.((.((.#)).)).)).)).))))))))).	2899	2921	0.82692	-23	1	5.14E-06	0.4	4.2	3.61	-7	17	22	9	CDS
mmu-miR-214-3p	NM_001166482	Mthfsd	ACAGCAGGCACAGACAGGCAGT#CTGCAGTTTAGCAGGATGGAGCTGTCTGTTCTTCCTGCTGC#.(((((((....((((((((((#.))))))))))...))))))).	1407	1448	0.84615	-27	1	2.94E-07	0.4	1.3	1.58	-12	17	22	10	3UTR

mmu-miR-214-3p	NM_001166553	Rnf145	ACAGCAGGCACAGACAGGCAGT#ACTGTGACAACAGGCACAGTTGCCACTCAAGCCAGGTCTCGTGCCTGTTCTGTG#((((((((((((((.(((...#.)))..)))).)))))))..)))).	1479	1533	0.84615	-27	1	0.0001345	0.5	-0	-0.1	-4	18	25	7	3UTR
mmu-miR-214-3p	NM_001166597	Dgkz	ACAGCAGGCACAGACAGGCAGT#GCCTCTGTGCCTCTGG#.(((.((((((((((..(((...#)))))))))))))))).	2725	2741	0.84615	-25	0	5.22E-07	0.4	3.7	4.42	-9	15	16	15	CDS
mmu-miR-214-3p	NM_001166597	Dgkz	ACAGCAGGCACAGACAGGCAGT#GCCTGCTGTATTCACTTGCCCGCTGC#.((((.((((((((.(((((...#)))))))))))........))).)))).	3924	3950	0.84615	-24	0	0.0033838	0.4	2.6	1.1	-7	16	26	9	3UTR
mmu-miR-214-3p	NM_001166629	Dynlt1a	ACAGCAGGCACAGACAGGCAGT#ACTGACCTGTCTCCCCGATATCCCTGGCTGAAGAGCCTTGCTGTG#(((((((((.((((((((((((#)))).)))))))...................).)))).)))))).	2075	2120	0.84615	-26	1	2.12E-05	0.6	-0	0.04	-5	21	45	7	3UTR
mmu-miR-214-3p	NM_001166629	Dynlt1a	ACAGCAGGCACAGACAGGCAGT#CTGCTTGGATGCTGCCATGCTC#..((((((((((..(((((((.#)))))))..)).)))).)))).	2427	2449	0.84615	-25	1	2.01E-05	0.5	0	0	-7	17	22	7	3UTR
mmu-miR-214-3p	NM_001166644	Zfp651	ACAGCAGGCACAGACAGGCAGT#ACTGGCCTGGTTGGGAAGGCCACGCCTCATCTGTGCACGCCTGGTTCTGAGGCCTGCTGG#.((((((((.(((((((((.((#.)))))))..)))).	5719	5779	0.92308	-30	1	6.56E-06	0.4	-1	-0.2	-5	19	25	8	3UTR
mmu-miR-214-3p	NM_001167949	Atp2b4	ACAGCAGGCACAGACAGGCAGT#GCTGCCCAGGGAAGTCTGTGCCTTTCTGC#.(((.((((((((((.((((((#))))))).......))))))))))..))).	5540	5569	0.92308	-28	0	0.0582564	0.4	2.8	3.66	-8	19	29	10	3UTR
mmu-miR-214-3p	NM_001167949	Atp2b4	ACAGCAGGCACAGACAGGCAGT#CTGCAGTTCGGCCTGCTGC#.((((((((.(.(((..((((.#)))).))).))))))))).	7691	7710	0.92308	-24	1	7.09E-05	0.5	0.3	1.97	-9	16	19	9	3UTR
mmu-miR-214-3p	NM_001167981	Galnt7	ACAGCAGGCACAGACAGGCAGT#ACCGCCTGGAACCTGCTGC#.(((((((......(((((.((#)).)))))...))))))).	2110	2129	0.96154	-21	1	0.0012447	0.6	2.1	2.56	-14	14	19	7	3UTR
mmu-miR-214-3p	NM_001168255	Slc45a4	ACAGCAGGCACAGACAGGCAGT#GCATGCCTGTTTGTTGCTGTC#((((((...(((((((((((((#)).)))))))))))))))))).	4542	4563	0.84615	-25	0	0.0001058	0.5	3.4	2.53	-9	19	21	17	3UTR
mmu-miR-214-3p	NM_001168255	Slc45a4	ACAGCAGGCACAGACAGGCAGT#GCGCCTGCTGTGGCTGTC#...((((.(((((.(((((.((#)))))))))))).)))).	1420	1438	0.92308	-25	0	2.36E-05	0.4	-1	-0.6	-10	16	18	12	CDS

mmu-miR-214-3p	NM_001168273	Sec24c	ACAGCAGGCACAGACAGGCAGT#CTGATGTAGCTGACATGTTTGTGCCACTGCTGG#.((((((((((((((.(((((#.)))).).))))))))))).	1594	1627	0.92308	-28	1	0.0028797	0.5	4.6	4.1	-4	20	26	10	CDS
mmu-miR-214-3p	NM_001168273	Sec24c	ACAGCAGGCACAGACAGGCAGT#TCTGTCATGTCCTGCTGA#.(((((((.(((((((((....#)))))).)))))))))).	3339	3357	1	-24	1	0.0162169	0.5	1.4	1.28	-5	16	18	10	3UTR
mmu-miR-214-3p	NM_001168621	Znrf1	ACAGCAGGCACAGACAGGCAGT#ACTGACCCTGCTGGGCCTGCTT#..(((((((.((((.((((((((#))))..))))))).))))))).	1180	1202	0.92308	-26	1	6.05E-05	0.4	3.8	3.13	-7	18	22	7	CDS
mmu-miR-214-3p	NM_001168621	Znrf1	ACAGCAGGCACAGACAGGCAGT#GCTGTCATGTCCTCTGAATTTGGACCCAGATTGCCAGTGCCTGGTGTG#(((.(((((((.((((((((((#)))))).))))........................))))))).))).	3605	3653	0.92308	-26	0	4.09E-05	0.4	-0	0.25	-10	20	48	7	3UTR
mmu-miR-214-3p	NM_001168622	Znrf1	ACAGCAGGCACAGACAGGCAGT#ACTGACCCTGCTGGGCCTGCTT#..(((((((.((((.((((((((#))))..))))))).))))))).	1084	1106	0.92308	-26	1	1.97E-05	0.4	3.8	3.03	-7	18	22	7	CDS
mmu-miR-214-3p	NM_001168622	Znrf1	ACAGCAGGCACAGACAGGCAGT#GCTGTCATGTCCTCTGAATTTGGACCCAGATTGCCAGTGCCTGGTGTG#(((.(((((((.((((((((((#)))))).))))........................))))))).))).	4620	4668	0.92308	-26	0	4.09E-05	0.4	-0	0.25	-10	20	48	7	3UTR
mmu-miR-214-3p	NM_001168623	Znrf1	ACAGCAGGCACAGACAGGCAGT#ACTGACCCTGCTGGGCCTGCTT#..(((((((.((((.((((((((#))))..))))))).))))))).	379	401	0.92308	-26	1	6.05E-05	0.4	3.8	3.13	-7	18	22	7	CDS
mmu-miR-214-3p	NM_001168623	Znrf1	ACAGCAGGCACAGACAGGCAGT#GCTGTCATGTCCTCTGAATTTGGACCCAGATTGCCAGTGCCTGGTGTG#(((.(((((((.((((((((((#)))))).))))........................))))))).))).	2804	2852	0.92308	-26	0	4.09E-05	0.4	-0	0.25	-10	20	48	7	3UTR
mmu-miR-214-3p	NM_001168624	C2cd4c	ACAGCAGGCACAGACAGGCAGT#GCTGCGTGGGGCTGTGCCTGGTGC#.((.(((((((((.((.(((((#))))).))...))))))))).)).	1187	1211	0.84615	-31	0	6.57E-06	0.3	0.1	0.04	-8	18	24	9	CDS
mmu-miR-214-3p	NM_001168624	C2cd4c	ACAGCAGGCACAGACAGGCAGT#GCTCAGGCCCGCCTGCGTGTGCGCCTGCTGG#.(((((((((((..(((((.((#.))..)))))..)))..)))))	1108	1139	0.92308	-28	1	1.37E-06	0.3	0.1	0.14	-6	18	26	8	CDS
mmu-miR-214-3p	NM_001168624	C2cd4c	ACAGCAGGCACAGACAGGCAGT#CTGTGTCTCTGTGTCTGACTGTG#((((((((((((((.(.((((.#)))).).))))))))))).)))).	4186	4209	0.84615	-27	1	0.0014278	0.4	-0	-0.4	-5	19	23	10	3UTR

mmu-miR-214-3p	NM_001170847	Rbm20	ACAGCAGGCACAGACAGGCAGT#GCTGCCACCGTCTCTGGAAGATGGATGGGAAGCCTGCTGA#.((((((((..((((.((((((#))))))...))))..................)))))))).	3709	3749	0.84615	-26	1	0.0001873	0.4	0.1	0.28	-8	18	40	8	3UTR
mmu-miR-214-3p	NM_001170847	Rbm20	ACAGCAGGCACAGACAGGCAGT#ATTGCAAGCTGTGTGGCCTGTTC#..((((((((.((.(((((((((#)))))...)))).))))))))).	3502	3525	0.84615	-23	1	3.60E-07	0.4	3.9	3.6	-7	18	23	9	CDS
mmu-miR-214-3p	NM_001170855	Trim36	ACAGCAGGCACAGACAGGCAGT#CTGTTTGTACTGTGTGATTCTTTGTTTTGTCTTGTCCTGCTGTG#(((((((((((((((((((.#)))))))).)))))......................)))))))).	2531	2575	0.92308	-28	1	0.0230877	0.5	3.5	3	-4	21	44	8	3UTR
mmu-miR-214-3p	NM_001170855	Trim36	ACAGCAGGCACAGACAGGCAGT#CTGCCTGCCTCTGCCTCCCAAGTGCTGG#.(((((((((.((.(((((((.#))))))).)).))))).......)))).	3949	3977	0.84615	-26	1	3.00E-05	0.4	3.1	3.14	-7	18	28	7	3UTR
mmu-miR-214-3p	NM_001170869	Pym1	ACAGCAGGCACAGACAGGCAGT#GCTGCTGATCTGGGTGTCTTGTTGA#.(((((((((((((..((((((#))))))..)))..)))))).)))).	932	957	0.92308	-23	1	0.003142	0.4	1.5	1	-5	19	25	6	3UTR
mmu-miR-214-3p	NM_001170912	Trim66	ACAGCAGGCACAGACAGGCAGT#GCTGTCCTGTGTGTGTATGCCATCTCCTTTCCTTCCCTGCTC#..((((((((((.(((((((((#)))).))))).))))....................)))))).	7530	7572	0.84615	-26	1	0.6097228	0.5	1.4	0.96	-6	19	42	6	3UTR
mmu-miR-214-3p	NM_001170912	Trim66	ACAGCAGGCACAGACAGGCAGT#GCTGCTATGCTGTGACCGCTGC#.((((.((((((((.((((((((#)))))).))))))).)))))).	3509	3531	0.84615	-25	0	2.33E-05	0.4	0.2	0.26	-7	19	22	7	CDS
mmu-miR-214-3p	NM_001170912	Trim66	ACAGCAGGCACAGACAGGCAGT#CTGCTGTCTGGCTCAGCTGG#.((((.(((.((((((.((((.#)))))))))))))..)))).	8379	8399	1	-25	0	8.20E-06	0.5	4.2	3.06	-7	17	20	13	3UTR
mmu-miR-214-3p	NM_001170913	Trim66	ACAGCAGGCACAGACAGGCAGT#GCTGTCCTGTGTGTGTATGCCATCTCCTTTCCTTCCCTGCTC#..((((((((((.(((((((((#)))).))))).))))....................)))))).	7593	7635	0.92308	-26	1	0.6097228	0.5	0.9	1.21	-6	19	42	6	3UTR
mmu-miR-214-3p	NM_001170913	Trim66	ACAGCAGGCACAGACAGGCAGT#GCTGCTATGCTGTGACCGCTGC#.((((.((((((((.((((((((#)))))).))))))).)))))).	3547	3569	0.84615	-25	0	2.33E-05	0.4	0.3	0.27	-7	19	22	7	CDS
mmu-miR-214-3p	NM_001170913	Trim66	ACAGCAGGCACAGACAGGCAGT#CTGCTGTCTGGCTCAGCTGG#.((((.(((.((((((.((((.#)))))))))))))..)))).	8442	8462	1	-25	0	8.20E-06	0.5	4.2	3.14	-7	17	20	13	3UTR

mmu-miR-214-3p	NM_001170985	Kirrel	ACAGCAGGCACAGACAGGCAGT#GCTGCTGACACCACCCGTGGGCCAGTAAAGAGGACTGAGGTCTGTGGCTTGTTGTA#(((((((((((((((...((((#.))))..))))))).)))))))).	6306	6362	1	-25	1	0.000362	0.5	1.7	2.68	-4	19	24	8	3UTR
mmu-miR-214-3p	NM_001172096	Cacul1	ACAGCAGGCACAGACAGGCAGT#GCTCCCTCTCTGGTCCCCTGCTGC#.(((((((..((((.(((.(((#))).))).))))....))))))).	4974	4998	1	-23	1	0.0092343	0.4	3.5	3.18	-7	17	24	7	3UTR
mmu-miR-214-3p	NM_001172096	Cacul1	ACAGCAGGCACAGACAGGCAGT#ACTGACCTGCTGTCATAACTGCA#...((((..((((.((((((((#)))).))))))))).....)))).	3814	3837	0.92308	-20	0	0.0022807	0.6	0.5	1.54	-11	16	23	8	3UTR
mmu-miR-214-3p	NM_001172097	Cacul1	ACAGCAGGCACAGACAGGCAGT#GCTCCCTCTCTGGTCCCCTGCTGC#.(((((((..((((.(((.(((#))).))).))))....))))))).	4835	4859	1	-23	1	0.0092343	0.4	3.5	3.18	-7	17	24	7	3UTR
mmu-miR-214-3p	NM_001172097	Cacul1	ACAGCAGGCACAGACAGGCAGT#ACTGACCTGCTGTCATAACTGCA#...((((..((((.((((((((#)))).))))))))).....)))).	3675	3698	1	-20	0	0.0022807	0.6	0.2	0.4	-11	16	23	8	3UTR
mmu-miR-214-3p	NM_001173550	C5ar1	ACAGCAGGCACAGACAGGCAGT#ACTGTAGACCTGTGCTGTTGTG#((((((.((((((....(((((#)))))....)))))))))))).	1880	1902	1	-22	0	0.0059625	0.6	1	1.65	-7	17	22	12	3UTR
mmu-miR-214-3p	NM_001173550	C5ar1	ACAGCAGGCACAGACAGGCAGT#TGCGTGTCCCTGGCCTACATCAACTGCTGTG#(((((((((.((((((.(((..#))).)))).))))))).........)	1046	1077	0.84615	-22	1	0.0164258	0.5	-0	-0.2	-5	18	31	6	CDS
mmu-miR-214-3p	NM_001173550	C5ar1	ACAGCAGGCACAGACAGGCAGT#ACTGCCTGTCCTGTTC#..((((((.....(((((((((#)))))))))))))))).	454	470	0.84615	-21	1	0.0048166	0.5	0.2	0.14	-15	15	16	15	CDS
mmu-miR-214-3p	NM_001173553	Cdc14a	ACAGCAGGCACAGACAGGCAGT#GCTGCTTGCCCGGAGGATGTCCCTGCC#...(((((.(((..((((((((#))))))))).........))).))))).	2279	2306	0.84615	-20	0	0.0001851	0.5	1.1	0.73	-10	16	27	8	3UTR
mmu-miR-214-3p	NM_001177412	Usp35	ACAGCAGGCACAGACAGGCAGT#GCCGCGTGCTGCGCCTGCTGC#.((((((((.((((.((.(((.((#)).))).))))).)))))))).	616	637	0.84615	-28	1	2.34E-05	0.2	0.1	-0.2	-6	17	21	8	CDS
mmu-miR-214-3p	NM_001177412	Usp35	ACAGCAGGCACAGACAGGCAGT#GCTGCCTGGAGCAGCTGGCAGAGCTGGTGCACTGCATGG#.((((((((((...((((((((#))))))))).................)))).)))).)).	1471	1510	0.92308	-25	1	0.0002683	0.4	2.9	2.14	-9	18	39	8	CDS
mmu-miR-214-3p	NM_001177412	Usp35	ACAGCAGGCACAGACAGGCAGT#TGAGTGTCTGCACCTGCTGTG#((((((((..((((((..(((..#))..))))))..)))))))).	3917	3938	1	-25	1	0.001053	0.6	2.5	1.86	-6	16	21	8	3UTR

mmu-miR-214-3p	NM_001177653	H2bc6	ACAGCAGGCACAGACAGGCAGT#ACTTCAGCCTGACTGTCTGCTGTGCACTGGCATGTGTACCTCCAAGCCTGCTGC#.((((((((((((.((((((((#.)))))))))))))....................	2323	2377	1	-30	1	0.004505	0.5	3.2	2.77	-5	20	44	12	3UTR
mmu-miR-214-3p	NM_001177656	Grin1	ACAGCAGGCACAGACAGGCAGT#GCTGCTGGTGGGGCTGTCAGTTCATGTGGTGGCCGTGATGCTGTACCTGCTGG#.(((((((.((((.(((((...#.))).))...)))).))))))).	2052	2105	0.92308	-24	1	6.29E-05	0.5	4.2	3.8	-6	16	23	7	CDS
mmu-miR-214-3p	NM_001177657	Grin1	ACAGCAGGCACAGACAGGCAGT#GCTGCTGGTGGGGCTGTCAGTTCATGTGGTGGCCGTGATGCTGTACCTGCTGG#.(((((((.((((.(((((...#.))).))...)))).))))))).	1989	2042	0.84615	-24	1	6.29E-05	0.5	5.4	4.08	-6	16	23	7	CDS
mmu-miR-214-3p	NM_001177751	Tsc22d1	ACAGCAGGCACAGACAGGCAGT#ACTGGCTGCTGCTGCTGCTGTC#((((((.((((((.(((.((((#)))).)))))).))))))))).	566	588	0.84615	-26	0	2.61E-05	0.5	0.6	1.37	-6	19	22	9	3UTR
mmu-miR-214-3p	NM_001177751	Tsc22d1	ACAGCAGGCACAGACAGGCAGT#GCTGCCTGCTTGTTTGTA#...(((((((.((.((((((((#)))))))))))))))))).	81	99	1	-23	0	7.60E-05	0.6	0.6	0.03	-10	17	18	17	5UTR
mmu-miR-214-3p	NM_001177884	Nsd2	ACAGCAGGCACAGACAGGCAGT#TTTGTCTGATGCTTGCA#...((((((((((((((....#)))))))).))))))).	1798	1815	1	-22	0	0.0192348	0.5	0	0	-7	15	17	8	CDS
mmu-miR-214-3p	NM_001177884	Nsd2	ACAGCAGGCACAGACAGGCAGT#ACTGCCTGTCCATGAGATGTCCCGTGAGGTC#((..((((.(((((((((((((#))))))))))).......))).)).)	5178	5209	0.84615	-22	0	0.0002265	0.5	0	0	-10	19	31	10	3UTR
mmu-miR-214-3p	NM_001177900	Rd3	ACAGCAGGCACAGACAGGCAGT#GCTGCTACTGCTGTCCCTGCC#...(((((.((((.((((((((#)))))..))))))).))))).	2767	2788	0.84615	-24	0	0.0021808	0.4	-0	-0	-8	17	21	7	3UTR
mmu-miR-214-3p	NM_001177900	Rd3	ACAGCAGGCACAGACAGGCAGT#CTGTCTGTCTGTCTGTC#...((((..(((((((((((((.#))))))))))))))))).	3203	3220	1	-24	0	0.015112	0.5	0.3	0.2	-10	16	17	16	3UTR
mmu-miR-214-3p	NM_001177900	Rd3	ACAGCAGGCACAGACAGGCAGT#GCCTGCGAACCTGCTGC#.(((((((....(.(((((...#))))))...))))))).	1512	1529	1	-23	1	0.0005707	0.4	0.3	0	-13	13	17	7	3UTR
mmu-miR-214-3p	NM_001177902	Zfp518b	ACAGCAGGCACAGACAGGCAGT#AGTGCCTGCTGCTCCACCAGTCACTGGATGTGGGAGGATGCCTGCA#...(((((((((.((((((.(#).)))))))))).........................))))))).	543	589	0.92308	-24	0	4.88E-05	0.5	0.2	0.26	-7	17	46	9	5UTR

mmu-miR-214-3p	NM_001177902	Zfp518b	ACAGCAGGCACAGACAGGCAGT#CTGCCTGCTGCTCTGTGGCCATGTG#...(((((((((((((((((.#)))))))....)))))).)).))).	5749	5774	0.92308	-24	0	0.0002458	0.5	2.7	1.59	-7	18	25	7	3UTR
mmu-miR-214-3p	NM_001177902	Zfp518b	ACAGCAGGCACAGACAGGCAGT#TTGTCCTGTCTGAGAGGACTAGGTGTCAGCTGG#.((((.((((((((((((((.#))).)))))))..........)))))).)))).	3580	3613	0.92308	-24	0	4.37E-06	0.4	-0	0.26	-6	19	33	7	CDS
mmu-miR-214-3p	NM_001190483	Pcsk5	ACAGCAGGCACAGACAGGCAGT#GCTGCAGCCGCCCGGGACGGCGGGACCTGCTGTGCGTGCTGG#.(((((.((((((.((((....#.)))))))))).)))))).	502	544	0.92308	-29	0	3.46E-06	0.3	0.5	0.06	-6	15	18	10	CDS
mmu-miR-214-3p	NM_001190483	Pcsk5	ACAGCAGGCACAGACAGGCAGT#CTGCCTGATCTGCTCATCAGCTTGGACCTGCCTGGCCTGTC#((((((((((((((((((((.#)))))))).)))).................))))))..)))).	4832	4873	0.92308	-27	1	3.01E-05	0.4	2.3	3.01	-4	21	41	7	CDS
mmu-miR-214-3p	NM_001190852	Pdlim5	ACAGCAGGCACAGACAGGCAGT#GCTGCCACTGTGTCTGCTGC#.((((((((((((...((((((#)))))).)))))))))))).	972	992	1	-32	1	0.0008272	0.4	0.8	0.62	-6	18	20	12	CDS
mmu-miR-214-3p	NM_001190853	Pdlim5	ACAGCAGGCACAGACAGGCAGT#GCTTGGAAAGAGTTGTAATAAAGTTGTCTGTACCGTTGC#.((((.((.((((((((.....#.))))))))	1774	1813	0.84615	-17	0	0.0230703	0.6	4	3.62	-7	14	17	8	3UTR
mmu-miR-214-3p	NM_001190854	Pdlim5	ACAGCAGGCACAGACAGGCAGT#ACTGTCTCTCTAATTGTTCGGTCTGACTGCTGCCTGTTGA#.((((((((((((.(((((...#.))))).))).))))))))).	4078	4118	0.92308	-27	1	0.0010799	0.6	3.8	3.14	-5	17	21	9	3UTR
mmu-miR-214-3p	NM_001190854	Pdlim5	ACAGCAGGCACAGACAGGCAGT#TGTGTGTGTGTGTGTGCG#...(((.(((((.(((.(((..#))).))).))))).))).	3029	3047	0.84615	-23	0	0.0060812	0.5	0.2	0.79	-9	14	18	5	3UTR
mmu-miR-214-3p	NM_001190855	Pdlim5	ACAGCAGGCACAGACAGGCAGT#ACTGTCTCTCTAATTGTTCGGTCTGACTGCTGCCTGTTGA#.((((((((((((.(((((...#.))))).))).))))))))).	4210	4250	0.92308	-27	1	0.0010799	0.6	3.8	3.14	-5	17	21	9	3UTR
mmu-miR-214-3p	NM_001190856	Pdlim5	ACAGCAGGCACAGACAGGCAGT#GCTGCCACTGTGTCTGCTGC#.((((((((((((...((((((#)))))).)))))))))))).	852	872	1	-32	1	0.0008272	0.4	0.9	1.23	-6	18	20	12	CDS
mmu-miR-214-3p	NM_001190857	Pdlim5	ACAGCAGGCACAGACAGGCAGT#GCTTGGAAAGAGTTGTAATAAAGTTGTCTGTACCGTTGC#.((((.((.((((((((.....#.))))))))	1723	1762	0.92308	-17	0	0.0230703	0.6	2.4	3	-7	14	17	8	3UTR

mmu-miR-214-3p	NM_001194921	Cldn18	ACAGCAGGCACAGACAGGCAGT#GCAATCATTGGTGTGTCTGTGTTTGCC#...(((((((((((((.(((((#.)))).).))))))))))))).	445	472	0.92308	-25	0	0.0006784	0.5	0	-0.1	-7	18	22	13	CDS
mmu-miR-214-3p	NM_001194921	Cldn18	ACAGCAGGCACAGACAGGCAGT#CTGTGTGTCTGGCATAGCTA#..(((..(((.((((((.((((.#)))).))))))))...))).	1901	1921	0.84615	-21	0	9.42E-05	0.5	-0	1.22	-10	15	20	8	3UTR
mmu-miR-214-3p	NM_001194922	Cldn18	ACAGCAGGCACAGACAGGCAGT#ATTGGTGTGTCTGTGTTTGCC#...((((((((((((((.(((((#)))).).))))))))))))).	514	535	0.92308	-24	0	0.0006784	0.5	0.2	-0.1	-7	18	21	13	CDS
mmu-miR-214-3p	NM_001194922	Cldn18	ACAGCAGGCACAGACAGGCAGT#CTGTCCCTCCTGGGTCTGGCCGGCTGC#.((((.(((.((((((((.((.#.)))))..)))))))).)))).	151	178	0.92308	-23	0	6.64E-07	0.4	0.1	0.18	-7	17	22	8	CDS
mmu-miR-214-3p	NM_001194922	Cldn18	ACAGCAGGCACAGACAGGCAGT#CTGTGTGTCTGGCATAGCTA#..(((..(((.((((((.((((.#)))).))))))))...))).	1976	1996	0.92308	-21	0	9.42E-05	0.5	1.1	1.73	-10	15	20	8	3UTR
mmu-miR-214-3p	NM_001194923	Cldn18	ACAGCAGGCACAGACAGGCAGT#GCAATCATTGGTGTGTCTGTGTTTGCC#...(((((((((((((.(((((#.)))).).))))))))))))).	445	472	0.92308	-25	0	0.0006784	0.5	0	-0.1	-7	18	22	13	CDS
mmu-miR-214-3p	NM_001194923	Cldn18	ACAGCAGGCACAGACAGGCAGT#CTGTGTGTCTGGCATAGCTA#..(((..(((.((((((.((((.#)))).))))))))...))).	1913	1933	0.92308	-21	0	9.42E-05	0.5	0.6	1.18	-10	15	20	8	3UTR
mmu-miR-214-3p	NM_001195390	Commd7	ACAGCAGGCACAGACAGGCAGT#GCTGCCTGATTCTTCTGCAAGCCCCACTGG#.(((..(((.((((((((((((#))))))))).....))))...)))...)	904	934	0.84615	-23	0	0.0083119	0.4	0.2	0.01	-11	18	30	8	3UTR
mmu-miR-214-3p	NM_001195539	Dclk1	ACAGCAGGCACAGACAGGCAGT#ATTGTCTCTCTGTGTCTGTCTGTC#((((((((((((((.(((((((#))))))).)))))))))))).)))).	5323	5347	0.84615	-28	1	0.1847508	0.6	0.1	-0.1	-5	21	24	10	3UTR
mmu-miR-214-3p	NM_001195539	Dclk1	ACAGCAGGCACAGACAGGCAGT#GCTGCTTGCCCATTACCTGCTGC#.(((((((......((((((((#))))))))).......))))))).	2410	2433	0.92308	-23	1	0.0017245	0.4	2.4	0.39	-14	15	23	8	3UTR
mmu-miR-214-3p	NM_001195540	Dclk1	ACAGCAGGCACAGACAGGCAGT#GCTGCCTTGTCTTTAGCTGAAACAGTAAACATCTGTGAGGATGCTCTGCTGG#.((((((((((((((((((((#))))))).))))).........................	2253	2305	0.80769	-26	1	0.0001099	0.6	1.8	1.88	-4	21	52	7	3UTR
mmu-miR-214-3p	NM_001195540	Dclk1	ACAGCAGGCACAGACAGGCAGT#ACTGTCTGTGTACATTTGTGCATTTGTGCCTATCTGC#.(((.((((((((((((((((#))))))))))..............)))))))..))).	4299	4336	0.92308	-24	0	0.0020753	0.5	0.4	0.64	-8	20	37	9	3UTR

mmu-miR-214-3p	NM_001195540	Dclk1	ACAGCAGGCACAGACAGGCAGT#GTGAGTGTGTGTCTGGTGG#.((.((((((((.((..((...#))..)).)))))))).)).	28	47	0.84615	-22	0	0.0104424	0.4	0.6	1.34	-8	14	19	8	5UTR
mmu-miR-214-3p	NM_001195565	Camta1	ACAGCAGGCACAGACAGGCAGT#ACTAGATGAAGGCTCCTGGGACTGTGCCTGTGCTGG#.(((((((((((.((((.(((#.)))))))...))))))))..)))).	1445	1481	0.84615	-27	1	0.0005509	0.4	-0	-0.3	-5	19	26	8	3UTR
mmu-miR-214-3p	NM_001195565	Camta1	ACAGCAGGCACAGACAGGCAGT#CTGCAACACTGCCTCTGGCCTGCC#...((((((.((((..((((((#.)))))))))))))))))).	1377	1401	1	-27	0	0.0003043	0.4	-0	0.3	-9	16	18	16	3UTR
mmu-miR-214-3p	NM_001195816	Pld5	ACAGCAGGCACAGACAGGCAGT#GCTCCTGACTCTGTGCTC#......(((((((((((.(((#)))))))..)))))))).	2994	3012	0.84615	-19	0	0.0014394	0.4	0.6	1.07	-16	15	18	8	3UTR
mmu-miR-214-3p	NM_001195816	Pld5	ACAGCAGGCACAGACAGGCAGT#AGTGCATCGTGATCTTCGCTCTGGTCTGCTGC#.((((((((.((((..((....#.))..)))))))))))).	333	365	1	-19	1	0.0007676	0.5	1.2	0.83	-6	14	18	12	CDS
mmu-miR-214-3p	NM_001197046	Cep43	ACAGCAGGCACAGACAGGCAGT#ACAGCCATGGTTTGTGCCACTATGTGTCTGTGGCTGCTC#..(((((.((((((((((((..#.))))...))))))))).))))).	1582	1621	1	-27	0	0.0014486	0.5	-0	-0.1	-7	17	26	8	3UTR
mmu-miR-214-3p	NM_001197046	Cep43	ACAGCAGGCACAGACAGGCAGT#GCTGGAATGTCCGCTCCTGCTGG#.(((((((..(.((((..((((#))))...)))).)..))))))).	1350	1373	0.92308	-23	1	0.0001657	0.5	0.8	0.85	-9	16	23	7	3UTR
mmu-miR-214-3p	NM_001198565	Sulf1	ACAGCAGGCACAGACAGGCAGT#GCTGCTTGCCGTGCTGGGCACACAGCTGCTGG#.((((((((((.(.((((((((#)))))))))).))))......	505	537	0.84615	-26	1	1.29E-08	0.3	1.5	1.46	-8	19	32	9	CDS
mmu-miR-214-3p	NM_001198565	Sulf1	ACAGCAGGCACAGACAGGCAGT#TCTTTCTGTGCCTGCA#...(((((((((((.(((....#))).))))))))))).	2768	2784	1	-24	0	0.035048	0.5	5.1	4.23	-7	14	16	11	CDS
mmu-miR-214-3p	NM_001198566	Sulf1	ACAGCAGGCACAGACAGGCAGT#GCTGCTTGCCGTGCTGGGCACACAGCTGCTGG#.((((((((((.(.((((((((#)))))))))).))))......	410	442	0.84615	-26	1	1.29E-08	0.3	1.5	1.46	-8	19	32	9	CDS
mmu-miR-214-3p	NM_001198566	Sulf1	ACAGCAGGCACAGACAGGCAGT#TCTTTCTGTGCCTGCA#...(((((((((((.(((....#))).))))))))))).	2673	2689	1	-24	0	0.035048	0.5	5.1	4.23	-7	14	16	11	CDS
mmu-miR-214-3p	NM_001198570	Abi2	ACAGCAGGCACAGACAGGCAGT#CTGCCTCTGCCTGCCGAGTGCTGG#.((((((((((((..(((((.#)))))))))..))))...))))).	2244	2268	0.84615	-24	1	6.55E-05	0.4	-0	-0	-5	18	24	9	3UTR

mmu-miR-214-3p	NM_001198570	Abi2	ACAGCAGGCACAGACAGGCAGT#GCCTGCTGTGTTTGG#....(((((((((.(((((...#)))))))))))))).	3309	3324	1	-24	0	0.0042277	0.7	0.6	0.51	-10	14	15	14	3UTR
mmu-miR-214-3p	NM_001198570	Abi2	ACAGCAGGCACAGACAGGCAGT#GCCCCTGCTGGCTCTGCTGG#.((((((((.(((.((((..((#)).))))))))).)))))).	1033	1053	0.84615	-23	1	5.68E-07	0.4	2.6	2.74	-6	17	20	9	CDS
mmu-miR-214-3p	NM_001198571	Abi2	ACAGCAGGCACAGACAGGCAGT#CTGCCTCTGCCTGCCGAGTGCTGG#.((((((((((((..(((((.#)))))))))..))))...))))).	3315	3339	0.84615	-24	1	6.55E-05	0.4	-0	-0	-5	18	24	9	3UTR
mmu-miR-214-3p	NM_001198571	Abi2	ACAGCAGGCACAGACAGGCAGT#GCCTGCTGTGTTTGG#....(((((((((.(((((...#)))))))))))))).	4380	4395	1	-24	0	0.0042277	0.7	0.6	0.51	-10	14	15	14	3UTR
mmu-miR-214-3p	NM_001198587	Nrxn3	ACAGCAGGCACAGACAGGCAGT#GCTGCCTGGCCTCTGTGGACTTGAATGGACGCCTGCCTGA#.((((((((((((((((((((#))))))))...)))))..............)))))).)).	3730	3770	0.92308	-28	1	0.0070194	0.4	4.3	3.87	-4	21	40	8	CDS
mmu-miR-214-3p	NM_001198587	Nrxn3	ACAGCAGGCACAGACAGGCAGT#GCTGCTCCTGTATTTGGATGATGGTGGTGTCTGTGACTTCCTCTGTCTCTCCCTGGTGG#.((.((((((((((((((((((#))))))).....................))))))))................)))).)).	866	925	0.92308	-23	0	0.0025306	0.4	3.5	3.3	-7	20	59	8	CDS
mmu-miR-214-3p	NM_001198587	Nrxn3	ACAGCAGGCACAGACAGGCAGT#CTGCTCCTCTGTGCTCTGA#....((((((((((..(((((.#)))))..))))))).))).	649	668	0.84615	-23	0	0.0043441	0.4	-0	0.52	-11	15	19	7	5UTR
mmu-miR-214-3p	NM_001198635	Map7	ACAGCAGGCACAGACAGGCAGT#GCCACCTGAGCCTGCTGC#.((((((((.(((...(((...#)))..))).)))))))).	1350	1368	1	-25	1	0.0002441	0.3	0.7	0.25	-7	14	18	8	CDS
mmu-miR-214-3p	NM_001198635	Map7	ACAGCAGGCACAGACAGGCAGT#ACTGTCAGCCTCTCCTGTCTTATCATTCTTCACTCCTGCTGCTCAGCCCTGCTGTA#(((((((((.(((.((((.(((#.))))))))))....))).)))))).	3446	3502	0.92308	-23	1	0.0017568	0.6	0.1	0.47	-6	19	26	10	3UTR
mmu-miR-214-3p	NM_001198955	Gm7694	ACAGCAGGCACAGACAGGCAGT#GCTGGCTGCTGCCTGCAGCTTTGGCTGCTGG#.((((((.((.((.((((((((#.))))))))...)).)).))))))	1113	1144	0.90385	-29	1	1.20E-08	0.4	-0	0.03	-10	18	25	8	3UTR
mmu-miR-214-3p	NM_001198955	Gm7694	ACAGCAGGCACAGACAGGCAGT#AGTGCTTCTTTGTGCTTGTGCTGC#.((((((((((((.(((((.(#).))))).))))))))..))))).	1904	1928	0.84615	-23	1	7.49E-05	0.5	-0	-0.4	-5	19	24	8	3UTR

mmu-miR-214-3p	NM_001199217	Slc38a3	ACAGCAGGCACAGACAGGCAGT#ATTGCCACTGGCCTGCTT#..(((((((.(((...((((((#)))))).)))))))))).	1375	1393	0.84615	-24	1	0.0002361	0.4	4.7	3.68	-8	16	18	10	CDS
mmu-miR-214-3p	NM_001199218	Slc38a3	ACAGCAGGCACAGACAGGCAGT#ACTGCTGCTGAGGCCTTCTGTA#((((.((((.(((.(((.(((((#))))))))))..)))).)))).	972	994	1	-23	0	5.77E-05	0.4	3.4	4.02	-10	18	22	10	CDS
mmu-miR-214-3p	NM_001199296	Acly	ACAGCAGGCACAGACAGGCAGT#GCTGCTTGTGGGTGTGGACGAAAAGCTGA#.((((..((((..((((((((#))))))))..))))........)))	681	710	0.92308	-22	0	0.0502609	0.5	0.8	1.15	-12	17	29	9	CDS
mmu-miR-214-3p	NM_001199296	Acly	ACAGCAGGCACAGACAGGCAGT#TTGCTCTGCTACCTGCTGTA#((((((((...(((.((((((((.#)))).))))).)))))))).	3783	3803	0.84615	-22	1	0.0122395	0.6	4.4	3.76	-10	17	20	8	3UTR
mmu-miR-214-3p	NM_001200038	Rubcn	ACAGCAGGCACAGACAGGCAGT#GCTGTTTGTGTACAACCTGTGTTTAGAACTGGATTCTCACCTGCTGTA#(((((((((((((((((((((#)))))))))).......)))))................))))))))	5214	5262	0.92308	-25	1	0.0110986	0.5	0.2	0.31	-4	22	48	9	3UTR
mmu-miR-214-3p	NM_001201341	Msi2	ACAGCAGGCACAGACAGGCAGT#GCTGCCTCAGGTGACCAGAAAAAGGTTGGCTTGTCAGACTCTGCTGC#.(((((((....(((((((...#.)))))))....))))))).	543	590	0.84615	-22	1	0.0012862	0.4	2.4	2.79	-12	14	20	7	CDS
mmu-miR-214-3p	NM_001201367	Tmem200b	ACAGCAGGCACAGACAGGCAGT#GCCTGTTCGTGTTCATCTGTGCCAACACATTGCTC#..((((((((((((((((...#)))))).........))))))))).......)))).	319	354	0.92308	-23	1	0.0014233	0.4	2.7	1.88	-5	17	35	8	CDS
mmu-miR-214-3p	NM_001201367	Tmem200b	ACAGCAGGCACAGACAGGCAGT#GCTGCCTGCCCTGCTC#..((((((....(.((((((((#)))))))))))))))).	584	600	1	-22	1	6.83E-06	0.3	0.7	0.87	-14	15	16	15	CDS
mmu-miR-214-3p	NM_001205068	Jmjd4	ACAGCAGGCACAGACAGGCAGT#TGCCTGGCTGAGCCTGCA#...((((((.(((.((((((..#)))))).))).)))))).	3806	3824	0.92308	-28	0	4.88E-05	0.5	-0	0.12	-9	15	18	6	3UTR
mmu-miR-214-3p	NM_001205068	Jmjd4	ACAGCAGGCACAGACAGGCAGT#GCTGTGGCTGAGAACAGGCCTCTGAGAGCCTGCTGTT#(((((((((.((((..(((.((#.))..)))))))...))))))))).	2519	2556	0.84615	-25	1	1.26E-05	0.4	0.2	0.28	-6	18	25	9	3UTR
mmu-miR-214-3p	NM_001205068	Jmjd4	ACAGCAGGCACAGACAGGCAGT#GCTGCCTAACCTGCCCTGCGTGTT#(((((((..(((..(((((((#)))))))...))).))))).))).	352	376	0.92308	-23	1	0.0011119	0.4	3.2	2.29	-8	18	24	7	CDS

mmu-miR-214-3p	NM_001205345	Sun2	ACAGCAGGCACAGACAGGCAGT#TGCCTCTGACGACTGTTGTCTGTGCCAACCTAAGAGATTCCCAGTGCTGG#.(((((((((((((((.(((((#.))))))))))))))).................))))).	2954	3004	1	-29	1	5.78E-05	0.5	2	1.48	-4	20	40	15	3UTR
mmu-miR-214-3p	NM_001205346	Sun2	ACAGCAGGCACAGACAGGCAGT#TGCCTCTGACGACTGTTGTCTGTGCCAACCTAAGAGATTCCCAGTGCTGG#.(((((((((((((((.(((((#.))))))))))))))).................))))).	2948	2998	1	-29	1	5.78E-05	0.5	2	1.43	-4	20	40	15	3UTR
mmu-miR-214-3p	NM_001205361	Dcun1d1	ACAGCAGGCACAGACAGGCAGT#CTGCTTGTGTGTTTACGTGTGTTGC#.(((((.(((((.((((((((.#)))))))).)))....)).))))).	3997	4022	0.92308	-24	0	0.0421748	0.5	4.8	4.03	-7	18	25	8	3UTR
mmu-miR-214-3p	NM_001205361	Dcun1d1	ACAGCAGGCACAGACAGGCAGT#TGCCTTGCTCTCCTGCTGA#.(((((((...(((((((((..#))))).).)))))))))).	2276	2295	1	-21	1	0.0100913	0.6	0.9	1.31	-9	16	19	10	3UTR
mmu-miR-214-3p	NM_001205361	Dcun1d1	ACAGCAGGCACAGACAGGCAGT#GCCATGTTGGCTGCTGA#.((((((.((...((((((...#))).))))).)))))).	2039	2056	0.92308	-20	1	7.09E-05	0.5	0.1	0.48	-14	14	17	6	3UTR
mmu-miR-214-3p	NM_001205362	Dcun1d1	ACAGCAGGCACAGACAGGCAGT#CTGCTTGTGTGTTTACGTGTGTTGC#.(((((.(((((.((((((((.#)))))))).)))....)).))))).	3957	3982	1	-24	0	0.0421748	0.5	4.1	3.84	-7	18	25	8	3UTR
mmu-miR-214-3p	NM_001205362	Dcun1d1	ACAGCAGGCACAGACAGGCAGT#TGCCTTGCTCTCCTGCTGA#.(((((((...(((((((((..#))))).).)))))))))).	2236	2255	1	-21	1	0.0100913	0.6	1	0.9	-9	16	19	10	3UTR
mmu-miR-214-3p	NM_001205362	Dcun1d1	ACAGCAGGCACAGACAGGCAGT#GCCATGTTGGCTGCTGA#.((((((.((...((((((...#))).))))).)))))).	1999	2016	0.92308	-20	1	7.09E-05	0.5	0.8	0.5	-14	14	17	6	3UTR
mmu-miR-214-3p	NM_001205391	Atg16l1	ACAGCAGGCACAGACAGGCAGT#GCTCCCTGTCTGGCAGTAATGCTGG#.(((((.((.((((((((.(((#))).)))))))))).....))))).	1315	1340	0.92308	-24	0	2.71E-06	0.6	1.9	3.62	-7	18	25	10	CDS
mmu-miR-214-3p	NM_001205391	Atg16l1	ACAGCAGGCACAGACAGGCAGT#ACTGCCTGGACCTGCGCACCAAACTGCA#...((((((.(((.((((((((#))))))))...))).)).....)))).	619	647	1	-23	0	0.0002909	0.4	2.2	2.86	-9	17	28	8	CDS
mmu-miR-214-3p	NM_001205392	Atg16l1	ACAGCAGGCACAGACAGGCAGT#GCTCCCTGTCTGGCAGTAATGCTGG#.(((((.((.((((((((.(((#))).)))))))))).....))))).	1210	1235	0.92308	-24	0	2.71E-06	0.6	1.9	3.62	-7	18	25	10	CDS
mmu-miR-214-3p	NM_001205392	Atg16l1	ACAGCAGGCACAGACAGGCAGT#ACTGCCTGGACCTGCGCACCAAACTGCA#...((((((.(((.((((((((#))))))))...))).)).....)))).	619	647	1	-23	0	0.0002909	0.4	2.2	2.86	-9	17	28	8	CDS

mmu-miR-214-3p	NM_001205392	Atg16l1	ACAGCAGGCACAGACAGGCAGT#GCTCAGCCGAGGGTTCTCTTTATGTCTGGAGTGTGCTGA#.(((((.((.((((((((((((#)))..)))..............)))))).)).))))).	1786	1825	0.92308	-22	0	0.0001774	0.5	3	3.26	-7	19	39	6	CDS
mmu-miR-214-3p	NM_001242389	Trip10	ACAGCAGGCACAGACAGGCAGT#CCTGTGTAACCTGCTGTC#((((((((...(.((((((....#)))))).)..)))))))).	2020	2038	1	-20	1	0.0523865	0.4	1.5	0.62	-10	14	18	8	3UTR
mmu-miR-214-3p	NM_001242389	Trip10	ACAGCAGGCACAGACAGGCAGT#GCCGCCGAGTCTGTGGATGCTA#..((((..(((((((.(((.((#)).)))..)))))))..)))).	859	881	1	-24	0	4.63E-05	0.5	1	2.88	-9	16	22	7	CDS
mmu-miR-214-3p	NM_001242390	Trip10	ACAGCAGGCACAGACAGGCAGT#CCTGTGTAACCTGCTGTC#((((((((...(.((((((....#)))))).)..)))))))).	2017	2035	1	-20	1	0.0523865	0.4	1.5	0.62	-10	14	18	8	3UTR
mmu-miR-214-3p	NM_001242390	Trip10	ACAGCAGGCACAGACAGGCAGT#GCCGCCGAGTCTGTGGATGCTA#..((((..(((((((.(((.((#)).)))..)))))))..)))).	859	881	1	-24	0	4.63E-05	0.5	1	2.88	-9	16	22	7	CDS
mmu-miR-214-3p	NM_001242391	Trip10	ACAGCAGGCACAGACAGGCAGT#CCTGTGTAACCTGCTGTC#((((((((...(.((((((....#)))))).)..)))))))).	1849	1867	1	-20	1	0.0523865	0.4	1.5	0.62	-10	14	18	8	3UTR
mmu-miR-214-3p	NM_001242391	Trip10	ACAGCAGGCACAGACAGGCAGT#GCCGCCGAGTCTGTGGATGCTA#..((((..(((((((.(((.((#)).)))..)))))))..)))).	859	881	1	-24	0	4.63E-05	0.5	1	2.88	-9	16	22	7	CDS
mmu-miR-214-3p	NM_001242419	Susd6	ACAGCAGGCACAGACAGGCAGT#AGTGTCTGCTGTCCCTGCTGG#.(((((((.((((.((((((.(#).)))))))))).))))))).	3026	3047	1	-28	1	1.06E-05	0.4	0.2	0.44	-6	18	21	10	3UTR
mmu-miR-214-3p	NM_001242419	Susd6	ACAGCAGGCACAGACAGGCAGT#GCTAGAGCCTTCTCTGTGGGGCCTGCTGG#.(((((((((((((.((((...#.))))..)))))...))))))))).	4563	4592	0.92308	-28	1	6.38E-09	0.5	-1	0.44	-5	17	24	8	3UTR
mmu-miR-214-3p	NM_001242419	Susd6	ACAGCAGGCACAGACAGGCAGT#GCCTGCTGTGCCACTGCTC#..(((((((((((.(((((...#))))))))))))..))))).	2416	2435	1	-28	1	0.0061675	0.4	1	0.74	-6	16	19	12	3UTR
mmu-miR-214-3p	NM_001242934	Bcl11a	ACAGCAGGCACAGACAGGCAGT#ACTGTCTGTGTTTTTTTGGCAGTTGTCTGCA#...(((((((((.(((((((((#))))))))).)..........)).)))))	6018	6049	0.84615	-21	0	5.89E-05	0.6	5.6	4.89	-8	18	31	9	3UTR
mmu-miR-214-3p	NM_001243050	Atp6v0a1	ACAGCAGGCACAGACAGGCAGT#CTGCCCGTCTCCACAGTGATCAGCTGTGTCTCTCTTGCCTGCTGG#.(((((((((.((((.((((((.#))))).))))........................))))))))).	2721	2766	0.92308	-28	1	0.0048566	0.4	0.3	0.06	-7	18	45	9	3UTR

mmu-miR-214-3p	NM_001243050	Atp6v0a1	ACAGCAGGCACAGACAGGCAGT#TGCCTGGGCCTTTGTGTCGCCCTGCAACCCCTTGTCCCTGCTGTG#((((((((((((((((((..#))))))....))))))...................)))))))).	2780	2825	0.84615	-25	1	0.031657	0.4	0.3	0.1	-4	20	45	8	3UTR
mmu-miR-214-3p	NM_001243050	Atp6v0a1	ACAGCAGGCACAGACAGGCAGT#GCTGAATGACTGTGCCTGA#....(((((((((.((..((((#))))..)).))))))))).	2885	2904	0.80769	-25	0	0.0353039	0.5	-0	-0.1	-10	15	19	9	3UTR
mmu-miR-214-3p	NM_001243051	Atp6v0a1	ACAGCAGGCACAGACAGGCAGT#CTGCCCGTCTCCACAGTGATCAGCTGTGTCTCTCTTGCCTGCTGG#.((((((((.((((.(((((.#))))).))))........................))))))))).	2742	2787	0.92308	-28	1	0.0048566	0.4	0.3	0.06	-7	18	45	9	3UTR
mmu-miR-214-3p	NM_001243051	Atp6v0a1	ACAGCAGGCACAGACAGGCAGT#TGCCTGGGCCTTTGTGTCGCCCTGCAACCCCTTGTCCCTGCTGTG#((((((((((((((((((..#))))))....))))))...................)))))))).	2801	2846	0.84615	-25	1	0.031657	0.4	0.3	0.1	-4	20	45	8	3UTR
mmu-miR-214-3p	NM_001243051	Atp6v0a1	ACAGCAGGCACAGACAGGCAGT#GCTGAATGACTGTGCCTGA#....(((((((((.((..((((#))))..)).))))))))).	2906	2925	0.80769	-25	0	0.0353039	0.5	-0	-0.1	-10	15	19	9	3UTR
mmu-miR-214-3p	NM_001243792	Esrrg	ACAGCAGGCACAGACAGGCAGT#GCTGCCTGTATACAAACTCTGTCTCAGAAGGAGCTGTG#((((((((((.(((((((((((#)))))))))......)).)))))........))))).	2993	3031	1	-23	1	0.0002872	0.5	1.1	1.24	-7	21	38	9	3UTR
mmu-miR-214-3p	NM_001243792	Esrrg	ACAGCAGGCACAGACAGGCAGT#ACTGTCGTCTTGTTCTCGTGGTGTTTGGATTCTTGTGGTGTGTGCTT#..((((.((((((((.((((((#))))))))))).........................)))).)))).	3541	3588	0.92308	-20	0	0.0236703	0.6	3.3	3.95	-7	18	47	10	3UTR
mmu-miR-214-3p	NM_001243792	Esrrg	ACAGCAGGCACAGACAGGCAGT#TTGTTTGTTTGTTTCCTTTCCTTCCTTCTGCTGC#.(((((((.((((((((((((.#))))))))))))))..........	2111	2145	0.92308	-20	1	0.0980333	0.6	0.1	0.88	-5	19	34	12	3UTR
mmu-miR-214-3p	NM_001244891	Celf1	ACAGCAGGCACAGACAGGCAGT#GCTGCTTTCTGTGGAAATGCTGA#.(((((..((((((.((((((((#)))))))))))))....))))).	2219	2242	1	-25	0	0.0013871	0.6	0.1	-0.2	-8	18	23	13	3UTR
mmu-miR-214-3p	NM_001244891	Celf1	ACAGCAGGCACAGACAGGCAGT#GCTGCCAGTGCTGTGCTCCCTAACCTTCTGTTCTGCC#...((((((((((((.((((((#)))))).)).))))))...............)))).	3841	3878	1	-24	0	0.1571897	0.4	0.8	0.95	-7	18	37	6	3UTR

mmu-miR-214-3p	NM_001244891	Celf1	ACAGCAGGCACAGACAGGCAGT#GCTGCCTTTCTGATTTCCAGCTGC#.((((.((..((((.(((((((#))))))).))))....)).)))).	7136	7160	0.92308	-24	0	0.0028507	0.6	3.2	3.8	-9	17	24	7	3UTR
mmu-miR-214-3p	NM_001244903	Celf1	ACAGCAGGCACAGACAGGCAGT#GCTGCTTTCTGTGGAAATGCTGA#.(((((..((((((.(((((((#))))))))))))....))))).	2166	2189	1	-25	0	0.0013871	0.6	0.1	-0.2	-8	18	23	13	3UTR
mmu-miR-214-3p	NM_001244903	Celf1	ACAGCAGGCACAGACAGGCAGT#GCTGCCAGTGCTGTGCTCCCTAACCTTCTGTTCTGCC#...(((((((((((.((((((#)))))).)).))))))...............)))).	3788	3825	1	-24	0	0.1571897	0.4	0.8	0.95	-7	18	37	6	3UTR
mmu-miR-214-3p	NM_001244903	Celf1	ACAGCAGGCACAGACAGGCAGT#GCTGCCTTTCTGATTTCCAGCTGC#.((((.((..((((.(((((((#))))))).))))....)).)))).	7083	7107	0.92308	-24	0	0.0028507	0.6	3.2	3.8	-9	17	24	7	3UTR
mmu-miR-214-3p	NM_001252220	Nbr1	ACAGCAGGCACAGACAGGCAGT#GCTCCTCGTCTGCCTGCTGC#.((((((((..(((((((.(((#)))))).)))))))))))).	1502	1522	1	-25	1	0.0028815	0.5	1.5	1.64	-8	18	20	12	CDS
mmu-miR-214-3p	NM_001252220	Nbr1	ACAGCAGGCACAGACAGGCAGT#GCTGCCAGTGAAAGGGAGCTGTACATTCCATCTGTGGACCTCCTGACTGCCCAGGACCTGCTGTC#((((((((((((((.((((((#))))))).).....................))))))..................)))))))).	2294	2359	0.84615	-24	1	1.61E-05	0.5	3.5	2.38	-4	21	65	8	CDS
mmu-miR-214-3p	NM_001252220	Nbr1	ACAGCAGGCACAGACAGGCAGT#TTGTCTGTTGCTGCCTCTGC#.(((.(((((..(((((((((.#)))))))))..)))))))).	3404	3424	1	-23	0	0.0011664	0.4	3.3	2.54	-14	17	20	9	CDS
mmu-miR-214-3p	NM_001252222	Nbr1	ACAGCAGGCACAGACAGGCAGT#GCTCCTCGTCTGCCTGCTGC#.((((((((..(((((((.(((#)))))).)))))))))))).	1325	1345	1	-25	1	0.0028815	0.5	1.5	1.64	-8	18	20	12	CDS
mmu-miR-214-3p	NM_001252222	Nbr1	ACAGCAGGCACAGACAGGCAGT#GCTGCCAGTGAAAGGGAGCTGTACATTCCATCTGTGGACCTCCTGACTGCCCAGGACCTGCTGTC#((((((((((((((.((((((#))))))).).....................))))))..................)))))))).	2117	2182	0.84615	-24	1	1.61E-05	0.5	3.5	2.38	-4	21	65	8	CDS
mmu-miR-214-3p	NM_001252222	Nbr1	ACAGCAGGCACAGACAGGCAGT#TTGTCTGTTGCTGCCTCTGC#.(((.(((((..(((((((((.#)))))))))..)))))))).	3116	3136	1	-23	0	0.0011664	0.4	3.3	2.54	-14	17	20	9	CDS
mmu-miR-214-3p	NM_001252223	Nbr1	ACAGCAGGCACAGACAGGCAGT#GCTCCTCGTCTGCCTGCTGC#.((((((((..(((((((.(((#)))))).)))))))))))).	1325	1345	1	-25	1	0.0028815	0.5	1.5	1.64	-8	18	20	12	CDS

mmu-miR-214-3p	NM_001252223	Nbr1	ACAGCAGGCACAGACAGGCAGT#GCTGCCAGTGAAAGGGAGCTGTACATTCCATCTGTGGACCTCCTGACTGCCCAGGACCTGCTGTC#((((((((((((((.((((((#)))))).)......................))))))..................)))))))).	2117	2182	0.84615	-24	1	1.61E-05	0.5	3.5	2.38	-4	21	65	8	CDS
mmu-miR-214-3p	NM_001252223	Nbr1	ACAGCAGGCACAGACAGGCAGT#ACTGCCTGCCGCTCAGAGGTGGTCTCTCTGCTGTC#((((((((((((.((((((((#))))))))...))....)))).....)))))))).	3376	3411	0.92308	-23	1	0.0061065	0.4	1	1.41	-5	21	35	8	3UTR
mmu-miR-214-3p	NM_001252444	Ppil2	ACAGCAGGCACAGACAGGCAGT#GCGCCATAGTCAGTGTCTGCTGTG#(((((((((((.(((.(((.((#)))))...))).))))))))))).	32	56	1	-27	1	0.0015739	0.4	0.7	0.42	-8	19	24	11	5UTR
mmu-miR-214-3p	NM_001252444	Ppil2	ACAGCAGGCACAGACAGGCAGT#GCTCTGTTTGCTCGAGCTTGCTGGTCCTGCTGTT#((((((((..(((.(((((...#.))))))))..)))))))	1923	1957	1	-26	1	0.0020201	0.4	3.9	3.01	-7	16	20	8	3UTR
mmu-miR-214-3p	NM_001252445	Ppil2	ACAGCAGGCACAGACAGGCAGT#GCGCCATAGTCAGTGTCTGCTGTG#(((((((((((.(((.(((.((#)))))...))).))))))))))).	32	56	0.92308	-27	1	0.0015739	0.4	0.2	-0.1	-8	19	24	11	5UTR
mmu-miR-214-3p	NM_001252501	Sorcs1	ACAGCAGGCACAGACAGGCAGT#TGCACTGTTTGCTGCCCTACTGTT#((((..((((((((((((((..#))).))))))).))))...)))).	1724	1748	0.84615	-22	0	0.0545115	0.5	1.8	3.11	-10	18	24	7	CDS
mmu-miR-214-3p	NM_001252508	D430019H16Rik	ACAGCAGGCACAGACAGGCAGT#GCAGGTTGGGGAAGGATCTGTGTCTGCTGTA#((((((((((((((........#.))))))))))))))).	1858	1889	1	-25	1	0.017128	0.5	0.1	0.03	-5	14	16	14	3UTR
mmu-miR-214-3p	NM_001252560	Mllt10	ACAGCAGGCACAGACAGGCAGT#CTGCTGTTCTGTGCTTCACTGGCTGCTGC#.((((((((((((..(((((.#)))))..))))))).......))))))).	3516	3545	1	-27	1	2.01E-05	0.5	1.4	1.58	-5	18	29	7	3UTR
mmu-miR-214-3p	NM_001252560	Mllt10	ACAGCAGGCACAGACAGGCAGT#GCGCCTGTGCGGAGGCCTGCTT#..(((((((...(((((((.((#)))))))))).)....))))))).	83	105	0.92308	-24	1	7.33E-05	0.3	2.3	0.96	-12	16	22	8	5UTR
mmu-miR-214-3p	NM_001252560	Mllt10	ACAGCAGGCACAGACAGGCAGT#GCTGCGTCATCTGTAACCCCTGCTGC#.((((((((.(((((.(.(((((#))))).)..)))))....))))))).	2119	2145	0.92308	-24	1	0.0222278	0.5	1.5	2.14	-6	18	26	7	CDS
mmu-miR-214-3p	NM_001252561	Mllt10	ACAGCAGGCACAGACAGGCAGT#CTGCTGTTCTGTGCTTCACTGGCTGCTGC#.((((((((((((..(((((.#)))))..))))))).......))))))).	3567	3596	1	-27	1	2.01E-05	0.5	1.4	1.58	-5	18	29	7	3UTR

mmu-miR-214-3p	NM_001252561	Mllt10	ACAGCAGGCACAGACAGGCAGT#GCGCCTGTGCGGAGGCCTGCTT#..(((((((...((((((((.((#)))))))).)....))))))).	83	105	0.92308	-24	1	7.33E-05	0.3	2.3	0.96	-12	16	22	8	5UTR
mmu-miR-214-3p	NM_001252561	Mllt10	ACAGCAGGCACAGACAGGCAGT#GCTGCGTCATCTGTAACCCCTGCTGC#.(((((((.(((((.(.(((((#))))).)..)))))....))))))).	2170	2196	0.92308	-24	1	0.0222278	0.5	1.5	2.14	-6	18	26	7	CDS
mmu-miR-214-3p	NM_001252654	Kcnab2	ACAGCAGGCACAGACAGGCAGT#ACTGCCTGCCTGTGAACCACACCTCAGAGCTAAGCTCCTGCTGG#.((((((((((((.((((((((#)))))))).)))))......................))))))).	2890	2934	0.92308	-32	1	2.06E-06	0.5	3.9	3.93	-5	20	44	8	3UTR
mmu-miR-214-3p	NM_001252655	Kcnab2	ACAGCAGGCACAGACAGGCAGT#ACTGCCTGCCTGTGAACCACACCTCAGAGCTAAGCTCCTGCTGG#.((((((((((((.((((((((#)))))))).)))))......................))))))).	2919	2963	0.89231	-32	1	2.06E-06	0.5	3.6	3.86	-5	20	44	8	3UTR
mmu-miR-214-3p	NM_001252656	Kcnab2	ACAGCAGGCACAGACAGGCAGT#ACTGCCTGCCTGTGAACCACACCTCAGAGCTAAGCTCCTGCTGG#.((((((((((((.((((((((#)))))))).)))))......................))))))).	2961	3005	0.89231	-32	1	2.06E-06	0.5	3.5	4.07	-5	20	44	8	3UTR
mmu-miR-214-3p	NM_001252658	Ldlr	ACAGCAGGCACAGACAGGCAGT#GCTGCCTGTCTGCCCCAGGTTCTGTTTTATATATTTATTGTCTGGGGACAGAAAAGGCTACTGGCTGTG#(((((((((((((((((((((#))))))))))))..........................)))))....................	2795	2864	0.98077	-27	1	0.0009377	0.6	0.6	-0.3	-4	22	69	12	3UTR
mmu-miR-214-3p	NM_001252658	Ldlr	ACAGCAGGCACAGACAGGCAGT#GCCTGTCCTGTGCGTTGCTT#..((((((((((((((((...#))))))).))))).))))).	4266	4286	0.92308	-28	1	0.0299477	0.5	-0	0.43	-5	17	20	7	3UTR
mmu-miR-214-3p	NM_001253704	Pym1	ACAGCAGGCACAGACAGGCAGT#GCTGCTGATCTGGGTGTCTTGTTGA#.(((((((((((((..((((((#))))))..)))..)))))).)))).	987	1012	0.92308	-23	1	0.003142	0.4	1.5	1	-5	19	25	6	3UTR
mmu-miR-214-3p	NM_001253768	Ssx2ip	ACAGCAGGCACAGACAGGCAGT#CTGCCTTTCTCTTTCCTTGTTCCTGCTGTG#((((((((.(((((.((((((.#)))))).)))........)).))))))))	3631	3661	0.92308	-25	1	0.0979845	0.6	0.4	-0.1	-6	19	30	8	3UTR
mmu-miR-214-3p	NM_001253768	Ssx2ip	ACAGCAGGCACAGACAGGCAGT#TTGTTTGTTTGGACTTCGCTGTC#((((((((..((((((((((((.#)))))))))))..))).))))).	2478	2501	1	-22	1	0.000716	0.6	1.4	1.28	-6	19	23	11	3UTR
mmu-miR-214-3p	NM_001253769	Ssx2ip	ACAGCAGGCACAGACAGGCAGT#CTGCCTTTCTCTTTCCTTGTTCCTGCTGTG#((((((((.(((((.((((((.#)))))).)))........)).))))))))	3527	3557	0.92308	-25	1	0.0979845	0.6	0.4	-0.1	-6	19	30	8	3UTR

mmu-miR-214-3p	NM_001253769	Ssx2ip	ACAGCAGGCACAGACAGGCAGT#TTGTTTGTTTGGACTTCGCTGTC#((((((((..((((((((((((.#)))))))))))..))).))))).	2374	2397	1	-22	1	0.000716	0.6	1.4	1.28	-6	19	23	11	3UTR
mmu-miR-214-3p	NM_001253770	Ssx2ip	ACAGCAGGCACAGACAGGCAGT#CTGCCTTTCTCTTTCCTTGTTCCTGCTGTG#((((((((.(((((.((((((.#)))))).)))........)).))))))))	3376	3406	0.92308	-25	1	0.0979845	0.6	0.4	-0.1	-6	19	30	8	3UTR
mmu-miR-214-3p	NM_001253770	Ssx2ip	ACAGCAGGCACAGACAGGCAGT#TTGTTTGTTTGGACTTCGCTGTC#((((((((..((((((((((((.#)))))))))))..))).))))).	2223	2246	1	-22	1	0.000716	0.6	1.4	1.28	-6	19	23	11	3UTR
mmu-miR-214-3p	NM_001253784	Def8	ACAGCAGGCACAGACAGGCAGT#CTGCCCCTTTGCTCCTTGTCTGTGAATTGCTGTT#(((((((.(((((((((((((.#)))))).........)))))))).))))))).	2725	2759	0.84615	-27	1	0.0068241	0.5	-0	-0.4	-6	20	34	8	3UTR
mmu-miR-214-3p	NM_001253784	Def8	ACAGCAGGCACAGACAGGCAGT#GGTGCCTGCTGGCCTTGCC#...((((((.(((.((((((.(#).))))))))))))).)).	1581	1600	0.84615	-24	0	4.00E-06	0.4	-0	0.04	-9	16	19	13	3UTR
mmu-miR-214-3p	NM_001253805	Ywhaz	ACAGCAGGCACAGACAGGCAGT#ACTGCTTCCATGTCTAAGCAAAGAAAACTGCCTACATATTGGTGTGTGCTGG#.(((((.((((....(((((((#.))))))).........)))).))))).	1451	1503	0.92308	-25	0	0.0001701	0.5	5	3.51	-12	16	27	7	3UTR
mmu-miR-214-3p	NM_001253805	Ywhaz	ACAGCAGGCACAGACAGGCAGT#TTGCTTGGTGTGCCCGGCTC#..(((.((((((..((((((((.#))))))).))))))..))).	1841	1861	0.92308	-23	0	3.22E-05	0.5	3.3	3.55	-9	16	20	7	3UTR
mmu-miR-214-3p	NM_001253817	Tmem184b	ACAGCAGGCACAGACAGGCAGT#GCTCTGTGGACCCTCCCCCTCTCCCAACCTAGGCCTGTCTGGCATGCTGC#.(((((.((.(((((((((...#.)))))))))))).))))).	1784	1834	1	-30	0	5.95E-05	0.3	0.2	-0.1	-7	16	19	11	3UTR
mmu-miR-214-3p	NM_001253819	Tmem184b	ACAGCAGGCACAGACAGGCAGT#GCTCTGTGGACCCTCCCCCTCTCCCAACCTAGGCCTGTCTGGCATGCTGC#.(((((.((.(((((((((...#.)))))))))))).))))).	1826	1876	0.92308	-30	0	5.95E-05	0.3	0.4	0.29	-7	16	19	11	3UTR
mmu-miR-214-3p	NM_001253860	Scn5a	ACAGCAGGCACAGACAGGCAGT#GCTGGGCCTGTCTCGCATGGGCAACCTGTCTGTGCTGCGTTCCTTCCGTCTGCTGC#.(((((((((((((((((..((#.))..)))))))))))))............	2438	2494	1	-29	1	0.0048814	0.4	2.3	2.02	-4	19	37	11	CDS
mmu-miR-214-3p	NM_001255997	Cacna1c	ACAGCAGGCACAGACAGGCAGT#CCTGTCATGTGCCATGCCTGTT#(((((((((((((((((((....#)))))).))))))).))).))).	12964	####	0.96154	-26	1	0.0031596	0.5	2.4	3.11	-4	18	22	6	3UTR

mmu-miR-214-3p	NM_001255997	Cacna1c	ACAGCAGGCACAGACAGGCAGT#CCTGCTCTGTGTCTGCC#...((((((((((((((....#)))).))))))))))).	7511	7528	0.92308	-26	0	0.0055044	0.6	2.3	1.24	-7	15	17	11	3UTR
mmu-miR-214-3p	NM_001255997	Cacna1c	ACAGCAGGCACAGACAGGCAGT#CTGCCTGCTGCGCCTTCTC#..((.((((.(((.((((((.#)))))))))).)))).)).	13138	####	0.84615	-26	0	1.86E-05	0.4	-0	0.92	-11	16	19	10	3UTR
mmu-miR-214-3p	NM_001255998	Cacna1c	ACAGCAGGCACAGACAGGCAGT#CCTGTCATGTGCCATGCCTGTT#(((((((((((((((((....#)))))).)))))).))).))).	13039	####	0.96154	-26	1	0.0031596	0.5	2.4	3.11	-4	18	22	6	3UTR
mmu-miR-214-3p	NM_001255998	Cacna1c	ACAGCAGGCACAGACAGGCAGT#CCTGCTCTGTGTCTGCC#...((((((((((((((....#)))).))))))))))).	7586	7603	0.92308	-26	0	0.0055044	0.6	1.2	1.43	-7	15	17	11	3UTR
mmu-miR-214-3p	NM_001255998	Cacna1c	ACAGCAGGCACAGACAGGCAGT#CTGCCTGCTGCGCCTTCTC#..((.((((.(((.((((((.#)))))))))).)))).)).	13213	####	0.84615	-26	0	1.86E-05	0.4	-0	0.92	-11	16	19	10	3UTR
mmu-miR-214-3p	NM_001255999	Cacna1c	ACAGCAGGCACAGACAGGCAGT#CCTGTCATGTGCCATGCCTGTT#(((((((((((((((((....#)))))).)))))).))).))).	12964	####	0.96154	-26	1	0.0031596	0.5	2.4	3.11	-4	18	22	6	3UTR
mmu-miR-214-3p	NM_001255999	Cacna1c	ACAGCAGGCACAGACAGGCAGT#CCTGCTCTGTGTCTGCC#...((((((((((((((....#)))).))))))))))).	7511	7528	0.92308	-26	0	0.0055044	0.6	2.3	1.24	-7	15	17	11	3UTR
mmu-miR-214-3p	NM_001255999	Cacna1c	ACAGCAGGCACAGACAGGCAGT#CTGCCTGCTGCGCCTTCTC#..((.((((.(((.((((((.#)))))))))).)))).)).	13138	####	0.84615	-26	0	1.86E-05	0.4	-0	0.92	-11	16	19	10	3UTR
mmu-miR-214-3p	NM_001256000	Cacna1c	ACAGCAGGCACAGACAGGCAGT#CCTGTCATGTGCCATGCCTGTT#(((((((((((((((((....#)))))).)))))).))).))).	13258	####	0.96154	-26	1	0.0031596	0.5	1.7	1.67	-4	18	22	6	3UTR
mmu-miR-214-3p	NM_001256000	Cacna1c	ACAGCAGGCACAGACAGGCAGT#CCTGCTCTGTGTCTGCC#...((((((((((((((....#)))).))))))))))).	7805	7822	1	-26	0	0.0055044	0.6	3.7	3.48	-7	15	17	11	3UTR
mmu-miR-214-3p	NM_001256000	Cacna1c	ACAGCAGGCACAGACAGGCAGT#CTGCCTGCTGCGCCTTCTC#..((.((((.(((.((((((.#)))))))))).)))).)).	13432	####	0.92308	-26	0	1.86E-05	0.4	0.7	0.84	-11	16	19	10	3UTR
mmu-miR-214-3p	NM_001256002	Cacna1c	ACAGCAGGCACAGACAGGCAGT#CCTGTCATGTGCCATGCCTGTT#(((((((((((((((((....#)))))).)))))).))).))).	13465	####	0.96154	-26	1	0.0031596	0.5	1.7	1.67	-4	18	22	6	3UTR

mmu-miR-214-3p	NM_001256002	Cacna1c	ACAGCAGGCACAGACAGGCAGT#CCTGCTCTGTGTCTGCC#...((((((((((((((....#)))).)))))))))).	8012	8029	1	-26	0	0.0055044	0.6	2.7	3.26	-7	15	17	11	3UTR
mmu-miR-214-3p	NM_001256002	Cacna1c	ACAGCAGGCACAGACAGGCAGT#CTGCCTGCTGCGCCTTCTC#..((.((((.(((.(((((((.#)))))))))).)))).)).	13639	####	0.92308	-26	0	1.86E-05	0.4	0.7	0.84	-11	16	19	10	3UTR
mmu-miR-214-3p	NM_001271447	Enox2	ACAGCAGGCACAGACAGGCAGT#GCTGCAATGACGCTGCCTGTGTCTGATCCAGCTGC#.((((.((..(((((((((((#.)))))))..)))))	378	413	1	-27	0	0.0031637	0.5	1.3	1.21	-8	18	25	7	5UTR
mmu-miR-214-3p	NM_001271447	Enox2	ACAGCAGGCACAGACAGGCAGT#ACTGTCTGTTGGTCCCATTTTTTCCTGCTGTT#((((((((.((.((((((((((#)))))))))).)).........))	3029	3061	0.92308	-25	1	0.0763281	0.6	4.1	3.54	-8	20	32	10	3UTR
mmu-miR-214-3p	NM_001271448	Enox2	ACAGCAGGCACAGACAGGCAGT#GCTGCAATGACGCTGCCTGTGTCTGATCCAGCTGC#.((((.((..(((((((((((#.)))))))..)))))	223	258	1	-27	0	0.0031636	0.5	0.3	0.48	-8	18	25	7	5UTR
mmu-miR-214-3p	NM_001271448	Enox2	ACAGCAGGCACAGACAGGCAGT#ACTGTCTGTTGGTCCCATTTTTTCCTGCTGTT#((((((((.((.((((((((((#)))))))))).)).........))	2874	2906	0.92308	-25	1	0.0763281	0.6	2.6	3.69	-8	20	32	10	3UTR
mmu-miR-214-3p	NM_001271449	Enox2	ACAGCAGGCACAGACAGGCAGT#GCTGCAATGACGCTGCCTGTGTCTGATCCAGCTGC#.((((.((..(((((((((((#.)))))))..)))))	367	402	1	-27	0	0.0031637	0.5	0.7	0.96	-8	18	25	7	5UTR
mmu-miR-214-3p	NM_001271449	Enox2	ACAGCAGGCACAGACAGGCAGT#ACTGTCTGTTGGTCCCATTTTTTCCTGCTGTT#((((((((.((.((((((((((#)))))))))).)).........))	3018	3050	0.92308	-25	1	0.0763281	0.6	3.8	3.66	-8	20	32	10	3UTR
mmu-miR-214-3p	NM_001271450	Enox2	ACAGCAGGCACAGACAGGCAGT#GCTGCAATGACGCTGCCTGTGTCTGATCCAGCTGC#.((((.((..(((((((((((#.)))))))..)))))	367	402	1	-27	0	0.0031637	0.5	0.7	0.96	-8	18	25	7	5UTR
mmu-miR-214-3p	NM_001271450	Enox2	ACAGCAGGCACAGACAGGCAGT#ACTGTCTGTTGGTCCCATTTTTTCCTGCTGTT#((((((((.((.((((((((((#)))))))))).)).........))	2985	3017	0.92308	-25	1	0.0763281	0.6	3.8	3.66	-8	20	32	10	3UTR
mmu-miR-214-3p	NM_001271451	Enox2	ACAGCAGGCACAGACAGGCAGT#GCTGCAATGACGCTGCCTGTGTCTGATCCAGCTGC#.((((.((..(((((((((((#.)))))))..)))))	367	402	1	-27	0	0.0031637	0.5	0.7	0.96	-8	18	25	7	5UTR
mmu-miR-214-3p	NM_001271451	Enox2	ACAGCAGGCACAGACAGGCAGT#ACTGTCTGTTGGTCCCATTTTTTCCTGCTGTT#((((((((.((.((((((((((#)))))))))).)).........))	2970	3002	0.92308	-25	1	0.0763281	0.6	3.8	3.66	-8	20	32	10	3UTR

mmu-miR-214-3p	NM_001271584	Dnajc5	ACAGCAGGCACAGACAGGCAGT#GCTGTTTGTTGTTTGTGGCCTCCTCACCTGCTGC#.((((((((((((((((((((#)))))...)))))))))........))))))).	600	634	1	-25	1	0.0004061	0.5	3	4.32	-4	21	34	9	CDS
mmu-miR-214-3p	NM_001271858	Add2	ACAGCAGGCACAGACAGGCAGT#CTGCCGGTCTCCCATAATGCCCTGCTGG#.((((((((.((((.(((((.#))))).)))).......)))).))))).	1128	1156	0.84615	-25	1	0.000647	0.4	3.5	3.46	-7	18	28	5	CDS
mmu-miR-214-3p	NM_001271858	Add2	ACAGCAGGCACAGACAGGCAGT#CTGTCTGTATTGTGCTTCTCACAGGCTGA#.(((((((((((((((((((.#)))))))).))))))))).......)))).	3293	3322	1	-25	1	0.000123	0.6	0.4	0.26	-4	20	29	8	3UTR
mmu-miR-214-3p	NM_001271858	Add2	ACAGCAGGCACAGACAGGCAGT#CTGCTTGTGGGTTTCTCTGTGTGTGCCGTT#((.(((.((((((((((((((.#))))))))........))))))).))).)	8124	8154	1	-25	0	0.0626007	0.5	1	1.09	-7	19	30	7	3UTR
mmu-miR-214-3p	NM_001271859	Add2	ACAGCAGGCACAGACAGGCAGT#CTGCCGGTCTCCCATAATGCCCTGCTGG#.((((((((.((((.(((((.#))))).)))).......)))).))))).	929	957	0.84615	-25	1	0.000647	0.4	3.5	3.46	-7	18	28	5	CDS
mmu-miR-214-3p	NM_001271859	Add2	ACAGCAGGCACAGACAGGCAGT#CTGTCTGTATTGTGCTTCTCACAGGCTGA#.(((((((((((((((((((.#)))))))).))))))))).......)))).	3094	3123	1	-25	1	0.000123	0.6	0.4	0.26	-4	20	29	8	3UTR
mmu-miR-214-3p	NM_001271859	Add2	ACAGCAGGCACAGACAGGCAGT#CTGCTTGTGGGTTTCTCTGTGTGTGCCGTT#((.(((.((((((((((((((.#))))))))........))))))).))).)	7925	7955	1	-25	0	0.0565355	0.5	1	1.09	-7	19	30	7	3UTR
mmu-miR-214-3p	NM_001271860	Add2	ACAGCAGGCACAGACAGGCAGT#CTGCCGGTCTCCCATAATGCCCTGCTGG#.((((((((.((((.(((((.#))))).)))).......)))).))))).	1131	1159	0.84615	-25	1	0.000647	0.4	3.5	3.46	-7	18	28	5	CDS
mmu-miR-214-3p	NM_001271860	Add2	ACAGCAGGCACAGACAGGCAGT#GCCTCTCTCAGTGTCCTGCTGA#.(((((((((((((.((((...#)))).)))..))).))))))).	2273	2295	1	-26	1	0.0002545	0.4	-0	0.08	-5	17	22	7	3UTR
mmu-miR-214-3p	NM_001271860	Add2	ACAGCAGGCACAGACAGGCAGT#TGCAGGTCTGGCCTCTGG#.(((.((((.(((((..(((..#)))..)))))))))))).	3191	3209	0.92308	-24	0	5.55E-05	0.5	0.9	0.39	-9	15	18	12	3UTR
mmu-miR-214-3p	NM_001271861	Add2	ACAGCAGGCACAGACAGGCAGT#CTGCCGGTCTCCCATAATGCCCTGCTGG#.((((((((.((((.(((((.#))))).)))).......)))).))))).	932	960	0.84615	-25	1	0.000647	0.4	3.5	3.46	-7	18	28	5	CDS
mmu-miR-214-3p	NM_001271861	Add2	ACAGCAGGCACAGACAGGCAGT#GCCTCTCTCAGTGTCCTGCTGA#.(((((((((((((.((((...#)))).)))..))).))))))).	2074	2096	1	-26	1	0.0002545	0.4	-0	0.08	-5	17	22	7	3UTR

mmu-miR-214-3p	NM_001271861	Add2	ACAGCAGGCACAGACAGGCAGT#TGCAGGTCTGGCCTCTGG#.(((.((((.(((((..(((..#)))..)))))))))))).	2992	3010	0.92308	-24	0	5.55E-05	0.5	0.9	0.39	-9	15	18	12	3UTR
mmu-miR-214-3p	NM_001271879	Ano5	ACAGCAGGCACAGACAGGCAGT#ACAGCCTGTCAGTGTTCCTGATAGCTGA#.((((((((((.(((((((.((#)).))))))).)))..)))....)))).	2364	2392	0.92308	-23	1	0.003515	0.5	1.4	1.28	-7	19	28	7	CDS
mmu-miR-214-3p	NM_001271879	Ano5	ACAGCAGGCACAGACAGGCAGT#GCTGCTTTACTGCTGTCTGTC#...((((((((((..((((((((#)))))))..))).))))))).	6994	7015	0.84615	-22	0	0.0005445	0.6	-0	-0.2	-8	17	21	7	3UTR
mmu-miR-214-3p	NM_001272028	Arvcf	ACAGCAGGCACAGACAGGCAGT#ACTGCCGGCAGCAGTGGGGGGAACTGCTGTTCAGTGCCTGCTGTT#((((((((((((.(((..((((((#.))))))..)).))))))))))).	3691	3736	0.92308	-32	1	4.43E-05	0.5	-0	0.57	-8	19	24	11	3UTR
mmu-miR-214-3p	NM_001272028	Arvcf	ACAGCAGGCACAGACAGGCAGT#ACTGCCTGTCTTCTGCCTTGGCTC#..((((((((.((((((((((((#))))))))))))..)))))..))).	3853	3877	1	-29	1	0.0003356	0.5	-0	0.53	-8	19	24	11	3UTR
mmu-miR-214-3p	NM_001272029	Arvcf	ACAGCAGGCACAGACAGGCAGT#ACTGCCGGCAGCAGTGGGGGGAACTGCTGTTCAGTGCCTGCTGTT#((((((((((((.(((..((((((#.))))))..)).))))))))))).	3824	3869	0.92308	-32	1	4.43E-05	0.5	-0	0.57	-8	19	24	11	3UTR
mmu-miR-214-3p	NM_001272029	Arvcf	ACAGCAGGCACAGACAGGCAGT#ACTGCCTGTCTTCTGCCTTGGCTC#..((((((((.((((((((((((#))))))))))))..)))))..))).	3986	4010	1	-29	1	0.0003356	0.5	-0	0.53	-8	19	24	11	3UTR
mmu-miR-214-3p	NM_001272030	Arvcf	ACAGCAGGCACAGACAGGCAGT#ACTGCCGGCAGCAGTGGGGGGAACTGCTGTTCAGTGCCTGCTGTT#((((((((((((.(((..((((((#.))))))..)).))))))))))).	3673	3718	0.92308	-32	1	4.43E-05	0.5	-0	0.57	-8	19	24	11	3UTR
mmu-miR-214-3p	NM_001272030	Arvcf	ACAGCAGGCACAGACAGGCAGT#ACTGCCTGTCTTCTGCCTTGGCTC#..((((((((.((((((((((((#))))))))))))..)))))..))).	3835	3859	1	-29	1	0.0003356	0.5	-0	0.53	-8	19	24	11	3UTR
mmu-miR-214-3p	NM_001272031	Arvcf	ACAGCAGGCACAGACAGGCAGT#ACTGCCGGCAGCAGTGGGGGGAACTGCTGTTCAGTGCCTGCTGTT#((((((((((((.(((..((((((#.))))))..)).))))))))))).	3434	3479	0.92308	-32	1	4.43E-05	0.5	-0	0.57	-8	19	24	11	3UTR
mmu-miR-214-3p	NM_001272031	Arvcf	ACAGCAGGCACAGACAGGCAGT#ACTGCCTGTCTTCTGCCTTGGCTC#..((((((((.((((((((((((#))))))))))))..)))))..))).	3596	3620	1	-29	1	0.0003356	0.5	-0	0.53	-8	19	24	11	3UTR

mmu-miR-214-3p	NM_001272032	Arvcf	ACAGCAGGCACAGACAGGCAGT#ACTGCCGGCAGCAGTGGGGGGAACTGCTGTTCAGTGCCTGCTGTT#(((((((((((.((..((((((#.))))))..)).))))))))))).	3416	3461	0.92308	-32	1	4.43E-05	0.5	-0	0.57	-8	19	24	11	3UTR
mmu-miR-214-3p	NM_001272032	Arvcf	ACAGCAGGCACAGACAGGCAGT#ACTGCCTGTCTTCTGCCTTGGCTC#..((((((((.(((((((((((#)))))))))))..)))))..))).	3578	3602	1	-29	1	0.0003356	0.5	-0	0.53	-8	19	24	11	3UTR
mmu-miR-214-3p	NM_001276263	Cxadr	ACAGCAGGCACAGACAGGCAGT#GCGCGCCTACTGTGCTTCGTGCTC#..(((((((((((..((((.((#))..)))).)))))))...))))).	164	188	0.84615	-20	1	0.000928	0.4	0.5	0.93	-7	17	24	7	CDS
mmu-miR-214-3p	NM_001276263	Cxadr	ACAGCAGGCACAGACAGGCAGT#CTTCCTGCTGTGTTTTGAGGGTTGTG#((((((((((((.((((.((.#)).))))))))))))).....))))).	809	835	0.84615	-20	1	0.0148666	0.5	0	0.12	-5	19	26	12	3UTR
mmu-miR-214-3p	NM_001276431	Wfdc8	ACAGCAGGCACAGACAGGCAGT#GCTCTGCTGCTGCTGCTGCTGTT#(((((((((((((.(((((...#)).)))))).)))..))))))).	559	582	0.92308	-23	1	0.0007537	0.4	1.1	-0	-5	18	23	7	CDS
mmu-miR-214-3p	NM_001276455	Slc19a2	ACAGCAGGCACAGACAGGCAGT#GCATGTGTGTGTGTGTCTGCA#...(((((((((.(((.(((((#)).))).))).))))))))).	2040	2061	1	-26	0	0.0009372	0.5	0.6	0.35	-8	17	21	9	3UTR
mmu-miR-214-3p	NM_001276455	Slc19a2	ACAGCAGGCACAGACAGGCAGT#ACTACCTCCGGTACAAGCCTGTCATCCTGCTGC#.(((((((....(((((((...#.)))))))..))))))).	484	517	0.92308	-26	1	0.0010439	0.4	3.8	3.22	-12	14	18	7	CDS
mmu-miR-214-3p	NM_001276455	Slc19a2	ACAGCAGGCACAGACAGGCAGT#CTGTGTTTCTGTGGCTTTTGCTGTG#(((((((.((((((.(.((((.#)))).).)))))).))...))))).	794	819	0.84615	-24	1	0.0002105	0.5	2.6	2.57	-7	18	25	6	CDS
mmu-miR-214-3p	NM_001276684	Arc	ACAGCAGGCACAGACAGGCAGT#GCCTGTCTTTGCCGCTGC#.((((.((((.((((((((...#)))))))).)))))))).	474	492	0.84615	-29	0	0.0002203	0.4	0	0.13	-9	16	18	8	CDS
mmu-miR-214-3p	NM_001276684	Arc	ACAGCAGGCACAGACAGGCAGT#CTGCCTGGTGATTGCCCAGCTGA#.((((.((((....(((((((.#)))))))).....)))).))))).	2636	2659	0.92308	-24	0	0.0002145	0.4	1.7	2.03	-14	15	23	7	3UTR
mmu-miR-214-3p	NM_001276719	Ackr2	ACAGCAGGCACAGACAGGCAGT#GCTTGTGTGTGGAAAGCCTTTGGAAAGCTGCTGTG#(((((((.((((.((((((...#)))))).))))................))))))).	2343	2378	0.92308	-23	1	0.0037857	0.5	0	0.17	-8	17	35	7	3UTR
mmu-miR-214-3p	NM_001276719	Ackr2	ACAGCAGGCACAGACAGGCAGT#CTCCTGTCGTGCTCCCTGCA#...((((((((.((((((.((.#))))))))))))...))))).	2234	2254	0.84615	-22	0	1.06E-05	0.4	-0	0.11	-10	16	20	12	3UTR

mmu-miR-214-3p	NM_001276719	Ackr2	ACAGCAGGCACAGACAGGCAGT#CTGAAGGCATTTCTGTCTGTGATGTTGA#.(((((..((((((((((....#.)))))))))).))))).	1464	1492	0.92308	-21	0	0.0173136	0.4	0.5	0.87	-8	15	18	10	CDS
mmu-miR-214-3p	NM_001277231	Crkl	ACAGCAGGCACAGACAGGCAGT#ATTGCTGTGCTGTCCCTGCTGC#.(((((((.((((...((((((#))))))...)))).))))))).	938	960	1	-26	1	0.0047068	0.4	1.1	2.38	-7	17	22	7	3UTR
mmu-miR-214-3p	NM_001278534	Rtl5	ACAGCAGGCACAGACAGGCAGT#TTGCCTGTCTTCACGGTGCTTCTTAGTCTGAATGCTTGTG#((((((((((((((((((((.#)))))))))))).....)))))............)))).)).	3926	3966	0.92308	-24	1	0.0007276	0.5	-0	0.2	-4	21	40	10	3UTR
mmu-miR-214-3p	NM_001278605	Umod	ACAGCAGGCACAGACAGGCAGT#ACTGTCTGTTGTGCTGGGCA#...((.((((((.((((((((((#)))))))))))))))..)).	2369	2389	0.84615	-24	0	2.09E-05	0.5	-0	0.5	-10	17	20	15	3UTR
mmu-miR-214-3p	NM_001278605	Umod	ACAGCAGGCACAGACAGGCAGT#GCTCCTGAGCATCTGGCTGCTGTT#((((((.((.((((((((.(((#))))))))....)))))))))))).	2011	2035	1	-21	0	0.0008576	0.5	1.5	1.13	-7	19	24	12	CDS
mmu-miR-214-3p	NM_001281803	Def8	ACAGCAGGCACAGACAGGCAGT#CTGCCCCTTTGCTCCTTGTCTGTGAATTGCTGTT#(((((((.(((((((((((((.#)))))...........))))))))..))))))).	2729	2763	0.84615	-27	1	0.0068241	0.5	-0	-0.4	-6	20	34	8	3UTR
mmu-miR-214-3p	NM_001281803	Def8	ACAGCAGGCACAGACAGGCAGT#GGTGCCTGCTGGCCTTGCC#...((((((.(((.((((((.(#).)))))))))))))).)).	1585	1604	0.84615	-24	0	4.00E-06	0.4	-0	0.04	-9	16	19	13	3UTR
mmu-miR-214-3p	NM_001281880	Sema4d	ACAGCAGGCACAGACAGGCAGT#GCTCCAGTCTGTGCTGCTGG#.(((((.((((((((.((.(((#))))).))))))))))))).	409	429	0.96154	-27	0	2.56E-05	0.4	-0	-0.2	-6	18	20	13	5UTR
mmu-miR-214-3p	NM_001281880	Sema4d	ACAGCAGGCACAGACAGGCAGT#ACTGTGTGTTAGCACGGGACCCCTACTGTGCCTGGAGC#.(..((((((((((((.(((((#))))).))).................)))))))))..).	2059	2097	0.92308	-24	0	0.0003036	0.4	2	1.55	-8	18	38	9	CDS
mmu-miR-214-3p	NM_001282071	Sftpb	ACAGCAGGCACAGACAGGCAGT#GCTGCTTGTGCCCCGGTGTCGCCAAGTGCTTGATGTC#(((.(((((((..(((((((((#)))))))))).................))))))).))).	329	366	0.84615	-22	0	0.0079746	0.5	1	0.27	-12	19	37	9	CDS
mmu-miR-214-3p	NM_001284270	Tmem201	ACAGCAGGCACAGACAGGCAGT#CTGCCTGCTGGCCATGCTGC#.((((((((.((((.(((((((.#))))))))))))).))))).	1041	1061	0.84615	-31	1	6.31E-08	0.3	0	-0.3	-6	18	20	13	CDS

mmu-miR-214-3p	NM_001284270	Tmem201	ACAGCAGGCACAGACAGGCAGT#GCTGCCTGTGCCCGTCTGCTGC#.((((((((....(((((((((#)))))))))....)))))))).	2380	2402	0.92308	-30	1	5.03E-06	0.3	0.5	1.2	-12	17	22	9	3UTR
mmu-miR-214-3p	NM_001284270	Tmem201	ACAGCAGGCACAGACAGGCAGT#ACTGCCTGCTGAGGGCACTGCAGGCTTGCTGG#.((((((((.(((.((((((((#)))))))))))))))))).........	2891	2923	1	-28	1	9.71E-07	0.4	1.5	1.57	-6	19	32	11	3UTR
mmu-miR-214-3p	NM_001284345	Atp8a1	ACAGCAGGCACAGACAGGCAGT#ATTGCCATGTCTCTTGTGTGTTCTGTG#(((((.(.(((((((((((((((#)))))).)))))...)))).).)))).	1136	1163	0.84615	-23	0	0.0133841	0.5	0.6	0.6	-10	20	27	6	CDS
mmu-miR-214-3p	NM_001284345	Atp8a1	ACAGCAGGCACAGACAGGCAGT#GCTGCGTGGATAGTGTCTAATCTGTG#((((.((((((((..((.(((((#))))).))..).)))))))...)))).	7944	7970	0.84615	-22	0	0.0006857	0.5	1	1.15	-10	18	26	6	3UTR
mmu-miR-214-3p	NM_001284345	Atp8a1	ACAGCAGGCACAGACAGGCAGT#ACTGCTTTTCTGTCGTTTGTC#...((((((((((((.((((((((#))))))).))))).)))))).	4413	4434	0.92308	-22	0	0.1259908	0.6	-0	1.19	-7	18	21	7	3UTR
mmu-miR-214-3p	NM_001284398	Septin4	ACAGCAGGCACAGACAGGCAGT#GCCTTATATCCCTGCTGA#.(((((((.......((((...#))))......))))))).	1279	1297	0.92308	-19	1	0.0152221	0.5	2.1	1.7	-15	11	18	7	3UTR
mmu-miR-214-3p	NM_001285412	Calu	ACAGCAGGCACAGACAGGCAGT#CTGCCATTGTCTGGGCAACGATAGAGTCCTGTGCTGTA#((((((.((.(((((((((((.#)))))..))))))).))..............)))))).	1429	1467	0.92308	-23	0	0.0017366	0.5	0.1	0.31	-7	19	38	6	3UTR
mmu-miR-214-3p	NM_001285412	Calu	ACAGCAGGCACAGACAGGCAGT#TGTGTGTCAGGATCGATGCTTAGAGGAGAATGCTTTCTCTTGCCTGTTGA#.((((((((((.(((.(((((..#.))))))))..)))))))))).	1326	1376	1	-22	1	0.0005397	0.5	0.2	0.54	-7	17	21	9	3UTR
mmu-miR-214-3p	NM_001285452	Tmem33	ACAGCAGGCACAGACAGGCAGT#CTGCACCATAACAGAGAAGTCTGTCTGCGTCTTATGACCTGTGAAGCTGTT#((((((((((.((((((((((...#.)))))))))).))))............))))).	1669	1720	0.92308	-25	1	0.0038065	0.5	0.3	0.54	-5	18	34	9	3UTR
mmu-miR-214-3p	NM_001285452	Tmem33	ACAGCAGGCACAGACAGGCAGT#GCTGCCACTACCTGCTGTA#((((((((...((...(((((((#)))))).)).)))))))).	698	717	1	-23	1	0.018122	0.5	3.4	4.02	-11	16	19	8	CDS
mmu-miR-214-3p	NM_001285452	Tmem33	ACAGCAGGCACAGACAGGCAGT#GCTGCTTTCTAGGGCAGGTGTGTTTAGCCTGCA#...(((((((((.(((((((((#))))))))..........)).)	1641	1674	0.92308	-22	0	8.39E-05	0.5	0.9	0.56	-8	18	33	7	3UTR

mmu-miR-214-3p	NM_001285913	Cdh8	ACAGCAGGCACAGACAGGCAGT#GTCTGTCTGGAACCACAACACTCACAGTGACCCTGACTGA#.(((((((..(((((((((...#))))))))))......................)))).))).	1353	1393	0.92308	-22	1	0.0019579	0.6	2.5	3.57	-6	16	40	9	CDS
mmu-miR-214-3p	NM_001285913	Cdh8	ACAGCAGGCACAGACAGGCAGT#AGTGCCTGTTGCTATTAAAGTGCTGG#.(((((((((...(((((((.(#).)))))))))))).......))))).	2026	2052	0.84615	-20	1	0.0063455	0.5	3.5	3.7	-11	17	26	11	CDS
mmu-miR-214-3p	NM_001285914	Cdh8	ACAGCAGGCACAGACAGGCAGT#GTCTGTCTGGAACCACAACACTCACAGTGACCCTGACTGA#.(((((((..(((((((((...#))))))))))......................)))).))).	1353	1393	1	-22	1	0.0019579	0.6	2.4	2.97	-6	16	40	9	CDS
mmu-miR-214-3p	NM_001285914	Cdh8	ACAGCAGGCACAGACAGGCAGT#AGTGCCTGTTGCTATTAAAGTGCTGG#.(((((((((...(((((((.(#).)))))))))))).......))))).	2026	2052	0.84615	-20	1	0.0063455	0.5	4.3	3.57	-11	17	26	11	CDS
mmu-miR-214-3p	NM_001285920	Mfn2	ACAGCAGGCACAGACAGGCAGT#ACTGGACAGCTCGGCTCCAAGGAGTTATGCATGTGTCTGTGTCTGCTC#..((((((((((((((.(((..#.)))...)))))))))))))).	2922	2970	1	-28	1	0.0003483	0.5	2.5	3.04	-5	17	22	14	3UTR
mmu-miR-214-3p	NM_001285923	Mfn2	ACAGCAGGCACAGACAGGCAGT#ACTGGACAGCTCGGCTCCAAGGAGTTATGCATGTGTCTGTGTCTGCTC#..((((((((((((((.(((..#.)))...)))))))))))))).	2836	2884	1	-28	1	0.0003483	0.5	3.2	2.98	-5	17	22	14	3UTR
mmu-miR-214-3p	NM_001285923	Mfn2	ACAGCAGGCACAGACAGGCAGT#CTGCCCATGTCTTGTGCTCACTCGCCCCTCAGTCTACCCTTTTTGCTGG#.((((((((((((((((((((.#)))))..))))).))))).........................))))	3966	4015	0.84615	-25	1	0.0001683	0.4	4.2	2.8	-4	20	49	5	3UTR
mmu-miR-214-3p	NM_001285991	Tbc1d5	ACAGCAGGCACAGACAGGCAGT#ACTGTCTCCACTCTATGCAATGTATAAGTCTGTGTCTGA#....(((((((((((((((((#))))))).....................))))))))))).	331	370	0.84615	-24	0	0.0002919	0.6	-0	-0.2	-10	18	39	11	5UTR
mmu-miR-214-3p	NM_001285991	Tbc1d5	ACAGCAGGCACAGACAGGCAGT#ACTGATCCATCCAGGTCTGGGACAGTGCCGGCCATGTCTGTCACTGCTGA#.((((((..((((((((((.((#.))..))).)))))))..)))))).	4322	4372	0.80769	-27	1	0.0003899	0.4	4.2	4.2	-7	18	25	7	3UTR
mmu-miR-214-3p	NM_001285993	Tbc1d5	ACAGCAGGCACAGACAGGCAGT#ACTGTCTCCACTCTATGCAATGTATAAGTCTGTGTCTGA#....(((((((((((((((((#))))))).....................))))))))))).	298	337	0.84615	-24	0	0.0002919	0.6	-0	-0.2	-10	18	39	11	5UTR

mmu-miR-214-3p	NM_001285993	Tbc1d5	ACAGCAGGCACAGACAGGCAGT#ACTGATCCATCCAGGTCTGGGACAGTGCCGGCCATGTCTGTCACTGCTGA#.((((((..(((((((((((.((#.))..))).)))))))..)))))).	4223	4273	0.80769	-27	1	0.0003899	0.4	3.9	3.77	-7	18	25	7	3UTR
mmu-miR-214-3p	NM_001285993	Tbc1d5	ACAGCAGGCACAGACAGGCAGT#GCTGCTGTTTGGCAGAGAGTTCCCTCTGCAGGACCTGCTGG#.(((((((..((((((.(((((#)))))))))))).......................))))))).	1324	1365	0.84615	-23	1	4.41E-06	0.4	-0	-0.3	-6	18	41	11	CDS
mmu-miR-214-3p	NM_001286040	Anks1	ACAGCAGGCACAGACAGGCAGT#CTGCGGGCTGTCTGCCTGCTGC#.((((((((..((((((((((.#))))...)))))))))))))).	6013	6035	1	-28	1	8.56E-09	0.4	-0	-0.6	-8	18	22	14	3UTR
mmu-miR-214-3p	NM_001286040	Anks1	ACAGCAGGCACAGACAGGCAGT#CTGCCTCTCTCTGATGCTTGTTGA#.((((((((((((((.((((((.#))))))...)))).))))))))).	5542	5566	1	-27	1	0.0053076	0.4	0.3	0.16	-5	19	24	9	3UTR
mmu-miR-214-3p	NM_001286040	Anks1	ACAGCAGGCACAGACAGGCAGT#CTGCCTTTGGCGCTTGTGTGAAACCAAGGCTGTGCCTGAACTGTG#((((((((((((..((((((.#))))))......................)))))))))..)))).	6488	6533	0.84615	-26	1	0.000572	0.5	0.5	-0.1	-6	19	45	9	3UTR
mmu-miR-214-3p	NM_001286041	Anks1	ACAGCAGGCACAGACAGGCAGT#CTGCGGGCTGTCTGCCTGCTGC#.((((((((..((((((((((.#))))...)))))))))))))).	5950	5972	1	-28	1	8.56E-09	0.4	-0	-0.6	-8	18	22	14	3UTR
mmu-miR-214-3p	NM_001286041	Anks1	ACAGCAGGCACAGACAGGCAGT#CTGCCTCTCTCTGATGCTTGTTGA#.((((((((((((((.((((((.#))))))...)))).))))))))).	5479	5503	1	-27	1	0.0053076	0.4	0.3	0.16	-5	19	24	9	3UTR
mmu-miR-214-3p	NM_001286041	Anks1	ACAGCAGGCACAGACAGGCAGT#CTGCCTTTGGCGCTTGTGTGAAACCAAGGCTGTGCCTGAACTGTG#((((((((((((..((((((.#))))))......................)))))))))..)))).	6425	6470	0.84615	-26	1	0.000572	0.5	0.5	-0.1	-6	19	45	9	3UTR
mmu-miR-214-3p	NM_001286122	Traf3	ACAGCAGGCACAGACAGGCAGT#GCTGCCATCTGTCCTGGGTGTGCCTTCGCCCCAGTGCCTGCTGG#.((((((((((.((((((((((#))))))...))))...................))))))))))).	3717	3761	0.84615	-30	1	4.61E-06	0.3	1.5	1.46	-7	20	44	10	3UTR
mmu-miR-214-3p	NM_001286178	Eif4e1b	ACAGCAGGCACAGACAGGCAGT#ACTGCCACAGCTCATCACTGTGCCTGCA#...((((((((((...((((((#))))))...........)))))))))).	1004	1032	0.92308	-27	0	0.0130548	0.5	0	-0.2	-8	16	28	10	3UTR

mmu-miR-214-3p	NM_001286178	Eif4e1b	ACAGCAGGCACAGACAGGCAGT#ACTGCGCCACTTCGAACTGGACCGTCTGTGGCTGGAGACGTTGCTGTG#(((((((.((((((((.((((((#.))))..))))))))).)).........))))).	476	524	1	-23	1	0.0064849	0.5	1	3.37	-6	20	34	9	CDS
mmu-miR-214-3p	NM_001286179	Eif4e1b	ACAGCAGGCACAGACAGGCAGT#ACTGCCACAGCTCATCACTGTGCCTGCA#...((((((((((...((((((#))))))...........)))))))))).	1007	1035	0.92308	-27	0	0.0130548	0.5	0	-0.2	-8	16	28	10	3UTR
mmu-miR-214-3p	NM_001286179	Eif4e1b	ACAGCAGGCACAGACAGGCAGT#ACTGCGCCACTTCGAACTGGACCGTCTGTGGCTGGAGACGTTGCTGTG#(((((((.((((((((.((((((#.))))..))))))))).)).........))))).	479	527	1	-23	1	0.0064849	0.5	1	3.37	-6	20	34	9	CDS
mmu-miR-214-3p	NM_001286180	Eif4e1b	ACAGCAGGCACAGACAGGCAGT#ACTGCCACAGCTCATCACTGTGCCTGCA#...((((((((((...((((((#))))))...........)))))))))).	966	994	0.92308	-27	0	0.0130548	0.5	0	-0.2	-8	16	28	10	3UTR
mmu-miR-214-3p	NM_001286255	Nadk2	ACAGCAGGCACAGACAGGCAGT#ACGCGTGTGCTCTCCTGCTGA#.(((((((.....(((.(((.((#)))).)))......))))))).	1580	1601	1	-19	1	0.0006188	0.5	0.2	0.56	-14	14	21	7	3UTR
mmu-miR-214-3p	NM_001286343	Bcl11b	ACAGCAGGCACAGACAGGCAGT#ATTGCTTGGTATTTGTGCTCTGCTT#..(((((((((((((((((((#))))))))...))))))).))))).	7050	7075	1	-25	1	0.0005827	0.7	4.9	3.43	-5	20	25	8	3UTR
mmu-miR-214-3p	NM_001286343	Bcl11b	ACAGCAGGCACAGACAGGCAGT#GGTGCTTTCTGTGTCTGCC#...(((((((((((.(((((.(#).)))))))))))))))).	6882	6901	1	-25	0	0.013824	0.5	3.1	4.03	-7	17	19	16	3UTR
mmu-miR-214-3p	NM_001286343	Bcl11b	ACAGCAGGCACAGACAGGCAGT#GCCTAACCTGTGTCTGCG#...((((((((((..(((((...#))))...))))))))))).	2467	2485	1	-25	0	0.000411	0.4	3.1	3.07	-8	14	18	10	3UTR
mmu-miR-214-3p	NM_001286539	Sigmar1	ACAGCAGGCACAGACAGGCAGT#GCCTTTAGTGTGTGTGTGCA#...(((.(((((.((((((...#))))...)).))))).))).	1446	1466	0.87179	-22	0	6.49E-06	0.5	0.1	0.29	-9	14	20	5	3UTR
mmu-miR-214-3p	NM_001286544	Ccpg1	ACAGCAGGCACAGACAGGCAGT#GCTGGCTTGTCTTGTGAACTCTCTTGCA#...((((((((((((((((((#)))).))))))).)))......))))).	2991	3019	1	-21	0	0.0078911	0.6	2.1	1.78	-7	19	28	7	3UTR
mmu-miR-214-3p	NM_001286545	Ccpg1	ACAGCAGGCACAGACAGGCAGT#GCTGGCTTGTCTTGTGAACTCTCTTGCA#...((((((((((((((((((#)))).))))))).)))......))))).	2523	2551	1	-21	0	0.0114652	0.6	2.1	2.01	-7	19	28	7	3UTR
mmu-miR-214-3p	NM_001286553	Ppp2r1b	ACAGCAGGCACAGACAGGCAGT#TGCACTGTCTGAAGCCTGTGGTA#((.(((((((.((((((((((..#))).)))))))..)))))).)).	1614	1637	1	-26	0	4.26E-05	0.5	3.5	3.95	-7	18	23	7	CDS

mmu-miR-214-3p	NM_001286553	Ppp2r1b	ACAGCAGGCACAGACAGGCAGT#GCTGCTGGCTGGCCAGCTGG#.((((.(((.(((.(.((((((#)))))).))))))).)))).	1380	1400	0.84615	-25	0	8.14E-07	0.5	2.4	4.37	-8	17	20	7	CDS
mmu-miR-214-3p	NM_001286553	Ppp2r1b	ACAGCAGGCACAGACAGGCAGT#TGCCTAGTCTTCCCTTGCCTGCCTGG#.(((((((((.(((((((((..#))))).))))).....))))))).)).	3321	3347	1	-25	1	0.0001749	0.5	0.1	0.45	-7	18	26	7	3UTR
mmu-miR-214-3p	NM_001286662	Pdpk1	ACAGCAGGCACAGACAGGCAGT#CTGTTTTTCTGTGCTCCAGCTGG#.((((.((((((((.((((((.#)))))).))))))))...)))).	4323	4346	0.88462	-25	0	0.0013956	0.4	0.4	-0.2	-6	18	23	8	3UTR
mmu-miR-214-3p	NM_001286684	Slc8a1	ACAGCAGGCACAGACAGGCAGT#TGTCTGTCTGTCCTCTGG#.(((.(((.(((((((((((..#))))))))))))))))).	12375	####	1	-26	0	0.0009438	0.6	0.8	0.08	-9	17	18	17	3UTR
mmu-miR-214-3p	NM_001286684	Slc8a1	ACAGCAGGCACAGACAGGCAGT#ACTGCCGTTGTGTTTGTTGC#.(((((((((((.((.((((((#))))))))))))))))))).	2606	2626	1	-25	1	0.0018778	0.5	0.7	0.42	-5	19	20	19	CDS
mmu-miR-214-3p	NM_001286684	Slc8a1	ACAGCAGGCACAGACAGGCAGT#GCTGCTTGTCCATTTGCTCCGGCTGG#.((((.((((..((((((((((#))))))))))....))))...)))).	12511	####	0.92308	-25	0	0.0001054	0.4	1.1	0.86	-12	18	26	10	3UTR
mmu-miR-214-3p	NM_001286684	Slc8a1	ACAGCAGGCACAGACAGGCAGT#TTGTCTGTTGTGTGATGCTGTG#((((((.((((((.((((((((.#)))))))))))))..)))))).	3477	3499	0.96154	-25	0	0.0037416	0.5	-0	-0.1	-7	19	22	13	3UTR
mmu-miR-214-3p	NM_001286785	Magi1	ACAGCAGGCACAGACAGGCAGT#ACCGCCATGCTGTGCCTTGCA#...((((((((((.(((((.((#)).))).)))))))))).)).	1007	1028	0.92308	-24	0	7.70E-05	0.4	3.4	2.48	-7	17	21	10	CDS
mmu-miR-214-3p	NM_001286785	Magi1	ACAGCAGGCACAGACAGGCAGT#GTCTTGTGCCTGGTGTA#(((.(((((((((((.......#)))).))))))).))).	5303	5320	1	-24	0	0.1128847	0.8	3.9	3.86	-7	14	17	7	3UTR
mmu-miR-214-3p	NM_001286785	Magi1	ACAGCAGGCACAGACAGGCAGT#ACTGCCTAGCTGAGCCAAAGTGTTTGCTTGTA#((((((((((.(((..(((((((#)))))))..))).)))).......	6396	6428	0.84615	-23	1	0.0073733	0.5	5.5	3.95	-7	19	32	7	3UTR
mmu-miR-214-3p	NM_001286786	Magi1	ACAGCAGGCACAGACAGGCAGT#ACCGCCATGCTGTGCCTTGCA#...((((((((((.(((((.((#)).))).)))))))))).)).	1007	1028	0.92308	-24	0	7.70E-05	0.4	0.4	0.4	-7	17	21	10	CDS
mmu-miR-214-3p	NM_001286786	Magi1	ACAGCAGGCACAGACAGGCAGT#GCCTCTGGAAGAATGTGAAGATGATGAGTTGCCTGCTGG#.((((((((((((((..(((...#))))))).....................))))))))).	1611	1650	0.92308	-24	1	0.001867	0.5	3.9	4.29	-5	16	39	9	CDS

mmu-miR-214-3p	NM_001286809	Camk2a	ACAGCAGGCACAGACAGGCAGT#GCTGCCTGCAAATGCTGCTCTTTCTCACGCTGTGGGCCCTGGTGC#.((.((((((((.((((((((#))))))))).....................)))))...)))).)).	282	327	0.84615	-24	0	7.81E-06	0.5	1.2	0.99	-8	19	45	8	5UTR
mmu-miR-214-3p	NM_001286809	Camk2a	ACAGCAGGCACAGACAGGCAGT#GCTGCCTCTCCGTGCTTTTGG#.(((.((((((.((.(((((((#))))))).)).))))))))).	2608	2629	0.92308	-23	0	0.0175643	0.5	1.5	1.25	-12	18	21	9	3UTR
mmu-miR-214-3p	NM_001286809	Camk2a	ACAGCAGGCACAGACAGGCAGT#CTGCGTCCTTGCTTCGCAGAGATCCGCTCTTTGTCCGTGGAATGTGGCTGCTGG#.((((((.(((((((((((.(((#.))).))))))).......)))).))))))).	881	935	0.84615	-23	1	0.0008487	0.5	0.5	1.81	-6	19	30	6	3UTR
mmu-miR-214-3p	NM_001287139	Zzz3	ACAGCAGGCACAGACAGGCAGT#GCTGCTTGCCTGCATGTCTGCTGC#.((((((((((((.(((((((((#)))))))).)))..))))))))).	7070	7094	0.92308	-30	1	2.88E-07	0.4	0.1	0.25	-5	20	24	9	3UTR
mmu-miR-214-3p	NM_001287139	Zzz3	ACAGCAGGCACAGACAGGCAGT#GCTGCCAGTGTGTGCTGTGTGTTGG#.(((((((((((.((.((((((#)))))).)).)))))))...))))).	7118	7143	0.84615	-27	1	0.0003369	0.5	0.6	0.17	-5	19	25	6	3UTR
mmu-miR-214-3p	NM_001289674	Fbxo41	ACAGCAGGCACAGACAGGCAGT#ACGCGGGGTCTGTGCCTCTGC#.(((.((((((((((..(((.((#))))...))))))))))))).	216	237	0.84615	-24	0	3.86E-06	0.2	0.7	1.3	-8	17	21	13	5UTR
mmu-miR-214-3p	NM_001289675	Fbxo41	ACAGCAGGCACAGACAGGCAGT#ACGCGGGGTCTGTGCCTCTGC#.(((.((((((((((..(((.((#))))...))))))))))))).	216	237	0.84615	-24	0	2.43E-06	0.2	0.7	1.3	-8	17	21	13	5UTR
mmu-miR-214-3p	NM_001289748	Tgm6	ACAGCAGGCACAGACAGGCAGT#GCTGGCTCTGTGCTTGA#....((((((((((..(.((((#)))).))))))))))).	1603	1620	0.84615	-22	0	1.42E-06	0.3	1	0.85	-11	15	17	11	CDS
mmu-miR-214-3p	NM_001289749	Tgm6	ACAGCAGGCACAGACAGGCAGT#GCTGGCTCTGTGCTTGA#....((((((((((..(.((((#)))).))))))))))).	1260	1277	0.84615	-22	0	1.42E-06	0.3	1	0.85	-11	15	17	11	CDS
mmu-miR-214-3p	NM_001289775	Rufy3	ACAGCAGGCACAGACAGGCAGT#GCCTGGGGGACGGAAGCACTGTCTGTGTGCTTGCG#...((((((((..(((((((((#.))))))))))))	1977	2012	1	-27	0	0.0007071	0.4	0.9	0.47	-11	17	19	17	3UTR
mmu-miR-214-3p	NM_001289775	Rufy3	ACAGCAGGCACAGACAGGCAGT#GCTGCTCCTGCGTGCTCCTGCTGTA#((((((((..((..((((((((#)))))..)))..))..)))))))).	3518	3543	0.84615	-25	1	1.19E-06	0.4	1.1	0.67	-8	18	25	8	3UTR
mmu-miR-214-3p	NM_001289775	Rufy3	ACAGCAGGCACAGACAGGCAGT#CTGCCCTGCAGTCCTGCTGG#.(((((((.((.(.(((((((.#)))))).))).))))))))).	2465	2485	1	-24	1	6.63E-05	0.4	0.5	0.42	-9	17	20	9	3UTR

mmu-miR-214-3p	NM_001289776	Rufy3	ACAGCAGGCACAGACAGGCAGT#ACTGTCTGTGTGCTTGCG#...((((((((..(((((((((#))))))))))))))))).	2416	2434	0.92308	-26	0	0.0007071	0.4	0.3	0.28	-11	17	18	17	3UTR
mmu-miR-214-3p	NM_001289776	Rufy3	ACAGCAGGCACAGACAGGCAGT#TGTGTGTGTGTGTGTGTTGTG#((((((.(((((.(((.(((..#))).))).))))).)))))).	589	610	0.92308	-26	0	0.0913976	0.5	2.5	2.67	-7	17	21	6	5UTR
mmu-miR-214-3p	NM_001289776	Rufy3	ACAGCAGGCACAGACAGGCAGT#GCTGCTCCTGCGTGCTCCTGCTGTA#((((((((..((..((((((((#)))))..)))..))..)))))))).	3940	3965	0.84615	-25	1	1.19E-06	0.4	1.1	0.67	-8	18	25	8	3UTR
mmu-miR-214-3p	NM_001289776	Rufy3	ACAGCAGGCACAGACAGGCAGT#CTGCCCTGCAGTCCTGCTGG#.(((((((.(((.(.((((((((.#))))).))).))))))))).	2887	2907	1	-24	1	6.63E-05	0.4	0.5	0.42	-9	17	20	9	3UTR
mmu-miR-214-3p	NM_001289777	Rufy3	ACAGCAGGCACAGACAGGCAGT#GCCTGGGGGACGGAAGCACTGTCTGTGTGCTTGCG#...((((((((..((((((((((#.)))))))))))))	2224	2259	1	-27	0	0.0007071	0.4	0.9	0.47	-11	17	19	17	3UTR
mmu-miR-214-3p	NM_001289777	Rufy3	ACAGCAGGCACAGACAGGCAGT#GCTGCTCCTGCGTGCTCCTGCTGTA#((((((((..((..((((((((#)))))..)))..))..)))))))).	3765	3790	0.84615	-25	1	1.19E-06	0.4	1.1	0.67	-8	18	25	8	3UTR
mmu-miR-214-3p	NM_001289777	Rufy3	ACAGCAGGCACAGACAGGCAGT#CTGCCCTGCAGTCCTGCTGG#.(((((((.(((.(.((((((((.#))))).))).))))))))).	2712	2732	1	-24	1	6.63E-05	0.4	0.5	0.42	-9	17	20	9	3UTR
mmu-miR-214-3p	NM_001289916	Rora	ACAGCAGGCACAGACAGGCAGT#CTGTCTGTCTGTGTGAATTCTTCACCGTCTCATCTTGTTGA#.((((((((((((((((((((.#)))))))))))))).....................))))))).	6343	6384	1	-27	1	0.0019717	0.6	0.7	1.11	-4	20	41	13	3UTR
mmu-miR-214-3p	NM_001289916	Rora	ACAGCAGGCACAGACAGGCAGT#TGTGTGTGTGTGTGTGCG#...(((.(((((.(((.(((..#))).))).))))).))).	7401	7419	0.84615	-23	0	0.0050871	0.4	0.4	0.09	-9	14	18	5	3UTR
mmu-miR-214-3p	NM_001289924	Aopep	ACAGCAGGCACAGACAGGCAGT#ACTGCTGTGATTTATCTGTGCTAAAGGTAGAGGAGGTGGATGTGGCTGCTGTG#(((((((.((((..((((((((#))))))).............................))..)))).))))))).	707	760	0.84615	-26	1	0.0001882	0.5	4	1.87	-8	19	53	7	CDS
mmu-miR-214-3p	NM_001289926	Aopep	ACAGCAGGCACAGACAGGCAGT#ACTGCTGTGATTTATCTGTGCTAAAGGTAGAGGAGGTGGATGTGGCTGCTGTG#(((((((.((((..((((((((#))))))).............................))..)))).))))))).	707	760	0.84615	-26	1	0.0001882	0.5	4	1.87	-8	19	53	7	CDS

mmu-miR-214-3p	NM_001289926	Aopep	ACAGCAGGCACAGACAGGCAGT#GCAGTGAGAATGCCTGTCTGAAGGGCCTTGTCTGTA#((((((((.((((((((((..#.)))))))))).…)))).).)))).	5418	5454	0.84615	-28	1	1.43E-05	0.5	-1	-0.7	-5	19	27	10	3UTR
mmu-miR-214-3p	NM_001290148	Snx29	ACAGCAGGCACAGACAGGCAGT#CTGCCTCAGTGCCTGA#....(((((((....((((((.#))))))..))))))).	397	413	0.92308	-22	0	0.0002215	0.4	1.5	2.18	-16	13	16	7	CDS
mmu-miR-214-3p	NM_001290299	Plppr2	ACAGCAGGCACAGACAGGCAGT#GCTCTCGCCTGGTGAAACCTTCCCTCTGCCTGGCCCTGCTGTG#((((((((......(((((((.#.)))))))..)))))))).	1027	1070	0.84615	-26	1	0.0008208	0.3	3.2	2.52	-15	15	19	8	CDS
mmu-miR-214-3p	NM_001290308	Col12a1	ACAGCAGGCACAGACAGGCAGT#AGTGCTCTGTCAGTGCCTGG#....(((((((.((((((((.(#).))).))))).))))))).	733	753	0.84615	-26	0	9.83E-05	0.5	0.6	0.49	-13	16	20	7	CDS
mmu-miR-214-3p	NM_001290308	Col12a1	ACAGCAGGCACAGACAGGCAGT#CTGCCTGAACGAGAGCCCTTCATCGTGCCTGTG#...((((((((.(((((((((.#)))))))).............)))))))))).	10727	####	0.84615	-24	0	0.0001435	0.4	4.5	2.37	-10	17	33	10	3UTR
mmu-miR-214-3p	NM_001290308	Col12a1	ACAGCAGGCACAGACAGGCAGT#ACAGCCTCTCTGTAGCCTGG#....((((((((((.((((.((#)).)))).))))).))))).	6460	6480	0.84615	-23	0	7.87E-05	0.4	2.7	1.74	-10	16	20	5	CDS
mmu-miR-214-3p	NM_001290335	Cacna1c	ACAGCAGGCACAGACAGGCAGT#CCTGTCATGTGCCATGCCTGTT#((((((((((((((((((....#)))))).)))))).))).))).	12280	####	0.96154	-26	1	0.0031596	0.5	3.4	1.98	-4	18	22	6	3UTR
mmu-miR-214-3p	NM_001290335	Cacna1c	ACAGCAGGCACAGACAGGCAGT#CCTGCTCTGTGTCTGCC#...((((((((((((((....#)))).))))))))))).	6827	6844	1	-26	0	0.0055044	0.6	2.8	3.03	-7	15	17	11	3UTR
mmu-miR-214-3p	NM_001290335	Cacna1c	ACAGCAGGCACAGACAGGCAGT#CTGCCTGCTGCGCCTTCTC#..((.((((.(((.(((((((.#)))))))))).)))).)).	12454	####	0.92308	-26	0	1.86E-05	0.4	0.3	0.2	-11	16	19	10	3UTR
mmu-miR-214-3p	NM_001290356	Sorcs1	ACAGCAGGCACAGACAGGCAGT#CTGTGGTGTTTGTGGGGCTCGCTGTA#(((((.((((((((((.((((.#))))..)))))))...))).))))).	3500	3526	0.84615	-22	0	0.0071401	0.5	0	0.63	-6	19	26	7	CDS
mmu-miR-214-3p	NM_001290413	Traf2	ACAGCAGGCACAGACAGGCAGT#GCTGCCTGTGTCTATGAAGGCCTGTA#...((((((..(.(((((((((#))))))))).)........)))))).	338	364	0.92308	-24	0	0.0089001	0.5	-0	-0	-12	16	26	9	CDS

mmu-miR-214-3p	NM_001290413	Traf2	ACAGCAGGCACAGACAGGCAGT#TGTCTGTGCTGAGTGGTGTTCATCTGGCTCCTGCTGTG#((((((((((((((((......#))))))))).....................))))))).	2177	2215	1	-23	1	0.0011057	0.5	1	0.73	-4	16	38	9	3UTR
mmu-miR-214-3p	NM_001290416	Eps8l1	ACAGCAGGCACAGACAGGCAGT#GCTCAGCCCGCGCTCTGAGGCCTCGGTGGTCCGTGCCTGGCTGC#.((((((((((.(((((((...#.)))).....))).))))))).))).	2022	2066	0.84615	-26	1	2.53E-06	0.3	0.6	1.27	-7	17	26	7	CDS
mmu-miR-214-3p	NM_001290508	Dolpp1	ACAGCAGGCACAGACAGGCAGT#GCTGAGTCAGGTCCTCAGCCAGGACCCTGGGAGGGCCTGCTGTG#(((((((((.....((((....#.)))).....))))))))).	1275	1319	0.92308	-24	1	1.61E-07	0.4	1.2	1.25	-13	13	20	9	3UTR
mmu-miR-214-3p	NM_001290509	Dolpp1	ACAGCAGGCACAGACAGGCAGT#GCTGAGTCAGGTCCTCAGCCAGGACCCTGGGAGGGCCTGCTGTG#(((((((((.....((((....#.)))).....))))))))).	939	983	0.92308	-24	1	1.61E-07	0.4	1.2	1.25	-13	13	20	9	3UTR
mmu-miR-214-3p	NM_001290546	Phf6	ACAGCAGGCACAGACAGGCAGT#ATTTCAGGTCTGTGTCTGCTT#..(((((((((((((..(.(((#))).)..))))))))))))).	4110	4131	1	-24	1	0.0066615	0.6	0	0	-5	17	21	13	3UTR
mmu-miR-214-3p	NM_001290558	Slc25a25	ACAGCAGGCACAGACAGGCAGT#GCTGAGCAACCGGTCTCACCAGCATGCTCTGCCTGTGCCTGTATGTG#((((((((((((.((((((((#.)).))).))).))))))))))).))).	91	138	0.92308	-28	1	0.000127	0.4	0.3	0.61	-5	21	27	10	5UTR
mmu-miR-214-3p	NM_001290558	Slc25a25	ACAGCAGGCACAGACAGGCAGT#GCTGCCCATGCTCAATGAGTGGCCTGCTGG#.((((((((.((..((((((((#))))))).........))..))))))))	2984	3014	0.92308	-27	1	5.25E-09	0.4	3.6	2.97	-7	18	30	10	3UTR
mmu-miR-214-3p	NM_001290558	Slc25a25	ACAGCAGGCACAGACAGGCAGT#GCTGCCTGCATGGGCCTAACGTCCTCTGAGCTGC#.((((((((.((..((((((((#))))))))..)).)))) ....)))).	2193	2227	0.84615	-26	1	0.0011327	0.4	4.8	3.56	-7	18	34	8	3UTR
mmu-miR-214-3p	NM_001290649	Golga1	ACAGCAGGCACAGACAGGCAGT#TTGCCTTCTGTGGTACCTGCTC#..((((((((((((.((((((.#))))))))))))...)))))).	3353	3375	1	-25	1	0.0034663	0.6	0.7	0.62	-6	18	22	12	3UTR
mmu-miR-214-3p	NM_001290649	Golga1	ACAGCAGGCACAGACAGGCAGT#CTGCCTCTGCCTCTGCCTCCAAGTGCTGA#.((((((((((((..(((((.#)))))))))....)))))......)))).	4635	4664	1	-21	1	0.0002933	0.5	0.4	0.7	-5	18	29	9	3UTR

mmu-miR-214-3p	NM_001290696	Foxj3	ACAGCAGGCACAGACAGGCAGT#TGTCTGTCACGGTGCCATGCA#...((((((((.((((((((..#))))))))...))))).))).	1695	1716	0.84615	-25	0	3.23E-06	0.4	2.8	3.14	-10	16	21	8	CDS
mmu-miR-214-3p	NM_001290696	Foxj3	ACAGCAGGCACAGACAGGCAGT#ACTGACTGCGTGACCTGCTGTT#(((((((((((.(.(((.((((#)))).))))))).)))))))).	3114	3136	0.84615	-25	1	0.0003494	0.4	0.5	0.05	-8	19	22	8	3UTR
mmu-miR-214-3p	NM_001290696	Foxj3	ACAGCAGGCACAGACAGGCAGT#CTGTGTGTGTGTGTGTGTGTGTG#((((((.(((((.(((.((((.#)))).))).))))).))).))).	3462	3485	1	-24	0	0.8502462	0.5	0.1	0.33	-7	18	23	5	3UTR
mmu-miR-214-3p	NM_001290717	Sytl4	ACAGCAGGCACAGACAGGCAGT#GCAGAGAAGAAATGCCTGTCAGTGACATCCAGCTGC#.((((.(((((.((((((((..#.)))))))).))))....)).))))).	3077	3113	1	-25	0	0.0005731	0.5	1.8	2.19	-9	17	25	8	3UTR
mmu-miR-214-3p	NM_001290717	Sytl4	ACAGCAGGCACAGACAGGCAGT#TGTCTGGGTGTGCTGCTA#..((((.(((((..((((((..#))))))..))))))))).	2728	2746	0.92308	-23	0	0.0010361	0.6	1.5	2.93	-9	15	18	9	3UTR
mmu-miR-214-3p	NM_001290719	Sytl4	ACAGCAGGCACAGACAGGCAGT#CTGCTGGTCTGTGTTGGAAGGAGCTTTGCATGGAGCTGTCTGTA#((((((((((((((.((((((.#))))).))))))))....................))).))))).	1729	1773	0.92308	-25	1	2.82E-05	0.5	2.1	2.91	-4	20	44	8	3UTR
mmu-miR-214-3p	NM_001290745	Nckap1	ACAGCAGGCACAGACAGGCAGT#CCATGTCTGTGACTTGCTGA#.(((((((((((((((((....#)).))))))))).))))))).	549	569	1	-26	1	0.0006263	0.6	1.6	2.13	-4	17	20	8	CDS
mmu-miR-214-3p	NM_001290745	Nckap1	ACAGCAGGCACAGACAGGCAGT#GCTGCCCGGCTGGCATCTGCACTGCCTTTACCCTGCTGTA#((((((((..(((.(.((((((#)))))).).)))...................))))))))).	4231	4271	0.84615	-24	1	0.0688861	0.5	-0	0.28	-7	18	40	8	3UTR
mmu-miR-214-3p	NM_001290745	Nckap1	ACAGCAGGCACAGACAGGCAGT#CTGGAATCTGCTGTGAAATTCATAGTCAGAAAATTTCCTGCTGTA#(((((((((((((.(((((((.#)))...))))))))).....................))))))))).	391	436	0.92308	-23	1	0.0009636	0.6	3.4	2.77	-5	20	45	9	CDS
mmu-miR-214-3p	NM_001290753	Ephb2	ACAGCAGGCACAGACAGGCAGT#GCTGCTCTGGGAGAGTGTGCCTGG#....((((((((..((((((((#))))).)))......)))))))).	6314	6338	0.92308	-25	0	0.0002628	0.4	0.3	0.07	-12	16	24	8	3UTR
mmu-miR-214-3p	NM_001290753	Ephb2	ACAGCAGGCACAGACAGGCAGT#GCCTGGCTCCCCCTGCTGG#.(((((((...((.(((((...#))))).))...))))))).	9377	9396	0.84615	-24	1	6.01E-06	0.4	3.7	3.71	-10	14	19	7	3UTR

mmu-miR-214-3p	NM_001290765	Slc39a13	ACAGCAGGCACAGACAGGCAGT#GCTGCCTGTCGCCTGG#....(((((...((((((((((#)))))))))))))))).	298	314	0.92308	-27	0	0.0006521	0.4	0.2	-0.1	-17	15	16	15	CDS
mmu-miR-214-3p	NM_001290765	Slc39a13	ACAGCAGGCACAGACAGGCAGT#GCATGCCTGGATGTCCCTGCC#...(((((.(((..((((((((#)).))))))..))).))))).	167	188	0.92308	-25	0	0.0006005	0.3	2.1	0.96	-10	16	21	6	5UTR
mmu-miR-214-3p	NM_001290765	Slc39a13	ACAGCAGGCACAGACAGGCAGT#GCCTTCTGGGGGCCTGCTT#..(((((((.((((.((((...#))))))))...))))))).	1067	1086	1	-24	1	8.15E-08	0.4	0.8	0.07	-7	15	19	8	CDS
mmu-miR-214-3p	NM_001290814	Aff3	ACAGCAGGCACAGACAGGCAGT#GCTGTACAGATGGTTGTGAGCCTTCATGTGCTTGTTGG#.(((((((((((((.((((...#.)))))).)))))))))))).	6513	6551	1	-25	1	0.0035453	0.5	1.2	2.11	-5	17	20	11	3UTR
mmu-miR-214-3p	NM_001290814	Aff3	ACAGCAGGCACAGACAGGCAGT#GCTGCCTGTGTATGTGTGTA#...(((.(((.(.(((((((((#)))))))))).).))).))).	3955	3975	1	-24	0	0.0146111	0.6	3.2	3.89	-12	16	20	9	3UTR
mmu-miR-214-3p	NM_001290824	Tubgcp4	ACAGCAGGCACAGACAGGCAGT#CTGCCACATTCTGATGTCTGTAACAAATCTCTCAAATCATTCATCAAATGCCTGCA#...((((((((((((((((((.#)))))).........)))))))...........................)))))).	2259	2315	0.84615	-25	0	0.0046998	0.6	-0	0.99	-7	18	56	7	3UTR
mmu-miR-214-3p	NM_001290824	Tubgcp4	ACAGCAGGCACAGACAGGCAGT#TTGTTTGCTGTACTGCTAAAGTGACAAGCCTGCTGA#.(((((((((((.(((((((.#))))))))))))).................)))))))).	3622	3658	1	-24	1	0.001646	0.6	0.3	0.37	-5	19	36	11	3UTR
mmu-miR-214-3p	NM_001290837	Serf2	ACAGCAGGCACAGACAGGCAGT#GCTTCGTGTCCAACCCTCTTGCCCTCCGCCTGTGTGCCTGGAGC#.(..(((((((..((((((...#.)))))))))))))..).	447	491	0.84615	-24	0	2.28E-05	0.4	2.1	2.64	-13	14	18	13	CDS
mmu-miR-214-3p	NM_001290837	Serf2	ACAGCAGGCACAGACAGGCAGT#CTGCCCTGGTTTGTTCTGTGGTC#((.(((((.((((((.(((((.#)))))...))))))))))).)).	1233	1256	0.92308	-20	0	0.0083991	0.4	0.6	0.73	-7	18	23	11	3UTR
mmu-miR-214-3p	NM_001290995	Kif1b	ACAGCAGGCACAGACAGGCAGT#CTGCCCTGTGTGGCTCCTGCTGG#.(((((((..((.((((((((.#))))).))).))...))))))).	9181	9204	0.92308	-28	1	6.64E-05	0.4	5	3.99	-7	17	23	7	3UTR
mmu-miR-214-3p	NM_001290995	Kif1b	ACAGCAGGCACAGACAGGCAGT#TGTGTGTCTGGTGTCTGTCTGTC#((((((((((((((((.(((..#))).))))))).)))))).)))).	6430	6453	0.84615	-27	1	0.1367421	0.5	4.1	4.47	-4	19	23	6	3UTR

mmu-miR-214-3p	NM_001290995	Kif1b	ACAGCAGGCACAGACAGGCAGT#TGTGTGTGTGTGTCTGTCTGTC#((((((((((((.(((.(((..#))).))).)))))))).)))).	6497	6519	0.84615	-27	1	0.311283	0.6	3.6	4.59	-5	18	22	8	3UTR
mmu-miR-214-3p	NM_001291016	Bcl2l11	ACAGCAGGCACAGACAGGCAGT#TTGCCCCTGATTCTGTCCTGCA#...(((((.((((((((((((.#)))))..))..)))))))))).	566	588	0.92308	-21	0	0.0045305	0.5	-0	0.01	-8	17	22	10	3UTR
mmu-miR-214-3p	NM_001291048	D3Ertd751e	ACAGCAGGCACAGACAGGCAGT#ACTTGGGATGGTTTCCCAGTGAGACATGAGCCAGTGTGTGTCAGGCTGA#.((((.((((((.((.(((...#.))).)).))))))..)))).	432	481	0.92308	-24	0	0.0001777	0.4	2.1	1.45	-7	15	21	6	CDS
mmu-miR-214-3p	NM_001291124	Tgif2	ACAGCAGGCACAGACAGGCAGT#GCTGCACTGTTACGGGGCCTGTACTCTGCTGA#.(((((((.(((((((((((((#))))).))))........)))).	986	1018	0.84615	-24	1	1.67E-05	0.4	0.3	0.74	-5	20	32	6	3UTR
mmu-miR-214-3p	NM_001291124	Tgif2	ACAGCAGGCACAGACAGGCAGT#ACTGCTACCCCACCTGTCTGTGAGACCCCATGATGGCTGTA#(((((...((((((((((((((#))))).......)))))))))............))))).	2446	2487	0.92308	-23	0	0.0001092	0.4	0.1	0.1	-10	19	41	9	3UTR
mmu-miR-214-3p	NM_001291124	Tgif2	ACAGCAGGCACAGACAGGCAGT#ACTGGCTTGGAGTGCCTGTTC#..(((((((((...((((((((#)))).))))..))))))))).	2161	2182	1	-23	1	2.21E-05	0.4	0.4	0.24	-10	17	21	9	3UTR
mmu-miR-214-3p	NM_001291137	Ralgapb	ACAGCAGGCACAGACAGGCAGT#GCTGCCTCAGGGTCGCCCTGTGCCCCCGCTT#..(((.(((((((((((((((((#)))))))....)))....)))))))..	4300	4331	0.92308	-24	0	0.0069695	0.4	2.5	3.56	-7	19	31	7	CDS
mmu-miR-214-3p	NM_001291137	Ralgapb	ACAGCAGGCACAGACAGGCAGT#AGTGTCTCTGTGTCTGGCTGA#.((((((((((((..((((.(#).))))))))))))))).))).	2829	2850	0.84615	-23	1	9.93E-06	0.5	5.2	4.36	-5	18	21	14	CDS
mmu-miR-214-3p	NM_001291137	Ralgapb	ACAGCAGGCACAGACAGGCAGT#TGCCTGTGTCCCTTGCTGC#.(((((((...(.((((((..#))))))).)..))))))).	8254	8273	0.84615	-22	1	0.0002727	0.5	1	2.16	-10	15	19	7	3UTR
mmu-miR-214-3p	NM_001291138	Ralgapb	ACAGCAGGCACAGACAGGCAGT#GCTGCCTCAGGGTCGCCCTGTGCCCCCGCTT#..(((.(((((((((((((((((#)))))))....)))....)))))))..	4264	4295	0.92308	-24	0	0.0069695	0.4	2.5	3.56	-7	19	31	7	CDS
mmu-miR-214-3p	NM_001291138	Ralgapb	ACAGCAGGCACAGACAGGCAGT#AGTGTCTCTGTGTCTGGCTGA#.((((((((((((..((((.(#).))))))))))))))).))).	2793	2814	0.84615	-23	1	9.93E-06	0.5	5.2	4.36	-5	18	21	14	CDS
mmu-miR-214-3p	NM_001291138	Ralgapb	ACAGCAGGCACAGACAGGCAGT#TGCCTGTGTCCCTTGCTGC#.(((((((...(.((((((..#))))))).)..))))))).	8218	8237	0.84615	-22	1	0.0002727	0.5	1	2.16	-10	15	19	7	3UTR

mmu-miR-214-3p	NM_001291144	Dhx35	ACAGCAGGCACAGACAGGCAGT#GCTCTCTTGGCCTCCTGTGTCTTGCCTGCTGTG#((((((((((.((((((((...#.)))).....))))))))))))))	2567	2600	0.92308	-28	1	0.0016528	0.4	0.2	-0.3	-7	18	25	14	3UTR
mmu-miR-214-3p	NM_001291145	Emilin3	ACAGCAGGCACAGACAGGCAGT#GTCTCTCTGTGCCTATGTG#(((..(((((((((.((((...#)))).))))))))).))).	332	351	0.84615	-23	0	5.28E-05	0.4	0.8	-0	-12	16	19	9	CDS
mmu-miR-214-3p	NM_001291171	Nfatc2	ACAGCAGGCACAGACAGGCAGT#GCTGCCCTCTACCAGAGAAGCAAGAGCCTGAGTCCCGGCCTGCTGG#.((((((((...(((((((...#.))))..)))...)))))))).	1273	1319	0.92308	-26	1	2.90E-06	0.4	0.2	-0.1	-11	15	22	8	CDS
mmu-miR-214-3p	NM_001291172	Nfatc2	ACAGCAGGCACAGACAGGCAGT#CTGTCTGTCTGCTCTCTCTTGGGTCCTCTGG#.(((.(((..((((((((((.#))))))))))))............))))	2248	2279	1	-22	0	0.000333	0.5	2.4	1.84	-10	17	31	11	3UTR
mmu-miR-214-3p	NM_001291212	Myo18a	ACAGCAGGCACAGACAGGCAGT#TGCCTCCCCTCTGCTGGGGTGAGGGCTTTCTCTTCTCAGTGCCTGCTGTC#((((((((((((.((..(((((.#.)))))...................)).))))))))))).	6610	6660	0.84615	-26	1	0.0078529	0.6	1.9	1.02	-8	18	41	11	3UTR
mmu-miR-214-3p	NM_001291212	Myo18a	ACAGCAGGCACAGACAGGCAGT#CTGCTTGTCCTTAGCACCCGAGGGGCTCCTGCTGTG#((((((((....(((((((((.#))))))))).................)))))))).	1440	1476	0.92308	-25	1	9.43E-06	0.5	2.8	3.33	-12	17	36	9	CDS
mmu-miR-214-3p	NM_001291212	Myo18a	ACAGCAGGCACAGACAGGCAGT#GCTGCTGTCTGGGCCTAAGCCGG#.(.((((((.((((((.(((((#)))))))))))).)))))..)).).	3589	3612	0.84615	-24	0	6.93E-06	0.4	2.5	3.37	-7	18	23	11	CDS
mmu-miR-214-3p	NM_001291213	Myo18a	ACAGCAGGCACAGACAGGCAGT#GCTGGCGTCTGTGCTCGGGCCTCCTGCTGG#.(((((((((((((.(.((((#)))).)))))))))).........))))	1191	1221	1	-28	1	8.65E-06	0.4	2.5	2.02	-4	19	30	9	CDS
mmu-miR-214-3p	NM_001291213	Myo18a	ACAGCAGGCACAGACAGGCAGT#TGCCTCCCCTCTGCTGGGGTGAGGGCTTTCTCTTCTCAGTGCCTGCTGTC#((((((((((((.((..(((((.#.)))))...................)).))))))))))).	6718	6768	0.84615	-26	1	0.0078529	0.6	1.9	1.02	-8	18	41	11	3UTR
mmu-miR-214-3p	NM_001291213	Myo18a	ACAGCAGGCACAGACAGGCAGT#CTGCTTGTCCTTAGCACCCGAGGGGCTCCTGCTGTG#((((((((....(((((((((.#))))))))).................)))))))).	1548	1584	0.92308	-25	1	9.43E-06	0.5	2.8	3.33	-12	17	36	9	CDS

mmu-miR-214-3p	NM_001291214	Myo18a	ACAGCAGGCACAGACAGGCAGT#TGCCTCCCCTCTGCTGGGGTGAGGGCTTTCTCTTCTCAGTGCCTGCTGTC#(((((((((((.((..(((((.#.)))))...................)).))))))))))).	5565	5615	0.84615	-26	1	0.0078529	0.6	1.9	1.02	-8	18	41	11	3UTR
mmu-miR-214-3p	NM_001291214	Myo18a	ACAGCAGGCACAGACAGGCAGT#CTGCTTGTCCTTAGCACCCGAGGGGCTCCTGCTGTG#((((((((....(((((((((.#))))))))).................)))))))).	407	443	0.92308	-25	1	9.43E-06	0.5	2.8	3.33	-12	17	36	9	CDS
mmu-miR-214-3p	NM_001291214	Myo18a	ACAGCAGGCACAGACAGGCAGT#GCTGCTGTCTGGGCCTAAGCCGG#.(.((((((.((((((.(((((#))))))))))).)))))..)).).	2544	2567	0.84615	-24	0	6.93E-06	0.4	2.5	3.37	-7	18	23	11	CDS
mmu-miR-214-3p	NM_001291215	Myo18a	ACAGCAGGCACAGACAGGCAGT#TGCCTCCCCTCTGCTGGGGTGAGGGCTTTCTCTTCTCAGTGCCTGCTGTC#(((((((((((.((..(((((.#.)))))...................)).))))))))))).	5631	5681	0.84615	-26	1	0.0078529	0.6	1.9	1.02	-8	18	41	11	3UTR
mmu-miR-214-3p	NM_001291215	Myo18a	ACAGCAGGCACAGACAGGCAGT#CTGCTTGTCCTTAGCACCCGAGGGGCTCCTGCTGTG#((((((((....(((((((((.#))))))))).................)))))))).	443	479	0.92308	-25	1	9.43E-06	0.5	2.8	3.33	-12	17	36	9	CDS
mmu-miR-214-3p	NM_001291215	Myo18a	ACAGCAGGCACAGACAGGCAGT#GCTGCTGTCTGGGCCTAAGCCGG#.(.((((((.((((((.(((((#))))))))))).)))))..)).).	2610	2633	0.84615	-24	0	6.93E-06	0.4	2.5	3.37	-7	18	23	11	CDS
mmu-miR-214-3p	NM_001291222	Kif7	ACAGCAGGCACAGACAGGCAGT#ACTGCCTGTTTTGCTTCTA#..((.(((((.(((((((((((#))))))))))))))))))).	4161	4180	1	-23	0	0.0005563	0.5	2.8	3.69	-12	18	19	18	3UTR
mmu-miR-214-3p	NM_001291222	Kif7	ACAGCAGGCACAGACAGGCAGT#TGACCTGCTGGACTGCCTGGTGC#.((.(((((((((.((((((..#)).)))))))...)))))).)).	468	491	1	-23	0	0.0002488	0.5	2.3	0.84	-8	17	23	7	CDS
mmu-miR-214-3p	NM_001291248	Cpsf4	ACAGCAGGCACAGACAGGCAGT#GCTGCTTGTCTGGGCTTGCTTGTG#(((((((((.((((((((((((#)))))))))))).))))))).)).	1455	1479	0.84615	-33	1	6.13E-05	0.4	-0	0.06	-5	21	24	12	3UTR
mmu-miR-214-3p	NM_001291249	Cpsf4	ACAGCAGGCACAGACAGGCAGT#GCTGCTTGTCTGGGCTTGCTTGTG#(((((((((.((((((((((((#)))))))))))).))))))).)).	1452	1476	0.84615	-33	1	6.13E-05	0.4	-0	0.06	-5	21	24	12	3UTR
mmu-miR-214-3p	NM_001291280	Nmb	ACAGCAGGCACAGACAGGCAGT#GCCTGTTTGGCACAGCC#...((.(((.(((((((((...#)))))))))).).)).	68	85	0.92308	-21	0	6.76E-05	0.4	0	0.84	-10	14	17	11	5UTR

mmu-miR-214-3p	NM_001291280	Nmb	ACAGCAGGCACAGACAGGCAGT#GTCTTTCCTGCTGTA#((((((((.(.((((.......#)))).))))))))).	619	634	0.92308	-19	1	0.0004857	0.6	-0	0.1	-8	13	15	9	3UTR
mmu-miR-214-3p	NM_001291442	Micu1	ACAGCAGGCACAGACAGGCAGT#ACTGCCTGGGACTTTGCTCTGCC#...(((((((.((.((((((((#))))))))...)).))).)))).	1548	1571	0.92308	-24	0	5.18E-05	0.5	3.3	2.95	-10	17	23	8	CDS
mmu-miR-214-3p	NM_001291442	Micu1	ACAGCAGGCACAGACAGGCAGT#CTGCTCTGACATCTGTCTCTCGCTGTG#((((((((.((((((((((((.#)))).)))...))))).))).)))))).	1890	1917	0.84615	-23	1	0.0006184	0.5	-0	0.14	-5	20	27	5	3UTR
mmu-miR-214-3p	NM_001291442	Micu1	ACAGCAGGCACAGACAGGCAGT#CTGCCATTTTCCTCCTGCTGTT#((((((((...(((..(((((.#)))))..)))...)))))))).	1941	1963	0.92308	-22	1	0.0060417	0.5	-0	0.23	-10	16	22	8	3UTR
mmu-miR-214-3p	NM_001291443	Micu1	ACAGCAGGCACAGACAGGCAGT#ACTGCCTGGGACTTTGCTCTGCC#...(((((((.((.((((((((#))))))))...)).))).)))).	1542	1565	0.92308	-24	0	5.18E-05	0.5	3.3	2.95	-10	17	23	8	CDS
mmu-miR-214-3p	NM_001291443	Micu1	ACAGCAGGCACAGACAGGCAGT#CTGCTCTGACATCTGTCTCTCGCTGTG#((((((((.((((((((((((.#)))).)))...))))).))).)))))).	1884	1911	0.84615	-23	1	0.0006184	0.5	-0	0.14	-5	20	27	5	3UTR
mmu-miR-214-3p	NM_001291443	Micu1	ACAGCAGGCACAGACAGGCAGT#CTGCCATTTTCCTCCTGCTGTT#((((((((...(((..(((((.#)))))..)))...)))))))).	1935	1957	0.92308	-22	1	0.0060417	0.5	-0	0.23	-10	16	22	8	3UTR
mmu-miR-214-3p	NM_001293300	Clasp1	ACAGCAGGCACAGACAGGCAGT#CCTGTCTGGCCCAGGTGCTGC#.((((((((.(((((((((....#)))))))))))....))))).	767	788	1	-26	1	3.77E-06	0.4	2.6	2.95	-5	16	21	11	CDS
mmu-miR-214-3p	NM_001293300	Clasp1	ACAGCAGGCACAGACAGGCAGT#TTGCACGTGTCTGCACCACCTGCTGA#.(((((((..(((((((((((.#)))).).))))))).....))))))).	5603	5629	0.84615	-23	1	0.0005851	0.5	0.6	1.25	-6	18	26	7	3UTR
mmu-miR-214-3p	NM_001293301	Clasp1	ACAGCAGGCACAGACAGGCAGT#CCTGTCTGGCCCAGGTGCTGC#.((((((((.(((((((((....#)))))))))))....))))).	767	788	1	-26	1	3.77E-06	0.4	2.6	2.95	-5	16	21	11	CDS
mmu-miR-214-3p	NM_001293301	Clasp1	ACAGCAGGCACAGACAGGCAGT#TTGCACGTGTCTGCACCACCTGCTGA#.(((((((..(((((((((((.#)))).).))))))).....))))))).	5303	5329	0.84615	-23	1	0.0005851	0.5	0.6	1.25	-6	18	26	7	3UTR
mmu-miR-214-3p	NM_001293727	Cgn	ACAGCAGGCACAGACAGGCAGT#CTGCTCTGTCTTGCCTGCTT#..((((((((.((((((((((.#)))).))))))))))))))).	3731	3751	0.92308	-30	1	0.0109007	0.5	0.3	1.75	-8	18	20	14	3UTR

mmu-miR-214-3p	NM_001294149	Kif1a	ACAGCAGGCACAGACAGGCAGT#CTGACCTGTACATATGTGGTTGCTGTG#(((((((.(((((((((((((.#))).))))).)...)))).))))))).	8087	8114	0.82051	-25	1	0.0012501	0.4	3.7	1.83	-6	20	27	7	3UTR
mmu-miR-214-3p	NM_001294149	Kif1a	ACAGCAGGCACAGACAGGCAGT#GCTGATGAAGTTAACAACAACACCTGTTCAGCTGTGCCTCCTGA#.(((.(((((((((((((....#.)))))....)))))))).))).	3039	3083	0.84615	-24	0	0.0001367	0.5	3.8	2.99	-8	16	23	8	CDS
mmu-miR-214-3p	NM_001294150	Kif1a	ACAGCAGGCACAGACAGGCAGT#CTGACCTGTACATATGTGGTTGCTGTG#(((((((.(((((((((((((.#))).))))).)...)))).))))))).	7975	8002	0.89744	-25	1	0.0012501	0.4	3.7	4.12	-6	20	27	7	3UTR
mmu-miR-214-3p	NM_001294150	Kif1a	ACAGCAGGCACAGACAGGCAGT#GCTGCCCTGGATGGGCCTCTGG#.(((.((((.((..((((((((#)))))).))..)).))))))).	3023	3045	0.84615	-25	0	1.16E-05	0.4	1.8	2.28	-11	17	22	7	CDS
mmu-miR-214-3p	NM_001294150	Kif1a	ACAGCAGGCACAGACAGGCAGT#GCTGATGAAGTTAACAACAACACCTGTTCAGCTGTGCCTCCTGA#.(((.(((((((((((((....#.)))))....)))))))).))).	2954	2998	0.92308	-24	0	0.0001367	0.5	-0	0.52	-8	16	23	8	CDS
mmu-miR-214-3p	NM_001294262	Agbl5	ACAGCAGGCACAGACAGGCAGT#GCTTGCCTGTTTCCTGCTC#..((((((...((((((((((((#))).)))))))))))))).	2113	2132	0.92308	-23	1	0.0002095	0.5	0.8	1.63	-10	17	19	14	CDS
mmu-miR-214-3p	NM_001294262	Agbl5	ACAGCAGGCACAGACAGGCAGT#GCCTGACTGTGCTG#......(((((((.(((((...#))))).))))))).	413	427	1	-22	0	0.0003434	0.5	4.1	3.33	-16	12	14	7	CDS
mmu-miR-214-3p	NM_001297554	Apela	ACAGCAGGCACAGACAGGCAGT#ACGAGGTTAAAGCACATGCGTTTGTCTGAATAACAATCTCTCCTGCTGTT#((((((((..(((((((((.((#.)))))))))))............)))))))).	604	654	1	-22	1	0.0292141	0.6	1.5	1.34	-6	19	34	11	3UTR
mmu-miR-214-3p	NM_001297591	Inpp4b	ACAGCAGGCACAGACAGGCAGT#ATTGTCTTCAGTCAGGCGCTTGCTGG#.((((((((.(.((((((((((#)))))))...)))..).)))))))).	2285	2311	1	-23	1	4.19E-05	0.6	3.5	3.13	-8	19	26	8	CDS
mmu-miR-214-3p	NM_001297591	Inpp4b	ACAGCAGGCACAGACAGGCAGT#GCTGATCGAAATGTGTCTGCTT#..((((((((((....((((((#)))).))....)))))))))).	3677	3699	0.92308	-22	1	0.00675	0.6	0.3	0.77	-8	16	22	10	3UTR
mmu-miR-214-3p	NM_001297596	Inpp4b	ACAGCAGGCACAGACAGGCAGT#ATTGTCTTCAGTCAGGCGCTTGCTGG#.((((((((.(.((((((((((#)))))))...)))..).)))))))).	2285	2311	1	-23	1	4.19E-05	0.6	3.5	3.13	-8	19	26	8	CDS

mmu-miR-214-3p	NM_001301424	Slc7a1	ACAGCAGGCACAGACAGGCAGT#ACTGCTTTGTTTGTGCCTTGAAGCTT#..((((((((((((((((((#))))))).))))))))))....))).	6526	6552	1	-27	1	0.001694	0.6	4	3.72	-5	20	26	10	3UTR
mmu-miR-214-3p	NM_001301424	Slc7a1	ACAGCAGGCACAGACAGGCAGT#GCTGCCTGGTGCCTTCAAGCACCAGGGCTGG#.((((((((((...((((((((#)))))))))))))))).........	1108	1139	0.84615	-27	1	5.74E-05	0.4	-0	-0.3	-9	18	31	14	CDS
mmu-miR-214-3p	NM_001301424	Slc7a1	ACAGCAGGCACAGACAGGCAGT#GCTGTGGTGCCTTTGCCTGCTGC#.(((((((((.((.((.(((((#)))))..)).)).))))))))).	3104	3127	0.92308	-26	1	0.0008232	0.4	-0	-0	-7	18	23	9	3UTR
mmu-miR-214-3p	NM_001301427	Vsx2	ACAGCAGGCACAGACAGGCAGT#CTGCATTCTCTGCCCTGCCTGCTGA#.(((((((((((((...((((.#))))....))))...))))))))).	1855	1880	1	-26	1	0.002908	0.5	-1	-0.1	-5	17	25	9	3UTR
mmu-miR-214-3p	NM_001301427	Vsx2	ACAGCAGGCACAGACAGGCAGT#ACTGCTGTTGTAGCCCATTGGTTCACATGTGCCTGCTT#..((((((((((((((.(((((#))))))))).................)))))))))).	2432	2470	1	-26	1	0.0020788	0.5	0.1	-0.1	-5	19	38	10	3UTR
mmu-miR-214-3p	NM_001301427	Vsx2	ACAGCAGGCACAGACAGGCAGT#CTGGACCCTGGCTGGCTCCTGCTGTG#((((((((..(((.(((((((.#)))...)))).)))...)))))))).	1759	1785	0.84615	-25	1	8.80E-09	0.4	-0	-0.2	-7	18	26	8	3UTR
mmu-miR-214-3p	NM_001301628	Usp15	ACAGCAGGCACAGACAGGCAGT#GCTGCCTGTCTGCGCCATCCGGGTACTTAGCTGCTGTG#(((((((((.((((((((((((#)))))))))))).)))..............)))))).	3386	3424	1	-35	1	0.0168787	0.4	1.2	0.49	-5	21	38	12	3UTR
mmu-miR-214-3p	NM_001301811	Irf8	ACAGCAGGCACAGACAGGCAGT#GCTGGCCTTTCTGTGTGCTGA#.((((..((((((((.(((((((#)))).))).)))))))))))).	2102	2123	1	-24	0	0.0010206	0.5	0.2	-0.1	-8	18	21	11	3UTR
mmu-miR-214-3p	NM_001301811	Irf8	ACAGCAGGCACAGACAGGCAGT#GCTGCCGTCTCTCCCTGAGCCAGCCGGGGCTGCCTAAGTTGTA#((((((((((((((.((((((#)))))))))))..................).)))))..))))).	1265	1308	0.84615	-22	1	0.0007772	0.3	0.9	1.91	-4	21	43	10	CDS
mmu-miR-214-3p	NM_001302475	Pld2	ACAGCAGGCACAGACAGGCAGT#ACTGCCTCCGCCAGCCTGCTGA#.((((((((......(((((((#)))))))......)))))))).	3646	3668	0.80769	-27	1	0.0012041	0.5	0.6	0.67	-14	15	22	8	3UTR
mmu-miR-214-3p	NM_001302475	Pld2	ACAGCAGGCACAGACAGGCAGT#CTGGATCTCCGAGACCCTGTCTGTGATGACTTCTTCCAGCTGTG#(((((.(((((((((((....#.))))))))))..........)).))))).	2870	2914	0.92308	-25	0	0.0003802	0.5	2.3	2.17	-6	17	30	10	CDS

mmu-miR-214-3p	NM_001302475	Pld2	ACAGCAGGCACAGACAGGCAGT#GCTCCTGGCTGAGTTCTGCCTGCTT#..(((((((((((.((((.(((#))))))).))).....)))))))).	13	38	0.92308	-24	1	2.54E-05	0.3	-0	0.31	-6	18	25	8	5UTR
mmu-miR-214-3p	NM_001302476	Pld2	ACAGCAGGCACAGACAGGCAGT#GCTCCTGGCTGAGTTCTGCCTGCTT#..(((((((((((.((((.(((#))))))).))).....)))))))).	13	38	0.92308	-24	1	2.54E-05	0.3	-0	0.31	-6	18	25	8	5UTR
mmu-miR-214-3p	NM_001302476	Pld2	ACAGCAGGCACAGACAGGCAGT#TGTGTGTGTGTGTGGTGTG#(((.((.(((((.(((.(((..#))).))).))))).)).))).	2941	2962	0.92308	-24	0	0.540478	0.6	0	0	-10	16	21	5	3UTR
mmu-miR-214-3p	NM_001302476	Pld2	ACAGCAGGCACAGACAGGCAGT#ACGCCATGGATGTGCTTGGTGC#.((.((((((((..(((((.((#))))).))..)))))))).)).	3158	3180	1	-24	0	1.39E-05	0.5	0.2	0.11	-9	17	22	8	3UTR
mmu-miR-214-3p	NM_001302476	Pld2	ACAGCAGGCACAGACAGGCAGT#GCTGCCATGTGGGGTGGGTGCTGG#.(((((..(((..(((((((((#)))))).)))...)))..))))).	3241	3265	0.84615	-24	0	9.67E-05	0.5	0	0.08	-12	17	24	6	3UTR
mmu-miR-214-3p	NM_001302888	Kcnq2	ACAGCAGGCACAGACAGGCAGT#GCTATGTCTGTTGTCTGCTGTA#((((((((((((((((((...#))).))))))).))))))))).	4314	4336	1	-30	1	0.009891	0.5	0.1	-0.1	-4	19	22	9	3UTR
mmu-miR-214-3p	NM_001302888	Kcnq2	ACAGCAGGCACAGACAGGCAGT#GCCTGTACACATGCCCGCCTTGTTGTGCCAGGCTGTG#(((((.((((((((((((...#))))))).................))))))..))))).	5981	6018	0.92308	-27	0	7.10E-07	0.4	2.1	1.67	-6	18	37	7	3UTR
mmu-miR-214-3p	NM_001302888	Kcnq2	ACAGCAGGCACAGACAGGCAGT#ATTGCCTCCATTGCTGTGCTGGCTGCTGG#.(((((((((((..(((((((#)))))))......))))))...)))))).	725	754	0.84615	-26	1	0.0001418	0.4	-0	0.24	-5	19	29	7	CDS
mmu-miR-214-3p	NM_001303132	Rd3	ACAGCAGGCACAGACAGGCAGT#GCTGCTACTGCTGTCCCTGCC#...(((((.((((.((((((((#)))))..)))))))).))))).	2974	2995	0.84615	-24	0	0.0021808	0.4	-0	-0	-8	17	21	7	3UTR
mmu-miR-214-3p	NM_001303132	Rd3	ACAGCAGGCACAGACAGGCAGT#CTGTCTGTCTGTCTGTC#...((((..((((((((((((.#)))))))))))))))).	3410	3427	1	-24	0	0.015112	0.5	0.3	0.2	-10	16	17	16	3UTR
mmu-miR-214-3p	NM_001303132	Rd3	ACAGCAGGCACAGACAGGCAGT#GCCTGCGAACCTGCTGC#.(((((((....(.(((((...#))))))...))))))).	1719	1736	1	-23	1	0.0005707	0.4	0.3	0	-13	13	17	7	3UTR
mmu-miR-214-3p	NM_001304572	Vezt	ACAGCAGGCACAGACAGGCAGT#ACCCTGAGATTTGCCTGTCTCTGCCTGCC#...(((((((.((((((((((.#.)))))))))).))))))).	7579	7608	0.92308	-32	0	2.77E-06	0.5	0.7	0.05	-9	17	20	10	3UTR

mmu-miR-214-3p	NM_001304572	Vezt	ACAGCAGGCACAGACAGGCAGT#GCCGTGTTGTGTGCCTGCC#...((((((((((((((((...#))).)))).))))))))).	6549	6568	0.84615	-27	0	0.004952	0.5	0.2	-0.1	-7	16	19	9	3UTR
mmu-miR-214-3p	NM_001304575	Vezt	ACAGCAGGCACAGACAGGCAGT#ACCCTGAGATTTGCCTGTCTCTGCCTGCC#...(((((((.((((((((((.#.)))))))))).))))))).	6588	6617	0.88462	-32	0	2.77E-06	0.5	-0	-0.1	-9	17	20	10	3UTR
mmu-miR-214-3p	NM_001304575	Vezt	ACAGCAGGCACAGACAGGCAGT#GCCGTGTTGTGTGCCTGCC#...((((((((((((((((...#))).)))).))))))))).	5558	5577	0.84615	-27	0	0.004952	0.5	1.1	0.98	-7	16	19	9	3UTR
mmu-miR-214-3p	NM_001304720	Vezt	ACAGCAGGCACAGACAGGCAGT#ACCCTGAGATTTGCCTGTCTCTGCCTGCC#...(((((((.((((((((((.#.)))))))))).))))))).	9424	9453	0.92308	-32	0	2.77E-06	0.5	0.6	0.24	-9	17	20	10	3UTR
mmu-miR-214-3p	NM_001304720	Vezt	ACAGCAGGCACAGACAGGCAGT#GCCGTGTTGTGTGCCTGCC#...((((((((((((((((...#))).)))).))))))))).	8394	8413	0.81538	-27	0	0.004952	0.5	1	1.01	-7	16	19	9	3UTR
mmu-miR-214-3p	NM_001305231	Retreg2	ACAGCAGGCACAGACAGGCAGT#GCTGCCTGTGCTGTGCTGGCTGTG#(((((.((((((((((((((((#))))))))).))))))).))))).	635	659	1	-37	0	0.0029183	0.4	1.9	3.8	-6	21	24	9	CDS
mmu-miR-214-3p	NM_001305231	Retreg2	ACAGCAGGCACAGACAGGCAGT#CTGCCTGCCTGCCTGCCTGCTGTC#(((((((((((((.(((((((.#))))))).)))..)))))))))).	1730	1754	0.92308	-34	1	0.0011505	0.4	1.2	0.81	-5	20	24	10	3UTR
mmu-miR-214-3p	NM_001305278	Cnr2	ACAGCAGGCACAGACAGGCAGT#GCCTTCTGTTCCATGCTGTG#((((((((.(((((.(((((...#)))))))))).)).))))))).	1142	1162	1	-25	1	0.0020866	0.5	2	2.04	-6	17	20	9	CDS
mmu-miR-214-3p	NM_001305278	Cnr2	ACAGCAGGCACAGACAGGCAGT#GCTTCCTGTTGCTGTGTGCATCCTGTTGTT#((((((((((((((((((.(((#))).)))))..))))).....))))))))	74	104	1	-24	1	0.005375	0.5	0.5	0.02	-4	21	30	8	5UTR
mmu-miR-214-3p	NM_001305436	Pter	ACAGCAGGCACAGACAGGCAGT#CCTGACTCTGTGATCTGCA#...((((((((((((((....#))))..)))))).))))).	1452	1471	0.92308	-21	0	0.0096744	0.6	0.4	0.9	-7	15	19	6	3UTR
mmu-miR-214-3p	NM_001305436	Pter	ACAGCAGGCACAGACAGGCAGT#GCTGCTGGAGTTTGCTCAACTTGGCTGCTA#..(((((.(((((((.((((((#))))))...)))))).......)).)))))	903	933	0.92308	-20	0	0.0025362	0.6	3.5	3.32	-8	18	30	6	CDS
mmu-miR-214-3p	NM_001305668	Vps28	ACAGCAGGCACAGACAGGCAGT#GCTGTATAGCTGCCTGGGCCTGCA#...(((((((.(...((((((((#.)))))))))))))))).	105	129	0.92308	-25	0	2.85E-07	0.4	-0	0.17	-13	15	17	15	5UTR

mmu-miR-214-3p	NM_001308144	Zcchc4	ACAGCAGGCACAGACAGGCAGT#CTGCCCCGCTGTGCCCGCA#...((.(((((((...(((((.#)))))...))))))).)).	139	158	0.84615	-26	0	2.59E-05	0.4	1.2	1.64	-10	14	19	7	CDS
mmu-miR-214-3p	NM_001308144	Zcchc4	ACAGCAGGCACAGACAGGCAGT#TGCCTGCTCAGCCTGTA#...((((((..((.((((((..#))))))))..)))))).	218	235	0.92308	-23	0	0.003095	0.6	4.6	3.42	-11	14	17	8	CDS
mmu-miR-214-3p	NM_001308144	Zcchc4	ACAGCAGGCACAGACAGGCAGT#GCTGTGCACTCCCAGACCATTCCTGTTTGGGGCCTAAAGATGGCTGC#.((((((((.((((((((((((#)))).................))))))))..)))).......)))).	1318	1365	1	-22	1	0.000535	0.5	2.1	3.14	-5	20	47	8	CDS
mmu-miR-214-3p	NM_001308482	Nsd3	ACAGCAGGCACAGACAGGCAGT#GCCGAGACCGGGGCTGGGTTGCCGGGTGTCTGCTGG#.((((((((((.....(((((.#.)))))..))))	32	68	0.84615	-25	1	1.18E-05	0.3	-0	0.37	-10	15	19	10	5UTR
mmu-miR-214-3p	NM_001309195	Zfp950	ACAGCAGGCACAGACAGGCAGT#TGTGCTTGCTGTC#((((((((((((.........#))))))))))))).	1210	1223	1	-20	1	0.0006299	0.7	0.3	0.08	-7	12	13	12	3UTR
mmu-miR-214-3p	NM_001310452	Mapk8	ACAGCAGGCACAGACAGGCAGT#CTGCCTGGACTGTGATGTCTGTT#((((((..(((((.((((((((.#)))))))..))))).)).)))).	5293	5316	0.84615	-25	0	0.0002166	0.6	5.3	5.01	-9	18	23	7	3UTR
mmu-miR-214-3p	NM_001310452	Mapk8	ACAGCAGGCACAGACAGGCAGT#GCTGGACCTCTGGGCTGCTGTA#((((((((.(.((((....((((#))))....))))).)))))))).	1309	1331	1	-23	1	4.87E-08	0.4	0.1	0.63	-8	16	22	7	3UTR
mmu-miR-214-3p	NM_001310452	Mapk8	ACAGCAGGCACAGACAGGCAGT#CTGTCCTGGCTACGAGCCTGCTT#..((((((((..((.(((((((.#))).)))).))....))))))).	2173	2196	1	-22	1	0.0001554	0.5	1.7	0.45	-10	16	23	7	3UTR
mmu-miR-214-3p	NM_001310453	Mapk8	ACAGCAGGCACAGACAGGCAGT#CTGCCTGGACTGTGATGTCTGTT#((((((..(((((.((((((((.#)))))))..))))).)).)))).	5432	5455	0.84615	-25	0	0.0002166	0.6	5.5	4.4	-9	18	23	7	3UTR
mmu-miR-214-3p	NM_001310453	Mapk8	ACAGCAGGCACAGACAGGCAGT#GCTGGACCTCTGGGCTGCTGTA#((((((((.(.((((....((((#))))....))))).)))))))).	1448	1470	0.92308	-23	1	4.87E-08	0.4	0.5	-0	-8	16	22	7	CDS
mmu-miR-214-3p	NM_001310453	Mapk8	ACAGCAGGCACAGACAGGCAGT#CTGTCCTGGCTACGAGCCTGCTT#..((((((((..((.(((((((.#))).)))).))....))))))).	2312	2335	1	-22	1	0.0001554	0.5	2.1	0.25	-10	16	23	7	3UTR
mmu-miR-214-3p	NM_001310454	Mapk8	ACAGCAGGCACAGACAGGCAGT#CTGCCTGGACTGTGATGTCTGTT#((((((..(((((.((((((((.#)))))))..))))).)).)))).	5432	5455	0.84615	-25	0	0.0002166	0.6	5.5	4.4	-9	18	23	7	3UTR

mmu-miR-214-3p	NM_001310454	Mapk8	ACAGCAGGCACAGACAGGCAGT#GCTGGACCTCTGGGCTGCTGTA#(((((((.(.((((....((((#))))....))))).))))))).	1448	1470	0.92308	-23	1	4.87E-08	0.4	0.5	-0	-8	16	22	7	CDS
mmu-miR-214-3p	NM_001310454	Mapk8	ACAGCAGGCACAGACAGGCAGT#CTGTCCTGGCTACGAGCCTGCTT#..(((((((..((.(((((((.#))).)))).))....))))))).	2312	2335	1	-22	1	0.0001554	0.5	2.1	0.25	-10	16	23	7	3UTR
mmu-miR-214-3p	NM_001310481	Trps1	ACAGCAGGCACAGACAGGCAGT#TTGCTTGTAGAAAAAGTAATTGTTCCCACTGTGCACTGTTGC#.((((((((((((((((((.#))))))))..................)))))).)))))).	1863	1905	1	-25	1	0.0003906	0.5	0.3	0.22	-4	20	42	8	CDS
mmu-miR-214-3p	NM_001310481	Trps1	ACAGCAGGCACAGACAGGCAGT#CTGTTTTAATCTCACCTGTCTGTGGCATAGCTC#..(((.(.((((((((((....#.)))))))))).)...))).	6285	6318	0.84615	-23	0	0.0005859	0.6	3.6	4.31	-9	14	20	10	3UTR
mmu-miR-214-3p	NM_001310481	Trps1	ACAGCAGGCACAGACAGGCAGT#TGCTTTCTGGCGTGCTGC#.(((((.((.((((.(((((..#)))))))))))).))))).	966	984	0.84615	-23	0	1.08E-05	0.5	1.2	1.6	-7	16	18	11	CDS
mmu-miR-214-3p	NM_001310485	Trps1	ACAGCAGGCACAGACAGGCAGT#CTGTTTTAATCTCACCTGTCTGTGGCATAGCTC#..(((.(.((((((((((....#.)))))))))).)...))).	5718	5751	0.84615	-23	0	0.0005859	0.6	1.6	0.93	-9	14	20	10	3UTR
mmu-miR-214-3p	NM_001310485	Trps1	ACAGCAGGCACAGACAGGCAGT#TGCTTTCTGGCGTGCTGC#.(((((.((.((((.(((((..#)))))))))))).))))).	966	984	0.84615	-23	0	1.08E-05	0.5	1.2	1.6	-7	16	18	11	CDS
mmu-miR-214-3p	NM_001310485	Trps1	ACAGCAGGCACAGACAGGCAGT#GCTGGTAGTCTGGTGGTCATGCTGTC#((((((((.(((((.(.((((#)))).).)))))...))).))))))).	7409	7435	1	-22	1	0.0001868	0.5	2.3	2.09	-5	19	26	6	3UTR
mmu-miR-214-3p	NM_001310530	Chd9	ACAGCAGGCACAGACAGGCAGT#ATTGCCTGCCAATGCCATGCA#...(((((((..(.((((((((#)))))))).)..)))).))).	5588	5609	1	-22	0	0.0069271	0.5	1.9	2.92	-14	16	21	8	CDS
mmu-miR-214-3p	NM_001310530	Chd9	ACAGCAGGCACAGACAGGCAGT#ACTGCAGGGCTGATGGGCATGCCTGCCGG#.(.((((((((((.(..(((((#)))))..).)))).......))))))).	8193	8222	0.84615	-22	0	3.33E-05	0.4	3.8	3.57	-6	17	29	7	CDS
mmu-miR-214-3p	NM_001310530	Chd9	ACAGCAGGCACAGACAGGCAGT#CTGCAGCTGTTTGTGGAAAATCCAAGTGAAGAAGATGCTGC#.(((((..(((((((((((((.#))))..))))))))))....................))))).	2190	2231	0.92308	-22	0	0.0005269	0.6	3.5	3.01	-8	18	41	9	CDS
mmu-miR-214-3p	NM_001310533	Ankrd39	ACAGCAGGCACAGACAGGCAGT#GCTGCCTCCCTCAGCCTGCA#...((((((..((..(((((((#)))))))..))..))))))).	486	506	0.92308	-25	0	3.24E-05	0.4	0.1	-0.1	-12	15	20	7	CDS

mmu-miR-214-3p	NM_001310533	Ankrd39	ACAGCAGGCACAGACAGGCAGT#ACGCCTAGCATGTGACCTGCTGC#.(((((((((((..(((((.((#)))))).)..)))).))))))).	593	616	0.92308	-25	1	7.54E-05	0.4	-0	-0.1	-6	18	23	7	3UTR
mmu-miR-214-3p	NM_001310584	Tmem260	ACAGCAGGCACAGACAGGCAGT#GCTGTCTGTCTCTCCACACACTCCAGTGCCCTGCTGC#.((((((((((((((((((((#)))))))))))..............))))).))))).	5093	5130	0.92308	-31	1	0.0011408	0.4	0.5	0.77	-4	21	37	11	3UTR
mmu-miR-214-3p	NM_001310584	Tmem260	ACAGCAGGCACAGACAGGCAGT#CTGCCTGTCTCGTCCTACCTCAGTCGCGCCCGCTGG#.((((.((((((((((((((.#))))))))))).))..............))).)))).	988	1024	0.92308	-28	0	4.30E-05	0.3	2.6	2.96	-6	19	36	10	CDS
mmu-miR-214-3p	NM_001310584	Tmem260	ACAGCAGGCACAGACAGGCAGT#GCTGCTGAGGCTGACTCTGGCCTTCTCTGCTGGCCTGCTGC#.((((((((.(((.((((((((#)))))...................)))))))))))))).	936	977	0.92308	-27	1	0.0002412	0.4	1.2	2.58	-6	19	41	14	CDS
mmu-miR-214-3p	NM_001310594	Spata13	ACAGCAGGCACAGACAGGCAGT#CCTGCTGTGCACCTTGGCATGGTCACTTATAGGCAGCACTGCTGA#.((((((((((((.((((....#)))))))))))..........................)))))).	3299	3344	0.84615	-22	1	2.55E-05	0.4	-0	-0.3	-5	16	45	10	3UTR
mmu-miR-214-3p	NM_001310594	Spata13	ACAGCAGGCACAGACAGGCAGT#GCAGCAGGTCTTTGCCTTGGCTGA#.((((((((((.((((..((.((#)).))..)))).)))))..)))).	1885	1909	0.84615	-22	1	1.28E-05	0.4	2.6	2.62	-7	17	24	5	CDS
mmu-miR-214-3p	NM_001310594	Spata13	ACAGCAGGCACAGACAGGCAGT#GCGCCGTGTGTGTGTGTGTGTGTG#((((((.(((((.((((((.((#))))).))).))))).))).))).	3418	3442	0.84615	-22	0	0.1052438	0.5	-0	-0.3	-7	19	24	5	3UTR
mmu-miR-214-3p	NM_001310594	Spata13	ACAGCAGGCACAGACAGGCAGT#GCCTGCCGCCTGCTC#..(((((((...(.(((((...#)))))).))))))).	1091	1106	0.92308	-22	1	2.11E-06	0.4	3.8	3.47	-13	13	15	7	CDS
mmu-miR-214-3p	NM_001310632	Gm21761	ACAGCAGGCACAGACAGGCAGT#GTGTGTTTGGAGCCCATGAACCCTGATGCCTGCTA#..((((((((((((((.((...#)).)))))).................))))))))).	698	733	0.84615	-22	1	0.0039594	0.5	-0	-0.1	-5	16	35	8	3UTR
mmu-miR-214-3p	NM_001310632	Gm21761	ACAGCAGGCACAGACAGGCAGT#TTGCAATTAGGGAATCTTCTGTCAGCTCCTGCTGTT#((((((((..(.((((((....#.)))))).)..))))	737	773	0.92308	-21	1	4.43E-05	0.5	-0	0.02	-10	15	20	8	3UTR

mmu-miR-214-3p	NM_001310637	Pdcl2	ACAGCAGGCACAGACAGGCAGT#CTGTGGGTTGTAATTCATCTCTATAGATCAAGTGTCCCAATGTGCTTGGTGG#.((.((((((((....((((.(#.).))))....)))))))).)).	347	399	0.92308	-20	0	3.83E-05	0.6	1	1.51	-10	15	23	8	CDS
mmu-miR-214-3p	NM_001310637	Pdcl2	ACAGCAGGCACAGACAGGCAGT#TGCCTTATTTCCTGCTGA#.(((((((.......(((((..#))))).....))))))).	778	796	0.84615	-19	1	0.0069884	0.6	2.6	2.67	-15	12	18	7	3UTR
mmu-miR-214-3p	NM_001310646	Tsc22d1	ACAGCAGGCACAGACAGGCAGT#ACTGGCTGCTGCTGCTGCTGTC#((((((.((((((.(((.((((#)))).)))))).))))))))).	3930	3952	0.84615	-26	0	2.61E-05	0.5	0.6	1.37	-6	19	22	9	3UTR
mmu-miR-214-3p	NM_001310647	Tsc22d1	ACAGCAGGCACAGACAGGCAGT#ACTGGCTGCTGCTGCTGCTGTC#((((((.((((((.(((.((((#)))).)))))).))))))))).	394	416	0.84615	-26	0	2.61E-05	0.5	0.6	1.37	-6	19	22	9	3UTR
mmu-miR-214-3p	NM_001310753	Dapp1	ACAGCAGGCACAGACAGGCAGT#ACAAATCAAAAGTAGATCGTGGTTGGGAGCTGTCTGCTGTCTGCTGA#.((((((..((((.((((((((#.))))))))))))))))))).	941	988	0.92308	-26	1	1.82E-06	0.5	0.8	0.15	-8	18	20	18	3UTR
mmu-miR-214-3p	NM_001310753	Dapp1	ACAGCAGGCACAGACAGGCAGT#CTGCCTGTAACTCCAGGTGCGCGCA#...((..((((..((((((((.#))))))))........))))..)).	1426	1451	0.84615	-22	0	0.0002322	0.4	0.1	-0	-15	14	25	8	3UTR
mmu-miR-214-3p	NM_001310841	Cadm1	ACAGCAGGCACAGACAGGCAGT#ACTGTGACCCAGACGCTGTCGTCTGCTGTG#((((((((((...((((((((((#)))))..........)))))))))))	2377	2407	0.84615	-23	1	0.0002798	0.5	1.8	1.85	-11	19	30	14	3UTR
mmu-miR-214-3p	NM_001311093	Tmem209	ACAGCAGGCACAGACAGGCAGT#GCCTGTGTGCTGGCGCCTGTGTA#(((.((((((.(((((((((...#))))))...)))..)))))))).	2468	2491	0.84615	-24	0	8.28E-06	0.4	4.1	3.95	-8	17	23	8	3UTR
mmu-miR-214-3p	NM_001311094	Rassf5	ACAGCAGGCACAGACAGGCAGT#GCTGCCTGAGGTCTCATGCCCAGCCTTACACCTCTCTCCCTTAAGAAGCCCCCGTCCTGCTGTG#((((((((.(((((((((((((#)))))))).......................)))..................)))))))))).	2617	2681	0.84615	-24	1	0.0119349	0.5	3.4	2.36	-5	21	64	10	3UTR
mmu-miR-214-3p	NM_001311143	Xpo6	ACAGCAGGCACAGACAGGCAGT#AGTGCCTGCTTGCCTTCCTGGCTGCTCACTGCCCCAGGGATGTGCCTGCTGC#.(((((((((((..(.((((((#.))))))....)..))))))))))).	3979	4031	0.92308	-32	1	1.05E-05	0.4	1	1.62	-6	18	26	11	3UTR
mmu-miR-214-3p	NM_001311790	Ctsc	ACAGCAGGCACAGACAGGCAGT#ATTGCCTGTTTGTATTCCAGTCTGCTT#..((((((((((((((((((((#)))))))))))))......))))))).	3122	3149	0.92308	-28	1	0.0270844	0.6	0	0.02	-5	20	27	13	3UTR

mmu-miR-214-3p	NM_001313712	Sp100	ACAGCAGGCACAGACAGGCAGT#TGCCCTGTGACCTTCTGC#.(((.((((((((...((((..#)))))))).)))).))).	1553	1571	1	-19	0	0.0002651	0.5	-0	0.13	-10	15	18	9	CDS
mmu-miR-214-3p	NM_001313712	Sp100	ACAGCAGGCACAGACAGGCAGT#CCTGTTTTGTGCAACTGG#.(((...(((((((((((....#))))))).))))..))).	814	832	0.84615	-18	0	0.0044501	0.7	1.1	0.19	-11	14	18	7	CDS
mmu-miR-214-3p	NM_001313713	Sp100	ACAGCAGGCACAGACAGGCAGT#TGCCCTGTGACCTTCTGC#.(((.((((((((...((((..#)))))))).)))).))).	1499	1517	1	-19	0	0.0002651	0.5	-0	0.13	-10	15	18	9	CDS
mmu-miR-214-3p	NM_001313713	Sp100	ACAGCAGGCACAGACAGGCAGT#CCTGTTTTGTGCAACTGG#.(((...(((((((((((....#))))))).))))..))).	814	832	0.84615	-18	0	0.0044501	0.7	1.1	0.19	-11	14	18	7	CDS
mmu-miR-214-3p	NM_001313714	Sp100	ACAGCAGGCACAGACAGGCAGT#GCTGTCTCCCTGTTGCCTGCTGA#.((((((((((((..(((((((#)))))))..)))).)))))))).	1850	1873	1	-31	1	0.0084043	0.5	0.1	0.08	-5	19	23	8	3UTR
mmu-miR-214-3p	NM_001313737	Asb11	ACAGCAGGCACAGACAGGCAGT#GCTGCGTGTGTCAATGTGCTGTTGG#.(((((.(((((((((.(((((#)))))...))))..)))))))))).	480	505	1	-23	0	0.0097838	0.4	2	2.28	-5	19	25	10	CDS
mmu-miR-214-3p	NM_001313737	Asb11	ACAGCAGGCACAGACAGGCAGT#ACATGTAGTTTATTGCCTGCTGTA#((((((((((.((((..(((((#)).))).))))..)))))))))).	1029	1053	1	-22	1	3.13E-05	0.6	0.6	0.92	-7	19	24	10	3UTR
mmu-miR-214-3p	NM_001313757	Acvr2b	ACAGCAGGCACAGACAGGCAGT#CTGCCCTGGGCTGTCCTGCTGTC#(((((((.((((.(((((((.#))))).))..))))))))))).	8843	8866	1	-29	1	0.0001143	0.3	0.2	0.05	-6	19	23	12	3UTR
mmu-miR-214-3p	NM_001313757	Acvr2b	ACAGCAGGCACAGACAGGCAGT#GCCTGTCCCTGCATCCTGCCTGCTGG#.(((((((((((((((((...#)))))))..)).....))))))))).	7774	7800	1	-30	1	0.0002526	0.4	0.1	-0.1	-4	18	26	9	3UTR
mmu-miR-214-3p	NM_001313763	Vgll1	ACAGCAGGCACAGACAGGCAGT#ACCGTCTCTTTCTGTCCCTGCTGC#.(((((((.(((((.((((.((#)).))))...))))).))))))).	1295	1319	1	-23	1	0.0081269	0.5	0.2	0.22	-6	18	24	7	3UTR
mmu-miR-214-3p	NM_001313763	Vgll1	ACAGCAGGCACAGACAGGCAGT#CTGCCTCCCTACCCTGTCCACTACAAGAAGAAAATCTGTGCTCGTGGAGCTGC#.((((.((((((((.((((((.#)))))).........................))))))))......)))).	1083	1136	0.84615	-21	0	6.97E-06	0.5	0	0.32	-6	18	53	8	CDS
mmu-miR-214-3p	NM_001316691	Mapkapk3	ACAGCAGGCACAGACAGGCAGT#GGATGTGTCCTGCTGTG#((((((((((((..(......#)..)))).)))))))).	1324	1341	0.92308	-22	1	1.02E-05	0.5	4	3.28	-6	13	17	8	3UTR

mmu-miR-214-3p	NM_001316691	Mapkapk3	ACAGCAGGCACAGACAGGCAGT#GCTGCAGAGTCCTGATTGCTTGCTT#..(((((((((((((..(((((#)))))...))).))..)))))))).	1532	1557	0.80769	-21	1	0.0066708	0.4	4.5	4.38	-5	18	25	8	3UTR
mmu-miR-214-3p	NM_001316691	Mapkapk3	ACAGCAGGCACAGACAGGCAGT#CTGGCTGGTGAAGGGCTGCTGA#.((((((.(.....(((.(((.#))).)))......).))))))).	1946	1968	0.92308	-21	1	3.21E-05	0.4	3.2	2.99	-16	13	22	6	3UTR
mmu-miR-214-3p	NM_001317242	Mtch2	ACAGCAGGCACAGACAGGCAGT#ACCAGAGGGCAGCAGAACTTTGGTTGTGCCTGTGCATCTGGCCTGCTGA#.((((((((.((((((((((..#.))))))....))))))))))))).	2533	2582	0.92308	-31	1	0.0005011	0.5	1.4	-0	-5	18	24	12	3UTR
mmu-miR-214-3p	NM_001317243	Mtch2	ACAGCAGGCACAGACAGGCAGT#ACCAGAGGGCAGCAGAACTTTGGTTGTGCCTGTGCATCTGGCCTGCTGA#.((((((((.((((((((((..#.))))))....))))))))))))).	2479	2528	0.92308	-31	1	0.0005011	0.5	1.4	-0	-5	18	24	12	3UTR
mmu-miR-214-3p	NM_001317722	Col11a2	ACAGCAGGCACAGACAGGCAGT#GCCTGTCTGCTTCATGGGCTAGGCCTGTC#...((((((.(((((((((...#)))))))))............)))))).	5643	5672	0.84615	-25	0	2.10E-05	0.4	3.9	1.44	-8	15	29	9	CDS
mmu-miR-214-3p	NM_001317722	Col11a2	ACAGCAGGCACAGACAGGCAGT#GCCTCCTGGCCTGCCTGG#.((((((((.(((..((((...#)))).))))))))).)).	4830	4848	0.92308	-23	1	6.20E-06	0.3	3.8	3.1	-7	15	18	9	CDS
mmu-miR-214-3p	NM_001327821	Creb5	ACAGCAGGCACAGACAGGCAGT#ACAGCCTGTGGGTGACCGATAGTGTGTGTGCTGTT#((((((.(((((.((((((.((#)).))))))).............))))).)))))).	4909	4944	0.84615	-24	0	0.000375	0.5	0.5	0.04	-7	19	35	6	3UTR
mmu-miR-214-3p	NM_001327821	Creb5	ACAGCAGGCACAGACAGGCAGT#TTGTTTGTCTTTGTAAACAGCAAAGCCTTTGTGTTTGTGTGTCTGTCTGTA#((((((((((((((((((((.#)))))))))))).........................))))))	7534	7585	0.92308	-23	1	0.0225899	0.6	2	1.95	-4	21	51	10	3UTR
mmu-miR-214-3p	NM_001329627	Clgn	ACAGCAGGCACAGACAGGCAGT#CTGGCTGTGGATGTGCCGTGGCTGA#.((((.((((((.((((.(((.#))).))))...))))))...)))).	3741	3766	0.92308	-25	0	1.92E-05	0.5	0.3	-0.1	-7	17	25	6	3UTR
mmu-miR-214-3p	NM_001329627	Clgn	ACAGCAGGCACAGACAGGCAGT#GCTGTAGGTTTGCTCTGTTGTA#((((((((..(((((..(((((#)))))..))))).)))))))).	2373	2395	0.84615	-20	1	0.0016458	0.6	1.3	0.58	-6	18	22	8	3UTR
mmu-miR-214-3p	NM_001330198	Fuca2	ACAGCAGGCACAGACAGGCAGT#CTGCCGCTGCTGCTGCTGC#.(((((.((((((.(.(((((.#))))))))).)))))))).	164	183	0.84615	-23	0	3.91E-05	0.3	1.1	0	-6	17	19	9	CDS

mmu-miR-214-3p	NM_001331052	Hdgf	ACAGCAGGCACAGACAGGCAGT#CTGCCCCGTTCCTGCTGCTGTCTGGGTGCTACTGGGGAAACTGGCCATGGCCTGCA#...((((((.((((((.(((((#.))))))))))))...........................)))))).	967	1023	0.84615	-24	0	0.0015584	0.4	2.1	2.13	-8	17	44	11	3UTR
mmu-miR-214-3p	NM_001331052	Hdgf	ACAGCAGGCACAGACAGGCAGT#CTGCTGGGGTCTAGGCTGCTGTT#(((((((.(..((((.(((((.#)))))...)))).).))))))).	1194	1217	0.92308	-23	1	7.02E-05	0.4	-0	0.11	-11	17	23	7	3UTR
mmu-miR-214-3p	NM_001346552	Cyb5rl	ACAGCAGGCACAGACAGGCAGT#ACTTCCCTTCCTGTCTGCTGCGTCCTGCTGTA#((((((((.(((.(((((((.#.)))))))))).).)))))))).	1140	1172	0.92308	-31	1	0.0003768	0.4	0.2	-0.2	-6	19	23	10	3UTR
mmu-miR-214-3p	NM_001346552	Cyb5rl	ACAGCAGGCACAGACAGGCAGT#GCTGTCTGCTGGCCTGACTGA#.((((((((.(((.((((((((#)))))))))))))))).))).	971	992	1	-29	1	1.54E-05	0.4	0.6	1.38	-6	19	21	16	CDS
mmu-miR-214-3p	NM_001346655	Zzz3	ACAGCAGGCACAGACAGGCAGT#GCTGCTTGCCTGCATGTCTGCTGC#.(((((((((((.((((((((#)))))))).)))..))))))))).	5550	5574	0.92308	-30	1	2.88E-07	0.4	0.1	0.25	-5	20	24	9	3UTR
mmu-miR-214-3p	NM_001346655	Zzz3	ACAGCAGGCACAGACAGGCAGT#GCTGCCAGTGTGTGCTGTGTGTTGG#.(((((((((((.((.((((((#)))))).)).))))))...))))).	5598	5623	0.84615	-27	1	0.0003369	0.5	0.6	0.17	-5	19	25	6	3UTR
mmu-miR-214-3p	NM_001346760	Clint1	ACAGCAGGCACAGACAGGCAGT#ACAGTCGGAGAAGGAAGACTCGTGTGTTGTCTGTCTGTAGCGGTTGTT#(((((..((((((((((((((.#.))))))))))))).)).))))).	2804	2852	1	-26	0	0.0014846	0.5	0.4	1.15	-7	19	23	12	3UTR
mmu-miR-214-3p	NM_001346760	Clint1	ACAGCAGGCACAGACAGGCAGT#ACTGGATGTGTGTGCTTTTGCCAAGACTCCAGCAGTGCTGC#.(((((((((((.(((..((((#)))).))).))))))).................))))).	2621	2662	0.92308	-22	1	0.0002479	0.5	2.6	2.31	-5	18	41	6	3UTR
mmu-miR-214-3p	NM_001347055	Virma	ACAGCAGGCACAGACAGGCAGT#GCTGATGACTGCAATCTGCGACTGTCTGCTGG#.((((((((((((...(((((#.)))))..))))....))))))	4044	4076	1	-25	1	7.47E-05	0.5	2.5	2.65	-5	18	26	9	CDS
mmu-miR-214-3p	NM_001347055	Virma	ACAGCAGGCACAGACAGGCAGT#TTGCCTTGTGGCTGCA#...((((.((((...((((((.#))))))))))).)))).	2351	2367	1	-21	0	1.58E-05	0.5	3.4	3.06	-12	14	16	10	CDS
mmu-miR-214-3p	NM_001347083	Eif4e2	ACAGCAGGCACAGACAGGCAGT#TGTGTGTGTGTGTCTGTGTGTG#((((((((((((.(((.(((..#))).))).))))))))).))).	2483	2505	1	-25	1	0.9117894	0.5	0.1	-0	-5	18	22	9	3UTR

mmu-miR-214-3p	NM_001347087	Tmem209	ACAGCAGGCACAGACAGGCAGT#GCCTGTGTGCTGGCGCCTGTGTA#(((.(((((.((((((((...#))))))...)))..)))))))).	2281	2304	0.84615	-24	0	8.28E-06	0.4	3.2	3.92	-8	17	23	8	3UTR
mmu-miR-214-3p	NM_001347127	Fam155a	ACAGCAGGCACAGACAGGCAGT#CTGTCCCTAAGCAGACAACATCCGCTTGCTTCTGTCTGTGTCTCGCATGG#.(((((((((((((((((.(((#.)))))))))))))))).)).)).	2228	2278	1	-26	1	0.0018955	0.4	4.2	3.66	-4	20	24	16	3UTR
mmu-miR-214-3p	NM_001347127	Fam155a	ACAGCAGGCACAGACAGGCAGT#GGTGCTTGGATGTGTCGGCAGTA#((.((.((((((..((((((.(#).))))))..)))))).)).)).	148	171	0.84615	-22	0	0.0005245	0.5	1.6	2.03	-10	17	23	6	CDS
mmu-miR-214-3p	NM_001347159	Acot11	ACAGCAGGCACAGACAGGCAGT#GCTGGCTGTCCCTGCG#...(((((....(((((.((((#)))).)))))))))).	327	343	0.92308	-22	0	6.28E-06	0.4	-0	0.08	-15	14	16	10	CDS
mmu-miR-214-3p	NM_001347159	Acot11	ACAGCAGGCACAGACAGGCAGT#GCTTCTGTCTGTGGCGTGAGGG#.(..((.(((((((((((.(((#))))))))))))))).).))..).	1660	1682	0.92308	-22	0	1.03E-06	0.5	0.1	0.87	-10	17	22	13	CDS
mmu-miR-214-3p	NM_001347159	Acot11	ACAGCAGGCACAGACAGGCAGT#TGTGTGTGTGTGTGTGTGTGTG#((((((.((((((.(((.(((..#))).))).))))).))).))).	3669	3691	0.84615	-22	0	0.6368158	0.5	-0	0.44	-7	17	22	5	3UTR
mmu-miR-214-3p	NM_001347164	Atp6v0e2	ACAGCAGGCACAGACAGGCAGT#GCTGGTAGCTACTGCTGTCTGCTGTTACCTCTTATGCCCTGCTGTG#((((((((((((.((((((((#.))))))))))))..........))).))))))).	301	347	1	-28	1	0.0190031	0.4	2.6	1.71	-5	21	34	12	CDS
mmu-miR-214-3p	NM_001347210	Bpnt1	ACAGCAGGCACAGACAGGCAGT#ACTGTCCAAGTCATTTGTGCAGTCAACATTCCTGCTGA#.(((((((((((((.((((((#))))))...)))...)))))...........)))))).	1924	1962	0.84615	-22	1	0.0025219	0.5	0.5	0.35	-4	20	38	6	3UTR
mmu-miR-214-3p	NM_001347212	Bpnt1	ACAGCAGGCACAGACAGGCAGT#ACTGTCCAAGTCATTTGTGCAGTCAACATTCCTGCTGA#.(((((((((((((.((((((#))))))...)))...)))))...........)))))).	1752	1790	0.84615	-22	1	0.0025219	0.5	0.5	0.35	-4	20	38	6	3UTR
mmu-miR-214-3p	NM_001347239	Naa50	ACAGCAGGCACAGACAGGCAGT#GCTTCCACTCTGTGCCTACAATGTTGA#.(((((((((((((..((.(((#))).))..)))))))))).....)))).	1975	2002	0.84615	-22	1	0.0282815	0.6	0.7	0.95	-5	18	27	9	3UTR
mmu-miR-214-3p	NM_001347239	Naa50	ACAGCAGGCACAGACAGGCAGT#CTGTGGTACTTTGAGTTATACAGACTGTCTGTGGGGTGCTA#......(((((..(((((((((#.)))))))))...))))).	1253	1294	1	-21	0	0.0001437	0.5	1.3	1.06	-20	14	19	9	3UTR

mmu-miR-214-3p	NM_001347239	Naa50	ACAGCAGGCACAGACAGGCAGT#AGTGATCTGTCTGTAACTGTC#...((((..((((((((((((.(#).)).)))))))))..)))).	3657	3678	1	-20	0	0.0056227	0.7	1.1	1.3	-10	16	21	9	3UTR
mmu-miR-214-3p	NM_001347313	Tet3	ACAGCAGGCACAGACAGGCAGT#GCCTTGTTTTTGTTGTCCCTGCCTGCTGTG#((((((((((..(((((((...#)))).........)))..)))))))))).	9051	9081	0.92308	-27	1	0.0195214	0.5	2.3	1.22	-10	17	30	10	3UTR
mmu-miR-214-3p	NM_001347313	Tet3	ACAGCAGGCACAGACAGGCAGT#TGCCTGTGGATCTGTTCAGCCTGCTC#..(((((((((((((((((..#)))))))....)))))...))))))).	10762	####	0.92308	-27	1	0.0256242	0.5	1.9	2.41	-5	18	26	7	3UTR
mmu-miR-214-3p	NM_001347313	Tet3	ACAGCAGGCACAGACAGGCAGT#CTGCTGTGCCTGGTGC#.((.(((((((((.(((.....#)))))))))))).)).	3265	3281	1	-25	0	8.00E-06	0.4	1.3	1.12	-8	14	16	12	CDS
mmu-miR-214-3p	NM_001347341	Ephx3	ACAGCAGGCACAGACAGGCAGT#AGTGTGTGTGTGTGTGTGTGTGTG#((((((.(((((.(((.(((.(#).))).))).))))).))).))).	2189	2213	1	-22	0	0.7081606	0.5	1.7	0.47	-7	18	24	5	3UTR
mmu-miR-214-3p	NM_001347341	Ephx3	ACAGCAGGCACAGACAGGCAGT#TGTGTGTGTGTGTGTGTGTGTG#((((((.((((((.(((.(((..#))).))).))))).))).))).	1859	1881	0.92308	-22	0	0.2209187	0.5	2.1	1.44	-7	17	22	5	3UTR
mmu-miR-214-3p	NM_001347395	Ttll5	ACAGCAGGCACAGACAGGCAGT#CTGCCCTCCCCCTGCCCTGCTAGGTCCCTCTGTCTCTCCTCCTGCTGC#.(((((((...((((((((((.#.)))))).............)))))......)))))))).	4431	4479	0.84615	-23	1	0.0260191	0.4	0.2	0.74	-9	17	38	7	3UTR
mmu-miR-214-3p	NM_001347395	Ttll5	ACAGCAGGCACAGACAGGCAGT#CCTGCTGTGCGCTCCTGG#.(((.((((((((.((((....#))))))))))).)).))).	4178	4196	1	-22	0	0.0001385	0.4	-1	0.35	-9	15	18	10	3UTR
mmu-miR-214-3p	NM_001347411	Dlgap1	ACAGCAGGCACAGACAGGCAGT#CTGCTCTGGGACTTGCTGAATGTCACCTGAAGGCCCCTGTGTCTGCTT#..(((((((((((...(((...#.))).))))))))))).	891	939	1	-24	1	0.0007864	0.5	0.3	0.53	-7	14	17	11	5UTR
mmu-miR-214-3p	NM_001347411	Dlgap1	ACAGCAGGCACAGACAGGCAGT#CTGCATCTGTGCCCTCCCTCGCAGCTGTA#(((((.((((((((...((((.#)))).)))))))).........))))).	4517	4546	1	-23	0	0.0030793	0.4	0.3	1	-7	17	29	8	3UTR
mmu-miR-214-3p	NM_001347412	Dlgap1	ACAGCAGGCACAGACAGGCAGT#CTGCTCTGGGACTTGCTGAATGTCACCTGAAGGCCCCTGTGTCTGCTT#..(((((((((((...(((...#.))).))))))))))).	891	939	1	-24	1	0.0007864	0.5	0.3	0.53	-7	14	17	11	5UTR

mmu-miR-214-3p	NM_001347412	Dlgap1	ACAGCAGGCACAGACAGGCAGT#CTGCATCTGTGCCCTCCCTCGCAGCTGTA#(((((.((((((((...((((.#)))).))))))))..........))))).	4571	4600	1	-23	0	0.0030793	0.4	0.3	1	-7	17	29	8	3UTR
mmu-miR-214-3p	NM_001347413	Dlgap1	ACAGCAGGCACAGACAGGCAGT#CTGCATCTGTGCCCTCCCTCGCAGCTGTA#(((((.((((((((...((((.#)))).))))))))..........))))).	2950	2979	1	-23	0	0.0030793	0.4	0.3	1	-7	17	29	8	3UTR
mmu-miR-214-3p	NM_001347414	Dlgap1	ACAGCAGGCACAGACAGGCAGT#CTGCATCTGTGCCCTCCCTCGCAGCTGTA#(((((.((((((((...((((.#)))).))))))))..........))))).	2872	2901	1	-23	0	0.0030793	0.4	0.3	1	-7	17	29	8	3UTR
mmu-miR-214-3p	NM_001347422	Peli3	ACAGCAGGCACAGACAGGCAGT#GCTGGCCATCTGTACCTGCTGG#.(((((((.(((((..(.((((#)))).)..))))).))))))).	2680	2702	0.92308	-27	1	0.0028375	0.5	3.3	3.98	-6	17	22	7	3UTR
mmu-miR-214-3p	NM_001347422	Peli3	ACAGCAGGCACAGACAGGCAGT#CTCCTGCTGTTCAGAGGGAGCTCTGCATGTGGGACTCTGCCTGCTGG#.(((((((((.(((((.((((.#.)))).)))....)).))))))))).	1427	1474	1	-26	1	5.58E-07	0.4	-0	-0.3	-7	18	26	9	3UTR
mmu-miR-214-3p	NM_001347433	Slc6a12	ACAGCAGGCACAGACAGGCAGT#GCTAACAAGCCTGCATGGATCACCTGCTGTG#((((((((..((..(((((...#.)))))..)).....)))))))).	2260	2291	0.92308	-24	1	0.0004582	0.5	0	0	-8	15	24	8	3UTR
mmu-miR-214-3p	NM_001347433	Slc6a12	ACAGCAGGCACAGACAGGCAGT#GCTGGCCTTGTGCCTCCTGC#.(((.(((((((...(((((((#))))).)))))))))).))).	681	701	1	-22	0	0.0014479	0.4	2.9	2.55	-11	17	20	10	CDS
mmu-miR-214-3p	NM_001347462	Rasgef1c	ACAGCAGGCACAGACAGGCAGT#CTTGTTGAGTGCCATGTGCTGTG#(((((((((((.((((((....#))))))..)))))...))))))).	2236	2259	1	-22	1	0.0003247	0.5	-0	-0	-7	17	23	6	3UTR
mmu-miR-214-3p	NM_001347596	Dock9	ACAGCAGGCACAGACAGGCAGT#TGAACCCGGGCAGTATCGTGAAGGACGAAGCCCTGGCTGTGCCTGCTGG#.(((((((((((((.((((....#.)))).)))))))))))))).	3657	3706	1	-32	1	4.96E-05	0.4	0	-0.1	-5	16	19	12	CDS
mmu-miR-214-3p	NM_001347656	Cdipt	ACAGCAGGCACAGACAGGCAGT#ACTAGCCTGGCATGTGGTAGCTGC#.((((.(.((((..((((((((#))).)))))...)))).).)))).	1736	1760	0.84615	-22	0	0.0001282	0.6	0.2	0.28	-10	17	24	5	3UTR
mmu-miR-214-3p	NM_001355173	Kcnab2	ACAGCAGGCACAGACAGGCAGT#ACTGCCTGCCTGTGAACCACACCTCAGAGCTAAGCTCCTGCTGG#.((((((((((((.((((((((#)))))))).)))))......................))))))).	2980	3024	0.81538	-32	1	2.06E-06	0.5	3.3	3.88	-5	20	44	8	3UTR

mmu-miR-214-3p	NM_001355193	Ctnna2	ACAGCAGGCACAGACAGGCAGT#GCTGACTTGTCTGCCTCTGG#.(((.((((..((((((((((((#)))).))))))))))))).	3057	3077	1	-23	0	0.0017291	0.6	3.6	4.16	-12	18	20	14	3UTR
mmu-miR-214-3p	NM_001355431	Slc23a2	ACAGCAGGCACAGACAGGCAGT#ACTGATAGAAGTGGTCATTGGCCTCCTTGGCCTGCCTGGGGCTCTGCTGA#.((((((((.(((.(((((...#.))))).)))..)).)))))).	817	867	0.92308	-27	1	7.56E-05	0.4	4.7	3.38	-6	16	22	6	CDS
mmu-miR-214-3p	NM_001355494	Ube2j1	ACAGCAGGCACAGACAGGCAGT#CTGCCTGTTTTGCTTGTA#...(((((((.((((((((((.#))))))))))))))))).	3286	3304	1	-26	0	0.0003137	0.5	0.2	0.86	-9	17	18	17	3UTR
mmu-miR-214-3p	NM_001355494	Ube2j1	ACAGCAGGCACAGACAGGCAGT#ACTCCTGTCATGTGATGTTGTG#((((((..((((((((((.(((#))))))))).)))).))))))).	2392	2414	0.84615	-23	0	0.0006187	0.6	0.4	0.69	-8	19	22	9	3UTR
mmu-miR-214-3p	NM_001355494	Ube2j1	ACAGCAGGCACAGACAGGCAGT#TGCTGGTTTGTGACTTAAGAGCTGTG#((((((((((((((.((((..#)))).))))))).)))....))))).	1043	1069	1	-21	1	7.94E-05	0.6	1.6	3.1	-4	19	26	7	CDS
mmu-miR-214-3p	NM_001355494	Ube2j1	ACAGCAGGCACAGACAGGCAGT#CTGCCATGAAGGATGTCCTGTTGC#.((((((.(((..(((((((.#))))).)).....)))))))))).	666	690	0.92308	-21	1	0.0018185	0.5	1.8	3.19	-7	17	24	10	CDS
mmu-miR-214-3p	NM_001355693	Phf24	ACAGCAGGCACAGACAGGCAGT#GCTGGCTGTTTGGCTAGTCCGAAGGCTGAGGCTGCTGC#.((((((((.(((((((.((((#)))).))))))))).................)))))).	32	70	0.92308	-25	1	4.36E-05	0.5	0.3	0.39	-5	19	38	9	5UTR
mmu-miR-214-3p	NM_001355693	Phf24	ACAGCAGGCACAGACAGGCAGT#GCCTGGCTGTGTCAGCC#...((.(((((((.((((((...#))))).))))))).)).	280	297	0.84615	-24	0	4.39E-07	0.4	4	3.37	-9	14	17	7	CDS
mmu-miR-214-3p	NM_001355695	Phf24	ACAGCAGGCACAGACAGGCAGT#GCCTGTCGTGCCGCC#...((.(((((.(((((((...#)))))))))))))).	36	51	0.84615	-26	0	0.0012588	0.2	-0	0.18	-12	14	15	14	5UTR
mmu-miR-214-3p	NM_001355695	Phf24	ACAGCAGGCACAGACAGGCAGT#GCACATGTGGTGCCTGTCAAAGCTCCCTTCAAGGGACCTGCTGTA#((((((((..(.(((((((((.(#.).))))))))).............)..)))))))).	5958	6003	0.84615	-26	1	1.26E-05	0.4	-0	0.21	-10	18	38	8	3UTR
mmu-miR-214-3p	NM_001355724	Pramel12	ACAGCAGGCACAGACAGGCAGT#ACTGCCTGGGCCTGCTGG#.((((((((.(...((((((((#)))))))))))))))))).	615	633	0.84615	-29	1	1.99E-05	0.5	0.3	0.03	-11	17	18	17	5UTR

mmu-miR-214-3p	NM_001355724	Pramel12	ACAGCAGGCACAGACAGGCAGT#ACAGCCAGTCTGAGCCAGCTGA#.((((.(((.(((((.(((.((#)).))).))))).))).)))).	1901	1923	0.84615	-25	0	0.0026022	0.4	-1	-0.1	-7	17	22	5	CDS
mmu-miR-214-3p	NM_001356265	Zfand3	ACAGCAGGCACAGACAGGCAGT#GCTGCCCACGTCTGGCTGCTGA#.(((((.((.(((((.((((((#))))))...)))))))))))).	2042	2064	0.84615	-27	0	1.37E-05	0.4	0.9	0.27	-7	18	22	12	3UTR
mmu-miR-214-3p	NM_001356265	Zfand3	ACAGCAGGCACAGACAGGCAGT#CTGGCTGGTTACTAAGGTGCCTGCTA#..(((((((((...(((.(((.#))).))).........))))))))).	1063	1089	0.84615	-24	1	0.0002362	0.7	3.6	3.31	-10	15	26	9	3UTR
mmu-miR-214-3p	NM_001356265	Zfand3	ACAGCAGGCACAGACAGGCAGT#ACATGCCTGTCACTGCTGG#.((((((.....((((((((((#)).)))))))).)))))).	1155	1174	0.92308	-23	1	0.0008343	0.5	2.5	2.96	-15	16	19	8	3UTR
mmu-miR-214-3p	NM_001356301	Tspan9	ACAGCAGGCACAGACAGGCAGT#GTCATGTCTCCTGCCTGCTC#..((((((((.((((((((...#))).)))))..)))))))).	860	880	0.92308	-24	1	0.0626299	0.3	-0	-0.3	-8	16	20	8	3UTR
mmu-miR-214-3p	NM_001356314	Stx8	ACAGCAGGCACAGACAGGCAGT#CTGCCTGCCTCTGCCTCCCAAGTGCTGG#.(((((((((.((.(((((((.#))))))).)).)))))).......)))).	1108	1136	1	-26	1	3.26E-05	0.5	0.2	0.06	-7	18	28	7	3UTR
mmu-miR-214-3p	NM_001356323	Errfi1	ACAGCAGGCACAGACAGGCAGT#GCTGCCTGTGGTATGGCCTCTGC#.(((.((((....(((((((((#)))))))))......))))))).	1661	1684	0.84615	-25	0	3.26E-07	0.5	1.2	0.71	-17	16	23	9	CDS
mmu-miR-214-3p	NM_001356323	Errfi1	ACAGCAGGCACAGACAGGCAGT#ACGAGCTGGTCTTGTGTTGCTGG#.(((((.((((((((.(((.((#))..))).)))).))))))))).	2657	2680	1	-21	0	0.0001184	0.6	3.3	1.22	-6	18	23	9	3UTR
mmu-miR-214-3p	NM_001356323	Errfi1	ACAGCAGGCACAGACAGGCAGT#ACTGTTGGATGTGCTGCTC#..((((.(((((..((.(((((#)))))))..))))))))).	575	594	0.84615	-20	0	7.27E-05	0.4	-1	0.69	-9	16	19	9	CDS
mmu-miR-214-3p	NM_001356329	Qtrt2	ACAGCAGGCACAGACAGGCAGT#ACTGTCCTGCTGTGTCTGTA#...((((((((((.((((((((#)))).)))))))))))))).	2020	2040	1	-28	0	0.0002148	0.5	0	0	-7	18	20	14	3UTR
mmu-miR-214-3p	NM_001356354	Tmem268	ACAGCAGGCACAGACAGGCAGT#TGCAGCTGTGCCTGTTGA#.((((((((((((.(..(((..#))).))))))))))))).	210	228	1	-26	1	0.0001766	0.5	0.2	-0	-5	16	18	13	5UTR
mmu-miR-214-3p	NM_001356354	Tmem268	ACAGCAGGCACAGACAGGCAGT#GCTGTCTGTCTGCCATGGCTTGTG#...((((((.((((((((((((#))))))))))))).....)))))).	348	372	1	-25	0	3.64E-06	0.4	-0	0.51	-8	18	24	12	5UTR

mmu-miR-214-3p	NM_001356381	Dvl1	ACAGCAGGCACAGACAGGCAGT#GCTGAGTCTGTGCTCCTGCTGTG#(((((((((((((((...((((#)))).))))))))..))))))).	2491	2514	0.84615	-28	1	0.0006406	0.3	-0	1.04	-4	19	23	8	3UTR
mmu-miR-214-3p	NM_001356470	Plch2	ACAGCAGGCACAGACAGGCAGT#GCTGCCGCTGTGTGGAGGTGGACTGCTGG#.((((((((((((.(.((((((#))))))))))))).........))))	1261	1290	0.84615	-26	1	0.0021455	0.3	-1	0.99	-5	19	29	13	CDS
mmu-miR-214-3p	NM_001356470	Plch2	ACAGCAGGCACAGACAGGCAGT#GCTGCCCGTCCTGACCTGCCTCCTGTG#((((.((((((((((.((((((#)))))).))).))...))))).)))).	4293	4320	0.84615	-26	0	0.0287406	0.4	3.4	4	-8	20	27	6	3UTR
mmu-miR-214-3p	NM_001356589	Crtac1	ACAGCAGGCACAGACAGGCAGT#TGTATCCTGTGCCTGCTGA#.((((((((((((....(((..#)))...)))))))))))).	2375	2394	1	-26	1	0.0129309	0.5	0.3	0.99	-6	15	19	12	3UTR
mmu-miR-214-3p	NM_001356589	Crtac1	ACAGCAGGCACAGACAGGCAGT#GCCTGTGCTGCTGCTGCTGC#.(((((.((((((((((((...#)))))).))).))))))))).	2398	2418	0.96923	-27	0	0.0001072	0.5	0.6	0.88	-5	17	20	8	3UTR
mmu-miR-214-3p	NM_001357026	Gusb	ACAGCAGGCACAGACAGGCAGT#TCTGTGCTAGGCTGG#.((((.((((((((........#))))))))..)))).	976	991	1	-20	0	0.0001503	0.4	1.4	1.31	-7	12	15	8	CDS
mmu-miR-214-3p	NM_001357027	Gusb	ACAGCAGGCACAGACAGGCAGT#TCTGTGCTAGGCTGG#.((((.((((((((........#))))))))..)))).	794	809	1	-20	0	0.0001503	0.4	1.4	1.31	-7	12	15	8	3UTR
mmu-miR-214-3p	NM_001357180	Slc27a1	ACAGCAGGCACAGACAGGCAGT#TTGGCTGCTGTGTCCTGCA#...((((((((((.(((.(((.#))).)))))))).))))).	2326	2345	0.84615	-25	0	3.19E-07	0.4	-0	-0.3	-7	16	19	8	3UTR
mmu-miR-214-3p	NM_001357180	Slc27a1	ACAGCAGGCACAGACAGGCAGT#CTGTGGAGCAAGTGGGGGCCCACATAGCTGCTGTCCCTGCTGA#.(((((((....((((.(((((#.))))))))))))))))).	2441	2484	0.84615	-23	1	3.94E-06	0.4	-0	-0.2	-12	16	18	16	3UTR
mmu-miR-214-3p	NM_001357257	Igdcc3	ACAGCAGGCACAGACAGGCAGT#GCTGCCTGCGCCAAGCCTGGGTCTGGGCCATTCTGCTGA#.((((((((.((((((((((((#)))))))............))))).)))....))))).	380	419	1	-27	1	0.0004564	0.4	0.6	1.34	-5	20	39	7	CDS
mmu-miR-214-3p	NM_001357428	Ildr2	ACAGCAGGCACAGACAGGCAGT#GCTGCTTGCTGATCTCTTGCCCAGTTTTGCTGTG#(((((((((((((.((((((((#)))))))))))......))))......))))))).	791	825	1	-25	1	0.0001338	0.5	4	3.39	-5	21	34	11	CDS

mmu-miR-214-3p	NM_001357428	Ildr2	ACAGCAGGCACAGACAGGCAGT#GCTGCCCTCACAGTTGCTGCTGCTATGTCCGCTGCCCATGCTGC#.((((((((((.((((((((((#.)))))).)))).).))))..))))).	913	957	0.92308	-26	1	0.0012616	0.4	3.9	3.17	-7	20	27	6	CDS
mmu-miR-214-3p	NM_001357428	Ildr2	ACAGCAGGCACAGACAGGCAGT#CTGCCCAGTTGTTTTTGTGCCTTGTCTGTG#((((((((((((((((((((.#)))))....))))..))))))).).)))	7931	7961	0.92308	-25	1	0.0006165	0.6	0.8	0.15	-4	21	30	7	3UTR
mmu-miR-214-3p	NM_001357429	Ildr2	ACAGCAGGCACAGACAGGCAGT#CTGCCCAGTTGTTTTTGTGCCTTGTCTGTG#((((((((((((((((((((.#)))))....))))..))))))).).)))	7727	7757	0.92308	-25	1	0.0006165	0.6	0.8	0.15	-4	21	30	7	3UTR
mmu-miR-214-3p	NM_001357429	Ildr2	ACAGCAGGCACAGACAGGCAGT#ACTGCCTAGCTGAGCCGAGCC#...((.(((.(((..(((((((#)))))))..))).)))..)).	3246	3267	1	-23	0	0.0039313	0.4	1.3	0.12	-11	15	21	7	3UTR
mmu-miR-214-3p	NM_001357429	Ildr2	ACAGCAGGCACAGACAGGCAGT#CTGTCTCTGTCTGTGACTCACAGCTGA#.((((((.(((((((((((((.#))))..))))))))).))....)))).	4835	4862	0.92308	-23	1	4.30E-05	0.5	0.3	0.31	-6	19	27	9	3UTR
mmu-miR-214-3p	NM_130796	Snx18	ACAGCAGGCACAGACAGGCAGT#GCCTGCTGTGGATGCC#...(((..(((((.(((((...#))))))))))..))).	2374	2390	0.84615	-23	0	0.0056876	0.5	1.7	1.53	-11	13	16	10	3UTR
mmu-miR-214-3p	NM_130796	Snx18	ACAGCAGGCACAGACAGGCAGT#GCTGCCTCCTGCTGA#.(((((((.......(((((((#)))))))))))))).	4124	4139	0.88462	-22	1	6.42E-06	0.5	4.4	2.26	-15	14	15	14	3UTR
mmu-miR-214-3p	NM_130858	Nxph3	ACAGCAGGCACAGACAGGCAGT#GCGCCTGTCTGGAGCGGCA#...((..((.((((((((((.((#))))))))))))..)).)).	56	75	0.92308	-23	0	6.21E-07	0.3	0.6	0.76	-11	15	19	11	5UTR
mmu-miR-214-3p	NM_130858	Nxph3	ACAGCAGGCACAGACAGGCAGT#TGCTGGAACTGTGCCCTAGTGTTGTC#(((((((((((((...((((..#))))....)))))))....)))))).	1957	1983	0.84615	-22	1	0.0003318	0.4	0.9	0.64	-6	17	26	7	3UTR
mmu-miR-214-3p	NM_130858	Nxph3	ACAGCAGGCACAGACAGGCAGT#GCCCTGAACCTGCTGG#.(((((((..(((...(((...#))))))..))))))).	688	704	0.92308	-21	1	1.20E-05	0.5	0.3	0.23	-8	13	16	7	CDS
mmu-miR-214-3p	NM_130867	Kirrel	ACAGCAGGCACAGACAGGCAGT#GCTGCTGACACCACCCGTGGGCCAGTAAAGAGGACTGAGGTCTGTGGCTTGTTGTA#(((((((((((((((...((((#.))))..))))))).)))))))).	6303	6359	1	-25	1	0.000362	0.5	2.1	2.76	-4	19	24	8	3UTR
mmu-miR-214-3p	NM_130885	Oxr1	ACAGCAGGCACAGACAGGCAGT#GTCTGTTTGGCCATTTACTGCA#...((((((.((((((((((...#))))))))))))......))).	2598	2620	0.92308	-20	0	0.0177324	0.7	0.7	1.67	-8	15	22	12	3UTR

mmu-miR-214-3p	NM_130885	Oxr1	ACAGCAGGCACAGACAGGCAGT#CCTCGTCTGTGTAAAGCTC#..(((..(((((((((((....#))).))))))))...))).	4005	4024	0.97436	-18	0	0.0046943	0.6	1.4	1.54	-8	14	19	8	3UTR
mmu-miR-214-3p	NM_133186	Steap3	ACAGCAGGCACAGACAGGCAGT#CTGCCTGCCGCTGCGCCGCTC#..(((.(((.(((.(((((((.#)))))))...))).)))))).	1025	1046	0.84615	-25	0	0.0030076	0.3	0.7	0.3	-9	16	21	7	CDS
mmu-miR-214-3p	NM_133186	Steap3	ACAGCAGGCACAGACAGGCAGT#GCTGCCCCATCTGATCCTAGCTGA#.(((((((..((((..((((((#))))))...))))..))).))))).	2134	2158	0.92308	-23	1	0.0036215	0.4	-0	1.99	-7	17	24	6	3UTR
mmu-miR-214-3p	NM_133201	Mfn2	ACAGCAGGCACAGACAGGCAGT#ACTGGACAGCTCGGCTCCAAGGAGTTATGCATGTGTCTGTGTCTGCTC#..((((((((((((((.(((..#.)))...)))))))))))))).	2839	2887	1	-28	1	0.0003483	0.5	3.7	2.91	-5	17	22	14	3UTR
mmu-miR-214-3p	NM_133206	Znrf1	ACAGCAGGCACAGACAGGCAGT#ACTGACCCTGCTGGGCCTGCTT#..((((((((.((((.((((((((#))))..)))))))).)))))))).	1180	1202	0.92308	-26	1	1.97E-05	0.4	3.8	3.03	-7	18	22	7	CDS
mmu-miR-214-3p	NM_133206	Znrf1	ACAGCAGGCACAGACAGGCAGT#GCTGTCATGTCCTCTGAATTTGGACCCAGATTGCCAGTGCCTGGTGTG#(((.((((((((.((((((((((#)))))).))))......................)))))))).))).	4716	4764	0.92308	-26	0	4.09E-05	0.4	-0	0.25	-10	20	48	7	3UTR
mmu-miR-214-3p	NM_133251	Vgll1	ACAGCAGGCACAGACAGGCAGT#ACCGTCTCTTTCTGTCCCTGCTGC#.((((((((.(((((.((((.((#)).))))...))))).)))))))).	1279	1303	1	-23	1	0.0081269	0.5	0.2	0.22	-6	18	24	7	3UTR
mmu-miR-214-3p	NM_133251	Vgll1	ACAGCAGGCACAGACAGGCAGT#CTGCCTCCCTACCCTGTCCACTACAAGAAGAAAATCTGTGCTCGTGGAGCTGC#.((((.((((((((.((((((.#))))))).........................))))))))......)))).	1067	1120	0.84615	-21	0	6.97E-06	0.5	0	0.32	-6	18	53	8	CDS
mmu-miR-214-3p	NM_133255	Hook2	ACAGCAGGCACAGACAGGCAGT#GCTGCCTGCTGGAGTCTTCCAGTTTACATTTACCCAGCTGG#.((((((((.(((.((((((((#))))))))))..))))..................)))).	2253	2294	0.92308	-25	1	0.0001077	0.5	-0	0.16	-6	19	41	11	3UTR
mmu-miR-214-3p	NM_133255	Hook2	ACAGCAGGCACAGACAGGCAGT#GCTGCTTATCAGTGCCTGGTA#..(.((((((((.((.((((((((#)))))))).)).))))))).).	2061	2082	0.84615	-25	0	3.60E-05	0.4	2.3	2.5	-12	17	21	7	CDS
mmu-miR-214-3p	NM_133255	Hook2	ACAGCAGGCACAGACAGGCAGT#GCTGCCGTCGTCGCCTGG#....((((((((.(((.((((((#)))))))))))).))))).	1031	1049	0.92308	-23	0	6.75E-07	0.3	2.3	2.93	-13	16	18	11	CDS

mmu-miR-214-3p	NM_133345	Ing4	ACAGCAGGCACAGACAGGCAGT#GCTATCCATGCTAGCCTGCTGTGTTTATTGTA#((((.((((((((.(((((((#.))).))))))))))))).))))	1342	1374	0.92308	-24	0	0.0056677	0.6	0.3	0.39	-9	20	24	13	3UTR
mmu-miR-214-3p	NM_133363	Myoz3	ACAGCAGGCACAGACAGGCAGT#CTGCCTATGATGTGTGTCTGTGGGAAGTTAGAGCCATGCCTGCC#...((((((((((((((((.#))))))).........))))))...............)))))).	1344	1388	0.84615	-25	0	0.0014495	0.5	-0	-0.1	-7	18	44	6	3UTR
mmu-miR-214-3p	NM_133363	Myoz3	ACAGCAGGCACAGACAGGCAGT#TGCTTGTCTTGACTTTGCTTGCTT#..((((((((((((((((..#)))))))))).)....)))))))).	813	837	0.92308	-23	1	0.0111247	0.6	0.3	0.13	-5	18	24	9	3UTR
mmu-miR-214-3p	NM_133661	Slc6a12	ACAGCAGGCACAGACAGGCAGT#GCTAACAAGCCTGCATGGATCACCTGCTGTG#((((((((..((..(((((...#.)))))..)).....)))))))).	2531	2562	0.92308	-24	1	0.0004582	0.5	0	0	-8	15	24	8	3UTR
mmu-miR-214-3p	NM_133661	Slc6a12	ACAGCAGGCACAGACAGGCAGT#GCTGGCCTTGTGCCTCCTGC#.(((.(((((((...(((((((#)))).))))))))))).))).	952	972	1	-22	0	0.0014479	0.4	2.9	2.55	-11	17	20	10	CDS
mmu-miR-214-3p	NM_133674	Arhgef5	ACAGCAGGCACAGACAGGCAGT#ACTGTCTGTTGCTGTCTCGGCC#...(((((((..((((((((((#))))))))))..)))))..)).	4334	4356	1	-21	0	0.0004021	0.4	3.2	2.67	-13	17	22	10	CDS
mmu-miR-214-3p	NM_133687	Cxxc5	ACAGCAGGCACAGACAGGCAGT#TGTCAATGTTGTCTGTGCGGGAGATGCTGTT#((((((.(((((((((((((..#)))).....)))))))))))......))	1652	1683	0.92308	-22	0	0.0001999	0.6	4	4.35	-5	19	31	9	3UTR
mmu-miR-214-3p	NM_133719	Metrn	ACAGCAGGCACAGACAGGCAGT#GCTGCCCTTCTAACCCTTCTTAGCCTGCTGA#.((((((((..(((..((((((#))))))..)))...........))))))	1187	1218	0.84615	-25	1	0.0044265	0.5	1.6	1.08	-9	17	31	8	3UTR
mmu-miR-214-3p	NM_133719	Metrn	ACAGCAGGCACAGACAGGCAGT#CCAGCTCTGTGCCTGCC#...((((((((((((.((....#)).).))))))))))).	1147	1164	1	-23	0	0.004539	0.4	2.3	1.83	-7	14	17	11	3UTR
mmu-miR-214-3p	NM_133753	Errfi1	ACAGCAGGCACAGACAGGCAGT#GCTGCCTGTGGTATGGCCTCTGC#.(((.(((((....((((((((((#))))))))))......))))))).	1553	1576	0.84615	-25	0	3.26E-07	0.5	1.2	0.71	-17	16	23	9	CDS
mmu-miR-214-3p	NM_133753	Errfi1	ACAGCAGGCACAGACAGGCAGT#ACGAGCTGGTCTTGTGTTGCTGG#.(((((.((((((((.(((.((#))..))).)))).))))))))).	2549	2572	1	-21	0	0.0001184	0.6	3.3	1.22	-6	18	23	9	3UTR
mmu-miR-214-3p	NM_133753	Errfi1	ACAGCAGGCACAGACAGGCAGT#ACTGTTGGATGTGCTGCTC#..((((.(((((..(((.(((((#)))))))..))))))))).	467	486	0.84615	-20	0	7.27E-05	0.4	-1	0.69	-9	16	19	9	CDS

mmu-miR-214-3p	NM_133756	Gpn1	ACAGCAGGCACAGACAGGCAGT#GCCTGGTGTTGGATGAGTTCTACAGCTCACTCAGGGTGGTGGGTGTGTCTGCTGTG#((((((((((((..((..(((.(#.).)).)).))..)))))))))))).	714	770	0.90385	-24	1	1.99E-07	0.4	4.4	3.81	-6	17	23	12	CDS
mmu-miR-214-3p	NM_133756	Gpn1	ACAGCAGGCACAGACAGGCAGT#GCCCCCCGTGTGTCTGTTGG#.(((((((((((....(((...#))).....))))))))))).	82	102	1	-23	1	0.0001927	0.4	2.8	1.89	-7	14	20	11	CDS
mmu-miR-214-3p	NM_133764	Atp6v0e2	ACAGCAGGCACAGACAGGCAGT#GCTACTGCTGTCTGCTGTT#((((((((((((...((((...#))).))).)))))))))).	308	327	0.92308	-22	1	0.003032	0.5	2.6	3.15	-6	16	19	10	CDS
mmu-miR-214-3p	NM_133828	Creb1	ACAGCAGGCACAGACAGGCAGT#TTGTCCTGCTGAGCTTGCTGG#.((((((((.(((.(((((((.#))).))))))).)))))))).	2916	2937	0.92308	-26	1	8.12E-05	0.5	1	1.29	-6	18	21	8	3UTR
mmu-miR-214-3p	NM_133828	Creb1	ACAGCAGGCACAGACAGGCAGT#GCTGCCTGTGGCCCGGGCGGCTGG#.((((.(.(....(((((((((#)))))))))......).).)))).	61	85	1	-24	0	5.03E-07	0.3	2.5	2.76	-17	15	24	9	5UTR
mmu-miR-214-3p	NM_133828	Creb1	ACAGCAGGCACAGACAGGCAGT#CTGTCTGTGCTAGCA#...((.(((((((((((.....#)))))))))))).)).	2956	2971	0.92308	-23	0	0.0003018	0.6	0.6	1.05	-8	13	15	11	3UTR
mmu-miR-214-3p	NM_133850	Commd7	ACAGCAGGCACAGACAGGCAGT#GCTGCCTGATTCTTCTGCAAGCCCCACTGG#.(((..(((.((((((((((((#))))))))).....))))...)))...)	829	859	0.84615	-23	0	0.0083119	0.4	0.2	0.04	-11	18	30	8	3UTR
mmu-miR-214-3p	NM_133850	Commd7	ACAGCAGGCACAGACAGGCAGT#ATTGCCAGCCACTGTCTGCCGAGTCGTCTGTGTAAAAGCCTGTG#...((((((((((((((((((#.)))))))........))))))......)))))).	1100	1144	0.84615	-23	0	0.0643717	0.5	-0	0.01	-7	19	35	7	3UTR
mmu-miR-214-3p	NM_133916	Eif3b	ACAGCAGGCACAGACAGGCAGT#GCCCATGCTGTGGATGCTGTG#((((((..((((((.(((((...#)))..)))))))..)))))).	743	764	0.87692	-23	0	0.0002743	0.4	4.2	3.83	-9	16	21	7	CDS
mmu-miR-214-3p	NM_133916	Eif3b	ACAGCAGGCACAGACAGGCAGT#TTGCCCTGTTTGGGTTTTTATCAGCCGTGTCTGCA#...((((((((((((((((((.#))))).)))))...............)))))))).	2659	2694	0.84615	-21	0	0.0777712	0.5	0.3	0.25	-7	18	35	8	3UTR
mmu-miR-214-3p	NM_133917	Mlxip	ACAGCAGGCACAGACAGGCAGT#TGTGTGTGTGTGTGTGCTGG#.(((((.(((((.(((.(((..#))).))).))))).))))).	4987	5007	0.84615	-27	0	0.0114296	0.5	0.7	0.11	-7	16	20	5	3UTR

mmu-miR-214-3p	NM_133917	Mlxip	ACAGCAGGCACAGACAGGCAGT#GCTGGGGCCCTTCTGTGCCTGTTC#..((((((((((((..(((...#.)))..)))))))))))).	3668	3692	1	-27	1	0.0650625	0.4	-0	-0.4	-6	15	19	12	3UTR
mmu-miR-214-3p	NM_133917	Mlxip	ACAGCAGGCACAGACAGGCAGT#GCTCGCCTGAGCCTGGTCCTCTTGAAGCCTGCTGTC#(((((((((.(((.((((((((#))).)))))...)))..........))))))))).	7172	7208	0.84615	-26	1	0.0023872	0.4	-0	0.7	-6	20	36	9	3UTR
mmu-miR-214-3p	NM_133940	Fbxl14	ACAGCAGGCACAGACAGGCAGT#GCCCTTCTAGTGCCCAGGCTGG#.((((.((((((((..(((...#)))..))).)))))...)))).	3610	3632	0.92308	-23	0	2.62E-07	0.4	0.1	0.66	-7	15	22	5	3UTR
mmu-miR-214-3p	NM_133940	Fbxl14	ACAGCAGGCACAGACAGGCAGT#GCTGCCTGGGCCTGGAGCAGCTC#..(((..((.(((.((((((((#))))))))...)))..)).))).	790	813	0.84615	-22	0	2.87E-07	0.3	4.1	3.54	-11	16	23	8	CDS
mmu-miR-214-3p	NM_133947	Numa1	ACAGCAGGCACAGACAGGCAGT#TTGCCTGCACCCTGGCCTGCTA#..((((((((.(((.(((((((.#)))))))....)))))))))).	6580	6602	0.92308	-26	1	0.0025794	0.4	0.4	0.71	-7	17	22	10	3UTR
mmu-miR-214-3p	NM_133947	Numa1	ACAGCAGGCACAGACAGGCAGT#GCCCATGGCCTGCTGG#.((((((((.((....(((...#)))..)))))))))).	4470	4486	1	-23	1	5.62E-08	0.3	1.7	1.51	-8	13	16	10	CDS
mmu-miR-214-3p	NM_133993	Pwp1	ACAGCAGGCACAGACAGGCAGT#GCTGCCTTGTGACTGCTTCCGCTGA#.((((((((...((((((((#))))))).))...)))))..)))).	1304	1329	0.84615	-23	1	0.0020267	0.5	4.6	3.59	-11	18	25	7	CDS
mmu-miR-214-3p	NM_133993	Pwp1	ACAGCAGGCACAGACAGGCAGT#ACTCCTGTCTGCATACCCTCTGA#.(((.(((..((((((((.(((#))))))))))).....)))))).	654	677	1	-20	0	0.0152987	0.5	3	4.03	-10	17	23	11	CDS
mmu-miR-214-3p	NM_134007	Cisd1	ACAGCAGGCACAGACAGGCAGT#GCTGCACCCAGCGTGTTGTGATGTCACCTGCTGA#.((((((((((.(((.((...#.)).))))))))......))	436	470	0.92308	-22	1	5.26E-05	0.4	0.6	0.77	-5	16	25	7	3UTR
mmu-miR-214-3p	NM_134009	Ncln	ACAGCAGGCACAGACAGGCAGT#GCTGCTTCTGTCCCTCACGCCTGCTGA#.((((((((((((..((((((#)))))))))))).......)))))))).	2549	2576	0.84615	-27	1	9.26E-05	0.3	3.1	3.46	-5	19	27	11	3UTR
mmu-miR-214-3p	NM_134009	Ncln	ACAGCAGGCACAGACAGGCAGT#GCCTTCGTTCTGTGCCTGGACACTGTG#((((((((((((((((((((...#)))))..)).)))))))))....))))).	1015	1042	0.92308	-26	1	0.0001723	0.4	0.1	0.18	-4	19	27	9	CDS
mmu-miR-214-3p	NM_134009	Ncln	ACAGCAGGCACAGACAGGCAGT#GCATGCGTGTCGTAGAGCGAGAGGGCTCAGCCTGCTGC#.((((((((((.((((.(((((#)).))).))))))................)))))))).	2641	2679	0.89231	-24	1	1.07E-06	0.4	3	3.35	-7	19	38	8	3UTR

mmu-miR-214-3p	NM_134037	Acly	ACAGCAGGCACAGACAGGCAGT#GCTGCTTGTGGGTGTGGACGAAAAGCTGA#.((((..((((..(((((((((#)))))))))..))))........)))	681	710	0.92308	-22	0	0.0502609	0.5	0.8	1.15	-12	17	29	9	CDS
mmu-miR-214-3p	NM_134037	Acly	ACAGCAGGCACAGACAGGCAGT#TTGCTCTGCTACCTGCTGTA#((((((((...((.((((((((.#)))).))))).)))))))).	3753	3773	0.84615	-22	1	0.0122395	0.6	4.4	3.76	-10	17	20	8	3UTR
mmu-miR-214-3p	NM_134044	Vipas39	ACAGCAGGCACAGACAGGCAGT#CTTAGGAGAGTCACAGCATTCTACACGTGAGCCTGTCATTCACAGGCAGTGCCTGCTGG#.((((((((((.(((((((...#.)))))))).........))	1640	1699	0.92308	-32	1	8.74E-08	0.5	2.3	2.68	-7	17	30	10	3UTR
mmu-miR-214-3p	NM_134046	Cenpo	ACAGCAGGCACAGACAGGCAGT#CTGCCTGCTGGTCATGCCCTCTCATTACAACGCCTGCG#...((((((.(((.(((((((.#))))))))))....................)))))).	1907	1945	0.84615	-25	0	0.0451244	0.4	1.5	0.18	-9	16	38	10	3UTR
mmu-miR-214-3p	NM_134046	Cenpo	ACAGCAGGCACAGACAGGCAGT#CTGTGCTGTGCAGTGTGTGTTGTT#((((((.((((.(((((((((.#)))).)))).).)))).)))))).	1852	1876	0.92308	-22	0	0.0103751	0.5	1.2	0.18	-9	19	24	6	3UTR
mmu-miR-214-3p	NM_134074	Dock9	ACAGCAGGCACAGACAGGCAGT#TGAACCCGGGCAGTATCGTGAAGGACGAAGCCCTGGCTGTGCCTGCTGG#.(((((((((((((.((((....#.)))).))))))))))))).	3678	3727	1	-32	1	4.96E-05	0.4	4.5	4.1	-5	16	19	12	CDS
mmu-miR-214-3p	NM_134103	Il1rap	ACAGCAGGCACAGACAGGCAGT#GCTGTCGCCGAGCCTGCTGG#.((((((((......((((((#))))))....)))))))).	148	168	0.84615	-23	1	4.09E-06	0.4	-0	0.48	-14	14	20	8	5UTR
mmu-miR-214-3p	NM_134125	Trip10	ACAGCAGGCACAGACAGGCAGT#CCTGTGTAACCTGCTGTC#((((((((...(.((((((....#)))))).)..)))))))).	1852	1870	1	-20	1	0.0523865	0.4	1.5	0.62	-10	14	18	8	3UTR
mmu-miR-214-3p	NM_134125	Trip10	ACAGCAGGCACAGACAGGCAGT#GCCGCCGAGTCTGTGGATGCTA#..((((..(((((((.(((.((#)).)))..)))))))..)))).	859	881	1	-24	0	4.63E-05	0.5	1	2.88	-9	16	22	7	CDS
mmu-miR-214-3p	NM_134138	Psmg2	ACAGCAGGCACAGACAGGCAGT#CTGCTGAGATCACAAAAGAGGCTGTTGGGTGTGTGCTGTA#((((((.((((.(((((.(...#.).)))))..)))).)))))).	911	951	0.92308	-25	0	0.0006568	0.5	0	0.4	-9	16	22	6	3UTR
mmu-miR-214-3p	NM_134138	Psmg2	ACAGCAGGCACAGACAGGCAGT#ATTGCCTGGTGCCAATGGTTGG#.((((.((((((...((((((((#))))))))))))))....)))).	240	262	1	-21	0	0.000114	0.6	4	2.93	-11	17	22	13	CDS

mmu-miR-214-3p	NM_134142	Tmem109	ACAGCAGGCACAGACAGGCAGT#GCCATCTCTGTGGCCTGCTT#..((((((((((((..(((...#)))...)))))).))))))).	370	390	0.84615	-25	1	0.0001594	0.4	-0	0.06	-6	15	20	6	CDS
mmu-miR-214-3p	NM_134142	Tmem109	ACAGCAGGCACAGACAGGCAGT#ACTGGTTGCTGTCCCTGCTC#..((((((.((((.(((.((((#)))).))))))).)))))).	524	544	0.92308	-24	1	8.88E-06	0.4	1.1	0.41	-7	17	20	7	CDS
mmu-miR-214-3p	NM_134437	Il17rd	ACAGCAGGCACAGACAGGCAGT#GCCATCACTGTGCCTCTGG#.(((.((((((((...(((...#)))....)))))))))))).	1006	1025	1	-23	0	0.0040904	0.4	3.7	3.72	-10	14	19	11	CDS
mmu-miR-214-3p	NM_134437	Il17rd	ACAGCAGGCACAGACAGGCAGT#ACTGATCCTTGGATCTGAGCCTGCTC#..(((((((.((((((((((((#))))...))))..)))).))))))).	2572	2598	0.84615	-22	1	0.0001576	0.5	0.2	0.26	-6	19	26	7	3UTR
mmu-miR-214-3p	NM_138306	Dgkz	ACAGCAGGCACAGACAGGCAGT#GCTCAAGGAAGCCTCTGTGCCTCTGG#.(((.(((((((((..(((...#.))))))))))))))).	2114	2140	0.84615	-25	0	5.22E-07	0.4	4.7	2.52	-9	15	17	15	CDS
mmu-miR-214-3p	NM_138306	Dgkz	ACAGCAGGCACAGACAGGCAGT#ACTCAAGAGCCTGCTGTATTCACTTGCCCGCTGC#.((((.(((((((.(((((...#.)))))))))).......)	3315	3349	0.84615	-24	0	0.0033838	0.4	1.8	1.38	-7	16	27	9	3UTR
mmu-miR-214-3p	NM_138659	Prpf8	ACAGCAGGCACAGACAGGCAGT#CTAGCCGGTTCCCGCCTGTTGTA#(((((((((...(((.(((((.#)).))).)))...))))))))).	4237	4260	0.92308	-22	1	0.0002207	0.4	4.1	4.83	-12	17	23	9	CDS
mmu-miR-214-3p	NM_138666	Nlgn1	ACAGCAGGCACAGACAGGCAGT#TGCTTGGTGGCTGTGCTGCTGG#.(((((.((((((.((((((..#))))))....)))))))))))).	4498	4520	1	-24	0	2.00E-05	0.6	-0	0.1	-6	17	22	11	3UTR
mmu-miR-214-3p	NM_138666	Nlgn1	ACAGCAGGCACAGACAGGCAGT#CTGCTTGTTGGGATGTTTGCTA#..(((((((((.(((((((((.#)))))))))..).)))))))).	1393	1415	0.84615	-23	1	0.0004424	0.5	0.1	-0	-8	18	22	9	CDS
mmu-miR-214-3p	NM_138666	Nlgn1	ACAGCAGGCACAGACAGGCAGT#GCGGCATCTGTCTGTGTGAAATCAATGGGAGCTACTTACTGCATGA#.(((((((((((((((((.((#)).))..))))))))))....................)))).))).	1118	1164	0.84615	-21	1	0.0065081	0.6	-0	0.08	-4	20	46	10	5UTR
mmu-miR-214-3p	NM_138744	Ssx2ip	ACAGCAGGCACAGACAGGCAGT#CTGCCTTTCTCTTTCCTTGTTCCTGCTGTG#((((((((.(((((.((((((.#)))))).)))........)).))))))))	3379	3409	0.92308	-25	1	0.0979845	0.6	0.4	-0.1	-6	19	30	8	3UTR
mmu-miR-214-3p	NM_138744	Ssx2ip	ACAGCAGGCACAGACAGGCAGT#TTGTTTGTTTGGACTTCGCTGTC#((((((((..(((((((((((.#))))))))))..))).))))).	2226	2249	1	-22	1	0.000716	0.6	1.4	1.28	-6	19	23	11	3UTR

mmu-miR-214-3p	NM_138748	Ptpa	ACAGCAGGCACAGACAGGCAGT#ACTGCCCATCCTGTGTTTGTGCCCGCTT#..(((.((((((((((((((#)))))).......)))))))))).))).	2310	2338	0.92308	-28	0	0.0059927	0.5	-0	0.2	-7	19	28	10	3UTR
mmu-miR-214-3p	NM_138748	Ptpa	ACAGCAGGCACAGACAGGCAGT#GCTGCTGCTGTGCCTGAAGTG#((..(((((((((.((.(((((#))))))))))))))))..)).	636	657	1	-27	0	2.34E-05	0.4	3	3.36	-9	18	21	16	CDS
mmu-miR-214-3p	NM_138748	Ptpa	ACAGCAGGCACAGACAGGCAGT#GCTGCCTCTGCCTGCTGA#.(((((((((.....((((((#))))))).))))))))).	2026	2044	0.92308	-27	1	0.0004732	0.4	-0	-0.3	-13	16	18	9	3UTR
mmu-miR-214-3p	NM_138950	Wdr81	ACAGCAGGCACAGACAGGCAGT#CCTGCTGCGCCTGCTGA#.((((((((.(((.((((....#))))))).)))))))).	4000	4017	0.92308	-28	1	0.0009534	0.4	2.9	2.41	-6	15	17	8	CDS
mmu-miR-214-3p	NM_138950	Wdr81	ACAGCAGGCACAGACAGGCAGT#GCCTGAGATGTGGCCCCTGCTGC#.(((((((((((..(((((...#)))))...))))...))))))).	6821	6844	0.92308	-26	1	5.95E-05	0.5	-0	0.64	-6	16	23	7	3UTR
mmu-miR-214-3p	NM_144517	Tbc1d19	ACAGCAGGCACAGACAGGCAGT#GCTGCCGTGTTTGCTT#..(((((((((.....((((((#)))))))))))))))).	1576	1592	1	-20	1	0.0020713	0.5	4.9	4.36	-11	15	16	15	CDS
mmu-miR-214-3p	NM_144522	Tbc1d10b	ACAGCAGGCACAGACAGGCAGT#CTGCCTGTCACCCCACTGCCTACTGG#.(((.(((((..(((((((((.#)))))))))).......))))).))).	2848	2874	0.92308	-26	0	0.0002035	0.4	1.5	0.84	-14	17	26	9	3UTR
mmu-miR-214-3p	NM_144522	Tbc1d10b	ACAGCAGGCACAGACAGGCAGT#GCTGCGTGTCTGGGACATGTTTTTCTGTG#((((.((((((((((((.(((((#))))).))))))).....)))))..)))).	1673	1702	0.84615	-23	0	0.0007737	0.5	3	2.35	-8	20	29	6	CDS
mmu-miR-214-3p	NM_144525	Tmem214	ACAGCAGGCACAGACAGGCAGT#GCTGCTTCGATCCTCCGGTCTCTTGCCTGTTGG#.(((((((((.(((((((((((#)))))))).........))))..	1630	1663	0.84615	-26	1	5.99E-05	0.4	1.2	1.68	-7	20	33	9	CDS
mmu-miR-214-3p	NM_144525	Tmem214	ACAGCAGGCACAGACAGGCAGT#GCGCCCTCTGTCAGCCTGCTGA#.(((((((((((((..(((.((#))))).)))))..)))))))).	2757	2779	0.88462	-25	1	1.02E-06	0.4	0.3	0.18	-5	18	22	8	3UTR
mmu-miR-214-3p	NM_144525	Tmem214	ACAGCAGGCACAGACAGGCAGT#GCCAGCCTGACCCAGTGCCTGACTGTG#(((((((((((.(.(((((.((#))..))))).)...))))))).)))).	1247	1274	1	-25	1	0.0002069	0.4	2.5	2.93	-8	19	27	7	CDS
mmu-miR-214-3p	NM_144557	Myrip	ACAGCAGGCACAGACAGGCAGT#GCCTGATCTGGAGTCAGCTGTG#(((((.(((.((((((((((...#))))).))))..))).))))).	2701	2723	0.84615	-26	0	3.72E-05	0.5	1.4	1.3	-7	17	22	5	CDS

mmu-miR-214-3p	NM_144557	Myrip	ACAGCAGGCACAGACAGGCAGT#TGCTGGTCTCGCTGGGTGCCTGCA#...((((((((((((.((((..#)))).))))......)))))))).	3751	3775	0.92308	-25	0	2.22E-05	0.5	-0	0.14	-7	16	24	8	3UTR
mmu-miR-214-3p	NM_144557	Myrip	ACAGCAGGCACAGACAGGCAGT#ACTGCCTGCCCCTCCTGTGAAAGCTGA#.((((...(((((.((((((((#))))))))......)))))...)))).	2557	2584	1	-24	0	0.0291427	0.5	2.5	2.01	-10	17	27	8	CDS
mmu-miR-214-3p	NM_144557	Myrip	ACAGCAGGCACAGACAGGCAGT#GTTTGCTGTGTCTGCC#...((((((((((.(((((...#))))))))))))))).	467	483	0.92308	-24	0	7.72E-05	0.5	3.4	3.33	-7	15	16	15	CDS
mmu-miR-214-3p	NM_144731	Galnt7	ACAGCAGGCACAGACAGGCAGT#ACCGCCTGGAACCTGCTGC#.(((((((......(((((.((#)).)))))...))))))).	2110	2129	0.96154	-21	1	0.0012447	0.6	2.1	2.56	-14	14	19	7	3UTR
mmu-miR-214-3p	NM_144809	Prdm9	ACAGCAGGCACAGACAGGCAGT#GCTGGCCTCGAACTCTGAAATCTGCCTGCC#...((((((((((((((((((#)))).))).)...)))).....)))))	3007	3037	0.84615	-21	0	0.0120101	0.4	0	0.16	-7	19	30	7	3UTR
mmu-miR-214-3p	NM_144809	Prdm9	ACAGCAGGCACAGACAGGCAGT#GCTGCCTTGTCAGCCATAAGTGCTT#..(((((((...((((((((((#))))))).))).)))).....)))).	3188	3213	0.92308	-21	1	0.0003043	0.6	0.4	1.11	-12	17	25	7	3UTR
mmu-miR-214-3p	NM_144822	Micu1	ACAGCAGGCACAGACAGGCAGT#ACTGCCTGGGACTTTGCTCTGCC#...(((((((.((.((((((((#))))))))...)).))).)))).	1530	1553	0.92308	-24	0	5.18E-05	0.5	3.3	2.95	-10	17	23	8	CDS
mmu-miR-214-3p	NM_144822	Micu1	ACAGCAGGCACAGACAGGCAGT#CTGCTCTGACATCTGTCTCTCGCTGTG#((((((((.((((((((((((.#)))).)))...))))).))).)))))).	1872	1899	0.84615	-23	1	0.0006184	0.5	-0	0.14	-5	20	27	5	3UTR
mmu-miR-214-3p	NM_144822	Micu1	ACAGCAGGCACAGACAGGCAGT#CTGCCATTTTCCTCCTGCTGTT#((((((((...(((..(((((.#)))))..)))...)))))))).	1923	1945	0.92308	-22	1	0.0060417	0.5	-0	0.23	-10	16	22	8	3UTR
mmu-miR-214-3p	NM_144884	Tor1a	ACAGCAGGCACAGACAGGCAGT#CCTGTTTGTGGCCACGCTA#..(((.((((((((((((....#)))))))))).))..))).	456	475	0.84615	-21	0	1.72E-05	0.5	2.8	1.5	-7	15	19	10	CDS
mmu-miR-214-3p	NM_144905	Tmem268	ACAGCAGGCACAGACAGGCAGT#TGCAGCTGTGCCTGTTGA#.((((((((((((.(..(((..#))).))))))))))))).	194	212	0.92308	-26	1	0.0002855	0.5	0.2	-0	-5	16	18	13	5UTR
mmu-miR-214-3p	NM_144905	Tmem268	ACAGCAGGCACAGACAGGCAGT#GCTGTCTGTCTGCCATGGCTTGTG#...((((((.(((((((((((((#))))))))))))).....)))))).	332	356	1	-25	0	3.64E-06	0.4	-0	0.51	-8	18	24	12	5UTR

mmu-miR-214-3p	NM_144918	Smyd5	ACAGCAGGCACAGACAGGCAGT#CTGCCACAGCTGCAGCTGAAGTACTGGCCTGCC#...((((((.(((.((((((((#.)))))..))).....)))	1757	1790	0.84615	-22	0	2.45E-05	0.4	-0	-0.1	-9	17	26	9	3UTR
mmu-miR-214-3p	NM_144935	Plppr2	ACAGCAGGCACAGACAGGCAGT#GCTCTCGCCTGGTGAAACCTTCCCTCTGCCTGGCCCTGCTGTG#((((((((......(((((((.#.)))))))..)))))))).	999	1042	0.84615	-26	1	0.0008208	0.3	3.2	2.52	-15	15	19	8	CDS
mmu-miR-214-3p	NM_144954	Ppil2	ACAGCAGGCACAGACAGGCAGT#GCGCCATAGTCAGTGTCTGCTGTG#((((((((((((.(((.(((.((#)))))...))).)))))))))))).	32	56	1	-27	1	0.0015739	0.4	0.7	0.42	-8	19	24	11	5UTR
mmu-miR-214-3p	NM_144954	Ppil2	ACAGCAGGCACAGACAGGCAGT#GCTCTGTTTGCTCGAGCTTGCTGGTCCTGCTGTT#((((((((..(((.(((((...#.))))))))..))))))))	1825	1859	1	-26	1	0.0020201	0.4	3.9	3.01	-7	16	20	8	3UTR
mmu-miR-214-3p	NM_145066	Gpr85	ACAGCAGGCACAGACAGGCAGT#GCCTGATTTGGGGCCGGCTGA#.((((.(((.((((((((...#))))).))))..))).))))).	591	612	0.92308	-25	0	0.0046155	0.4	2	1.31	-7	16	21	5	5UTR
mmu-miR-214-3p	NM_145066	Gpr85	ACAGCAGGCACAGACAGGCAGT#CTGCTGGATCTGTGCTGCTC#..((((.((((((((.(((((.#))))).).)))))))))))).	1389	1409	0.84615	-24	0	9.77E-07	0.5	3.8	5.07	-7	17	20	11	CDS
mmu-miR-214-3p	NM_145066	Gpr85	ACAGCAGGCACAGACAGGCAGT#GCTGTCTGGATGAGTTTCGCCCAAGCAGGAATCAATCCCTTTGTCTGCA#...(((((((((..((((((((#))))))))..))...........................)))))))	2184	2233	0.84615	-22	0	0.0043402	0.5	4.2	4.47	-9	17	49	8	CDS
mmu-miR-214-3p	NM_145123	Crtac1	ACAGCAGGCACAGACAGGCAGT#TGTATCCTGTGCCTGCTGA#.((((((((((((....(((..#)))...)))))))))))).	2459	2478	1	-26	1	0.0129309	0.5	0.3	0.99	-6	15	19	12	3UTR
mmu-miR-214-3p	NM_145123	Crtac1	ACAGCAGGCACAGACAGGCAGT#GCCTGTGCTGCTGCTGCTGC#.(((((.((((((((((((...#)))))).))).)))))))).	2482	2502	0.96923	-27	0	0.0001072	0.5	0.6	0.88	-5	17	20	8	3UTR
mmu-miR-214-3p	NM_145356	Zbtb7c	ACAGCAGGCACAGACAGGCAGT#CTGCTTGACCTGTGACCTTAGGAGGGTCAACCCTCTGTGCCTCAGAGTCACCCTGCTGTG#((((((((((((((((((((.#))))))))..........................)))))))))............))))).	2921	2981	1	-27	1	0.0002188	0.4	0.2	-0	-4	21	60	9	3UTR
mmu-miR-214-3p	NM_145356	Zbtb7c	ACAGCAGGCACAGACAGGCAGT#CTGTCTGTCTGTGGGAGACCTTTGG#.(((.((((((((((((((((.#))))))))))))).....)))))).	4358	4383	1	-25	0	6.79E-05	0.6	1.6	1.39	-8	19	25	13	3UTR

mmu-miR-214-3p	NM_145356	Zbtb7c	ACAGCAGGCACAGACAGGCAGT#CTGCCTGCCAGTGCCA#......(((((.(.(((((((.#))))))).).))))).	3882	3898	0.90385	-23	0	0.0154576	0.4	-0	0.22	-20	13	16	7	3UTR
mmu-miR-214-3p	NM_145399	Scgn	ACAGCAGGCACAGACAGGCAGT#TGTCTGTGTTCCTGCTGTG#((((((((((((((((......#))))))))..)))))))).	1040	1059	0.92308	-26	1	0.017395	0.5	0.7	0.57	-4	16	19	8	3UTR
mmu-miR-214-3p	NM_145399	Scgn	ACAGCAGGCACAGACAGGCAGT#GCTCGGCTGGATGCTGCCTGCTT#..((((((((((..(((.((((#))).).)))..)).)))))))).	208	231	0.92308	-25	1	2.69E-09	0.4	0.2	-0.2	-7	17	23	8	CDS
mmu-miR-214-3p	NM_145400	Ube4a	ACAGCAGGCACAGACAGGCAGT#GCTGCCTCCAGTGTCTGTATTCTGCTT#..((((((.(((((((((((((#)))))))).....))))))..)))))).	5185	5212	0.92308	-25	1	0.0214602	0.5	3.4	3.94	-6	19	27	7	3UTR
mmu-miR-214-3p	NM_145400	Ube4a	ACAGCAGGCACAGACAGGCAGT#GCTGCAAAACTGTCTGAATTTGCAGGTGTCCATGGCTGTTCTGCTGG#.(((((((((((((((((((((#)))))....))))))).................))).)))))).	1991	2038	0.84615	-24	1	0.0001573	0.5	-0	0.23	-4	21	47	7	CDS
mmu-miR-214-3p	NM_145400	Ube4a	ACAGCAGGCACAGACAGGCAGT#CCTGTCTGATCCCAGCTGTG#(((((.((..((((((((....#))))))))...)).))))).	1717	1737	0.84615	-22	0	9.99E-05	0.4	0.7	0.65	-8	15	20	8	CDS
mmu-miR-214-3p	NM_145426	Mfap3	ACAGCAGGCACAGACAGGCAGT#ACTGCCTGTCCATCTTACTGGTGGTCACTCTTGTGCCAGCTGC#.((((.(((((((((((((((#))))))))))...................)))))).)))).	254	297	0.92308	-32	0	0.0063081	0.4	1	1.05	-6	20	43	10	CDS
mmu-miR-214-3p	NM_145452	Rasa1	ACAGCAGGCACAGACAGGCAGT#GCTTTCACCAAGGGGACTCTGTCTTTGCCTGCTGA#.((((((((.(((((((....#.))))))).)))))))	894	929	0.84615	-28	1	0.0001531	0.3	3.4	2.23	-7	16	19	9	CDS
mmu-miR-214-3p	NM_145524	Mettl8	ACAGCAGGCACAGACAGGCAGT#GCAGCTCTGCTGGTCTGCTGTC#(((((((((.(((.(((((.((#)).)).))))))))))))))).	1521	1543	1	-25	1	3.16E-05	0.5	3.6	2.01	-6	19	22	15	3UTR
mmu-miR-214-3p	NM_145524	Mettl8	ACAGCAGGCACAGACAGGCAGT#ACTGTCTGTGCTGTGGTGGCC#...((.(.((((((((((((((#)))))))))).))))).).)).	2024	2045	1	-24	0	0.0002929	0.6	2.3	1.7	-11	17	21	9	3UTR
mmu-miR-214-3p	NM_145524	Mettl8	ACAGCAGGCACAGACAGGCAGT#CTGCCTCCTTGACTTCCTGCTGTA#((((((((..(((..((((((.#))))))..)))....)))))))).	2291	2315	0.92308	-23	1	0.0165881	0.5	-1	-0.4	-8	17	24	8	3UTR
mmu-miR-214-3p	NM_145545	Gbp7	ACAGCAGGCACAGACAGGCAGT#CCTGGCTGTGCTTCTGA#.(((.((((((((.((((....#)))).)))))))))))).	478	495	1	-22	0	1.50E-05	0.5	1.7	1.75	-9	15	17	11	CDS

mmu-miR-214-3p	NM_145545	Gbp7	ACAGCAGGCACAGACAGGCAGT#GCTCCTGTGTGCCTAGTGG#.((..((((((..(((((.(((#))))))))))))))..)).	152	171	1	-21	0	0.0004454	0.5	1.5	0.43	-16	16	19	14	CDS
mmu-miR-214-3p	NM_145557	9430015G10Rik	ACAGCAGGCACAGACAGGCAGT#GCTCCTGACTGGCCTTCTGA#.(((.((((.(((.((((.(((#))))))).))))))).))).	181	201	0.84615	-22	0	0.0129436	0.4	1.6	0.66	-10	17	20	7	CDS
mmu-miR-214-3p	NM_145557	9430015G10Rik	ACAGCAGGCACAGACAGGCAGT#GCGCTAGCTGTGTCCGGTGCTGG#.((((((((((((..((((...#)).))..)))))))...))))).	339	362	0.92308	-22	1	2.35E-05	0.4	0.9	0.77	-5	16	23	7	CDS
mmu-miR-214-3p	NM_145582	Ctu1	ACAGCAGGCACAGACAGGCAGT#CTGCCAGGCCTGTGCCCTCCTGG#.(((..((((((.(.(((((.#)))))..).)))))))...))).	973	996	0.84615	-24	0	0.0018304	0.3	2.6	2.4	-11	16	23	7	CDS
mmu-miR-214-3p	NM_145582	Ctu1	ACAGCAGGCACAGACAGGCAGT#ACTGACCTGTTTCCGTGTGGAGTGGCTGTA#(((((..((((((((((((((#)))).)))))))..))))......)))	1464	1494	0.84615	-23	0	0.0007051	0.4	0.1	0.05	-7	20	30	7	3UTR
mmu-miR-214-3p	NM_145582	Ctu1	ACAGCAGGCACAGACAGGCAGT#CTGCCTCAGTTCCTGCTGA#.(((((((.((....((((((.#))))))..)).))))))).	2381	2400	0.84615	-23	1	1.85E-05	0.5	0.2	0.16	-11	15	19	7	3UTR
mmu-miR-214-3p	NM_145582	Ctu1	ACAGCAGGCACAGACAGGCAGT#GCTCCTGCTTCTGTGCTGCC#...(((.(((((((((((.(((#))))))))..))))))))))).	159	179	0.84615	-22	0	5.40E-05	0.4	2.5	1.81	-8	17	20	10	CDS
mmu-miR-214-3p	NM_145587	Sbk1	ACAGCAGGCACAGACAGGCAGT#CTGCCTGAGTCCAGTTTACAAACTGTGGTTGCTC#..(((((.(((((.(((((((.#))))))).............))))).))))).	3096	3130	0.84615	-24	0	5.49E-05	0.4	0.3	0.62	-8	17	34	7	3UTR
mmu-miR-214-3p	NM_145587	Sbk1	ACAGCAGGCACAGACAGGCAGT#CTGCGTCTGAGCCGCTGC#.((((.(((.(((((..((((.#))))))))).))))))).	1855	1873	1	-23	0	6.50E-06	0.3	3.6	1.25	-7	16	18	9	CDS
mmu-miR-214-3p	NM_145604	D230025D16Rik	ACAGCAGGCACAGACAGGCAGT#ACTGGCCTGGCCTGGCCTGGCTA#..(((((((.((((.((((((((#)))).))))..)))))))).)).	2303	2326	1	-24	1	1.48E-06	0.4	0.4	0.8	-7	18	23	8	3UTR
mmu-miR-214-3p	NM_145604	D230025D16Rik	ACAGCAGGCACAGACAGGCAGT#TCTGTCCTAAGCCTGCTGTT#(((((((((...((((((....#))))))....))))))))).	1684	1704	0.84615	-24	1	5.19E-06	0.5	0.7	1.01	-11	15	20	9	3UTR
mmu-miR-214-3p	NM_145604	D230025D16Rik	ACAGCAGGCACAGACAGGCAGT#CTGCCCCAGCTGTCTGTATGGTTCCTGGGCCTACAGCCAGGCCTGTGTGG#.((((((((((((((((((((.#)))))).....)))))))......................)))	2623	2673	0.92308	-24	1	1.98E-06	0.4	0.5	1.57	-4	20	50	7	3UTR

mmu-miR-214-3p	NM_145732	Ctnna2	ACAGCAGGCACAGACAGGCAGT#GCTGACTTGTCTGCCTCTGG#.(((.((((..((((((((((((#)))).))))))))))))))).	3159	3179	1	-23	0	0.0017291	0.6	0	0	-12	18	20	14	3UTR
mmu-miR-214-3p	NM_145742	Dhx35	ACAGCAGGCACAGACAGGCAGT#GCTCTCTTGGCCTCCTGTGTCTTGCCTGCTGTG#((((((((((.((((((((...#.)))).....))))))))))))	2475	2508	0.92308	-28	1	0.0016528	0.4	0.2	-0.3	-7	18	25	14	3UTR
mmu-miR-214-3p	NM_145851	Cables2	ACAGCAGGCACAGACAGGCAGT#GCTCAAAGGATAGTAAATACGTTTTAGCCTGTCCATGCTTATGCTGG#.(((((((((..((((((((...#.))))))))..)))))..))))).	2412	2459	0.84615	-26	1	1.34E-06	0.6	2.5	3.35	-10	16	22	7	3UTR
mmu-miR-214-3p	NM_145853	Tpcn1	ACAGCAGGCACAGACAGGCAGT#GCTGTCAACGGTGTCTGGATCCTGGTGG#.((.((((..((((((((((((#)))))).....))))))...)))).)).	1599	1627	0.84615	-24	0	0.0009898	0.5	-0	-0.3	-9	18	28	6	CDS
mmu-miR-214-3p	NM_145853	Tpcn1	ACAGCAGGCACAGACAGGCAGT#GCCAACACAGGTGTCTGTGTCTGGTA#..(.(((((((((((((((...#))).........)))))))))))).).	3077	3103	1	-24	0	6.73E-05	0.5	3	3.84	-8	16	26	12	3UTR
mmu-miR-214-3p	NM_145853	Tpcn1	ACAGCAGGCACAGACAGGCAGT#GCTCCTGCTGCTGCTGTCGCTGTG#(((((.((((((.((((.(((#))))))))))).))))...))))).	596	620	0.84615	-23	0	9.11E-05	0.3	-1	-0.6	-7	19	24	10	CDS
mmu-miR-214-3p	NM_145929	Gga1	ACAGCAGGCACAGACAGGCAGT#TTGCCACTCGCTTGCTGG#.((((((((...((..(((((.#)))))..)))))))))).	521	539	0.84615	-20	1	0.0001548	0.4	3.1	3.83	-12	15	18	10	CDS
mmu-miR-214-3p	NM_145951	Enox2	ACAGCAGGCACAGACAGGCAGT#GCTGCAATGACGCTGCCTGTGTCTGATCCAGCTGC#.((((.(((..((((((((((((#.))))))))..)))))	322	357	1	-27	0	0.0031637	0.5	0.6	0.66	-8	18	25	7	5UTR
mmu-miR-214-3p	NM_145951	Enox2	ACAGCAGGCACAGACAGGCAGT#ACTGTCTGTTGGTCCCATTTTTTCCTGCTGTT#((((((((.(((.((((((((((#)))))))))).)).........))	2973	3005	0.84615	-25	1	0.0763281	0.6	2.3	3.79	-8	20	32	10	3UTR
mmu-miR-214-3p	NM_145958	Kbtbd2	ACAGCAGGCACAGACAGGCAGT#GCCTGTATAGGGGACTTGAGGGAGTGCCACGTGCTGA#.((((((((((..(((((((...#))))))).................)))))...))))).	2505	2542	0.84615	-23	1	0.001667	0.5	4.2	4.55	-8	16	37	6	CDS
mmu-miR-214-3p	NM_145958	Kbtbd2	ACAGCAGGCACAGACAGGCAGT#TGCAGAGAACTGTGTGCGGCTGCTGA#.((((((.(.((.((((.....#.)))).)).).)))))).	1029	1055	0.84615	-22	1	0.0045187	0.6	0.4	0.7	-9	13	18	6	CDS
mmu-miR-214-3p	NM_145965	Gphn	ACAGCAGGCACAGACAGGCAGT#GCTCCTGTCAGTGCGGTGACTGC#.(((((.(((((.((((((.(((#)))))))))).))))..)).))).	90	113	0.92308	-23	0	1.27E-05	0.4	1.9	2.45	-9	18	23	9	5UTR

mmu-miR-214-3p	NM_145965	Gphn	ACAGCAGGCACAGACAGGCAGT#GCTTTTAGTAGACAGCGGTAGGTGCCTGCA#...((((((((..((..((.((#.)).)).))..)))))))).	2721	2751	0.92308	-22	0	0.0002091	0.6	4.9	4.84	-11	14	20	8	3UTR
mmu-miR-214-3p	NM_146005	Ank3	ACAGCAGGCACAGACAGGCAGT#GCTGTCTAATCCCCAGTCACAGGGGCCCTGTCTGTGCCTCCA#...(.(((((((((((((....#.)))))))))))))).).	8011	8053	0.92308	-26	0	0.0015804	0.5	0.2	0.57	-11	14	17	13	3UTR
mmu-miR-214-3p	NM_146005	Ank3	ACAGCAGGCACAGACAGGCAGT#CTGTGTGTGTGTGTGTGTGTGTG#((((((.(((((.(((.((((.#)))).))).))))).))).))).	28	51	1	-24	0	0.4260992	0.5	0.5	1.05	-7	18	23	5	5UTR
mmu-miR-214-3p	NM_146118	Slc25a25	ACAGCAGGCACAGACAGGCAGT#GCTGCCCATGCTCAATGAGTGGCCTGCTGG#.((((((((.((..((((((((#)))))).........))..))))))))	3151	3181	0.84615	-27	1	5.25E-09	0.4	1.7	1.25	-7	18	30	10	3UTR
mmu-miR-214-3p	NM_146118	Slc25a25	ACAGCAGGCACAGACAGGCAGT#GCTGCCTGCATGGGCCTAACGTCCTCTGAGCTGC#.((((((((.((..((((((((#))))))))..)).)))))............)))).	2360	2394	0.84615	-26	1	0.0011327	0.4	4.6	3.21	-7	18	34	8	3UTR
mmu-miR-214-3p	NM_146118	Slc25a25	ACAGCAGGCACAGACAGGCAGT#GCTGCCTGACAGCGTGGTGCATGGCTTTGCTGA#.((((((((((...((((((((#))))))))........)))).....)))))).	2291	2324	0.92308	-22	1	2.47E-05	0.4	4	3.86	-9	18	33	8	3UTR
mmu-miR-214-3p	NM_146175	Zfp282	ACAGCAGGCACAGACAGGCAGT#GCTGCAGGAGTACGGCCTGCTGC#.((((((((.....(..(((((#)))))..)......)))))))).	587	610	0.84615	-23	1	4.09E-05	0.3	2.7	2.47	-14	14	23	8	CDS
mmu-miR-214-3p	NM_146175	Zfp282	ACAGCAGGCACAGACAGGCAGT#CTGCCTGCTTCTGTCTCTTTGAGTGCTGG#.(((((((.((((((((((((.#)))))))..))))))).......))))).	5165	5194	1	-24	1	9.22E-05	0.5	0.1	0.2	-5	19	29	7	3UTR
mmu-miR-214-3p	NM_146175	Zfp282	ACAGCAGGCACAGACAGGCAGT#TGTCTGTCTGTCTTGGGGTG#((..((((.(((((((((((..#)))))))))))))))..)).	4323	4343	1	-23	0	0.0132959	0.6	-0	-0	-10	17	20	15	3UTR
mmu-miR-214-3p	NM_146193	Btbd1	ACAGCAGGCACAGACAGGCAGT#GCTGCCGGACGTGGAGCCCGCCGCCTTCCTGGCGCTGCTGA#.((((((((.(((..((((.((#.)).))))..))))).))))))).	417	458	0.84615	-24	1	0.0017522	0.5	0.3	0.44	-7	17	23	6	CDS
mmu-miR-214-3p	NM_146193	Btbd1	ACAGCAGGCACAGACAGGCAGT#CTGCCCAATGTCTGTTACACAGCGTGCG#...(((.((((((((((((((.#)))))...)))))))......)).))).	1309	1337	0.84615	-24	0	0.0003744	0.5	0.3	0.92	-8	17	28	7	CDS

mmu-miR-214-3p	NM_146223	Cplx3	ACAGCAGGCACAGACAGGCAGT#TGCCCTGTGCCTGGAGTT#((..((((((((...((((..#)))))))))))))..)).	2636	2654	0.92308	-23	0	8.02E-05	0.5	1.7	2.71	-10	15	18	13	3UTR
mmu-miR-214-3p	NM_146223	Cplx3	ACAGCAGGCACAGACAGGCAGT#TTGTCTGCACACATGTCTTTGCTCATGTGTGCTTGGTGA#.((.((((((((.(..(((((.#.))))).).).)))))))).)).	2570	2609	0.84615	-22	0	4.51E-06	0.5	4.3	2.66	-9	16	22	8	3UTR
mmu-miR-214-3p	NM_146253	Zbtb6	ACAGCAGGCACAGACAGGCAGT#CTGCCTCTGTCCCCTGAGTGCTGG#.((((((.((((((..(((((.#)))))))))))).)).....))))).	3450	3474	0.84615	-22	1	2.04E-05	0.4	2.4	2.91	-6	17	24	10	3UTR
mmu-miR-214-3p	NM_146260	Tmie	ACAGCAGGCACAGACAGGCAGT#ACCACCGTCTCCGCCTGCTGG#.((((((((..((((.(((..((#))..)))))))..)))))))).	1043	1064	1	-23	1	6.06E-05	0.3	-0	0.01	-8	16	21	8	5UTR
mmu-miR-214-3p	NM_147779	Sftpb	ACAGCAGGCACAGACAGGCAGT#GCTGCTTGTGCCCCGGTGTCGCCAAGTGCTTGATGTC#(((.(((((((..(((((((((#)))))))))...............))))))).))).	329	366	0.84615	-22	0	0.0079746	0.5	1	0.27	-12	19	37	9	CDS
mmu-miR-214-3p	NM_148926	Zfand3	ACAGCAGGCACAGACAGGCAGT#GCTGCCCACGTCTGGCTGCTGA#.(((((.((.(((((.((((((#))))))...))))))))))))).	1976	1998	0.84615	-27	0	1.37E-05	0.4	0.9	0.27	-7	18	22	12	3UTR
mmu-miR-214-3p	NM_148926	Zfand3	ACAGCAGGCACAGACAGGCAGT#CTGGCTGGTTACTAAGGTGCCTGCTA#..(((((((((...(((.(((.#))).))).........))))))))).	997	1023	0.84615	-24	1	0.0002362	0.7	3.6	3.31	-10	15	26	9	3UTR
mmu-miR-214-3p	NM_148926	Zfand3	ACAGCAGGCACAGACAGGCAGT#ACATGCCTGTCACTGCTGG#.((((((.....((((((((((#)).)))))))).)))))).	1089	1108	0.92308	-23	1	0.0008343	0.5	2.5	2.96	-15	16	19	8	3UTR
mmu-miR-214-3p	NM_148948	Dicer1	ACAGCAGGCACAGACAGGCAGT#ACTGCAGAGCTGTGCCAGCA#...((.(((((((....(((((#)))))....))))))).)).	9738	9758	1	-24	0	2.51E-05	0.5	1.7	1.44	-10	14	20	7	3UTR
mmu-miR-214-3p	NM_148953	Asb16	ACAGCAGGCACAGACAGGCAGT#GCTGCGGAGGTCTGGCCGAGCTGC#.((((.(((.(((((..(((((#)))))....))))))))..)))).	955	979	0.84615	-27	0	4.29E-05	0.3	2.7	1.97	-7	17	24	8	CDS
mmu-miR-214-3p	NM_148953	Asb16	ACAGCAGGCACAGACAGGCAGT#ACTGCGTGCTCTCCGCCTGCA#...((((((..(((((.(((((#))))).)).)))..)))))).	107	128	0.84615	-25	0	2.14E-05	0.4	1.4	1.57	-11	16	21	6	CDS
mmu-miR-214-3p	NM_148953	Asb16	ACAGCAGGCACAGACAGGCAGT#CTGCCTCCTGGCCTGTGTGTT#(((((((((.(((..((((((.#)))))).))))))))).))).	1867	1888	0.84615	-24	1	0.000186	0.4	0.1	-0.1	-7	18	21	9	3UTR

mmu-miR-214-3p	NM_152804	Plk2	ACAGCAGGCACAGACAGGCAGT#ATTCCTCTGTGGCCTGCTGG#.((((((((((((........#.)))))).))))))).	2319	2339	0.92308	-23	1	9.81E-05	0.5	0.8	1.36	-5	13	16	7	3UTR
mmu-miR-214-3p	NM_152807	Ccdc137	ACAGCAGGCACAGACAGGCAGT#TGCCTATATGTGCCTGTG#...(((((((((...(((((..#)))))...))))))))).	2079	2097	0.92308	-25	0	0.0056059	0.5	0	0.01	-9	14	18	9	3UTR
mmu-miR-214-3p	NM_152807	Ccdc137	ACAGCAGGCACAGACAGGCAGT#CTGCCCACTCCAAGGTCAGGTCTGGTCTCTGCTGC#.(((((((..(((((.(((((.#)))))............)))))..).))))))).	2335	2370	0.84615	-23	1	0.0003202	0.4	0.2	0.14	-6	17	35	6	3UTR
mmu-miR-214-3p	NM_153057	Nomo1	ACAGCAGGCACAGACAGGCAGT#CTGCCTGTCCAAGCCTGGTGTG#(((.((((((...((((((((((.#))))))))))...))))).))).	1989	2011	0.92308	-31	0	0.0005824	0.4	4.4	4.13	-14	17	22	9	CDS
mmu-miR-214-3p	NM_153057	Nomo1	ACAGCAGGCACAGACAGGCAGT#CTGCCTGTCTGTCCTCTCCTGTG#((((.((((.((((((((((((.#)))))))))))))))...)))).	3903	3926	0.92308	-30	0	0.1454775	0.4	-1	-0.1	-9	19	23	15	3UTR
mmu-miR-214-3p	NM_153057	Nomo1	ACAGCAGGCACAGACAGGCAGT#GTCTGCTGTGCCTTCTGG#.(((.((((((((.((((((...#)))))))))))))).))).	4115	4133	0.94872	-27	0	0.0002649	0.4	-0	-0.1	-9	16	18	13	3UTR
mmu-miR-214-3p	NM_153060	Spns2	ACAGCAGGCACAGACAGGCAGT#GCCTGGGCTATGCCCTCATGCTGTG#((((((((((.((.((((((...#))))))..)).))))....)))))).	1623	1648	0.84615	-24	1	0.000855	0.4	3.9	3.3	-8	17	25	6	CDS
mmu-miR-214-3p	NM_153060	Spns2	ACAGCAGGCACAGACAGGCAGT#CTGCCTCATCTTTGTGGCTGCC#...((((.(((((((.((((((.#))))))....)))))).)))).	1357	1379	1	-24	0	0.0006668	0.5	1.1	0.34	-10	16	22	6	CDS
mmu-miR-214-3p	NM_153079	Nmur2	ACAGCAGGCACAGACAGGCAGT#GCTGTTTGTCTTGGTCCTCGTGTTTGCTA#..((((((((((((((((((((#)))))))))))........))))))))).	886	915	0.92308	-25	1	0.0031826	0.5	-0	-0	-5	20	29	11	CDS
mmu-miR-214-3p	NM_153079	Nmur2	ACAGCAGGCACAGACAGGCAGT#GCTGGCGCTGTGCCTACTCGTTGC#.((((((((((((.(.(.((((#)))).))))))))))....)))).	1876	1900	1	-22	1	8.19E-05	0.5	0.3	0.05	-5	18	24	10	3UTR
mmu-miR-214-3p	NM_153079	Nmur2	ACAGCAGGCACAGACAGGCAGT#GCCTAGTCTGGAGCTTCTCTGTG#((((.(((((.((((((((((...#)))).))))))..))))..)))).	582	605	0.84615	-22	0	0.0003469	0.5	0.8	0.13	-9	17	23	5	CDS
mmu-miR-214-3p	NM_153083	Thtpa	ACAGCAGGCACAGACAGGCAGT#ACTGCTCGGGTCTGGGGAGCAGAAGCCTGCAGGTGTGGCTGCTGTC#(((((((.((((..(((((...#.)))))....)))).))))))).	957	1003	0.92308	-28	1	3.76E-06	0.3	-0	0.8	-8	16	23	7	CDS

mmu-miR-214-3p	NM_153088	Ctdsp1	ACAGCAGGCACAGACAGGCAGT#AGTGCCTTCTCTGTGACCTGA#....((((((((((.(((((.(#).)))))..)))))).)))).	2420	2441	0.92308	-23	0	0.0080959	0.6	0.3	0.34	-10	16	21	6	3UTR
mmu-miR-214-3p	NM_153088	Ctdsp1	ACAGCAGGCACAGACAGGCAGT#TCTGCTGTGTCTGCCGG#.(.((((((((((.((((....#))))))))))))))).).	416	433	0.92308	-22	0	0.0011608	0.3	3.8	1.95	-6	15	17	14	CDS
mmu-miR-214-3p	NM_153088	Ctdsp1	ACAGCAGGCACAGACAGGCAGT#CTGCCTGCTGCTCCCCACTTCTGTCTTATCCACGCTGC#.((((((((((((.(((((((.#))))))))))...........))))).......)))).	1299	1337	1	-22	1	0.2222535	0.5	-0	0.29	-5	19	38	10	3UTR
mmu-miR-214-3p	NM_153123	Atf7ip2	ACAGCAGGCACAGACAGGCAGT#CTGTGGTGGCTATGGAATTGCTGCTGTGTCTGA#....(((((((((.((.(((((#.)))))))))))))))).	1742	1775	0.84615	-21	0	0.0238403	0.5	-0	-0.1	-10	16	18	16	3UTR
mmu-miR-214-3p	NM_153178	Ago2	ACAGCAGGCACAGACAGGCAGT#GCTGCTCTGTGCTCTGTGGGTTTTTTCTACCCCTGCTGA#.(((((((((((((((((((((#))))).)))...))))))............))))))).	4339	4378	0.84615	-27	1	0.0003563	0.4	1.2	1.09	-4	21	39	7	3UTR
mmu-miR-214-3p	NM_153178	Ago2	ACAGCAGGCACAGACAGGCAGT#CTGCCTCGTCCATGTGTGTCGCTC#..(((.(((((((((((((((.#)))))).)))....)))))))))).	3857	3881	0.96154	-23	0	0.0074009	0.5	0.5	-0.3	-7	18	24	9	3UTR
mmu-miR-214-3p	NM_153178	Ago2	ACAGCAGGCACAGACAGGCAGT#CTGCCTGGCAAAACTCCTGTGTATGCG#...(((.((((((.(((((((.#)))))))).........)))))).))).	1731	1758	0.84615	-22	0	0.0018638	0.4	1.7	0.53	-9	16	27	7	CDS
mmu-miR-214-3p	NM_153198	Hbp1	ACAGCAGGCACAGACAGGCAGT#CTGTGCTGTCAGTGTGTCTCTGG#.(((.((((((((((((((((.#)))).)))))..))))))))))).	2370	2393	1	-23	0	1.56E-05	0.5	2.4	3.55	-8	19	23	10	3UTR
mmu-miR-214-3p	NM_153387	Tubgcp4	ACAGCAGGCACAGACAGGCAGT#CTGCCACATTCTGATGTCTGTAACAAATCTCTCAAATCATTCATCAAATGCCTGCA#...(((((((((((((((((((.#)))))).........)))))))...........................)))))).	2262	2318	0.84615	-25	0	0.0046998	0.6	-0	0.99	-7	18	56	7	3UTR
mmu-miR-214-3p	NM_153387	Tubgcp4	ACAGCAGGCACAGACAGGCAGT#TTGTTTGCTGTACTGCTAAAGTGACAAGCCTGCTGA#.(((((((((((((.(((((((.#))))))))))))...............)))))))).	3625	3661	1	-24	1	0.001646	0.6	0.3	0.37	-5	19	36	11	3UTR

mmu-miR-214-3p	NM_153394	Gpr156	ACAGCAGGCACAGACAGGCAGT#ACTGTGAACTGCTGTGTCTTCATTCCCCAGCTGA#.((((((((((((.((((((((#)))))...))))))))))))..........))))).	1379	1413	1	-22	1	0.0025168	0.5	3.9	2.69	-5	20	34	11	CDS
mmu-miR-214-3p	NM_153423	Wasf2	ACAGCAGGCACAGACAGGCAGT#GCCAGCCTGCTGCACCACCACCACCACCACCTTCTGAGGCCACCAAGCCCAAGTCTTCATTGCCTGCTGTG#((((((((((.((((.((((...#.)))...))))....)))))))))).	1363	1434	1	-26	1	0.002472	0.4	0.6	1.35	-7	17	26	10	CDS
mmu-miR-214-3p	NM_153489	Ubap2l	ACAGCAGGCACAGACAGGCAGT#CTTCCAGTATGGGCCTGCTGTG#(((((((((.((.((.((.((.#)).)).)).)).))))))))).	2899	2921	0.92308	-23	1	5.14E-06	0.4	3.4	3	-7	17	22	9	CDS
mmu-miR-214-3p	NM_153514	Rhobtb2	ACAGCAGGCACAGACAGGCAGT#GCTGCCAGCTGGACCTGCG#...(((((..((((.(.(((((((#)))))).)))))..))))).	934	953	0.92308	-25	0	0.0002355	0.4	-1	-0.2	-10	15	19	6	CDS
mmu-miR-214-3p	NM_153514	Rhobtb2	ACAGCAGGCACAGACAGGCAGT#ACTCGGGCTTGTCTGTATTGGTGTCTGCTT#..(((((((((..(((((((((#.))).))))))....))))))))).	3025	3055	0.92308	-25	1	0.0119833	0.4	1.8	2.93	-10	18	25	9	3UTR
mmu-miR-214-3p	NM_153515	Ammecr1l	ACAGCAGGCACAGACAGGCAGT#ACTGCCTCCGGTGCCTGCTGG#.((((((((((....(((((((#)))))))...)))))))))).	91	112	1	-32	1	7.66E-06	0.4	1.7	1.43	-10	17	21	10	5UTR
mmu-miR-214-3p	NM_153541	Zbtb8b	ACAGCAGGCACAGACAGGCAGT#TGCCTGTCAACCTTAGCCTGGTGG#.((.((((((...((((((((..#))))))))).......))))).)).	1367	1391	0.82051	-25	0	0.0001994	0.5	-0	0.64	-14	15	24	8	CDS
mmu-miR-214-3p	NM_153541	Zbtb8b	ACAGCAGGCACAGACAGGCAGT#GCCGCTGTGGCTGCC#...((((.(((((.(.(((...#))))))))).))))).	478	493	0.84615	-22	0	1.55E-06	0.3	-0	0.07	-10	13	15	9	CDS
mmu-miR-214-3p	NM_153541	Zbtb8b	ACAGCAGGCACAGACAGGCAGT#TTGGTTGCTGTGGAACCTGCTT#..((((((((((.((((.((((.#))).))))))))...)))))).	2829	2851	0.92308	-21	1	0.0070499	0.4	3.2	2.69	-6	17	22	8	3UTR
mmu-miR-214-3p	NM_153557	Paxx	ACAGCAGGCACAGACAGGCAGT#CCTGTGGTCCTGCTGTT#((((((((.((..(((((....#))))).)))))))))).	895	912	1	-22	1	0.0013533	0.4	-1	-0.3	-10	15	17	10	3UTR
mmu-miR-214-3p	NM_153582	Cmtm4	ACAGCAGGCACAGACAGGCAGT#GCAAAGCTGTCTGTGTATGCTGA#.(((((.(((((((((((.....#.)))))))))).))))).	6094	6117	1	-26	0	0.0016747	0.5	0.4	0.42	-5	15	18	10	3UTR

mmu-miR-214-3p	NM_153588	Mrtfb	ACAGCAGGCACAGACAGGCAGT#GCTTGTAACTGCCTGCTC#..((((((((...((((((...#))))))...)))))))).	4421	4439	1	-24	1	0.0056026	0.6	1.3	0.44	-12	14	18	8	3UTR
mmu-miR-214-3p	NM_153588	Mrtfb	ACAGCAGGCACAGACAGGCAGT#ACTGTGTCCTCAGCGAAGTCTGGCCCAATGCTGG#.((((((((.((((((.(((((#))))).)..........))))))))...))))).	977	1011	0.84615	-22	1	6.44E-05	0.5	2.7	2.34	-5	19	34	8	CDS
mmu-miR-214-3p	NM_170687	Ank3	ACAGCAGGCACAGACAGGCAGT#GCTGTCTAATCCCCAGTCACAGGGGCCCTGTCTGTGCCTCCA#...(.(((((((((((((....#.)))))))))))).).	5540	5582	0.92308	-26	0	0.0015804	0.5	0.2	0.57	-11	14	17	13	3UTR
mmu-miR-214-3p	NM_170687	Ank3	ACAGCAGGCACAGACAGGCAGT#CCTGTGTGCCTGTCGG#.(.((((((((..(((((....#)))))))))))).).	3759	3775	1	-21	0	1.19E-05	0.5	3.1	2.09	-10	14	16	13	CDS
mmu-miR-214-3p	NM_170689	Ank3	ACAGCAGGCACAGACAGGCAGT#GCTGTCTAATCCCCAGTCACAGGGGCCCTGTCTGTGCCTCCA#...(.(((((((((((((....#.)))))))))))).).	7957	7999	0.92308	-26	0	0.0015804	0.5	0.2	0.57	-11	14	17	13	3UTR
mmu-miR-214-3p	NM_170689	Ank3	ACAGCAGGCACAGACAGGCAGT#CTGTGTGTGTGTGTGTGTGTGTG#((((((.((((((.(((.((((.#)))).))).))))).))).))).	28	51	1	-24	0	0.4260992	0.5	0.5	1.05	-7	18	23	5	5UTR
mmu-miR-214-3p	NM_170690	Ank3	ACAGCAGGCACAGACAGGCAGT#GCTGTCTAATCCCCAGTCACAGGGGCCCTGTCTGTGCCTCCA#...(.(((((((((((((....#.)))))))))))).).	7948	7990	0.92308	-26	0	0.0015804	0.5	0.2	0.57	-11	14	17	13	3UTR
mmu-miR-214-3p	NM_170690	Ank3	ACAGCAGGCACAGACAGGCAGT#CTGTGTGTGTGTGTGTGTGTGTG#((((((.((((((.(((.((((.#)))).))).))))).))).))).	28	51	1	-24	0	0.4260992	0.5	0.5	1.05	-7	18	23	5	5UTR
mmu-miR-214-3p	NM_170728	Ank3	ACAGCAGGCACAGACAGGCAGT#GCTGTCTAATCCCCAGTCACAGGGGCCCTGTCTGTGCCTCCA#...(.(((((((((((((....#.)))))))))))).).	7306	7348	0.92308	-26	0	0.0015804	0.5	0.2	0.57	-11	14	17	13	3UTR
mmu-miR-214-3p	NM_170728	Ank3	ACAGCAGGCACAGACAGGCAGT#CTGTGTGTGTGTGTGTGTGTGTG#((((((.((((((.(((.((((.#)))).))).))))).))).))).	28	51	1	-24	0	0.4260992	0.5	0.5	1.05	-7	18	23	5	5UTR

mmu-miR-214-3p	NM_170729	Ank3	ACAGCAGGCACAGACAGGCAGT#GCTGTCTAATCCCCAGTCACAGGGGCCCTGTCTGTGCCTCCA#...(.((((((((((((....#.))))))))))))).).	7894	7936	0.92308	-26	0	0.0015804	0.5	0.2	0.57	-11	14	17	13	3UTR
mmu-miR-214-3p	NM_170729	Ank3	ACAGCAGGCACAGACAGGCAGT#CTGTGTGTGTGTGTGTGTGTGTG#((((((.(((((.(((.((((.#)))).))).))))).))).))).	28	51	1	-24	0	0.4260992	0.5	0.5	1.05	-7	18	23	5	5UTR
mmu-miR-214-3p	NM_170730	Ank3	ACAGCAGGCACAGACAGGCAGT#GCTGTCTAATCCCCAGTCACAGGGGCCCTGTCTGTGCCTCCA#...(.((((((((((((....#.))))))))))))).).	4642	4684	0.92308	-26	0	0.0015804	0.5	0.2	0.57	-11	14	17	13	3UTR
mmu-miR-214-3p	NM_170730	Ank3	ACAGCAGGCACAGACAGGCAGT#CTGTGTGTGTGTGTGTGTGTGTG#((((((.(((((.(((.((((.#)))).))).))))).))).))).	28	51	1	-24	0	0.4260992	0.5	0.5	1.05	-7	18	23	5	5UTR
mmu-miR-214-3p	NM_170730	Ank3	ACAGCAGGCACAGACAGGCAGT#TGTGTGTGTGTGTGTGCA#...(((.(((((.(((.(((..#))).))).))))).))).	61	79	0.84615	-23	0	0.0041616	0.4	0.4	0.72	-9	14	18	5	5UTR
mmu-miR-214-3p	NM_170755	Retreg2	ACAGCAGGCACAGACAGGCAGT#GCTGCCTGTGCTGTGCTGGCTGTG#(((((.(((((((((((((((#)))))))))).))))))).))))).	635	659	1	-37	0	0.0029183	0.4	1.9	3.8	-6	21	24	9	CDS
mmu-miR-214-3p	NM_170755	Retreg2	ACAGCAGGCACAGACAGGCAGT#CTGCCTGCCTGCCTGCCTGCTGTC#(((((((((((((.(((((((.#))))))).)))..))))))))))).	1841	1865	0.92308	-34	1	0.0011505	0.4	1.2	0.81	-5	20	24	10	3UTR
mmu-miR-214-3p	NM_172119	Dio3	ACAGCAGGCACAGACAGGCAGT#ACTGACAGCTTTGTCCCTGCTGG#.((((((((((((...(((((#)))).)...))))).))))))).	1139	1162	0.84615	-21	1	2.00E-05	0.5	-0	-0.3	-6	17	23	7	3UTR
mmu-miR-214-3p	NM_172286	6430548M08Rik	ACAGCAGGCACAGACAGGCAGT#CCTGCCTGTGTCTGCTC#..(((((((((((.((((....#)))).))))))))))).	1651	1668	1	-26	1	0.0651706	0.4	0.6	0.26	-6	15	17	11	3UTR
mmu-miR-214-3p	NM_172294	Sulf1	ACAGCAGGCACAGACAGGCAGT#GCTGCTTGCCGTGCTGGGCACACAGCTGCTGG#.((((((((((.(.((((((((#))))))))).))))......	489	521	0.84615	-26	1	1.29E-08	0.3	1.5	1.46	-8	19	32	9	CDS
mmu-miR-214-3p	NM_172294	Sulf1	ACAGCAGGCACAGACAGGCAGT#TCTTTCTGTGCCTGCA#...(((((((((((.(((....#))).))))))))))).	2752	2768	1	-24	0	0.035048	0.5	5.1	4.23	-7	14	16	11	CDS
mmu-miR-214-3p	NM_172415	Arhgef10l	ACAGCAGGCACAGACAGGCAGT#GGTGCCCCATCCTGGCCTGCTGC#.((((((((.(((...((((.(#).))))).....))))))))))).	2480	2503	0.92308	-25	1	0.0002516	0.3	0.4	1.58	-7	16	23	11	CDS

mmu-miR-214-3p	NM_172418	Mamstr	ACAGCAGGCACAGACAGGCAGT#GCTGCCTCCCTCAGAGACCTGCAGGTTGTGTGGCTGCCTGTC#(((((((.(((((((..((((.#.))))..))).)))).)))).))).	1607	1649	0.84615	-24	1	4.10E-05	0.5	0.2	0.25	-6	18	25	4	3UTR
mmu-miR-214-3p	NM_172418	Mamstr	ACAGCAGGCACAGACAGGCAGT#GCTGCCTCCCGTGCCCTTGGACTTCCCTGGCTCATTTGACCTGCTGTC#((((((((((.(..(((((((#))))))))..))))))........................)))))).	1120	1168	0.84615	-24	1	0.0353532	0.4	1.5	1.33	-9	19	48	7	CDS
mmu-miR-214-3p	NM_172444	Thsd4	ACAGCAGGCACAGACAGGCAGT#CTGCCTGTCCCTCTCACTGTGCCTCTGC#.(((.(((((((((((((((.#)))))))))........)))))))))).	3888	3916	0.84615	-30	0	0.0770473	0.4	-0	-0.2	-8	19	28	10	3UTR
mmu-miR-214-3p	NM_172493	Diaph2	ACAGCAGGCACAGACAGGCAGT#TGTGTGTGTGTGTTTGTGTGTG#(((((((((((.(((.(((..#))).))).))))))))).))).	4763	4785	1	-22	1	0.3357614	0.6	0.7	0.49	-5	18	22	9	3UTR
mmu-miR-214-3p	NM_172493	Diaph2	ACAGCAGGCACAGACAGGCAGT#TGTGTGTGTGTGTGTGTGTGTG#((((((.(((((.(((.(((..#))).))).))))).))).))).	4731	4753	0.84615	-22	0	0.1954812	0.6	0.2	0.53	-7	17	22	5	3UTR
mmu-miR-214-3p	NM_172499	Mfsd9	ACAGCAGGCACAGACAGGCAGT#CCTGTCTGCTGCTGG#.((((((...((((((((....#)))))))))))))).	1095	1110	1	-23	1	9.69E-05	0.4	0.1	0.1	-8	14	15	14	CDS
mmu-miR-214-3p	NM_172507	Sh3bgrl2	ACAGCAGGCACAGACAGGCAGT#CTGCCTGTCTGCCTGACTT#..(((((((..((((((((((.#))))))))))))))).)).	2702	2721	1	-28	1	0.0036852	0.5	-0	-0.2	-9	17	19	15	3UTR
mmu-miR-214-3p	NM_172507	Sh3bgrl2	ACAGCAGGCACAGACAGGCAGT#ACTGCTCTGGTACTTTGTAATAACGCCTGCTGG#.((((((((((((((((((((#))))).)))....))))).....)))))))).	947	980	0.92308	-26	1	0.0033104	0.5	0.4	0.32	-4	21	33	8	3UTR
mmu-miR-214-3p	NM_172507	Sh3bgrl2	ACAGCAGGCACAGACAGGCAGT#CTGCCTGCTTGTGGACAAAGGAAGTATGCTGCTGA#.((((((.(((((.((((((((.#))))))).))))).............))))))).	2959	2994	1	-24	1	0.0001796	0.5	0.2	0.63	-7	18	35	7	3UTR
mmu-miR-214-3p	NM_172507	Sh3bgrl2	ACAGCAGGCACAGACAGGCAGT#TTGCCAGTGTGTGCCA#......((((((.((.(((((.#))))).)).)))))).	2327	2343	0.92308	-22	0	0.0008695	0.6	-0	-0	-17	13	16	6	3UTR
mmu-miR-214-3p	NM_172528	Lrrc1	ACAGCAGGCACAGACAGGCAGT#ATTGACCTCCGAGTTTTACCTGCTGTGTCTTCCCTGCTGTA#((((((((...((((((((((.#.))))).)))))..)))))))).	1453	1494	1	-26	1	0.0067684	0.5	3.4	3.44	-9	18	23	8	3UTR

mmu-miR-214-3p	NM_172528	Lrrc1	ACAGCAGGCACAGACAGGCAGT#GCTCGGGTGTGTGCAGCATCAGGCTGTGCCTGCTC#..(((((((((((....((.((#.)).))......)))	2274	2309	0.84615	-24	1	4.87E-08	0.4	2.4	3.72	-7	15	24	11	3UTR
mmu-miR-214-3p	NM_172536	Zfp609	ACAGCAGGCACAGACAGGCAGT#GCCCCTGGTCTGCTGC#.((((((((.(((...(((...#))).))))))))))).	4804	4820	0.97436	-23	1	0.0012756	0.4	3.3	2.84	-7	14	16	11	3UTR
mmu-miR-214-3p	NM_172536	Zfp609	ACAGCAGGCACAGACAGGCAGT#GCTACCGGGGGATGCCTGCTGTG#((((((((((......((.(((#))).))......)))))))))).	3959	3982	0.84615	-23	1	1.56E-07	0.4	1.9	1.49	-13	15	23	10	CDS
mmu-miR-214-3p	NM_172538	Vezt	ACAGCAGGCACAGACAGGCAGT#ACCCTGAGATTTGCCTGTCTCTGCCTGCC#...(((((((.((((((((((.#.)))))))))).))))))).	6576	6605	0.88462	-32	0	2.77E-06	0.5	-0	-0.1	-9	17	20	10	3UTR
mmu-miR-214-3p	NM_172538	Vezt	ACAGCAGGCACAGACAGGCAGT#GCCGTGTTGTGTGCCTGCC#...(((((((((((((((...#))).)))).))))))))).	5546	5565	0.81538	-27	0	0.004952	0.5	1.1	1.11	-7	16	19	9	3UTR
mmu-miR-214-3p	NM_172544	Nrxn3	ACAGCAGGCACAGACAGGCAGT#GCTGCCTGGCCTCTGTGGACTTGAATGGACGCCTGCCTGA#.((((((((((((((((((((#))))))))...)))))..............)))))).)).	2620	2660	0.92308	-28	1	0.0067673	0.4	4.3	3.87	-4	21	40	8	CDS
mmu-miR-214-3p	NM_172584	Itpk1	ACAGCAGGCACAGACAGGCAGT#GCATGTCTGTCTTCATGGCTGTTGTG#(((((((.((.(((((((((((#)).)))))))))...)).))))))).	1662	1688	0.84615	-26	1	0.0024517	0.4	-0	2.24	-9	20	26	9	3UTR
mmu-miR-214-3p	NM_172584	Itpk1	ACAGCAGGCACAGACAGGCAGT#GGTGCGTGTGTGTGTGTGTGTGTG#((((((.(((((.(((.(((.(#).))).))).))))).))).))).	1595	1619	1	-24	0	0.2185143	0.5	2.1	2.32	-7	18	24	5	3UTR
mmu-miR-214-3p	NM_172584	Itpk1	ACAGCAGGCACAGACAGGCAGT#GCCGCCTGACTCCTGCTGTG#((((((((...((.(((((.((#)).))))).)))))))))).	1509	1529	1	-23	1	0.0009335	0.4	0.9	1.46	-10	17	20	10	3UTR
mmu-miR-214-3p	NM_172596	Sec24c	ACAGCAGGCACAGACAGGCAGT#CTGATGTAGCTGACATGTTTGTGCCACTGCTGG#.((((((((((((((.(((((#.)))).).))))))))))).	1822	1855	0.92308	-28	1	0.0028797	0.5	4.6	4.1	-4	20	26	10	CDS
mmu-miR-214-3p	NM_172596	Sec24c	ACAGCAGGCACAGACAGGCAGT#TCTGTCATGTCCTGCTGA#.(((((((.(((((((((....#)))))).)))))))))).	3567	3585	1	-24	1	0.0162169	0.5	1.4	1.28	-5	16	18	10	3UTR
mmu-miR-214-3p	NM_172600	Tmem260	ACAGCAGGCACAGACAGGCAGT#GCTGTCTGTCTCTCCACACACTCCAGTGCCCTGCTGC#.((((((((((((((((((((#)))))))))))..............))))).))))).	4902	4939	0.92308	-31	1	0.0011408	0.4	0.5	0.77	-4	21	37	11	3UTR

mmu-miR-214-3p	NM_172600	Tmem260	ACAGCAGGCACAGACAGGCAGT#CTGCCTGTCTCGTCCTACCTCAGTCGCGCCCGCTGG#.((((.((((((((((((((.#)))))))))).)).............))).)))).	797	833	0.92308	-28	0	4.30E-05	0.3	2.6	2.96	-6	19	36	10	CDS
mmu-miR-214-3p	NM_172600	Tmem260	ACAGCAGGCACAGACAGGCAGT#GCTGCTGAGGCTGACTCTGGCCTTCTCTGCTGGCCTGCTGC#.((((((((.(((.((((((((#)))))....................)))))))))))))).	745	786	0.92308	-27	1	0.0002412	0.4	1.2	2.58	-6	19	41	14	CDS
mmu-miR-214-3p	NM_172604	Scara3	ACAGCAGGCACAGACAGGCAGT#GCTGGTTAGAAGGAAAGGCTGTGAGAGCCTGCTGG#.((((((((.(..((((.(...#.).))))..).)))	2289	2324	1	-25	1	1.10E-05	0.4	1.8	1.95	-10	14	20	8	3UTR
mmu-miR-214-3p	NM_172606	Marchf6	ACAGCAGGCACAGACAGGCAGT#ACTGCCTGATTGTTTTCATGTGTCTGTGTTTGTA#...((((((((((((((((((#)))))))).............)	3527	3561	0.89744	-27	0	0.0348019	0.7	2.7	3.07	-7	19	34	12	3UTR
mmu-miR-214-3p	NM_172606	Marchf6	ACAGCAGGCACAGACAGGCAGT#GCTTGTGCGTGCATGGACTGTTACTGCTGG#.((((((((((..((((((...#))))))..)))).........)))))	1868	1898	0.84615	-23	1	0.0246561	0.5	0.1	0.44	-8	16	30	6	CDS
mmu-miR-214-3p	NM_172608	Tmem184b	ACAGCAGGCACAGACAGGCAGT#GCTCTGTGGACCCTCCCCCTCTCCCAACCTAGGCCTGTCTGGCATGCTGC#.(((((.((.((((((((((...#.))))))))))).))))).	1830	1880	1	-30	0	5.95E-05	0.3	0.7	0.36	-7	16	19	11	3UTR
mmu-miR-214-3p	NM_172612	Rnd1	ACAGCAGGCACAGACAGGCAGT#CTGCCTGCCTCTGCTTCCTAAGTGCTGG#.(((((((((.((.(((((((.#))))))).)).))))......))))).	1823	1851	0.92308	-25	1	0.0005815	0.5	3.5	4.02	-7	18	28	7	3UTR
mmu-miR-214-3p	NM_172615	Rubcn	ACAGCAGGCACAGACAGGCAGT#GCTGTTTGTGTACAACCTGTGTTTAGAACTGGATTCTCACCTGCTGTA#((((((((((((((((((((#))))))))))......)))))................))))))))	5169	5217	0.92308	-25	1	0.0110986	0.5	0.2	0.31	-4	22	48	9	3UTR
mmu-miR-214-3p	NM_172643	Zbtb41	ACAGCAGGCACAGACAGGCAGT#CTGAGTCTTAATATCCCTGTTGCCTGCTGG#.(((((((((...(((((....#.)))))))))))))).	6796	6826	1	-24	1	0.0004797	0.6	0.9	0.6	-11	14	16	14	3UTR
mmu-miR-214-3p	NM_172674	Phf20	ACAGCAGGCACAGACAGGCAGT#GCTTGCTTGCTGGTCTGACTGTC#(((((((((.(((.((((((((#))).))))))))))))).)))).	461	484	0.92308	-24	1	3.70E-05	0.5	5.4	3.61	-6	20	23	13	CDS
mmu-miR-214-3p	NM_172674	Phf20	ACAGCAGGCACAGACAGGCAGT#CTGCCTGGTGTGTCGTGTGGG#.(.(((((((((..(((((((.#))))))).)))))).))).).	3636	3657	0.84615	-23	0	0.0026428	0.4	0.8	0.59	-7	17	21	7	3UTR

mmu-miR-214-3p	NM_172674	Phf20	ACAGCAGGCACAGACAGGCAGT#GCCTGAATGTCCCTGCAGTG#((.(((((.(((..(((((...#)))))..))).))))).)).	1847	1867	1	-23	0	0.005504	0.6	1.6	2.14	-9	15	20	5	CDS
mmu-miR-214-3p	NM_172676	Samd10	ACAGCAGGCACAGACAGGCAGT#ACCATATTGAAGAATGTGTGTTTGTGTCTGTCTGTC#((((((((((((((((.(((..#.))).))))))))))	1600	1636	0.92308	-28	1	0.0632484	0.5	3.9	3.37	-4	19	23	12	3UTR
mmu-miR-214-3p	NM_172690	Phf24	ACAGCAGGCACAGACAGGCAGT#GCCTGTCGTGCCGCC#...((.(((((.(((((((...#))))))))))))).	36	51	0.84615	-26	0	0.0012588	0.2	-0	0.18	-12	14	15	14	5UTR
mmu-miR-214-3p	NM_172690	Phf24	ACAGCAGGCACAGACAGGCAGT#GCACATGTGGTGCCTGTCAAAGCTCCCTTCAAGGGACCTGCTGTA#((((((((..(.((((((((.(#.).))))))))...............)..)))))))).	6085	6130	0.84615	-26	1	1.26E-05	0.4	-0	0.21	-10	18	38	8	3UTR
mmu-miR-214-3p	NM_172699	Foxj3	ACAGCAGGCACAGACAGGCAGT#TGTCTGTCACGGTGCCATGCA#...((((((((.((((((((..#))))))))...))))).))).	1797	1818	0.84615	-25	0	3.23E-06	0.4	2.8	3.14	-10	16	21	8	CDS
mmu-miR-214-3p	NM_172699	Foxj3	ACAGCAGGCACAGACAGGCAGT#ACTGACTGCGTGACCTGCTGTT#(((((((((((.(.((((.((((#)))).))))))).)))))))).	3216	3238	0.84615	-25	1	0.0003494	0.4	0.5	0.05	-8	19	22	8	3UTR
mmu-miR-214-3p	NM_172699	Foxj3	ACAGCAGGCACAGACAGGCAGT#CTGTGTGTGTGTGTGTGTGTGTG#((((((.(((((.((((.((((.#)))).))).))))).))).))).	3564	3587	1	-24	0	0.8502462	0.5	0.1	0.33	-7	18	23	5	3UTR
mmu-miR-214-3p	NM_172723	Adap1	ACAGCAGGCACAGACAGGCAGT#GCTGCCCTGTGTCATGTC#...((((((((((...((((((#))))))))))))).))).	2350	2368	0.92308	-21	0	0.0121499	0.4	0.5	0.26	-8	16	18	13	3UTR
mmu-miR-214-3p	NM_172728	Creb5	ACAGCAGGCACAGACAGGCAGT#AGTGTGTGTGTGTGTGTGTGTGTG#((((((.(((((.(((.(((.(#).))).))).))))).))).))).	2709	2733	1	-22	0	0.5825285	0.5	1	0.66	-7	18	24	5	3UTR
mmu-miR-214-3p	NM_172731	Fgd5	ACAGCAGGCACAGACAGGCAGT#GGTGCCTGTCACTCATGCCATGATGTGCATGAACTGCGGCTGTG#(((((..(((((((((((((.(#).))))))))).............))))).........))))).	3934	3978	0.92308	-24	0	3.84E-05	0.4	3.2	2.77	-7	19	44	8	CDS
mmu-miR-214-3p	NM_172731	Fgd5	ACAGCAGGCACAGACAGGCAGT#CTGAGTCTTGTCTGACACTGTCTGCA#...((((((((((((((((((.#)))...))))))))....))))))).	3759	3785	0.92308	-22	0	0.0013358	0.4	1.7	2.05	-7	18	26	8	CDS

mmu-miR-214-3p	NM_172731	Fgd5	ACAGCAGGCACAGACAGGCAGT#AGTGCCTCAAGAACAGGATGGCCAAGGTCTGTGATGGCTGC#.((((...((((((((((((.(#).)))))..................)))))))...)))).	4065	4106	0.84615	-22	0	7.07E-05	0.4	3.6	2.67	-10	17	41	7	CDS
mmu-miR-214-3p	NM_172741	Garre1	ACAGCAGGCACAGACAGGCAGT#CTGCGTGGCTGTTGTCTGTGTTAGCTGTC#(((((.((((((((((.(((((#.))))))))))))))).))))).	5632	5661	0.92308	-27	0	0.0113721	0.5	1.6	0.96	-6	20	23	15	3UTR
mmu-miR-214-3p	NM_172741	Garre1	ACAGCAGGCACAGACAGGCAGT#GCTGCCTGAGGCCTCAGCTGTT#((((((((.(...((((((((#)))))))).))))))..))))).	5472	5494	0.92308	-25	1	0.0004202	0.5	2.1	1.39	-11	18	22	8	3UTR
mmu-miR-214-3p	NM_172751	Arhgef10	ACAGCAGGCACAGACAGGCAGT#GCTGCGTGTGCCCAGTGGTTACTCTGTGCCTGTGCCCTGTG#(((((((((((((((.(((((#))))).))).............)))))))))).....)))).	591	632	0.92308	-28	1	0.0001095	0.4	2.1	1.17	-4	21	41	10	CDS
mmu-miR-214-3p	NM_172751	Arhgef10	ACAGCAGGCACAGACAGGCAGT#CTGCCATTGCTGCTGCCTGCC#...(((((((((.((((((((.#))))))..))))).))))))).	652	673	1	-26	0	0.0159207	0.4	1.5	2.44	-7	17	21	7	CDS
mmu-miR-214-3p	NM_172751	Arhgef10	ACAGCAGGCACAGACAGGCAGT#ACAGCAGTCTGTGTGACCTGCTGA#.(((((((((((((..(((.((#)).)).)))))))...))))))).	4067	4091	0.84615	-25	1	1.50E-06	0.4	1.7	1.13	-4	18	24	7	CDS
mmu-miR-214-3p	NM_172761	Mthfsd	ACAGCAGGCACAGACAGGCAGT#GCTGCCTGTGTGCTTTCAGAAGCACTTGTGCCGTTGA#.((((.((((((((((((((((#))))))))))..............))))))))))).	1817	1854	0.92308	-27	0	0.0001132	0.5	0	-0.4	-6	20	37	11	3UTR
mmu-miR-214-3p	NM_172761	Mthfsd	ACAGCAGGCACAGACAGGCAGT#GCTGTCTGTTCTTCCTGCTGC#.(((((((....((((((((((#))))))))))...))))))).	2303	2324	0.92308	-27	1	2.94E-07	0.4	0.3	0.73	-12	17	21	10	3UTR
mmu-miR-214-3p	NM_172827	Lnpep	ACAGCAGGCACAGACAGGCAGT#CCTGAGTCAGGATGTAAATGGAACTCTGGTTTCTGTGTATGCTGTA#((((((.(((((((.((.(((.#.))).))))))))).)))))).	1180	1226	0.84615	-24	0	0.0005794	0.6	0.1	0.61	-6	18	22	9	CDS
mmu-miR-214-3p	NM_172827	Lnpep	ACAGCAGGCACAGACAGGCAGT#GCTGCTTTCGCAAAGGTTGAGTGAAGGCCGGCCTGCTGC#.((((((((...((.(((((((#))))))))))....................)))))))).	3188	3227	0.84615	-23	1	1.91E-05	0.5	1.2	2.16	-12	17	39	9	3UTR

mmu-miR-214-3p	NM_172827	Lnpep	ACAGCAGGCACAGACAGGCAGT#CTGCTGTAAGAACCACTGTGTTTGTAGGTTGCTGA#.((((((.(((((((((((((.#))))))............)))))))).).))))))).	3473	3508	0.84615	-22	1	0.0104888	0.6	3.7	3.18	-6	19	35	7	3UTR
mmu-miR-214-3p	NM_172835	Peli3	ACAGCAGGCACAGACAGGCAGT#GCTGCCTGCCCCTTCTGTGGAGCTTGGCTCACCGGTGAGCTTGGCTGTG#((((((((((((((((((((#)))))))))).....)))))....................))))).	1413	1462	0.89231	-26	1	2.79E-06	0.4	3.5	3.84	-4	22	49	8	CDS
mmu-miR-214-3p	NM_172835	Peli3	ACAGCAGGCACAGACAGGCAGT#CTCCTGCTGTTCAGAGGGAGCTCTGCATGTGGGACTCTGCCTGCTGG#.(((((((((.((((.((((.#.)))).)))....)).))))))))).	1545	1592	0.87179	-26	1	5.58E-07	0.4	2	2.38	-7	18	26	9	3UTR
mmu-miR-214-3p	NM_172862	Frem2	ACAGCAGGCACAGACAGGCAGT#GGTGCCTGTGAGTGCCAGCCTGTC#((((.((((((..((((((((.(#).))))))))..)))))).)).))).	11287	####	1	-27	0	0.000386	0.4	2.1	2.81	-10	18	24	7	3UTR
mmu-miR-214-3p	NM_172862	Frem2	ACAGCAGGCACAGACAGGCAGT#GGTGCCTGTGAGTGCCAGCCTGTC#((((.((((((..((((((((.(#).))))))))..)))))).)).))).	11443	####	0.92308	-27	0	0.0033969	0.4	2.1	2.22	-10	18	24	7	3UTR
mmu-miR-214-3p	NM_172863	Zfp697	ACAGCAGGCACAGACAGGCAGT#GTCTTCTTTAGTGAAGACTTACACTTGCAAAGTCTGTGCCGCCTATTCTGACTCATGCTGTC#((((((((((((((((..((((.#.))))...))))))))))...............))))))).	3798	3860	0.92308	-26	1	0.0039285	0.5	0.5	0.43	-4	19	39	9	3UTR
mmu-miR-214-3p	NM_172877	Pramel12	ACAGCAGGCACAGACAGGCAGT#ACTGCCTGGGCCTGCTGG#.((((((((.(...((((((((#)))))))))))))))))).	615	633	0.84615	-29	1	1.26E-05	0.5	0.3	0.07	-11	17	18	17	5UTR
mmu-miR-214-3p	NM_172877	Pramel12	ACAGCAGGCACAGACAGGCAGT#ACAGCCAGTCTGAGCCAGCTGA#.((((.((((.((((((.((((.((#)).))).)))))).))).)))).	1966	1988	0.84615	-25	0	0.0026022	0.4	-1	-0.1	-7	17	22	5	CDS
mmu-miR-214-3p	NM_172904	Fsd2	ACAGCAGGCACAGACAGGCAGT#ACTGGAGCCCTTTTCACCTGTGTGCTGTGCCCTGCTGA#.((((((((((((.((.((((.#.)))).))))))))).))))).	2465	2503	1	-30	1	5.94E-05	0.4	-0	0.4	-5	18	22	9	3UTR
mmu-miR-214-3p	NM_172924	Peak1	ACAGCAGGCACAGACAGGCAGT#ACTGTCCTTCACCTTCACCCTGTCCCGCCTGCTGA#.((((((((...((((((....#.))))))..))))))))	6261	6296	1	-26	1	0.0608137	0.5	0.1	0.6	-11	14	18	8	3UTR

mmu-miR-214-3p	NM_172924	Peak1	ACAGCAGGCACAGACAGGCAGT#TTGCCTGTTGGTTTGTGGCAAGTCTGTGTGTCTGCA#...((((((((((((((((.#))))))))))...................))))))))).	10461	####	1	-26	0	0.0003197	0.5	4.1	2.39	-7	18	36	9	3UTR
mmu-miR-214-3p	NM_172943	Alkbh5	ACAGCAGGCACAGACAGGCAGT#CTGCATTGGGTCTGCTGTGCCCATACATCTCACCTGCTGTC#(((((((((((((.(((((...#.))))))))))))).............)))))).	2356	2397	0.84615	-26	1	0.0200833	0.5	0.2	0.23	-5	18	33	12	3UTR
mmu-miR-214-3p	NM_172943	Alkbh5	ACAGCAGGCACAGACAGGCAGT#GGTGCTTGTCTGTAATGCCAGCA#...((.(((((((((((((.(#).)))))))))))...))).)).	4881	4904	1	-24	0	0.0004026	0.5	0.2	0.09	-8	17	23	11	3UTR
mmu-miR-214-3p	NM_172947	Lsm12	ACAGCAGGCACAGACAGGCAGT#CTGTGACTTTCCTTCCCTAGTCTGTGTCTGCC#...(((((((((((((((....#.))).)))))))))))).	944	976	0.84615	-26	0	0.0204087	0.5	4.3	4.27	-7	15	18	12	3UTR
mmu-miR-214-3p	NM_172952	Gphn	ACAGCAGGCACAGACAGGCAGT#GCTCCTGTCAGTGCGGTGACTGC#.(((((.((((.((((((.(((#))))))))).)))))..)).))).	90	113	0.92308	-23	0	1.27E-05	0.4	1.9	2.45	-9	18	23	9	5UTR
mmu-miR-214-3p	NM_172952	Gphn	ACAGCAGGCACAGACAGGCAGT#GCTTTTAGTAGACAGCGGTAGGTGCCTGCA#...((((((((..((..((.((#.)).)).))..)))))))).	2712	2742	0.92308	-22	0	0.0002091	0.6	4.9	4.84	-11	14	20	8	3UTR
mmu-miR-214-3p	NM_173002	Zxdc	ACAGCAGGCACAGACAGGCAGT#CTGCCTGTCTACGTCCTCCAGGAGGTGCTTCCAGCACCTGGAGGTTTTGCTGG#.(((((((((((((((((((.#)))))))))))..............))))).................))))).	2634	2687	0.92308	-27	1	4.25E-05	0.4	1.3	0.82	-4	20	53	10	CDS
mmu-miR-214-3p	NM_173002	Zxdc	ACAGCAGGCACAGACAGGCAGT#GCTGCCTGGTGGCTCTGCCTGTG#((((((((.((..((((((((#)))))))).)))).)))).))).	1996	2019	0.84615	-26	1	5.18E-06	0.5	1.2	1.27	-7	19	23	8	CDS
mmu-miR-214-3p	NM_173005	Setd5	ACAGCAGGCACAGACAGGCAGT#AGTGCATTGTGCCTGTTGTA#((((((((((((.....(((.(#).)))..)))))))))))).	5584	5604	0.92308	-23	1	0.0006783	0.4	1.8	1.49	-7	16	20	12	3UTR
mmu-miR-214-3p	NM_173053	Limk2	ACAGCAGGCACAGACAGGCAGT#TGCCTGTGTGTGTCTAGCC#...(((((((((.((((((((..#))))))).))))))).)).	84	103	0.92308	-27	0	0.0020599	0.4	-1	-0.1	-8	16	19	7	5UTR
mmu-miR-214-3p	NM_173053	Limk2	ACAGCAGGCACAGACAGGCAGT#GCTGCCCTACTCTGTCACGCTCATCTCCATGCCTGCTA#..((((((((((((..((((((#))))))....)))))...............))))))).	754	792	0.84615	-25	1	0.2652571	0.4	-0	-0.5	-6	18	38	7	CDS

mmu-miR-214-3p	NM_173053	Limk2	ACAGCAGGCACAGACAGGCAGT#GCTGAGCCTGTTTATCTGCTGTG#((((((((...((((((((((#)))..)))))))).)))))))).	352	375	1	-24	1	0.002662	0.5	0.1	-0.1	-9	19	23	8	5UTR
mmu-miR-214-3p	NM_173364	Zfp445	ACAGCAGGCACAGACAGGCAGT#ACTGATTCTCCTGCTGTCTCGGTGCCTGACTA#..(((((((((((((.((((.#.)))))))))..))))))).))	809	841	1	-26	1	0.0001693	0.5	0.4	0.12	-5	18	23	9	CDS
mmu-miR-214-3p	NM_173364	Zfp445	ACAGCAGGCACAGACAGGCAGT#GCTTGTGTTGGTGCTGTTCTGCTGA#.((((((((((..((((((...#))))))....))))....)))))).	3559	3584	0.92308	-23	1	0.0001659	0.6	1.5	0.56	-8	16	25	6	3UTR
mmu-miR-214-3p	NM_173364	Zfp445	ACAGCAGGCACAGACAGGCAGT#GCATGTCTGTAGCCTAGTAAGCGTTGTCTGTTGG#.(((((((((((((((.((...#)).))))))))...............)))))))).	3489	3523	0.92308	-22	1	0.0007909	0.5	2.3	1.24	-4	17	34	8	3UTR
mmu-miR-214-3p	NM_173382	Proser1	ACAGCAGGCACAGACAGGCAGT#GCTGTCTGTGTGTGCAGTGTGCGCTAGGACCCGTGCTGG#.(((((.(((((.(((((((((#)))))))))).)))))..................))))).	4057	4096	0.84615	-27	0	1.68E-05	0.4	0.7	0.58	-7	19	39	9	3UTR
mmu-miR-214-3p	NM_173382	Proser1	ACAGCAGGCACAGACAGGCAGT#CTGCGGCTGTTCCTGCTGC#.(((((((.((((....((((.#))))..)))).))))))).	2011	2030	0.84615	-24	1	1.43E-05	0.4	-0	0.94	-7	15	19	7	CDS
mmu-miR-214-3p	NM_173396	Tgif2	ACAGCAGGCACAGACAGGCAGT#CTGCTGGCTGTGTCTGTTC#..(((((((((((.(.(((((.#))))).)))))))))))).	602	621	1	-25	1	3.50E-05	0.4	1	1.31	-6	17	19	12	CDS
mmu-miR-214-3p	NM_173396	Tgif2	ACAGCAGGCACAGACAGGCAGT#GCTGCACTGTTACGGGGCCTGTACTCTGCTGA#.(((((((.(((((((((((((#))))).))))........)))).	1103	1135	0.84615	-24	1	1.67E-05	0.4	0.3	0.74	-5	20	32	6	3UTR
mmu-miR-214-3p	NM_173396	Tgif2	ACAGCAGGCACAGACAGGCAGT#ACTGCTACCCCACCTGTCTGTGAGACCCCATGATGGCTGTA#(((((...(((((((((((((#))))).........)))))))))..............))))).	2563	2604	0.92308	-23	0	0.0001092	0.4	0.1	0.1	-10	19	41	9	3UTR
mmu-miR-214-3p	NM_173396	Tgif2	ACAGCAGGCACAGACAGGCAGT#ACTGGCTTGGAGTGCCTGTTC#..(((((((((...((((((((#)))).))))..))))))))).	2278	2299	1	-23	1	2.21E-05	0.4	0.4	0.24	-10	17	21	9	3UTR
mmu-miR-214-3p	NM_173399	Zbtb5	ACAGCAGGCACAGACAGGCAGT#TGCCTGCAAAATCTGCTGTA#((((((((....(.((((((..#)))))))....)))))))).	2010	2030	0.84615	-22	1	0.0037742	0.5	-0	-0	-13	15	20	8	CDS

mmu-miR-214-3p	NM_173408	Dcun1d3	ACAGCAGGCACAGACAGGCAGT#CTGCCTGCTGCCCTGCA#...(((((..(((.(((((((.#)))))))))).))))).	1257	1274	0.92308	-26	0	8.07E-06	0.5	-0	0.35	-10	15	17	10	3UTR
mmu-miR-214-3p	NM_173408	Dcun1d3	ACAGCAGGCACAGACAGGCAGT#GCTGCCACTGAGGCCTGCC#...((((((.(((...(((((#)))))).)))..)))))).	492	511	0.84615	-25	0	3.89E-07	0.4	0.2	0.52	-10	15	19	6	CDS
mmu-miR-214-3p	NM_173408	Dcun1d3	ACAGCAGGCACAGACAGGCAGT#CTGCCTGTCCATCGCCAGCA#...((.(((...(((((((((.#)))))))))....))).)).	109	129	0.92308	-24	0	0.005744	0.4	1.9	1.45	-16	14	20	9	5UTR
mmu-miR-214-3p	NM_173413	Rab8b	ACAGCAGGCACAGACAGGCAGT#ACTGCCTGTCCTGGAAGCTGCTGTC#((((((...((((((((((((#)))))))))).))....))))))).	942	967	1	-31	1	0.0013987	0.5	1.9	0.65	-9	19	25	10	3UTR
mmu-miR-214-3p	NM_173442	Gcnt1	ACAGCAGGCACAGACAGGCAGT#CTGCAGGCACAGGCTCCAGGCTGTTGGAAAGCCTGCTGG#.((((((((...(((((.(...#.).))))))).....)))))))).	92	131	1	-24	1	8.31E-05	0.4	-0	0.1	-11	14	22	8	5UTR
mmu-miR-214-3p	NM_173446	Fam155a	ACAGCAGGCACAGACAGGCAGT#CTGTCCCTAAGCAGACAACATCCGCTTGCTTCTGTCTGTGTCTCGCATGG#.(((((((((((((((((.(((#.)))))))))))))))).)).)).	2048	2098	0.96154	-26	1	0.0018955	0.4	3.8	4.2	-4	20	24	16	3UTR
mmu-miR-214-3p	NM_173446	Fam155a	ACAGCAGGCACAGACAGGCAGT#GGTGCTTGGATGTGTCGGCAGTA#((.(((.((((((..((((((.(#).))))))..)))))).)).)).	14	37	0.84615	-22	0	3.29E-05	0.5	2.2	2.9	-10	17	23	6	CDS
mmu-miR-214-3p	NM_173739	Galnt18	ACAGCAGGCACAGACAGGCAGT#GCTGCAATGCCGCCTGCTGTC#(((((((((.(((.....(((((#)))))..))..))))))))).	2346	2367	0.84615	-23	1	0.0008328	0.4	1	0.61	-8	16	21	9	3UTR
mmu-miR-214-3p	NM_174846	Glyctk	ACAGCAGGCACAGACAGGCAGT#CTGATCATGGTTTCTCCTCTCCTGCTGTTGCCTGACTGG#.((((((((((((.((((.((.#.)))))))))).))))).))).	2458	2497	1	-25	1	0.0009536	0.4	1.9	2.61	-5	18	22	10	3UTR
mmu-miR-214-3p	NM_174846	Glyctk	ACAGCAGGCACAGACAGGCAGT#ACTGTCACCATTGCCTGCTGA#.(((((((((......((((((#)))))).....))))))))).	3485	3506	1	-24	1	0.0032604	0.5	1.2	2.47	-13	15	21	9	3UTR
mmu-miR-214-3p	NM_174849	Agbl5	ACAGCAGGCACAGACAGGCAGT#GCTTGCCTGTTTCCTGCTC#..((((((...(((((((((((#))).)))))))))))))).	2002	2021	0.92308	-23	1	0.0002095	0.5	0.8	1.63	-10	17	19	14	CDS

mmu-miR-214-3p	NM_175074	Hmgn3	ACAGCAGGCACAGACAGGCAGT#GGTGCTTTTGTGCCTA#.....(((((((((..((((.(#).))))))))))))).	761	777	0.84615	-17	0	2.07E-05	0.7	1.4	1.02	-15	14	16	13	3UTR
mmu-miR-214-3p	NM_175104	Fam53c	ACAGCAGGCACAGACAGGCAGT#ACGCCTGCATCTGTGCTGCGTGTG#((((((.((((((((((((.((#)))))))..)))))))))).))).	4199	4223	0.84615	-25	0	0.0001901	0.4	2.2	1.48	-5	20	24	10	3UTR
mmu-miR-214-3p	NM_175104	Fam53c	ACAGCAGGCACAGACAGGCAGT#CTGCCTCTGTGGCTGTG#...((((.((((((..(((((.#))))))))))).)))).	4468	4485	0.92308	-24	0	4.11E-05	0.7	2.9	2.68	-10	15	17	11	3UTR
mmu-miR-214-3p	NM_175104	Fam53c	ACAGCAGGCACAGACAGGCAGT#ACTGCCTTTTGGAAGCTGGCTGTA#(((((.(((.((((.(((((((#)))))))))))...))).))))).	2805	2829	1	-24	0	3.06E-05	0.5	0.4	0.16	-8	19	24	11	3UTR
mmu-miR-214-3p	NM_175124	Lrrc28	ACAGCAGGCACAGACAGGCAGT#GCTGCTCCACCCAGTGTCTGCGGACTTTTAACCTGCTGTG#(((((((((.((((((((((((#))))))........)))))).).........)))))))).	1188	1228	0.84615	-26	1	0.0019835	0.5	-0	-0	-5	21	40	8	CDS
mmu-miR-214-3p	NM_175187	Tmem161b	ACAGCAGGCACAGACAGGCAGT#ACTGTCTGTCCCACGACCTGTTC#..(((((((...((((((((((#))))))))))....).))))))).	105	128	0.84615	-22	1	0.0002756	0.5	0.3	1.59	-12	17	23	10	5UTR
mmu-miR-214-3p	NM_175187	Tmem161b	ACAGCAGGCACAGACAGGCAGT#ACTGTTAGTTTTGGGCCTTGCTGTA#((((((((((.(((((.((((((#)))))).)))).).)))).))))).	2279	2304	0.92308	-22	1	0.0281235	0.7	0.3	0.8	-5	20	25	6	3UTR
mmu-miR-214-3p	NM_175231	Nsd2	ACAGCAGGCACAGACAGGCAGT#ACTGTCTGTCCTTTGGCTTGCTT#..(((((((.((((((((((((#))))))))))...))))))))).	5149	5172	0.84615	-26	1	0.0001266	0.4	-1	-0.5	-6	19	23	10	3UTR
mmu-miR-214-3p	NM_175231	Nsd2	ACAGCAGGCACAGACAGGCAGT#GCTGCCTTTGTTTTGCCTGTTT#..((((((((.((((((((((#)))))))..)))))))))))).	4766	4788	0.92308	-25	1	0.0579734	0.5	-0	-0.3	-8	19	22	12	3UTR
mmu-miR-214-3p	NM_175231	Nsd2	ACAGCAGGCACAGACAGGCAGT#TGCCTGTCTGGCAGCA#...((..(((.((((((((((..#))))))))))))).))).	2416	2432	1	-25	0	2.21E-06	0.5	2.9	3.11	-11	14	16	12	CDS
mmu-miR-214-3p	NM_175231	Nsd2	ACAGCAGGCACAGACAGGCAGT#ACTCACTGCTGTCCTGCTGC#.(((((((.((((.(((..(((#)))..)))))))))))))).	5326	5346	0.98077	-24	1	0.0002404	0.5	-1	-0.1	-6	17	20	14	3UTR
mmu-miR-214-3p	NM_175303	Sall4	ACAGCAGGCACAGACAGGCAGT#GCTGCCTGTGTCGCTGGGGGCCAGCTC#..(((.(((.(((((((((((#)))))))))))....)))...))).))).	3192	3219	0.92308	-24	0	4.58E-07	0.4	2.3	3.44	-8	18	27	9	CDS

mmu-miR-214-3p	NM_175303	Sall4	ACAGCAGGCACAGACAGGCAGT#CCTGGAGCAGATCCTGTGCCTGCA#...((((((((((....((...#.)).....)))))))))).	798	822	1	-23	0	8.34E-05	0.4	0.3	0.13	-8	12	19	10	CDS
mmu-miR-214-3p	NM_175380	Gpd1l	ACAGCAGGCACAGACAGGCAGT#GCCTCTCTGACCTGCTGTT#((((((((..((((.((((...#)))).)))).)))))))).	2207	2226	1	-26	1	0.0032438	0.4	-0	-0	-7	16	19	8	3UTR
mmu-miR-214-3p	NM_175388	Rnf169	ACAGCAGGCACAGACAGGCAGT#GCTGGCCTTTCCTGTGTGTGTGAGCTGTT#(((((..(((((.(((((((((#)))).......))))).)))))..))))).	3286	3315	1	-26	0	0.0004885	0.5	0.5	0.31	-9	19	29	5	3UTR
mmu-miR-214-3p	NM_175388	Rnf169	ACAGCAGGCACAGACAGGCAGT#GCTGCCTCTCTGCCCCTGACTTAACCATCGAAAAGCGCCTGCC#...((((((.((((.(((((((#))))))).))))......................)))))).	991	1034	0.92308	-24	0	0.0046954	0.4	0.4	0.15	-8	17	43	7	CDS
mmu-miR-214-3p	NM_175398	Inka2	ACAGCAGGCACAGACAGGCAGT#CTGAGTGCCCTCGCTGGCAGGGTAGTGGAGGGCCTGCTGGGCCTGCTGC#.(((((((((.(((.(((((...#.)))))))).)))))))).	390	439	0.97436	-31	1	3.64E-05	0.4	0.2	0.27	-6	16	19	8	CDS
mmu-miR-214-3p	NM_175398	Inka2	ACAGCAGGCACAGACAGGCAGT#GCCTGTCTGTGGAACTGGGGCCTGTCTGC#.((((((((((((((((...#)))))))))))).........))))).))).	4332	4361	0.96154	-30	1	0.0004283	0.4	-0	-0.3	-4	18	29	10	3UTR
mmu-miR-214-3p	NM_175403	Mlec	ACAGCAGGCACAGACAGGCAGT#GCTGCCTGTTGATGAAGTGTGTGACCTTTTCTCCTGCTGA#.(((((((((((((((((((#)))))))))).......)))).........))))))).	3935	3975	0.84615	-29	1	0.000617	0.4	1.5	1.75	-4	21	40	10	3UTR
mmu-miR-214-3p	NM_175403	Mlec	ACAGCAGGCACAGACAGGCAGT#GCTGCTTCTGTTGCTGCTGC#.((((((..(((((..((((((#)))))))))))..)))))).	189	209	0.92308	-25	1	0.0007177	0.3	0.6	0.75	-8	17	20	11	CDS
mmu-miR-214-3p	NM_175403	Mlec	ACAGCAGGCACAGACAGGCAGT#TTGCACTGTTTGTTCCCCGCTGTG#(((((.((.(((((((((((.#)))).)))))))).))..))))).	3478	3502	0.84615	-24	0	0.0539131	0.5	0.3	0.27	-7	19	24	8	3UTR
mmu-miR-214-3p	NM_175403	Mlec	ACAGCAGGCACAGACAGGCAGT#ACTGTCTTCTGTGTTCTCTGTG#((((..((((((((.((((((#))))))))))))))..)))).	2729	2751	1	-24	0	0.0051424	0.5	0.4	0.87	-11	19	22	15	3UTR
mmu-miR-214-3p	NM_175414	Tspan9	ACAGCAGGCACAGACAGGCAGT#GCTGCCTGGGGGCCATCAAGGAGAACAAGTGTCTGCTT#..(((((((((...((((((((#))))))))..................))))))))).	393	431	0.84615	-26	1	0.0061318	0.5	0.2	0.68	-10	17	38	9	CDS

mmu-miR-214-3p	NM_175414	Tspan9	ACAGCAGGCACAGACAGGCAGT#GTCATGTCTCCTGCCTGCTC#..((((((((.((((((((...#))).)))))..)))))))).	960	980	0.92308	-24	1	0.0626299	0.3	-0	-0.1	-8	16	20	8	3UTR
mmu-miR-214-3p	NM_175433	Zfp710	ACAGCAGGCACAGACAGGCAGT#GCTGCCATGTCTGTGTCGAATGCG#...((((((((((((((((((#)))))).))))))))))...))).	1503	1527	0.84615	-30	0	4.49E-06	0.4	3.5	3.89	-7	19	24	10	CDS
mmu-miR-214-3p	NM_175433	Zfp710	ACAGCAGGCACAGACAGGCAGT#ATGAGGTGTCTGTGCCTGGTGA#.((.((((((((((((......#.)))))))))))).)).	651	673	1	-27	0	3.92E-06	0.4	0.4	0.62	-7	14	17	12	CDS
mmu-miR-214-3p	NM_175471	Cyb5rl	ACAGCAGGCACAGACAGGCAGT#ACTTCCCTTCCTGTCTGCTGCGTCCTGCTGTA#(((((((((.(((.(((((((.#.)))))))))).).)))))))).	1684	1716	0.92308	-31	1	0.0003768	0.4	0	0	-6	19	23	10	3UTR
mmu-miR-214-3p	NM_175471	Cyb5rl	ACAGCAGGCACAGACAGGCAGT#GCTGTCTGCTGGCCTGACTGA#.((((((((.(((.((((((((#)))))))))))))))).))).	1515	1536	0.92308	-29	1	1.54E-05	0.4	0	0	-6	19	21	16	CDS
mmu-miR-214-3p	NM_175483	Snx33	ACAGCAGGCACAGACAGGCAGT#CTGCTCCTGGGTCCTGCTGC#.((((((((.(((...(((((.#))))).))).).))))))).	4508	4528	1	-23	1	8.73E-06	0.5	0	-0.3	-7	16	20	7	3UTR
mmu-miR-214-3p	NM_175485	Prtg	ACAGCAGGCACAGACAGGCAGT#GCTGTGTGTCCATTGTGTGTGTGCTGTG#((((((.(((((((((.(((((#))))).)))).....))))).))))))).	4129	4157	0.84615	-29	0	0.0296734	0.5	1.2	1.44	-5	20	28	6	3UTR
mmu-miR-214-3p	NM_175485	Prtg	ACAGCAGGCACAGACAGGCAGT#GCTGTAAAACCCTCTCTGTCCTGGAACCTGCTGTA#((((((((..((((((((((((#)))))).........)))))).))...)))))))).	7974	8009	0.92308	-25	1	0.0173133	0.5	0	0.14	-6	20	35	8	3UTR
mmu-miR-214-3p	NM_175556	Plch2	ACAGCAGGCACAGACAGGCAGT#GCTGCCGCTGTGTGGAGGTGGACTGCTGG#.((((((((((((.(.((((((#)))))))))))))).........))))	1359	1388	1	-26	1	0.0021455	0.3	2.5	2.37	-5	19	29	13	CDS
mmu-miR-214-3p	NM_175556	Plch2	ACAGCAGGCACAGACAGGCAGT#GCTGCCCGTCCTGACCTGCCTCCTGTG#((((.((((((((((.((((((#)))))).))).))...))))).)))).	4391	4418	0.92308	-26	0	0.0287406	0.4	2.7	2.94	-8	20	27	6	3UTR
mmu-miR-214-3p	NM_175631	Cbln4	ACAGCAGGCACAGACAGGCAGT#GCTGCCTGTCTGGGCA#.......((.((((((((((((#)))))))))))).)).	1224	1240	0.84615	-26	0	9.12E-06	0.4	4.4	4.22	-18	14	16	12	CDS
mmu-miR-214-3p	NM_175631	Cbln4	ACAGCAGGCACAGACAGGCAGT#ACTGTCAGAAGCCGTCTCAAGGTTTGTCCTGTTTAGTTTCCTGCTGTT#((((((((.(((((((((((((.#.))).))))))).))..)))))))).	2004	2052	1	-24	1	0.0026695	0.5	0.7	0.21	-5	20	26	8	3UTR

mmu-miR-214-3p	NM_175684	Fchsd1	ACAGCAGGCACAGACAGGCAGT#GCTGCCTTCTGGATGCCACTGTG#((((..((((((((.(((((((#))))))))))))..)))).))))).	258	281	1	-24	0	0.0052265	0.4	1.9	2.45	-11	19	23	11	CDS
mmu-miR-214-3p	NM_175684	Fchsd1	ACAGCAGGCACAGACAGGCAGT#CTGGCTATCCGTCCCCTGGCAATGCCTGCTGG#.(((((((((....((((....#.))))....))))))))).	2842	2874	1	-24	1	2.84E-05	0.4	2.1	1.95	-12	13	19	9	3UTR
mmu-miR-214-3p	NM_176832	Spire1	ACAGCAGGCACAGACAGGCAGT#GCTTCCTGTTCCTCCAGCCACCTTGTGCAGGACCGTTTGTCCTGCTTTCCTGCTGTG#((((((((..(((((((((.((#.)).))))))).))....))))))	3881	3938	1	-23	1	0.0008571	0.5	1.2	2.24	-6	19	27	8	3UTR
mmu-miR-214-3p	NM_176839	Abcc5	ACAGCAGGCACAGACAGGCAGT#CCTGTCCATCGTGTGCCTGATGA#.((.((((((((((((((....#))))))).....)))))))).)).	738	761	0.92308	-23	0	9.32E-05	0.4	1.9	2.58	-7	16	23	8	CDS
mmu-miR-214-3p	NM_176893	Mink1	ACAGCAGGCACAGACAGGCAGT#GCTGCTTGCTGATAGCAATGGCTACACAAACCTGCCTGATGTG#(((.((((((((((.((((((((#))))))))))))....................)))))).))).	2810	2853	1	-24	0	0.0011015	0.5	3.5	2.98	-8	20	43	11	CDS
mmu-miR-214-3p	NM_176893	Mink1	ACAGCAGGCACAGACAGGCAGT#GCTGCCTTCCCAGCCTCCCACGACCCTGACCCTGCTGC#.(((((((..(((..((((((((#)))))))..................)))..))))))).	2025	2063	0.84615	-23	1	0.1190285	0.3	2.9	2.39	-8	17	38	7	CDS
mmu-miR-214-3p	NM_176916	Pld5	ACAGCAGGCACAGACAGGCAGT#AGTGCATCGTGATCTTCGCTCTGGTCTGCTGC#.((((((((.((((..((....#.))..))))))))))))).	637	669	1	-19	1	0.0007216	0.5	0.3	0.85	-6	14	18	12	CDS
mmu-miR-214-3p	NM_177077	Exoc6b	ACAGCAGGCACAGACAGGCAGT#GCTGCCTGTCTAGTTGCCACCTCTGTC#((((..(((((((((((((((((#))))))))))))).)).)))....)))).	4351	4378	0.92308	-28	0	0.0539729	0.5	3.6	4.22	-10	20	27	11	3UTR
mmu-miR-214-3p	NM_177077	Exoc6b	ACAGCAGGCACAGACAGGCAGT#GCTTGCCTTTGCCCAGCTCTGCGTCTGCTGTG#(((((((((.(((((.(((((.#.)))))..).)))).)))))))))	4496	4528	0.92308	-27	1	9.44E-06	0.4	3	2.91	-5	19	25	9	3UTR
mmu-miR-214-3p	NM_177077	Exoc6b	ACAGCAGGCACAGACAGGCAGT#GCTGCCTGGAAGTCTGTGGCCTTCTA#..((.(((((((((((((((((#)))))))....)))))))).))).)).	4762	4788	0.92308	-26	0	0.002002	0.4	3.4	3.85	-9	19	26	7	3UTR
mmu-miR-214-3p	NM_177089	Tacc1	ACAGCAGGCACAGACAGGCAGT#CTGCTCAGCTGTTCCTGCTGTG#((((((((.(((((...(((((.#)))))...)))).)))))))).	6280	6302	0.92308	-27	1	2.67E-05	0.5	1.9	1.52	-7	17	22	8	3UTR

mmu-miR-214-3p	NM_177205	9430015G10Rik	ACAGCAGGCACAGACAGGCAGT#GCTCCTGACTGGCCTTCTGA#.(((.((((.(((.(((.(((#))))))).))))))).))).	331	351	0.84615	-22	0	0.0129436	0.4	1.6	0.66	-10	17	20	7	CDS
mmu-miR-214-3p	NM_177205	9430015G10Rik	ACAGCAGGCACAGACAGGCAGT#GCGCTAGCTGTGTCCGGTGCTGG#.(((((((((((((..((((...#)).))..)))))))...))))).	489	512	0.92308	-22	1	2.35E-05	0.4	0.9	0.77	-5	16	23	7	CDS
mmu-miR-214-3p	NM_177224	Chd9	ACAGCAGGCACAGACAGGCAGT#ATTGCCTGCCAATGCCATGCA#...(((((((..(.((((((((#)))))))).)..)))).))).	5794	5815	1	-22	0	0.0069271	0.5	1.9	2.92	-14	16	21	8	CDS
mmu-miR-214-3p	NM_177224	Chd9	ACAGCAGGCACAGACAGGCAGT#ACTGCAGGGCTGATGGGCATGCCTGCCGG#.(.((((((((((.(..(((((#)))))..).)))......))))))).	8351	8380	0.84615	-22	0	3.33E-05	0.4	3.8	3.57	-6	17	29	7	CDS
mmu-miR-214-3p	NM_177242	Pptc7	ACAGCAGGCACAGACAGGCAGT#TTGTCTGTCTGTGCGTTTGTATATTTGAGTAGCTGTC#((((((.((((((((((((((.#)))))))))))))))).)..............))))).	2583	2620	0.92308	-28	0	0.003375	0.7	1.7	2.81	-5	20	37	14	3UTR
mmu-miR-214-3p	NM_177242	Pptc7	ACAGCAGGCACAGACAGGCAGT#CTGCCACATGTGCTGATCCTGCTGG#.(((((((..(((((((((((.#)))))...))).)))..))))))).	1252	1277	0.84615	-26	1	0.0001902	0.4	1.2	1.15	-6	18	25	7	3UTR
mmu-miR-214-3p	NM_177242	Pptc7	ACAGCAGGCACAGACAGGCAGT#GCTGCCTGTAAGATGCCACGGGCCTTCCAGGGCTAGAGTGTGCAGCTGC#.((((..(((((.(((((((((#)))))))))...........................))))).)	1995	2044	0.92308	-25	0	6.73E-05	0.4	-0	0.17	-9	18	49	9	3UTR
mmu-miR-214-3p	NM_177242	Pptc7	ACAGCAGGCACAGACAGGCAGT#ATTGGCTGAAGCTGTGCGTGTCTGA#.(((((.((((((.(((.((((#)))).)))...)))))).)).))).	4112	4137	0.92308	-24	0	0.0004959	0.5	0.7	0.45	-6	18	25	6	3UTR
mmu-miR-214-3p	NM_177260	Tmem154	ACAGCAGGCACAGACAGGCAGT#GCTGCCTGCCAGCCCACTCATGGTGAGGGAGCCCTGTGTGGTGCTGG#.(((((.((((((.((((((((#))))))))......................))))))..))))).	914	961	0.92308	-26	0	0.0002283	0.4	0.1	0.04	-6	19	47	8	3UTR
mmu-miR-214-3p	NM_177260	Tmem154	ACAGCAGGCACAGACAGGCAGT#ACTGTCTTGGGACTGTGCAGTTCTCAGACAAAAGGCTGTG#(((((..((((((.((((((((#))))))).)...))))))...............))))).	2135	2175	0.84615	-21	0	3.90E-05	0.5	0.5	-0.2	-8	19	40	7	3UTR
mmu-miR-214-3p	NM_177275	Amigo3	ACAGCAGGCACAGACAGGCAGT#ACTGCCTGAGATCTCCTGCTGC#.(((((((......((((((((#))))))))......))))))).	1748	1770	1	-26	1	0.0010159	0.5	0.6	0.86	-14	15	22	8	CDS

mmu-miR-214-3p	NM_177275	Amigo3	ACAGCAGGCACAGACAGGCAGT#GCTGGTGTTGCTCTACTTGTTTGCACCACCCTGTCGTGGCTGCTGTC#(((((((.(((.((((((....#.))))))))).))))))).	1408	1455	0.82692	-28	1	1.48E-05	0.4	2.9	1.84	-10	16	19	9	CDS
mmu-miR-214-3p	NM_177290	Itgb8	ACAGCAGGCACAGACAGGCAGT#GCTGTCCAGTTGATTCAATAGAATACCTGTCTGTGCATGTTGTA#((((((.(((((((((((....#.))))))))))).)))))).	755	799	1	-30	0	0.0051468	0.6	2.2	2.47	-5	17	20	11	CDS
mmu-miR-214-3p	NM_177290	Itgb8	ACAGCAGGCACAGACAGGCAGT#TTGCCTGGTGCCATTGCTGG#.((((((((((...(((((((.#))))))))))))..)))))).	1549	1569	1	-25	1	5.80E-05	0.5	3.5	2.95	-9	17	20	12	CDS
mmu-miR-214-3p	NM_177333	Exoc3	ACAGCAGGCACAGACAGGCAGT#CCTGTCCTCCCTGCTGC#.(((((((....((((((....#))))))...))))))).	2329	2346	0.92308	-22	1	0.0339092	0.5	0.4	3.28	-12	13	17	7	3UTR
mmu-miR-214-3p	NM_177333	Exoc3	ACAGCAGGCACAGACAGGCAGT#CTGCCTGCTTTTTGACAATGAAGGAGACAGACCTGGAGGCTGTTGC#.((((((.(.((((.((((((((.#))))))))........................)))..).))))))).	73	119	0.84615	-21	1	0.0005508	0.5	0.2	0.19	-8	17	46	7	CDS
mmu-miR-214-3p	NM_177351	Hykk	ACAGCAGGCACAGACAGGCAGT#ACTCTTGTCTGATGTTTGCCTGCC#...((((((((((((((.(((#)))))))))))).....))))))).	4139	4163	0.84615	-25	0	0.0868571	0.6	-0	0.29	-7	18	24	11	3UTR
mmu-miR-214-3p	NM_177351	Hykk	ACAGCAGGCACAGACAGGCAGT#CTGCCCAGTCATTGGCCGCTGG#.((((.((((.(((((.(((((.#)))))..)))..))))))))).	2115	2137	1	-23	0	0.0001003	0.6	0	0	-7	17	22	9	3UTR
mmu-miR-214-3p	NM_177351	Hykk	ACAGCAGGCACAGACAGGCAGT#GCTGCTTGCCCTTCTCACAGTCAGCCATCGAGCCCTGCTGC#.(((((((....((((((((((#))))))))..................))....))))))).	4449	4490	0.84615	-22	1	0.0044155	0.5	0.2	0.39	-12	17	41	8	3UTR
mmu-miR-214-3p	NM_177378	Rnf150	ACAGCAGGCACAGACAGGCAGT#CTGTCACTCTGGACCCTGCTGTG#((((((((..((((..(((((.#)))))..))))...)))))))).	1747	1770	1	-26	1	0.0004419	0.4	2	2.37	-7	17	23	8	CDS
mmu-miR-214-3p	NM_177378	Rnf150	ACAGCAGGCACAGACAGGCAGT#GCTGTCTTCCGAACCTGTCTGTAGAATTTTATTGCAAGGGTCAGAGCGCCCTCTGCTGG#.((((((((((((((((((((#)))).........)))))))))........................)))...))))).	7940	7999	1	-24	1	0.0001708	0.6	0.5	0.15	-4	21	59	9	3UTR

mmu-miR-214-3p	NM_177378	Rnf150	ACAGCAGGCACAGACAGGCAGT#GCAGGCCTGTTTTAAAACGTTGCTGTGTTCTGTTGC#.(((((((((((((((((.((#))..))))))..... ....)))))).)))))).	8160	8196	1	-24	1	0.0039189	0.5	0.3	0.2	-4	20	36	6	3UTR
mmu-miR-214-3p	NM_177390	Myo1d	ACAGCAGGCACAGACAGGCAGT#TTGCCAGTGTCTGCCCAGGAGGGAGCATGCCTGCTT#..((((((((((((((((((.#)))))..))))))..............))))))).	4398	4434	0.92308	-26	1	1.48E-06	0.4	2.5	3.4	-5	19	36	8	3UTR
mmu-miR-214-3p	NM_177390	Myo1d	ACAGCAGGCACAGACAGGCAGT#GCCTGAGCTGTTGAGGAGGAGGCCTATGCCGGCACAGGCTTGCTGTG#((((((((((((((.((((((...#))))))..))))).........................))))))))).	4463	4510	0.84615	-24	1	1.53E-06	0.4	3	3.4	-5	18	47	9	3UTR
mmu-miR-214-3p	NM_177391	Pheta2	ACAGCAGGCACAGACAGGCAGT#TTGCTCTGTGTGTGTGTGTGTGTG#((((((.(((((.((((((((.#)))).)))).))))).))).))).	1185	1209	1	-24	0	0.035005	0.5	0.3	0.11	-7	19	24	5	3UTR
mmu-miR-214-3p	NM_177391	Pheta2	ACAGCAGGCACAGACAGGCAGT#GCTGCCCTGTCCATGTATAGTGCTGG#.(((((...(((((((((((((#)))))).)))))..)))....))))).	1516	1542	0.92308	-23	0	0.0001307	0.4	0.3	0.07	-9	18	26	6	3UTR
mmu-miR-214-3p	NM_177407	Camk2a	ACAGCAGGCACAGACAGGCAGT#GCCTGAGAATCTGTTGCTGGCCTCGAAGCTCAAGGGCGCTGCTGTG#((((((((((((((((((((...#))))))....)))))).....................)).))))))).	557	603	0.80769	-24	1	4.38E-05	0.4	3.9	4.13	-4	19	46	7	CDS
mmu-miR-214-3p	NM_177407	Camk2a	ACAGCAGGCACAGACAGGCAGT#GCTGCCTCTCCGTGCTTTTGG#.(((.((((((.(((.(((((((#))))))).)).))))))))).	3329	3350	0.92308	-23	0	0.0175643	0.5	1.5	1.25	-12	18	21	9	3UTR
mmu-miR-214-3p	NM_177407	Camk2a	ACAGCAGGCACAGACAGGCAGT#CTGCGTCCTTGCTTCGCAGAGATCCGCTCTTTGTCCGTGGAATGTGGCTGCTGG#.((((((.((((((((((.(((#.))).))))))).......)))).))))))).	1602	1656	0.84615	-23	1	0.0008487	0.5	0.5	1.81	-6	19	30	6	3UTR
mmu-miR-214-3p	NM_177410	Bcl2	ACAGCAGGCACAGACAGGCAGT#GCTGCTCCTTGGGCACCCGCGGCCCCAGCGCGTCCGTGCCTGCA#...((((((((.((((..((...#.))..))).)))))))).	298	342	1	-25	0	0.0004201	0.4	4.3	3.14	-10	13	18	8	5UTR
mmu-miR-214-3p	NM_177460	Parp16	ACAGCAGGCACAGACAGGCAGT#CTGCGGGTGAAGTACCTGCTGG#.((((((((.((..(((..(((((.#))))..))...)).))))))).	1070	1092	0.84615	-21	1	1.22E-05	0.4	3.7	3.12	-10	15	22	7	CDS

mmu-miR-214-3p	NM_177460	Parp16	ACAGCAGGCACAGACAGGCAGT#CCTGTATCTGCTGCTGCTGC#.(((((.(((((((((((....#))))..)))).)))))))).	1171	1191	1	-20	0	0.0029457	0.5	2.6	2.35	-5	16	20	8	CDS
mmu-miR-214-3p	NM_177561	Usp46	ACAGCAGGCACAGACAGGCAGT#GCTGCCCATGATTTTGGCCCTGCA#...(((((..((((((((((((#))))))..))..))))..))))).	863	887	1	-21	0	0.0019127	0.5	-0	0.35	-9	17	24	6	CDS
mmu-miR-214-3p	NM_177561	Usp46	ACAGCAGGCACAGACAGGCAGT#ACTGTCTTTTCAACCCTGCTGA#.(((((((....((.(((((((#))))))).))....))))))).	2074	2096	0.84615	-21	1	0.0175198	0.5	4.1	4.5	-13	16	22	7	3UTR
mmu-miR-214-3p	NM_177582	Mlxip	ACAGCAGGCACAGACAGGCAGT#GCTCCTAAACCTGAGCCTGTCTCCTTGGTGTTGAAGAATGCCTGCA#...(((((((.((((((((...#.))))))))................))))))).	1824	1870	0.92308	-25	0	0.0035418	0.5	2.5	1.83	-9	15	33	8	CDS
mmu-miR-214-3p	NM_177618	Wscd1	ACAGCAGGCACAGACAGGCAGT#ACTGCTTGTCTGACATCCCATAAATGTGTGTGCTGTG#((((((.((((((((((((((#)))))))))))..............)))).)))))).	2684	2721	1	-31	0	0.0031171	0.6	0.4	-0.2	-5	21	37	11	3UTR
mmu-miR-214-3p	NM_177618	Wscd1	ACAGCAGGCACAGACAGGCAGT#ACTGCCAGGATGCCTGTGCTGAGCGGTCTTATGTCTATGCTGG#.((((((((((((.((((((((#))))))....)).)))))))................))))).	902	945	0.84615	-25	1	0.0001596	0.4	2.4	2.4	-5	20	43	7	CDS
mmu-miR-214-3p	NM_177629	Fam216b	ACAGCAGGCACAGACAGGCAGT#CTGGGTTACATCACTCAACAGGAGGCCTTGTCTTGTGCTGCTGTG#((((((.((((((((((((((...#.)))).)))).))))))))))).	301	346	0.92308	-28	0	2.66E-06	0.5	0.1	-0.2	-5	18	22	10	CDS
mmu-miR-214-3p	NM_177639	Dlgap1	ACAGCAGGCACAGACAGGCAGT#CTGCTCTGGGACTTGCTGAATGTCACCTGAAGGCCCCTGTGTCTGCTT#..((((((((((((...(((...#.))).)))))))))))).	11	59	1	-24	1	0.0005504	0.5	0.3	0.48	-7	14	17	11	5UTR
mmu-miR-214-3p	NM_177639	Dlgap1	ACAGCAGGCACAGACAGGCAGT#CTGCATCTGTGCCCTCCCTCGCAGCTGTA#(((((.((((((((...((((.#)))).))))))))..........))))).	3721	3750	1	-23	0	0.0030793	0.4	0.3	1	-7	17	29	8	3UTR
mmu-miR-214-3p	NM_177646	Dgkd	ACAGCAGGCACAGACAGGCAGT#CTGCCTGTGAGTGCCAAGTGTA#(((...(((((..((((((((.#))))))))..)))))...))).	740	762	1	-27	0	0.0170572	0.5	2.9	3.63	-18	16	22	8	CDS

mmu-miR-214-3p	NM_177646	Dgkd	ACAGCAGGCACAGACAGGCAGT#GCCTCAGTGGCCCTCACCAGAATGTTCTCTGTGCCTGCTA#..((((((((((((.((((...#)))).......................))))))))))))).	4116	4156	0.84615	-26	1	0.0033658	0.4	0.5	0.03	-6	16	40	12	3UTR
mmu-miR-214-3p	NM_177646	Dgkd	ACAGCAGGCACAGACAGGCAGT#TTGCCCTGTGTGTGGTTGTTGC#.((((((.((((.((((((((.#))))).))).)))).)))))).	5109	5131	0.84615	-26	1	0.0030704	0.5	-1	-0.5	-7	18	22	6	3UTR
mmu-miR-214-3p	NM_177660	Zbtb10	ACAGCAGGCACAGACAGGCAGT#CTGTGTGTGTGTGTGTGTGTGTG#((((((.((((((.((((.((((.#)))).))).))))).))).))).	6194	6217	1	-24	0	0.1919124	0.5	-0	0.54	-7	18	23	5	3UTR
mmu-miR-214-3p	NM_177660	Zbtb10	ACAGCAGGCACAGACAGGCAGT#CTGTTTGTCTGTGTA#.......((((((((((((((.#)))))))))))))))).	7376	7391	1	-23	0	0.0104741	0.7	1.9	2.5	-17	14	15	14	3UTR
mmu-miR-214-3p	NM_177672	Tmem201	ACAGCAGGCACAGACAGGCAGT#GCTGAGCCTGTCACCCTGCCTGTTGG#.((((((((((..((((((((((#)))..)))))))....))))))))).	3087	3113	1	-28	1	0.0012852	0.4	3.1	3.51	-10	19	26	9	3UTR
mmu-miR-214-3p	NM_177687	Crebl2	ACAGCAGGCACAGACAGGCAGT#TTGCTTGTCTGTGAGCCATTCCAGCATAAGCTGTG#(((((.(((((((((((((((.#))))))))))))))..)))............))))).	1118	1153	1	-26	0	3.80E-06	0.6	1.1	1.29	-6	20	35	12	3UTR
mmu-miR-214-3p	NM_177694	Ano5	ACAGCAGGCACAGACAGGCAGT#ACAGCCTGTCAGTGTTCCTGATAGCTGA#.((((((((((.(((((((.((#)).)))))))).)))..)))....)))).	2514	2542	0.92308	-23	1	0.003515	0.5	1.4	1.28	-7	19	28	7	CDS
mmu-miR-214-3p	NM_177694	Ano5	ACAGCAGGCACAGACAGGCAGT#GCTGCTTTACTGCTGTCTGTC#...((((((((((..(((((((#)))))))..))).)))))))).	7144	7165	0.84615	-22	0	0.0005445	0.6	-0	-0.2	-8	17	21	7	3UTR
mmu-miR-214-3p	NM_177696	Gdpd4	ACAGCAGGCACAGACAGGCAGT#TGCCTGGTAATAGCATTCATCTTGTCTGTGCTTTTTCTGTT#((((.((((((((((((....#.)))))))))))))...)))).	303	344	0.92308	-23	0	0.0028561	0.6	0.2	0.79	-8	17	22	13	CDS
mmu-miR-214-3p	NM_177696	Gdpd4	ACAGCAGGCACAGACAGGCAGT#TTGCCTAGTCATTGTCCTGTGCTCCTTCTTACTTGCTGTT#((((((((((((((((((((.#))))))).))).......))))))..........)))))).	548	588	0.84615	-23	1	0.0780883	0.5	-0	0.77	-4	21	40	6	CDS
mmu-miR-214-3p	NM_177700	Atmin	ACAGCAGGCACAGACAGGCAGT#CTGCCTGGGGGGCTCTGTGCTAAGTAGATCATGGATTCATAGCTGG#.((((.(((((((((((((((.#)))))))))......))))))))...................)))).	4206	4252	0.84615	-25	0	0.0071438	0.5	0.1	0.16	-6	19	46	8	3UTR

mmu-miR-214-3p	NM_177708	Rtn4rl1	ACAGCAGGCACAGACAGGCAGT#GCTGCTGTTGCTGCTCGCTGG#.((((.((((..(((((.(((((#)))))))))..)))).)))).	318	339	0.84615	-23	0	4.59E-05	0.4	3.8	1.96	-12	17	21	9	CDS
mmu-miR-214-3p	NM_177718	Mb21d2	ACAGCAGGCACAGACAGGCAGT#ACTGCCTGGTCAACAAGATGTGCCCTAATTATTTCATCCCTCAGTGCAACATGCTGG#.((((((((((((((((((((#)))))))).)).......))))))..........................))))).	1134	1191	0.84615	-26	1	0.0002064	0.5	0.2	0.2	-4	21	57	8	CDS
mmu-miR-214-3p	NM_177718	Mb21d2	ACAGCAGGCACAGACAGGCAGT#ACTGAGAGTCCTAAGAACCTGCGGCCCTGTGCATGCTGG#.(((((.((((((....((((.#.))))....)))))).))))).	1938	1977	0.92308	-25	0	2.61E-05	0.5	3.1	4.1	-7	15	22	6	3UTR
mmu-miR-214-3p	NM_177726	Tgm6	ACAGCAGGCACAGACAGGCAGT#GCTGGCTCTGTGCTTGA#....((((((((((..(.((((#)))).))))))))))).	1622	1639	0.84615	-22	0	1.42E-06	0.3	1	0.85	-11	15	17	11	CDS
mmu-miR-214-3p	NM_177735	Tmem130	ACAGCAGGCACAGACAGGCAGT#GCCTGCCCCTGCCTGCA#...(((((((..(.((((((...#)))))).)..))))))).	215	232	0.92308	-24	0	5.94E-05	0.4	0	-0.3	-14	13	17	7	CDS
mmu-miR-214-3p	NM_177735	Tmem130	ACAGCAGGCACAGACAGGCAGT#TGCTTGTGTGTGTGTAGTGTA#(((..(.((((((.(((((((((..#))))))).))))).)..))).	2187	2208	0.92308	-23	0	0.0127122	0.5	2.4	1.39	-14	16	21	7	3UTR
mmu-miR-214-3p	NM_177736	Lrrc61	ACAGCAGGCACAGACAGGCAGT#CTGTTTACGAGGAAGGCTGTGAGCTCTCAGCTTCCTGCTCTGGCCACCATGCCTGCTGG#.((((((((((((((((((.(((#.))).)))).))))......	2769	2828	1	-29	1	7.01E-06	0.4	0	0.13	-4	20	31	9	3UTR
mmu-miR-214-3p	NM_177736	Lrrc61	ACAGCAGGCACAGACAGGCAGT#GCTGCAGTGTCTGGCTGGGTTGC#.((((.(((.((((((.(((((#)))))..)))))))))..)))).	773	796	1	-24	0	0.0001608	0.3	2.8	1.29	-7	18	23	9	CDS
mmu-miR-214-3p	NM_177771	Klhl18	ACAGCAGGCACAGACAGGCAGT#CTGCCCCTCAGTTTGCCTGCTGA#.((((((((..(((..(((((.#)))))..))....))))))))).	3802	3825	0.92308	-26	1	2.67E-06	0.4	2.8	2.96	-11	16	23	9	3UTR
mmu-miR-214-3p	NM_177771	Klhl18	ACAGCAGGCACAGACAGGCAGT#ATTGCCTGGGTGTGCGCCAGTTTGCCGAGACGATGATGTGTGCTGTG#((((((((((((..((((((((#))))))))..))))).)......................)))))).	515	562	0.92308	-26	1	0.0111414	0.5	0	-0	-6	20	47	8	CDS
mmu-miR-214-3p	NM_177771	Klhl18	ACAGCAGGCACAGACAGGCAGT#TTGCCCTCTGCTGCCTGCCTGG#.((((((((((((((..((((((.#))))).)))).))))))).)).	3522	3544	0.92308	-26	1	0.0011452	0.4	4.2	3.72	-5	18	22	7	3UTR

mmu-miR-214-3p	NM_177819	Fam135b	ACAGCAGGCACAGACAGGCAGT#GCTGGCACTTAACAAGCTAACCAAGTCTGTGTCTGCA#...(((((((((((((.((.(((#.)))..))..))))))))))))).	2465	2502	0.92308	-25	0	0.0005687	0.4	-0	0.21	-7	17	23	12	CDS
mmu-miR-214-3p	NM_177819	Fam135b	ACAGCAGGCACAGACAGGCAGT#CTTGTCTGTGTCTTGCA#...((((((((((((((....#)))))))))))))).)).	3275	3292	0.84615	-24	0	0.0005807	0.5	3.3	3.2	-7	15	17	13	CDS
mmu-miR-214-3p	NM_177838	Fam163a	ACAGCAGGCACAGACAGGCAGT#ATTGCTGTCCTGTGCTACTGTAGGCTCCAGTATTACTGCTGC#.(((((((((((((((.(((((#))))))))).)))))...................)))))).	507	549	0.92308	-23	1	9.57E-05	0.5	-1	0.29	-4	20	42	9	CDS
mmu-miR-214-3p	NM_177838	Fam163a	ACAGCAGGCACAGACAGGCAGT#GCTCCTGTCCTCCTGTCTACTGCTGTG#(((((((..(((((((((.(((#)))))))))))....)))...))))))).	345	372	0.84615	-23	1	0.0103228	0.5	-0	-0.1	-7	19	27	9	5UTR
mmu-miR-214-3p	NM_177878	Mblac1	ACAGCAGGCACAGACAGGCAGT#TGTGCGTGTGTGCCTGTG#...(((((((((.((..(((..#)))..)).))))))))).	1127	1145	0.92308	-22	0	0.0003681	0.6	-0	0.02	-8	14	18	9	3UTR
mmu-miR-214-3p	NM_177993	Hbp1	ACAGCAGGCACAGACAGGCAGT#CTGTGCCTGCA#...((((((((((.........#)))))))))).	1511	1522	1	-21	0	0.0043493	0.6	2.2	2.87	-8	10	11	10	3UTR
mmu-miR-214-3p	NM_177993	Hbp1	ACAGCAGGCACAGACAGGCAGT#CCTGTCTGGTACTTGCTT#..((((((..((((((((....#))))))))...)))))).	2038	2056	0.92308	-22	1	0.0015005	0.6	4.8	3.74	-7	14	18	8	3UTR
mmu-miR-214-3p	NM_178076	Mcf2l	ACAGCAGGCACAGACAGGCAGT#ACTGATGAAATCATGCACCAGGACATCGTCCCGCTCTGTGCCGCTGA#.((((.((((((((..((....#.))..))))))))))))).	140	187	1	-23	0	0.0005228	0.4	4.2	1.94	-7	14	18	12	CDS
mmu-miR-214-3p	NM_178076	Mcf2l	ACAGCAGGCACAGACAGGCAGT#CTCCCTGTCTCCTCACCCTGCTGC#.(((((((...((((((((.((.#)).))))))))......))))))).	5071	5095	1	-23	1	0.1218334	0.4	-0	0.23	-9	16	24	7	3UTR
mmu-miR-214-3p	NM_178194	H2bc6	ACAGCAGGCACAGACAGGCAGT#ACTTCAGCCTGACTGTCTGCTGTGCACTGGCATGTGTACCTCCAAGCCTGCTGC#.((((((((((((.((((((((#.)))))))))))))))))).....................	2349	2403	1	-30	1	0.004505	0.5	3	2.08	-5	20	44	12	3UTR
mmu-miR-214-3p	NM_178378	Iqcg	ACAGCAGGCACAGACAGGCAGT#GCTGCCACTCTGCGGTCTGCTGG#.((((((((.((((..((((((#))))))..))))..)))))))).	3831	3854	0.84615	-30	1	0.0003728	0.4	0.2	-0.1	-6	18	23	8	3UTR

mmu-miR-214-3p	NM_178378	Iqcg	ACAGCAGGCACAGACAGGCAGT#ACTGACCTGCTGTGTGCTGA#.((((..((((((.((((((((#)))).)))))))))))))).	3412	3432	1	-25	0	8.19E-06	0.4	1.4	1.05	-8	18	20	14	3UTR
mmu-miR-214-3p	NM_178576	Cpsf4	ACAGCAGGCACAGACAGGCAGT#GCTGCTTGTCTGGGCTTGCTTGTG#((((((((.((((((((((((#)))))))))))).))))))).)).	1356	1380	0.84615	-33	1	6.13E-05	0.4	-0	0.06	-5	21	24	12	3UTR
mmu-miR-214-3p	NM_178625	Tmem209	ACAGCAGGCACAGACAGGCAGT#ACTGCGTGTGCGCGAGGGCCTGTGTGCTGG#.((((..(((((((((.(((((#))))).)))..........))))))	2411	2441	0.84615	-22	0	1.65E-05	0.5	3.9	3.55	-7	18	30	10	3UTR
mmu-miR-214-3p	NM_178659	Jmjd4	ACAGCAGGCACAGACAGGCAGT#TGCCTGGCTGAGCCTGCA#...((((((.(((.((((((..#)))))).))).)))))).	3842	3860	0.92308	-28	0	4.88E-05	0.5	-0	0.12	-9	15	18	6	3UTR
mmu-miR-214-3p	NM_178659	Jmjd4	ACAGCAGGCACAGACAGGCAGT#GCTGTGGCTGAGAACAGGCCTCTGAGAGCCTGCTGTT#(((((((((.((((..(((.((#.))..)))))))...))))))))).	2555	2592	0.84615	-25	1	1.26E-05	0.4	0.2	0.28	-6	18	25	9	3UTR
mmu-miR-214-3p	NM_178659	Jmjd4	ACAGCAGGCACAGACAGGCAGT#GCTGCCTAACCTGCCCTGCGTGTT#(((((((..(((..(((((((#)))))))...))).))))).))).	352	376	0.92308	-23	1	0.0011119	0.4	3.2	2.29	-8	18	24	7	CDS
mmu-miR-214-3p	NM_178665	Lpp	ACAGCAGGCACAGACAGGCAGT#CTGCCTCCCTTTAACCTCTCTCTCTACTGTGCCAACTAATGCTGC#.((((((((((((..((((((.#))))))....................)))))))......))))).	6954	6999	0.84615	-24	1	0.058855	0.6	-0	-0.3	-5	18	45	7	3UTR
mmu-miR-214-3p	NM_178665	Lpp	ACAGCAGGCACAGACAGGCAGT#ACTTTGGCCGTTGTGCCCGCTGTG#(((((.((((((.((.(((...#.))))))))))).))))).	1787	1811	1	-25	0	0.000118	0.5	4	3.66	-7	16	19	11	CDS
mmu-miR-214-3p	NM_178682	Susd6	ACAGCAGGCACAGACAGGCAGT#AGTGTCTGCTGTCCCTGCTGG#.(((((((.((((.((((((.(#).)))))))))).))))))).	2963	2984	1	-28	1	1.06E-05	0.4	0.2	0.44	-6	18	21	10	3UTR
mmu-miR-214-3p	NM_178682	Susd6	ACAGCAGGCACAGACAGGCAGT#GCTAGAGCCTTCTCTGTGGGGCCTGCTGG#.(((((((((((((.((((...#.))))..)))))...)))))))).	4500	4529	0.92308	-28	1	6.38E-09	0.5	-1	0.44	-5	17	24	8	3UTR
mmu-miR-214-3p	NM_178682	Susd6	ACAGCAGGCACAGACAGGCAGT#GCCTGCTGTGCCACTGCTC#..(((((((((((.(((((...#)))))))))))))..)))).	2353	2372	1	-28	1	0.0061675	0.4	1	0.74	-6	16	19	12	3UTR
mmu-miR-214-3p	NM_178684	Mapk1ip1l	ACAGCAGGCACAGACAGGCAGT#GCTACCTGAACAGTCCTCTGCCAAACCGCCTGCTGTG#(((((((((.......(((((.#.))))).....))	165	202	1	-25	1	0.0123796	0.5	1.6	1.94	-14	14	21	9	CDS

mmu-miR-214-3p	NM_178684	Mapk1ip1l	ACAGCAGGCACAGACAGGCAGT#CTGTGTGTGTGTGTGTGTGTGTGTG#((((((.(((((.(((.((((.#)))).))).))))).))).))).	1336	1359	1	-24	0	0.2167066	0.6	0	0.23	-7	18	23	5	3UTR
mmu-miR-214-3p	NM_178685	Pcdh20	ACAGCAGGCACAGACAGGCAGT#ACTGGCTTGTTCTTTCACTGAAGTGCCTGCTA#..(((((((((((((((((((#)))).))))).)).........)	4399	4431	1	-25	1	0.0004048	0.6	3.6	2.94	-5	20	32	9	3UTR
mmu-miR-214-3p	NM_178685	Pcdh20	ACAGCAGGCACAGACAGGCAGT#TTGCCTTTTGCTTCTGGACGTGCTGG#.(((((.(..((((.((((((.#))))))......))))..).))))).	648	674	0.84615	-21	0	0.001312	0.5	-0	-0	-8	16	26	6	CDS
mmu-miR-214-3p	NM_178704	Dpy19l3	ACAGCAGGCACAGACAGGCAGT#ATTGCCTGTTTGTGGCATCTGTTGG#.((((((((((((((((((((#))))))))))))))...))))))).	7564	7589	0.84615	-30	1	0.0085819	0.6	-0	0.03	-4	21	25	14	3UTR
mmu-miR-214-3p	NM_178704	Dpy19l3	ACAGCAGGCACAGACAGGCAGT#GCTTAATGAGGCTGTAGTGCCTGCTGC#.((((((((((..((((.(...#.).)))).)))))))))).	3912	3939	1	-28	1	5.77E-07	0.4	0.2	0.94	-8	15	19	10	3UTR
mmu-miR-214-3p	NM_178704	Dpy19l3	ACAGCAGGCACAGACAGGCAGT#GCTGCCTGCTCCTCGTCTGCA#...((((((...((((((((((#)))))))).))...)))))).	1122	1143	0.92308	-25	0	0.0017004	0.5	-0	0.12	-14	16	21	8	CDS
mmu-miR-214-3p	NM_178743	Slc26a11	ACAGCAGGCACAGACAGGCAGT#CTGCTACCACTGTGTGTGACGTTCCTGCTGTC#((((((((((((.((((((((.#))))).....)))).))))....	1373	1405	1	-27	1	0.002479	0.4	2.3	2.27	-5	20	32	8	CDS
mmu-miR-214-3p	NM_178743	Slc26a11	ACAGCAGGCACAGACAGGCAGT#CTGCCTATGCTGTGCTGCTT#..((((.((((((((.((((((.#)))))).).)))))))))).	429	449	0.92308	-24	0	0.0002471	0.4	2.8	2.88	-7	17	20	10	CDS
mmu-miR-214-3p	NM_178743	Slc26a11	ACAGCAGGCACAGACAGGCAGT#CTGGCCTGGGTGCCTGACTA#..(((((((((...(((((((.#))).)))).))))))).)).	179	199	0.92308	-23	1	1.32E-05	0.4	1.8	1.49	-10	16	20	7	CDS
mmu-miR-214-3p	NM_178788	Dctd	ACAGCAGGCACAGACAGGCAGT#CCTGTGTCTCCTGCTGTG#((((((((.(.(.(((((....#))))).).))))))))).	1677	1695	0.84615	-21	1	0.0060064	0.4	0.4	-0.4	-9	15	18	9	3UTR
mmu-miR-214-3p	NM_178795	Ppip5k1	ACAGCAGGCACAGACAGGCAGT#GCTGCCTGGAGTTAACATTGTTTCCTGCTGG#.((((((((.(((..((((((((#))))))))..........)))..))))	42	73	0.84615	-26	1	3.85E-06	0.5	-0	1.21	-7	18	31	8	5UTR
mmu-miR-214-3p	NM_178795	Ppip5k1	ACAGCAGGCACAGACAGGCAGT#CTGCCTAGTCCTGCTTCTGTG#((((.((((((..((((((((((.#)))))).))).))))))))).	5050	5071	0.92308	-23	0	0.0006543	0.6	4.4	4.31	-14	18	21	9	3UTR

mmu-miR-214-3p	NM_178872	Trim36	ACAGCAGGCACAGACAGGCAGT#CTGTTTGTACTGTGTGATTCTTTGTTTTGTCTTGTCCTGCTGTG#((((((((((((((((((((.#)))))))).)))))....................)))))))).	2532	2576	0.92308	-28	1	0.0230877	0.5	3.7	2.96	-4	21	44	8	3UTR
mmu-miR-214-3p	NM_178872	Trim36	ACAGCAGGCACAGACAGGCAGT#CTGCCTGCCTCTGCCTCCCAAGTGCTGG#.(((((((((.((.(((((((.#))))))).)).))))).......)))).	3950	3978	0.84615	-26	1	3.00E-05	0.4	2.8	3.12	-7	18	28	7	3UTR
mmu-miR-214-3p	NM_178897	Tyw1	ACAGCAGGCACAGACAGGCAGT#GCTGTCACCTCTCTGGACCTGCCTGTG#((((((((..((((.((((((#))))...))).))))..))))).))).	296	323	0.92308	-24	1	0.0016521	0.5	2.1	2.14	-7	19	27	5	CDS
mmu-miR-214-3p	NM_178897	Tyw1	ACAGCAGGCACAGACAGGCAGT#CCGTCTGTGCCTTCCTGG#.(((.((((((((((.((....#))))))))))))..))).	387	405	1	-23	0	1.94E-05	0.5	2.6	2.62	-8	15	18	12	CDS
mmu-miR-214-3p	NM_178897	Tyw1	ACAGCAGGCACAGACAGGCAGT#AGTGTGTGTGTGTGTGTGTGTGTG#((((((.(((((.(((.(((.(#).))).))).))))).))).))).	3194	3218	1	-22	0	0.6155152	0.6	0.2	0.16	-7	18	24	5	3UTR
mmu-miR-214-3p	NM_178907	Mapkapk3	ACAGCAGGCACAGACAGGCAGT#GGATGTGTCCTGCTGTG#((((((((((((..(.......#)..)))).)))))))).	1888	1905	0.92308	-22	1	1.02E-05	0.5	4.4	3.05	-6	13	17	8	3UTR
mmu-miR-214-3p	NM_178919	Lmf2	ACAGCAGGCACAGACAGGCAGT#ACTGCTGGAACTGACTGTCTATGGGCTGCTGG#.((((((.(..((((((.((((#.)))).))))))...).)))))	1157	1189	0.92308	-25	1	5.24E-05	0.5	2.6	2.99	-10	17	24	6	CDS
mmu-miR-214-3p	NM_178919	Lmf2	ACAGCAGGCACAGACAGGCAGT#CTGTCTGCTGAGCCTGG#....(((((.(((.(((((((.#)))))))))).))))).	1077	1094	1	-24	0	0.0015867	0.4	3	2.45	-12	15	17	10	CDS
mmu-miR-214-3p	NM_178919	Lmf2	ACAGCAGGCACAGACAGGCAGT#ACTGCTCTGACCTACCACTATGAGACTCAGTGCCTGCC#...((((((((.((((((((((#))))).)))................)).)))))))).	798	836	0.82692	-24	0	0.0080893	0.4	0.9	0.65	-10	18	38	8	CDS
mmu-miR-214-3p	NM_178939	Pdrg1	ACAGCAGGCACAGACAGGCAGT#GCTGGCTTTCTGTAGTAGAGGTGCTGCCAGTCACCCTCTGTGCTTGCTC#..((((((((((((((.((((((#.)))))).)))......))))))))))).	979	1028	1	-28	1	0.0068607	0.5	2.2	1.69	-5	19	28	10	3UTR
mmu-miR-214-3p	NM_180599	Mfap3	ACAGCAGGCACAGACAGGCAGT#ACTGCCTGTCCATCTTACTGGTGGTCACTCTTGTGCCAGCTGC#.((((.(((((((((((((((#)))))))))))....................)))))).))))).	218	261	0.92308	-32	0	0.0063081	0.4	1	1.05	-6	20	43	10	CDS

mmu-miR-214-3p	NM_181058	Zbtb20	ACAGCAGGCACAGACAGGCAGT#ACCACCAGTCTGTTGCCTGTTGC#.((((((((((((((.((..((#))..)).)))))).)))))))).	190	213	1	-26	1	0.0002084	0.4	0.8	1.42	-4	18	23	8	5UTR
mmu-miR-214-3p	NM_181071	Tanc2	ACAGCAGGCACAGACAGGCAGT#ACTGCCCTGCCTCGTGCTTGTTGA#.((((((((((((.((((((((#)))))).)).)).)))))))))).	10287	####	0.84615	-28	1	0.0046531	0.5	-0	0.82	-5	20	24	10	3UTR
mmu-miR-214-3p	NM_181071	Tanc2	ACAGCAGGCACAGACAGGCAGT#TTGCTTGTTTGTTTTGTTCCTGACATTTGACTGATTCCTGCTGA#.(((((((.((((((((((((.#)))))))))))))......................))))))).	11228	####	0.92308	-24	1	0.0197082	0.5	-0	0.57	-5	19	44	12	3UTR
mmu-miR-214-3p	NM_181071	Tanc2	ACAGCAGGCACAGACAGGCAGT#ACTGCCTGCCCTGCTTTGCTA#..((((((((..(.(((((((((#)))))))))..))))).))).	8646	8667	0.92308	-23	1	0.0008741	0.5	1.3	0.71	-12	17	21	9	3UTR
mmu-miR-214-3p	NM_181413	Anks1	ACAGCAGGCACAGACAGGCAGT#CTGCGGGCTGTCTGCCTGCTGC#.((((((((..((((((((((.#))))...))))))))))))).	4744	4766	1	-28	1	8.56E-09	0.4	-0	-0.6	-8	18	22	14	3UTR
mmu-miR-214-3p	NM_181413	Anks1	ACAGCAGGCACAGACAGGCAGT#CTGCCTCTCTCTGATGCTTGTTGA#.(((((((((((((.((((((.#))))))...)))).)))))))))).	4273	4297	1	-27	1	0.0053076	0.4	0.3	0.16	-5	19	24	9	3UTR
mmu-miR-214-3p	NM_181413	Anks1	ACAGCAGGCACAGACAGGCAGT#CTGCCTTTGGCGCTTGTGTGAAACCAAGGCTGTGCCTGAACTGTG#((((((((((((..((((((.#))))))......................))))))))..)))).	5219	5264	0.84615	-26	1	0.000572	0.5	0.5	-0.1	-6	19	45	9	3UTR
mmu-miR-214-3p	NM_181444	Gprc5a	ACAGCAGGCACAGACAGGCAGT#ACCGCCGTCTTGCCTGGGGCTGC#.(((((((((.((((.(((.((#)).)))))))))))))...)))).	1322	1345	1	-22	1	4.71E-05	0.4	-0	-0.1	-7	18	23	12	3UTR
mmu-miR-214-3p	NM_181444	Gprc5a	ACAGCAGGCACAGACAGGCAGT#TGTCTTTTCTGAGCTGCCTGCTC#..(((((((((((.(((((..#)))))..))))...)))))))).	619	642	0.84615	-22	1	0.0001402	0.5	0.3	2.18	-6	17	23	8	CDS
mmu-miR-214-3p	NM_181444	Gprc5a	ACAGCAGGCACAGACAGGCAGT#CTGCTTCTCTTGCCTCCTGG#.(((.(((((.(((.((((((.#)))))).)))))))).))).	451	471	0.84615	-22	0	0.0004588	0.4	4.8	2.07	-11	17	20	8	CDS
mmu-miR-214-3p	NM_181444	Gprc5a	ACAGCAGGCACAGACAGGCAGT#GCTGCCGTCGGGGCTGTGGCTACCGTGGCTTGCATGTT#(((((((((.(((((.((((((#)))))))))................)))))))).))).	233	271	0.92308	-22	1	0.000117	0.4	1.4	1.98	-5	20	38	9	CDS

mmu-miR-214-3p	NM_181734	Ttpal	ACAGCAGGCACAGACAGGCAGT#ACTGCCTCCTGGAACGCCGTGCTGC#.((((((((.(((..(((((((#))))))).)))....))).))))).	965	990	0.92308	-24	1	0.0062847	0.4	1.9	2.85	-7	18	25	7	CDS
mmu-miR-214-3p	NM_181734	Ttpal	ACAGCAGGCACAGACAGGCAGT#CTGCCAGCCCATGCCTGCCTGC#.((((((((..(.(.(((((.#))))).).)..))))))).)).	1021	1043	1	-22	1	0.0101874	0.4	0.4	2.08	-11	16	22	7	CDS
mmu-miR-214-3p	NM_181734	Ttpal	ACAGCAGGCACAGACAGGCAGT#ACTGCAGTCTGTTCACTTTGTAAACCTGCTT#..((((((.((((((..(((((#))))).))))))............)))	3957	3988	0.92308	-22	1	0.0108516	0.5	0	-0	-6	17	31	6	3UTR
mmu-miR-214-3p	NM_181853	Trim66	ACAGCAGGCACAGACAGGCAGT#GCTGTCCTGTGTGTGTATGCCATCTCCTTTCCTTCCCTGCTC#..((((((((((.(((((((((#)))).))))).)))).....................)))))).	7467	7509	1	-26	1	0.6097228	0.5	0.6	1.17	-6	19	42	6	3UTR
mmu-miR-214-3p	NM_181853	Trim66	ACAGCAGGCACAGACAGGCAGT#GCTGCTATGCTGTGACCGCTGC#.((((.((((((.((((((((#))))))).))))))).)))))).	3446	3468	0.84615	-25	0	2.33E-05	0.4	0.3	0.18	-7	19	22	7	CDS
mmu-miR-214-3p	NM_181853	Trim66	ACAGCAGGCACAGACAGGCAGT#CTGCTGTCTGGCTCAGCTGG#.((((.(((.((((((.((((.#)))))))))))))..)))).	8316	8336	1	-25	0	8.20E-06	0.5	2.8	3.2	-7	17	20	13	3UTR
mmu-miR-214-3p	NM_182839	Tppp	ACAGCAGGCACAGACAGGCAGT#ACTGCCTGTCTGTCTGCTTGGTTCTCTCTCTGTA#(((((((((((((((((((((#))))))))))))..)))))..........)))).	4372	4406	0.84615	-30	1	0.0004307	0.5	-1	-0.3	-4	22	34	13	3UTR
mmu-miR-214-3p	NM_182839	Tppp	ACAGCAGGCACAGACAGGCAGT#ACTGTCTGTCAGGTCAGGATGGCCCTGCTGTC#((((((((..((((((((((((#))))))))))).........))..	4893	4925	1	-29	1	3.07E-06	0.4	-0	0.13	-6	20	32	10	3UTR
mmu-miR-214-3p	NM_182840	Emilin3	ACAGCAGGCACAGACAGGCAGT#GTCTCTCTGTGCCTATGTG#(((..(((((((((.((((...#)))).))))))))).))).	332	351	0.84615	-23	0	5.28E-05	0.4	0.8	-0	-12	16	19	9	CDS
mmu-miR-214-3p	NM_183016	Cdc42bpb	ACAGCAGGCACAGACAGGCAGT#CTGCCTGTTTGTCGCTGTG#...((((..((((((((((((.#)))))))))))..)))).	4478	4497	1	-26	0	0.0017244	0.5	3.8	3.1	-10	16	19	12	CDS
mmu-miR-214-3p	NM_183016	Cdc42bpb	ACAGCAGGCACAGACAGGCAGT#GCTGGCTCCCAGTGTGCCTGCA#...((((((((...((.((((#)))).)).....))))))))).	6108	6130	0.84615	-25	0	6.71E-06	0.4	2.1	2.79	-9	15	22	9	3UTR
mmu-miR-214-3p	NM_183037	Trim46	ACAGCAGGCACAGACAGGCAGT#GCTAGGCCTCTGGTGCCTGCTA#..((((((((....(((((((#)))..))))...))))))))).	3008	3030	1	-24	1	7.74E-06	0.4	1.4	2.11	-11	16	22	9	3UTR

mmu-miR-214-3p	NM_183087	Fam189a1	ACAGCAGGCACAGACAGGCAGT#GCCTTTGGCCTGCTGG#.((((((((.(((..((((...#)))))))))))))))).	1835	1851	0.92308	-25	1	2.69E-07	0.4	-1	-0.2	-7	15	16	15	3UTR
mmu-miR-214-3p	NM_183138	Tet3	ACAGCAGGCACAGACAGGCAGT#GCCTTGTTTTTGTTGTCCCTGCCTGCTGTG#((((((((((..(((((((...#)))).........)))..)))))))))).	8345	8375	0.92308	-27	1	0.0195214	0.5	2.2	3.7	-10	17	30	10	3UTR
mmu-miR-214-3p	NM_183138	Tet3	ACAGCAGGCACAGACAGGCAGT#TGCCTGTGGATCTGTTCAGCCTGCTC#..((((((((((((((((((..#)))))))....)))))...))))))).	10056	####	0.92308	-27	1	0.0256242	0.5	2.6	2.59	-5	18	26	7	3UTR
mmu-miR-214-3p	NM_183138	Tet3	ACAGCAGGCACAGACAGGCAGT#CTGCTGTGCCTGGTGC#.((.(((((((((.(((.....#)))))))))))).)).	2559	2575	1	-25	0	8.00E-06	0.4	-0	0.37	-8	14	16	12	CDS
mmu-miR-214-3p	NM_183140	Zfp691	ACAGCAGGCACAGACAGGCAGT#GCTGGCGACTCTGTGCTGCTGA#.(((((.(((((((..(.((((#)))).)...)))))))))))).	405	427	1	-26	0	0.0003562	0.4	0.2	0.13	-6	17	22	12	5UTR
mmu-miR-214-3p	NM_183141	Elfn2	ACAGCAGGCACAGACAGGCAGT#GCTGCCTGTTTGGCATGGTCATCGTGCTGG#.(((((.(..((((((((((((#))))))))))))).........).)))	1729	1759	0.84615	-27	0	5.38E-06	0.4	0.2	-0.3	-8	18	30	12	CDS
mmu-miR-214-3p	NM_183141	Elfn2	ACAGCAGGCACAGACAGGCAGT#CTGCACCCCACTTGTCTGGCTCCTGCTGTG#((((((((..((((((((....#.))))))))...)))))))).	4018	4048	0.84615	-27	1	0.0003331	0.3	-0	0.04	-6	16	21	8	3UTR
mmu-miR-214-3p	NM_183312	Synm	ACAGCAGGCACAGACAGGCAGT#GCAGCTGTGGCCTGTTGTT#((((((((((((.(..((...#)).)))))).)))))))).	2112	2131	1	-22	1	0.000184	0.6	-0	-0.3	-5	16	19	8	3UTR
mmu-miR-214-3p	NM_184053	Calu	ACAGCAGGCACAGACAGGCAGT#CTGCCATTGTCTGGGCAACGATAGAGTCCTGTGCTGTA#((((((.((.(((((((((((.#)))))..)))))).))..............)))))).	1612	1650	0.92308	-23	0	0.0017366	0.5	0.1	0.31	-7	19	38	6	3UTR
mmu-miR-214-3p	NM_184053	Calu	ACAGCAGGCACAGACAGGCAGT#TGCCTGTCCCTGTGCACAGCCTTTGCTT#..(((((((((((((((((..#))))))))..)))))......))))).	133	161	0.92308	-24	1	0.0001723	0.5	2.8	2.15	-5	18	28	8	CDS
mmu-miR-214-3p	NM_184053	Calu	ACAGCAGGCACAGACAGGCAGT#TGCTTTCTCTTGCCTGTTGA#.(((((((((.(((.(((((..#))))))))..))))))))).	1539	1559	1	-22	1	0.0005397	0.5	-0	0.23	-7	17	20	9	3UTR
mmu-miR-214-3p	NM_194340	Prr14l	ACAGCAGGCACAGACAGGCAGT#GCCCATGTACTGTGCCTGCC#...((((((((((((((...#)))..))).)))))))))).	4464	4484	1	-28	0	0.0003018	0.5	1.8	1.94	-7	16	20	10	CDS

mmu-miR-214-3p	NM_194340	Prr14l	ACAGCAGGCACAGACAGGCAGT#GCTGCCTGGTCTGTGTGTTCTC#..((.(.((((((((((((((((#)))))))).))))))).).)).	6584	6606	0.84615	-27	0	0.0001447	0.4	-0	-0.2	-11	18	22	8	3UTR
mmu-miR-214-3p	NM_194340	Prr14l	ACAGCAGGCACAGACAGGCAGT#GCTGCCGTCTGTCGTCTGA#....(((((((((((.((((((#)))))))))))).))))).	7381	7400	0.84615	-26	0	9.53E-07	0.3	0.2	0.32	-10	17	19	12	3UTR
mmu-miR-214-3p	NM_194340	Prr14l	ACAGCAGGCACAGACAGGCAGT#TGTCTGTCTTCCTGCTGC#.(((((((...(((((((((..#))))))))).))))))).	8845	8863	1	-26	1	0.0023959	0.5	0.1	0.29	-9	16	18	9	3UTR
mmu-miR-214-3p	NM_194341	Synrg	ACAGCAGGCACAGACAGGCAGT#GCTTCCCTGTCTCAGTCTGCTA#..(((((((..(((((((.(((#)))..)))))))..))))))).	6307	6329	0.92308	-23	1	3.83E-05	0.5	-0	0.3	-9	17	22	7	3UTR
mmu-miR-214-3p	NM_194341	Synrg	ACAGCAGGCACAGACAGGCAGT#TTGCCTTCTGTCCTGTGGTTGGTGTG#(((.(((.(((((((((((((.#))))))...))).)))).))).))).	6221	6247	1	-23	0	0.0015751	0.4	0.4	0.47	-9	19	26	6	3UTR
mmu-miR-214-3p	NM_194342	Sun2	ACAGCAGGCACAGACAGGCAGT#TGCCTCTGACGACTGTTGTCTGTGCCAACCTAAGAGATTCCCAGTGCTGG#.(((((((((((((((.(((((#.)))))))))))))))))..................))))).	2858	2908	0.92308	-29	1	5.78E-05	0.5	1.4	1.53	-4	20	40	15	3UTR
mmu-miR-214-3p	NM_197940	Wipf2	ACAGCAGGCACAGACAGGCAGT#TGTCTGTCTGTCTGTCTGTCTGTC#(((((((((((((((((((((..#))))))))))))..))))).)))).	6900	6924	0.84615	-28	1	0.0367675	0.5	-0	0.2	-4	20	24	11	3UTR
mmu-miR-214-3p	NM_197940	Wipf2	ACAGCAGGCACAGACAGGCAGT#GCTGCGCTGTCTGCCCTTCTGGAAACCCAACCTGCCATCAGCTGTA#(((((.((((((((((((((((#))))).)))))))...................)))).))))).	5097	5143	1	-27	0	0.0279144	0.5	0.9	0.85	-6	21	46	7	3UTR
mmu-miR-214-3p	NM_197940	Wipf2	ACAGCAGGCACAGACAGGCAGT#ACTGCTGATTGCTCAGGCACAGCTGTGTGTGCTGTG#((((((.((((((...((((((#))))))...............)))))).)))))).	7266	7302	1	-26	0	0.0004541	0.5	0.5	0.33	-7	18	36	6	3UTR
mmu-miR-214-3p	NM_197999	Ces2g	ACAGCAGGCACAGACAGGCAGT#GCTGCCTCCTGAGTGTAGTGATCTGTTGA#.((((((((((.((((((((#))))))).....)).).))).)))))).	1212	1241	0.84615	-23	1	0.0316293	0.5	0.2	0.09	-5	20	29	7	CDS
mmu-miR-214-3p	NM_198035	Zbtb39	ACAGCAGGCACAGACAGGCAGT#CTGTTCGGTCTGTGCCAGCA#...((.(((((((((.(((((.#)))))..))))))))).)).	1703	1723	0.92308	-26	0	2.45E-05	0.5	3.2	2.99	-9	16	20	9	CDS

mmu-miR-214-3p	NM_198035	Zbtb39	ACAGCAGGCACAGACAGGCAGT#CTGTCTGTCTGGGAGTGCG#...(((..(.((((((((((((.#))))))))))).)..))).	5544	5563	1	-24	0	0.0001748	0.4	-0	0.35	-12	15	19	11	3UTR
mmu-miR-214-3p	NM_198035	Zbtb39	ACAGCAGGCACAGACAGGCAGT#GCTTGCCTTGTGTGTGTTGTGTTGA#.(((((.(((((.(((((((((#))).)))).)).)))))..))))).	2408	2433	1	-23	0	0.0014615	0.5	1.9	0.76	-7	19	25	5	3UTR
mmu-miR-214-3p	NM_198101	Gmip	ACAGCAGGCACAGACAGGCAGT#GCTGACCGCAGTGGTGCTTGCTGTA#((((((((((((.....((((((#)))).))......))))))))))).	3595	3620	1	-26	1	2.55E-05	0.4	0.4	0.04	-10	17	25	11	3UTR
mmu-miR-214-3p	NM_198127	Abi2	ACAGCAGGCACAGACAGGCAGT#CTGCCTCTGCCTGCCGAGTGCTGG#.((((((((((((((..(((((.#)))))))))..))))...))))).	3213	3237	0.84615	-24	1	6.55E-05	0.4	-0	-0	-5	18	24	9	3UTR
mmu-miR-214-3p	NM_198127	Abi2	ACAGCAGGCACAGACAGGCAGT#GCCTGCTGTGTTTGG#....(((((((((.(((((...#))))))))))))))).	4278	4293	1	-24	0	0.0042277	0.7	0.6	0.51	-10	14	15	14	3UTR
mmu-miR-214-3p	NM_198163	Rab35	ACAGCAGGCACAGACAGGCAGT#GCTGCACACCCTCAGCCTGCTCTGGCTTTTGGCTCCTTCCTGCCAACCTGCTGC#.(((((((..(((((((((...#.))))).))))....................))	1061	1115	0.92308	-24	1	0.0043599	0.4	0.8	2.12	-6	16	41	7	3UTR
mmu-miR-214-3p	NM_198163	Rab35	ACAGCAGGCACAGACAGGCAGT#CTGCCTTCTAGATGCCTCTGC#.(((.(((((((((.((((((.#))))))))).).)))))))).	1366	1387	1	-23	0	0.0051031	0.5	0.2	0.24	-9	18	21	9	3UTR
mmu-miR-214-3p	NM_198248	Zbtb40	ACAGCAGGCACAGACAGGCAGT#GCTCCTGGGGGCAGGCCTGTGCCTGCATGTG#(((((((((((((.(..((...#.))..).)))))))))).))).	4640	4671	0.92308	-27	1	0.0038246	0.4	0.3	1.99	-5	16	22	10	3UTR
mmu-miR-214-3p	NM_198303	Eif5b	ACAGCAGGCACAGACAGGCAGT#CTGCTCTGTGTGCAGCCATGGCTGG#.((((.(((.((.((((((((.#)))).)))).))..)))...)))).	4387	4412	0.92308	-24	0	2.30E-06	0.5	1.5	1.62	-8	17	25	4	3UTR
mmu-miR-214-3p	NM_198303	Eif5b	ACAGCAGGCACAGACAGGCAGT#CTGCCTTTGTCTTTGATCATATGCTGC#.(((((((((.((((((((((.#))))))..)))).)).))...))))).	7002	7029	0.84615	-20	1	0.019092	0.5	1.3	1.81	-7	19	27	6	3UTR
mmu-miR-214-3p	NM_198421	Usp49	ACAGCAGGCACAGACAGGCAGT#CTCAGAATGCAGTGAAGATGAGATGAGCTTGTCTGAATGCCTGCTGTC#((((((((((((((((((((...#.)))))))))..)))))))))).	3156	3204	0.92308	-34	1	0.0095379	0.4	0.2	-0	-4	19	23	10	3UTR

mmu-miR-214-3p	NM_198599	Map6d1	ACAGCAGGCACAGACAGGCAGT#GCTCTCCTGCATACTTAAGTGGGAAAAGGGGCCATCTGTGCCACTGCTGC#.((((((((((((..(((...#.))).))))))))..))))).	2283	2333	0.92308	-26	1	0.0003534	0.4	0.1	0.14	-5	16	21	8	3UTR
mmu-miR-214-3p	NM_198599	Map6d1	ACAGCAGGCACAGACAGGCAGT#GCCGCGCTGTGCCGCTC#..(((.(((((((...(((...#)))...)))))))))).	226	243	1	-24	0	2.45E-07	0.3	0.2	0.1	-9	13	17	10	CDS
mmu-miR-214-3p	NM_198614	C2cd4c	ACAGCAGGCACAGACAGGCAGT#GCTGCGTGGGGCTGTGCCTGGTGC#.((.(((((((((.((.(((((#))))).))...))))))))).)).	1313	1337	0.84615	-31	0	6.57E-06	0.3	-0	0.02	-8	18	24	9	CDS
mmu-miR-214-3p	NM_198614	C2cd4c	ACAGCAGGCACAGACAGGCAGT#GCTCAGGCCCGCCTGCGTGTGCGCCTGCTGG#.((((((((((..(((((.((#.))..)))))..)))..)))))	1234	1265	0.92308	-28	1	1.37E-06	0.3	0	-0	-6	18	26	8	CDS
mmu-miR-214-3p	NM_198647	Tbc1d22b	ACAGCAGGCACAGACAGGCAGT#GGTGTGTGTGTGTGTGTGTGTGTG#((((((.(((((.(((.(((.(#).))).))).))))).))).))).	2489	2513	1	-22	0	0.2240756	0.5	0.3	0.31	-7	18	24	5	3UTR
mmu-miR-214-3p	NM_198683	Celf1	ACAGCAGGCACAGACAGGCAGT#GCTGCCTTTCTGATTTCCAGCTGC#.((((.((..((((.(((((((#))))))).))))....)).)))).	4008	4032	0.92308	-24	0	0.0028507	0.6	3.2	3.8	-9	17	24	7	3UTR
mmu-miR-214-3p	NM_198683	Celf1	ACAGCAGGCACAGACAGGCAGT#GCCTGCTGTGTGCCCACAGTCTGACTGC#.((((((((((((.(((((...#)))))))))))).........))).))).	3759	3787	0.92308	-23	1	0.0007356	0.4	1.9	0.84	-5	17	28	11	3UTR
mmu-miR-214-3p	NM_198683	Celf1	ACAGCAGGCACAGACAGGCAGT#CTGCCTGCCTGCGCTTT#.....((((.(((.(((((((.#))))))).))).)))).	3083	3100	1	-22	0	4.61E-05	0.5	1	1.05	-16	14	17	7	3UTR
mmu-miR-214-3p	NM_198899	Uggt1	ACAGCAGGCACAGACAGGCAGT#CTGCCTGTTGTCCTGTTC#..((((((.(((.((((((((.#))))))))))))))))).	2065	2083	0.92308	-25	1	8.85E-06	0.5	0.2	-0	-7	17	18	17	CDS
mmu-miR-214-3p	NM_198899	Uggt1	ACAGCAGGCACAGACAGGCAGT#ACTGGCCTTACCGTGCCTGTTT#..(((((((((.(..((((((((#))))).)))..).))))))))).	264	286	1	-23	1	0.0001096	0.5	0.2	-0.1	-10	17	22	9	CDS
mmu-miR-214-3p	NM_198899	Uggt1	ACAGCAGGCACAGACAGGCAGT#GCCTGTGCTTTCTGCTTGCC#...(((((((.((((((((...#)))))).))...))))))).	8709	8729	0.92308	-22	0	0.0045612	0.4	2.2	1.83	-9	15	20	7	3UTR
mmu-miR-214-3p	NM_198932	Pou2f1	ACAGCAGGCACAGACAGGCAGT#CTCCCTGTCTGTGGCTGTC#...((((.((((((((((.((.#)).)))))))))).)))).	7969	7988	1	-27	0	9.14E-05	0.4	0.2	0.98	-9	16	19	10	3UTR

mmu-miR-214-3p	NM_198932	Pou2f1	ACAGCAGGCACAGACAGGCAGT#GCTGCCCTAGTGTGCAGGAGACAGTGTCCTGCTGG#.(((((((((((....((((((#))))))....)))))............)))))).	6617	6652	0.92308	-25	1	2.93E-05	0.4	0.5	0.53	-7	17	35	6	3UTR
mmu-miR-214-3p	NM_198932	Pou2f1	ACAGCAGGCACAGACAGGCAGT#GCTTCCTTCTACTTCCCATGGGAACAGCTGTGCCTGTTGC#.((((((((((((..(((.(((#))).))).................)))))))))))).	3332	3372	0.92308	-24	1	0.0002413	0.5	-1	-0	-5	18	40	12	3UTR
mmu-miR-214-3p	NM_198932	Pou2f1	ACAGCAGGCACAGACAGGCAGT#ACTGCTTGGAAGTGGCTGTCCTGTT#(((((((.(((...((((((((#))))))))...))).)))..))))).	11385	####	1	-23	1	6.72E-05	0.5	3.3	3.11	-12	18	25	8	3UTR
mmu-miR-214-3p	NM_198933	Pou2f1	ACAGCAGGCACAGACAGGCAGT#CTCCCTGTCTGTGGCTGTC#...((((.((((((((((.((.#)).)))))))))).)))).	8041	8060	1	-27	0	9.14E-05	0.4	0.4	0.94	-9	16	19	10	3UTR
mmu-miR-214-3p	NM_198933	Pou2f1	ACAGCAGGCACAGACAGGCAGT#GCTGCCCTAGTGTGCAGGAGACAGTGTCCTGCTGG#.(((((((((((....((((((#))))))....)))))............)))))).	6689	6724	0.92308	-25	1	2.93E-05	0.4	0.2	0.48	-7	17	35	6	3UTR
mmu-miR-214-3p	NM_198933	Pou2f1	ACAGCAGGCACAGACAGGCAGT#GCTTCCTTCTACTTCCCATGGGAACAGCTGTGCCTGTTGC#.((((((((((((..(((.(((#))).))).................)))))))))))).	3404	3444	0.92308	-24	1	0.0002413	0.5	-0	0.04	-5	18	40	12	3UTR
mmu-miR-214-3p	NM_198933	Pou2f1	ACAGCAGGCACAGACAGGCAGT#ACTGCTTGGAAGTGGCTGTCCTGTT#(((((((.(((...((((((((#))))))))...))).)))..))))).	11457	####	1	-23	1	6.72E-05	0.5	3.9	3.77	-12	18	25	8	3UTR
mmu-miR-214-3p	NM_198934	Pou2f1	ACAGCAGGCACAGACAGGCAGT#CTCCCTGTCTGTGGCTGTC#...((((.((((((((((.((.#)).)))))))))).)))).	8113	8132	1	-27	0	9.14E-05	0.4	1.2	1.73	-9	16	19	10	3UTR
mmu-miR-214-3p	NM_198934	Pou2f1	ACAGCAGGCACAGACAGGCAGT#GCTGCCCTAGTGTGCAGGAGACAGTGTCCTGCTGG#.(((((((((((....((((((#))))))....)))))............)))))).	6761	6796	0.92308	-25	1	2.93E-05	0.4	0.1	0.49	-7	17	35	6	3UTR
mmu-miR-214-3p	NM_198934	Pou2f1	ACAGCAGGCACAGACAGGCAGT#GCTTCCTTCTACTTCCCATGGGAACAGCTGTGCCTGTTGC#.((((((((((((..(((.(((#))).))).................)))))))))))).	3476	3516	0.92308	-24	1	0.0002413	0.5	0.7	0.24	-5	18	40	12	3UTR

mmu-miR-214-3p	NM_198934	Pou2f1	ACAGCAGGCACAGACAGGCAGT#ACTGCTTGGAAGTGGCTGTCCTGTT#(((((((.(((...((((((((#))))))))...))).)))..)))).	11529	####	1	-23	1	6.72E-05	0.5	3.9	3.77	-12	18	25	8	3UTR
mmu-miR-214-3p	NM_199027	Zfp335	ACAGCAGGCACAGACAGGCAGT#GCTGCCTTCAAGTGCCCTGACTGC#.((((((((((.((.(((((((#)))))))))..))))).)).))).	3416	3440	0.84615	-24	1	0.0081867	0.3	1.4	1.46	-8	19	24	9	CDS
mmu-miR-214-3p	NM_199027	Zfp335	ACAGCAGGCACAGACAGGCAGT#GCCTTATCTGTGCCGCA#...((.((((((((.((((...#))))..)))))))))).	1675	1692	1	-24	0	0.0005592	0.4	2.1	0.85	-9	14	17	10	CDS
mmu-miR-214-3p	NM_199027	Zfp335	ACAGCAGGCACAGACAGGCAGT#GCCTGTTTGGCACTGAGGA#.(..(((((.((((((((...#))))))))))).)))..).	4203	4222	0.84615	-23	0	3.50E-05	0.3	2.4	2.67	-10	15	19	11	CDS
mmu-miR-214-3p	NM_199060	AF067061	ACAGCAGGCACAGACAGGCAGT#TTGCAATTAGGGAATCTTCTGTCAGCTCCTGCTGTT#((((((((..(.((((((....#.)))))).)..))))	754	790	0.84615	-21	1	1.64E-05	0.5	0	0	-10	15	20	8	3UTR
mmu-miR-214-3p	NM_199060	AF067061	ACAGCAGGCACAGACAGGCAGT#ACTGTGTTTGGAGCCCATGAACCCTGATGCCTGCTA#..(((((((((((.(((((...#.)))..))....)))	714	750	0.84615	-21	1	0.0034708	0.5	0	0	-6	16	25	8	3UTR
mmu-miR-214-3p	NM_199079	Ddx17	ACAGCAGGCACAGACAGGCAGT#GCCGCCATTTTGTGCAGTTGCTGG#.((((((((((((..(((.((#)).)))..)))))))..)))))).	179	203	0.92308	-23	1	0.0021992	0.3	1.4	1.75	-5	18	24	7	5UTR
mmu-miR-214-3p	NM_199079	Ddx17	ACAGCAGGCACAGACAGGCAGT#ATTGGCGTATTTGCTGCCTGCG#...((((((((((..(.((((#)))).)...)))).))))))).	925	947	0.84615	-20	0	0.0003933	0.5	-0	0.32	-7	16	22	7	CDS
mmu-miR-214-3p	NM_199080	Ddx17	ACAGCAGGCACAGACAGGCAGT#ACTGCATTCTTTGGCTGTGCCCTGAGGGTGCCTGCTC#..(((((((((...((((((((#.))))...))))...))))))))).	3785	3822	0.84615	-26	1	3.63E-06	0.5	4.2	3.9	-10	17	25	9	3UTR
mmu-miR-214-3p	NM_199080	Ddx17	ACAGCAGGCACAGACAGGCAGT#GCCGCCATTTTGTGCAGTTGCTGG#.((((((((((((..(((.((#)).)))..)))))))..)))))).	179	203	0.92308	-23	1	0.0021992	0.3	2.3	1.88	-5	18	24	7	5UTR
mmu-miR-214-3p	NM_199080	Ddx17	ACAGCAGGCACAGACAGGCAGT#TGTCCTGTCTTGTCTGTC#...(((((((.(((((((((..#)).))))))))))))))).	4042	4060	0.92308	-22	0	0.2279617	0.5	3	3.05	-9	16	18	14	3UTR
mmu-miR-214-3p	NM_199143	Znrf2	ACAGCAGGCACAGACAGGCAGT#GCTGTCCTGCTGTGCTGCGCTGTG#(((((.((((((((.((((((((#)))).)))))))))))..))))).	2244	2268	0.84615	-31	0	3.73E-05	0.3	0	-0.1	-7	20	24	11	3UTR

mmu-miR-214-3p	NM_199146	Trim30d	ACAGCAGGCACAGACAGGCAGT#AGTGACCTGCCCAATCTGTCTGGAGCTCTTGAAGGAATCTGTGAGTGCTGA#.(((((..(((((((((((((.#.)))))))..............))))))..))))).	270	321	0.84615	-25	0	0.0001514	0.5	-0	-0	-8	18	37	7	CDS
mmu-miR-214-3p	NM_199146	Trim30d	ACAGCAGGCACAGACAGGCAGT#ACTGCCCTGTGTGCCGAGTTC#..(((.((((((.(..((((((#)))))).).))))))..))).	395	416	0.94872	-21	0	0.0089171	0.5	-0	-0.1	-9	16	21	6	CDS
mmu-miR-214-3p	NM_199299	Jade2	ACAGCAGGCACAGACAGGCAGT#GCAGGCCGAGGCTGGCCTGCTGTG#((((((((((.(((...(((.((#))..)))....))))))))))).	3849	3873	1	-27	1	9.04E-09	0.3	1	0.63	-7	17	24	12	3UTR
mmu-miR-214-3p	NM_199299	Jade2	ACAGCAGGCACAGACAGGCAGT#ACTGCCTGGTGGTCTGACCTGTG#(((((((((.((..((((((((#)))))))).)))))))..)))).	4845	4868	0.92308	-24	1	5.63E-05	0.5	4.4	1.79	-7	19	23	8	3UTR
mmu-miR-214-3p	NM_199306	Wdtc1	ACAGCAGGCACAGACAGGCAGT#GCTTGCCTGGCCTGTTGTT#(((((((((.(((.(((((...#))))).)))))))))))).	2839	2858	0.92308	-27	1	0.0003133	0.3	3.8	3.72	-6	17	19	12	3UTR
mmu-miR-214-3p	NM_199306	Wdtc1	ACAGCAGGCACAGACAGGCAGT#GCTGCTCTGCTGCTTCTCCTGCTGC#.((((((((..(((.((((((((#))))).))))))).....))))))).	3850	3875	1	-25	1	0.0012107	0.4	1.9	1.48	-7	18	25	7	3UTR
mmu-miR-214-3p	NM_199306	Wdtc1	ACAGCAGGCACAGACAGGCAGT#GCTGTGTCAACTGTCTGGAGTGGAATGAGAAAGGAGACCTGCTGG#.((((((((((...((((((((#.))))))))..)))..............))))))).	561	606	0.84615	-25	1	0.0023752	0.5	0.9	1.01	-9	18	37	8	CDS
mmu-miR-214-3p	NM_199323	Tacc1	ACAGCAGGCACAGACAGGCAGT#CTGCTCAGCTGTTCCTGCTGTG#((((((((.((((...(((((.#)))))...)))).)))))))).	5056	5078	0.92308	-27	1	2.67E-05	0.5	0.7	0.48	-7	17	22	8	3UTR
mmu-miR-214-3p	NM_201230	Cep43	ACAGCAGGCACAGACAGGCAGT#ACAGCCATGGTTTGTGCCACTATGTGTCTGTGGCTGCTC#..(((((.((((((((((((..#.))))......)))))))).))))).	1522	1561	1	-27	0	0.0014486	0.5	-0	-0.1	-7	17	26	8	3UTR
mmu-miR-214-3p	NM_201230	Cep43	ACAGCAGGCACAGACAGGCAGT#GCTGGAATGTCCGCTCCTGCTGG#.((((((((..(.((((..((((#))))...)))).)..))))))).	1290	1313	0.92308	-23	1	0.0001657	0.5	0.8	0.85	-9	16	23	7	3UTR
mmu-miR-214-3p	NM_201395	Sall4	ACAGCAGGCACAGACAGGCAGT#GCTGCCTGTGTCGCTGGGGGCCAGCTC#..(((.(((.((((((((((((#))))))))))....)))...))).))).	1860	1887	0.84615	-24	0	4.58E-07	0.4	1.3	2.5	-8	18	27	9	CDS

mmu-miR-214-3p	NM_201395	Sall4	ACAGCAGGCACAGACAGGCAGT#CCTGGAGCAGATCCTGTGCCTGCA#...((((((((((....((...#.)).....)))))))))).	798	822	1	-23	0	8.34E-05	0.4	0.3	0.13	-8	12	19	10	CDS
mmu-miR-214-3p	NM_201396	Sall4	ACAGCAGGCACAGACAGGCAGT#GCTGCCTGTGTCGCTGGGGGCCAGCTC#..(((.(((.((((((((((((#)))))))))....)))...))).))).	825	852	0.84615	-24	0	4.58E-07	0.4	0.1	0.08	-8	18	27	9	CDS
mmu-miR-214-3p	NM_201396	Sall4	ACAGCAGGCACAGACAGGCAGT#ACTGCCTGTTTCCTAGTGG#.((..(((...(((((((((((#))))))))))))))..)).	1907	1926	1	-21	0	3.87E-05	0.4	3.7	2.25	-16	16	19	14	3UTR
mmu-miR-214-3p	NM_201622	Nr1h5	ACAGCAGGCACAGACAGGCAGT#ATTCCAGTCTGAGTCTGTTT#..(((((((.(((((.((.(((#))))).))))).))))))).	2438	2458	0.84615	-18	1	0.002787	0.6	2.2	1.91	-6	17	20	7	3UTR
mmu-miR-214-3p	NM_207205	Igsf3	ACAGCAGGCACAGACAGGCAGT#GCTGTCTGGCTGTGCTGGGTGTG#(((.(.(((((((.((((((((#)))))))).))))))).).))).	747	770	0.84615	-29	0	2.37E-06	0.4	1.8	1.61	-10	19	23	8	CDS
mmu-miR-214-3p	NM_207205	Igsf3	ACAGCAGGCACAGACAGGCAGT#TGTGTGTGTGTGCGTGCG#...(((.(((((.(((.(((..#))).))).))))).))).	5716	5734	0.92308	-25	0	0.0001348	0.5	0.2	-0.1	-9	14	18	5	3UTR
mmu-miR-214-3p	NM_207205	Igsf3	ACAGCAGGCACAGACAGGCAGT#GCCTGTCTGTTCTGGAGA#.(..((((.((((((((((...#))))))))))))))..).	3195	3213	1	-24	0	0.0001769	0.4	2	1.9	-9	15	18	14	CDS
mmu-miR-214-3p	NM_207652	Tsc22d1	ACAGCAGGCACAGACAGGCAGT#ACTGGCTGCTGCTGCTGCTGTC#((((((.((((((.(((.((((#)))).)))))).))))))))).	3515	3537	0.84615	-26	0	2.61E-05	0.5	0.6	1.37	-6	19	22	9	3UTR
mmu-miR-214-3p	NM_207659	Hook3	ACAGCAGGCACAGACAGGCAGT#AGTGTGTGTCTGTGTGTGTGTGTA#((((((.(((((((((.(((.(#).))).))))))))).))).))).	6478	6502	0.84615	-26	0	0.1359257	0.5	-0	0.05	-5	19	24	9	3UTR
mmu-miR-214-3p	NM_207659	Hook3	ACAGCAGGCACAGACAGGCAGT#GCATGTGTGTCTGTGTGTGTG#...(((.((((((((.(((((#)).))).))))))))).))).	6417	6438	0.92308	-25	0	0.0040269	0.5	-0	-0.1	-8	17	21	9	3UTR
mmu-miR-214-3p	NM_207659	Hook3	ACAGCAGGCACAGACAGGCAGT#TGCTAATTTCAGTGTCTGTAGCCTCTGCTGTT#((((((((((((((((((((..#))))........))))))).)))).	4912	4944	0.92308	-24	1	0.0030336	0.6	0	0.25	-4	20	32	7	3UTR
mmu-miR-214-3p	NM_207667	Fgf14	ACAGCAGGCACAGACAGGCAGT#GCTGCTCGCTCCACTGTCTGTGACCTTTAGCA#...(((((((((((((((((((#)))))........)))))))))).)	1643	1675	1	-24	0	9.42E-05	0.5	3.3	3.04	-7	19	32	9	3UTR

mmu-miR-214-3p	NM_207667	Fgf14	ACAGCAGGCACAGACAGGCAGT#TGCTTGTCTTATGCTGTC#((((((.....(((((((((..#)))))))))..)))))).	1276	1294	0.92308	-20	0	0.0010639	0.5	1.8	2.73	-13	15	18	9	3UTR
mmu-miR-214-3p	NM_207667	Fgf14	ACAGCAGGCACAGACAGGCAGT#CCTTGCCTGCTGA#.(((((((((.....(((....#)))))))))))).	1480	1493	1	-18	1	0.036681	0.6	2.7	1.52	-13	12	13	12	3UTR
mmu-miR-214-3p	NM_207675	Cadm1	ACAGCAGGCACAGACAGGCAGT#GCTGCCCATGTCTTAAATACGTAGAGCAGCGAAGACTAGAAACGTAGACTGTGACCCAGACGCTGTCGTCTGCTGTG#(((((((((...((((((((((#.)))))..........)))))))))))))).	2094	2171	0.84615	-23	1	0.0002798	0.5	1.5	1.26	-11	19	31	14	3UTR
mmu-miR-214-3p	NM_207676	Cadm1	ACAGCAGGCACAGACAGGCAGT#GCTGCCCATGTCTTAAATACGTAGAGCAGCGAAGACTAGAAACGTAGACTGTGACCCAGACGCTGTCGTCTGCTGTG#(((((((((...((((((((((#.)))))..........)))))))))))))).	2010	2087	0.84615	-23	1	0.0002798	0.5	1.5	1.26	-11	19	31	14	3UTR
mmu-miR-214-3p	NM_207680	Bcl2l11	ACAGCAGGCACAGACAGGCAGT#ACTGCGGTGGCTGTGTTTGCC#...((((((((((.((.(((((#)))))..)).)))))))))).	1729	1750	0.92308	-24	0	8.07E-06	0.4	-0	-0.6	-7	17	21	10	3UTR
mmu-miR-214-3p	NM_207681	Bcl2l11	ACAGCAGGCACAGACAGGCAGT#ACTGCGGTGGCTGTGTTTGCC#...((((((((((.((.(((((#)))))..)).)))))))))).	1561	1582	0.92308	-24	0	8.07E-06	0.4	-0	-0.6	-7	17	21	10	3UTR
mmu-miR-214-3p	NM_207682	Kif1b	ACAGCAGGCACAGACAGGCAGT#CTGCCCTGTGTGGCTCCTGCTGG#.(((((((..((.((((((((.#))))).))).))...))))))).	9008	9031	1	-28	1	6.64E-05	0.4	3.8	4.39	-7	17	23	7	3UTR
mmu-miR-214-3p	NM_207682	Kif1b	ACAGCAGGCACAGACAGGCAGT#TGTGTGTCTGGTGTCTGTCTGTC#((((((((((((((((.(((..#))).)))))).)))))).)))).	6257	6280	0.84615	-27	1	0.1367421	0.5	3.3	3.97	-4	19	23	6	3UTR
mmu-miR-214-3p	NM_207682	Kif1b	ACAGCAGGCACAGACAGGCAGT#TGTGTGTGTGTGTCTGTCTGTC#(((((((((((.(((.(((..#))).))).))))))))).)))).	6324	6346	0.92308	-27	1	0.311283	0.6	3.5	4.03	-5	18	22	8	3UTR
mmu-miR-214-3p	NM_207687	Espn	ACAGCAGGCACAGACAGGCAGT#GCTGCACTGTCTGCGCTACTTGGTGG#.((.(((((.(((((((((((#))))).))))))).)))...)).)).	325	351	1	-27	0	0.0001681	0.3	2.9	2.59	-8	19	26	7	CDS
mmu-miR-214-3p	NM_207688	Espn	ACAGCAGGCACAGACAGGCAGT#GCTGGAGTGCTCTCGCAAACCCCTGCTGTCGCCTGG#....(((((((((.((((....#.)))))))).))))	1823	1859	0.84615	-21	0	0.0024151	0.4	4	1.56	-10	13	16	8	3UTR

mmu-miR-214-3p	NM_207689	Espn	ACAGCAGGCACAGACAGGCAGT#GCTGGAGTGCTCTCGCAAACCCCTGCTGTCGCCTGG#....(((((((((.((((....#.)))))))).))))	1580	1616	0.84615	-21	0	0.0024151	0.4	4	1.56	-10	13	16	8	3UTR
mmu-miR-214-3p	NM_207690	Espn	ACAGCAGGCACAGACAGGCAGT#GCTGGAGTGCTCTCGCAAACCCCTGCTGTCGCCTGG#....(((((((((.((((....#.)))))))).))))	1553	1589	0.84615	-21	0	0.0024151	0.4	4	1.56	-10	13	16	8	3UTR
mmu-miR-214-3p	NM_207691	Espn	ACAGCAGGCACAGACAGGCAGT#GCTGGAGTGCTCTCGCAAACCCCTGCTGTCGCCTGG#....(((((((((.((((....#.)))))))).))))	1796	1832	0.84615	-21	0	0.0024151	0.4	4	1.56	-10	13	16	12	3UTR
mmu-miR-214-3p	NM_207692	Mapk9	ACAGCAGGCACAGACAGGCAGT#CTGTGCCTGTTGG#.((((((((((((.........#))))))))))))).	2662	2675	0.92308	-23	1	0.0004919	0.5	-0	-0.1	-6	12	13	12	3UTR
mmu-miR-214-3p	NM_213616	Atp2b4	ACAGCAGGCACAGACAGGCAGT#GCTGCCCAGGGAAGTCTGTGCCTTTCTGC#.(((.((((((((((.((((((#))))))).......)))))))))..))).	5349	5378	0.92308	-28	0	0.0582564	0.4	2.8	3.66	-8	19	29	10	3UTR
mmu-miR-214-3p	NM_213616	Atp2b4	ACAGCAGGCACAGACAGGCAGT#CTGCAGTTCGGCCTGCTGC#.((((((((.(.(((..((((.#)))).))).))))))))).	7500	7519	0.92308	-24	1	7.09E-05	0.5	0.3	1.97	-9	16	19	9	3UTR
mmu-miR-214-3p	XM_006495650	Creb1	ACAGCAGGCACAGACAGGCAGT#TTGTCCTGCTGAGCTTGCTGG#.((((((((.(((.(((((((.#))).))))))).)))))))).	2830	2851	0.92308	-26	1	8.12E-05	0.5	0	0	-6	18	21	8	3UTR
mmu-miR-214-3p	XM_006495650	Creb1	ACAGCAGGCACAGACAGGCAGT#GCTGGCCTGTCCCACTGCTGC#.((((((.....((((((((((#)))).))))))...)))))).	5689	5710	0.84615	-24	1	1.89E-08	0.4	0	0	-15	16	21	6	3UTR
mmu-miR-214-3p	XM_006495650	Creb1	ACAGCAGGCACAGACAGGCAGT#CTGTCTGTGCTAGCA#...((.(((((((((((.....#)))))))))))).)).	2870	2885	1	-23	0	0.0003018	0.6	0	0	-8	13	15	11	3UTR
mmu-miR-214-3p	XM_006495651	Creb1	ACAGCAGGCACAGACAGGCAGT#TTGTCCTGCTGAGCTTGCTGG#.((((((((.(((.(((((((.#))).))))))).)))))))).	2792	2813	0.92308	-26	1	8.12E-05	0.5	0	0	-6	18	21	8	3UTR
mmu-miR-214-3p	XM_006495651	Creb1	ACAGCAGGCACAGACAGGCAGT#GCTGGCCTGTCCCACTGCTGC#.((((((.....((((((((((#)))).))))))...)))))).	5651	5672	0.84615	-24	1	1.89E-08	0.4	0	0	-15	16	21	6	3UTR
mmu-miR-214-3p	XM_006495651	Creb1	ACAGCAGGCACAGACAGGCAGT#CTGTCTGTGCTAGCA#...((.(((((((((((.....#)))))))))))).)).	2832	2847	1	-23	0	0.0003018	0.6	0	0	-8	13	15	11	3UTR

mmu-miR-214-3p	XM_006496081	Abi2	ACAGCAGGCACAGACAGGCAGT#GCCTGCTGTGTTTGG#....(((((((((.(((((...#))))))))))))).	4474	4489	1	-24	0	0.0042277	0.7	0	0	-10	14	15	14	3UTR
mmu-miR-214-3p	XM_006496081	Abi2	ACAGCAGGCACAGACAGGCAGT#ACTGTCCTGTCATTCTGAGTCTACTGTG#((((.((((.((((((((((((#)))).))))))....)).)))).)))).	6568	6596	0.92308	-22	0	0.0630664	0.6	0	0	-9	20	28	6	3UTR
mmu-miR-214-3p	XM_006496084	Abi2	ACAGCAGGCACAGACAGGCAGT#GCTGCCTGGAAGTTCAGTGGCTCTGCCTGTTC#..((((((((.((.((((((((#))))))))............)).)	3399	3431	1	-24	1	0.0009319	0.5	0	0	-9	18	32	8	5UTR
mmu-miR-214-3p	XM_006496084	Abi2	ACAGCAGGCACAGACAGGCAGT#GCCTAAAGTCTGTGTTTCCTGG#.(((.((((((((((((((...#))))...)))))))))).))).	2064	2086	1	-24	0	4.04E-05	0.6	0	0	-8	17	22	10	5UTR
mmu-miR-214-3p	XM_006496084	Abi2	ACAGCAGGCACAGACAGGCAGT#GCCTGCTGTGTTTGG#....(((((((((.(((((...#))))))))))))).	8165	8180	1	-24	0	0.0042277	0.7	0	0	-10	14	15	14	3UTR
mmu-miR-214-3p	XM_006496818	Akt3	ACAGCAGGCACAGACAGGCAGT#TGTGCTGCTGTGCCCAATACTGCTGG#.(((((((((((.((((((..#))).))))))))))......))))).	1735	1761	0.84615	-24	1	2.86E-05	0.5	0	0	-5	18	26	10	5UTR
mmu-miR-214-3p	XM_006496818	Akt3	ACAGCAGGCACAGACAGGCAGT#ATTGTGTGTGTGTGTGTGTGTGTG#((((((.(((((.(((.(((((#))))).))).))))).))).))).	2361	2385	0.92308	-23	0	0.2464754	0.5	0	0	-7	19	24	5	5UTR
mmu-miR-214-3p	XM_006497170	Esrrg	ACAGCAGGCACAGACAGGCAGT#GCTGCCTGTATACAAACTCTGTCTCAGAAGGAGCTGTG#((((((((((.(((((((((((#))))))))).......)).)))))........))))).	2942	2980	0.92308	-23	1	0.0002872	0.5	0	0	-7	21	38	9	3UTR
mmu-miR-214-3p	XM_006497402	Pter	ACAGCAGGCACAGACAGGCAGT#CCTGACTCTGTGATCTGCA#...((((((((((((((....#))))..)))))).))))).	1342	1361	0.92308	-21	0	0.0096744	0.6	0	0	-7	15	19	6	3UTR
mmu-miR-214-3p	XM_006497531	Plxdc2	ACAGCAGGCACAGACAGGCAGT#ACTGCTGTCGTGTTTGGCTGC#.((((((((((.((((.(((((#)))))))))))))))))).))).	12730	####	1	-24	1	7.13E-05	0.7	0	0	-7	19	21	16	3UTR
mmu-miR-214-3p	XM_006497531	Plxdc2	ACAGCAGGCACAGACAGGCAGT#TTGCTAAGAAATTCTAGTTCTGTTTGTGCTTCTGG#.(((.((((((((((((((....#.)))))))))))))))	5374	5409	1	-23	0	0.0437957	0.7	0	0	-8	16	18	16	3UTR
mmu-miR-214-3p	XM_006497531	Plxdc2	ACAGCAGGCACAGACAGGCAGT#GCTCCCTGTCTGCTGCTA#..(((((...((((((((.(((#))).))))))))))))).	11835	####	1	-22	0	0.0074306	0.5	0	0	-10	16	18	13	3UTR

mmu-miR-214-3p	XM_006497952	Prrc2b	ACAGCAGGCACAGACAGGCAGT#GCTGTCCTCTCTCTGCTCTGCTGTC#((((((((((.(((.(((((((#)))).))).))).))).))))))).	5424	5449	0.92308	-26	1	0.0205359	0.5	0	0	-7	20	25	7	3UTR
mmu-miR-214-3p	XM_006497952	Prrc2b	ACAGCAGGCACAGACAGGCAGT#ACGGCCTGTGTCTGGAGCAAGATGTGACTGTGCCTGG#....((((((((((((((.((#)).))))))..................)))))))).	3126	3163	0.92308	-25	0	0.0001434	0.4	0	0	-10	17	37	9	CDS
mmu-miR-214-3p	XM_006497952	Prrc2b	ACAGCAGGCACAGACAGGCAGT#GCTGGCTGCCTGTGCTGCC#...(((.((((((.(((.((((#)))).))).)))))))))).	1915	1934	0.84615	-25	0	5.11E-08	0.4	0	0	-9	16	19	9	CDS
mmu-miR-214-3p	XM_006498541	Dgkz	ACAGCAGGCACAGACAGGCAGT#GCTCAAGGAAGCCTCTGTGCCTCTGG#.(((.((((((((((..(((...#.))))))))))))))).	2539	2565	1	-25	0	5.22E-07	0.4	0	0	-9	15	17	15	CDS
mmu-miR-214-3p	XM_006498543	Dgkz	ACAGCAGGCACAGACAGGCAGT#GCCTCTGTGCCTCTGG#.(((.((((((((((..(((...#))))))))))))))).	2037	2053	1	-25	0	5.22E-07	0.4	0	0	-9	15	16	15	CDS
mmu-miR-214-3p	XM_006498543	Dgkz	ACAGCAGGCACAGACAGGCAGT#GCTGCGATGTGGCTGCTGC#.((((((.((((.....(((((#)))))..)))).)))))).	52	71	0.84615	-25	1	0.00042	0.3	0	0	-9	15	19	6	CDS
mmu-miR-214-3p	XM_006498994	Ptprj	ACAGCAGGCACAGACAGGCAGT#CTGTGTGTCTGTGTGTGTCTCTGTG#((((((.(((((((((.((((.#)))).))))))))).))...)))).	4761	4786	0.92308	-28	0	0.1576382	0.6	0	0	-5	19	25	9	3UTR
mmu-miR-214-3p	XM_006498994	Ptprj	ACAGCAGGCACAGACAGGCAGT#ACCCCCGAAATGCTTGATTGTGCTTGTTGA#.((((((((((((.((((((..#.)))))).)))))))))))).	6843	6873	1	-26	1	0.0002306	0.5	0	0	-5	18	21	12	3UTR
mmu-miR-214-3p	XM_006498994	Ptprj	ACAGCAGGCACAGACAGGCAGT#ACTGCCTTCTGATGTTCTGCC#...(((((((((((.(((((((#))))))))))).))).)))).	5403	5424	0.81538	-24	0	0.1601578	0.4	0	0	-7	18	21	11	3UTR
mmu-miR-214-3p	XM_006499102	Tfap2c	ACAGCAGGCACAGACAGGCAGT#ACTGGACCTCTAAGCCTGTCCGTGCTCCTTAAATAATTGTTGTG#(((((((((((.(((((((...#.))))))).)))))...........)))))).	2816	2860	1	-26	1	0.2150844	0.7	0	0	-7	18	32	7	3UTR
mmu-miR-214-3p	XM_006499483	D430041D05Rik	ACAGCAGGCACAGACAGGCAGT#CTGCTTGCTGTGCTGGGCCTGG#.((((.(((((((.((((((((.#))))))))))))))..)).)).	554	576	0.92308	-26	0	4.24E-06	0.4	0	0	-7	18	22	14	5UTR
mmu-miR-214-3p	XM_006499483	D430041D05Rik	ACAGCAGGCACAGACAGGCAGT#TTGCCTGCTGTGGTGATGGACGTGCTGG#.(((((.((((((.((((((((.#))))))))))))........).))))).	5633	5661	0.92308	-26	0	7.59E-06	0.4	0	0	-6	18	28	12	CDS

mmu-miR-214-3p	XM_006499483	D430041D05Rik	ACAGCAGGCACAGACAGGCAGT#GCTGCTCTGTCTTCAGTGACCTCTGC#.(((.(((((((((((((((((#))))).))))))...))).)))))).	8868	8894	0.92308	-25	0	0.0044315	0.5	0	0	-8	20	26	6	3UTR
mmu-miR-214-3p	XM_006499487	D430041D05Rik	ACAGCAGGCACAGACAGGCAGT#CTGCTTGCTGTGCTGGGCCTGG#.((((.(((((((.(((((((.#))))))))))))))..)).)).	554	576	0.92308	-26	0	4.24E-06	0.4	0	0	-7	18	22	14	5UTR
mmu-miR-214-3p	XM_006499487	D430041D05Rik	ACAGCAGGCACAGACAGGCAGT#TTGCCTGCTGTGGTGATGGACGTGCTGG#.(((((.((((((.(((((((.#))))))))))))........).))))).	3122	3150	0.92308	-26	0	7.59E-06	0.4	0	0	-6	18	28	12	CDS
mmu-miR-214-3p	XM_006499487	D430041D05Rik	ACAGCAGGCACAGACAGGCAGT#GCTGCTCTGTCTTCAGTGACCTCTGC#.(((.(((((((((((((((((#))))).))))))...))).)))))).	6357	6383	0.92308	-25	0	0.0044315	0.5	0	0	-8	20	26	6	3UTR
mmu-miR-214-3p	XM_006500565	Kcnq2	ACAGCAGGCACAGACAGGCAGT#GCTATGTCTGTTGTCTGCTGTA#(((((((((((((((((((...#))).))))))).))))))))).	4603	4625	1	-30	1	0.009891	0.5	0	0	-4	19	22	9	3UTR
mmu-miR-214-3p	XM_006500565	Kcnq2	ACAGCAGGCACAGACAGGCAGT#ATTGCCTCCATTGCTGTGCTGGCTGCTGG#.((((((((((((..(((((((#)))))))......))))))...)))))).	1011	1040	0.92308	-26	1	0.0001418	0.4	0	0	-5	19	29	7	CDS
mmu-miR-214-3p	XM_006500567	Kcnq2	ACAGCAGGCACAGACAGGCAGT#GCTATGTCTGTTGTCTGCTGTA#(((((((((((((((((((...#))).))))))).))))))))).	4573	4595	1	-30	1	0.009891	0.5	0	0	-4	19	22	9	3UTR
mmu-miR-214-3p	XM_006500567	Kcnq2	ACAGCAGGCACAGACAGGCAGT#ATTGCCTCCATTGCTGTGCTGGCTGCTGG#.((((((((((((..(((((((#)))))))......))))))...)))))).	1011	1040	0.92308	-26	1	0.0001418	0.4	0	0	-5	19	29	7	CDS
mmu-miR-214-3p	XM_006500568	Kcnq2	ACAGCAGGCACAGACAGGCAGT#GCTATGTCTGTTGTCTGCTGTA#(((((((((((((((((((...#))).))))))).))))))))).	4564	4586	1	-30	1	0.009891	0.5	0	0	-4	19	22	9	3UTR
mmu-miR-214-3p	XM_006500568	Kcnq2	ACAGCAGGCACAGACAGGCAGT#ATTGCCTCCATTGCTGTGCTGGCTGCTGG#.((((((((((((..(((((((#)))))))......))))))...)))))).	1011	1040	0.92308	-26	1	0.0001418	0.4	0	0	-5	19	29	7	CDS
mmu-miR-214-3p	XM_006500848	Zzz3	ACAGCAGGCACAGACAGGCAGT#CTGCCTCTGTGTGGAGTTGTC#(((((..(((((((..(((((.#))))))))))))...))))).	3972	3993	0.94872	-22	0	3.68E-07	0.5	0	0	-8	17	21	12	3UTR
mmu-miR-214-3p	XM_006500848	Zzz3	ACAGCAGGCACAGACAGGCAGT#ACTGCCTGCCTACCTGTG#...(((((...((.((((((((#)))))))).)).))))).	1358	1376	0.92308	-21	0	0.0075791	0.5	0	0	-12	15	18	8	CDS

mmu-miR-214-3p	XM_006500848	Zzz3	ACAGCAGGCACAGACAGGCAGT#ACTGTCTGGTGGTCACTGATGGCCTGTA#...((((((.((..((((((((#))))))))...........)))))))).	3676	3704	0.84615	-21	0	0.0001948	0.5	0	0	-10	16	28	8	3UTR
mmu-miR-214-3p	XM_006500856	Zzz3	ACAGCAGGCACAGACAGGCAGT#GCTGCCTCGTCCAGAGCTGTGGGTGCAGAGCTGCCAGTGTGTGCTGTG#((((((.((((.((((((((((#))))))).))).......................)))).)))))	5428	5476	0.84615	-27	0	0.0001091	0.5	0	0	-9	20	48	7	3UTR
mmu-miR-214-3p	XM_006500856	Zzz3	ACAGCAGGCACAGACAGGCAGT#GCTGCCAGTGTGTGCTGTGTGTTGG#.(((((((((((.((.((((((#)))))).)).))))))...))))).	5457	5482	0.84615	-27	1	0.0003369	0.5	0	0	-5	19	25	6	3UTR
mmu-miR-214-3p	XM_006501049	Kcna2	ACAGCAGGCACAGACAGGCAGT#ACTGAAACCTCACATCTGAGTGACCTTATGCCAGCCTGTCTTGTTGAATTTGTGTCTGTCTGTC#((((((((((((((((((.((#.))..))))))))..........))))))).)))).	8865	8929	1	-26	1	0.0062272	0.5	0	0	-4	21	36	8	3UTR
mmu-miR-214-3p	XM_006501050	Kcna2	ACAGCAGGCACAGACAGGCAGT#GCTGCCTGGCGACGCCTGCTC#..(((((((...(.((((((((#)))))))).)...))))))).	143	164	0.84615	-28	1	2.71E-06	0.4	0	0	-13	16	21	8	5UTR
mmu-miR-214-3p	XM_006501050	Kcna2	ACAGCAGGCACAGACAGGCAGT#GCCAGCCTGTCTTGTTGAATTTGTGTCTGTCTGTC#((((((((((((((((((((.((#))..)))))))))..........))))))).)))).	8349	8384	1	-26	1	0.0062272	0.5	0	0	-4	21	35	8	3UTR
mmu-miR-214-3p	XM_006501120	Nras	ACAGCAGGCACAGACAGGCAGT#CTGCCTATTATTCCTGCTTACAGACTTTTGCCTGATGCCTGCTGTT#((((((((((((.(((((((.#)))))).......................).))).)))))))))))).	1917	1963	0.84615	-28	1	0.0023665	0.6	0	0	-5	20	46	10	3UTR
mmu-miR-214-3p	XM_006501120	Nras	ACAGCAGGCACAGACAGGCAGT#TGTGTGTGTGTGTGTGCA#...(((.(((((.(((.(((..#))).))).))))).))).	2943	2961	0.92308	-23	0	0.0034139	0.5	0	0	-9	14	18	5	3UTR
mmu-miR-214-3p	XM_006501122	Nras	ACAGCAGGCACAGACAGGCAGT#CTGCCTATTATTCCTGCTTACAGACTTTTGCCTGATGCCTGCTGTT#((((((((((((.(((((((.#)))))).......................).))).)))))))))))).	1938	1984	0.84615	-28	1	0.0023665	0.6	0	0	-5	20	46	10	3UTR
mmu-miR-214-3p	XM_006501122	Nras	ACAGCAGGCACAGACAGGCAGT#TGTGTGTGTGTGTGTGCA#...(((.(((((.(((.(((..#))).))).))))).))).	2964	2982	0.92308	-23	0	0.0034139	0.5	0	0	-9	14	18	5	3UTR

mmu-miR-214-3p	XM_006502342	Igsf3	ACAGCAGGCACAGACAGGCAGT#GCTGTCTGGCTGTGCTGGGTGTG#(((.(.(((((((.((((((((#)))))))).))))))).).))).	887	910	0.84615	-29	0	2.37E-06	0.4	0	0	-10	19	23	8	CDS
mmu-miR-214-3p	XM_006502342	Igsf3	ACAGCAGGCACAGACAGGCAGT#TGTGTGTGTGTGCGTGCG#...(((.(((((.(((.(((..#))).))).))))).))).	5916	5934	0.92308	-25	0	0.0001348	0.5	0	0	-9	14	18	5	3UTR
mmu-miR-214-3p	XM_006502342	Igsf3	ACAGCAGGCACAGACAGGCAGT#GCCTGTCTGTTCTGGAGA#.(..((((.((((((((((...#))))))))))))))..).	3395	3413	1	-24	0	0.0001769	0.4	0	0	-9	15	18	14	CDS
mmu-miR-214-3p	XM_006502983	Tut4	ACAGCAGGCACAGACAGGCAGT#TGCTTGTTTCTAAGCCTGA#....(((((..(((((((((..#)))))))))....))))).	3507	3526	1	-19	0	0.0019176	0.6	0	0	-14	14	19	9	3UTR
mmu-miR-214-3p	XM_006503658	Nsd2	ACAGCAGGCACAGACAGGCAGT#ACTGTCTGTCCTTTGGCTTGCTT#..(((((((.((((((((((((#))))))))))))...)))))))).	9158	9181	0.92308	-26	1	0.0001266	0.4	0	0	-6	19	23	10	3UTR
mmu-miR-214-3p	XM_006503658	Nsd2	ACAGCAGGCACAGACAGGCAGT#ACTGAAAATCTTGACTTGGATGGTCTGGGAGCCTGCTGTG#(((((((((.(((((...(((.#.)))....)))))...))))))))).	1865	1905	1	-25	1	0.0001692	0.6	0	0	-5	17	26	9	5UTR
mmu-miR-214-3p	XM_006503658	Nsd2	ACAGCAGGCACAGACAGGCAGT#GCTGCCTTTGTTTTGCCTGTTT#..((((((((.((((((((((#))))))))..)))))))))))).	8775	8797	1	-25	1	0.0579734	0.5	0	0	-8	19	22	12	3UTR
mmu-miR-214-3p	XM_006503662	Nsd2	ACAGCAGGCACAGACAGGCAGT#GTCTGGGAGCCTGCTGTG#(((((((((.(((((.......#)))))...))))))))).	1887	1905	1	-25	1	0.0001692	0.6	0	0	-5	14	18	9	5UTR
mmu-miR-214-3p	XM_006503876	Agbl5	ACAGCAGGCACAGACAGGCAGT#GCTTGCCTGTTTCCTGCTC#..((((((...((((((((((((#))).)))))))))))))).	2058	2077	0.92308	-23	1	0.0002095	0.5	0	0	-10	17	19	14	CDS
mmu-miR-214-3p	XM_006503876	Agbl5	ACAGCAGGCACAGACAGGCAGT#GCCTGACTGTGCTG#......(((((((.(((((...#))))).))))))).	358	372	1	-22	0	0.0003434	0.5	0	0	-16	12	14	7	CDS
mmu-miR-214-3p	XM_006504832	Ubl3	ACAGCAGGCACAGACAGGCAGT#CTGCACGTGTCTGTGGCGCCCAGTTCTCCTGTGTGTA#((((((((((((((((((((.#)))).).)))))))))............))))).))).	1512	1549	0.84615	-23	1	6.25E-05	0.4	0	0	-4	21	37	8	3UTR
mmu-miR-214-3p	XM_006505427	Cacna1c	ACAGCAGGCACAGACAGGCAGT#CCTGTCATGTGCCATGCCTGTT#(((((((((((((((((....#)))))).)))))).))).))).	14014	####	1	-26	1	0.0031596	0.5	0	0	-4	18	22	6	3UTR

mmu-miR-214-3p	XM_006505427	Cacna1c	ACAGCAGGCACAGACAGGCAGT#CCTGCTCTGTGTCTGCC#...((((((((((((((....#)))).))))))))))).	8561	8578	1	-26	0	0.0055044	0.6	0	0	-7	15	17	11	3UTR
mmu-miR-214-3p	XM_006505427	Cacna1c	ACAGCAGGCACAGACAGGCAGT#CTGCCTGCTGCGCCTTCTC#..((.((((.(((.(((((((.#)))))))))).)))).)).	14188	####	0.92308	-26	0	1.86E-05	0.4	0	0	-11	16	19	10	3UTR
mmu-miR-214-3p	XM_006505536	Slc6a12	ACAGCAGGCACAGACAGGCAGT#GCTAACAAGCCTGCATGGATCACCTGCTGTG#((((((((..((..(((((...#.)))))..)).....)))))))).	2253	2284	0.92308	-24	1	0.0004582	0.5	0	0	-8	15	24	8	3UTR
mmu-miR-214-3p	XM_006505536	Slc6a12	ACAGCAGGCACAGACAGGCAGT#GCTGGCCTTGTGCCTCCTGC#.(((.(((((((...(((((((#)))).)))))))))).))).	674	694	1	-22	0	0.0014479	0.4	0	0	-11	17	20	10	CDS
mmu-miR-214-3p	XM_006506063	Tpra1	ACAGCAGGCACAGACAGGCAGT#GCCCCACCAGTGCCTGCTGC#.((((((((((.....(((...#)))......)))))))))).	664	684	0.84615	-25	1	0.0002779	0.5	0	0	-10	13	20	10	CDS
mmu-miR-214-3p	XM_006506918	Kras	ACAGCAGGCACAGACAGGCAGT#GCCTGTCTCTCACCTGCC#...(((((...((((((((...#))))))))....))))).	2740	2758	0.84615	-24	0	0.0546249	0.6	0	0	-12	13	18	8	3UTR
mmu-miR-214-3p	XM_006506918	Kras	ACAGCAGGCACAGACAGGCAGT#ACTGGATGTCTGTCTGATTTTAAGCAAATCACTGTCTGCTT#..((((((((((((((((((..#.))))))))))................)))))))).	2482	2523	0.92308	-25	1	0.0039511	0.6	0	0	-5	18	36	10	3UTR
mmu-miR-214-3p	XM_006507015	Bicd1	ACAGCAGGCACAGACAGGCAGT#GCTGCTCCTGTGTGTGTGTGTGTGTG#((((((.(((((.(((((((((#)))))..)))).))))).))).))).	4336	4362	0.92308	-25	0	0.1463821	0.5	0	0	-7	20	26	5	3UTR
mmu-miR-214-3p	XM_006507024	Bicd1	ACAGCAGGCACAGACAGGCAGT#GCAGTCGGCTGTGCTGCGCTGC#.((((.(((((((.(.(((.((#)).))).))))))))..)))).	60	82	1	-21	0	1.31E-06	0.4	0	0	-7	17	22	8	5UTR
mmu-miR-214-3p	XM_006508621	Tspan32	ACAGCAGGCACAGACAGGCAGT#GCTGACAGATAGGAGGACACAATGATACCTACTGTGTCTGCTGTG#(((((((((((((..(((....#.))).))))))))))))).	15	60	1	-26	1	0.0015236	0.5	0	0	-6	16	19	13	5UTR
mmu-miR-214-3p	XM_006508621	Tspan32	ACAGCAGGCACAGACAGGCAGT#TGCTATGATGCTCTGTGCCTTCCTGTG#((((.((((((((((((((((..#))))....)).))))))))))..)))).	747	774	0.84615	-23	0	0.0034939	0.5	0	0	-8	19	27	9	CDS
mmu-miR-214-3p	XM_006508851	Grtp1	ACAGCAGGCACAGACAGGCAGT#CTGCCTGTTTGTGGACATCCTACCTGTG#...((((((((((((((((((.#))))))))))))).........))))).	890	918	1	-27	0	0.0014729	0.4	0	0	-7	18	28	13	CDS

mmu-miR-214-3p	XM_006508851	Grtp1	ACAGCAGGCACAGACAGGCAGT#GCTGCTTCCGCCTGCTGA#.((((((((...((..((((((#)))))))).)))))))).	1397	1415	0.92308	-24	1	4.13E-05	0.5	0	0	-12	16	18	8	3UTR
mmu-miR-214-3p	XM_006509023	Ikbkb	ACAGCAGGCACAGACAGGCAGT#GCTGCTCCCTGACAAGCCTGCTA#..(((((((.(((...((((((#))))))..)))....))))))).	1217	1240	0.92308	-21	1	0.0003155	0.4	0	0	-8	16	23	7	CDS
mmu-miR-214-3p	XM_006509251	Slc7a2	ACAGCAGGCACAGACAGGCAGT#GCTGCACTGTGTGGGTGTCTGCTGTT#(((((((((((..(((((((((#))))).))))....))))))))))).	7026	7052	0.84615	-31	1	0.0001555	0.4	0	0	-9	20	26	11	3UTR
mmu-miR-214-3p	XM_006509251	Slc7a2	ACAGCAGGCACAGACAGGCAGT#GCTGGGCTGTCGTGGGTCTGCTGTG#(((((((((.(((((((.((((#))))..))))).)).))))))))).	2225	2250	1	-28	1	1.72E-05	0.4	0	0	-5	20	25	9	3UTR
mmu-miR-214-3p	XM_006509963	Cbl	ACAGCAGGCACAGACAGGCAGT#CTGCACATTGTTTTGTGCCTGCTGA#.(((((((((((((((((((.#))))...)))))).))))))))).	9459	9484	1	-29	1	0.0004257	0.4	0	0	-4	20	25	10	3UTR
mmu-miR-214-3p	XM_006509963	Cbl	ACAGCAGGCACAGACAGGCAGT#ACTGGCCACACTGTGCCTGCA#...((((((((((...((((((#)))).))...)))))))))).	8341	8362	1	-27	0	4.69E-06	0.5	0	0	-8	16	21	10	3UTR
mmu-miR-214-3p	XM_006509963	Cbl	ACAGCAGGCACAGACAGGCAGT#CTGTTGTCTGTGTGTCCTGTCATTATAAGATTATCCTGCTGTG#((((((((((((((((.((((.#))))))))))))......................)))))))).	3335	3378	0.92308	-27	1	0.002629	0.5	0	0	-4	20	43	12	3UTR
mmu-miR-214-3p	XM_006510002	Ube4a	ACAGCAGGCACAGACAGGCAGT#GCTGCAAAACTGTCTGAATTTGCAGGTGTCCATGGCTGTTCTGCTGG#.(((((((((((((((((((((#)))))....)))))))...................))).))))))).	1967	2014	0.84615	-24	1	0.0001573	0.5	0	0	-4	21	47	7	CDS
mmu-miR-214-3p	XM_006510002	Ube4a	ACAGCAGGCACAGACAGGCAGT#CCTGTCTGATCCCAGCTGTG#(((((.((..((((((((....#))))))))...)).))))).	1693	1713	0.88462	-22	0	9.99E-05	0.4	0	0	-8	15	20	8	CDS
mmu-miR-214-3p	XM_006510590	Sik3	ACAGCAGGCACAGACAGGCAGT#ACAGCCTGCTGTGCGATCGACACAAGAAACATAAAACTCTGCG#...((((((((((.(((((.((#)).))))))))))))).......................)))).	1192	1235	0.81538	-22	0	0.0624992	0.4	0	0	-7	17	43	11	CDS
mmu-miR-214-3p	XM_006510590	Sik3	ACAGCAGGCACAGACAGGCAGT#ACTGTGCTGTTTGTGTGATCTCCATTCTTATTCTCTACTGCG#...(((((((((((((((((((#))))).))))))))))).....................)))).	4836	4878	0.84615	-22	0	0.0636651	0.6	0	0	-7	19	42	10	3UTR

mmu-miR-214-3p	XM_006510641	Ppp2r1b	ACAGCAGGCACAGACAGGCAGT#TGCACTGTCTGAAGCCTGTGGTA#((.((((((.((((((((((..#))).)))))))..)))))).)).	1885	1908	1	-26	0	4.26E-05	0.5	0	0	-7	18	23	7	CDS
mmu-miR-214-3p	XM_006510641	Ppp2r1b	ACAGCAGGCACAGACAGGCAGT#GCTGCTGGCTGGCCAGCTGG#.((((.(((.(((.(.((((((#)))))).))))))).)))).	1651	1671	0.84615	-25	0	8.14E-07	0.5	0	0	-8	17	20	7	CDS
mmu-miR-214-3p	XM_006510641	Ppp2r1b	ACAGCAGGCACAGACAGGCAGT#GCCCCGCCTGTTTGTGTGTGG#....((.(((((((((((((.((#))...)))))))))))).)).	222	243	1	-24	0	1.72E-05	0.3	0	0	-11	16	21	12	CDS
mmu-miR-214-3p	XM_006510907	Thsd4	ACAGCAGGCACAGACAGGCAGT#CTGCCTGTCCCTCTCACTGTGCCTCTGC#.(((.(((((((((((((((((.#))))))))))........)))))))))).	4969	4997	0.84615	-30	0	0.0770473	0.4	0	0	-8	19	28	10	3UTR
mmu-miR-214-3p	XM_006511027	Tle3	ACAGCAGGCACAGACAGGCAGT#GCTCGGCCCCCTTCGCCTGTCTGTGTGTGGTA#..(.(((.((((((((((((...#.)))))))))))).))).).	82	114	1	-28	0	1.17E-05	0.4	0	0	-10	15	19	12	5UTR
mmu-miR-214-3p	XM_006511027	Tle3	ACAGCAGGCACAGACAGGCAGT#GCTCGCCGTGGATTTTGCCTGCTGTA#((((((((((.(..((((((((#))).)))).))..)..))))))))).	3909	3935	0.84615	-26	1	0.0093044	0.5	0	0	-9	19	26	10	3UTR
mmu-miR-214-3p	XM_006511027	Tle3	ACAGCAGGCACAGACAGGCAGT#TGCCAAAGTCTGTTTTTCCTGCTGC#.(((((((.((((((.((((..#))))...))))))....))))))).	2878	2903	0.84615	-25	1	0.0167808	0.5	0	0	-5	17	25	7	CDS
mmu-miR-214-3p	XM_006511028	Tle3	ACAGCAGGCACAGACAGGCAGT#GCTCGGCCCCCTTCGCCTGTCTGTGTGTGGTA#..(.(((.((((((((((((...#.)))))))))))).))).).	82	114	1	-28	0	1.17E-05	0.4	0	0	-10	15	19	12	5UTR
mmu-miR-214-3p	XM_006511028	Tle3	ACAGCAGGCACAGACAGGCAGT#GCTCGCCGTGGATTTTGCCTGCTGTA#((((((((((.(..((((((((#))).)))).))..)..))))))))).	3894	3920	0.84615	-26	1	0.0093044	0.5	0	0	-9	19	26	10	3UTR
mmu-miR-214-3p	XM_006511028	Tle3	ACAGCAGGCACAGACAGGCAGT#TGCCAAAGTCTGTTTTTCCTGCTGC#.(((((((.((((((.((((..#))))...))))))....))))))).	2863	2888	0.84615	-25	1	0.0167808	0.5	0	0	-5	17	25	7	CDS
mmu-miR-214-3p	XM_006511029	Tle3	ACAGCAGGCACAGACAGGCAGT#GCTCGGCCCCCTTCGCCTGTCTGTGTGTGGTA#..(.(((.((((((((((((...#.)))))))))))).))).).	82	114	1	-28	0	1.17E-05	0.4	0	0	-10	15	19	12	5UTR
mmu-miR-214-3p	XM_006511029	Tle3	ACAGCAGGCACAGACAGGCAGT#GCTCGCCGTGGATTTTGCCTGCTGTA#((((((((((.(..((((((((#))).)))).))..)..))))))))).	3888	3914	0.84615	-26	1	0.0093044	0.5	0	0	-9	19	26	10	3UTR

mmu-miR-214-3p	XM_006511029	Tle3	ACAGCAGGCACAGACAGGCAGT#TGCCAAAGTCTGTTTTTCCTGCTGC#.(((((((.((((((.((((..#))))...))))))....))))))).	2857	2882	0.84615	-25	1	0.0167808	0.5	0	0	-5	17	25	7	CDS
mmu-miR-214-3p	XM_006511085	Dmxl2	ACAGCAGGCACAGACAGGCAGT#ACTGCAATTCTGCAAGTGCTTGCTT#..(((((((((...((((((((#)))))....)))...))))))))).	2746	2771	0.84615	-22	1	0.0001371	0.6	0	0	-10	17	25	9	CDS
mmu-miR-214-3p	XM_006511398	Aph1c	ACAGCAGGCACAGACAGGCAGT#GCTGCTGTGTGTGCTGTCTGA#.(((..((((((.(((.(((((#)))))))).))))))..))).	2163	2184	1	-25	0	0.0015389	0.5	0	0	-11	17	21	8	3UTR
mmu-miR-214-3p	XM_006511398	Aph1c	ACAGCAGGCACAGACAGGCAGT#GTCATGTCAGTGCCTCTGCTGG#.((((((((((.(((((((...#))).)))).))))))..))))).	2966	2988	0.92308	-25	1	9.93E-05	0.6	0	0	-7	17	22	6	3UTR
mmu-miR-214-3p	XM_006511398	Aph1c	ACAGCAGGCACAGACAGGCAGT#GCTGCCGAAGCCTGAAATTCTGCCTGCTC#..(((((((((((...((((((#))))))).....)))......)))))))).	1322	1351	0.84615	-24	1	0.0015989	0.5	0	0	-7	17	29	8	CDS
mmu-miR-214-3p	XM_006511591	Hmgn3	ACAGCAGGCACAGACAGGCAGT#TGTCTCAGCCTGCA#...((((((..(((((......#)))))).))))))).	1200	1214	0.86538	-19	0	0.0822448	0.5	0	0	-11	11	14	6	CDS
mmu-miR-214-3p	XM_006511997	Scn5a	ACAGCAGGCACAGACAGGCAGT#GCTGGGCCTGTCTCGCATGGGCAACCTGTCTGTGCTGCGTTCCTTCCGTCTGCTGC#.((((((((((((((((..((#.))..))))))))))))...........	2620	2676	0.92308	-29	1	0.0048814	0.4	0	0	-4	19	37	11	CDS
mmu-miR-214-3p	XM_006511997	Scn5a	ACAGCAGGCACAGACAGGCAGT#GGTGTCTGGGCAGTCGCTGTGCTTGCTGG#.((((((((((((.((((((.(#).))))))........)))))))))))	2965	2994	0.92308	-27	1	6.20E-05	0.4	0	0	-5	19	29	12	CDS
mmu-miR-214-3p	XM_006513133	Ank3	ACAGCAGGCACAGACAGGCAGT#CTGCCTGCCTCTGCCTCCCAAGTGCTGG#.((((((((.((.(((((((.#))))))).)).)))))).......)))).	17133	####	1	-26	1	2.43E-05	0.4	0	0	-7	18	28	7	5UTR
mmu-miR-214-3p	XM_006513133	Ank3	ACAGCAGGCACAGACAGGCAGT#GCTGTCTAATCCCCAGTCACAGGGGCCCTGTCTGTGCCTCCA#...(.((((((((((((....#.))))))))))))).).	45962	####	0.84615	-26	0	0.0015804	0.5	0	0	-11	14	17	13	3UTR
mmu-miR-214-3p	XM_006513139	Ank3	ACAGCAGGCACAGACAGGCAGT#GCTGTCTAATCCCCAGTCACAGGGGCCCTGTCTGTGCCTCCA#...(.((((((((((((....#.))))))))))))).).	9183	9225	0.84615	-26	0	0.0015804	0.5	0	0	-11	14	17	13	3UTR

mmu-miR-214-3p	XM_006513335	Nfic	ACAGCAGGCACAGACAGGCAGT#GCCTGAGCACCATTGACTGCCTGCTGTT#(((((((((((((.(((((...#)))))…….)))..)))))))))).	7175	7203	1	-24	1	0.0361053	0.5	0	0	-5	18	28	10	3UTR
mmu-miR-214-3p	XM_006514060	1500009L16Rik	ACAGCAGGCACAGACAGGCAGT#ATTCCTGCTGTGCAACCTCGCCCGAATCCTAGCTGTG#(((((((((((((.((((.(((#)))))))))))))……………)).))))).	451	488	0.92308	-20	1	0.0136856	0.5	0	0	-5	20	37	13	3UTR
mmu-miR-214-3p	XM_006514230	Btbd11	ACAGCAGGCACAGACAGGCAGT#CTGTCTGTCTGTACTGGGCCAAGGAGCTGC#.((((.((((((((((((((.#))))))))))))).....))).....))	5421	5451	0.84615	-26	0	3.40E-05	0.4	0	0	-6	19	30	12	3UTR
mmu-miR-214-3p	XM_006514610	Tcn2	ACAGCAGGCACAGACAGGCAGT#ACTGCCAGGCATCCAGGGTCATGTTGGTGCCAGCTGTT#(((((.(((((.((((((((((#))))))………….)))).))))).))))).	1148	1186	0.84615	-27	0	0.0006268	0.4	0	0	-9	20	38	6	CDS
mmu-miR-214-3p	XM_006514610	Tcn2	ACAGCAGGCACAGACAGGCAGT#ATGGCCTGCTGTGGCTCTGA#.(((.((.((((((.(((((.((#)).)))))))))).))))).	1832	1852	0.84615	-23	0	1.90E-05	0.4	0	0	-11	17	20	10	3UTR
mmu-miR-214-3p	XM_006514611	Tcn2	ACAGCAGGCACAGACAGGCAGT#ACTGCCAGGCATCCAGGGTCATGTTGGTGCCAGCTGTT#(((((.(((((.((((((((((#))))))………….)))).))))).))))).	1120	1158	0.84615	-27	0	0.0006268	0.4	0	0	-9	20	38	6	CDS
mmu-miR-214-3p	XM_006514611	Tcn2	ACAGCAGGCACAGACAGGCAGT#ATGGCCTGCTGTGGCTCTGA#.(((.((.((((((.(((((.((#)).)))))))))).))))).	1804	1824	0.84615	-23	0	1.90E-05	0.4	0	0	-11	17	20	10	3UTR
mmu-miR-214-3p	XM_006514612	Tcn2	ACAGCAGGCACAGACAGGCAGT#ACTGCCAGGCATCCAGGGTCATGTTGGTGCCAGCTGTT#(((((.(((((.((((((((((#))))))………….)))).))))).))))).	1189	1227	0.84615	-27	0	0.0006268	0.4	0	0	-9	20	38	6	CDS
mmu-miR-214-3p	XM_006514612	Tcn2	ACAGCAGGCACAGACAGGCAGT#ATGGCCTGCTGTGGCTCTGA#.(((.((.((((((.(((((.((#)).)))))))))).))))).	1873	1893	0.84615	-23	0	1.90E-05	0.4	0	0	-11	17	20	10	3UTR
mmu-miR-214-3p	XM_006514613	Tcn2	ACAGCAGGCACAGACAGGCAGT#ACTGCCAGGCATCCAGGGTCATGTTGGTGCCAGCTGTT#(((((.(((((.((((((((((#))))))………….)))).))))).))))).	1161	1199	0.84615	-27	0	0.0006268	0.4	0	0	-9	20	38	6	CDS
mmu-miR-214-3p	XM_006514613	Tcn2	ACAGCAGGCACAGACAGGCAGT#ATGGCCTGCTGTGGCTCTGA#.(((.((.((((((.(((((.((#)).)))))))))).))))).	1845	1865	0.84615	-23	0	1.90E-05	0.4	0	0	-11	17	20	10	3UTR

mmu-miR-214-3p	XM_006514793	Cpeb4	ACAGCAGGCACAGACAGGCAGT#ACCGCCTGGCTGGTGACCCTTCTGCTGTC#(((((((((((((.(((((.((#)).))))).))).)).....)))))))).	5086	5115	0.84615	-23	1	6.99E-06	0.5	0	0	-5	20	29	8	3UTR
mmu-miR-214-3p	XM_006514793	Cpeb4	ACAGCAGGCACAGACAGGCAGT#GCTTGGGGTGTGCTATTGTGCTGG#.((((((((((((..(((((...#)))))...))))))....))))).	5403	5427	1	-23	1	0.000947	0.5	0	0	-6	16	24	6	3UTR
mmu-miR-214-3p	XM_006514848	Mtmr3	ACAGCAGGCACAGACAGGCAGT#GCTGCCTGAGTGGGCACATGTCTGACTCCTGTT#((((((((((((..((((((((#))))))))..))......))))))....)))).	4821	4854	0.92308	-23	1	0.0103716	0.5	0	0	-6	20	33	8	3UTR
mmu-miR-214-3p	XM_006514848	Mtmr3	ACAGCAGGCACAGACAGGCAGT#GCGCGCTCTGTGTGGCCGCTGC#.((((.((((.((.(((((.((#))..)).)))).))))))))).	62	84	0.92308	-22	0	0.0014596	0.4	0	0	-8	17	22	9	5UTR
mmu-miR-214-3p	XM_006515635	Sos2	ACAGCAGGCACAGACAGGCAGT#CTGCACTATCGCAGCACTCTAGACAGAATGCTGGACTCTGTGCTGCTGA#.(((((.(((((((....((((#.))))...)))))))))))).	1646	1695	0.84615	-23	0	1.97E-06	0.5	0	0	-6	16	21	12	CDS
mmu-miR-214-3p	XM_006516006	Ttll5	ACAGCAGGCACAGACAGGCAGT#CCTGCTGTGCGCTCCTGG#.(((.((((((((.((((....#)))))))))).)).))).	2406	2424	1	-22	0	0.0001139	0.4	0	0	-9	15	18	10	3UTR
mmu-miR-214-3p	XM_006516006	Ttll5	ACAGCAGGCACAGACAGGCAGT#TCTGTCTCTCCTCCTGCTGC#.(((((((...(((((((....#))))))).....))))))).	2687	2707	1	-20	1	0.0260191	0.4	0	0	-9	14	20	7	3UTR
mmu-miR-214-3p	XM_006516872	Mak	ACAGCAGGCACAGACAGGCAGT#CTGCCCGTCTCGTTTTAGTCCTGTTGTT#(((((((((((((((.(((((.#))))).)))).))....).)))))))).	2794	2822	1	-24	1	0.0220696	0.5	0	0	-4	20	28	8	3UTR
mmu-miR-214-3p	XM_006516872	Mak	ACAGCAGGCACAGACAGGCAGT#GCCTGTCTCCAGTGTAGCA#...((..((((((((((((...#))))))))...)))).)).	208	227	0.84615	-21	0	0.0001661	0.6	0	0	-10	14	19	8	5UTR
mmu-miR-214-3p	XM_006516931	Fgd3	ACAGCAGGCACAGACAGGCAGT#GCTGTCACTCTGGGAAAGGCAGAGCTGTCTGTGGCCTGA#....((((((((((((((((((#)))))..................))))))))).)))).	3836	3875	0.96154	-24	0	7.32E-05	0.4	0	0	-10	18	39	9	3UTR
mmu-miR-214-3p	XM_006516931	Fgd3	ACAGCAGGCACAGACAGGCAGT#ACTGCTCTCTCCTGAAGCATTCCTTGTCTGCTATTCCTGCTGA#.(((((((..((((((((((((#)))))).................)))))).....))))))).	3943	3986	0.84615	-24	1	0.0062105	0.4	0	0	-6	19	43	7	3UTR

mmu-miR-214-3p	XM_006517420	Rmi1	ACAGCAGGCACAGACAGGCAGT#CTGGCTGTCGTGGGATCCTGTAGATCTCCTGCCGCTGC#.((((.(((((((((((.(((.#))).))))).))................)))))))).	568	606	0.84615	-22	0	0.0013053	0.4	0	0	-6	18	38	8	5UTR
mmu-miR-214-3p	XM_006517732	BC147527	ACAGCAGGCACAGACAGGCAGT#ACTGTTTGGAGCCCAGATGCCTGCTA#..(((((((((...((((((((#))))))))).......).)))))))).	1428	1454	0.84615	-21	1	0.0091835	0.5	0	0	-10	17	26	8	3UTR
mmu-miR-214-3p	XM_006517732	BC147527	ACAGCAGGCACAGACAGGCAGT#ATTTTCTGTCAGCTCCTGCTGTT#((((((((..(.((((((.(((#))).)))))).)..)))))))).	1471	1494	0.92308	-21	1	1.53E-05	0.6	0	0	-10	18	23	8	3UTR
mmu-miR-214-3p	XM_006517734	Gm20767	ACAGCAGGCACAGACAGGCAGT#TTGCAATTAGGGAATCTTCTGTCAGCTCCTGCTGTT#((((((((..(.((((((....#.)))))).)..)))))	762	798	1	-21	1	5.18E-05	0.5	0	0	-10	15	20	8	3UTR
mmu-miR-214-3p	XM_006517966	Thrb	ACAGCAGGCACAGACAGGCAGT#GCTGCCTGAGTTCTACTTTGTACTCTGCAGCCTGG#....(((((.((((((((((((#)))))))))..............)))))..))))).	3547	3582	0.84615	-24	0	4.30E-05	0.6	0	0	-11	17	35	8	3UTR
mmu-miR-214-3p	XM_006517966	Thrb	ACAGCAGGCACAGACAGGCAGT#GCCTGTACCTGGCCTCCTGC#.(((.((((.(((((((((...#))))))..))))))).))).	3982	4002	0.92308	-24	0	4.63E-05	0.5	0	0	-9	16	20	7	3UTR
mmu-miR-214-3p	XM_006518668	Il17rd	ACAGCAGGCACAGACAGGCAGT#GCCATCACTGTGCCTCTGG#.(((.((((((((...(((...#)))....)))))))))))).	782	801	0.92308	-23	0	0.0040904	0.4	0	0	-10	14	19	11	CDS
mmu-miR-214-3p	XM_006519193	Colq	ACAGCAGGCACAGACAGGCAGT#GCTGCCTCTGGGCTGTTGG#.((((((.(.((((..((((((#)))))))))))).)))))).	3155	3174	0.98077	-25	1	0.0002815	0.5	0	0	-8	17	19	11	3UTR
mmu-miR-214-3p	XM_006519193	Colq	ACAGCAGGCACAGACAGGCAGT#GCCTGTCTCTAGCTTGTG#...((((((..((((((((...#))))))))...)))))).	653	671	0.84615	-23	0	0.004571	0.4	0	0	-11	14	18	8	5UTR
mmu-miR-214-3p	XM_006519542	Naa30	ACAGCAGGCACAGACAGGCAGT#GCGTCTGCCAGTGCCTGTGTA#(((.((((((((.(.((((((.((#))))))).).)))))))))).	1820	1841	0.84615	-21	0	0.0004244	0.6	0	0	-11	18	21	10	3UTR
mmu-miR-214-3p	XM_006519674	Glt8d1	ACAGCAGGCACAGACAGGCAGT#GCCCTACGTGGCTGCTGC#.((((((.(((.....(((...#)))....))).)))))).	17	35	0.84615	-21	1	6.24E-05	0.3	0	0	-13	12	18	6	5UTR
mmu-miR-214-3p	XM_006519674	Glt8d1	ACAGCAGGCACAGACAGGCAGT#GCTGCTGTAGGTAGGAACTGCTGTT#(((((((..((..(((.(((((#))))))))..)).....))))))).	1564	1589	0.84615	-20	1	8.46E-05	0.6	0	0	-12	17	25	8	3UTR

mmu-miR-214-3p	XM_006520531	Oxr1	ACAGCAGGCACAGACAGGCAGT#GGCTGTGCCGTGCCTGTTC#..(((((((((.(((((.(...#).)))).).))))))))).	3867	3886	0.92308	-23	1	0.0011623	0.6	0	0	-8	15	19	9	5UTR
mmu-miR-214-3p	XM_006520531	Oxr1	ACAGCAGGCACAGACAGGCAGT#TTGTGTGTGTGTGTGTGTGTGTG#((((((.(((((.(((.((((.#)))).))).))))).))).))).	4173	4196	0.92308	-22	0	0.5467099	0.6	0	0	-7	18	23	5	5UTR
mmu-miR-214-3p	XM_006522111	Mrtfb	ACAGCAGGCACAGACAGGCAGT#ACTCCTCAGCCTTCTGTGCCTCCTGG#.((((.(((((((((.((((...#.))))))))))))).))).	5789	5815	1	-28	0	0.0003147	0.5	0	0	-9	16	19	13	3UTR
mmu-miR-214-3p	XM_006522111	Mrtfb	ACAGCAGGCACAGACAGGCAGT#CTGTGTGTGTGTGTGTGTGTGTG#((((((.(((((.(((.((((.#)))).))).))))).))).))).	4158	4181	0.84615	-24	0	0.018177	0.4	0	0	-7	18	23	5	3UTR
mmu-miR-214-3p	XM_006522111	Mrtfb	ACAGCAGGCACAGACAGGCAGT#GCTGCTTGCTGACTGTGCAGCTT#..(((..((((((.((((((((#))))))))....)))))).))).	760	783	0.84615	-23	0	3.11E-06	0.5	0	0	-9	17	23	8	3UTR
mmu-miR-214-3p	XM_006523564	Col11a2	ACAGCAGGCACAGACAGGCAGT#GCCTCCTGGCCTGCCTGG#.((((((((.(((..((((...#)))).)))))))))).)).	5543	5561	0.92308	-23	1	6.20E-06	0.3	0	0	-7	15	18	9	CDS
mmu-miR-214-3p	XM_006523567	Col11a2	ACAGCAGGCACAGACAGGCAGT#GCCTCCTGGCCTGCCTGG#.((((((((.(((..((((...#)))).)))))))))).)).	5402	5420	0.92308	-23	1	6.20E-06	0.3	0	0	-7	15	18	9	CDS
mmu-miR-214-3p	XM_006523569	Col11a2	ACAGCAGGCACAGACAGGCAGT#GCCTCCTGGCCTGCCTGG#.((((((((.(((..((((...#)))).)))))))))).)).	5300	5318	0.92308	-23	1	6.20E-06	0.3	0	0	-7	15	18	9	CDS
mmu-miR-214-3p	XM_006523570	Col11a2	ACAGCAGGCACAGACAGGCAGT#GCCTCCTGGCCTGCCTGG#.((((((((.(((..((((...#)))).)))))))))).)).	5222	5240	0.92308	-23	1	6.20E-06	0.3	0	0	-7	15	18	9	CDS
mmu-miR-214-3p	XM_006523765	Afdn	ACAGCAGGCACAGACAGGCAGT#ACTGTGTTACTGTGCCTCCTCTGA#.(((.((((((((..(.(((((#))))).)..))))))))...))).	6571	6595	1	-23	0	0.0328463	0.6	0	0	-10	17	24	8	3UTR
mmu-miR-214-3p	XM_006523766	Afdn	ACAGCAGGCACAGACAGGCAGT#ACTGTGTTACTGTGCCTCCTCTGA#.(((.((((((((..(.(((((#))))).)..))))))))...))).	6589	6613	1	-23	0	0.0328463	0.6	0	0	-10	17	24	8	3UTR
mmu-miR-214-3p	XM_006523937	Slc8a1	ACAGCAGGCACAGACAGGCAGT#TGTCTGTCTGTCCTCTGG#.(((.(((.((((((((((((..#))))))))))))))))))).	15820	####	1	-26	0	0.0009438	0.6	0	0	-9	17	18	17	3UTR

mmu-miR-214-3p	XM_006523937	Slc8a1	ACAGCAGGCACAGACAGGCAGT#ACTGCCGTTGTGTTTGTTGC#.(((((((((((.(((.((((((#)))))))))))))))))))).	3289	3309	1	-25	1	0.0018778	0.5	0	0	-5	19	20	19	CDS
mmu-miR-214-3p	XM_006523937	Slc8a1	ACAGCAGGCACAGACAGGCAGT#GCTGCTTGTCCATTTGCTCCGGCTGG#.((((.((((..((((((((((#))))))))))....))))...)))).	15956	####	0.92308	-25	0	0.0001054	0.4	0	0	-12	18	26	10	3UTR
mmu-miR-214-3p	XM_006524091	Anks1	ACAGCAGGCACAGACAGGCAGT#CTGCGGGCTGTCTGCCTGCTGC#.((((((((..((((((((((.#))))...))))))))))))))).	3482	3504	1	-28	1	8.56E-09	0.4	0	0	-8	18	22	14	3UTR
mmu-miR-214-3p	XM_006524091	Anks1	ACAGCAGGCACAGACAGGCAGT#CTGCCTCTCTCTGATGCTTGTTGA#.(((((((((((((.((((((.#))))))...)))).))))))))).	3011	3035	1	-27	1	0.0053076	0.4	0	0	-5	19	24	9	3UTR
mmu-miR-214-3p	XM_006524091	Anks1	ACAGCAGGCACAGACAGGCAGT#CTGCCTTTGGCGCTTGTGTGAAACCAAGGCTGTGCCTGAACTGTG#((((((((((((..((((((.#))))))......................)))))))))..))))).	3957	4002	0.84615	-26	1	0.000572	0.5	0	0	-6	19	45	9	3UTR
mmu-miR-214-3p	XM_006524091	Anks1	ACAGCAGGCACAGACAGGCAGT#ACTGCCACCTTGTGGCTGCTC#..(((((.(((((...((((((#))))))...))))).))))).	3430	3451	0.92308	-25	0	6.15E-07	0.4	0	0	-9	16	21	6	3UTR
mmu-miR-214-3p	XM_006524135	Clic5	ACAGCAGGCACAGACAGGCAGT#GCTTGGCTGTCTGGCCCAATTTCTGCTGC#.((((((((.(((((((.((((#))).).))))))))))).......))))).	1568	1597	1	-26	1	0.0103347	0.5	0	0	-5	19	29	10	CDS
mmu-miR-214-3p	XM_006524135	Clic5	ACAGCAGGCACAGACAGGCAGT#CTGAGATCTTCCTGTTTGTGAAGGCTGG#.((((...((((((((((.((.#.)).))))))))))...)))).	852	880	1	-24	0	0.0004873	0.5	0	0	-10	16	22	10	CDS
mmu-miR-214-3p	XM_006524183	Dlgap1	ACAGCAGGCACAGACAGGCAGT#CTGCCTGCTGTGCTGAGGCTA#..(((.(((((((.(((((((.#))))))))))))))...))).	2658	2679	1	-28	0	5.69E-05	0.5	0	0	-8	17	21	14	5UTR
mmu-miR-214-3p	XM_006524183	Dlgap1	ACAGCAGGCACAGACAGGCAGT#GCTGCCAGTGACTGCCTGTA#...(((((((...(((.((((((#)))))).))...))))))).	4561	4581	0.84615	-24	0	2.55E-05	0.5	0	0	-14	15	20	7	CDS
mmu-miR-214-3p	XM_006524183	Dlgap1	ACAGCAGGCACAGACAGGCAGT#CTGCATCTGTGCCCTCCCTCGCAGCTGTA#(((((.((((((((...((((.#)))).))))))))..........))))).	7161	7190	0.84615	-23	0	0.0030793	0.4	0	0	-7	17	29	8	3UTR
mmu-miR-214-3p	XM_006525541	Camk2a	ACAGCAGGCACAGACAGGCAGT#GCTGCCTCTCCGTGCTTTTGG#.(((.((((((.(((.(((((((#))))))).)).))))))))).	3687	3708	0.92308	-23	0	0.0175643	0.5	0	0	-12	18	21	9	3UTR

mmu-miR-214-3p	XM_006525541	Camk2a	ACAGCAGGCACAGACAGGCAGT#CTGCGTCCTTGCTTCGCAGAGATCCGCTCTTTGTCCGTGGAATGTGGCTGCTGG#.((((((.((((((((((.(((#.))).)))))).......)))).)))))).	1960	2014	1	-23	1	0.0008487	0.5	0	0	-6	19	30	6	3UTR
mmu-miR-214-3p	XM_006526206	Spire1	ACAGCAGGCACAGACAGGCAGT#GCTTCCTGTTCCTCCAGCCACCTTGTGCAGGACCGTTTGTCCTGCTTTCCTGCTGTG#((((((((..(((((((((.((#.)).)))))))).))....))))))	6545	6602	0.92308	-23	1	0.0008571	0.5	0	0	-6	19	27	8	3UTR
mmu-miR-214-3p	XM_006527313	Atad1	ACAGCAGGCACAGACAGGCAGT#GGTGCTTGTCTGACCTTTTTAGCCATATGCTGTT#(((((((((.((((((((((.(#).)))))))))))).........)))...)))))).	1402	1436	0.92308	-24	1	0.0050192	0.6	0	0	-5	20	34	10	3UTR
mmu-miR-214-3p	XM_006527313	Atad1	ACAGCAGGCACAGACAGGCAGT#GCTCCTGGTGTCTGTCTCCTGCA#...(((((...(((((((((.(#.).)))))))))))))).	41	64	1	-22	0	0.0001507	0.5	0	0	-12	15	18	14	5UTR
mmu-miR-214-3p	XM_006527317	Tmem109	ACAGCAGGCACAGACAGGCAGT#GCCATCTCTGTGGCCTGCTT#..((((((((((((..(((...#)))...)))))).)))))).	871	891	0.84615	-25	1	0.0001594	0.4	0	0	-6	15	20	6	CDS
mmu-miR-214-3p	XM_006527317	Tmem109	ACAGCAGGCACAGACAGGCAGT#ACTGGTTGCTGTCCCTGCTC#..((((((.((((.(((.((((#)))).))))))).)))))).	1025	1045	1	-24	1	8.88E-06	0.4	0	0	-7	17	20	7	CDS
mmu-miR-214-3p	XM_006528101	Rtl5	ACAGCAGGCACAGACAGGCAGT#TTGCCTGTCTTCACGGTGCTTCTTAGTCTGAATGCTTGTG#(((((((((((((((((((((.#)))))))))).....))))))............)))).)).	3947	3987	0.92308	-24	1	0.0007276	0.5	0	0	-4	21	40	10	3UTR
mmu-miR-214-3p	XM_006528582	Diaph2	ACAGCAGGCACAGACAGGCAGT#TGTGTGTGTGTGTTTGTGTGTG#(((((((((((.(((.(((..#))).))).))))))))).))).	4833	4855	0.92308	-22	1	0.3357614	0.6	0	0	-5	18	22	9	3UTR
mmu-miR-214-3p	XM_006529077	Hdlbp	ACAGCAGGCACAGACAGGCAGT#GCTCCTGATGCTGTGCTCTGG#....((((((((.((((.(((#)))))))...)))))).))).	4200	4221	0.92308	-21	0	1.60E-05	0.4	0	0	-10	16	21	7	3UTR
mmu-miR-214-3p	XM_006529158	Kif1a	ACAGCAGGCACAGACAGGCAGT#GCTGATGAAGTTAACAACAACACCTGTTCAGCTGTGCCTCCTGA#.(((.((((((((((((....#.)))))....))))))))).))).	3509	3553	0.92308	-24	0	0.0001367	0.5	0	0	-8	16	23	8	CDS
mmu-miR-214-3p	XM_006529288	Sp100	ACAGCAGGCACAGACAGGCAGT#GCTGCCTGTTGTGAGCG#...((...((((.(((((((((#))))))))))))).)).	2202	2219	0.92308	-21	0	0.0001195	0.5	0	0	-14	15	17	13	3UTR

mmu-miR-214-3p	XM_006529723	Atp2b4	ACAGCAGGCACAGACAGGCAGT#GCTGCCCAGGGAAGTCTGTGCCTTTCTGC#.(((.((((((((((.((((((#)))))).......))))))))))..))).	5774	5803	0.92308	-28	0	0.0582564	0.4	0	0	-8	19	29	10	3UTR
mmu-miR-214-3p	XM_006529723	Atp2b4	ACAGCAGGCACAGACAGGCAGT#CTGCAGTTCGGCCTGCTGC#.((((((((.(.(((..((((.#)))).))).))))))))).	7925	7944	0.92308	-24	1	7.09E-05	0.5	0	0	-9	16	19	9	3UTR
mmu-miR-214-3p	XM_006529727	Atp2b4	ACAGCAGGCACAGACAGGCAGT#GCTGCCCAGGGAAGTCTGTGCCTTTCTGC#.(((.((((((((((.((((((#)))))).......))))))))))..))).	5955	5984	0.92308	-28	0	0.0582564	0.4	0	0	-8	19	29	10	3UTR
mmu-miR-214-3p	XM_006529727	Atp2b4	ACAGCAGGCACAGACAGGCAGT#CTGCAGTTCGGCCTGCTGC#.((((((((.(.(((..((((.#)))).))).))))))))).	8106	8125	0.92308	-24	1	7.09E-05	0.5	0	0	-9	16	19	9	3UTR
mmu-miR-214-3p	XM_006529766	Stx6	ACAGCAGGCACAGACAGGCAGT#GCGGACAAACACTGAAGCTCTGAACGGTGCCTGTTCAGGTGGCTGCTGA#.((((((.(((.((((((((.(#.).))))))))...))).)))))).	1713	1762	1	-29	1	0.0002436	0.5	0	0	-9	18	25	8	3UTR
mmu-miR-214-3p	XM_006529845	Steap3	ACAGCAGGCACAGACAGGCAGT#CTGCCTGCCGCTGCGCCGCTC#..(((.(((.(((.(((((((.#)))))))...))).)))))).	1243	1264	0.84615	-25	0	0.0030076	0.3	0	0	-9	16	21	7	CDS
mmu-miR-214-3p	XM_006529845	Steap3	ACAGCAGGCACAGACAGGCAGT#CTGTCCCTGGGTGTGCTGGCCCTGGGCATGTTGTCGCTGCTGG#.(((((((((((..((((((((.#)))..))))..)))))...................)))))).	1361	1404	0.84615	-23	1	0.0014271	0.4	0	0	-6	18	43	6	CDS
mmu-miR-214-3p	XM_006529898	Traf3ip1	ACAGCAGGCACAGACAGGCAGT#ACCGCCCCTCTGAGCCCCAGTCTGCTGAGAAATGGCCTGCTGTG#(((((((((.(((((.(((...#.)))...))))).........))))))))).	2193	2237	0.92308	-26	1	0.0005687	0.4	0	0	-5	17	32	9	3UTR
mmu-miR-214-3p	XM_006530863	Inpp4b	ACAGCAGGCACAGACAGGCAGT#ATTGTCTTCAGTCAGGCGCTTGCTGG#.((((((((.(.((((((((((#)))))))...)))..).)))))))).	1658	1684	0.84615	-23	1	4.19E-05	0.6	0	0	-8	19	26	8	CDS
mmu-miR-214-3p	XM_006530863	Inpp4b	ACAGCAGGCACAGACAGGCAGT#GCTGATCGAAATGTGTCTGCTT#..((((((((((....((((((#)))).))....)))))))))).	3050	3072	1	-22	1	0.00675	0.6	0	0	-8	16	22	10	3UTR
mmu-miR-214-3p	XM_006530975	Def8	ACAGCAGGCACAGACAGGCAGT#ACTGGAGAACGCCAGGCACCGTATCCACCCCTCCGGCTGGTGCCTGCTGG#.(((((((((((.(.((.((.#.)))).)))).))))))))).	1578	1628	0.83333	-25	1	5.23E-09	0.4	0	0	-5	17	21	9	3UTR

mmu-miR-214-3p	XM_006530975	Def8	ACAGCAGGCACAGACAGGCAGT#TTGCCTGAGCTGCCCGCTGG#.((((.((((....(((((((.#)))))))...)))).)))).	348	368	0.84615	-24	0	6.97E-05	0.4	0	0	-14	15	20	7	CDS
mmu-miR-214-3p	XM_006531722	Kdm2a	ACAGCAGGCACAGACAGGCAGT#CTGTGTGTCTGGCTAAGTGTGCTGC#.((((((((.((((((.((((.#)))).))))))))).....))))).	5438	5463	0.92308	-26	1	8.91E-07	0.4	0	0	-5	18	25	9	3UTR
mmu-miR-214-3p	XM_006531722	Kdm2a	ACAGCAGGCACAGACAGGCAGT#ACTGCCTGTCTGATGAGAAACTGA#.(((....((((((((((((((#)))))))))))))).)).....))).	4292	4316	0.92308	-25	0	0.0001653	0.5	0	0	-14	17	24	12	CDS
mmu-miR-214-3p	XM_006531722	Kdm2a	ACAGCAGGCACAGACAGGCAGT#CCTGTAAGGCCTGCTGC#.((((((((.(..(((((....#)))))..))))))))).	2497	2514	0.92308	-24	1	0.0001166	0.4	0	0	-10	14	17	9	CDS
mmu-miR-214-3p	XM_006532178	Ezh1	ACAGCAGGCACAGACAGGCAGT#GCTGGTCTGGTTTACTCCCTGTCTGTAGTACCTGCTGTT#((((((((((((((((((.(((#.))).))))))))))).)..))))))))).	3162	3201	0.92308	-30	1	0.0017932	0.5	0	0	-4	21	27	9	3UTR
mmu-miR-214-3p	XM_006532202	Gas7	ACAGCAGGCACAGACAGGCAGT#CTGCTAATGTCTGCTAATGTCTGGTGG#.((.(((((((((((((((((.#)))))..))))))....)))))).)).	1262	1289	0.92308	-25	0	0.0003429	0.5	0	0	-7	19	27	6	5UTR
mmu-miR-214-3p	XM_006532202	Gas7	ACAGCAGGCACAGACAGGCAGT#GCCTAAAACGCTGAGAGGTTTGTGCCCAGCTGA#.((((.(((((((((...((((#.))))....)))))))))	463	496	1	-25	0	0.0013948	0.5	0	0	-6	17	25	9	5UTR
mmu-miR-214-3p	XM_006532202	Gas7	ACAGCAGGCACAGACAGGCAGT#GCTGCCTGATGGCTTAGCCTGG#.((((((((.((..((((((((#)))))))).)))))).)).)).	7080	7102	0.92308	-23	1	1.27E-06	0.5	0	0	-7	18	22	8	3UTR
mmu-miR-214-3p	XM_006532315	Jup	ACAGCAGGCACAGACAGGCAGT#GCCGGAGACAAAGATGACATCACAGAGCCCGCTGTCTGTGCCCTGCG#...((((((((((((((...((#.))...))))))))))).))).	1515	1562	0.92308	-26	0	0.0005946	0.4	0	0	-7	16	22	11	CDS
mmu-miR-214-3p	XM_006532315	Jup	ACAGCAGGCACAGACAGGCAGT#CTGCTTTTCTGTCCTGGGCTGG#.(((((((.(((((.((((((.#)))))).))))))))..)))).	2591	2613	0.84615	-24	1	4.00E-05	0.4	0	0	-6	18	22	8	3UTR
mmu-miR-214-3p	XM_006532493	Septin4	ACAGCAGGCACAGACAGGCAGT#CCTTTCTGTGAGGACCTGCTGTT#((((((((((((((.(((....#))).))))))....))))))))).	1385	1408	1	-24	1	8.88E-05	0.5	0	0	-5	17	23	8	CDS

mmu-miR-214-3p	XM_006532931	Wscd1	ACAGCAGGCACAGACAGGCAGT#ACTGCTTGTCTGACATCCCATAAATGTGTGTGCTGTG#((((((.((((((((((((((#)))))))))))).............)))).)))))).	3436	3473	1	-31	0	0.0060842	0.5	0	0	-5	21	37	11	3UTR
mmu-miR-214-3p	XM_006532931	Wscd1	ACAGCAGGCACAGACAGGCAGT#ACTGCCAGGATGCCTGTGCTGAGCGGTCTTATGTCTATGCTGG#.((((((((((((.((((((((#))))))....)).)))))))...............))))).	1654	1697	0.84615	-25	1	0.0001596	0.4	0	0	-5	20	43	7	CDS
mmu-miR-214-3p	XM_006532984	Synrg	ACAGCAGGCACAGACAGGCAGT#GCTTCCCTGTCTCAGTCTGCTA#..(((((((..(((((((.(((#)))..)))))))..))))))).	6614	6636	0.92308	-23	1	3.83E-05	0.5	0	0	-9	17	22	7	3UTR
mmu-miR-214-3p	XM_006532984	Synrg	ACAGCAGGCACAGACAGGCAGT#TTGCCTTCTGTCCTGTGGTTGGTGTG#(((.(((.((((((((((((((.#))))))...))).)))).))).))).	6528	6554	0.92308	-23	0	0.0015751	0.4	0	0	-9	19	26	6	3UTR
mmu-miR-214-3p	XM_006533264	Hexdc	ACAGCAGGCACAGACAGGCAGT#GCCTGTGCTCTTCCTGCTGA#.(((((((...((((((((...#)))))).))...))))))).	2283	2303	0.84615	-25	1	0.0015131	0.4	0	0	-9	15	20	7	3UTR
mmu-miR-214-3p	XM_006533264	Hexdc	ACAGCAGGCACAGACAGGCAGT#CTGGCCTGGCTGGGGCTCCCCAAGATGCTGTG#(((((((((.(((.(((((((.#))).)))).)))..)))........)))))).	1636	1668	0.92308	-22	1	0.016682	0.4	0	0	-6	19	32	6	CDS
mmu-miR-214-3p	XM_006533264	Hexdc	ACAGCAGGCACAGACAGGCAGT#GCTGGCAGAGGTACGACCACTTCTCTGTGCTGTGTGAGCTGC#.((((..(((((((((((.(((#.))).).)))).))))))..)))).	1423	1465	0.84615	-22	0	0.000366	0.4	0	0	-7	18	25	6	CDS
mmu-miR-214-3p	XM_006533604	Myo18a	ACAGCAGGCACAGACAGGCAGT#TGCCTCCCCTCTGCTGGGGTGAGGGCTTTCTCTTCTCAGTGCCTGCTGTC#((((((((((((.((..(((((.#.)))))..................)).))))))))))).	6751	6801	0.92308	-26	1	0.0078529	0.6	0	0	-8	18	41	11	3UTR
mmu-miR-214-3p	XM_006533604	Myo18a	ACAGCAGGCACAGACAGGCAGT#CTGCTTGTCCTTAGCACCCGAGGGGCTCCTGCTGTG#((((((((....(((((((((.#)))))))))..................)))))))).	1536	1572	0.84615	-25	1	9.43E-06	0.5	0	0	-12	17	36	9	CDS
mmu-miR-214-3p	XM_006533604	Myo18a	ACAGCAGGCACAGACAGGCAGT#GCTGCTGTCTGGGCCTAAGCCGG#.(.((((((.((((((.(((((#))))))))))).))))..)).).	3685	3708	0.92308	-24	0	6.93E-06	0.4	0	0	-7	18	23	11	CDS

mmu-miR-214-3p	XM_006533622	Rap1gap2	ACAGCAGGCACAGACAGGCAGT#ACTGTCTGTCTTGGGTCGCAGTGCAGTGCCTTCCTGG#.(((.((((((((((((((((#)))))))))))).............))))))..))).	2794	2831	0.89744	-27	0	1.20E-07	0.5	0	0	-8	20	37	11	3UTR
mmu-miR-214-3p	XM_006533622	Rap1gap2	ACAGCAGGCACAGACAGGCAGT#CTGACCGTTTAGGCCTGCTGTC#(((((((((..((((.(((((.#))).))))))..))))))))).	2483	2505	1	-26	1	0.0017136	0.4	0	0	-8	18	22	9	3UTR
mmu-miR-214-3p	XM_006533622	Rap1gap2	ACAGCAGGCACAGACAGGCAGT#ACTGCTAGGGAAGATGTGCCTGCC#...((((((((..(.((((((#))))))..).....))))))))).	1400	1424	1	-25	0	0.0006894	0.4	0	0	-9	16	24	9	CDS
mmu-miR-214-3p	XM_006533622	Rap1gap2	ACAGCAGGCACAGACAGGCAGT#CTGCCTGCAGGCATTACCTCTGGGCTGGATGACTGTGTGCTGA#.(((((.(((((((((((((.#)))))))...........))))...........))).))))).	4299	4342	0.84615	-23	0	0.0076445	0.4	0	0	-5	19	43	7	3UTR
mmu-miR-214-3p	XM_006534036	Slc25a39	ACAGCAGGCACAGACAGGCAGT#GCTGGCGCTCTCTGTGGCTGGGCTGG#.((((((.((((((..(.((((#)))).)....)))))).))..)))).	821	847	0.92308	-23	1	8.33E-06	0.4	0	0	-7	17	26	6	CDS
mmu-miR-214-3p	XM_006534580	Trim16	ACAGCAGGCACAGACAGGCAGT#CTGTTTGTTTGTGCTAAGTTGA#.((((.(((((((((((((((.#)))))))))))))))..)))).	2786	2808	1	-26	0	0.0041643	0.5	0	0	-6	19	22	15	3UTR
mmu-miR-214-3p	XM_006534580	Trim16	ACAGCAGGCACAGACAGGCAGT#AGTGAAATCCTGTCTGACCTGCATGG#.(((((((..((((((((....#.)))))))).))))).)).	439	465	0.96154	-23	1	1.03E-05	0.5	0	0	-6	15	19	8	5UTR
mmu-miR-214-3p	XM_006534580	Trim16	ACAGCAGGCACAGACAGGCAGT#GCGTGTTTCCCTGGCCTGCC#...((((((.((((((.((...#)).)))....))))))))).	3607	3627	1	-22	0	0.0226532	0.6	0	0	-8	14	20	9	3UTR
mmu-miR-214-3p	XM_006535374	Dcun1d1	ACAGCAGGCACAGACAGGCAGT#TGCCTTGCTCTCCTGCTGA#.(((((((...(((((((((..#))))).).)))))))))).	2224	2243	1	-21	1	0.0100913	0.6	0	0	-9	16	19	10	3UTR
mmu-miR-214-3p	XM_006535374	Dcun1d1	ACAGCAGGCACAGACAGGCAGT#GCCATGTTGGCTGCTGA#.((((((.((...((((((...#))).))))).)))))).	1987	2004	1	-20	1	7.09E-05	0.5	0	0	-14	14	17	6	3UTR
mmu-miR-214-3p	XM_006535374	Dcun1d1	ACAGCAGGCACAGACAGGCAGT#ACTGCTAAATCACTGTGGTTGCC#...(((((.(((((...((((((#))))))......))))).)))).	2019	2042	0.84615	-19	0	0.0001805	0.5	0	0	-11	15	23	6	3UTR

mmu-miR-214-3p	XM_006535579	Tbl1xr1	ACAGCAGGCACAGACAGGCAGT#ACTGTGTGCTTTGCTGCTGCTCTCACTGTCCCTGTAGCCTGCTGA#.((((((((((((((((((#.))))).....)))))..))).)))))))).	4899	4944	1	-29	1	0.0037274	0.4	0	0	-4	21	31	8	3UTR
mmu-miR-214-3p	XM_006535580	Tbl1xr1	ACAGCAGGCACAGACAGGCAGT#GCCTGCTGACGTGCCATGATGCTGC#.((((((((((((.(((((...#)))))))...)))))....))))).	4901	4926	1	-24	1	0.0001327	0.4	0	0	-5	17	25	7	3UTR
mmu-miR-214-3p	XM_006535764	Lmbr1	ACAGCAGGCACAGACAGGCAGT#ACTCCACTCTGTGCTTGTTGTA#((((((((((((..((.(((#)))))..)))))))))))))).	2367	2389	1	-27	1	0.002946	0.6	0	0	-5	19	22	14	3UTR
mmu-miR-214-3p	XM_006535764	Lmbr1	ACAGCAGGCACAGACAGGCAGT#GCGTGCAATATTCTTTGCCTGCTGG#.(((((((((.(((...(((((#)).)))).....))).))))))))).	1141	1166	0.92308	-23	1	3.49E-06	0.5	0	0	-8	17	25	9	CDS
mmu-miR-214-3p	XM_006535768	Lmbr1	ACAGCAGGCACAGACAGGCAGT#ACTCCACTCTGTGCTTGTTGTA#((((((((((((..((.(((#)))))..)))))))))))))).	2671	2693	1	-27	1	0.002946	0.6	0	0	-5	19	22	14	3UTR
mmu-miR-214-3p	XM_006535768	Lmbr1	ACAGCAGGCACAGACAGGCAGT#GCGTGCAATATTCTTTGCCTGCTGG#.(((((((((.(((...(((((#)).)))).....))).))))))))).	1445	1470	0.92308	-23	1	3.49E-06	0.5	0	0	-8	17	25	9	CDS
mmu-miR-214-3p	XM_006537909	Rgp1	ACAGCAGGCACAGACAGGCAGT#GCAGGGGTGTGTGTGCTGG#.(((((.(((((..(..((...#))..)..))))).))))).	3072	3091	0.84615	-23	0	1.91E-05	0.3	0	0	-8	13	19	5	3UTR
mmu-miR-214-3p	XM_006538034	Fkbp15	ACAGCAGGCACAGACAGGCAGT#ACAGCCTCTCTGCTGATCCTGTTGTG#((((((((((((((.((((.((#)).)))).)))).))..)))))))).	1115	1141	0.92308	-23	1	0.0039879	0.4	0	0	-5	20	26	8	CDS
mmu-miR-214-3p	XM_006538034	Fkbp15	ACAGCAGGCACAGACAGGCAGT#GCACTTTCAAGATTGCCTAGTTGATGCCTGCA#...(((((((..((((((((((#.))))))).)))..))))))).	4785	4817	0.84615	-27	0	5.96E-05	0.5	0	0	-13	17	22	7	3UTR
mmu-miR-214-3p	XM_006538292	C9orf72	ACAGCAGGCACAGACAGGCAGT#CTGCCCCTCTGTGTTCTGTGGTA#((.(((((((((((..(((((.#)))))..))))))).)))).)).	2121	2144	1	-25	0	0.0022271	0.5	0	0	-6	18	23	7	3UTR
mmu-miR-214-3p	XM_006538292	C9orf72	ACAGCAGGCACAGACAGGCAGT#GCTGCCGTGTGTGCCTGGCTT#..((((((((((....((((((#)))))).))))))))).)).	2583	2602	0.92308	-23	1	3.58E-05	0.5	0	0	-8	16	19	8	3UTR
mmu-miR-214-3p	XM_006538292	C9orf72	ACAGCAGGCACAGACAGGCAGT#CTGCCTGTACTCTGGTGTTTCCCGTGTCACCTGCA#...((((((((((((((((((.#)))))))).))...)))............))))).	2693	2728	0.84615	-22	0	0.0004624	0.5	0	0	-7	18	35	8	3UTR

mmu-miR-214-3p	XM_006538531	Ephb2	ACAGCAGGCACAGACAGGCAGT#GCTGCTGCTGCTGCCGCTGC#.((((.(((((((.((.(((((#))))))))))).)))))))).	206	226	0.84615	-24	0	2.38E-06	0.3	0	0	-7	18	20	10	CDS
mmu-miR-214-3p	XM_006538921	Luzp1	ACAGCAGGCACAGACAGGCAGT#GCTGCCTGTCTGCATGTATCCTTTGGCTGTA#((((((((((((((((((((#))))))))))))..))...)))...)	7345	7376	1	-29	1	0.0001349	0.5	0	0	-4	22	31	12	3UTR
mmu-miR-214-3p	XM_006538940	Plch2	ACAGCAGGCACAGACAGGCAGT#GCTGCCGCTGTGTGGAGGTGGACTGCTGG#.((((((((((((.(.((((((#)))))))))))))))).........))))	1543	1572	0.84615	-26	1	0.0021455	0.3	0	0	-5	19	29	13	CDS
mmu-miR-214-3p	XM_006538940	Plch2	ACAGCAGGCACAGACAGGCAGT#GCTGCCCGTCCTGACCTGCCTCCTGTG#((((.((((((((((.((((((#)))))).))).))...))))).)))).	4044	4071	0.84615	-26	0	0.0287406	0.4	0	0	-8	20	27	6	CDS
mmu-miR-214-3p	XM_006538940	Plch2	ACAGCAGGCACAGACAGGCAGT#GCTGTGTGGATGTGCCAATGCCTGC#.(((((((((((..(((.(((((#))))).))..))))))..))).)).	4645	4670	0.84615	-25	1	4.42E-06	0.4	0	0	-6	18	25	6	CDS
mmu-miR-214-3p	XM_006539060	Espn	ACAGCAGGCACAGACAGGCAGT#GCTGCACTGTCTGCGCTACTTGGTGG#.((.(((((.((((((((((((#))))).))))))).)))...)).)).	350	376	0.92308	-27	0	0.0001681	0.3	0	0	-8	19	26	7	CDS
mmu-miR-214-3p	XM_006539063	Espn	ACAGCAGGCACAGACAGGCAGT#GCTGCACTGTCTGCGCTACTTGGTGG#.((.(((((.((((((((((((#))))).))))))).)))...)).)).	349	375	0.92308	-27	0	0.0001681	0.3	0	0	-8	19	26	7	CDS
mmu-miR-214-3p	XM_006539082	Tmem200b	ACAGCAGGCACAGACAGGCAGT#GCCTGTTCGTGTTCATCTGTGCCAACACATTGCTC#..((((((((((((((((((...#))))))..........))))))))).......)))).	425	460	0.92308	-23	1	0.0014233	0.4	0	0	-5	17	35	8	CDS
mmu-miR-214-3p	XM_006539082	Tmem200b	ACAGCAGGCACAGACAGGCAGT#GCTGCCTGCCCTGCTC#..((((((....(.((((((((#)))))))))))))))).	690	706	1	-22	1	6.83E-06	0.3	0	0	-14	15	16	15	CDS
mmu-miR-214-3p	XM_006539202	Arhgef10l	ACAGCAGGCACAGACAGGCAGT#GGTGCCCCATCCTGGCCTGCTGC#.((((((((.(((...((((.(#).))))).....)))))))))))).	2577	2600	0.84615	-25	1	0.0002516	0.3	0	0	-7	16	23	11	CDS
mmu-miR-214-3p	XM_006539204	Arhgef10l	ACAGCAGGCACAGACAGGCAGT#GGTGCCCCATCCTGGCCTGCTGC#.((((((((.(((...((((.(#).))))).....)))))))))))).	2730	2753	0.84615	-25	1	0.0002516	0.3	0	0	-7	16	23	11	CDS
mmu-miR-214-3p	XM_006539204	Arhgef10l	ACAGCAGGCACAGACAGGCAGT#ACTGCCTCACACACTCAGTACTGTGCTAAGTGGGCTGG#.((((.((((((((((((((((#))))))))..........)).)))))))......)))).	803	841	0.92308	-23	0	3.17E-06	0.5	0	0	-6	20	38	7	5UTR

mmu-miR-214-3p	XM_006539895	Garre1	ACAGCAGGCACAGACAGGCAGT#CTGCGTGGCTGTTGTCTGTGTTAGCTGTC#(((((.((((((((((.(((((#.))))))))))))))))).))))).	5726	5755	0.84615	-27	0	0.0113721	0.5	0	0	-6	20	23	15	3UTR
mmu-miR-214-3p	XM_006540668	Kif7	ACAGCAGGCACAGACAGGCAGT#ACTGCCTGTTTTGCTTCTA#..((.(((((.(((((((((((#)))))))))))))))))).	4223	4242	0.84615	-23	0	0.0005563	0.5	0	0	-12	18	19	18	3UTR
mmu-miR-214-3p	XM_006540668	Kif7	ACAGCAGGCACAGACAGGCAGT#TGACCTGCTGGACTGCCTGGTGC#.((.(((((((((.((((((..#)).)))))))...)))))).)).	533	556	1	-23	0	0.0002488	0.5	0	0	-8	17	23	7	CDS
mmu-miR-214-3p	XM_006541256	Akap13	ACAGCAGGCACAGACAGGCAGT#ACTGCCTGTCCTAGATGCCTGTCTGC#.((((((((((.((((((((((#))))))))))...).)))))).))).	9360	9386	0.92308	-31	1	0.0006084	0.5	0	0	-7	20	26	10	3UTR
mmu-miR-214-3p	XM_006541256	Akap13	ACAGCAGGCACAGACAGGCAGT#GCTGCCTGCTGGCCAGGTGGAGAAATGCAAAGTATTCTAATGCTGC#.((((((((.(((.((((((((#))))))))))))))........................))))).	11948	####	1	-27	1	0.002657	0.5	0	0	-6	19	46	14	3UTR
mmu-miR-214-3p	XM_011239490	Ralgapb	ACAGCAGGCACAGACAGGCAGT#GCTGCCTCAGGGTCGCCCTGTGCCCCCGCTT#..(((.(((((((((((((#)))))))....)))....))))))..	4303	4334	1	-24	0	0.0069695	0.4	0	0	-7	19	31	7	CDS
mmu-miR-214-3p	XM_011239490	Ralgapb	ACAGCAGGCACAGACAGGCAGT#AGTGTCTCTGTGTCTGGCTGA#.(((((((((((((..((((.(#).)))))))))))))).))).	2832	2853	1	-23	1	9.93E-06	0.5	0	0	-5	18	21	14	CDS
mmu-miR-214-3p	XM_011239490	Ralgapb	ACAGCAGGCACAGACAGGCAGT#TGCCTGTGTCCCTTGCTGC#.(((((((...(.(((((((..#))))))).)..))))))).	8257	8276	1	-22	1	0.0002727	0.5	0	0	-10	15	19	7	3UTR
mmu-miR-214-3p	XM_011239617	Ppip5k1	ACAGCAGGCACAGACAGGCAGT#GCTGCCTGGAGTTAACATTGTTTCCTGCTGG#.(((((((.(((..((((((((#))))))))..........)))..))))	399	430	0.92308	-26	1	0.0008092	0.5	0	0	-7	18	31	8	5UTR
mmu-miR-214-3p	XM_011239617	Ppip5k1	ACAGCAGGCACAGACAGGCAGT#CTGCCTAGTCCTGCTTCTGTG#((((.(((((..(((((((((.#)))))).))).))))))))).	5344	5365	0.80769	-23	0	0.0006543	0.6	0	0	-14	18	21	9	3UTR
mmu-miR-214-3p	XM_011239923	Dnajc5	ACAGCAGGCACAGACAGGCAGT#GCTGTTTGTTGTTTGTGGCCTCCTCACCTGCTGC#.((((((((((((((((((((#)))))...)))))))))...........))))))).	527	561	0.92308	-25	1	0.0004061	0.5	0	0	-4	21	34	9	CDS
mmu-miR-214-3p	XM_011240152	Trim46	ACAGCAGGCACAGACAGGCAGT#GCTAGGCCTCTGGTGCCTGCTA#..(((((((((....((((((#)))..))))...))))))))).	2996	3018	0.84615	-24	1	7.74E-06	0.4	0	0	-11	16	22	9	3UTR

mmu-miR-214-3p	XM_011241192	Cacna1c	ACAGCAGGCACAGACAGGCAGT#CCTGCTCTGTGTCTGCC#...((((((((((((((....#)))).)))))))))).	8462	8479	1	-26	0	0.0055044	0.6	0	0	-7	15	17	11	3UTR
mmu-miR-214-3p	XM_011241192	Cacna1c	ACAGCAGGCACAGACAGGCAGT#CTGCCGAAACTACTTCAATATCCTGGACCTGCTGG#.(((((((..(((...(((((.#)))))................)))..))))))).	4143	4178	0.84615	-24	1	0.0010375	0.5	0	0	-8	15	35	7	CDS
mmu-miR-214-3p	XM_011241192	Cacna1c	ACAGCAGGCACAGACAGGCAGT#GCTGTCTTCTACTTCATCAGCTTCTACATGCTCTGTGCCTTCCTGATCATCAACCTCTTTGTAGCTGTT#(((((((((((((((((((((#))))))).....................).))))))))).......................)))	5572	5641	0.84615	-23	1	0.0531543	0.6	0	0	-4	22	69	9	CDS
mmu-miR-214-3p	XM_011241193	Cacna1c	ACAGCAGGCACAGACAGGCAGT#CCTGCTCTGTGTCTGCC#...((((((((((((((....#)))).)))))))))).	8462	8479	1	-26	0	0.0055044	0.6	0	0	-7	15	17	11	3UTR
mmu-miR-214-3p	XM_011241193	Cacna1c	ACAGCAGGCACAGACAGGCAGT#CTGCCGAAACTACTTCAATATCCTGGACCTGCTGG#.(((((((..(((...(((((.#)))))................)))..))))))).	4143	4178	0.84615	-24	1	0.0010375	0.5	0	0	-8	15	35	7	CDS
mmu-miR-214-3p	XM_011241193	Cacna1c	ACAGCAGGCACAGACAGGCAGT#GCTGTCTTCTACTTCATCAGCTTCTACATGCTCTGTGCCTTCCTGATCATCAACCTCTTTGTAGCTGTT#(((((((((((((((((((((#))))))).....................).))))))))).......................)))	5572	5641	0.84615	-23	1	0.0531543	0.6	0	0	-4	22	69	9	CDS
mmu-miR-214-3p	XM_011241482	Setd5	ACAGCAGGCACAGACAGGCAGT#ACTGGCTTCTCCCCATCAGTGCATTGTGCCTGTTGTA#(((((((((((((.((.((((#)))).)))))..............)))))))))))).	5915	5952	0.92308	-25	1	0.0006783	0.4	0	0	-5	20	37	11	3UTR
mmu-miR-214-3p	XM_011241482	Setd5	ACAGCAGGCACAGACAGGCAGT#CTGACCTGAATGGCCTGCCGTC#((.((((((.((..(((((((.#))).))))..)))))))).)).	714	736	0.84615	-24	0	0.0009567	0.4	0	0	-9	17	22	8	CDS
mmu-miR-214-3p	XM_011241749	Dcun1d3	ACAGCAGGCACAGACAGGCAGT#CTGCCTGCTGCCCTGCA#...(((((..(((.(((((((.#)))))))))).))))).	1296	1313	1	-26	0	8.07E-06	0.5	0	0	-10	15	17	10	3UTR
mmu-miR-214-3p	XM_011241749	Dcun1d3	ACAGCAGGCACAGACAGGCAGT#GCTGCCACTGAGGCCTGCC#...((((((.(((...((((((#)))))).)))..)))))).	531	550	0.84615	-25	0	3.89E-07	0.4	0	0	-10	15	19	6	CDS

mmu-miR-214-3p	XM_011241749	Dcun1d3	ACAGCAGGCACAGACAGGCAGT#GCAGGTCTGTACTGTGTGTAGTGCTGTT#((((((.((((((((((((.((#))..)))))).)))))).)...))))).	1701	1729	0.92308	-23	0	0.0009451	0.6	0	0	-5	20	28	6	3UTR
mmu-miR-214-3p	XM_011242247	Apela	ACAGCAGGCACAGACAGGCAGT#ACGAGGTTAAAGCACATGCGTTTGTCTGAATAACAATCTCTCCTGCTGTT#((((((((..(((((((((.((#.)))))))))))............)))))))).	635	685	1	-22	1	0.0292141	0.6	0	0	-6	19	34	11	3UTR
mmu-miR-214-3p	XM_011243284	Baz2a	ACAGCAGGCACAGACAGGCAGT#CTGCCTGGTCTGCCGGAAGGGCGATAATGATGAGTTTCTCCTGCTGTG#((((((((..(((((((((((.#))))))).))))..........................))))))))	5268	5316	0.92308	-27	1	0.0088492	0.5	0	0	-6	19	48	8	CDS
mmu-miR-214-3p	XM_011243284	Baz2a	ACAGCAGGCACAGACAGGCAGT#GCTGTCTGTCTGTCCCAGCA#...((.((.(((((((((((((#))))))))))))).)).)).	5395	5415	0.94872	-27	0	6.60E-05	0.5	0	0	-9	17	20	13	CDS
mmu-miR-214-3p	XM_011243284	Baz2a	ACAGCAGGCACAGACAGGCAGT#TGTCTGTCTGACCACCAGCCCCTGCCTGACTT#..((((((((((((((((((..#)))))))))))............))	2731	2763	1	-25	1	0.0013977	0.4	0	0	-5	18	32	10	CDS
mmu-miR-214-3p	XM_011243284	Baz2a	ACAGCAGGCACAGACAGGCAGT#ACTGCCCTGTGTGTGACTGA#.(((((.((((((...((((((#))))))))))))).)).))).	7348	7368	0.92308	-24	0	3.71E-05	0.4	0	0	-7	17	20	12	3UTR
mmu-miR-214-3p	XM_011243286	Baz2a	ACAGCAGGCACAGACAGGCAGT#CTGCCTGGTCTGCCGGAAGGGCGATAATGATGAGTTTCTCCTGCTGTG#((((((((..(((((((((((.#))))))).))))..........................))))))))	5265	5313	0.92308	-27	1	0.0088492	0.5	0	0	-6	19	48	8	CDS
mmu-miR-214-3p	XM_011243286	Baz2a	ACAGCAGGCACAGACAGGCAGT#GCTGTCTGTCTGTCCCAGCA#...((.((.(((((((((((((#))))))))))))).)).)).	5392	5412	0.94872	-27	0	6.60E-05	0.5	0	0	-9	17	20	13	CDS
mmu-miR-214-3p	XM_011243286	Baz2a	ACAGCAGGCACAGACAGGCAGT#TGTCTGTCTGACCACCAGCCCCTGCCTGACTT#..((((((((((((((((((..#)))))))))))............))	2728	2760	1	-25	1	0.0013977	0.4	0	0	-5	18	32	10	CDS
mmu-miR-214-3p	XM_011243286	Baz2a	ACAGCAGGCACAGACAGGCAGT#ACTGCCCTGTGTGTGACTGA#.(((((.((((((...((((((#))))))))))))).)).))).	7345	7365	0.92308	-24	0	3.71E-05	0.4	0	0	-7	17	20	12	3UTR
mmu-miR-214-3p	XM_011249663	Mecom	ACAGCAGGCACAGACAGGCAGT#GCTGGTTGTCTGCAAGCTTGTG#...((((((.(((((((.((((#)))).))))))))...)))))).	3800	3822	1	-23	0	0.0016131	0.6	0	0	-8	17	22	7	3UTR

mmu-miR-214-3p	XM_011249663	Mecom	ACAGCAGGCACAGACAGGCAGT#CTGCCAGAGAACCTGCTGC#.(((((((........(((((.#)))))......))))))).	1694	1713	0.84615	-20	1	0.0003356	0.4	0	0	-15	12	19	7	CDS
mmu-miR-214-3p	XM_011249663	Mecom	ACAGCAGGCACAGACAGGCAGT#CTGCAGTCCGTGCCC#......(((((.(((..((((.#)))).))).))))).	1646	1661	0.92308	-19	0	0.0064125	0.4	0	0	-19	12	15	5	CDS
mmu-miR-214-3p	XM_011250198	Kcnab2	ACAGCAGGCACAGACAGGCAGT#ACTGCCTGCCTGTGAACCACACCTCAGAGCTAAGCTCCTGCTGG#.((((((((((((.((((((((#)))))))).)))))......................))))))).	5405	5449	0.92308	-32	1	2.06E-06	0.5	0	0	-5	20	44	8	3UTR
mmu-miR-214-3p	XM_017312517	Chd9	ACAGCAGGCACAGACAGGCAGT#ACTAGGTGGTGCCTGCGCTCTAGTGGCGCCTGTCTGGCGCCGGCG#...((.(((.((((((((((.((#.)))))))))))..))).)).	504	549	0.81538	-27	0	6.25E-08	0.3	0	0	-10	16	21	11	5UTR
mmu-miR-214-3p	XM_017313279	Tle3	ACAGCAGGCACAGACAGGCAGT#GCTCGGCCCCCTTCGCCTGTCTGTGTGTGGTA#..(.(((.((((((((((((...#.))))))))))))).)).).	82	114	1	-28	0	1.17E-05	0.4	0	0	-10	15	19	12	5UTR
mmu-miR-214-3p	XM_017313279	Tle3	ACAGCAGGCACAGACAGGCAGT#GCTCGCCGTGGATTTTGCCTGCTGTA#((((((((((.(..((((((((#))).))).))..)..)))))))))).	3891	3917	0.84615	-26	1	0.0093044	0.5	0	0	-9	19	26	10	3UTR
mmu-miR-214-3p	XM_017313279	Tle3	ACAGCAGGCACAGACAGGCAGT#TGCCAAAGTCTGTTTTTCCTGCTGC#.((((((((.((((((.((((..#))))...))))))....))))))).	2860	2885	0.84615	-25	1	0.0167808	0.5	0	0	-5	17	25	7	CDS
mmu-miR-214-3p	XM_017313762	Ncln	ACAGCAGGCACAGACAGGCAGT#GCTGCTTCTGTCCCTCACGCCTGCTGA#.((((((((((((..((((((#))))))))))))).......)))))))).	3527	3554	0.84615	-27	1	9.26E-05	0.3	0	0	-5	19	27	11	3UTR
mmu-miR-214-3p	XM_017313762	Ncln	ACAGCAGGCACAGACAGGCAGT#GCCTTCGTTCTGTGCCTGGACACTGTG#((((((((((((((((((...#))))..)).)))))))))....)))).	1996	2023	0.92308	-26	1	0.0001723	0.4	0	0	-4	19	27	9	CDS
mmu-miR-214-3p	XM_017314262	Gas7	ACAGCAGGCACAGACAGGCAGT#GCTGCCTGATGGCTTAGCCTGG#.((((((((.((..((((((((#)))))))).)))))).)).)).	4710	4732	0.92308	-23	1	1.27E-06	0.5	0	0	-7	18	22	8	3UTR
mmu-miR-214-3p	XM_017314262	Gas7	ACAGCAGGCACAGACAGGCAGT#GCCTGTGGACCAACTGCTGC#.((((((......((((((...#)))))).......)))))).	1290	1310	0.84615	-22	1	0.0010601	0.5	0	0	-16	12	20	6	CDS
mmu-miR-214-3p	XM_017314469	Synrg	ACAGCAGGCACAGACAGGCAGT#GCTTCCCTGTCTCAGTCTGCTA#..(((((((..(((((((.(((#)))..)))))))..))))))).	6873	6895	0.92308	-23	1	3.83E-05	0.5	0	0	-9	17	22	7	3UTR

mmu-miR-214-3p	XM_017314469	Synrg	ACAGCAGGCACAGACAGGCAGT#TTGCCTTCTGTCCTGTGGTTGGTGTG#(((.(((.(((((((((((((.#))))))...))).)))).))).))).	6787	6813	0.92308	-23	0	0.0015751	0.4	0	0	-9	19	26	6	3UTR
mmu-miR-214-3p	XM_017315109	Ttll5	ACAGCAGGCACAGACAGGCAGT#GCCTCTGTGTGCTGACTGTA#((((..((((((.(.((((...#)))).).))))))..)))).	216	236	0.84615	-23	0	0.0007304	0.5	0	0	-12	15	20	6	5UTR
mmu-miR-214-3p	XM_017315109	Ttll5	ACAGCAGGCACAGACAGGCAGT#CCTGCTGTGCGCTCCTGG#.(((.((((((((.((((....#)))))))))).)).))).	4164	4182	1	-22	0	0.0001385	0.4	0	0	-9	15	18	10	3UTR
mmu-miR-214-3p	XM_017315114	Ttll5	ACAGCAGGCACAGACAGGCAGT#GCCTCTGTGTGCTGACTGTA#((((..((((((.(.((((...#)))).).))))))..)))).	216	236	0.84615	-23	0	0.0007304	0.5	0	0	-12	15	20	6	5UTR
mmu-miR-214-3p	XM_017315114	Ttll5	ACAGCAGGCACAGACAGGCAGT#GCGGTTCTGTGCTTGGTCTGTC#((((((((((((((...((...#))...))))))))))..)))).	1617	1639	0.92308	-22	1	0.0019899	0.4	0	0	-5	16	22	10	CDS
mmu-miR-214-3p	XM_017315281	Cry2	ACAGCAGGCACAGACAGGCAGT#GCTGAATTCGCGTCTGTTTGTAGTCCGGGGACAGCCAGCTGA#.((((.((((((((((((.((#.))))))))))))...........))).)))).	315	357	0.92308	-22	0	6.54E-06	0.5	0	0	-6	19	34	12	CDS
mmu-miR-214-3p	XM_017315485	Eif4e1b	ACAGCAGGCACAGACAGGCAGT#CTGCCTGTCTCTGCCTCCTGAGTGCTGG#.(((((((((.((((((((((.#)))))))))).)))))).......)))).	268	296	0.84615	-31	1	9.00E-05	0.5	0	0	-7	19	28	10	5UTR
mmu-miR-214-3p	XM_017315485	Eif4e1b	ACAGCAGGCACAGACAGGCAGT#ACTGCCACAGCTCATCACTGTGCCTGCA#...((((((((((...((((((#))))))...........)))))))))).	1331	1359	0.92308	-27	0	0.0130548	0.5	0	0	-8	16	28	10	3UTR
mmu-miR-214-3p	XM_017315485	Eif4e1b	ACAGCAGGCACAGACAGGCAGT#CTGTGTGTCTCCTCCTGCTGA#.(((((((...((((.((((.#)))).)))))...))))))).	1996	2017	1	-26	1	0.0074721	0.6	0	0	-9	16	21	7	3UTR
mmu-miR-214-3p	XM_017315763	Kcnq2	ACAGCAGGCACAGACAGGCAGT#GCTATGTCTGTTGTCTGCTGTA#(((((((((((((((((((...#))).))))))).)))))))))).	4567	4589	1	-30	1	0.009891	0.5	0	0	-4	19	22	9	3UTR
mmu-miR-214-3p	XM_017315763	Kcnq2	ACAGCAGGCACAGACAGGCAGT#ATTGCCTCCATTGCTGTGCTGGCTGCTGG#.((((((((((((..(((((((#)))))))......))))))...)))))).	1011	1040	0.92308	-26	1	0.0001418	0.4	0	0	-5	19	29	7	CDS
mmu-miR-214-3p	XM_017317120	Lztr1	ACAGCAGGCACAGACAGGCAGT#GCTGCCCACGCTGCGGTTGCTGA#.((((((.(.(((...((((((#))))))....))).).)))))).	2667	2690	0.92308	-24	1	8.61E-07	0.4	0	0	-9	16	23	6	CDS

mmu-miR-214-3p	XM_017317120	Lztr1	ACAGCAGGCACAGACAGGCAGT#GCCAGGGTCTGCTGGCAGATTTTCTAGCCTGCTGA#.((((((((.(((((.(((...#)))...)))))...............)))))))).	3036	3071	0.84615	-24	1	4.04E-05	0.5	0	0	-5	16	35	8	3UTR
mmu-miR-214-3p	XM_017320139	Tut4	ACAGCAGGCACAGACAGGCAGT#CTGCCTATAACTACTGCCAAAGTGCCTATTGTA#((((.((((((.....((((((#.))))))...)))))).))))	3479	3512	0.84615	-22	0	0.0126498	0.6	0	0	-15	16	22	6	CDS
mmu-miR-214-3p	XM_017320397	Arhgef10l	ACAGCAGGCACAGACAGGCAGT#GGTGCCCCATCCTGGCCTGCTGC#.((((((((.(((...((((.(#).)))).....)))))))))))).	2715	2738	0.84615	-25	1	0.0002516	0.3	0	0	-7	16	23	11	CDS
mmu-miR-214-3p	XM_017320397	Arhgef10l	ACAGCAGGCACAGACAGGCAGT#ACTGCCTCACACACTCAGTACTGTGCTAAGTGGGCTGG#.((((.((((((((((((((#)))))))..........)).)))))))......)))).	803	841	0.92308	-23	0	3.17E-06	0.5	0	0	-6	20	38	7	5UTR
mmu-miR-214-3p	XM_030242017	Arnt2	ACAGCAGGCACAGACAGGCAGT#GCTCCATGTGTGACATGCCATGTCTTGTGCACTAAGCTGG#.((((((((((((((((((((#.)).)))).))))).)))).))..)))).	5787	5827	0.84615	-26	1	0.0010159	0.5	0	0	-4	21	29	5	3UTR
mmu-miR-214-3p	XM_030242017	Arnt2	ACAGCAGGCACAGACAGGCAGT#CTGCCTGGTGCAGAAGCTGG#.((((..((((...(((((((.#))))))))))))....)))).	2706	2726	0.92308	-23	0	4.02E-07	0.4	0	0	-13	15	20	11	3UTR
mmu-miR-214-3p	XM_030243257	Zfhx3	ACAGCAGGCACAGACAGGCAGT#GCTGCCTGCCGATCTGGCCCAGCTC#..(((.(((.(((((((((((#))))))))....)))))))..))).	10153	####	0.84615	-25	0	5.15E-05	0.3	0	0	-8	18	25	8	CDS
mmu-miR-214-3p	XM_030243257	Zfhx3	ACAGCAGGCACAGACAGGCAGT#ATTGGCTGAGAGTGCCTTGCTGG#.((((((((((...(((.((((#)))).)))...))))))).)))).	2969	2992	1	-25	1	8.66E-06	0.4	0	0	-9	17	23	6	5UTR
mmu-miR-214-3p	XM_030243857	Gmip	ACAGCAGGCACAGACAGGCAGT#CTGCCTGCCTCTGCCTCCCGAGTGCTGG#.(((((((((.((.(((((((.#))))))).)).)))))).......)))).	3087	3115	0.92308	-26	1	1.35E-05	0.4	0	0	-7	18	28	7	3UTR
mmu-miR-214-3p	XM_030243857	Gmip	ACAGCAGGCACAGACAGGCAGT#GCCTGTCTGCTGGACTCCGGTCACCAGGCTCAGCTGG#.((((.(((.(((((((((...#))))))))))................)))..)))).	2725	2762	0.80769	-23	0	3.32E-06	0.4	0	0	-7	16	37	9	CDS
mmu-miR-214-3p	XM_030244128	Pml	ACAGCAGGCACAGACAGGCAGT#GCTGCCTTGCCTGCA#...(((((((.....((((((((#)))))))))))))))).	324	339	0.84615	-24	0	0.0007218	0.3	0	0	-16	14	15	14	CDS

mmu-miR-214-3p	XM_030244697	Slc38a3	ACAGCAGGCACAGACAGGCAGT#GCTTTCATGTGCCTGTCTGTC#(((((((((((((.((((...#)))))).)))))))).)))).	360	381	1	-24	1	0.0033587	0.5	0	0	-5	18	21	8	5UTR
mmu-miR-214-3p	XM_030244697	Slc38a3	ACAGCAGGCACAGACAGGCAGT#ACTGGCCTGCTTACGTGCATCAATCTGCTGG#.((((((((((((.((((((((#)))).))))))...))))......)	1764	1795	1	-24	1	0.0012247	0.5	0	0	-5	20	31	6	CDS
mmu-miR-214-3p	XM_030244831	Baz2a	ACAGCAGGCACAGACAGGCAGT#CTGCCTGGTCTGCCGGAAGGGCGATAATGATGAGTTTCTCCTGCTGTG#((((((((..(((((((((((.#))))))).))))..........................))))))))	5201	5249	0.92308	-27	1	0.0088492	0.5	0	0	-6	19	48	8	CDS
mmu-miR-214-3p	XM_030244831	Baz2a	ACAGCAGGCACAGACAGGCAGT#GCTGTCTGTCTGTCCCAGCA#...((.((.((((((((((((((#)))))))))))))).)).)).	5328	5348	0.94872	-27	0	6.60E-05	0.5	0	0	-9	17	20	13	CDS
mmu-miR-214-3p	XM_030244831	Baz2a	ACAGCAGGCACAGACAGGCAGT#TGTCTGTCTGACCACCAGCCCCTGCCTGACTT#..((((((((((((((((((..#))))))))))))............))	2664	2696	1	-25	1	0.0013977	0.4	0	0	-5	18	32	10	CDS
mmu-miR-214-3p	XM_030244831	Baz2a	ACAGCAGGCACAGACAGGCAGT#ACTGCCCTGTGTGTGACTGA#.(((((.((((((...((((((#)))))))))))))).)).))).	7281	7301	0.92308	-24	0	3.71E-05	0.4	0	0	-7	17	20	12	3UTR
mmu-miR-214-3p	XM_030245253	Nabp2	ACAGCAGGCACAGACAGGCAGT#ACGCCCCACCCCACCCCTGTCCTGCTGA#.(((((((.((((...((....#.)).))))))))))).	1066	1094	1	-19	1	0.0257125	0.4	0	0	-7	13	16	11	3UTR
mmu-miR-214-3p	XM_030245609	Limk2	ACAGCAGGCACAGACAGGCAGT#TGCCTGTGTGTGTCTAGCC#...(((((((((.(((((((..#))))))).))))))).)).	378	397	0.92308	-27	0	0.0020599	0.4	0	0	-8	16	19	7	5UTR
mmu-miR-214-3p	XM_030247102	Celf1	ACAGCAGGCACAGACAGGCAGT#GCTGCTTTCTGTGGAAATGCTGA#.(((((..((((((.(((((((#))))))))))))))....))))).	2307	2330	1	-25	0	0.0013871	0.6	0	0	-8	18	23	13	3UTR
mmu-miR-214-3p	XM_030247102	Celf1	ACAGCAGGCACAGACAGGCAGT#GCTGCCAGTGCTGTGCTCCCTAACCTTCTGTTCTGCC#...((((((((((((.((((((#)))))).)).))))))...............)))).	3929	3966	0.84615	-24	0	0.1571897	0.4	0	0	-7	18	37	6	3UTR
mmu-miR-214-3p	XM_030247102	Celf1	ACAGCAGGCACAGACAGGCAGT#GCTGCCTTTCTGATTTCCAGCTGC#.((((.(((..((((.(((((((#))))))).))))....)).))))).	7224	7248	1	-24	0	0.0028507	0.6	0	0	-9	17	24	7	3UTR
mmu-miR-214-3p	XM_030247110	Celf1	ACAGCAGGCACAGACAGGCAGT#GCTGCCTTTCTGATTTCCAGCTGC#.((((.(((..((((.(((((((#))))))).))))....)).))))).	4137	4161	1	-24	0	0.0028507	0.6	0	0	-9	17	24	7	3UTR

mmu-miR-214-3p	XM_030247110	Celf1	ACAGCAGGCACAGACAGGCAGT#GCCTGCTGTGTGCCCACAGTCTGACTGC#.((((((((((((.(((((...#)))))))))))).........))).))).	3888	3916	0.84615	-23	1	0.0007356	0.4	0	0	-5	17	28	11	3UTR
mmu-miR-214-3p	XM_030247110	Celf1	ACAGCAGGCACAGACAGGCAGT#CTGCCTGCCTGCGCTTT#.....((((.(((.(((((((.#))))))).))).)))).	3212	3229	0.92308	-22	0	4.61E-05	0.5	0	0	-16	14	17	7	3UTR
mmu-miR-214-3p	XM_030248527	Ywhaz	ACAGCAGGCACAGACAGGCAGT#ACTGCTTCCATGTCTAAGCAAAGAAAACTGCCTACATATTGGTGTGTGCTGG#.(((((.((((....(((((((#.)))))))........)))).))))).	1652	1704	0.92308	-25	0	0.0001701	0.5	0	0	-12	16	27	7	3UTR
mmu-miR-214-3p	XM_030248527	Ywhaz	ACAGCAGGCACAGACAGGCAGT#TGCGTGTGGTGCCCCTGCTGC#.((((((((((..(((.(((..#))).))).)))))..))))).	413	434	0.84615	-24	1	0.0001082	0.4	0	0	-8	16	21	5	5UTR
mmu-miR-214-3p	XM_030248527	Ywhaz	ACAGCAGGCACAGACAGGCAGT#TTGCTTGGTGTGCCCGGCTC#..(((.((((((..((((((((.#)))))))).))))))..))).	2042	2062	0.84615	-23	0	3.22E-05	0.5	0	0	-9	16	20	7	3UTR
mmu-miR-214-3p	XM_030248984	Cd47	ACAGCAGGCACAGACAGGCAGT#GCCTGTCTGCCACAATCCTTGTGTCTGTC#...(((((((((((((((...#))))))))............)))))))).	3636	3665	0.84615	-25	0	0.0110392	0.5	0	0	-7	16	29	8	5UTR
mmu-miR-214-3p	XM_030248990	Strbp	ACAGCAGGCACAGACAGGCAGT#CTGCTTAAGGAATCGGATAATCTGTCTGTCCGTCTGACTGTC#((((((((((...(((((((((.#.))))))))).))))).)))).	2941	2983	1	-25	1	4.56E-05	0.6	0	0	-11	18	22	9	3UTR
mmu-miR-214-3p	XM_030248990	Strbp	ACAGCAGGCACAGACAGGCAGT#GCTGTATGTGTGTGTGTGTATGTA#((((((.(((((.(((.(((((#))))).))).))))).))).))).	3118	3142	0.92308	-23	0	0.027114	0.7	0	0	-7	19	24	5	3UTR
mmu-miR-214-3p	XM_030249980	Slc9a3r2	ACAGCAGGCACAGACAGGCAGT#CTGCCATCCTGCCTCGGGCCTGCTGA#.((((((((.(....((((((.#.))))))..))))))))).	1974	2000	1	-26	1	0.0008569	0.5	0	0	-11	15	19	9	3UTR
mmu-miR-214-3p	XM_030249980	Slc9a3r2	ACAGCAGGCACAGACAGGCAGT#GCCAGCTGTGCCTGGAGGAGCTGTC#((((((((((((((.(.(((...#))).))))))))))......))))).	2214	2239	0.84615	-25	1	1.84E-06	0.4	0	0	-5	17	25	9	3UTR
mmu-miR-214-3p	XM_030250426	Ammecr1l	ACAGCAGGCACAGACAGGCAGT#ACTGCCTCCGGTGCCTGCTGG#.((((((((((....(((((((#)))))))...)))))))))).	1511	1532	0.92308	-32	1	7.66E-06	0.4	0	0	-10	17	21	10	5UTR

mmu-miR-214-3p	XM_030250590	Spire1	ACAGCAGGCACAGACAGGCAGT#GCTTCCTGTTCCTCCAGCCACCTTGTGCAGGACCGTTTGTCCTGCTTTCCTGCTGTG#((((((((..((((((((((.((#.)).)))))))).))....))))))	4651	4708	0.92308	-23	1	0.0008571	0.5	0	0	-6	19	27	8	3UTR
mmu-miR-214-3p	XM_030252287	Pou2f1	ACAGCAGGCACAGACAGGCAGT#CTCCCTGTCTGTGGCTGTC#...((((.((((((((((.((.#)).)))))))))).)))).	8070	8089	1	-27	0	9.14E-05	0.4	0	0	-9	16	19	10	3UTR
mmu-miR-214-3p	XM_030252287	Pou2f1	ACAGCAGGCACAGACAGGCAGT#GCTGCCCTAGTGTGCAGGAGACAGTGTCCTGCTGG#.(((((((((((....((((((#))))))....)))))............)))))).	6718	6753	0.92308	-25	1	2.93E-05	0.4	0	0	-7	17	35	6	3UTR
mmu-miR-214-3p	XM_030252287	Pou2f1	ACAGCAGGCACAGACAGGCAGT#GCTTCCTTCTACTTCCCATGGGAACAGCTGTGCCTGTTGC#.((((((((((((..(((.(((#))).)))..................))))))))))).	3433	3473	0.92308	-24	1	0.0002413	0.5	0	0	-5	18	40	12	3UTR
mmu-miR-214-3p	XM_030252287	Pou2f1	ACAGCAGGCACAGACAGGCAGT#ACTGCTTGGAAGTGGCTGTCCTGTT#((((((((.(((...((((((((#))))))))...))).)))..)))).	11486	####	0.92308	-23	1	6.72E-05	0.5	0	0	-12	18	25	8	3UTR
mmu-miR-214-3p	XM_030252416	Mecom	ACAGCAGGCACAGACAGGCAGT#GCTGGTTGTCTGCAAGCTTGTG#...((((((.((((((((.((((#)))).)))))))...)))))).	5417	5439	1	-23	0	0.0016131	0.6	0	0	-8	17	22	7	3UTR
mmu-miR-214-3p	XM_030252416	Mecom	ACAGCAGGCACAGACAGGCAGT#CTGGCTGCTGCTGATCTTATTTTTGTTTAATTCTGCTGTG#(((((((((((((.(((.(((.#))).)))))).)).................)))))))).	881	921	0.84615	-21	1	0.0231137	0.6	0	0	-5	19	40	8	5UTR
mmu-miR-214-3p	XM_030252416	Mecom	ACAGCAGGCACAGACAGGCAGT#CTGCGGGTCTTGCCCCTGGAGCTGC#.((((.((((.((((..((((.#))))..))))))))......)))).	483	508	0.92308	-20	0	3.19E-06	0.4	0	0	-9	16	25	8	5UTR
mmu-miR-214-3p	XM_030253045	Camta1	ACAGCAGGCACAGACAGGCAGT#GCTGTACCCACTCTGTGCTGCAGCTCCAGAACTGGCGGTGCTGTC#((((((((((((((..((((((#))))...))..))))))))..................)))))).	351	396	0.92308	-22	1	1.74E-06	0.4	0	0	-5	20	45	8	5UTR
mmu-miR-214-3p	XM_030255146	Atp2b4	ACAGCAGGCACAGACAGGCAGT#GCCCCAGTCTGGCCGGAGTTGG#.((((.((((.((((((.(((...#)))...))))))))...)))).	37	59	0.80769	-22	0	4.16E-08	0.4	0	0	-7	15	22	8	5UTR
mmu-miR-214-3p	XM_030255146	Atp2b4	ACAGCAGGCACAGACAGGCAGT#ACGCCTGGCTGTGCA#.......((((((.((((((.((#)))))))).))))))).	1928	1943	0.84615	-20	0	1.46E-05	0.4	0	0	-18	13	15	7	CDS

mmu-miR-214-3p	XM_030255527	Gpr85	ACAGCAGGCACAGACAGGCAGT#GCCTGATTTGGGGCCGGCTGA#.((((.(((.(((((((((...#))))).))))..))).)))).	591	612	0.92308	-25	0	0.0046155	0.4	0	0	-7	16	21	5	5UTR
mmu-miR-214-3p	XM_030255527	Gpr85	ACAGCAGGCACAGACAGGCAGT#CTGCTGGATCTGTGCTGCTC#..((((.((((((((.(((((.#))))).).)))))))))).	1403	1423	1	-24	0	9.77E-07	0.5	0	0	-7	17	20	11	CDS
mmu-miR-214-3p	XM_030255527	Gpr85	ACAGCAGGCACAGACAGGCAGT#GCTGTCTGGATGAGTTTCGCCCAAGCAGGAATCAATCCCTTTGTCTGCA#...((((((((..((((((((#))))))))..))...........................)))))))	2198	2247	0.84615	-22	0	0.0043402	0.5	0	0	-9	17	49	8	CDS
mmu-miR-214-3p	XM_036152258	Gpr85	ACAGCAGGCACAGACAGGCAGT#GCCTGATTTGGGGCCGGCTGA#.((((.(((.(((((((((...#))))).))))..))).)))).	309	330	0.92308	-25	0	0.0046155	0.4	0	0	-7	16	21	5	5UTR
mmu-miR-214-3p	XM_036152258	Gpr85	ACAGCAGGCACAGACAGGCAGT#CTGCTGGATCTGTGCTGCTC#..((((.((((((((.(((((.#))))).).)))))))))).	1107	1127	1	-24	0	9.77E-07	0.5	0	0	-7	17	20	11	CDS
mmu-miR-214-3p	XM_036152258	Gpr85	ACAGCAGGCACAGACAGGCAGT#GCTGTCTGGATGAGTTTCGCCCAAGCAGGAATCAATCCCTTTGTCTGCA#...((((((((..((((((((#))))))))..))...........................)))))))	1902	1951	0.84615	-22	0	0.0043402	0.5	0	0	-9	17	49	8	CDS
mmu-miR-214-3p	XM_036152317	Hnrnpu	ACAGCAGGCACAGACAGGCAGT#ACTGCCTGCCTGGCTGACACTGA#.(((..(((.(((.((((((((#)))))))).))))))....))).	3413	3436	1	-24	0	0.0014154	0.5	0	0	-12	17	23	8	5UTR
mmu-miR-214-3p	XM_036152317	Hnrnpu	ACAGCAGGCACAGACAGGCAGT#TGCCTGTTTGCAGGCTTCCAGCGGAAAGCTGTT#(((((((((.((((((((((..#))))))))))...)))).........))))).	6634	6667	0.92308	-23	1	2.63E-05	0.6	0	0	-5	19	33	10	CDS
mmu-miR-214-3p	XM_036152603	Arnt2	ACAGCAGGCACAGACAGGCAGT#GCTCCATGTGTGACATGCCATGTCTTGTGCACTAAGCTGG#.((((((((((((((((((((#.)).)))).))))).)))).))..)))).	5604	5644	0.84615	-26	1	0.0010159	0.5	0	0	-4	21	29	5	3UTR
mmu-miR-214-3p	XM_036152603	Arnt2	ACAGCAGGCACAGACAGGCAGT#CTGCCTGGTGCAGAAGCTGG#.((((..((((...((((((.#))))))))))....)))).	2523	2543	0.92308	-23	0	4.02E-07	0.4	0	0	-13	15	20	11	3UTR
mmu-miR-214-3p	XM_036152969	Ano5	ACAGCAGGCACAGACAGGCAGT#ACAGCCTGTCAGTGTTCCTGATAGCTGA#.((((((((((.(((((((.((#)).))))))).)))..)))....)))).	2623	2651	0.92308	-23	1	0.0024872	0.5	0	0	-7	19	28	7	CDS

mmu-miR-214-3p	XM_036153721	Dok4	ACAGCAGGCACAGACAGGCAGT#ATTGCCTGGGTCTGTGTTAACTGAGCCCAGTTCTCTCCTGCTA#..((((((((((((((((((#)))))))..)))))))...................)))))).	1826	1869	0.92308	-26	1	0.0080279	0.5	0	0	-5	20	43	7	5UTR
mmu-miR-214-3p	XM_036153721	Dok4	ACAGCAGGCACAGACAGGCAGT#AGTGCCTGTCCTCAGTGATACAACTCTCCTGCTGC#.((((((((((.((((((((.(#).))))))))....)))..........))))))).	4276	4311	1	-26	1	0.0071879	0.4	0	0	-7	19	35	8	3UTR
mmu-miR-214-3p	XM_036153721	Dok4	ACAGCAGGCACAGACAGGCAGT#GCCTTCTTGTGGCTGTTGG#.((((((.((((((.((((...#))))))).))).)))))).	237	256	1	-24	1	0.0002394	0.5	0	0	-7	16	19	7	5UTR
mmu-miR-214-3p	XM_036153774	Znrf1	ACAGCAGGCACAGACAGGCAGT#ACTGACCCTGCTGGGCCTGCTT#..(((((((.(((.((((((((#))))..))))))).))))))).	1716	1738	0.92308	-26	1	6.05E-05	0.4	0	0	-7	18	22	7	CDS
mmu-miR-214-3p	XM_036153774	Znrf1	ACAGCAGGCACAGACAGGCAGT#GCTGTCATGTCCTCTGAATTTGGACCCAGATTGCCAGTGCCTGGTGTG#(((.((((((((.(((((((((((#))))))).))))........................))))))).))).	4141	4189	0.92308	-26	0	4.09E-05	0.4	0	0	-10	20	48	7	3UTR
mmu-miR-214-3p	XM_036153774	Znrf1	ACAGCAGGCACAGACAGGCAGT#CTGCCTGTCTTCTAGCCAGGCTC#..(((.(((..((((((((((.#))))))))))....)))..))).	3277	3300	0.84615	-26	0	0.0001608	0.4	0	0	-11	16	23	10	3UTR
mmu-miR-214-3p	XM_036153954	Inpp4b	ACAGCAGGCACAGACAGGCAGT#ATTGTCTTCAGTCAGGCGCTTGCTGG#.((((((((.(.((((((((((#)))))))...)))..).)))))))).	2457	2483	0.84615	-23	1	4.19E-05	0.6	0	0	-8	19	26	8	CDS
mmu-miR-214-3p	XM_036153958	Inpp4b	ACAGCAGGCACAGACAGGCAGT#ATTGTCTTCAGTCAGGCGCTTGCTGG#.((((((((.(.((((((((((#)))))))...)))..).)))))))).	1531	1557	0.84615	-23	1	4.19E-05	0.6	0	0	-8	19	26	8	CDS
mmu-miR-214-3p	XM_036153958	Inpp4b	ACAGCAGGCACAGACAGGCAGT#GCTGATCGAAATGTGTCTGCTT#..((((((((((.....((((((#)))).))....)))))))))).	2923	2945	1	-22	1	0.00675	0.6	0	0	-8	16	22	10	3UTR
mmu-miR-214-3p	XM_036153975	Mthfsd	ACAGCAGGCACAGACAGGCAGT#CTGCAGTTTAGCAGGATGGAGCTGTCTGTTCTTCCTGCTGC#.(((((((....((((((((((#.))))))))))...))))))).	1343	1384	0.92308	-27	1	2.94E-07	0.4	0	0	-12	17	22	10	3UTR
mmu-miR-214-3p	XM_036153975	Mthfsd	ACAGCAGGCACAGACAGGCAGT#GCTGCTTACTGGCTTGCTC#..(((((((.(((..(((((((#))))))).)))))))))).	2172	2191	1	-23	1	0.0002427	0.5	0	0	-8	17	19	10	3UTR

mmu-miR-214-3p	XM_036153983	Def8	ACAGCAGGCACAGACAGGCAGT#ACTGGAGAACGCCAGGCACCGTATCCACCCCTCCGGCTGGTGCCTGCTGG#.((((((((((((.(.((.((.#.)))).)))).))))))))).	1528	1578	0.83333	-25	1	5.23E-09	0.4	0	0	-5	17	21	9	3UTR
mmu-miR-214-3p	XM_036153983	Def8	ACAGCAGGCACAGACAGGCAGT#TTGCCTGAGCTGCCCGCTGG#.((((.((((....(((((((.#)))))))...)))).)))).	298	318	0.84615	-24	0	6.97E-05	0.4	0	0	-14	15	20	7	CDS
mmu-miR-214-3p	XM_036154059	Slc27a1	ACAGCAGGCACAGACAGGCAGT#TTGGCTGCTGTGTCCTGCA#...((((((((((.(((.(((.#))).)))))))).))))).	2156	2175	0.92308	-25	0	3.19E-07	0.4	0	0	-7	16	19	8	3UTR
mmu-miR-214-3p	XM_036154286	Clasp1	ACAGCAGGCACAGACAGGCAGT#CCTGTCTGGCCCAGGTGCTGC#.((((((((.((((((((....#)))))))))))....))))).	2579	2600	1	-26	1	3.77E-06	0.4	0	0	-5	16	21	11	CDS
mmu-miR-214-3p	XM_036154894	Dmxl2	ACAGCAGGCACAGACAGGCAGT#ACTGCAATTCTGCAAGTGCTTGCTT#..(((((((((...((((((((#)))))....)))...))))))))).	2432	2457	0.84615	-22	1	0.0001371	0.6	0	0	-10	17	25	9	CDS
mmu-miR-214-3p	XM_036155552	Ank3	ACAGCAGGCACAGACAGGCAGT#GCTGTCTAATCCCCAGTCACAGGGGCCCTGTCTGTGCCTCCA#...(.(((((((((((((....#.))))))))))))).).	7183	7225	0.84615	-26	0	0.0015804	0.5	0	0	-11	14	17	13	3UTR
mmu-miR-214-3p	XM_036155552	Ank3	ACAGCAGGCACAGACAGGCAGT#ACTGCCCTCCATGTGGCTGCC#...((((.((((....((((((#)))))).....)))).)))).	1381	1402	0.84615	-23	0	7.73E-05	0.4	0	0	-12	14	21	6	CDS
mmu-miR-214-3p	XM_036155993	Gcc2	ACAGCAGGCACAGACAGGCAGT#CTGCCTGTGATAGACACCATGCTGC#.(((((((.....((((((((.#))))))))........)).))))).	5163	5188	0.84615	-22	1	0.0193368	0.5	0	0	-14	15	25	8	CDS
mmu-miR-214-3p	XM_036156594	Atxn7l3	ACAGCAGGCACAGACAGGCAGT#ACTGCCTCTGGCCTGG#....(((((.((((..((((((#)))))))))))))))).	942	958	1	-24	0	8.59E-05	0.5	0	0	-12	15	16	15	CDS
mmu-miR-214-3p	XM_036156594	Atxn7l3	ACAGCAGGCACAGACAGGCAGT#CTGCCCTGCCCTGCTGG#.(((((((..(((...(((((.#)))))))).))))))).	1920	1937	0.84615	-23	1	0.0001454	0.3	0	0	-8	15	17	8	3UTR
mmu-miR-214-3p	XM_036157037	Mtmr3	ACAGCAGGCACAGACAGGCAGT#GCTGCCTGAGTGGGCACATGTCTGACTCCTGTT#((((((((((((..((((((((#))))))))..))......))))))....)))).	4643	4676	0.92308	-23	1	0.0103716	0.5	0	0	-6	20	33	8	3UTR

mmu-miR-214-3p	XM_036157037	Mtmr3	ACAGCAGGCACAGACAGGCAGT#GCATGCCTGTGTACACGGACACAATCCAACAGCGCCTGCG#...((((((.((.((((((((((#)).)))))))).)..................).)))))).	3240	3280	0.84615	-22	0	0.0074499	0.4	0	0	-9	17	40	7	CDS
mmu-miR-214-3p	XM_036157038	Mtmr3	ACAGCAGGCACAGACAGGCAGT#GCTGCCTGAGTGGGCACATGTCTGACTCCTGTT#((((((((((((..((((((((#))))))))..))......))))))....))))).	4705	4738	0.92308	-23	1	0.0103716	0.5	0	0	-6	20	33	8	3UTR
mmu-miR-214-3p	XM_036157038	Mtmr3	ACAGCAGGCACAGACAGGCAGT#GCGCGCTCTGTGTGGCCGCTGC#.((((.((((.((.((((((.((#))..)).)))).))))))))).	62	84	0.92308	-22	0	0.0014596	0.4	0	0	-8	17	22	9	5UTR
mmu-miR-214-3p	XM_036157038	Mtmr3	ACAGCAGGCACAGACAGGCAGT#GCATGCCTGTGTACACGGACACAATCCAACAGCGCCTGCG#...((((((.((.((((((((((#)).)))))))).)..................).)))))).	3391	3431	0.84615	-22	0	0.0074499	0.4	0	0	-9	17	40	7	CDS
mmu-miR-214-3p	XM_036157234	Nrxn3	ACAGCAGGCACAGACAGGCAGT#GCTGCCTGGCCTCTGTGGACTTGAATGGACGCCTGCCTGA#.((((((((((((((((((((#))))))))...)))))..............)))))).)).	4134	4174	0.84615	-28	1	0.0070194	0.4	0	0	-4	21	40	8	CDS
mmu-miR-214-3p	XM_036157234	Nrxn3	ACAGCAGGCACAGACAGGCAGT#CTGCTCCTCTGTGCTCTGA#....((((((((((..(((((.#)))))..))))))).))).	1080	1099	1	-23	0	0.0043441	0.4	0	0	-11	15	19	7	5UTR
mmu-miR-214-3p	XM_036157883	Mak	ACAGCAGGCACAGACAGGCAGT#CTGCCCGTCTCGTTTTAGTCCTGTTGTT#((((((((((((((((.(((((.#))))).)))).))....).)))))))).	2922	2950	1	-24	1	0.0220696	0.5	0	0	-4	20	28	8	3UTR
mmu-miR-214-3p	XM_036157883	Mak	ACAGCAGGCACAGACAGGCAGT#GCCTGTCTCCAGTGTAGCA#...((..(((((((((((...#))))))))...)))).)).	261	280	0.84615	-21	0	0.0001661	0.6	0	0	-10	14	19	8	5UTR
mmu-miR-214-3p	XM_036158016	Celf1	ACAGCAGGCACAGACAGGCAGT#CTGTCTGTCTGTGTACATTTCTGTG#((((...((((((((((((((.#))))))))))))))......)))).	56	81	1	-27	0	0.0051261	0.6	0	0	-12	18	25	14	5UTR
mmu-miR-214-3p	XM_036158016	Celf1	ACAGCAGGCACAGACAGGCAGT#GCTGCTTTCTGTGGAAATGCTGA#.(((((..((((((.(((((((#))))))))))))....))))).	2181	2204	1	-25	0	0.0013871	0.6	0	0	-8	18	23	13	3UTR
mmu-miR-214-3p	XM_036158016	Celf1	ACAGCAGGCACAGACAGGCAGT#GCTGCCAGTGCTGTGCTCCCTAACCTTCTGTTCTGCC#...((((((((((((.((((((#)))))).)).))))))...............)))).	3803	3840	0.84615	-24	0	0.1571897	0.4	0	0	-7	18	37	6	3UTR

mmu-miR-214-3p	XM_036158574	Pcdh20	ACAGCAGGCACAGACAGGCAGT#AC<br>TGGCTTGTTCTTTCACTGAAGTGCCT<br>GCTA#..((((((((((((((((((((#)))).))))).)).........)	4561	4593	0.92308	-25	1	0.0004048	0.6	0	0	-5	20	32	9	3UTR
mmu-miR-214-3p	XM_036158574	Pcdh20	ACAGCAGGCACAGACAGGCAGT#TT<br>GCCTTTTGCTTCTGGACGTGCTGG#.((<br>(((.(..((((.((((((.#))))))......))))..).))))).	810	836	0.84615	-21	0	0.001312	0.5	0	0	-8	16	26	6	CDS
mmu-miR-214-3p	XM_036158897	Glt8d1	ACAGCAGGCACAGACAGGCAGT#GC<br>TGCTGTAGGTAGGAACTGCTGTT#((((<br>(((..((..(((.(((((#))))))))..)).....))))))).	1493	1518	0.84615	-20	1	8.46E-05	0.6	0	0	-12	17	25	8	3UTR
mmu-miR-214-3p	XM_036158897	Glt8d1	ACAGCAGGCACAGACAGGCAGT#GC<br>TGCCTCTGAAGACAGGCTGG#.((((..(((.(<br>(((..((((((#))))))))))..).)..)))).	356	378	0.84615	-19	0	9.34E-05	0.5	0	0	-9	16	22	10	CDS
mmu-miR-214-3p	XM_036159059	Lmf2	ACAGCAGGCACAGACAGGCAGT#AC<br>TGCTGGAACTGACTGTCTATGGGCTG<br>CTGG#.((((((.(..((((((.((((#.)))).))))))...).)))))	1126	1158	0.92308	-25	1	5.24E-05	0.5	0	0	-10	17	24	6	CDS
mmu-miR-214-3p	XM_036159059	Lmf2	ACAGCAGGCACAGACAGGCAGT#CT<br>GTCTGCTGAGCCTGG#....(((((.(((.(((((((.<br>#)))))))))).))))).	1046	1063	0.92308	-24	0	0.0015867	0.4	0	0	-12	15	17	10	CDS
mmu-miR-214-3p	XM_036159059	Lmf2	ACAGCAGGCACAGACAGGCAGT#AC<br>TGCTCTGACCTACCACTATGAGACTC<br>AGTGCCTGCC#...((((((((.((((((((((#))))).))<br>)................)).)))))))).	767	805	0.82692	-24	0	0.0080893	0.4	0	0	-10	18	38	8	CDS
mmu-miR-214-3p	XM_036159060	Lmf2	ACAGCAGGCACAGACAGGCAGT#AC<br>TGCTGGAACTGACTGTCTATGGGCTG<br>CTGG#.((((((.(..((((((.((((#.)))).))))))...).)))))	1640	1672	0.92308	-25	1	5.24E-05	0.5	0	0	-10	17	24	6	CDS
mmu-miR-214-3p	XM_036159060	Lmf2	ACAGCAGGCACAGACAGGCAGT#CT<br>GTCTGCTGAGCCTGG#....(((((.(((.(((((((.<br>#)))))))))).))))).	1560	1577	0.92308	-24	0	0.0015867	0.4	0	0	-12	15	17	10	CDS
mmu-miR-214-3p	XM_036159060	Lmf2	ACAGCAGGCACAGACAGGCAGT#AC<br>TGCTCTGACCTACCACTATGAGACTC<br>AGTGCCTGCC#...((((((((.((((((((((#))))).))<br>)................)).)))))))).	1281	1319	0.82692	-24	0	0.0080893	0.4	0	0	-10	18	38	8	CDS
mmu-miR-214-3p	XM_036159209	Oxr1	ACAGCAGGCACAGACAGGCAGT#CT<br>GCCTGTTCCAAGGGCGGAGCAGAAG<br>CAGGCCGTGGTCCTGCC#...(((((..(((((((<br>((((.#)))))))))........................))..))))).	108	152	0.84615	-22	0	8.90E-06	0.4	0	0	-9	16	44	9	5UTR

mmu-miR-214-3p	XM_036159354	Tmem184b	ACAGCAGGCACAGACAGGCAGT#GCTCTGTGGACCCTCCCCCTCTCCCAACCTAGGCCTGTCTGGCATGCTGC#.(((((.((.(((((((((...#.)))))))))))).))))).	1879	1929	1	-30	0	5.95E-05	0.3	0	0	-7	16	19	11	3UTR
mmu-miR-214-3p	XM_036159653	Trps1	ACAGCAGGCACAGACAGGCAGT#CTGTTTTAATCTCACCTGTCTGTGGCATAGCTC#..(((.(.((((((((((....#.)))))))))).)...))).	5666	5699	0.89744	-23	0	0.0005859	0.6	0	0	-9	14	20	10	3UTR
mmu-miR-214-3p	XM_036159653	Trps1	ACAGCAGGCACAGACAGGCAGT#TGCTTTCTGGCGTGCTGC#.(((((.((.((((.(((((.#))))))))))).))))).	947	965	1	-23	0	1.08E-05	0.5	0	0	-7	16	18	11	CDS
mmu-miR-214-3p	XM_036160058	Zbtb20	ACAGCAGGCACAGACAGGCAGT#ACCACCAGTCTGTTGCCTGTTGC#.((((((((((((((.((..((#))..)).)))))).)))))))).	172	195	1	-26	1	0.0002084	0.4	0	0	-4	18	23	8	5UTR
mmu-miR-214-3p	XM_036160058	Zbtb20	ACAGCAGGCACAGACAGGCAGT#GCTGCTTGCTGCTGCTGCTGA#.(((((.((((((.((((((((#)))))))))))).)))))))).	24268	####	1	-27	0	0.000866	0.5	0	0	-6	19	21	11	3UTR
mmu-miR-214-3p	XM_036160064	Zbtb20	ACAGCAGGCACAGACAGGCAGT#GCTGCTTGCTGCTGCTGCTGA#.(((((.((((((.((((((((#)))))))))))).)))))))).	24319	####	1	-27	0	0.000866	0.5	0	0	-6	19	21	11	3UTR
mmu-miR-214-3p	XM_036160064	Zbtb20	ACAGCAGGCACAGACAGGCAGT#GCTGTCTTCTGTTACTGTTGG#.((((((..(((((.(((((((#)))))))))))))..)))))).	25305	####	0.84615	-24	1	0.0049171	0.6	0	0	-8	18	21	12	3UTR
mmu-miR-214-3p	XM_036160064	Zbtb20	ACAGCAGGCACAGACAGGCAGT#TGTCTGTCTGTGAGTGTA#...(((..((((((((((((.#))))))))))))..))).	9639	9657	1	-24	0	0.0001046	0.5	0	0	-10	15	18	12	3UTR
mmu-miR-214-3p	XM_036160132	Naa50	ACAGCAGGCACAGACAGGCAGT#GCTTCCACTCTGTGCCTACAATGTTGA#.((((((((((((((..((.(((#))).))..))))))))))).....)))).	1787	1814	0.92308	-22	1	0.0282815	0.6	0	0	-5	18	27	9	3UTR
mmu-miR-214-3p	XM_036160132	Naa50	ACAGCAGGCACAGACAGGCAGT#CTGTGGTACTTTGAGTTATACAGACTGTCTGTGGGGTGCTA#......(((((..(((((((((#.)))))))))...))))).	1065	1106	1	-21	0	0.0001437	0.5	0	0	-20	14	19	9	3UTR
mmu-miR-214-3p	XM_036160132	Naa50	ACAGCAGGCACAGACAGGCAGT#AGTGATCTGTCTGTAACTGTC#...((((..((((((((((((.(#).)).)))))))))..)))).	3469	3490	1	-20	0	0.0056227	0.7	0	0	-10	16	21	9	3UTR
mmu-miR-214-3p	XM_036160426	Sox8	ACAGCAGGCACAGACAGGCAGT#ACTCCGTCAAGTGCCTGCTGA#.((((((((((.(((.(((.(((#)))))))))..)))))))))).	1711	1732	1	-26	1	5.79E-05	0.4	0	0	-7	18	21	10	3UTR

mmu-miR-214-3p	XM_036160518	Anks1	ACAGCAGGCACAGACAGGCAGT#CTGCGGGCTGTCTGCCTGCTGC#.((((((((..((((((((((.#))))...)))))))))))))).	4758	4780	1	-28	1	8.56E-09	0.4	0	0	-8	18	22	14	3UTR
mmu-miR-214-3p	XM_036160518	Anks1	ACAGCAGGCACAGACAGGCAGT#CTGCCTCTCTCTGATGCTTGTTGA#.(((((((((((((.((((((.#))))))...)))).))))))))).	4287	4311	1	-27	1	0.0053076	0.4	0	0	-5	19	24	9	3UTR
mmu-miR-214-3p	XM_036160518	Anks1	ACAGCAGGCACAGACAGGCAGT#CTGCCTTTGGCGCTTGTGTGAAACCAAGGCTGTGCCTGAACTGTG#((((((((((((..((((((.#))))))......................)))))))))..)))).	5233	5278	0.84615	-26	1	0.000572	0.5	0	0	-6	19	45	9	3UTR
mmu-miR-214-3p	XM_036160518	Anks1	ACAGCAGGCACAGACAGGCAGT#GCTGCCTGCCTTTCCTTGTGCCTTTCTGA#.(((.((((((((((((((((#))))))))....))..)))))))..))).	3438	3467	0.84615	-26	0	0.0097506	0.4	0	0	-8	20	29	8	3UTR
mmu-miR-214-3p	XM_036161268	Spire1	ACAGCAGGCACAGACAGGCAGT#GCTTCCTGTTCCTCCAGCCACCTTGTGCAGGACCGTTTGTCCTGCTTTCCTGCTGTG#((((((((..(((((((((.((#.)).)))))))).))....))))))	4494	4551	0.92308	-23	1	0.0008571	0.5	0	0	-6	19	27	8	3UTR
mmu-miR-214-3p	XM_036161434	Gcnt1	ACAGCAGGCACAGACAGGCAGT#ACTGGCTGTTGGAAAGCCTGCTGG#.((((((((...(((((.((((#)))).)))))).....)))))))).	688	712	0.92308	-29	1	8.58E-05	0.4	0	0	-11	17	24	8	5UTR
mmu-miR-214-3p	XM_036161434	Gcnt1	ACAGCAGGCACAGACAGGCAGT#CTGTCTGCTGTGGCTGG#....(((.(((((.(((((((.#)))))))))))))).))).	561	578	1	-25	0	2.44E-05	0.5	0	0	-13	15	17	12	5UTR
mmu-miR-214-3p	XM_036161513	Phf20	ACAGCAGGCACAGACAGGCAGT#GCTTGCTTGCTGGTCTGACTGTC#(((((((((.(((.((((((((#))).))))))))))))))).)))).	278	301	1	-24	1	3.70E-05	0.5	0	0	-6	20	23	13	5UTR
mmu-miR-214-3p	XM_036161513	Phf20	ACAGCAGGCACAGACAGGCAGT#CTGCCTGGTGTGTCGTGTGGG#.(.(((((((((..(((((((.#))))))).)))))).))).).	3433	3454	0.84615	-23	0	0.0026428	0.4	0	0	-7	17	21	7	3UTR
mmu-miR-214-3p	XM_036161513	Phf20	ACAGCAGGCACAGACAGGCAGT#GCCTGAATGTCCCTGCAGTG#((.(((((.(((..(((((...#)))))..))).))))).)).	1644	1664	0.92308	-23	0	0.005504	0.6	0	0	-9	15	20	5	CDS
mmu-miR-214-3p	XM_036161513	Phf20	ACAGCAGGCACAGACAGGCAGT#CTGTGACGTTGAATGTGCCAGCTGTG#(((((.((((((..(((((((.#))))....)))..)))))).))))).	1301	1327	1	-23	0	0.0001585	0.5	0	0	-8	18	26	6	CDS
mmu-miR-214-3p	XM_036161517	Vldlr	ACAGCAGGCACAGACAGGCAGT#TGTCTGTCTTTGCTCCTGCTA#..((((((((.(((((((((..#))))))))).)))..))))).	7117	7138	1	-24	1	0.0004164	0.6	0	0	-8	17	21	9	3UTR

mmu-miR-214-3p	XM_036161517	Vldlr	ACAGCAGGCACAGACAGGCAGT#GCTGTCTTAGTACTGTCACTGTTGTA#(((((((..((((((((((((((#))))))))..)).))))..)))))))).	8028	8054	0.84615	-23	1	0.0338413	0.5	0	0	-7	20	26	7	3UTR
mmu-miR-214-3p	XM_036161517	Vldlr	ACAGCAGGCACAGACAGGCAGT#GCTGCCATGGTCTAGGGTGAGGGTGCTGTC#((((((..(((((((.((((((#))))))...))))...)))....))	7360	7390	0.92308	-23	0	0.0004878	0.5	0	0	-8	19	30	6	3UTR
mmu-miR-214-3p	XM_036161622	Zfp950	ACAGCAGGCACAGACAGGCAGT#TGTGCTTGCTGTC#((((((((((((.........#))))))))))))).	1484	1497	1	-20	1	0.0006299	0.7	0	0	-7	12	13	12	3UTR
mmu-miR-214-3p	XM_036161820	Tsc22d3	ACAGCAGGCACAGACAGGCAGT#GCTTAGAAGTGCCTGCCTGG#.((((((((((....((((...#))))....)))))))).)).	554	574	1	-21	1	3.69E-05	0.5	0	0	-10	14	20	8	5UTR
mmu-miR-214-3p	XM_036162086	Asb11	ACAGCAGGCACAGACAGGCAGT#GCTGCGTGTGTCAATGTGCTGTTGG#.(((((.(((((((((.(((((#)))))...))))..)))))))))).	678	703	0.86538	-23	0	0.0097838	0.4	0	0	-5	19	25	10	CDS
mmu-miR-214-3p	XM_036162086	Asb11	ACAGCAGGCACAGACAGGCAGT#ACATGTAGTTTATTGCCTGCTGTA#((((((((((.((((..(((((#)).))).))))..)))))))))).	1227	1251	0.92308	-22	1	3.13E-05	0.6	0	0	-7	19	24	10	3UTR
mmu-miR-214-3p	XM_036162335	Aff3	ACAGCAGGCACAGACAGGCAGT#GCTGCCTGTGTATGTGTGTA#...(((.(((.(.((((((((((#))))))))).).))).))).	4048	4068	1	-24	0	0.0146111	0.6	0	0	-12	16	20	9	3UTR
mmu-miR-214-3p	XM_036162335	Aff3	ACAGCAGGCACAGACAGGCAGT#ACTGTGACTCTGTGGCTGTG#...((((.((((((...((((((#)))))...)))))).)))).	4671	4691	0.84615	-22	0	1.76E-06	0.5	0	0	-10	15	20	6	3UTR
mmu-miR-214-3p	XM_036162335	Aff3	ACAGCAGGCACAGACAGGCAGT#CTGCTGTGCACTGCA#...((((((((((.(((.....#)))))))))).)))).	965	980	0.84615	-22	0	8.19E-06	0.5	0	0	-7	13	15	9	CDS
mmu-miR-214-3p	XM_036162888	Mecom	ACAGCAGGCACAGACAGGCAGT#AGTGTGTGCGTGTGTGTGCGTGTG#...(((.(((((.(((.(((..#.))).))).))))).))).	150	174	0.84615	-24	0	0.0013638	0.5	0	0	-9	14	19	5	5UTR
mmu-miR-214-3p	XM_036162888	Mecom	ACAGCAGGCACAGACAGGCAGT#GCTGGTTGTCTGCAAGCTTGTG#...((((((.((((((((.((((#)))).)))))))...)))))).	4947	4969	1	-23	0	0.0016131	0.6	0	0	-8	17	22	7	3UTR
mmu-miR-214-3p	XM_036162891	Mecom	ACAGCAGGCACAGACAGGCAGT#GCTGGTTGTCTGCAAGCTTGTG#...((((((.((((((((.((((#)))).)))))))...)))))).	5409	5431	1	-23	0	0.0016131	0.6	0	0	-8	17	22	7	3UTR

mmu-miR-214-3p	XM_036162891	Mecom	ACAGCAGGCACAGACAGGCAGT#CTGGCTGCTGCTGATCTTATTTTTGTTTAATTCTGCTGTG#(((((((((((((.(((.(((.#))).)))))).)).................)))))))).	900	940	0.84615	-21	1	0.0231137	0.6	0	0	-5	19	40	8	5UTR
mmu-miR-214-3p	XM_036162891	Mecom	ACAGCAGGCACAGACAGGCAGT#CTGCGGGTCTTGCCCCTGGAGCTGC#.((((.((((.((((..((((.#))))..))))))))......)))).	502	527	0.92308	-20	0	3.19E-06	0.4	0	0	-9	16	25	8	5UTR
mmu-miR-214-3p	XM_036162892	Mecom	ACAGCAGGCACAGACAGGCAGT#GCTGGTTGTCTGCAAGCTTGTG#...((((((.(((((((.((((#)))).))))))))...)))))).	5206	5228	1	-23	0	0.0016131	0.6	0	0	-8	17	22	7	3UTR
mmu-miR-214-3p	XM_036162892	Mecom	ACAGCAGGCACAGACAGGCAGT#CTGGCTGCTGCTGATCTTATTTTTGTTTAATTCTGCTGTG#(((((((((((((.(((.(((.#))).)))))).)).................)))))))).	881	921	0.84615	-21	1	0.0231137	0.6	0	0	-5	19	40	8	5UTR
mmu-miR-214-3p	XM_036162892	Mecom	ACAGCAGGCACAGACAGGCAGT#CTGCGGGTCTTGCCCCTGGAGCTGC#.((((.((((.((((..((((.#))))..))))))))......)))).	483	508	0.92308	-20	0	3.19E-06	0.4	0	0	-9	16	25	8	5UTR
mmu-miR-214-3p	XM_036162949	Nlgn1	ACAGCAGGCACAGACAGGCAGT#TGCTTGGTGGCTGTGCTGCTGG#.(((((.((((((.((((((..#))))))....))))))))))).	3764	3786	1	-24	0	2.00E-05	0.6	0	0	-6	17	22	11	3UTR
mmu-miR-214-3p	XM_036162949	Nlgn1	ACAGCAGGCACAGACAGGCAGT#CTGCTTGTTGGGATGTTTGCTA#..(((((((((.(((((((((.#)))))))))..).))))))))).	659	681	0.92308	-23	1	0.0004424	0.5	0	0	-8	18	22	9	CDS
mmu-miR-214-3p	XM_036162949	Nlgn1	ACAGCAGGCACAGACAGGCAGT#GCGGCATCTGTCTGTGTGAAATCAATGGGAGCTACTTACTGCATGA#.((((((((((((((((((.((#)).))..))))))))))).....................)))).)).	384	430	0.84615	-21	1	0.0065081	0.6	0	0	-4	20	46	10	5UTR
mmu-miR-214-3p	XM_036163954	Zbtb8b	ACAGCAGGCACAGACAGGCAGT#CTGCCTGTCTGTCTGTATGTCTGTGTGTC#(((((((((((((((((((((.#)))))))))))))......))))))).))).	162	191	0.84615	-29	1	0.2859923	0.5	0	0	-4	21	29	12	5UTR
mmu-miR-214-3p	XM_036163954	Zbtb8b	ACAGCAGGCACAGACAGGCAGT#TGCCTGTCAACCTTAGCCTGGTGG#.((.((((((...((((((((..#)))))))).......))))).)).	3003	3027	0.89744	-25	0	0.0001994	0.5	0	0	-14	15	24	8	CDS
mmu-miR-214-3p	XM_036164017	Cyb5rl	ACAGCAGGCACAGACAGGCAGT#ACTTCCCTTCCTGTCTGCTGCGTCCTGCTGTA#(((((((((.(((.(((((((.#.)))))))))).).))))))))).	1062	1094	0.92308	-31	1	0.0003768	0.4	0	0	-6	19	23	10	3UTR

mmu-miR-214-3p	XM_036164017	Cyb5rl	ACAGCAGGCACAGACAGGCAGT#CTGCATTTAATGAGGACATGGCTAGGTGTTTGCTGTCTGCTGGCCTGACTGA#.((((((((.(((.((((((((#.))))))))))))))))).))).	862	914	0.92308	-30	1	1.54E-05	0.4	0	0	-6	19	22	16	CDS
mmu-miR-214-3p	XM_036164698	Sulf1	ACAGCAGGCACAGACAGGCAGT#TCTTTCTGTGCCTGCA#...(((((((((((.(((....#))).))))))))))).	3001	3017	1	-24	0	0.035048	0.5	0	0	-7	14	16	11	CDS
mmu-miR-214-3p	XM_036164706	Tyw1	ACAGCAGGCACAGACAGGCAGT#ACAGTCTGATAGGAGAGATCACTAGTAAAACCGTCTGTGCCTTCCTGG#.(((.(((((((((((.((....#.)))))))))))))..))).	509	557	1	-24	0	1.94E-05	0.5	0	0	-8	15	19	12	CDS
mmu-miR-214-3p	XM_036164706	Tyw1	ACAGCAGGCACAGACAGGCAGT#CCTCTCTGGACCTGCCTGTG#((((((((..((((.(((....#))).))))..))))).))).	455	475	0.92308	-21	1	0.0016521	0.5	0	0	-7	15	20	5	CDS
mmu-miR-214-3p	XM_036165466	Tmem214	ACAGCAGGCACAGACAGGCAGT#GCTGCTTCGATCCTCCGGTCTCTTGCCTGTTGG#.(((((((((.(((((((((((#))))))))..........))))..	1031	1064	0.84615	-26	1	5.99E-05	0.4	0	0	-7	20	33	9	CDS
mmu-miR-214-3p	XM_036165466	Tmem214	ACAGCAGGCACAGACAGGCAGT#GCGCCCTCTGTCAGCCTGCTGA#.(((((((((((((..(((.((#))))).)))))..)))))))).	2158	2180	0.91026	-25	1	1.02E-06	0.4	0	0	-5	18	22	8	3UTR
mmu-miR-214-3p	XM_036165466	Tmem214	ACAGCAGGCACAGACAGGCAGT#GCCAGCCTGACCCAGTGCCTGACTGTG#(((((((((((.(.(((((.((#))..))))).)...))))))).)))).	648	675	0.92308	-25	1	0.0002069	0.4	0	0	-8	19	27	7	CDS
mmu-miR-214-3p	XM_036166039	Creb5	ACAGCAGGCACAGACAGGCAGT#ACATGGATGTGTGTGCCTGAGTGTG#(((.((((((((.(((..((((#)).))..))).))))))))..))).	6251	6276	0.84615	-22	0	0.0486198	0.5	0	0	-8	18	25	8	3UTR
mmu-miR-214-3p	NM_001357430	Ildr2	ACAGCAGGCACAGACAGGCAGT#GCTGCCCTCACAGTTGCTGCTGCTATGTCCGCTGCCCATGCTGC#.((((((((((.((((((((((#.)))))).)))).).))))..))))).	856	900	0.84615	-26	1	0.0017727	0.4	3.9	3.17	-7	20	27	6	CDS
mmu-miR-214-3p	NM_001357430	Ildr2	ACAGCAGGCACAGACAGGCAGT#CTGCCCAGTTGTTTTTGTGCCTTGTCTGTG#(((((((((((((((((((((.#)))))....))))..))))))).).)))	8051	8081	0.92308	-25	1	0.0006165	0.6	0.8	0.15	-4	21	30	7	3UTR
mmu-miR-214-3p	NM_001357430	Ildr2	ACAGCAGGCACAGACAGGCAGT#ACTGCTGGTGTTGGAGTGGGTGTTTGTCGGCCTGGTGA#.((.((((((((((((((((((#)))))))..............))))))).))))).)).	767	805	0.84615	-24	0	0.0002789	0.5	3.3	3.18	-7	20	38	7	CDS

mmu-miR-214-3p	NM_001357431	Ildr2	ACAGCAGGCACAGACAGGCAGT#GCTGCTTGCTGATCTCTTGCCCAGTTTTGCTGTG#(((((((((((((.((((((((#))))))))))))......))))......)))))).	791	825	0.92308	-25	1	0.0001127	0.5	4	3.32	-5	21	34	11	CDS
mmu-miR-214-3p	NM_001357431	Ildr2	ACAGCAGGCACAGACAGGCAGT#CTGCCCAGTTGTTTTTGTGCCTTGTCTGTG#(((((((((((((((((((((.#)))))....))))..))))))).).)))	7784	7814	0.92308	-25	1	0.0006165	0.6	0.8	0.15	-4	21	30	7	3UTR
mmu-miR-214-3p	NM_001357431	Ildr2	ACAGCAGGCACAGACAGGCAGT#ACTGCCTAGCTGAGCCGAGCC#...((.(((.(((..(((((((#)))))))..))).)))..)).	3303	3324	1	-23	0	0.0039313	0.4	1.3	0.12	-11	15	21	7	3UTR
mmu-miR-214-3p	NM_001357431	Ildr2	ACAGCAGGCACAGACAGGCAGT#CTGTCTCTGTCTGTGACTCACAGCTGA#.((((((.(((((((((((((.#))))..))))))))).))....)))).	4892	4919	0.92308	-23	1	4.30E-05	0.5	0.3	0.31	-6	19	27	9	3UTR
mmu-miR-214-3p	NM_001357432	Ildr2	ACAGCAGGCACAGACAGGCAGT#GCTGCTTGCTGATCTCTTGCCCAGTTTTGCTGTG#(((((((((((((.((((((((#))))))))))))......))))......)))))).	791	825	1	-25	1	0.0001338	0.5	4	3.39	-5	21	34	11	CDS
mmu-miR-214-3p	NM_001357432	Ildr2	ACAGCAGGCACAGACAGGCAGT#GCTGCCCTCACAGTTGCTGCTGCTATGTCCGCTGCCCATGCTGC#.((((((((((.((((((((((#.)))))).)))).).))))..))))).	913	957	0.84615	-26	1	0.0017727	0.4	3.9	3.17	-7	20	27	6	CDS
mmu-miR-214-3p	NM_001357432	Ildr2	ACAGCAGGCACAGACAGGCAGT#CTGCCCAGTTGTTTTTGTGCCTTGTCTGTG#(((((((((((((((((((((.#)))))....))))..))))))).).)))	7411	7441	0.92308	-25	1	0.0006165	0.6	0.8	0.15	-4	21	30	7	3UTR
mmu-miR-214-3p	NM_001356456	Fam53c	ACAGCAGGCACAGACAGGCAGT#ACGCCTGCATCTGTGCTGCGTGTG#((((((.(((((((((((((.((#)))))))..)))))))))).))).	4175	4199	0.84615	-25	0	0.0001901	0.4	2.2	1.48	-5	20	24	10	3UTR
mmu-miR-214-3p	NM_001357456	Fam53c	ACAGCAGGCACAGACAGGCAGT#CTGCCTCTGTGGCTGTG#...((((.((((((..(((((.#))))))))))).)))).	4444	4461	0.92308	-24	0	4.11E-05	0.7	2.9	2.68	-10	15	17	11	3UTR
mmu-miR-214-3p	NM_001357456	Fam53c	ACAGCAGGCACAGACAGGCAGT#ACTGCCTTTTGGAAGCTGGCTGTA#((((((.(((.((((.(((((((#))))))))))))...))).)))))).	2781	2805	1	-24	0	3.06E-05	0.5	0.4	0.16	-8	19	24	11	3UTR
mmu-miR-214-3p	NM_001357475	Dclk1	ACAGCAGGCACAGACAGGCAGT#ATTGTCTCTCTGTGTCTGTCTGTC#(((((((((((((((.((((((((#)))))))).)))))))))).)))).	6265	6289	0.84615	-28	1	0.1847508	0.6	0.1	-0.1	-5	21	24	10	3UTR

mmu-miR-214-3p	NM_001357475	Dclk1	ACAGCAGGCACAGACAGGCAGT#GCTGCTTGCCCATTACCTGCTGC#.(((((((......((((((((#)))))))).......))))))).	3352	3375	0.92308	-23	1	0.0017245	0.4	2.4	0.39	-14	15	23	8	3UTR
mmu-miR-214-3p	NM_001357476	Dclk1	ACAGCAGGCACAGACAGGCAGT#ATTGTCTCTCTGTGTCTGTCTGTC#((((((((((((((((.(((((((#))))))).))))))))))).))))).	5346	5370	0.84615	-28	1	0.1847508	0.6	0.1	-0.1	-5	21	24	10	3UTR
mmu-miR-214-3p	NM_001357476	Dclk1	ACAGCAGGCACAGACAGGCAGT#GCTGCTTGCCCATTACCTGCTGC#.(((((((......((((((((#))))))))).......))))))).	2433	2456	0.92308	-23	1	0.0017245	0.4	2.4	0.39	-14	15	23	8	3UTR
mmu-miR-214-3p	NM_001357503	Rexo2	ACAGCAGGCACAGACAGGCAGT#CTGCCTGATAACTGACTCTGATCTTAACATTTTGGCTGA#.(((((((..(((((((((((.#))))))).........))))..)))..........)))).	249	288	0.84615	-18	1	0.0287017	0.5	4.5	3.52	-6	18	39	7	CDS
mmu-miR-214-3p	NM_001357505	Rexo2	ACAGCAGGCACAGACAGGCAGT#CTGCCTGATAACTGACTCTGATCTTAACATTTTGGCTGA#.(((((((..(((((((((((.#))))))).........))))..)))..........)))).	249	288	0.84615	-18	1	0.0287017	0.5	3.9	3.67	-6	18	39	7	CDS
mmu-miR-214-3p	NM_001357506	Zbtb7c	ACAGCAGGCACAGACAGGCAGT#CTGCTTGACCTGTGACCTTAGGAGGGTCAACCCTCTGTGCCTCAGAGTCACCCTGCTGTG#((((((((((((((((((((((.#)))))))).........................)))))))))............))))).	2749	2809	1	-27	1	0.0002188	0.4	0.2	-0	-4	21	60	9	3UTR
mmu-miR-214-3p	NM_001357506	Zbtb7c	ACAGCAGGCACAGACAGGCAGT#CTGTCTGTCTGTGGGAGACCTTTGG#.(((.(((((((((((((((.#))))))))))))))).....)))))).	4186	4211	1	-25	0	6.79E-05	0.6	1.6	1.39	-8	19	25	13	3UTR
mmu-miR-214-3p	NM_001357506	Zbtb7c	ACAGCAGGCACAGACAGGCAGT#CTGCCTGCCAGTGCCA#......(((((.(.(((((((.#))))))).).))))).	3710	3726	0.90385	-23	0	0.0154576	0.4	-0	0.22	-20	13	16	7	3UTR
mmu-miR-214-3p	NM_001357717	Pheta2	ACAGCAGGCACAGACAGGCAGT#TTGCTCTGTGTGTGTGTGTGTGTG#((((((.(((((.((((((((.#)))).)))).))))).))).))).	1344	1368	1	-24	0	0.035005	0.5	0.3	0.11	-7	19	24	5	3UTR
mmu-miR-214-3p	NM_001357717	Pheta2	ACAGCAGGCACAGACAGGCAGT#GCTGCCCTGTCCATGTATAGTGCTGG#.(((((...(((((((((((((#)))))).))))..)))....))))).	1675	1701	0.92308	-23	0	0.0001307	0.4	0.3	0.07	-9	18	26	6	3UTR
mmu-miR-214-3p	NM_001357722	Map3k4	ACAGCAGGCACAGACAGGCAGT#GCTGCTCCTGTCGCCTGCTGA#.(((((((((((...((((((#)))))).)))).))))))))).	4949	4970	1	-29	1	0.000279	0.4	0.9	1.34	-6	18	21	8	3UTR

mmu-miR-214-3p	NM_001357722	Map3k4	ACAGCAGGCACAGACAGGCAGT#GGTGCTCTGTCGGGTCCTGCTGA#.(((((((.((.((((((((.(#).))).)))))..))))))))).	1991	2014	0.84615	-26	1	0.0002391	0.4	4.5	3.85	-8	18	23	9	CDS
mmu-miR-214-3p	NM_001357722	Map3k4	ACAGCAGGCACAGACAGGCAGT#GCTGGTTGTCATGGAGTCTGCTC#..(((((((.(((((((.((((#)))).))))).))..))))))).	2978	3001	0.84615	-24	1	0.0001485	0.5	4.6	4.33	-6	18	23	7	CDS
mmu-miR-214-3p	NM_001357747	Slc45a4	ACAGCAGGCACAGACAGGCAGT#GCTGCCTCCCTGCTGCCTCCTGA#.(((.((((((((..(((((((#)))))))..))).))))).))).	6622	6645	0.84615	-27	0	0.0024074	0.3	0.5	1.95	-10	18	23	7	3UTR
mmu-miR-214-3p	NM_001357747	Slc45a4	ACAGCAGGCACAGACAGGCAGT#GCATGCCTGTTTGTTGCTGTC#((((((...(((((((((((((#)).))))))))))))))))).	5061	5082	0.84615	-25	0	0.0001058	0.5	3.9	3.54	-9	19	21	17	3UTR
mmu-miR-214-3p	NM_001357747	Slc45a4	ACAGCAGGCACAGACAGGCAGT#GCGCCTGCTGTGGCTGTC#...((((.(((((.(((((.((#)))))))))))).)))).	1939	1957	1	-25	0	2.36E-05	0.4	0.2	-0.3	-10	16	18	12	CDS
mmu-miR-214-3p	NM_001357796	Slc9a3r2	ACAGCAGGCACAGACAGGCAGT#CTGCCATCCTGCCTCGGGCCTGCTGA#.(((((((.(....((((((.#.))))))..))))))))).	1400	1426	1	-26	1	0.0008569	0.5	3.4	2.2	-11	15	19	9	3UTR
mmu-miR-214-3p	NM_001357796	Slc9a3r2	ACAGCAGGCACAGACAGGCAGT#GCCAGCTGTGCCTGGAGGAGCTGTC#(((((((((((((.(.(((...#))).))))))))))))......))))).	1640	1665	0.92308	-25	1	1.84E-06	0.4	1.1	2.25	-5	17	25	9	3UTR
mmu-miR-214-3p	NM_001357965	Wscd1	ACAGCAGGCACAGACAGGCAGT#ACTGCTTGTCTGACATCCCATAAATGTGTGTGCTGTG#((((((.(((((((((((((((#)))))))))))).............)))).))))))).	2776	2813	1	-31	0	0.0033626	0.6	0.4	-0.2	-5	21	37	11	3UTR
mmu-miR-214-3p	NM_001357965	Wscd1	ACAGCAGGCACAGACAGGCAGT#ACTGCCAGGATGCCTGTGCTGAGCGGTCTTATGTCTATGCTGG#.((((((((((((.((((((((#)))))))....)).))))))))................))))).	994	1037	0.84615	-25	1	0.0001596	0.4	2.4	2.4	-5	20	43	7	CDS
mmu-miR-214-3p	NM_001358081	Cdip1	ACAGCAGGCACAGACAGGCAGT#TGCCTGTGTGGACGCTGTATCCTGCTGC#.(((((((.((((((((((((..#)))))))).......)))).))))))).	1845	1873	1	-26	1	0.0008632	0.5	1.6	0.59	-5	18	28	7	3UTR
mmu-miR-214-3p	NM_001358081	Cdip1	ACAGCAGGCACAGACAGGCAGT#CTGCTGTCTTGCCTGGCTT#..((((((((.(((((.((((.#)))))))))))))))))).)).	1984	2003	0.86154	-23	1	0.0057751	0.4	0.7	0.79	-8	17	19	15	3UTR
mmu-miR-214-3p	NM_001358082	Cdip1	ACAGCAGGCACAGACAGGCAGT#TGCCTGTGTGGACGCTGTATCCTGCTGC#.(((((((.((((((((((((..#)))))))).......)))).))))))).	1926	1954	1	-26	1	0.0008632	0.5	1.4	1.06	-5	18	28	7	3UTR

mmu-miR-214-3p	NM_001358082	Cdip1	ACAGCAGGCACAGACAGGCAGT#CTGTCTACTTCTGTGTCTGTG#...(((((((((((.((((((.#))))))...))))))))))).	1640	1661	0.84615	-25	0	0.0214973	0.5	-1	-0.4	-7	17	21	11	3UTR
mmu-miR-214-3p	NM_001358082	Cdip1	ACAGCAGGCACAGACAGGCAGT#CTGCTGTCTTGCCTGGCTT#..((((((((.(((((.(((((.#)))))))))))))))).)).	2065	2084	0.92308	-23	1	0.0057751	0.4	0.3	0.3	-8	17	19	15	3UTR
mmu-miR-214-3p	NM_001358287	Katnip	ACAGCAGGCACAGACAGGCAGT#GCTGCTGGGAGCCAGGGATACCTGATTCCTCTCTGTGCCTCCCCTGTG#((((.(((((((((.((((((.#.)))...))).))))))))...))))).	5063	5111	0.92308	-25	0	0.0002576	0.4	0.1	-0.2	-9	19	28	9	3UTR
mmu-miR-214-3p	NM_001358692	Elfn2	ACAGCAGGCACAGACAGGCAGT#GCTGCCTGTTTGGCATGGTCATCGTGCTGG#.(((((.(..((((((((((((#))))))))))))..........).)))	1970	2000	0.84615	-27	0	5.38E-06	0.4	-0	-0.3	-8	18	30	12	CDS
mmu-miR-214-3p	NM_001358692	Elfn2	ACAGCAGGCACAGACAGGCAGT#CTGCACCCCACTTGTCTGGCTCCTGCTGTG#((((((((..((((((((....#.))))))))...)))))))).	4259	4289	0.92308	-27	1	0.0003331	0.3	1.9	1.12	-6	16	21	8	3UTR
mmu-miR-214-3p	NM_001358978	Oxr1	ACAGCAGGCACAGACAGGCAGT#CTGCCTGTTCCAAGGGCGGAGCAGAAGCAGGCCGTGGTCCTGCC#...(((((..(((((((((((.#))))))))))).......................))..))).	139	183	0.92308	-22	0	8.90E-06	0.4	0.1	0.42	-9	16	44	9	5UTR
mmu-miR-214-3p	NM_001358978	Oxr1	ACAGCAGGCACAGACAGGCAGT#GTCTGTTTGGCCATTTACTGCA#...((((((.(((((((((...#))))))))))))......))).	2876	2898	0.92308	-20	0	0.0177324	0.7	0.7	1.67	-8	15	22	12	3UTR
mmu-miR-214-3p	NM_001359035	Gabra1	ACAGCAGGCACAGACAGGCAGT#CTGCCTTTGTCCTAGCTGG#.(((((((.((((..((((((.#)))))))))))).))))).	74	93	0.92308	-22	1	1.40E-06	0.4	2.8	3.25	-6	17	19	13	5UTR
mmu-miR-214-3p	NM_001359035	Gabra1	ACAGCAGGCACAGACAGGCAGT#TGCCTAGGTTTGGGTCTGTA#...((((((.((((((((((..#)))))..))))).)))))).	3618	3638	0.92308	-21	0	0.0004177	0.6	4.1	4.49	-8	16	20	6	3UTR
mmu-miR-214-3p	NM_001359041	Gabra4	ACAGCAGGCACAGACAGGCAGT#CTGCACTTCCTGTGCCTGGCGG#.(..(((((((((..((((((.#)))).))..)))))))))..).	559	581	0.92308	-25	0	2.03E-06	0.4	-0	0.34	-10	16	22	9	CDS
mmu-miR-214-3p	NM_001359041	Gabra4	ACAGCAGGCACAGACAGGCAGT#TGCTCTGTCTGTCCGTCTCTGGTCTGCCTGTC#((((((((((((((((..#))).)))))))))........))))	732	764	0.87179	-25	1	0.0253159	0.5	0.5	0.14	-4	20	32	8	CDS

mmu-miR-214-3p	NM_001359041	Gabra4	ACAGCAGGCACAGACAGGCAGT#GCTGTCTGTTTTGCTTTTGTATTTTCGGCTCTTATTGAGTTTGCTGCTGTC#((((((((((.(((((((((((#))))))))))))))).........................))))	1546	1597	1	-25	1	0.0013845	0.6	0.1	0.64	-7	21	51	14	CDS
mmu-miR-214-3p	NM_001359050	Gabra4	ACAGCAGGCACAGACAGGCAGT#GCTGTGCTCCGGGGTCAGCCTCGCCCTCCTGCACTTCCTGTGCCTGGCGG#.(..((((((((((..((((((.#.)))).))..)))))))))..).	531	581	0.92308	-26	0	2.03E-06	0.4	0.3	0.46	-10	16	23	9	CDS
mmu-miR-214-3p	NM_001359077	Prdm15	ACAGCAGGCACAGACAGGCAGT#ACTGCGTGTGTGGTATGCTGC#.(((((.(((.(((.((((.(((((#))))).))).)))).))))).	545	566	1	-25	0	0.0001236	0.5	0.4	-0.2	-8	17	21	5	CDS
mmu-miR-214-3p	NM_001359199	Ubl3	ACAGCAGGCACAGACAGGCAGT#CTGCACGTGTCTGTGGCGCCCAGTTCTCCTGTGTGTA#(((((((((((((((((((.#)))).).)))))))).............))))).))).	1371	1408	0.84615	-23	1	6.25E-05	0.4	0.9	1.31	-4	21	37	8	3UTR
mmu-miR-214-3p	NM_001359232	Arhgef12	ACAGCAGGCACAGACAGGCAGT#GCCGCCTGCTTGAGCTTGCTCTGTGCCTGCTT#..((((((((((((((((...#.))))).)))))))))))).	9758	9790	1	-31	1	0.0021418	0.4	4.2	4.23	-5	17	20	12	3UTR
mmu-miR-214-3p	NM_001359232	Arhgef12	ACAGCAGGCACAGACAGGCAGT#CTGTCTGTCTGGTGCCAGGACTGG#.((((.((((((((((((((.#))))))))))).))))..).))).	6383	6407	0.92308	-29	0	4.82E-07	0.4	1.7	1.49	-6	19	24	11	3UTR
mmu-miR-214-3p	NM_001359330	Clasp1	ACAGCAGGCACAGACAGGCAGT#CCTGTCTGGCCCAGGTGCTGC#.((((((((.((((((((....#)))))))))))....))))).	767	788	1	-26	1	3.77E-06	0.4	0	0	-5	16	21	11	CDS
mmu-miR-214-3p	NM_001359334	Clasp1	ACAGCAGGCACAGACAGGCAGT#CCTGTCTGGCCCAGGTGCTGC#.((((((((.((((((((....#)))))))))))....))))).	767	788	1	-26	1	3.77E-06	0.4	2.6	2.95	-5	16	21	11	CDS
mmu-miR-214-3p	NM_001359334	Clasp1	ACAGCAGGCACAGACAGGCAGT#TTGCACGTGTCTGCACCACCTGCTGA#.((((((..(((((((((((.#)))).).))))))).....))))))).	5525	5551	0.84615	-23	1	0.0005851	0.5	0.6	1.25	-6	18	26	7	3UTR
mmu-miR-214-3p	NM_001359345	Clasp1	ACAGCAGGCACAGACAGGCAGT#CCTGTCTGGCCCAGGTGCTGC#.((((((((.((((((((....#)))))))))))....))))).	767	788	1	-26	1	3.77E-06	0.4	2.6	2.95	-5	16	21	11	CDS
mmu-miR-214-3p	NM_001359345	Clasp1	ACAGCAGGCACAGACAGGCAGT#TTGCACGTGTCTGCACCACCTGCTGA#.((((((..(((((((((((.#)))).).))))))).....))))))).	5378	5404	0.84615	-23	1	0.0005851	0.5	0.6	1.25	-6	18	26	7	3UTR

mmu-miR-214-3p	NM_001359346	Clasp1	ACAGCAGGCACAGACAGGCAGT#CCTGTCTGGCCCAGGTGCTGC#.((((((((.((((((((....#)))))))))))....))))).	767	788	1	-26	1	3.77E-06	0.4	2.6	2.95	-5	16	21	11	CDS
mmu-miR-214-3p	NM_001359346	Clasp1	ACAGCAGGCACAGACAGGCAGT#TTGCACGTGTCTGCACCACCTGCTGA#.(((((((..(((((((((((.#)))).).))))))).....))))))).	5354	5380	0.84615	-23	1	0.0005851	0.5	0.6	1.25	-6	18	26	7	3UTR
mmu-miR-214-3p	NM_001359505	Mtus2	ACAGCAGGCACAGACAGGCAGT#CTGCCTTCAGTGCCTGG#....(((((((.((.((((((.#)))))))).))))))).	222	239	1	-25	0	0.0026389	0.5	2.9	2.6	-14	15	17	8	CDS
mmu-miR-214-3p	NM_001359505	Mtus2	ACAGCAGGCACAGACAGGCAGT#CTGCCTGTCTTTGAGCATGCC#...(((.((..(((((((((((.#)))))))))))....)).))).	2737	2758	0.92308	-23	0	2.54E-05	0.5	0.1	0.19	-12	15	21	10	3UTR
mmu-miR-214-3p	NM_001359505	Mtus2	ACAGCAGGCACAGACAGGCAGT#CCTGCTGGCCTGCA#...((((((.(((.((((....#))))))))))))).	3580	3594	0.92308	-23	0	9.73E-07	0.4	0.3	-0.3	-9	13	14	13	3UTR
mmu-miR-214-3p	NM_001359511	Mtus2	ACAGCAGGCACAGACAGGCAGT#CTGCCTTCAGTGCCTGG#....(((((((.((.((((((.#)))))))).))))))).	222	239	1	-25	0	0.0026389	0.5	2.9	2.6	-14	15	17	8	CDS
mmu-miR-214-3p	NM_001359511	Mtus2	ACAGCAGGCACAGACAGGCAGT#CTGCCTGTCTTTGAGCATGCC#...(((.((..(((((((((((.#)))))))))))....)).))).	2564	2585	0.92308	-23	0	2.54E-05	0.5	0.1	0.19	-12	15	21	10	3UTR
mmu-miR-214-3p	NM_001359511	Mtus2	ACAGCAGGCACAGACAGGCAGT#CCTGCTGGCCTGCA#...((((((.(((.((((....#))))))))))))).	3407	3421	0.92308	-23	0	9.73E-07	0.4	0.3	-0.3	-9	13	14	13	3UTR
mmu-miR-214-3p	NM_001359563	Cnot2	ACAGCAGGCACAGACAGGCAGT#TTGGAAAATGTTTGCCCTGCTGTA#((((((((..((((((......#.)))))).)))))))).	2313	2337	1	-20	1	0.0123811	0.6	3.7	4.38	-6	14	17	8	3UTR
mmu-miR-214-3p	NM_001359752	Kcnc2	ACAGCAGGCACAGACAGGCAGT#GCCATGCCTGTGCCTGTC#...((((((((((.(((((...#))).)).)))))))))).	1473	1491	0.92308	-26	0	0.0003931	0.5	4.1	4.7	-7	15	18	10	CDS
mmu-miR-214-3p	NM_001359752	Kcnc2	ACAGCAGGCACAGACAGGCAGT#GCTGTCTGGTGCTGCTGTT#((((((.((((...((((((((#)))))))))))))))))).	3844	3863	1	-25	0	4.48E-05	0.6	2.1	1.97	-11	18	19	18	3UTR
mmu-miR-214-3p	NM_001360121	Treml2	ACAGCAGGCACAGACAGGCAGT#GCGAGACCCTGTCTGTGCAGTGCTC#..((((.(((((((((((....#.)))))))))))..)))).	604	629	1	-26	0	0.0026474	0.4	1.7	0.6	-7	15	19	11	CDS

mmu-miR-214-3p	NM_001360121	Treml2	ACAGCAGGCACAGACAGGCAGT#GCTGCTGCTGCTGTGGCTACAGGGCTGTG#(((((((.(((((.((((((((#)))))..)))))))).)).....))))).	524	553	0.92308	-25	1	8.69E-05	0.4	1.9	0.35	-7	20	29	8	CDS
mmu-miR-214-3p	NM_001360121	Treml2	ACAGCAGGCACAGACAGGCAGT#GCTGACGAAGGCCATGTGTCTTCTGATATTCCCTGACTGTGACTGCTGG#.((((((.(((((.((((.(((#.))).)))).))))).)))))).	1573	1622	0.92308	-25	1	0.0089237	0.5	0.4	-0.2	-7	18	23	6	3UTR
mmu-miR-214-3p	NM_001360306	Klhl18	ACAGCAGGCACAGACAGGCAGT#CTGCCCCTCAGTTTGCCTGCTGA#.((((((((((..((..(((((.#)))))..))....))))))))).	3817	3840	0.92308	-26	1	2.67E-06	0.4	2.8	2.96	-11	16	23	9	3UTR
mmu-miR-214-3p	NM_001360306	Klhl18	ACAGCAGGCACAGACAGGCAGT#ATTGCCTGGGTGTGCGCCAGTTTGCCGAGACGATGATGTGTGCTGTG#((((((((((((..((((((((#))))))))..))))).)......................)))))).	515	562	0.92308	-26	1	0.0111414	0.5	0	-0	-6	20	47	8	CDS
mmu-miR-214-3p	NM_001360306	Klhl18	ACAGCAGGCACAGACAGGCAGT#TTGCCCTCTGCTGCCTGCCTGG#.(((((((((((((..(((((.#))))).)))).))))))).)).	3537	3559	0.84615	-26	1	0.0011452	0.4	4.6	3.76	-5	18	22	7	3UTR
mmu-miR-214-3p	NM_001360665	Dlgap1	ACAGCAGGCACAGACAGGCAGT#CTGCTCTGGGACTTGCTGAATGTCACCTGAAGGCCCCTGTGTCTGCTT#..(((((((((((...(((...#.))).))))))))))).	1000	1048	1	-24	1	0.0007864	0.5	0.3	0.53	-7	14	17	11	5UTR
mmu-miR-214-3p	NM_001360665	Dlgap1	ACAGCAGGCACAGACAGGCAGT#CTGCATCTGTGCCCTCCCTCGCAGCTGTA#(((((.((((((((...((((.#)))).))))))))..........))))).	4680	4709	1	-23	0	0.0030793	0.4	0.3	1	-7	17	29	8	3UTR
mmu-miR-214-3p	NM_001360779	Crygf	ACAGCAGGCACAGACAGGCAGT#GCTGCAACTCTGTGCGAGTGGACAGTGGCTGCTGG#.(((((((((((((...(((((#)))))...)))))))............)))))).	130	165	0.84615	-26	1	3.13E-06	0.4	2.7	2.07	-5	18	35	7	CDS
mmu-miR-214-3p	NM_001360811	Sbk1	ACAGCAGGCACAGACAGGCAGT#TCTGTGCCTGCTGG#.(((((((((((((........#)))))))))))))).	187	201	0.92308	-27	1	1.68E-05	0.5	0	0	-5	13	14	13	5UTR
mmu-miR-214-3p	NM_001360827	Prox1	ACAGCAGGCACAGACAGGCAGT#ACTGCCTTGTGTGCCTT#.....(((((((.(.((((((((#)))))))).))))))).	7726	7743	1	-24	0	0.0249413	0.5	0.1	0.05	-16	15	17	8	3UTR
mmu-miR-214-3p	NM_001360827	Prox1	ACAGCAGGCACAGACAGGCAGT#GCTGTCTGTCTTTTCCCTCTGG#.(((.(((...((((((((((#)))))))))))....)))))).	3646	3668	1	-23	0	0.0005861	0.5	3	2.14	-13	17	22	11	3UTR

mmu-miR-214-3p	NM_001360827	Prox1	ACAGCAGGCACAGACAGGCAGT#GCTGCGCCCTGGCCTCGCTGA#.((((((((.((((....(((((#)))))...)))))))).)))).	263	284	0.84615	-23	1	2.39E-06	0.3	1.6	2.56	-7	16	21	7	5UTR
mmu-miR-214-3p	NM_001360827	Prox1	ACAGCAGGCACAGACAGGCAGT#GTTTGCTTTGTGCCTCTGA#.(((.((((((((((((((...#))))).)))))))))))).	5996	6015	1	-23	0	0.0218659	0.7	3.9	2.91	-8	17	19	12	3UTR
mmu-miR-214-3p	NM_001360847	Rnf2	ACAGCAGGCACAGACAGGCAGT#GCTAGCCTGGAACTTGCTGA#.(((((((......((((((((#))).)))))...))))))).	1828	1848	0.92308	-20	1	0.0005177	0.5	2.3	1.55	-14	15	20	7	3UTR
mmu-miR-214-3p	NM_001360897	Ipo11	ACAGCAGGCACAGACAGGCAGT#GCCTGCCATCCATTTATGAGCCTGCTGA#.((((((((.((..(((((...#)))))..........)).)))))))).	3211	3239	0.84615	-24	1	0.0078296	0.6	3.8	3.92	-7	15	28	8	3UTR
mmu-miR-214-3p	NM_001360897	Ipo11	ACAGCAGGCACAGACAGGCAGT#ACTGTGACAGTCGTGCTTGGCTGTG#(((((((((((.(((..(((((#)))))....)))))))))).)))).	3919	3944	0.90385	-23	1	3.36E-06	0.5	4.2	3.54	-8	19	25	10	3UTR
mmu-miR-214-3p	NM_001360903	Galnt18	ACAGCAGGCACAGACAGGCAGT#GCTGCAATGCCGCCTGCTGTC#(((((((((.(((.....(((((#)))))..))..))))))))).	2301	2322	0.84615	-23	1	0.0008328	0.4	1	0.61	-8	16	21	9	3UTR
mmu-miR-214-3p	NM_001360926	Gpa33	ACAGCAGGCACAGACAGGCAGT#GCCCAGTCTGGCCCAGGTGAGCTGG#.((((.((((.(((((.(((...#)))..))))))))).......)))).	193	218	0.84615	-21	0	2.81E-07	0.4	0.1	0.2	-7	15	25	8	5UTR
mmu-miR-214-3p	NM_001361075	Ube4a	ACAGCAGGCACAGACAGGCAGT#ACTGCCCTTGGTGTGTGTTTGTTT#..(((((((((((.(((.((((((#))))))....)).)))))))))).	2307	2331	1	-23	1	0.0028807	0.6	3.8	3.19	-6	18	24	10	3UTR
mmu-miR-214-3p	NM_001361075	Ube4a	ACAGCAGGCACAGACAGGCAGT#CCAGTCTGCTGCCTCGAGGCTGTT#(((((((((((((((.((....#)).))))).))))))....))))).	2391	2415	0.84615	-22	1	5.41E-05	0.5	0.2	2.35	-4	17	24	5	3UTR
mmu-miR-214-3p	NM_001361147	Pfn4	ACAGCAGGCACAGACAGGCAGT#TGCCAGTCTCATGCCTCCTT#..((.(((((.((((.((((..#)))).))))..))))).)).	1314	1334	0.84615	-19	0	0.0390663	0.5	0.2	0.13	-12	15	20	5	3UTR
mmu-miR-214-3p	NM_001361252	Smc6	ACAGCAGGCACAGACAGGCAGT#GCGCCTGTCTGCAGGGCTCCCGCCTGCA#...((((((.(((((((((.((#)))))))))))..........)))))).	252	280	0.89744	-26	0	1.10E-10	0.3	0.6	0.19	-8	17	28	11	5UTR
mmu-miR-214-3p	NM_001361352	Stx8	ACAGCAGGCACAGACAGGCAGT#ACTGTTGCTATAATTTTACATTCTGTCTTAAGTGTCTGCTC#..((((((((((((((((((((#)))))).................))))))...))))))))).	370	411	1	-24	1	0.0009678	0.6	0.8	0.81	-5	20	41	9	CDS

mmu-miR-214-3p	NM_001361623	Limk2	ACAGCAGGCACAGACAGGCAGT#TGCCTGTGTGTGTCTAGCC#...(((((((((.((((((((..#))))))).))))))).)).	84	103	0.92308	-27	0	0.0020599	0.4	-1	-0.1	-8	16	19	7	5UTR
mmu-miR-214-3p	NM_001361623	Limk2	ACAGCAGGCACAGACAGGCAGT#GCTGCCCTACTCTGTCACGCTCATCTCCATGCCTGCTA#..((((((((((((..((((((#))))))....)))))..............))))))).	754	792	0.84615	-25	1	0.2652571	0.4	-0	-0.5	-6	18	38	7	CDS
mmu-miR-214-3p	NM_001361623	Limk2	ACAGCAGGCACAGACAGGCAGT#GCTGAGCCTGTTTATCTGCTGTG#((((((((...(((((((((((#)))..)))))))).)))))))).	352	375	1	-24	1	0.002662	0.5	0.1	-0.1	-9	19	23	8	5UTR
mmu-miR-214-3p	NM_001361641	Cenpo	ACAGCAGGCACAGACAGGCAGT#CTGCCTGCTGGTCATGCCCTCTCATTACAACGCCTGCG#...((((((.(((.((((((((.#)))))))))).....................)))))).	1740	1778	0.84615	-25	0	0.0451244	0.4	1.5	0.14	-9	16	38	10	3UTR
mmu-miR-214-3p	NM_001361641	Cenpo	ACAGCAGGCACAGACAGGCAGT#CTGTGCTGTGCAGTGTGTGTTGTT#((((((.((((.((((((((((.#)))).)))).).)))).))))))).	1685	1709	0.84615	-22	0	0.0103751	0.5	0.5	0.29	-9	19	24	6	3UTR
mmu-miR-214-3p	NM_001361909	Tmem161b	ACAGCAGGCACAGACAGGCAGT#ACTGTCTGTCCCACGACCTGTTC#..((((((((...((((((((((#))))))))))....).))))))).	105	128	0.84615	-22	1	0.0014131	0.5	0.3	1.22	-12	17	23	10	5UTR
mmu-miR-214-3p	NM_001361909	Tmem161b	ACAGCAGGCACAGACAGGCAGT#ACTGTTAGTTTTGGGCCTTGCTGTA#(((((((((.(((((.((((((#)))))).)))).).)))).))))).	2137	2162	0.92308	-22	1	0.0281235	0.7	0.3	0.8	-5	20	25	6	3UTR
mmu-miR-214-3p	NM_001361987	Fbln5	ACAGCAGGCACAGACAGGCAGT#GCTGTGAGACAAGCAGAAGTCTCCGAACACTTCTGTCTGCGTTTGCTC#..((((((((.((((((((.(((#.))))))))))).))))))).	125	173	1	-24	1	0.0005438	0.5	0.3	0.12	-6	18	21	11	5UTR
mmu-miR-214-3p	NM_001361987	Fbln5	ACAGCAGGCACAGACAGGCAGT#ACCGCTGTATGTGTCCTGCTGA#.((((((((((.((((.(((.((#)).))))).)))).))))))).	1223	1245	1	-23	1	0.0059948	0.5	0.2	0.02	-5	18	22	7	CDS
mmu-miR-214-3p	NM_001362210	Kbtbd2	ACAGCAGGCACAGACAGGCAGT#GCCTGTATAGGGGACTTGAGGGAGTGCCACGTGCTGA#.((((((((((..((((((...#))))))..................)))))...))))).	2529	2566	0.84615	-23	1	0.001667	0.5	4.7	4.37	-8	16	37	6	CDS
mmu-miR-214-3p	NM_001362905	Slc25a25	ACAGCAGGCACAGACAGGCAGT#GCTGCCCATGCTCAATGAGTGGCCTGCTGG#.((((((((.((..((((((((#))))))).........))..))))))))	3334	3364	0.84615	-27	1	5.25E-09	0.4	0.7	0.72	-7	18	30	10	3UTR

mmu-miR-214-3p	NM_001362905	Slc25a25	ACAGCAGGCACAGACAGGCAGT#GCTGCCTGCATGGGCCTAACGTCCTCTGAGCTGC#.((((((((.((..((((((((#))))))))..)).))))............)))).	2543	2577	0.92308	-26	1	0.0011327	0.4	2.8	4.32	-7	18	34	8	3UTR
mmu-miR-214-3p	NM_001362905	Slc25a25	ACAGCAGGCACAGACAGGCAGT#GCTGCCTGACAGCGTGGTGCATGGCTTTGCTGA#.((((((((((...((((((((#))))))))........)))).....)))))).	2474	2507	0.92308	-22	1	2.47E-05	0.4	4.2	3.55	-9	18	33	8	3UTR
mmu-miR-214-3p	NM_001363158	Afmid	ACAGCAGGCACAGACAGGCAGT#ACAGCCTGTGGCTCCAGCCTGCCCTGTGCTGGTGCTTGTG#...(((((((((((((((((.#.))))).))).))).))))))).	782	822	0.92308	-24	0	3.07E-07	0.4	0.1	1.55	-7	18	23	7	CDS
mmu-miR-214-3p	NM_001363159	Afmid	ACAGCAGGCACAGACAGGCAGT#ACAGCCTGTGGCTCCAGCCTGCCCTGTGCTGGTGCTTGTG#...(((((((((((((((((.#.))))).))).))).))))))).	720	760	0.92308	-24	0	3.07E-07	0.4	0.1	1.55	-7	18	23	7	CDS
mmu-miR-214-3p	NM_001363195	Msi2	ACAGCAGGCACAGACAGGCAGT#GCCTGGATGTGGGGCTGG#.((((...((((..(((((...#)))))..))))..)))).	4637	4655	0.84615	-24	0	2.07E-07	0.4	1.8	2.35	-12	13	18	5	3UTR
mmu-miR-214-3p	NM_001363195	Msi2	ACAGCAGGCACAGACAGGCAGT#GCCTCTCTGTGCACAGCTT#..(((.((((((((.((((...#)))).))))))).).))).	2264	2283	0.80769	-23	0	0.0060659	0.5	3.1	3.58	-8	15	19	7	3UTR
mmu-miR-214-3p	NM_001363348	Stim2	ACAGCAGGCACAGACAGGCAGT#TTGCCTTCCCCAGATCCAGACATCCTGTCTGTGTCAAGTTGCCCTGCTC#..((((((((((((((((..((#.))..))))))))))).........)))))).	2044	2093	1	-26	1	0.0003609	0.5	3.3	1.77	-5	18	31	10	CDS
mmu-miR-214-3p	NM_001363348	Stim2	ACAGCAGGCACAGACAGGCAGT#GCCTGTCGGCTGCGGGAGCTGA#.((((..((((.(((((((...#))))))).).)))....)))).	1261	1283	0.84615	-23	0	4.23E-07	0.3	2.5	3.66	-11	15	22	7	CDS
mmu-miR-214-3p	NM_001363488	Znrf1	ACAGCAGGCACAGACAGGCAGT#ACTGACCCTGCTGGGCCTGCTT#..(((((((.(((.((((((((#))))..))))))).))))))).	1180	1202	1	-26	1	7.00E-05	0.5	3.8	3.29	-7	18	22	7	CDS
mmu-miR-214-3p	NM_001363488	Znrf1	ACAGCAGGCACAGACAGGCAGT#GCTGTCATGTCCTCTGAATTTGGACCCAGATTGCCAGTGCCTGGTGTG#(((.((((((((.((((((((((#))))))).))))......................))))))).))).	3929	3977	0.92308	-26	0	4.09E-05	0.4	-0	0.25	-10	20	48	7	3UTR

mmu-miR-214-3p	NM_001363489	Znrf1	ACAGCAGGCACAGACAGGCAGT#ACTGACCCTGCTGGGCCTGCTT#..(((((((.(((.((((((((#))))..))))))).))))))).	379	401	1	-26	1	7.00E-05	0.5	3.8	3.29	-7	18	22	7	CDS
mmu-miR-214-3p	NM_001363489	Znrf1	ACAGCAGGCACAGACAGGCAGT#GCTGTCATGTCCTCTGAATTTGGACCCAGATTGCCAGTGCCTGGTGTG#(((.((((((((.((((((((((#)))))).))))........................))))))).))).	3128	3176	0.92308	-26	0	4.09E-05	0.4	-0	0.25	-10	20	48	7	3UTR
mmu-miR-214-3p	NM_001363498	Hook2	ACAGCAGGCACAGACAGGCAGT#GCTGCCTGCTGGAGTCTTCCAGTTTACATTTACCCAGCTGG#.((((((((.(((.((((((((#))))))))))..))))..................)))).	2181	2222	0.92308	-25	1	0.0001077	0.5	-0	0.16	-6	19	41	11	3UTR
mmu-miR-214-3p	NM_001363498	Hook2	ACAGCAGGCACAGACAGGCAGT#GCTGCTTATCAGTGCCTGGTA#..(.((((((((.((.(((((((#))))))).)).))))))).).	1989	2010	0.84615	-25	0	3.60E-05	0.4	2.3	2.5	-12	17	21	7	CDS
mmu-miR-214-3p	NM_001363981	Ercc2	ACAGCAGGCACAGACAGGCAGT#ATCGCCTGCTGTGCCTCCCTGG#.(((.((((((((.(((((.((#)).)))))))))))))..))).	3064	3086	0.92308	-26	0	0.0001678	0.4	0.6	-0.1	-9	18	22	13	3UTR
mmu-miR-214-3p	NM_001364364	Traf3	ACAGCAGGCACAGACAGGCAGT#GCTGCCATCTGTCCTGGGTGTGCCTTCGCCCCAGTGCCTGCTGG#.((((((((((.((((((((((#))))))...))))...................)))))))))).	3828	3872	0.84615	-30	1	4.61E-06	0.3	1.5	1.46	-7	20	44	10	3UTR
mmu-miR-214-3p	NM_001364913	Pim1	ACAGCAGGCACAGACAGGCAGT#GCTGCTTGCTGGAGTGGGTCTAGGGTCTGCTGA#.((((((((.(((.((((((((#))))))))))))............)))))))).	1537	1570	0.92308	-25	1	3.75E-05	0.5	0.2	0.81	-6	19	33	11	3UTR
mmu-miR-214-3p	NM_001364913	Pim1	ACAGCAGGCACAGACAGGCAGT#AGTGCCTTCTGTGGGGACTCCGCCTGTGCTGG#.(((((((((((((.(((((.(#).))))))))))))........	796	828	0.84615	-22	1	0.0001831	0.5	2	1.52	-5	19	32	10	3UTR
mmu-miR-214-3p	NM_001364926	Med20	ACAGCAGGCACAGACAGGCAGT#ACTGGCTGATGGCCTGCA#...((((((.((..(((.((((#)))).))).)))))))).	1851	1869	1	-24	0	7.39E-05	0.5	0	0.07	-10	15	18	8	3UTR
mmu-miR-214-3p	NM_001364926	Med20	ACAGCAGGCACAGACAGGCAGT#GGTGCCTGAGATGCAGAACAGACCTTGTGCTTGCTA#..((((((((((((.((((((.(#).))))))).............))))))))))).	945	981	0.84615	-23	1	2.87E-05	0.5	0	-0.1	-6	18	36	11	3UTR
mmu-miR-214-3p	NM_001365070	Actr1a	ACAGCAGGCACAGACAGGCAGT#ACTGCCTGTGTGTGTGAATGA#.((....((((((.((((((((((#))))))))).)))))...)).	1211	1232	0.97436	-26	0	0.059774	0.5	0.4	0.58	-16	16	21	9	3UTR

mmu-miR-214-3p	NM_001365070	Actr1a	ACAGCAGGCACAGACAGGCAGT#CTGTGCTGTCCATGCCTCTGG#.(((.(((((..((((((((((.#)))).)))))..)))))))).	1552	1573	0.84615	-23	0	0.0003435	0.4	3.9	3.1	-14	17	21	8	3UTR
mmu-miR-214-3p	NM_001368147	Psmg2	ACAGCAGGCACAGACAGGCAGT#CTTTGTCAGCCCTATCAAGGCCTGTCTGCTAGCTGAGACTTTCGTTGCCTGCG#...((((((((((((((((...#.))))))))))...............))))))).	303	356	0.92308	-29	0	0.0003302	0.5	-0	-0.1	-7	16	35	9	5UTR
mmu-miR-214-3p	NM_001368147	Psmg2	ACAGCAGGCACAGACAGGCAGT#CTGCTGAGATCACAAAAGAGGCTGTTGGGTGTGTGCTGTA#((((((.((((.(((((.(...#.).)))))..)))).)))))).	1154	1194	0.92308	-25	0	0.0006568	0.5	0	0.4	-9	16	22	6	3UTR
mmu-miR-214-3p	NM_001368179	Rprd2	ACAGCAGGCACAGACAGGCAGT#GCCTGTGGAGAAACCTGCTGTC#((((((((.....((((((...#)))))).......)))))))).	1296	1318	1	-24	1	0.0001424	0.5	0.6	1.14	-14	14	22	8	CDS
mmu-miR-214-3p	NM_001368179	Rprd2	ACAGCAGGCACAGACAGGCAGT#GCTTGTCGTCCTGGTGTA#(((.((((.((.((((((((...#)))))))))))))).)))).	329	347	1	-23	0	5.83E-05	0.6	0.5	0.59	-11	16	18	13	CDS
mmu-miR-214-3p	NM_001368179	Rprd2	ACAGCAGGCACAGACAGGCAGT#GCTTGTCTCAGTCTACTGCTGG#.((((((..((((((((((...#))))))))..))...)))))).	2963	2985	1	-23	1	1.87E-07	0.5	-0	0.02	-7	16	22	8	CDS
mmu-miR-214-3p	NM_001368179	Rprd2	ACAGCAGGCACAGACAGGCAGT#ACTGCGAACCCTGTGCCAGCC#...((.(((((((....(((((#))))).....))))))).)).	1765	1786	1	-22	0	0.008102	0.5	0.1	0.31	-10	14	21	7	CDS
mmu-miR-214-3p	NM_001368351	Atxn1l	ACAGCAGGCACAGACAGGCAGT#ATTGTCTGTCTATGTTGTGCCTTAGTGCCTGCA#...((((((((((((((((((((#)))))))))))............	3304	3337	1	-29	0	0.0002849	0.4	2.1	1.64	-7	19	33	11	3UTR
mmu-miR-214-3p	NM_001368351	Atxn1l	ACAGCAGGCACAGACAGGCAGT#ACTGCTGTTGTCTGCTCATTGTCCTGCCTGTTGC#.(((((((((((((((((((((#))))))..))))))..........))))))))).	5331	5365	1	-28	1	0.0541656	0.5	1.4	1.08	-4	21	34	9	3UTR
mmu-miR-214-3p	NM_001368351	Atxn1l	ACAGCAGGCACAGACAGGCAGT#TTGTTTGCTCTGTGCTGCTGTG#((((((.(((((((((((((((.#))))))).))))))))))))).	5307	5329	0.92308	-28	0	0.000273	0.5	1.2	1.23	-5	20	22	13	3UTR
mmu-miR-214-3p	NM_001368638	Nras	ACAGCAGGCACAGACAGGCAGT#CTGCTTACAGACTTTTGCCTGATGCCTGCTGTT#((((((((((....(((((((.#.))))))).)))))))))).	2022	2055	1	-28	1	0.0023665	0.6	0.8	0.36	-12	17	20	10	3UTR

mmu-miR-214-3p	NM_001368638	Nras	ACAGCAGGCACAGACAGGCAGT#TGTGTGTGTGTGTGTGCA#...(((.(((((.(((.(((..#))).))).))))).))).	3035	3053	0.84615	-23	0	0.0034139	0.5	-0	0.14	-9	14	18	5	3UTR
mmu-miR-214-3p	NM_001368695	Ing4	ACAGCAGGCACAGACAGGCAGT#GCTATCCATGCTAGCCTGCTGTGTTTATTGTA#((((.((((((((.((((((((#.))).))))))))))))).))))	1345	1377	0.92308	-24	0	0.0056677	0.6	0.3	0.39	-9	20	24	13	3UTR
mmu-miR-214-3p	NM_001368736	Ppp1r12a	ACAGCAGGCACAGACAGGCAGT#AGTGCTGTCTGAAACTGTTGTG#(((((((...((((((.(((.(#).)))))))))...))))))).	4512	4534	1	-22	1	0.0037834	0.6	4	2.57	-9	17	22	9	3UTR
mmu-miR-214-3p	NM_001368736	Ppp1r12a	ACAGCAGGCACAGACAGGCAGT#CTGTGAAGTCTGGATGCCTGA#....(((((((((((..((((.#))))...))))))..)))))).	5256	5277	0.87179	-20	0	0.0141159	0.6	3.1	2.62	-10	15	21	6	3UTR
mmu-miR-214-3p	NM_001368761	Vgll3	ACAGCAGGCACAGACAGGCAGT#GTCTGTGATGCGCTGTGTCTGTG#...(((((((((((((((...#))))))).......)))))))))).	1805	1828	0.92308	-22	0	6.03E-05	0.5	1.3	1.84	-7	16	23	10	3UTR
mmu-miR-214-3p	NM_001368844	D430041D05Rik	ACAGCAGGCACAGACAGGCAGT#GCTGGTGTATTTGCCTGCTGTG#((((((((((...(((..((((#)))).)))...)))))))))).	1818	1840	1	-25	1	7.00E-05	0.5	0.2	-0	-11	17	22	10	CDS
mmu-miR-214-3p	NM_001368844	D430041D05Rik	ACAGCAGGCACAGACAGGCAGT#GCTGCTCTGTCTTCAGTGACCTCTGC#.(((.(((((((((((((((((#))))).))))))...))).)))))).	5063	5089	0.92308	-25	0	0.0044315	0.5	0.2	0.53	-8	20	26	6	3UTR
mmu-miR-214-3p	NM_001368858	Grtp1	ACAGCAGGCACAGACAGGCAGT#CTGCCTGTTTGTGGACATCCTACCTGTG#...(((((((((((((((((.#)))))))))))))).........))))).	870	898	1	-27	0	0.0014729	0.4	2.9	2.01	-7	18	28	13	CDS
mmu-miR-214-3p	NM_001368871	Vwde	ACAGCAGGCACAGACAGGCAGT#ACTGCTTGAACGGTGGATCATGTGTGCCTGA#....((((((((.((((((((#))))))))...........).))))	3649	3680	0.92308	-24	0	0.0029412	0.5	2.7	1.4	-11	17	31	8	CDS
mmu-miR-214-3p	NM_001368871	Vwde	ACAGCAGGCACAGACAGGCAGT#GCTGTCTGTCACCCTCCCTGTAAAAATGGTGGCCTGTG#...((((((.((((((((((((#))))))))))).................)))))))).	5031	5069	1	-24	0	0.0001439	0.5	2.5	2.68	-8	18	38	10	CDS
mmu-miR-214-3p	NM_001368871	Vwde	ACAGCAGGCACAGACAGGCAGT#ACATGCTTGTCTGGCAATCTCTGCA#...((((((.((((((((((((#)).))))))))))))).....)))).	4776	4801	1	-23	0	0.0012398	0.5	0.8	1.24	-8	18	25	12	CDS
mmu-miR-214-3p	NM_001369183	Ralgapb	ACAGCAGGCACAGACAGGCAGT#GCTGCCGTCCGGTGCCATTTGG#.(((..((((((.(((.((((((#)))))))))..)))))..))).	334	356	1	-24	0	0.0001476	0.5	0.5	1.6	-13	17	22	9	5UTR

mmu-miR-214-3p	NM_001369183	Ralgapb	ACAGCAGGCACAGACAGGCAGT#GCTGCCTCAGGGTCGCCCTGTGCCCCCGCTT#..(((.((((((((((((((((#)))))))....)))....))))))..	4173	4204	0.92308	-24	0	0.0069695	0.4	2.5	3.56	-7	19	31	7	CDS
mmu-miR-214-3p	NM_001369183	Ralgapb	ACAGCAGGCACAGACAGGCAGT#AGTGTCTCTGTGTCTGGCTGA#.(((((((((((((..((((.(#).)))))))))))))).))).	2702	2723	0.84615	-23	1	9.93E-06	0.5	5.2	4.36	-5	18	21	14	CDS
mmu-miR-214-3p	NM_001369243	Chd5	ACAGCAGGCACAGACAGGCAGT#GCTGCAACCATCCGTACCTCTTCCCTGTGGCTGCTGTG#(((((((.(((((....(((((#)))))...................))))).))))))).	3278	3316	0.84615	-25	1	0.0008816	0.4	2.6	3.72	-8	17	38	7	CDS
mmu-miR-214-3p	NM_001369243	Chd5	ACAGCAGGCACAGACAGGCAGT#CTGCCAATGCTGTCCTGCA#...(((((.((((.(((((((.#)))))..)))))))))))).	5846	5865	0.84615	-23	0	0.0009532	0.4	4.5	4.43	-8	16	19	11	CDS
mmu-miR-214-3p	NM_001371039	Kctd16	ACAGCAGGCACAGACAGGCAGT#TGTGTGTCTGTGTGTGTGTGTG#((((((.(((((((((.(((..#))).))))))))).))).))).	3832	3854	0.92308	-26	0	0.0749692	0.5	0.8	0.5	-5	18	22	9	3UTR
mmu-miR-214-3p	NM_001371041	Kctd16	ACAGCAGGCACAGACAGGCAGT#TGTGTGTCTGTGTGTGTGTGTG#((((((.(((((((((.(((..#))).))))))))).))).))).	3231	3253	0.92308	-26	0	0.0749692	0.5	0.8	0.5	-5	18	22	9	3UTR
mmu-miR-214-3p	NM_001371041	Kctd16	ACAGCAGGCACAGACAGGCAGT#GCTGCTACTCTGTGACCAGGTCTGA#.((((.((((((((..((((((#))))))..)))))).))..).))).	15975	####	1	-23	0	0.0003515	0.5	0.1	-0.3	-7	18	25	6	3UTR
mmu-miR-214-3p	NM_001371043	Mcf2l	ACAGCAGGCACAGACAGGCAGT#CCGCTCTGTGCCGCTGA#.((((.((((((((..((....#))..)))))))))))).	506	523	1	-23	0	0.0005952	0.4	2.9	2.23	-7	14	17	12	CDS
mmu-miR-214-3p	NM_001371043	Mcf2l	ACAGCAGGCACAGACAGGCAGT#CTCCCTGTCTCCTCACCCTGCTGC#.(((((((...(((((((.((.#)).)))))))......))))))).	5239	5263	1	-23	1	0.1218334	0.4	-0	0.23	-9	16	24	7	3UTR
mmu-miR-214-3p	NM_001371044	Mcf2l	ACAGCAGGCACAGACAGGCAGT#ACTGATGAAATCATGCACCAGGACATCGTCCCGCTCTGTGCCGCTGA#.((((.((((((((..((....#.))..)))))))))))).	140	187	1	-23	0	0.0005228	0.4	4.2	1.94	-7	14	18	12	CDS
mmu-miR-214-3p	NM_001371044	Mcf2l	ACAGCAGGCACAGACAGGCAGT#CTCCCTGTCTCCTCACCCTGCTGC#.(((((((...(((((((.((.#)).)))))))......))))))).	4839	4863	1	-23	1	0.1218334	0.4	-0	0.23	-9	16	24	7	3UTR
mmu-miR-214-3p	NM_001371045	Mcf2l	ACAGCAGGCACAGACAGGCAGT#CCGCTCTGTGCCGCTGA#.((((.((((((((..((....#))..)))))))))))).	335	352	1	-23	0	0.0013275	0.4	2.9	2.55	-7	14	17	12	CDS

mmu-miR-214-3p	NM_001371045	Mcf2l	ACAGCAGGCACAGACAGGCAGT#CTCCCTGTCTCCTCACCCTGCTGC#.(((((((...(((((((.((.#)).)))))))......))))))).	5068	5092	1	-23	1	0.1218334	0.4	-0	0.23	-9	16	24	7	3UTR
mmu-miR-214-3p	NM_001371049	Mcf2l	ACAGCAGGCACAGACAGGCAGT#ACTGATGAAATCATGCACCAGGACATCGTCCCGCTCTGTGCCGCTGA#.((((.((((((((..((....#.))..)))))))))))).	140	187	1	-23	0	0.0005228	0.4	4.2	1.94	-7	14	18	12	CDS
mmu-miR-214-3p	NM_001371049	Mcf2l	ACAGCAGGCACAGACAGGCAGT#CTCCCTGTCTCCTCACCCTGCTGC#.(((((((...(((((((.((.#)).)))))))......))))))).	4923	4947	1	-23	1	0.1218334	0.4	-0	0.23	-9	16	24	7	3UTR
mmu-miR-214-3p	NM_001371050	Mcf2l	ACAGCAGGCACAGACAGGCAGT#CCGCTCTGTGCCGCTGA#.((((.((((((((..((....#))..)))))))))))).	484	501	1	-23	0	0.0004355	0.4	2.9	2.18	-7	14	17	12	CDS
mmu-miR-214-3p	NM_001371050	Mcf2l	ACAGCAGGCACAGACAGGCAGT#CTCCCTGTCTCCTCACCCTGCTGC#.(((((((...(((((((.((.#)).)))))))......))))))).	5217	5241	1	-23	1	0.1218334	0.4	-0	0.23	-9	16	24	7	3UTR
mmu-miR-214-3p	NM_001372387	Mthfsd	ACAGCAGGCACAGACAGGCAGT#CTGCAGTTTAGCAGGATGGAGCTGTCTGTTCTTCCTGCTGC#.(((((((....((((((((((#.))))))))))...))))))).	1180	1221	0.92308	-27	1	2.94E-07	0.4	0	0	-12	17	22	10	3UTR
mmu-miR-214-3p	NM_001372387	Mthfsd	ACAGCAGGCACAGACAGGCAGT#GCTGCTTACTGGCTTGCTC#..(((((((.(((..(((((((#))))))).)))))))))).	2009	2028	1	-23	1	0.0002427	0.5	0	0	-8	17	19	10	3UTR
mmu-miR-214-3p	NM_001372513	Dhx35	ACAGCAGGCACAGACAGGCAGT#GCTCTCTTGGCCTCCTGTGTCTTGCCTGCTGTG#((((((((((.((((((((...#.))))).....)))))))))))	2547	2580	1	-28	1	0.0016528	0.4	0	0	-7	18	25	14	3UTR
mmu-miR-214-3p	NM_001372558	Grin1	ACAGCAGGCACAGACAGGCAGT#GCCGTGATGCTGTACCTGCTGG#.(((((((.((((.(((((...#))).))...)))).))))))).	2083	2105	0.84615	-24	1	6.29E-05	0.5	0	0	-6	16	22	7	CDS
mmu-miR-214-3p	NM_001372559	Grin1	ACAGCAGGCACAGACAGGCAGT#GCCGTGATGCTGTACCTGCTGG#.(((((((.((((.(((((...#))).))...)))).))))))).	2083	2105	0.84615	-24	1	6.29E-05	0.5	0	0	-6	16	22	7	CDS
mmu-miR-214-3p	NM_001372560	Grin1	ACAGCAGGCACAGACAGGCAGT#GCCGTGATGCTGTACCTGCTGG#.(((((((.((((.(((((...#))).))...)))).))))))).	2083	2105	0.84615	-24	1	6.29E-05	0.5	0	0	-6	16	22	7	CDS
mmu-miR-214-3p	NM_001372561	Grin1	ACAGCAGGCACAGACAGGCAGT#GCCGTGATGCTGTACCTGCTGG#.(((((((.((((.(((((...#))).))...)))).))))))).	2020	2042	0.84615	-24	1	6.29E-05	0.5	0	0	-6	16	22	7	CDS

mmu-miR-214-3p	NM_001372562	Grin1	ACAGCAGGCACAGACAGGCAGT#GCCGTGATGCTGTACCTGCTGG#.(((((((.((((.(((((...#))).))...)))).))))))).	2020	2042	0.84615	-24	1	6.29E-05	0.5	0	0	-6	16	22	7	CDS
mmu-miR-214-3p	NM_001373897	Mtmr3	ACAGCAGGCACAGACAGGCAGT#GCTGCCTGAGTGGGCACATGTCTGACTCCTGTT#(((((((((((..((((((((#))))))))..))......))))))....)))).	4832	4865	0.92308	-23	1	0.0103716	0.5	0	0	-6	20	33	8	3UTR
mmu-miR-214-3p	NM_001373897	Mtmr3	ACAGCAGGCACAGACAGGCAGT#GCATGCCTGTGTACACGGACACAATCCAACAGCGCCTGCG#...((((((.((.(((((((((#)).))))))).)..................).)))))).	3318	3358	0.84615	-22	0	0.0074499	0.4	0	0	-9	17	40	7	CDS
mmu-miR-214-3p	NM_001374138	Ly6e	ACAGCAGGCACAGACAGGCAGT#GCTGGTCACACATTCCCAAGCTTTGTCTGCCCCAGAGACTTCAGAGGCCTGCTGTT#(((((((((.((((((((...#.)))).)))))................	1691	1747	0.84615	-25	1	0.0003999	0.5	0	0	-5	18	38	9	3UTR
mmu-miR-214-3p	NM_001374716	Cpsf4	ACAGCAGGCACAGACAGGCAGT#GCTGCTTGTCTGGGCTTGCTTGTG#(((((((((.((((((((((((#))))))))))))).))))))).)).	1522	1546	0.92308	-33	1	6.13E-05	0.4	0	0	-5	21	24	12	3UTR
mmu-miR-214-3p	NM_001374737	Agbl5	ACAGCAGGCACAGACAGGCAGT#GCTTGCCTGTTTCCTGCTC#..((((((...((((((((((((#))).))))))))))))))).	2513	2532	0.92308	-23	1	0.0002095	0.5	0	0	-10	17	19	14	CDS
mmu-miR-214-3p	NM_001374737	Agbl5	ACAGCAGGCACAGACAGGCAGT#GCCTGACTGTGCTG#......(((((((.(((((...#)))))).))))))).	813	827	1	-22	0	0.0003434	0.5	0	0	-16	12	14	7	CDS
mmu-miR-214-3p	NM_001374776	Rubcn	ACAGCAGGCACAGACAGGCAGT#GCTGTTTGTGTACAACCTGTGTTTAGAACTGGATTCTCACCTGCTGTA#(((((((((((((((((((((#)))))))))).......)))))).................))))))))	5362	5410	0.92308	-25	1	0.0110986	0.5	0	0	-4	22	48	9	3UTR
mmu-miR-214-3p	NM_001374802	Anks1	ACAGCAGGCACAGACAGGCAGT#CTGCGGGCTGTCTGCCTGCTGC#.((((((((..((((((((((.#))))...))))))))))))))).	4644	4666	1	-28	1	8.56E-09	0.4	0	0	-8	18	22	14	3UTR
mmu-miR-214-3p	NM_001374802	Anks1	ACAGCAGGCACAGACAGGCAGT#CTGCCTCTCTCTGATGCTTGTTGA#.(((((((((((((.((((((.#))))))...)))).))))))))).	4173	4197	1	-27	1	0.0053076	0.4	0	0	-5	19	24	9	3UTR
mmu-miR-214-3p	NM_001374802	Anks1	ACAGCAGGCACAGACAGGCAGT#CTGCCTTTGGCGCTTGTGTGAAACCAAGGCTGTGCCTGAACTGTG#(((((((((((((..((((((.#))))))......................)))))))))..)))).	5119	5164	0.84615	-26	1	0.000572	0.5	0	0	-6	19	45	9	3UTR

mmu-miR-214-3p	NM_001374802	Anks1	ACAGCAGGCACAGACAGGCAGT#ACTGCCACCTTGTGGCTGCTC#..(((((.(((((...((((((#))))))...))))).))))).	4592	4613	0.92308	-25	0	6.15E-07	0.4	0	0	-9	16	21	6	3UTR
mmu-miR-214-3p	NM_001378316	Sema4d	ACAGCAGGCACAGACAGGCAGT#GCTCCAGTCTGTGCTGCTGG#.(((((.((((((((.((.(((#))))).))))))))))))).	236	256	0.96154	-27	0	2.56E-05	0.4	0	0	-6	18	20	13	5UTR
mmu-miR-214-3p	NM_001378316	Sema4d	ACAGCAGGCACAGACAGGCAGT#ACTGTGTGTTAGCACGGGACCCCTACTGTGCCTGGAGC#.(..((((((((((((.(((((#))))).)))................))))))))..).	1886	1924	0.92308	-24	0	0.0003036	0.4	0	0	-8	18	38	9	CDS
mmu-miR-214-3p	NM_001379315	Strbp	ACAGCAGGCACAGACAGGCAGT#CTGTCTGTCCGTCTGACTGTC#(((((((((...(((((((((.#))))))))).))))).)))).	6352	6373	1	-25	1	4.56E-05	0.6	0	0	-11	18	21	9	3UTR
mmu-miR-214-3p	NM_001379315	Strbp	ACAGCAGGCACAGACAGGCAGT#ACTGCCCCATGTCTTCTGGGCCTGTGGTG#((.((((((..((((((((((#))))))...))))).....)))))).)).	16192	####	0.84615	-24	0	0.0005456	0.5	0	0	-10	19	29	6	3UTR
mmu-miR-214-3p	NM_001379354	Steap3	ACAGCAGGCACAGACAGGCAGT#CTGCCTGCCGCTGCGCCGCTC#..(((.(((.((((.(((((((.#)))))))...))).)))))).	1258	1279	0.84615	-25	0	0.0030076	0.3	0	0	-9	16	21	7	CDS
mmu-miR-214-3p	NM_001379354	Steap3	ACAGCAGGCACAGACAGGCAGT#CTGTCCCTGGGTGTGCTGGCCCTGGGCATGTTGTCGCTGCTGG#.(((((((((((..((((((((.#)))..)))))..)))))...................)))))).	1376	1419	0.84615	-23	1	0.0014271	0.4	0	0	-6	18	43	6	CDS
mmu-miR-214-3p	NM_001379479	Hr	ACAGCAGGCACAGACAGGCAGT#GCTGCCTGTTCCCTGATCCTGA#....((((..((((((((((((#)))))))))...)))..)))).	2862	2884	0.92308	-23	0	0.1404714	0.4	0	0	-12	16	22	9	CDS
mmu-miR-214-3p	NM_001382305	Garre1	ACAGCAGGCACAGACAGGCAGT#CTGCGTGGCTGTTGTCTGTGTTAGCTGTC#(((((.((((((((((.(((((#.)))))))))))))))).))))).	5637	5666	0.84615	-27	0	0.0113721	0.5	0	0	-6	20	23	15	3UTR
mmu-miR-214-3p	NM_001382849	Slc22a23	ACAGCAGGCACAGACAGGCAGT#ACTGGTTGTGTTGGCCTGCA#...((((((.(((((((.((((#)))).)))).))))))))).	4493	4513	1	-24	0	0.0003211	0.5	0	0	-8	17	20	9	3UTR
mmu-miR-214-3p	NM_007395	Acvr1b	ACAGCAGGCACAGACAGGCAGT#GCTGCCTGTCTGTCCAGCCAAGTGCG#...((((((((((((((((((#)))))))))))))...)))...))).	2094	2120	1	-30	0	0.0007874	0.4	1.1	0.77	-7	19	26	13	3UTR
mmu-miR-214-3p	NM_007395	Acvr1b	ACAGCAGGCACAGACAGGCAGT#TGCCTGGTCTGTGCCACGCC#...((.((((((((((((((..#)))))).))))))))..)).	2146	2166	0.98077	-27	0	8.98E-05	0.4	1.4	1.27	-8	16	20	8	3UTR

mmu-miR-214-3p	NM_007395	Acvr1b	ACAGCAGGCACAGACAGGCAGT#GCCTGTCCAGTTTCTGCTGTA#((((((((.((.(((((((...#)))))))..)).)))))))).	4365	4386	1	-27	1	0.0003883	0.7	3.3	2.88	-8	17	21	8	3UTR
mmu-miR-214-3p	NM_007397	Acvr2b	ACAGCAGGCACAGACAGGCAGT#CTGCCCTGGGCTGTCCTGCTGTC#((((((((.((((.(((((((.#))))).))..)))))))))))).	8915	8938	1	-29	1	0.0001143	0.3	0.2	0.05	-6	19	23	12	3UTR
mmu-miR-214-3p	NM_007397	Acvr2b	ACAGCAGGCACAGACAGGCAGT#GCCTGTCCCTGCATCCTGCCTGCTGG#.(((((((((((((((((...#)))))))..)).....))))))))).	7846	7872	1	-30	1	0.0002526	0.4	0.1	-0.1	-4	18	26	9	3UTR
mmu-miR-214-3p	NM_007479	Arf4	ACAGCAGGCACAGACAGGCAGT#ACTTTGATGTGGTTTGGATTTGATAGCCAATTCTGTGCTGGTTGC#.((((.((((((((..((((...#.)))...)))))))).)))).	1390	1435	1	-22	0	0.0011698	0.7	1	1.16	-7	15	21	8	3UTR
mmu-miR-214-3p	NM_007479	Arf4	ACAGCAGGCACAGACAGGCAGT#GCGCCTGTTCGGCAAGAAGCAGATGCGCATTTTGATGGTTGGATTGGATGCTGCTGG#.((((((((.(((((((((.((#)))))))))).............)).))......................)))))).	315	372	0.92308	-18	1	0.000544	0.6	3.4	3.46	-5	19	57	9	CDS
mmu-miR-214-3p	NM_007480	Arf5	ACAGCAGGCACAGACAGGCAGT#GCTCTGCTCTCCTGCCTGCTGG#.(((((((((.((((((((...#)).))).)))..))))))))).	818	840	0.92308	-26	1	1.25E-05	0.3	1.3	1.05	-7	17	22	9	3UTR
mmu-miR-214-3p	NM_007480	Arf5	ACAGCAGGCACAGACAGGCAGT#CTGCTCCTGCGCCTGCCTGC#.((((((((.((((...((((((.#))))).))).)))))).)).	747	767	0.84615	-24	1	0.0006298	0.4	1.2	0.75	-7	16	20	6	3UTR
mmu-miR-214-3p	NM_007488	Arnt2	ACAGCAGGCACAGACAGGCAGT#CTGCCTGGTGCAGAAGCTGG#.((((..((((...((((((((.#)))))))))))....)))).	2623	2643	0.84615	-23	0	4.02E-07	0.4	0.1	0.08	-13	15	20	11	3UTR
mmu-miR-214-3p	NM_007496	Zfhx3	ACAGCAGGCACAGACAGGCAGT#GCTGCCTGCCGATCTGGCCCAGCTC#..((((.((((.(((((((((((((#))))))))....)))))))..))).	7122	7147	0.92308	-25	0	5.15E-05	0.3	2.9	3.4	-8	18	25	8	CDS
mmu-miR-214-3p	NM_007496	Zfhx3	ACAGCAGGCACAGACAGGCAGT#GCCAGTCTGTGGTGAACCTGCA#...(((((((((((((.((((...#))).)))))))).....))))).	11276	####	0.92308	-25	0	0.0004839	0.4	3.4	2.63	-7	15	22	7	CDS
mmu-miR-214-3p	NM_007513	Slc7a1	ACAGCAGGCACAGACAGGCAGT#ACTGCTTTGTTTGTGCCTTGAAGCTT#..((((((((((((((((((((#))))))).))))))))))....))).	6642	6668	1	-27	1	0.001694	0.6	3.7	3.78	-5	20	26	10	3UTR

mmu-miR-214-3p	NM_007513	Slc7a1	ACAGCAGGCACAGACAGGCAGT#GCTGTGGTGCCTTTGCCTGCTGC#.((((((((.((.((.(((((#)))))..)).)).))))))))).	3220	3243	1	-26	1	0.0008232	0.4	0.6	0.66	-7	18	23	9	3UTR
mmu-miR-214-3p	NM_007514	Slc7a2	ACAGCAGGCACAGACAGGCAGT#GCTGCACTGTGTGGGTGTCTGCTGTT#((((((((((..((((((((#))))).))))....)))))))))).	7049	7075	0.84615	-31	1	0.0001555	0.4	-0	0.14	-9	20	26	11	3UTR
mmu-miR-214-3p	NM_007514	Slc7a2	ACAGCAGGCACAGACAGGCAGT#GCTGGGCTGTCGTGGGTCTGCTGTG#(((((((((.(((((((.((((#))))..))))).)).))))))))).	2248	2273	1	-28	1	1.72E-05	0.4	0.7	0.1	-5	20	25	9	3UTR
mmu-miR-214-3p	NM_007577	C5ar1	ACAGCAGGCACAGACAGGCAGT#ACTGTAGACCTGTGCTGTTGTG#((((((.(((((....(((((#)))))....)))))))))))).	1819	1841	1	-22	0	0.0059625	0.6	1.3	0.12	-7	17	22	12	3UTR
mmu-miR-214-3p	NM_007577	C5ar1	ACAGCAGGCACAGACAGGCAGT#TGCGTGTCCCTGGCCTACATCAACTGCTGTG#(((((((((.((((((.(((..#))).)))))..))))))).........)	985	1016	0.92308	-22	1	0.0164258	0.5	0.1	-0.1	-5	18	31	6	CDS
mmu-miR-214-3p	NM_007577	C5ar1	ACAGCAGGCACAGACAGGCAGT#ACTGCCTGTCCTGTTC#..((((((.....(((((((((#)))))))))))))).	393	409	1	-21	1	0.0048166	0.5	-0	0.11	-15	15	16	15	CDS
mmu-miR-214-3p	NM_007594	Calu	ACAGCAGGCACAGACAGGCAGT#CTGCCATTGTCTGGGCAACGATAGAGTCCTGTGCTGTA#((((((.((.(((((((((((.#)))))..)))))).)).............)))))).	1612	1650	0.92308	-23	0	0.0017366	0.5	0.1	0.31	-7	19	38	6	3UTR
mmu-miR-214-3p	NM_007594	Calu	ACAGCAGGCACAGACAGGCAGT#TGCCTGTCCCTGTGCACAGCCTTTGCTT#..(((((((((((((((((..#))))))))..))))).......))))).	133	161	0.92308	-24	1	0.0001723	0.5	2.8	2.15	-5	18	28	8	CDS
mmu-miR-214-3p	NM_007594	Calu	ACAGCAGGCACAGACAGGCAGT#TGCTTTCTCTTGCCTGTTGA#.(((((((((.(((.(((((..#))))))))..))))))))).	1539	1559	1	-22	1	0.0005397	0.5	-0	0.23	-7	17	20	9	3UTR
mmu-miR-214-3p	NM_007614	Ctnnb1	ACAGCAGGCACAGACAGGCAGT#ACTGCCTGTTGTGGTTAAACTCCTGCA#...(((((((((.((((((((#))))))))))))))........))))).	1756	1783	0.80769	-26	0	0.003937	0.5	3.7	4.23	-8	18	27	13	CDS
mmu-miR-214-3p	NM_007614	Ctnnb1	ACAGCAGGCACAGACAGGCAGT#GCTGTCTGTCTGCTCTAGCAACAAGCCGGCC#...((.(((.((((((((((((#))))))))))))))............))	1324	1355	1	-24	0	0.0002893	0.4	3.9	4.36	-10	17	31	12	CDS
mmu-miR-214-3p	NM_007614	Ctnnb1	ACAGCAGGCACAGACAGGCAGT#GCCTGTTTGTAAATCTGCC#...(((((.((((((((((...#))))))))))...))))).	2735	2754	1	-23	0	0.0017889	0.6	1.5	1.71	-8	15	19	10	3UTR

mmu-miR-214-3p	NM_007619	Cbl	ACAGCAGGCACAGACAGGCAGT#CTGTTAAGGGTGTGTGTCTCTGTTGTCTGTGTGTCCTGTCATTATAAGATTATCCTGCTGTG#(((((((((((((((.((((.#.))))))))))))))....................)))))))).	3235	3297	1	-27	1	0.002629	0.5	2.1	3.01	-4	20	44	12	3UTR
mmu-miR-214-3p	NM_007619	Cbl	ACAGCAGGCACAGACAGGCAGT#GCACTGTCTGTAGCAGAAAACAGCGCCTCTGG#.(((.(((((((((((((...#)).))))))))))..........	2396	2428	0.97436	-23	0	0.0003773	0.4	2.9	1.64	-8	17	32	8	CDS
mmu-miR-214-3p	NM_007623	Cbx2	ACAGCAGGCACAGACAGGCAGT#GCCTGTCACCCTGCTGC#.(((((((....(((((((...#)))))))..))))))).	2538	2555	0.84615	-26	1	0.0027612	0.5	-1	-0.5	-12	14	17	7	3UTR
mmu-miR-214-3p	NM_007623	Cbx2	ACAGCAGGCACAGACAGGCAGT#CTGCGCTGTCTGAGCTGAGCA#...((.(((.((((((((((((.#)))).))))))).)))..)).	1406	1427	0.92308	-24	0	0.0005218	0.4	-1	1.33	-10	16	21	7	CDS
mmu-miR-214-3p	NM_007625	Cbx4	ACAGCAGGCACAGACAGGCAGT#GCGCTGAGTCTGGCCTGCC#...((((((.(((((.(((.((#)))))..))))))))))).	3218	3237	0.92308	-24	0	5.65E-05	0.3	-0	-0.3	-8	16	19	11	3UTR
mmu-miR-214-3p	NM_007625	Cbx4	ACAGCAGGCACAGACAGGCAGT#AGTGTGTGTTTGTGTGTGTGTGG#.(((((.((((((((.(((.(#).))).))))))))).))).)).	3570	3593	0.92308	-22	0	0.0238746	0.4	2.2	1.13	-5	18	23	9	3UTR
mmu-miR-214-3p	NM_007652	Cd59a	ACAGCAGGCACAGACAGGCAGT#GCTGCTTCTGGCTGTGTTCTGTTC#..(((((((((((.((((((((#)))))..))).)))))).))))).	177	201	0.92308	-23	1	0.0022634	0.5	0	-0	-6	19	24	6	CDS
mmu-miR-214-3p	NM_007652	Cd59a	ACAGCAGGCACAGACAGGCAGT#ATTGCCTGGGCTCTGCTAGCTT#..(((.(((((.((.((((((((#))))))))..)).)))).))).	1611	1633	1	-21	0	0.0021044	0.6	0.3	0.06	-11	17	22	8	3UTR
mmu-miR-214-3p	NM_007667	Cdh8	ACAGCAGGCACAGACAGGCAGT#GTCTGTCTGGAACCACAACACTCACAGTGACCCTGACTGA#.(((((((..(((((((((...#))))))))).....................)))).))).	1353	1393	0.92308	-22	1	0.0019579	0.6	1.2	2.09	-6	16	40	9	CDS
mmu-miR-214-3p	NM_007673	Cdx2	ACAGCAGGCACAGACAGGCAGT#GCTTGTTTAGAAAGCCTGTCACCAGAGCTGCTGTA#(((((((.....((((((((...#.))))))))).......)))	1846	1881	0.92308	-24	1	0.0009656	0.5	2.6	1.97	-15	14	23	7	3UTR
mmu-miR-214-3p	NM_007701	Vsx2	ACAGCAGGCACAGACAGGCAGT#CTGCATTCTCTGCCCTGCCTGCTGA#.(((((((((((((...((((.#))))....))))...))))))))).	1798	1823	1	-26	1	0.002908	0.5	-1	-0.1	-5	17	25	9	3UTR

mmu-miR-214-3p	NM_007701	Vsx2	ACAGCAGGCACAGACAGGCAGT#ACTGCTGTTGTAGCCCATTGGTTCACATGTGCCTGCTT#..((((((((((((((.(((((#)))))))))..................)))))))))).	2375	2413	1	-26	1	0.0020788	0.5	0.1	-0.1	-5	19	38	10	3UTR
mmu-miR-214-3p	NM_007701	Vsx2	ACAGCAGGCACAGACAGGCAGT#CTGGACCCTGGCTGGCTCCTGCTGTG#((((((((..(((.(((((((.#)))...)))).)))...)))))))).	1702	1728	0.84615	-25	1	8.80E-09	0.4	-0	-0.2	-7	18	26	8	3UTR
mmu-miR-214-3p	NM_007721	Ccr10	ACAGCAGGCACAGACAGGCAGT#TGCCTTTTTGGGCCTGCG#...((((((.((((.(((((..#))))).)))).)))))).	949	967	0.84615	-24	0	1.17E-05	0.4	2.2	1.81	-8	15	18	6	CDS
mmu-miR-214-3p	NM_007721	Ccr10	ACAGCAGGCACAGACAGGCAGT#GTCTTCGTGTGGCTGTTGTC#(((((((.((((((.((((...#)))))).)))).))))))).	506	526	0.84615	-21	1	8.59E-05	0.4	0.3	0.31	-7	17	20	7	CDS
mmu-miR-214-3p	NM_007764	Crkl	ACAGCAGGCACAGACAGGCAGT#ATTGCTGTGCTGTCCCTGCTGC#.(((((((.((((...((((((#))))))...)))).))))))).	1404	1426	1	-26	1	0.0047068	0.4	1.1	2.38	-7	17	22	7	CDS
mmu-miR-214-3p	NM_007778	Csf1	ACAGCAGGCACAGACAGGCAGT#CTGCCTGCTGAACAGCCTGTC#...((((((.(((.(((((((.#))))))))))....)))))).	2871	2892	0.92308	-25	0	0.0183691	0.5	0.2	0.4	-9	16	21	10	3UTR
mmu-miR-214-3p	NM_007778	Csf1	ACAGCAGGCACAGACAGGCAGT#TGTCTGTGTGTGTCTGGCGG#.(..((((((((.(((((((..#))))))).))))))))..).	129	149	0.92308	-26	0	0.0003268	0.5	1.5	1.59	-10	16	20	8	5UTR
mmu-miR-214-3p	NM_007778	Csf1	ACAGCAGGCACAGACAGGCAGT#GTCTGTCTTCCACCTGCTGG#.(((((((...((((((((...#))))))))....))))))).	1881	1901	0.84615	-26	1	0.0002574	0.4	0.5	0.81	-9	15	20	8	CDS
mmu-miR-214-3p	NM_007789	Ncan	ACAGCAGGCACAGACAGGCAGT#ACAGTATGTCTGTGTGTGCTGG#.(((((.(((((((((.(((.((#)).)).)))))))))).))))).	4179	4201	0.92308	-27	0	4.21E-06	0.4	1.1	1.18	-5	18	22	9	3UTR
mmu-miR-214-3p	NM_007789	Ncan	ACAGCAGGCACAGACAGGCAGT#CTGCTGCTGTGGCTGCTT#..(((((.(((((.(((.((((.#))))))))))))).))))).	157	175	1	-25	0	0.0006997	0.4	0	0.43	-8	16	18	11	CDS
mmu-miR-214-3p	NM_007789	Ncan	ACAGCAGGCACAGACAGGCAGT#CTGCCTGCCGCTGCCTCCCAAGAGCTGG#.(((((((((..(.(((((((.#))))))).)..)))))).......)))).	5512	5540	1	-25	1	0.0001212	0.4	-0	1.03	-11	17	28	7	3UTR
mmu-miR-214-3p	NM_007857	Dhh	ACAGCAGGCACAGACAGGCAGT#GCCTAGAGGTCATCCTGCCTGCTA#..((((((((..(((((((...#))))....)))....)))))))).	2230	2254	0.92308	-22	1	0.0012363	0.6	0.4	0.13	-11	15	24	8	3UTR

mmu-miR-214-3p	NM_007865	Dll1	ACAGCAGGCACAGACAGGCAGT#GCTGCCGCTGGACTGCCTGCTGG#.((((((((((((((.(.((((((#))))))))))...)))))))))).	2936	2959	1	-30	1	1.03E-05	0.4	-0	-0.3	-5	19	23	10	3UTR
mmu-miR-214-3p	NM_007865	Dll1	ACAGCAGGCACAGACAGGCAGT#GCTGCTGGGCTGTGCTGCTGTG#((((((.((((((...((((((#))))))...))))))))))))).	2256	2278	0.92308	-29	0	1.23E-05	0.4	2.7	3.48	-7	18	22	12	CDS
mmu-miR-214-3p	NM_007865	Dll1	ACAGCAGGCACAGACAGGCAGT#CTGCCCTGCTGTGCCAGGTCTGG#.((((.(((((((.(((((((.#))))).)))))))))..).))).	620	643	1	-27	0	0.0001882	0.4	1.4	1.66	-7	18	23	9	CDS
mmu-miR-214-3p	NM_007896	Mapre1	ACAGCAGGCACAGACAGGCAGT#GCTGAATGTGAATGAAGAAGTATCTGTGAGGATCTGGGTCTGCTGTG#(((((((((.(((((..((((.#.))))...).)))).))))))))).	5720	5767	1	-24	1	0.0001236	0.4	-0	0.05	-5	18	25	9	3UTR
mmu-miR-214-3p	NM_007896	Mapre1	ACAGCAGGCACAGACAGGCAGT#GCTGTCTGCTGGTCACTGCTC#..((((((..((((.(((((((((#))))))))))))..).))))).	1965	1986	1	-24	1	0.0073552	0.4	0.8	1.33	-8	17	21	11	3UTR
mmu-miR-214-3p	NM_007938	Epha6	ACAGCAGGCACAGACAGGCAGT#ACTATCTGCTGTGCCTCCTCTGC#.(((.(((((((.((((.(((#))).)))))))))))...))).	36	59	1	-23	0	0.0039129	0.4	0.6	1.11	-9	18	23	12	5UTR
mmu-miR-214-3p	NM_007938	Epha6	ACAGCAGGCACAGACAGGCAGT#GCTGTTGGTTTATTTTTAATGACTTAACTGTTGGTGCCTGATGTT#(((.(((((((.((((((((((#)))))......................))))).))))))).))).	4145	4190	0.92308	-23	0	0.0082961	0.7	0	0.3	-10	20	45	7	3UTR
mmu-miR-214-3p	NM_007948	Ercc1	ACAGCAGGCACAGACAGGCAGT#ACTTCGCCCTTCGTGTGCTGCTGG#.(((((.(((((((..((((((#)))..)))..)).)))))))))).	620	644	1	-22	0	0.0007437	0.4	2.1	3.19	-6	18	24	10	CDS
mmu-miR-214-3p	NM_007948	Ercc1	ACAGCAGGCACAGACAGGCAGT#GCTGGCTGTGCTGGCGTTGTGTCGCCTCTGTT#((((.((((.(((((((.((((#)))).)))).)))..........)	53	85	0.92308	-21	0	0.0268387	0.4	-0	0.07	-9	19	32	8	5UTR
mmu-miR-214-3p	NM_007948	Ercc1	ACAGCAGGCACAGACAGGCAGT#AGTGCCTCGATGACCTGCTGC#.(((((((((..((..((((.(#).))))))..)).))))))).	1017	1038	0.92308	-21	1	0.0009627	0.5	0.1	1.07	-11	16	21	7	CDS
mmu-miR-214-3p	NM_007949	Ercc2	ACAGCAGGCACAGACAGGCAGT#ATCGCCTGCTGTGCCTCCCTGG#.(((.((((((((.(((((.((#)).))))))))))))))..))).	3127	3149	0.92308	-26	0	0.0001678	0.4	0.6	-0.1	-9	18	22	13	3UTR
mmu-miR-214-3p	NM_007963	Mecom	ACAGCAGGCACAGACAGGCAGT#GCTGGTTGTCTGCAAGCTTGTG#...((((((.(((((((.((((#)))).)))))))...)))))).	4119	4141	1	-23	0	0.0016131	0.6	1.5	1.32	-8	17	22	7	3UTR

mmu-miR-214-3p	NM_007963	Mecom	ACAGCAGGCACAGACAGGCAGT#CTGGCTGCTGCTGATCTTATTTTTGTTTAATTCTGCTGTG#((((((((((((.(((.(((.#))).)))))).)).................)))))))).	582	622	0.92308	-21	1	0.0231137	0.6	4.3	2.6	-5	19	40	8	5UTR
mmu-miR-214-3p	NM_007970	Ezh1	ACAGCAGGCACAGACAGGCAGT#GCTGGTCTGGTTTACTCCCTGTCTGTAGTACCTGCTGTT#((((((((((((((((.(((#.))).))))))))).)..)))))))).	3158	3197	0.88462	-30	1	0.0017932	0.5	4.2	3.93	-4	21	27	9	3UTR
mmu-miR-214-3p	NM_008139	Gnaq	ACAGCAGGCACAGACAGGCAGT#GCTGCAGTGGTGCCTGCA#...((((((((..(((..((((((#))))).)).)))))))).	4704	4722	0.92308	-25	0	3.50E-05	0.5	0.6	1.63	-11	15	18	8	3UTR
mmu-miR-214-3p	NM_008169	Grin1	ACAGCAGGCACAGACAGGCAGT#GCTGCTGGTGGGGCTGTCAGTTCATGTGGTGGCCGTGATGCTGTACCTGCTGG#.(((((((.((((.(((((...#.))).))...)))).))))))).	1989	2042	0.84615	-24	1	6.29E-05	0.5	4.6	4.06	-6	16	23	7	CDS
mmu-miR-214-3p	NM_008231	Hdgf	ACAGCAGGCACAGACAGGCAGT#CTGCCCCGTTCCTGCTGCTGTCTGGGTGCTACTGGGGAAACTGGCCATGGCCTGCA#...((((((.((((((.(((((#.))))))))))).........................)))))).	1045	1101	0.84615	-24	0	0.0015584	0.4	2.1	2.13	-8	17	44	11	3UTR
mmu-miR-214-3p	NM_008231	Hdgf	ACAGCAGGCACAGACAGGCAGT#CTGCTGGGGTCTAGGCTGCTGTT#(((((((.(..((((.(((((.#)))))...)))).).))))))).	1272	1295	0.92308	-23	1	7.02E-05	0.4	-0	0.11	-11	17	23	7	3UTR
mmu-miR-214-3p	NM_008247	Plpp1	ACAGCAGGCACAGACAGGCAGT#ACTGCCCTGGTCCCTGCTC#..((((((..(((...((((((#)))))))))...)))))).	274	293	1	-21	1	0.0066964	0.4	-0	0.29	-9	15	19	9	5UTR
mmu-miR-214-3p	NM_008258	Jpt1	ACAGCAGGCACAGACAGGCAGT#GCTTTTGGAGTCGTCTGCGCCTGTTGG#.((((((((.(((((.(((...#.)))))))).)))))))).	39	66	0.92308	-27	1	6.58E-05	0.4	0.6	1.7	-5	16	19	8	5UTR
mmu-miR-214-3p	NM_008258	Jpt1	ACAGCAGGCACAGACAGGCAGT#CTGTCTGTCTGACTCCTGGGTCTGTG#((((((((..(((((((((((.#)))))))))))...))))...)))).	743	769	1	-26	1	3.54E-05	0.5	2.9	1.63	-6	19	26	11	3UTR
mmu-miR-214-3p	NM_008258	Jpt1	ACAGCAGGCACAGACAGGCAGT#CTGTCTGTCTGTTTGTTGTC#(((((((..(((((((((((((.#)))))))))))))))))))).	608	628	1	-26	1	0.0901037	0.5	1.6	0.9	-7	19	20	19	3UTR
mmu-miR-214-3p	NM_008277	Hpd	ACAGCAGGCACAGACAGGCAGT#ACAAGAGTCCGCCTTGCCTGAGTCCTCCCTGCTGA#.(((((((......(((((((.#.))))))).......))	1341	1376	0.92308	-23	1	0.0303701	0.5	2.3	1.24	-14	14	23	7	3UTR

mmu-miR-214-3p	NM_008277	Hpd	ACAGCAGGCACAGACAGGCAGT#TGTTTGTTCTCTGCTCTGCTC#..((((((((.(((((((((..#))))))).)).))).))))).	249	270	0.84615	-20	1	0.0003129	0.5	3.1	2.88	-8	17	21	7	CDS
mmu-miR-214-3p	NM_008308	Htr1a	ACAGCAGGCACAGACAGGCAGT#CTGTTTATCGCCCTGGATGTGCTGTGCTGC#.(((((((((((..((((....#.))))..)))))).))))).	891	921	1	-25	1	6.10E-05	0.4	3.2	4.1	-6	15	20	6	CDS
mmu-miR-214-3p	NM_008308	Htr1a	ACAGCAGGCACAGACAGGCAGT#GCTGTATCTTTCCAATGTCTGCACCTGCTGTT#((((((((..((((((.(((((#)))))..........))))))..)))	3574	3606	1	-24	1	0.0004475	0.6	-0	0.07	-6	19	32	8	3UTR
mmu-miR-214-3p	NM_008308	Htr1a	ACAGCAGGCACAGACAGGCAGT#TGCCTGCGTGGTTGCTGC#.((((((.(((.(.((((((..#)))))))))).)))))).	722	740	0.84615	-25	1	2.48E-05	0.4	3.9	2.88	-10	16	18	10	CDS
mmu-miR-214-3p	NM_008320	Irf8	ACAGCAGGCACAGACAGGCAGT#GCTGGCCTTTCTGTGTGCTGA#.((((..(((((((.(((((((#)))).))).)))))))))))).	1566	1587	1	-24	0	0.0010206	0.5	0.2	-0.1	-8	18	21	11	3UTR
mmu-miR-214-3p	NM_008320	Irf8	ACAGCAGGCACAGACAGGCAGT#GCTGCCGTCTCTCCCTGAGCCAGCCGGGGCTGCCTAAGTTGTA#((((((((((((((.((((((#))))))))))).................).)))))..))))).	729	772	0.84615	-22	1	0.0007772	0.3	0.9	1.91	-4	21	43	10	CDS
mmu-miR-214-3p	NM_008331	Ifit1	ACAGCAGGCACAGACAGGCAGT#GCTGAGCAAGAGAAGCCTGGCGACCTGGGGCAACTGTGCCTGGCTGC#.(((((((((((((.((((..((#.))..)))).......)))))))).))).	351	398	0.92308	-25	1	7.73E-06	0.4	2	1.1	-5	18	29	9	CDS
mmu-miR-214-3p	NM_008331	Ifit1	ACAGCAGGCACAGACAGGCAGT#TGTCTGTGCCTTTGG#.(((.(((((((((((......#))))))))))))))).	1766	1781	1	-22	0	0.0008044	0.6	-0	-0.1	-8	14	15	14	3UTR
mmu-miR-214-3p	NM_008349	Il10rb	ACAGCAGGCACAGACAGGCAGT#CTCCTGGGCTGCTTTGTCGTGCTGTG#((((((.(.(((...(((((((#.))))))))))).)))))).	800	826	0.92308	-21	0	0.0052569	0.5	-0	0.64	-9	17	20	11	CDS
mmu-miR-214-3p	NM_008364	Il1rap	ACAGCAGGCACAGACAGGCAGT#CTGTGTGAGCTGAGGCTATTTCACTCCTTTGCTTCTGTGTCTGTTGA#.(((((((((((((..(((((.#.))))))))))))))))))).	2346	2393	1	-26	1	0.0013303	0.6	0.3	-0	-5	18	20	18	3UTR
mmu-miR-214-3p	NM_008365	Il18r1	ACAGCAGGCACAGACAGGCAGT#TGTGTGTGTGTGTCTGTCTGTC#(((((((((((((.(((.(((..#))).))).)))))))).)))).	3679	3701	1	-27	1	0.4730247	0.5	-0	0.09	-5	18	22	8	3UTR

mmu-miR-214-3p	NM_008417	Kcna2	ACAGCAGGCACAGACAGGCAGT#GCCAGCCTGTCTTGTTGAATTTGTGTCTGTCTGTC#(((((((((((((((((((.((#))..))))))))..........))))))).)))).	7871	7906	1	-26	1	0.0062272	0.5	0.6	0.55	-4	21	35	8	3UTR
mmu-miR-214-3p	NM_008417	Kcna2	ACAGCAGGCACAGACAGGCAGT#GCTGCCTATTTGTGTATCTGTGCCATCAGATCTGCTC#..((((((((((((.(((((((#)))))))........))))))))........)))).	448	485	0.84615	-24	1	1.11E-05	0.5	1.2	1.19	-6	19	37	8	5UTR
mmu-miR-214-3p	NM_008423	Kcnd1	ACAGCAGGCACAGACAGGCAGT#TGCTTTCTTCTTGGGTTGGTCTGGAGTCTGGACCTGCTGA#.(((((((..(((((((((...#.)))))...)))))..))))))).	2858	2898	0.84615	-26	1	0.0001491	0.5	0.5	0.98	-6	16	23	7	3UTR
mmu-miR-214-3p	NM_008423	Kcnd1	ACAGCAGGCACAGACAGGCAGT#GCCTGTTCTCATCCTGCTGC#.(((((((...((((((((...#)))))).))...))))))).	1663	1683	0.84615	-25	1	0.0034852	0.4	0.9	2.88	-9	15	20	7	CDS
mmu-miR-214-3p	NM_008423	Kcnd1	ACAGCAGGCACAGACAGGCAGT#ATTGCCTTGCCTGTGCCAGTCATTGTA#(((((.(((((((.((((((((#)))))))).).))))))).))...))).	1281	1308	0.84615	-23	0	0.0147709	0.5	3.6	4.36	-7	20	27	7	CDS
mmu-miR-214-3p	NM_008440	Kif1a	ACAGCAGGCACAGACAGGCAGT#CTGACCTGTACATATGTGGTTGCTGTG#(((((((.(((((((((((((.#))).))))).)...)))).))))))).	8084	8111	0.82051	-25	1	0.0012501	0.4	3.7	1.83	-6	20	27	7	3UTR
mmu-miR-214-3p	NM_008440	Kif1a	ACAGCAGGCACAGACAGGCAGT#GCTGATGAAGTTAACAACAACACCTGTTCAGCTGTGCCTCCTGA#.(((.((((((((((((....#.)))))....))))))).))).	3039	3083	0.84615	-24	0	0.0001367	0.5	3.8	2.99	-8	16	23	8	CDS
mmu-miR-214-3p	NM_008441	Kif1b	ACAGCAGGCACAGACAGGCAGT#ACTTGGGCGAGTGAGGCCTGCTGTG#(((((((((.(..((..((...#.))..))..)))))))))).	5414	5439	0.84615	-23	1	1.83E-05	0.5	4.4	4.36	-10	14	20	10	3UTR
mmu-miR-214-3p	NM_008465	Kpna1	ACAGCAGGCACAGACAGGCAGT#ACTCCCGTCTGTCCTGTTGTT#((((((((.((((((.((.(((#))))).)))))))))))))).	3649	3670	1	-25	1	0.0041994	0.6	2	2.27	-5	19	21	14	3UTR
mmu-miR-214-3p	NM_008465	Kpna1	ACAGCAGGCACAGACAGGCAGT#CTGCTCTGGGATGGATGCTGTCTGATGTCTGTTA#..(((((((((((((((((((.#))))............))))))).))))))).	3585	3619	1	-24	1	0.0016019	0.5	2.1	1.59	-5	19	34	8	3UTR
mmu-miR-214-3p	NM_008466	Kpna3	ACAGCAGGCACAGACAGGCAGT#ACTTCAGCCTGTGTGTTCCTGCTGTT#((((((((.(((.((((((...#.)))))).))).)))))))).	2438	2464	0.90385	-31	1	0.0060107	0.6	5.9	3.44	-6	17	21	8	3UTR

mmu-miR-214-3p	NM_008473	Krt1	ACAGCAGGCACAGACAGGCAGT#ATTTGGTAGTATGGGGCCTGTCTGTCCCCCTGGTGG#.((.((((.((((((((((...#.))))))))))...	499	535	0.92308	-29	0	6.09E-06	0.5	1.3	0.23	-8	16	22	10	CDS
mmu-miR-214-3p	NM_008520	Ltbp3	ACAGCAGGCACAGACAGGCAGT#CTGCCAGGTGCCTGCTGC#.((((((((((.....(((((.#)))))..)))))))))).	1399	1417	0.92308	-28	1	1.06E-05	0.3	1	1.79	-10	15	18	10	CDS
mmu-miR-214-3p	NM_008520	Ltbp3	ACAGCAGGCACAGACAGGCAGT#GCTCCTGTCTGCAGAGTGCTGTCTGCTT#..((((((((((((((((.(((#)))))))))))))........)))))))).	4918	4946	0.80769	-26	1	0.0003469	0.5	-0	0.2	-5	19	28	11	3UTR
mmu-miR-214-3p	NM_008529	Ly6e	ACAGCAGGCACAGACAGGCAGT#GCTGGTCACACATTCCCAAGCTTTGTCTGCCCCAGAGACTTCAGAGGCCTGCTGTT#(((((((((.(((((((((...#.)))).)))))................	2073	2129	0.84615	-25	1	0.0003999	0.5	0	0	-5	18	38	9	3UTR
mmu-miR-214-3p	NM_008547	Mak	ACAGCAGGCACAGACAGGCAGT#CTGCCCGTCTCGTTTTAGTCCTGTTGTT#((((((((((((((((.((((((.#)))))).)))).))....).)))))))).	2692	2720	1	-24	1	0.0220696	0.5	3.2	4.36	-4	20	28	8	3UTR
mmu-miR-214-3p	NM_008547	Mak	ACAGCAGGCACAGACAGGCAGT#GCCTGTCTCCAGTGTAGCA#...((..((((((((((((...#))))))))...)))).)).	103	122	1	-21	0	0.0001661	0.6	1	0.65	-10	14	19	8	5UTR
mmu-miR-214-3p	NM_008551	Mapkapk2	ACAGCAGGCACAGACAGGCAGT#AGTGCCCACACATTGTGCACATCGTGGATGTCTATGAGAACCTGTATGCCGGGAGGAAGTGCCTGCTGA#.((((((((((.....((((..#.))))........)))))))))).	613	682	0.92308	-25	1	0.0008537	0.5	-0	-0.1	-10	14	24	10	CDS
mmu-miR-214-3p	NM_008614	Mobp	ACAGCAGGCACAGACAGGCAGT#TTGCTTTTACCAGAAGAAGGAGGAGGACTGGATCTGCTGTGCCTGCC#...((((((((((.((((((((#.))))..)))))))))))))).	288	335	0.97436	-28	0	4.47E-05	0.4	2.5	2.65	-7	18	22	14	CDS
mmu-miR-214-3p	NM_008614	Mobp	ACAGCAGGCACAGACAGGCAGT#TGCGTGTGTGTGTTTGCG#...(((((((((.(((.(((..#))).))).))))))))).	1494	1512	0.92308	-25	0	0.000114	0.4	-0	-0.1	-8	15	18	9	3UTR
mmu-miR-214-3p	NM_008626	Mrc2	ACAGCAGGCACAGACAGGCAGT#GCTGAAGAGGCTGGCCAAGCATCTGTCACTCCTGTGCCTGCTGG#.((((((((((((...(((((.#.)))))....)))))))))))).	5200	5244	1	-31	1	2.17E-05	0.4	0.1	0.22	-6	17	23	12	3UTR
mmu-miR-214-3p	NM_008635	Map7	ACAGCAGGCACAGACAGGCAGT#GCCACCTGAGCCTGCTGC#.((((((((.(((...((((...#)))..))).)))))))).	1326	1344	1	-25	1	0.0002441	0.3	0.7	0.25	-7	14	18	8	CDS

mmu-miR-214-3p	NM_008635	Map7	ACAGCAGGCACAGACAGGCAGT#ACTGTCAGCCTCTCCTGTCTTATCATTCTTCACTCCTGCTGCTCAGCCCTGCTGTA#((((((((.(((.((((.(((#.))))))))))....))).)))))).	3422	3478	0.92308	-23	1	0.0017568	0.6	0.1	0.47	-6	19	26	10	3UTR
mmu-miR-214-3p	NM_008676	Nbr1	ACAGCAGGCACAGACAGGCAGT#GCTCCTCGTCTGCCTGCTGC#.((((((((..(((((((.(((#)))))).))))))))))).	1325	1345	1	-25	1	0.0028815	0.5	1.5	1.64	-8	18	20	12	CDS
mmu-miR-214-3p	NM_008676	Nbr1	ACAGCAGGCACAGACAGGCAGT#GCTGCCAGTGAAAGGGAGCTGTACATTCCATCTGTGGACCTCCTGACTGCCCAGGACCTGCTGTC#((((((((((((((.((((((#)))))).)......................))))))...................)))))))).	2117	2182	0.84615	-24	1	1.61E-05	0.5	3.5	2.38	-4	21	65	8	CDS
mmu-miR-214-3p	NM_008676	Nbr1	ACAGCAGGCACAGACAGGCAGT#TTGTCTGTTGCTGCCTCTGC#.(((.(((((..(((((((((.#)))))))))..)))))))).	3227	3247	1	-23	0	0.0011664	0.4	3.3	2.54	-14	17	20	9	CDS
mmu-miR-214-3p	NM_008684	Neo1	ACAGCAGGCACAGACAGGCAGT#TTGCCTGCTGAGAGCCTGCA#...((((((.(((.(((((((.#)))))))))))...)))))).	5003	5023	0.87179	-27	0	1.68E-05	0.4	2.3	3.19	-9	16	20	10	3UTR
mmu-miR-214-3p	NM_008688	Nfic	ACAGCAGGCACAGACAGGCAGT#GCTGCCTGCAGCCCTTCTGCGCCCTGAGGG#.(..(((((.((((((((((((#))))))))).......)))).))).)).	1670	1700	0.84615	-24	0	1.06E-05	0.4	0.4	0.19	-10	18	30	8	3UTR
mmu-miR-214-3p	NM_008688	Nfic	ACAGCAGGCACAGACAGGCAGT#GCCTGAGCACCATTGACTGCCTGCTGTT#((((((((((((.(((((...#)))))).......)))..))))))))))).	6139	6167	0.92308	-24	1	0.0361053	0.5	3.8	4.11	-5	18	28	10	3UTR
mmu-miR-214-3p	NM_008739	Nsd1	ACAGCAGGCACAGACAGGCAGT#GCTTGCTGGTCTGCTGG#.((((((((.(((.(((((...#))))))))))))))))).	8040	8057	0.94872	-27	1	1.78E-05	0.5	1.7	1.35	-6	16	17	16	CDS
mmu-miR-214-3p	NM_008739	Nsd1	ACAGCAGGCACAGACAGGCAGT#GCTGACCTTGTCTGTTCCTGTGGCTCCAGAAGCCTCTCCGCGGCCTGCC#...((((((((((((((((((#)))).))).))))))).........................)))	4223	4272	0.84615	-25	0	0.0012912	0.4	1.7	1.9	-7	19	49	6	CDS
mmu-miR-214-3p	NM_008739	Nsd1	ACAGCAGGCACAGACAGGCAGT#GCTGCCTGAGCTGGGGCCCAAGGGCTGG#.((((.(((.(((.((((((((#))))))))..)))..))).....)))).	9532	9560	1	-25	0	9.41E-07	0.4	2.3	1.55	-8	18	28	8	3UTR
mmu-miR-214-3p	NM_008739	Nsd1	ACAGCAGGCACAGACAGGCAGT#GCAGCCTTCTGTGCTGTGATTCTTGCCCTGCTGC#.((((((((((((.((((.((#)).)))))))))))).............)))))).	5263	5297	1	-25	1	0.0215899	0.5	4.2	4.3	-5	19	34	11	CDS

mmu-miR-214-3p	NM_008842	Pim1	ACAGCAGGCACAGACAGGCAGT#GCTGCTTGCTGGAGTGGGTCTAGGGTCTGCTGA#.((((((((.(((.((((((((#))))))))))))...........)))))))).	2453	2486	0.92308	-25	1	3.75E-05	0.5	0.2	0.81	-6	19	33	11	3UTR
mmu-miR-214-3p	NM_008842	Pim1	ACAGCAGGCACAGACAGGCAGT#AGTGCCTTCTGTGGGGACTCCGCCTGTGCTGG#.(((((((((((((.(((((.(#).))))))))))))........	1712	1744	0.84615	-22	1	0.0001831	0.5	2	1.52	-5	19	32	10	3UTR
mmu-miR-214-3p	NM_008876	Pld2	ACAGCAGGCACAGACAGGCAGT#ACTGCCTCCGCCAGCCTGCTGA#.((((((((......(((((((#)))))))......)))))))).	3613	3635	0.80769	-27	1	0.0012041	0.5	0.6	0.67	-14	15	22	8	3UTR
mmu-miR-214-3p	NM_008876	Pld2	ACAGCAGGCACAGACAGGCAGT#CTGGATCTCCGAGACCCTGTCTGTGATGACTTCTTCCAGCTGTG#(((((.((((((((((((....#.)))))))))))..........)).))))).	2837	2881	0.92308	-25	0	0.0003802	0.5	2.3	2.17	-6	17	30	10	CDS
mmu-miR-214-3p	NM_008876	Pld2	ACAGCAGGCACAGACAGGCAGT#GCTCCTGGCTGAGTTCTGCCTGCTT#..(((((((((((.((((.(((#))))))).)))).....)))))))).	13	38	0.92308	-24	1	2.54E-05	0.3	-0	0.31	-6	18	25	8	5UTR
mmu-miR-214-3p	NM_008881	Plxna1	ACAGCAGGCACAGACAGGCAGT#ACTTCCTGTGAGCTGTGCCTGGGGTC#((..((((((((((((((.(((#))).)))))...))))))))))..)).	1828	1854	0.84615	-26	0	7.54E-06	0.4	-1	-0.1	-9	19	26	9	CDS
mmu-miR-214-3p	NM_008881	Plxna1	ACAGCAGGCACAGACAGGCAGT#ATTGCCTGCCTCTGCGCTTCTGG#.(((.((((.(((((((((((((#))))))))))..)))).))))))).	5501	5524	0.84615	-25	0	0.0003379	0.5	3.5	2.28	-9	19	23	8	CDS
mmu-miR-214-3p	NM_008901	Pou3f4	ACAGCAGGCACAGACAGGCAGT#GGTGCGTGTCTGGTTCTGTA#...(((((..((((((.((((.(#).))).))))))..))))).	1048	1068	0.84615	-22	0	0.0001505	0.6	4.5	4.25	-9	15	20	6	CDS
mmu-miR-214-3p	NM_008901	Pou3f4	ACAGCAGGCACAGACAGGCAGT#ATTGTCTGCGGTGTTTTACTACAAGCTGTA#(((((((((((.(.((((((((#))))))))).))))))........)))	1775	1805	0.92308	-20	1	0.0016874	0.7	3.1	2.17	-8	20	30	9	3UTR
mmu-miR-214-3p	NM_008903	Plpp1	ACAGCAGGCACAGACAGGCAGT#ACTGCCCTGGTCCCTGCTC#..((((((..(((...((((((#)))))))))...)))))).	274	293	1	-21	1	0.0066964	0.4	-0	0.29	-9	15	19	9	5UTR
mmu-miR-214-3p	NM_008904	Ppargc1a	ACAGCAGGCACAGACAGGCAGT#ACTGTCTGTCCTGTCGAATGCTTGCTC#..(((((((((((((((((((#)))))))))))).))).....))))))).	5034	5061	1	-27	1	0.010281	0.6	2	1.66	-5	20	27	10	3UTR
mmu-miR-214-3p	NM_008904	Ppargc1a	ACAGCAGGCACAGACAGGCAGT#TTGCCTGCATGAGTGTGTGCTGTG#((((((.((((...(((((((.#)))))))).....)))).)))))).	62	86	0.92308	-26	0	0.0022204	0.5	2.9	4.38	-11	17	24	7	5UTR

mmu-miR-214-3p	NM_008904	Ppargc1a	ACAGCAGGCACAGACAGGCAGT#TGACACTGCTGATGCCTGTTGTC#(((((((((((((.((((((..#)).).)))))).)))))))))).	3095	3118	1	-24	1	8.19E-06	0.5	1.3	1.41	-5	19	23	10	3UTR
mmu-miR-214-3p	NM_008904	Ppargc1a	ACAGCAGGCACAGACAGGCAGT#TGTGTGTGTGTGTCCGTTGA#.((((.((((((.(((.(((..#))).))).)))))).)))).	3877	3897	0.92308	-23	0	0.0277999	0.5	3.5	2.35	-7	16	20	6	3UTR
mmu-miR-214-3p	NM_008937	Prox1	ACAGCAGGCACAGACAGGCAGT#ACTGCCTTGTGTGCCTT#.....(((((((.(.(((((((#)))))))).))))))).	7933	7950	0.92308	-24	0	0.0249413	0.5	4.5	3.15	-16	15	17	8	3UTR
mmu-miR-214-3p	NM_008937	Prox1	ACAGCAGGCACAGACAGGCAGT#GCTGTCTGTCTTTTCCCTCTGG#.(((.(((...((((((((((#))))))))))))....)))))).	3853	3875	1	-23	0	0.0005861	0.5	0.8	1.06	-13	17	22	11	3UTR
mmu-miR-214-3p	NM_008937	Prox1	ACAGCAGGCACAGACAGGCAGT#GTTTGCTTTGTGCCTCTGA#.(((.(((((((((((((...#))))).)))))))))))).	6203	6222	1	-23	0	0.0218659	0.7	3.6	3.68	-8	17	19	12	3UTR
mmu-miR-214-3p	NM_008959	Ptdss1	ACAGCAGGCACAGACAGGCAGT#GCTGCCAGCAGTGGCTCTCTGCTGGTGTGTCTTGTGGATGTTGTA#((((((..((((((((((((((#))))))....................))))).)))..)))))).	2388	2433	0.84615	-23	0	0.0221679	0.6	0.2	0.01	-8	20	45	6	3UTR
mmu-miR-214-3p	NM_008959	Ptdss1	ACAGCAGGCACAGACAGGCAGT#GCTGCCTTCTCCCCCTGCTC#..((((((...(((.((((((((#))))))))))...)))))).	471	491	0.84615	-23	1	0.0153468	0.3	0.3	-0.3	-11	16	20	10	5UTR
mmu-miR-214-3p	NM_008959	Ptdss1	ACAGCAGGCACAGACAGGCAGT#CTGCCTGTCCATTTGATTGATTGTTTAAGTTCAGAGAGTCTGCTT#..((((((((((((((((((.#)))))))))...........)))............))))))).	2506	2551	0.92308	-23	1	0.0019166	0.7	0.3	0.29	-5	19	45	9	3UTR
mmu-miR-214-3p	NM_008961	Pter	ACAGCAGGCACAGACAGGCAGT#CCTGACTCTGTGATCTGCA#...((((((((((((((....#))))..)))))).))))).	1522	1541	0.92308	-21	0	0.0096744	0.6	0.4	0.9	-7	15	19	6	3UTR
mmu-miR-214-3p	NM_008961	Pter	ACAGCAGGCACAGACAGGCAGT#GCTGCTGGAGTTTGCTCAACTTGGCTGCTA#..(((((.(((((((.((((((#))))))...)))))).......)).)))))	903	933	0.92308	-20	0	0.0041135	0.5	3.5	3.48	-8	18	30	6	CDS
mmu-miR-214-3p	NM_008970	Pthlh	ACAGCAGGCACAGACAGGCAGT#ATTGCCTGTACATACTGTAAATGCA#...(((...(((((((((((((#)))))))))).....))))...))).	1060	1085	0.84615	-19	0	0.0276837	0.6	0.5	0.82	-11	16	25	9	3UTR
mmu-miR-214-3p	NM_008982	Ptprj	ACAGCAGGCACAGACAGGCAGT#CTGTGTGTCTGTGTGTGTCTCTGTG#((((((.(((((((((.((((.#)))).))))))))).))...)))).	5151	5176	0.92308	-28	0	0.1576382	0.6	0	0	-5	19	25	9	3UTR

mmu-miR-214-3p	NM_008982	Ptprj	ACAGCAGGCACAGACAGGCAGT#ACCCCCGAAATGCTTGATTGTGCTTGTTGA#.((((((((((((.((((((..#.)))))).)))))))))))).	7233	7263	1	-26	1	0.0002306	0.5	0	0	-5	18	21	12	3UTR
mmu-miR-214-3p	NM_008982	Ptprj	ACAGCAGGCACAGACAGGCAGT#ACTGCCTTCTGATGTTCTGCC#...(((((((((((.(((((((#))))))))))).))).)))).	5793	5814	0.81538	-24	0	0.1601578	0.4	0	0	-7	18	21	11	3UTR
mmu-miR-214-3p	NM_008988	Igdcc3	ACAGCAGGCACAGACAGGCAGT#GCTGCCTGCGCCAAGCCTGGGTCTGGGCCATTCTGCTGA#.((((((((.((((((((((((#)))))))............))))).)))....))))).	380	419	1	-27	1	0.0004564	0.4	0.6	1.34	-5	20	39	7	CDS
mmu-miR-214-3p	NM_009058	Ralgds	ACAGCAGGCACAGACAGGCAGT#ACACCCTGTCGTGTGAGCTGG#.((((..((((.((((((..((#))..))))))))))..)))).	2105	2126	0.84615	-23	0	2.80E-05	0.4	1.8	2.59	-11	16	21	10	CDS
mmu-miR-214-3p	NM_009070	Rnps1	ACAGCAGGCACAGACAGGCAGT#ACTGCTCTTCTGTTCGGCCGACCATGCCCTGCTGA#.(((((((((((((..((((((#))))))..)))))............))).))))).	1554	1589	0.84615	-24	1	0.0006179	0.5	-0	-0.6	-5	19	35	6	3UTR
mmu-miR-214-3p	NM_009070	Rnps1	ACAGCAGGCACAGACAGGCAGT#ACTGCTACTGCTGTGTTGGCA#...((.((((((((.((((((((#)))))..)))))))))).)).	991	1012	0.84615	-21	0	7.37E-05	0.4	2.5	3.49	-9	17	21	10	CDS
mmu-miR-214-3p	NM_009154	Sema5a	ACAGCAGGCACAGACAGGCAGT#GCTGCCTCTGCTGTCTGTGTCTTCA#...(.((((((((((((((((#)))))))).....)))))))))).).	1600	1625	0.92308	-26	0	0.0107424	0.5	2.8	3.39	-11	18	25	10	CDS
mmu-miR-214-3p	NM_009154	Sema5a	ACAGCAGGCACAGACAGGCAGT#ACTGCTTGTCTACACCTACTGC#.(((.(((...((((((((((#)))))))))))...))).))).	3615	3637	1	-24	0	0.0291547	0.4	1.9	3.41	-13	17	22	11	CDS
mmu-miR-214-3p	NM_009196	Slc16a1	ACAGCAGGCACAGACAGGCAGT#ACAGCCTGTCACCTGCTGG#.(((((((....((((((.((#)).))))))).))))))).	2326	2345	0.84615	-25	1	0.0004704	0.5	0	0.49	-12	16	19	7	3UTR
mmu-miR-214-3p	NM_009196	Slc16a1	ACAGCAGGCACAGACAGGCAGT#GCTGATTTTGTCTCAAGTCCTTTCAGTTCACATAGCCGTGGCTGCTGG#.((((((.((((((((((((#))))..)))))))).......................))).)))))).	4055	4103	0.84615	-22	1	4.35E-05	0.5	0.1	0.02	-6	20	48	7	3UTR
mmu-miR-214-3p	NM_009196	Slc16a1	ACAGCAGGCACAGACAGGCAGT#TTGTCTGTCTGGTTGCGGCTT#..(((..((((((((((((((.#))))))))))))..))).))).	486	507	0.92308	-22	0	0.002831	0.5	2.2	3.36	-8	17	21	11	CDS

mmu-miR-214-3p	NM_009208	Slc4a3	ACAGCAGGCACAGACAGGCAGT#CTGCGGGATGCTCTGCACTCCCAGTGTGTGGCTGCTGTG#(((((((.((((.((.((.(((#.))).)).)).)))).))))))).	2310	2349	1	-27	1	1.92E-06	0.4	2.6	3.37	-8	18	24	7	CDS
mmu-miR-214-3p	NM_009208	Slc4a3	ACAGCAGGCACAGACAGGCAGT#GCTGCCACTGTGCGCTCTGTC#((((.((((((((...((((((#)))))).)))))).)))))).	3342	3363	0.84615	-25	0	5.14E-05	0.4	4	3.33	-10	18	21	6	CDS
mmu-miR-214-3p	NM_009211	Smarcc1	ACAGCAGGCACAGACAGGCAGT#CTGCTTGTCTGTAACCTGAGGTCTGTA#((((((((.((((((((((((.#))))))))))))..))))....)))).	5256	5283	1	-27	1	0.0105261	0.6	1	1.43	-5	20	27	12	3UTR
mmu-miR-214-3p	NM_009211	Smarcc1	ACAGCAGGCACAGACAGGCAGT#ACTGCTTGCCGGCGAAACCTGACTGGAGATGTGTGTGCTGTG#((((((.(((((..((((((((#))))))))....................))))).)))))).	1614	1656	0.84615	-26	0	0.0001517	0.5	4.1	4.52	-8	19	42	8	CDS
mmu-miR-214-3p	NM_009211	Smarcc1	ACAGCAGGCACAGACAGGCAGT#CTGGTTGTCTGTGCAGAAGATTTGCA#...(((((((((((((.(((.#))).))))))))))).......)))).	4145	4171	1	-24	0	6.92E-05	0.5	1.3	0.17	-7	17	26	10	3UTR
mmu-miR-214-3p	NM_009261	Strbp	ACAGCAGGCACAGACAGGCAGT#CTGTCTGTCCGTCTGACTGTC#((((((((...(((((((((.#))))))))).))))).)))).	6266	6287	0.96154	-25	1	4.56E-05	0.6	-0	-0.1	-11	18	21	9	3UTR
mmu-miR-214-3p	NM_009261	Strbp	ACAGCAGGCACAGACAGGCAGT#CTGCCTGCCCCTGCCTCCCATGTGCTGG#.(((((((((..(.(((((((.#))))))).)..))))).......)))).	14872	####	0.84615	-24	1	2.02E-05	0.4	3.7	4.42	-11	17	28	7	3UTR
mmu-miR-214-3p	NM_009274	Srpk2	ACAGCAGGCACAGACAGGCAGT#GCTGACCTGGGAAATGCTTGTTGG#.(((((((((....((((((((#)))).))))).....))))))))).	1773	1797	0.92308	-24	1	0.0004488	0.6	0.1	0.22	-12	17	24	9	CDS
mmu-miR-214-3p	NM_009274	Srpk2	ACAGCAGGCACAGACAGGCAGT#CTGCTTGTTTGTCCATATTCCCTGCA#...(((((.((((((((((((.#))))))))))))........))))).	4689	4715	1	-24	0	0.1139983	0.6	3.6	3.68	-8	17	26	12	3UTR
mmu-miR-214-3p	NM_009295	Stxbp1	ACAGCAGGCACAGACAGGCAGT#CCTGATCTGTGTCCATACTGTTGTA#(((((((((((((((((....#)))).))))))).....))))))).	2543	2568	0.92308	-23	1	0.0176683	0.5	4.1	4.23	-4	18	25	7	3UTR
mmu-miR-214-3p	NM_009335	Tfap2c	ACAGCAGGCACAGACAGGCAGT#ACTGGACCTCTAAGCCTGTCCGTGCTCCTTAAATAATTGTTGTG#(((((((((((.(((((((...#.))))))).))))).........)))))).	2748	2792	1	-26	1	0.1536174	0.7	0.7	1	-7	18	32	7	3UTR

mmu-miR-214-3p	NM_009366	Tsc22d1	ACAGCAGGCACAGACAGGCAGT#ACTGGCTGCTGCTGCTGCTGTC#((((((.((((((.(((.((((#)))).)))))).))))))))).	658	680	0.84615	-26	0	2.61E-05	0.5	0.6	1.37	-6	19	22	9	3UTR
mmu-miR-214-3p	NM_009373	Tgm2	ACAGCAGGCACAGACAGGCAGT#GCTGCCAACAGCTACCTGCTGG#.(((((((........((((((#))))))........))))))).	1799	1821	0.84615	-23	1	9.58E-06	0.4	2.1	1.92	-15	13	22	7	CDS
mmu-miR-214-3p	NM_009373	Tgm2	ACAGCAGGCACAGACAGGCAGT#ACTCGAGAGTGTCGTCTCCTGCTCTGTGCCCGCACTGTCAGCTACAACGGGGTGCTGG#.(((((((((((((((((.((.#.)))))).))))))))).......................))))).	1595	1653	0.84615	-26	1	1.15E-06	0.4	1.6	2.16	-4	19	44	8	CDS
mmu-miR-214-3p	NM_009380	Thrb	ACAGCAGGCACAGACAGGCAGT#GCTGCCTGAGTTCTACTTTGTACTCTGCAGCCTGG#....(((((.((((((((((((#))))))))).............)))))..))))).	3287	3322	0.92308	-24	0	4.30E-05	0.6	0.2	0.3	-11	17	35	8	3UTR
mmu-miR-214-3p	NM_009380	Thrb	ACAGCAGGCACAGACAGGCAGT#GCCTGTACCTGGCCTCCTGC#.(((.((((.((((((((((...#))))))..))))))).))).	3722	3742	0.92308	-24	0	4.63E-05	0.5	0.3	-0.2	-9	16	20	7	3UTR
mmu-miR-214-3p	NM_009382	Thy1	ACAGCAGGCACAGACAGGCAGT#TTGCCCCTCTGTGCCAGCCCCTCTTGCTGTC#((((((((((((((..((((((.#)))))..)))))))))).........)	1468	1499	0.84615	-28	1	0.0003932	0.4	0.1	0.07	-5	19	31	8	3UTR
mmu-miR-214-3p	NM_009389	Tle3	ACAGCAGGCACAGACAGGCAGT#GCTCGGCCCCCTTCGCCTGTCTGTGTGTGGTA#..(.(((.((((((((((((...#.)))))))))))).)).).	42	74	1	-28	0	7.50E-06	0.4	1.4	2.46	-10	15	19	12	5UTR
mmu-miR-214-3p	NM_009389	Tle3	ACAGCAGGCACAGACAGGCAGT#GCTCGCCGTGGATTTTGCCTGCTGTA#((((((((((.(..((((((((#))).))).))..)..))))))))))).	3863	3889	0.92308	-26	1	0.0093044	0.5	2.7	0.75	-9	19	26	10	3UTR
mmu-miR-214-3p	NM_009389	Tle3	ACAGCAGGCACAGACAGGCAGT#TGCCAAAGTCTGTTTTTCCTGCTGC#.((((((((.((((((.((((..#))))...))))))....)))))))).	2832	2857	0.80769	-25	1	0.0167808	0.5	4.8	4.37	-5	17	25	7	CDS
mmu-miR-214-3p	NM_009422	Traf2	ACAGCAGGCACAGACAGGCAGT#GCTGCCTGTGTCTATGAAGGCCTGTA#...((((((..(.(((((((((#))))))))).)........)))))).	317	343	0.84615	-24	0	0.0089769	0.5	-0	-0	-12	16	26	9	CDS
mmu-miR-214-3p	NM_009422	Traf2	ACAGCAGGCACAGACAGGCAGT#TTGAGATGTCTGTGCTGAGTGGTGTTCATCTGGCTCCTGCTGTG#((((((((((((((((..((((.#)))...))))))))))).....................))))))).	2150	2194	0.88462	-24	1	0.0011057	0.5	0.1	0.92	-4	19	44	9	3UTR

mmu-miR-214-3p	NM_009424	Traf6	ACAGCAGGCACAGACAGGCAGT#ACTGCCTGTGCTGGTGCCAGTAGAGAAGTTACCGCTCTGGTGTTGA#.(((((((((((((((((((((#))))))))).))).))))......................))))).	2433	2479	1	-27	1	0.0002848	0.5	0.4	0.64	-4	21	46	9	3UTR
mmu-miR-214-3p	NM_009424	Traf6	ACAGCAGGCACAGACAGGCAGT#CTGCCTGACACAGGAGAAAAGGGTGGCCTGTTGG#.((((((((.((..(((((((.#))))))).................))))))))).	5574	5608	0.84615	-25	1	0.0001757	0.4	-0	-0.1	-7	17	34	10	3UTR
mmu-miR-214-3p	NM_009424	Traf6	ACAGCAGGCACAGACAGGCAGT#TGTGTGTCTGTGTATGCC#...(((.(((((((((.((((.#))).))))))))).))).	206	224	1	-24	0	0.0043719	0.5	0	-0.1	-8	15	18	9	5UTR
mmu-miR-214-3p	NM_009424	Traf6	ACAGCAGGCACAGACAGGCAGT#ACTGCCATGGAACCTTTCTTGTCTGTCCCGTGCA#...(((((.(((((((((((((#))))))............)))))))).)).))).	2732	2766	0.84615	-23	0	0.0012549	0.5	-0	0.21	-8	18	34	7	3UTR
mmu-miR-214-3p	NM_009443	Tgoln1	ACAGCAGGCACAGACAGGCAGT#GTTTGTTTTCCTGCTGTG#((((((((...((((((((...#)))))))).)))))))).	4151	4169	1	-22	1	0.0008261	0.6	-0	-0.2	-9	16	18	8	3UTR
mmu-miR-214-3p	NM_009451	Tubb4a	ACAGCAGGCACAGACAGGCAGT#GCTGCGTGTGACCCGAGACACGGTCGCTACCTGACTGTGGCTGCTGTC#(((((((.(((((.((((.(((#.))).)))).))))).))))))).	987	1035	1	-30	1	2.01E-05	0.4	4.5	4.27	-7	19	24	7	CDS
mmu-miR-214-3p	NM_009451	Tubb4a	ACAGCAGGCACAGACAGGCAGT#CTGTCTGTGCATCAGCTGG#.((((..((((((((((.....#))))))))))....)))).	645	664	1	-25	0	8.43E-06	0.5	3.2	1.94	-7	14	19	10	CDS
mmu-miR-214-3p	NM_009470	Umod	ACAGCAGGCACAGACAGGCAGT#ACTGTCTGTTGTGCTGGGCA#...((.((((((.((((((((((#))))))))))))))))..)).	2425	2445	0.92308	-24	0	2.09E-05	0.5	0.1	-0	-10	17	20	15	3UTR
mmu-miR-214-3p	NM_009470	Umod	ACAGCAGGCACAGACAGGCAGT#GCTCCTGAGCATCTGGCTGCTGTT#((((((.((.((((((((.(((#)))))))....)))))))))))).	2067	2091	1	-21	0	0.0008576	0.5	1.5	1.3	-7	19	24	12	CDS
mmu-miR-214-3p	NM_009500	Vav2	ACAGCAGGCACAGACAGGCAGT#CTGCCTGTCTTTTCTGCTGC#.(((((((...((((((((((.#))))))))))..))))))).	4142	4162	0.92308	-29	1	0.0213826	0.5	5.1	3.8	-9	17	20	10	3UTR

mmu-miR-214-3p	NM_009521	Wnt3	ACAGCAGGCACAGACAGGCAGT#GCTCTTGGCCTGGTGGGAAGGACAAGACCATGGCGAGATTCTGTGTCCAAGCTGC#.((((.((((((((((((...#.))))).........................)))))))...)))).	1522	1577	0.80769	-23	0	0.0151601	0.5	0.5	0.88	-6	17	49	8	3UTR
mmu-miR-214-3p	NM_009521	Wnt3	ACAGCAGGCACAGACAGGCAGT#GCTTCTGTCTAGGGTCTGCTA#..(((((((.(((((((((.(((#))))))))))).).))))))).	2468	2489	1	-22	1	0.0124699	0.5	0.2	-0.2	-6	18	21	10	3UTR
mmu-miR-214-3p	NM_009595	Abl2	ACAGCAGGCACAGACAGGCAGT#ACTGCTGAGTCTCTTGGCTGCCTGCTT#..((((((((((((.((((((#))))))..))))....).)))))))).	5994	6021	1	-25	1	0.0025318	0.5	1	0.64	-5	19	27	8	3UTR
mmu-miR-214-3p	NM_009595	Abl2	ACAGCAGGCACAGACAGGCAGT#GCTTGTTGGTGGCTGTCTGTA#(((((((.(((.(((((((...#))))))).))).))).)))).	4551	4572	0.92308	-25	1	9.60E-06	0.4	0.6	0.21	-10	17	21	7	3UTR
mmu-miR-214-3p	NM_009595	Abl2	ACAGCAGGCACAGACAGGCAGT#GCTGCTTGTACCTTTCCCTCCTTGCAGCCCTCTGGCTGGCTGTA#(((((.(((.(((((((((((#))))))))).....................))))))).))))).	4772	4816	0.92308	-24	0	8.40E-05	0.4	-0	-0.3	-7	20	44	8	3UTR
mmu-miR-214-3p	NM_009595	Abl2	ACAGCAGGCACAGACAGGCAGT#GCTGCTGGTGTGCCTGG#....((((((((....((((((#))))))..)))))))).	3727	3744	0.84615	-24	0	2.99E-06	0.5	1	3.56	-13	14	17	8	CDS
mmu-miR-214-3p	NM_009630	Adora2a	ACAGCAGGCACAGACAGGCAGT#GCTGCTGGGTCTGTGGTCGATCCTGCTGTG#((((((((((((((.((((((#))))))..))))))).....)))))	2220	2250	0.92308	-32	1	0.0002226	0.3	0.6	0.3	-4	21	30	8	3UTR
mmu-miR-214-3p	NM_009670	Ank3	ACAGCAGGCACAGACAGGCAGT#GCTGTCTAATCCCCAGTCACAGGGGCCCTGTCTGTGCCTCCA#...(.((((((((((((....#.))))))))))))).).	4952	4994	0.92308	-26	0	0.0015804	0.5	0.2	0.57	-11	14	17	13	3UTR
mmu-miR-214-3p	NM_009670	Ank3	ACAGCAGGCACAGACAGGCAGT#CCTGTGTGCCTGTCGG#.(.((((((((..((((((....#))))))))))))))).).	3171	3187	1	-21	0	1.19E-05	0.5	3.1	2.09	-10	14	16	13	CDS
mmu-miR-214-3p	NM_009671	Ankfy1	ACAGCAGGCACAGACAGGCAGT#GCTGACATGTCTGTGCCAGATGA#.((...((((((((((.(((((#)))).).))))))))))...)).	2057	2080	0.92308	-24	0	0.0023693	0.5	4.5	3.24	-13	17	23	10	CDS
mmu-miR-214-3p	NM_009678	Ap1m2	ACAGCAGGCACAGACAGGCAGT#GCTGCAGACTTTTAAGAGCTGTGTGTGTGTGTTTGTG#...(((((((((.(((.(((((#.))))).))).)	1396	1433	1	-25	0	0.0027594	0.5	3.2	3.34	-8	17	21	9	3UTR

mmu-miR-214-3p	NM_009678	Ap1m2	ACAGCAGGCACAGACAGGCAGT#GCTGAAGGTGTTTCTGTCTGGGATGCCAGAGCTGC#.((((.((((((((((((((((#))))........))))))))...))))...)))).	681	716	0.84615	-23	0	8.33E-06	0.4	3.7	2.9	-6	20	35	8	CDS
mmu-miR-214-3p	NM_009682	Ap3s2	ACAGCAGGCACAGACAGGCAGT#CTGTCTGTCTGATGGTATGCTC#..((((..((((((((((((.#))))))))))).))...)))).	2055	2077	1	-24	0	0.0001603	0.5	0	0.04	-9	17	22	11	3UTR
mmu-miR-214-3p	NM_009682	Ap3s2	ACAGCAGGCACAGACAGGCAGT#CTGGCCTGGCCTGGCCTGTA#...((((((.(((.(((((((.#))).)))))..))))))))).	4272	4292	0.92308	-24	0	2.84E-07	0.4	-0	-0.1	-9	16	20	9	3UTR
mmu-miR-214-3p	NM_009722	Atp2a2	ACAGCAGGCACAGACAGGCAGT#ACTGCTCTCACGTGCCTGGTGG#.((.(((((((.....((((((#)))))).....))))))).)).	1866	1888	0.84615	-24	0	4.73E-05	0.5	4	3.58	-13	15	22	7	CDS
mmu-miR-214-3p	NM_009727	Atp8a1	ACAGCAGGCACAGACAGGCAGT#ATTGCCATGTCTCTTGTGTGTTCTGTG#((((.(.(((((((((((((((#)))))).)))))...)))).).)))).	1136	1163	0.84615	-23	0	0.0133841	0.5	0.6	0.6	-10	20	27	6	CDS
mmu-miR-214-3p	NM_009727	Atp8a1	ACAGCAGGCACAGACAGGCAGT#GCTGCGTGGATAGTGTCTAATCTGTG#((((.(((((((..((.(((((#))))).))..).)))))))...)))).	7899	7925	0.84615	-22	0	0.0006857	0.5	1	1.15	-10	18	26	6	3UTR
mmu-miR-214-3p	NM_009727	Atp8a1	ACAGCAGGCACAGACAGGCAGT#ACTGCTTTTCTGTCGTTTGTC#...(((((((((((.(((((((#))))))).))))).)))))).	4368	4389	1	-22	0	0.1259908	0.6	1	0.45	-7	18	21	7	3UTR
mmu-miR-214-3p	NM_009741	Bcl2	ACAGCAGGCACAGACAGGCAGT#CTGCTGGCCTGTGCCTGTGGTC#((.((((((((((.(.(((((.#))))).).)))))))))).)).	2451	2473	1	-27	0	8.20E-06	0.5	0.8	0.97	-7	18	22	10	3UTR
mmu-miR-214-3p	NM_009741	Bcl2	ACAGCAGGCACAGACAGGCAGT#GCTTGCCTGTTCCCTCCGCTGTGCTGTTGG#.(((((.((((((((((((((#))).)))))))........))))))))))	4067	4097	0.92308	-25	0	0.0001391	0.3	0.1	0.42	-5	20	30	11	3UTR
mmu-miR-214-3p	NM_009741	Bcl2	ACAGCAGGCACAGACAGGCAGT#TTGCTTTGTGTGGCCTGTTGG#.((((((((.(((.((((((((.#))))))).)).)))))))))).	4336	4357	1	-25	1	8.61E-07	0.5	1.7	1.65	-6	18	21	10	3UTR
mmu-miR-214-3p	NM_009741	Bcl2	ACAGCAGGCACAGACAGGCAGT#GCTGCTCCTTGGGCACCCGCGGCCCCAGCGCGTCCGTGCCTGCA#...((((((((.(((..((...#.))..)))).)))))))).	298	342	0.92308	-25	0	0.0004201	0.4	0.2	0.64	-10	13	18	8	5UTR
mmu-miR-214-3p	NM_009753	Bicd1	ACAGCAGGCACAGACAGGCAGT#TGTGTGTGTGTGTGTGTGTGTG#((((((.(((((.(((.(((..#))).))).))))).))).)))).	3888	3910	0.84615	-22	0	0.6004242	0.5	0.2	0.87	-7	17	22	5	3UTR

mmu-miR-214-3p	NM_009754	Bcl2l11	ACAGCAGGCACAGACAGGCAGT#ACTGCGGTGGCTGTGTTTGCC#...(((((((((.((.(((((#)))))..)).)))))))))).	1471	1492	0.92308	-24	0	8.07E-06	0.4	-0	-0.6	-7	17	21	10	3UTR
mmu-miR-214-3p	NM_009760	Bnip3	ACAGCAGGCACAGACAGGCAGT#CTGTTACTGTCTCATCTGCTGG#.(((((((...(((((((((.#)))).))))))..))))))).	596	618	1	-22	1	0.033548	0.5	0.5	-0.1	-9	17	22	7	CDS
mmu-miR-214-3p	NM_009760	Bnip3	ACAGCAGGCACAGACAGGCAGT#CTGCCTCCTTGACATGCTGTATATATCCTGTTGTA#((((((((.((((..((((((.#))))))..........))))......)))))))).	1531	1566	0.84615	-20	1	0.0206926	0.6	1.1	2.37	-7	18	35	8	3UTR
mmu-miR-214-3p	NM_009781	Cacna1c	ACAGCAGGCACAGACAGGCAGT#CCTGTCATGTGCCATGCCTGTT#((((((((((((((((((....#)))))).)))))).))).))).	12964	####	0.96154	-26	1	0.0031596	0.5	2.4	3.11	-4	18	22	6	3UTR
mmu-miR-214-3p	NM_009781	Cacna1c	ACAGCAGGCACAGACAGGCAGT#CCTGCTCTGTGTCTGCC#...((((((((((((((....#)))).))))))))))).	7511	7528	0.92308	-26	0	0.0055044	0.6	2.3	1.24	-7	15	17	11	3UTR
mmu-miR-214-3p	NM_009781	Cacna1c	ACAGCAGGCACAGACAGGCAGT#CTGCCTGCTGCGCCTTCTC#..((.((((.(((.((((((.#))))))))))).)))).)).	13138	####	0.84615	-26	0	1.86E-05	0.4	-0	0.92	-11	16	19	10	3UTR
mmu-miR-214-3p	NM_009784	Cacna2d1	ACAGCAGGCACAGACAGGCAGT#GCAGGGAGGGCACTGATCTTCGATCGCGAAGATGGCTGCTGGCTGCCTGCTGG#.((((((((((((.(((.((..#.)).))))))..))))))))).	292	345	0.90385	-28	1	4.67E-06	0.4	0.9	0.9	-5	17	22	9	5UTR
mmu-miR-214-3p	NM_009792	Camk2a	ACAGCAGGCACAGACAGGCAGT#GCTGCCTGCAAATGCTGCTCTTTCTCACGCTGTGGGCCCTGGTGC#.((.(((((((((.((((((((#))))))))...................)))))...)))).)).	282	327	0.84615	-24	0	7.81E-06	0.5	1.2	0.99	-8	19	45	8	5UTR
mmu-miR-214-3p	NM_009792	Camk2a	ACAGCAGGCACAGACAGGCAGT#GCTGCCTCTCCGTGCTTTTGG#.(((.((((((.((.(((((((#))))))).)).))))))))).	2641	2662	0.92308	-23	0	0.0175643	0.5	1.5	1.25	-12	18	21	9	3UTR
mmu-miR-214-3p	NM_009792	Camk2a	ACAGCAGGCACAGACAGGCAGT#CTGCGTCCTTGCTTCGCAGAGATCCGCTCTTTGTCCGTGGAATGTGGCTGCTGG#.((((((.((((((((((.(((#.))).))))))).......)))).)))))).	914	968	0.84615	-23	1	0.0008487	0.5	0.5	1.81	-6	19	30	6	3UTR
mmu-miR-214-3p	NM_009797	Capza1	ACAGCAGGCACAGACAGGCAGT#CTGTCCTTTCTGTCCTGTC#...(((((.(((((.((((((.#))).)))).)))))))))).	3045	3064	0.95385	-21	0	0.1337057	0.6	3	2.9	-8	16	19	10	3UTR

mmu-miR-214-3p	NM_009797	Capza1	ACAGCAGGCACAGACAGGCAGT#GGTGTGTGTGTGTGTGTGTG#...(((.(((((.(((.(((.(#).))).))).))))).))).	2654	2674	0.84615	-21	0	0.0047918	0.5	4.8	3.17	-9	15	20	5	3UTR
mmu-miR-214-3p	NM_009819	Ctnna2	ACAGCAGGCACAGACAGGCAGT#GCTGACTTGTCTGCCTCTGG#.(((.((((..((((((((((((#)))).))))))))))))))).	3201	3221	1	-23	0	0.0017291	0.6	3.6	4.16	-12	18	20	14	3UTR
mmu-miR-214-3p	NM_009857	Cd8a	ACAGCAGGCACAGACAGGCAGT#GCTTTCTGTCGCTGAACCTGCTGC#.(((((((((..((((((.(((#))).))))))..))..))))))).	145	169	0.84615	-23	1	0.0034098	0.4	0.7	0.06	-10	18	24	7	CDS
mmu-miR-214-3p	NM_009857	Cd8a	ACAGCAGGCACAGACAGGCAGT#GCTGACTGTCAAAACTGCTGC#.((((((.....(((((.((((#)))).)))))....)))))).	2765	2786	0.84615	-21	1	0.0002509	0.5	0.4	-0	-15	15	21	6	3UTR
mmu-miR-214-3p	NM_009857	Cd8a	ACAGCAGGCACAGACAGGCAGT#CTGGGTCCCTGTGTCTGCGTGG#.((((((((((((.....(((.#))).....)))))))))).)).	1550	1572	0.84615	-21	1	1.24E-06	0.4	0	0.03	-6	15	22	10	3UTR
mmu-miR-214-3p	NM_009871	Cdk5r1	ACAGCAGGCACAGACAGGCAGT#GCTGACCTGTCTGTACCTCTC#..((.(((.(((((((((((((#)))).))))))))).))))).	1163	1184	1	-25	0	0.0093294	0.5	4.4	4.38	-10	18	21	9	CDS
mmu-miR-214-3p	NM_009872	Cdk5r2	ACAGCAGGCACAGACAGGCAGT#GTCTGTCTGCGCCTCCA#...(.((((.(((((((((...#))))))))).)))).).	1430	1447	0.92308	-24	0	0.2137127	0.4	0.5	0.18	-12	14	17	9	3UTR
mmu-miR-214-3p	NM_009872	Cdk5r2	ACAGCAGGCACAGACAGGCAGT#CTGCTTGTGTCGTCCTGCA#...(((((.(((.((((((((.#))))))))).).))))))).	1754	1773	1	-23	0	0.021339	0.4	0	0.17	-9	16	19	8	3UTR
mmu-miR-214-3p	NM_009873	Cdk6	ACAGCAGGCACAGACAGGCAGT#CTGCAGCTGCTGGGCCTGCTT#..(((((((.(((.(((((((.#))))..)))))).))))))).	1671	1692	1	-25	1	1.16E-05	0.5	2.2	1.59	-7	17	21	7	3UTR
mmu-miR-214-3p	NM_009873	Cdk6	ACAGCAGGCACAGACAGGCAGT#AGTGCTTGTTCTGTGTGTGTA#...(((.(((((((((((((.(#).))))))).)))))).))).	2572	2593	0.92308	-24	0	0.0058614	0.5	0.4	0.15	-8	17	21	7	3UTR
mmu-miR-214-3p	NM_009873	Cdk6	ACAGCAGGCACAGACAGGCAGT#GCTGCCTGGTGCAAGGTGTG#(((.(..((((...((((((((#)))))))))))))..).))).	6143	6163	0.84615	-23	0	9.54E-05	0.5	0.3	0.07	-16	16	20	12	3UTR
mmu-miR-214-3p	NM_009873	Cdk6	ACAGCAGGCACAGACAGGCAGT#ACTGCCTGGCTAACGACAGATGGCCCAGCTGC#.((((.(((.((..((((((((#)))))))).........))	2393	2425	0.84615	-23	0	0.0004176	0.4	-0	0.16	-9	17	32	8	3UTR

mmu-miR-214-3p	NM_009890	Ch25h	ACAGCAGGCACAGACAGGCAGT#GCTCCTAAGTCACGTCCTGATCTGCCTGCTGC#.((((((((..(((((((..((#.))..)))).))))))))))).	390	422	1	-24	1	0.0030347	0.4	0.9	0.53	-8	17	22	11	CDS
mmu-miR-214-3p	NM_009904	Clgn	ACAGCAGGCACAGACAGGCAGT#CTGGCTGTGGATGTGCCGTGGCTGA#.((((.((((((.((((.(((.#))).))))...))))))...)))).	3864	3889	0.92308	-25	0	1.92E-05	0.5	0.3	-0.1	-7	17	25	6	3UTR
mmu-miR-214-3p	NM_009904	Clgn	ACAGCAGGCACAGACAGGCAGT#GCTGTAGGTTTGCTCTGTTGTA#((((((((..((((((..(((((#)))))..))))).)))))))).	2496	2518	0.84615	-20	1	0.0016458	0.6	1.3	0.58	-6	18	22	8	3UTR
mmu-miR-214-3p	NM_009906	Tpp1	ACAGCAGGCACAGACAGGCAGT#GCTGAACTCCTGTTGCCTGCTGC#.(((((((((...((((((((((#))))....))))))))))))))).	1824	1847	1	-26	1	0.003582	0.5	1.6	2.32	-11	18	23	14	3UTR
mmu-miR-214-3p	NM_009924	Cnr2	ACAGCAGGCACAGACAGGCAGT#GCCTTCTGTTCCATGCTGTG#((((((((.(((((.(((((...#))))))))).)).))))))).	1320	1340	1	-25	1	0.0020866	0.5	2	2.04	-6	17	20	9	CDS
mmu-miR-214-3p	NM_009926	Col11a2	ACAGCAGGCACAGACAGGCAGT#GCCTGTCTGCTTCATGGGCTAGGCCTGTC#...((((((.(((((((((...#)))))))))).............)))))).	5448	5477	0.84615	-25	0	2.10E-05	0.4	3.9	1.44	-8	15	29	9	CDS
mmu-miR-214-3p	NM_009926	Col11a2	ACAGCAGGCACAGACAGGCAGT#GCCTCCTGGCCTGCCTGG#.((((((((.(((..((((...#)))).))))))))).)).	4635	4653	0.92308	-23	1	6.20E-06	0.3	3.8	3.1	-7	15	18	9	CDS
mmu-miR-214-3p	NM_009937	Colq	ACAGCAGGCACAGACAGGCAGT#GCTGCCTCTGGGCTGTTGG#.(((((((.(.((((..((((((#))))))))))).)))))).	2617	2636	0.92308	-25	1	0.0002815	0.5	0.9	1.91	-8	17	19	11	3UTR
mmu-miR-214-3p	NM_009937	Colq	ACAGCAGGCACAGACAGGCAGT#GCTGGCTGTAATGCCTTATCTCTTCTGGAAAGACGCTGG#.(((((((((...((((.((((#)))).)))))..)))))).................)))).	1779	1818	0.84615	-21	1	0.0015633	0.5	1.4	2.09	-11	17	39	5	3UTR
mmu-miR-214-3p	NM_009952	Creb1	ACAGCAGGCACAGACAGGCAGT#TTGTCCTGCTGAGCTTGCTGG#.((((((((.(((.(((((((.#))).))))))).)))))))).	2958	2979	0.92308	-26	1	8.12E-05	0.5	1	1.29	-6	18	21	8	3UTR
mmu-miR-214-3p	NM_009952	Creb1	ACAGCAGGCACAGACAGGCAGT#GCTGCCTGTGGCCCGGGCGGCTGG#.(((((.(.(....(((((((((#)))))))))......).).)))).	61	85	1	-24	0	5.03E-07	0.3	2.5	2.76	-17	15	24	9	5UTR
mmu-miR-214-3p	NM_009952	Creb1	ACAGCAGGCACAGACAGGCAGT#CTGTCTGTGCTAGCA#...((.(((((((((((.....#)))))))))))).)).	2998	3013	0.92308	-23	0	0.0003018	0.6	0.6	1.05	-8	13	15	11	3UTR

mmu-miR-214-3p	NM_009963	Cry2	ACAGCAGGCACAGACAGGCAGT#TGTGTGTGTGTGTGTGCTGTT#((((((.(((((.(((.(((..#))).))).))))).)))))).	3119	3140	1	-28	0	0.128208	0.6	4.5	3.47	-7	17	21	6	3UTR
mmu-miR-214-3p	NM_009963	Cry2	ACAGCAGGCACAGACAGGCAGT#GCGTCTGTTTGTAGTCCGGGGACAGCCAGCTGA#.((((.(((((((((((((.((#)))))))))))))............))).)))).	312	345	0.84615	-22	0	6.54E-06	0.5	0.3	0.04	-6	19	33	12	CDS
mmu-miR-214-3p	NM_009982	Ctsc	ACAGCAGGCACAGACAGGCAGT#ATTGCTTGCTGTGCAAGCA#...((..((((((.((((((((#))))))))))))))..)).	2080	2099	0.92308	-24	0	7.43E-08	0.5	0	-0	-11	16	19	14	3UTR
mmu-miR-214-3p	NM_009982	Ctsc	ACAGCAGGCACAGACAGGCAGT#CTACCTGTCTCTGCCTCCTA#..((.(((((.(((((((.((.#)).)))))))).))))).)).	3087	3107	0.84615	-22	0	0.0574472	0.7	0	0.03	-12	16	20	7	3UTR
mmu-miR-214-3p	NM_009982	Ctsc	ACAGCAGGCACAGACAGGCAGT#ATGGTCTGTTTGTGTTAGATAACTGCTT#..((((((((((((((((.((#)).)))))))))))).......))))).	4425	4453	0.92308	-22	1	0.0294184	0.6	0	0.02	-5	19	28	12	3UTR
mmu-miR-214-3p	NM_009988	Cxadr	ACAGCAGGCACAGACAGGCAGT#GCTGCTGGCCCTTGTGCTCATCGGGGCCATCCTCTTCTGCTGTC#((((((((((((...((((((#))))))).....))))))..................))))))).	898	942	0.92308	-22	1	0.0112644	0.4	1.8	2.84	-6	19	44	7	CDS
mmu-miR-214-3p	NM_009988	Cxadr	ACAGCAGGCACAGACAGGCAGT#GCCGTGATGTCCTGTTGTC#((((((((.(((....((()...#)))....))))))))))).	1404	1423	0.92308	-21	1	0.0249772	0.5	0.2	-0	-8	14	19	11	3UTR
mmu-miR-214-3p	NM_009988	Cxadr	ACAGCAGGCACAGACAGGCAGT#GCGCGCCTACTGTGCTTCGTGCTC#..(((((((((((..((((.((#))..)))).)))))))...)))).	164	188	0.84615	-20	1	0.000928	0.4	0.5	0.93	-7	17	24	7	CDS
mmu-miR-214-3p	NM_010091	Dvl1	ACAGCAGGCACAGACAGGCAGT#GCTGAGTCTGTGCTCCTGCTGTG#(((((((((((((((...((((#)))).))))))))..)))))))).	2452	2475	0.84615	-28	1	0.0006406	0.3	-0	1.04	-4	19	23	8	3UTR
mmu-miR-214-3p	NM_010101	S1pr3	ACAGCAGGCACAGACAGGCAGT#CTGCTTGCTGTGGCCAGACTGTG#(((((.(((((((.(((((((.#)))))))))))).)).).)))).	1698	1721	0.92308	-25	0	0.0021744	0.5	0.3	-0.1	-7	19	23	12	3UTR
mmu-miR-214-3p	NM_010101	S1pr3	ACAGCAGGCACAGACAGGCAGT#ACTGCAGTCTGTCTGCTGC#.((((((..((((((..((((((#))))).))))))))))).	2442	2461	0.92308	-25	1	5.93E-06	0.5	-0	-0.4	-7	17	19	12	3UTR
mmu-miR-214-3p	NM_010101	S1pr3	ACAGCAGGCACAGACAGGCAGT#TTGGCTCTCTGCGACCTGCTGG#.((((((((.((((.((.(((.#))).)).)))).).))))))).	714	736	1	-25	1	3.39E-05	0.5	3.4	3.3	-6	17	22	7	CDS

mmu-miR-214-3p	NM_010101	S1pr3	ACAGCAGGCACAGACAGGCAGT#GCTGCTTGTCCCCTTTGCTGTC#((((((((....((((((((((#))))))))))...)))))))).	2547	2569	0.92308	-24	1	0.0140499	0.5	-0	-0.1	-12	18	22	10	3UTR
mmu-miR-214-3p	NM_010131	Emx1	ACAGCAGGCACAGACAGGCAGT#ATTGCTTGCTGCTGGCCAAGGCTCCAGGGGGCCCATGCTGG#.((((((((.(((.((((((((#))))))))))...)))..................))))).	817	858	0.92308	-21	1	0.0001956	0.3	0.5	1.45	-6	19	41	11	3UTR
mmu-miR-214-3p	NM_010142	Ephb2	ACAGCAGGCACAGACAGGCAGT#GCTGCTCTGGGAGAGTGTGCCTGG#....((((((((..((((((((#))))).)))......)))))))).	6311	6335	0.92308	-25	0	0.0002628	0.4	0.3	-0	-12	16	24	8	3UTR
mmu-miR-214-3p	NM_010142	Ephb2	ACAGCAGGCACAGACAGGCAGT#GCCTGGCTCCCCCTGCTGG#.(((((((...((.(((((...#))))).))...))))))).	9374	9393	0.84615	-24	1	6.01E-06	0.4	3.7	3.71	-10	14	19	7	3UTR
mmu-miR-214-3p	NM_010192	Fem1a	ACAGCAGGCACAGACAGGCAGT#GCTGCCGCTGCTGCAGAAGCTGCTGG#.((((((((((((.(.((((((#)))))))))).)))).....)))))).	160	186	0.84615	-25	1	4.56E-06	0.3	3.1	2.72	-5	19	26	10	CDS
mmu-miR-214-3p	NM_010192	Fem1a	ACAGCAGGCACAGACAGGCAGT#CTGCCTCAGTTTTCCTGCCTGCA#...(((((((.((((((((((.#))))))..))))...))))))).	5946	5969	0.84615	-25	0	0.0030859	0.5	0.3	-0.1	-9	17	23	7	3UTR
mmu-miR-214-3p	NM_010192	Fem1a	ACAGCAGGCACAGACAGGCAGT#GCTGCCCATGGTGCCTCCTGA#.(((.((((((.....((((((#))))))....)))))).))).	6087	6108	0.84615	-24	0	7.93E-05	0.5	-0	-0	-14	15	21	6	3UTR
mmu-miR-214-3p	NM_010192	Fem1a	ACAGCAGGCACAGACAGGCAGT#AGTGCCTGTCAAGCATGCGTGGTGG#.((.(((.(((..((((((((.(#).))))))))).....))).)).)).	3382	3407	0.84615	-23	0	4.45E-05	0.4	0	0.04	-15	16	25	8	3UTR
mmu-miR-214-3p	NM_010201	Fgf14	ACAGCAGGCACAGACAGGCAGT#GCTGCTCGCTCCACTGTCTGTGACCTTTAGCA#...((((((((((((((((((#)))))........)))))))))).)	1243	1275	1	-24	0	9.42E-05	0.5	4.1	2.51	-7	19	32	9	3UTR
mmu-miR-214-3p	NM_010201	Fgf14	ACAGCAGGCACAGACAGGCAGT#TGCTTGTCTTATGCTGTC#((((((.....(((((((((..#)))))))))..)))))).	876	894	0.92308	-20	0	0.0010639	0.5	3.2	2.22	-13	15	18	9	3UTR
mmu-miR-214-3p	NM_010201	Fgf14	ACAGCAGGCACAGACAGGCAGT#CCTTGCCTGCTGA#.(((((((((.....(((....#)))))))))))).	1080	1093	0.92308	-18	1	0.036681	0.6	0.1	2.29	-13	12	13	12	3UTR
mmu-miR-214-3p	NM_010250	Gabra1	ACAGCAGGCACAGACAGGCAGT#TGCCTAGGTTTGGGTCTGTA#...((((((.((((((((((..#)))))..))))).)))))).	4226	4246	0.92308	-21	0	0.0004177	0.6	3.9	3.5	-8	16	20	6	3UTR

mmu-miR-214-3p	NM_010250	Gabra1	ACAGCAGGCACAGACAGGCAGT#ATTGTTTGATTTGGTCCTGCC#...(((((..((((((((((((#)))))))).))))..))))).	4020	4041	1	-20	0	0.0004845	0.7	0.6	1.36	-9	17	21	8	3UTR
mmu-miR-214-3p	NM_010251	Gabra4	ACAGCAGGCACAGACAGGCAGT#GCTGTGCTCCGGGGTCAGCCTCGCCCTCCTGCACTTCCTGTGCCTGGCGG#.(..(((((((((..((((((.#.)))).))..)))))))))..).	531	581	0.92308	-26	0	2.03E-06	0.4	0.3	0.46	-10	16	23	9	CDS
mmu-miR-214-3p	NM_010251	Gabra4	ACAGCAGGCACAGACAGGCAGT#GCTGTCTGTTTTGCTTTTGTATTTTCGGCTCTTATTGAGTTTGCTGCTGTC#((((((((((.(((((((((((#))))))))))))))).........................))))	1468	1519	1	-25	1	0.0013845	0.6	0.1	0.44	-7	21	51	14	CDS
mmu-miR-214-3p	NM_010265	Gcnt1	ACAGCAGGCACAGACAGGCAGT#GCCTGCTGGGACATGTCATCGCTGGATGCCTGGTGC#.((.(((((((((.(((((...#)))))))))..................)))))).)).	90	126	1	-24	0	3.02E-05	0.5	0.2	0.16	-8	16	36	8	5UTR
mmu-miR-214-3p	NM_010265	Gcnt1	ACAGCAGGCACAGACAGGCAGT#GCTTGTACAGTGTCTGTTT#..(((((((((.(((((((...#)))))).).))))))))).	3900	3919	0.92308	-21	1	0.0033711	0.5	2	1.78	-8	16	19	9	3UTR
mmu-miR-214-3p	NM_010271	Gpd1	ACAGCAGGCACAGACAGGCAGT#ACTAAGCCCCAGTGGTGCCTGCTC#..(((((((((..((.((((((#)))..)))...)).))))))))).	1289	1313	0.92308	-22	1	0.0003473	0.4	0.3	-0.2	-10	17	24	9	3UTR
mmu-miR-214-3p	NM_010275	Gdnf	ACAGCAGGCACAGACAGGCAGT#GCTGCTTGTCTTCTCGCCCCTGCCTTCTGTC#((((.(((((.(((((((((((#)))))))))))).........))))).)	75	106	0.92308	-26	0	6.85E-05	0.4	0.5	0.59	-11	20	31	11	5UTR
mmu-miR-214-3p	NM_010275	Gdnf	ACAGCAGGCACAGACAGGCAGT#TGGCTGTCTGCCTGGTGTT#(((.(((((..((((((.((..#)).)))))))))))).))).	1112	1131	0.92308	-24	0	6.24E-06	0.4	3.8	2.9	-11	16	19	11	CDS
mmu-miR-214-3p	NM_010275	Gdnf	ACAGCAGGCACAGACAGGCAGT#ATTGCCCCCTGGGGCTGCTGTG#(((((((.(.(((...((((((#)))))))..))).).))))))).	240	262	0.92308	-24	1	7.70E-07	0.4	-0	0.62	-9	17	22	7	5UTR
mmu-miR-214-3p	NM_010301	Gna11	ACAGCAGGCACAGACAGGCAGT#CTGTGTTCCCCTGCTGCAGGCAGGGCCTGCTGTT#(((((((((...(.(..(((((#.)))))..))...))))))	1896	1930	0.84615	-25	1	1.17E-05	0.4	0.1	0.31	-12	16	23	9	3UTR
mmu-miR-214-3p	NM_010301	Gna11	ACAGCAGGCACAGACAGGCAGT#CTGTCTGTCTGTCAGATTTCTTTACTTTTTGCACGTGCGTTCTGG#.(((.(.((((((((((((((.#))))))))))))).......................)).).))).	2304	2349	0.92308	-23	0	0.0029384	0.5	4	3.72	-9	18	45	12	3UTR

mmu-miR-214-3p	NM_010367	Magi1	ACAGCAGGCACAGACAGGCAGT#ACCGCCATGCTGTGCCTTGCA#...((((((((((.(((((.((#)).))).))))))))))).)).	1007	1028	0.92308	-24	0	7.70E-05	0.4	3.4	2.48	-7	17	21	10	CDS
mmu-miR-214-3p	NM_010367	Magi1	ACAGCAGGCACAGACAGGCAGT#GTCTTGTGCCTGGTGTA#(((.((((((((((.......#)))).))))))).))).	4976	4993	0.92308	-24	0	0.1128847	0.8	2.6	3.83	-7	14	17	7	3UTR
mmu-miR-214-3p	NM_010368	Gusb	ACAGCAGGCACAGACAGGCAGT#GCCTTGGCTTTGTGAACTCTTGAAAGCCTGCTGTG#(((((((((((((((((...#)))).).).)))))).............))))))))).	2386	2421	1	-23	1	0.0085617	0.6	0.6	1.12	-4	19	35	9	3UTR
mmu-miR-214-3p	NM_010415	Hbegf	ACAGCAGGCACAGACAGGCAGT#GCTGCCTCCCCGTCTCCGCCAGGCTCGGGACCATGAAGCTGCTGC#.((((((((..((((((((((((#)))))))....)))))..))).................))))).	260	305	0.94872	-23	1	0.0091994	0.4	0.5	1.06	-8	19	45	7	5UTR
mmu-miR-214-3p	NM_010415	Hbegf	ACAGCAGGCACAGACAGGCAGT#ACTGCAGTCTTGCCTAGGTGA#.((.((((((.(((..(((((#))))).)))))))))).).)).	1826	1847	0.92308	-22	0	0.0013265	0.5	2.1	1.62	-10	17	21	9	3UTR
mmu-miR-214-3p	NM_010546	Ikbkb	ACAGCAGGCACAGACAGGCAGT#ACTCCTGGGTGCCTGTCAGGTGTGTGTGGATATTGCTGC#.((((((((((.((((((((.(#.).))))))))..))))).........)))))).	4001	4040	1	-25	1	0.0011001	0.6	2.8	3.83	-7	19	33	8	3UTR
mmu-miR-214-3p	NM_010546	Ikbkb	ACAGCAGGCACAGACAGGCAGT#ACAGCCTGTCTGCTTTGGACTCCTGGGGGAGGAAGCTCTGCCCTGCC#...(((((((((((((((.((#)).)))))))))))........................)))).))).	3604	3651	0.84615	-24	0	0.0024968	0.4	3.1	2.8	-7	18	47	9	3UTR
mmu-miR-214-3p	NM_010593	Jup	ACAGCAGGCACAGACAGGCAGT#GCCCGCTGTCTGTGCCCTGCG#...(((((((((((((...((#))...))))))))))))).))).	1521	1542	0.84615	-26	0	0.0005946	0.4	-0	-0.2	-7	16	21	11	CDS
mmu-miR-214-3p	NM_010593	Jup	ACAGCAGGCACAGACAGGCAGT#GCTGGCTTCAGCCTCTGCTGTCCTGCTGTG#((((((((.(((((.((((...#.)))).).))))))))))))).	3521	3551	1	-25	1	1.00E-04	0.5	2.9	3.23	-6	17	21	12	3UTR
mmu-miR-214-3p	NM_010598	Kcnab2	ACAGCAGGCACAGACAGGCAGT#ACTGCCTGCCTGTGAACCACACCTCAGAGCTAAGCTCCTGCTGG#.((((((((((((.((((((((#)))))))).)))))......................))))))).	2932	2976	0.81538	-32	1	2.06E-06	0.5	3.3	3.88	-5	20	44	8	3UTR
mmu-miR-214-3p	NM_010611	Kcnq2	ACAGCAGGCACAGACAGGCAGT#GCTATGTCTGTTGTCTGCTGTA#(((((((((((((((((((...#))).))))))).)))))))))).	4371	4393	1	-30	1	0.009891	0.5	0.1	-0.2	-4	19	22	9	3UTR

mmu-miR-214-3p	NM_010611	Kcnq2	ACAGCAGGCACAGACAGGCAGT#GCCTGTACACATGCCCGCCTTGTTGTGCCAGGCTGTG#(((((.(((((((((((((...#)))))))................)))))))..))))).	6038	6075	0.84615	-27	0	7.10E-07	0.4	1.4	2.71	-6	18	37	7	3UTR
mmu-miR-214-3p	NM_010611	Kcnq2	ACAGCAGGCACAGACAGGCAGT#ATTGCCTCCATTGCTGTGCTGGCTGCTGG#.(((((((((((..(((((((#)))))))......))))))...))))))).	725	754	0.84615	-26	1	0.0001418	0.4	-0	0.24	-5	19	29	7	CDS
mmu-miR-214-3p	NM_010626	Kif7	ACAGCAGGCACAGACAGGCAGT#ACTGCCTGTTTTGCTTCTA#..((.(((((.(((((((((((#))))))))))))))))))).	4119	4138	1	-23	0	0.0005563	0.5	3.6	3.09	-12	18	19	18	3UTR
mmu-miR-214-3p	NM_010626	Kif7	ACAGCAGGCACAGACAGGCAGT#TGACCTGCTGGACTGCCTGGTGC#.((.((((((((((.((((((..#)).)))))))...)))))).)).	429	452	0.92308	-23	0	0.0002488	0.5	-1	1.04	-8	17	23	7	CDS
mmu-miR-214-3p	NM_010700	Ldlr	ACAGCAGGCACAGACAGGCAGT#GCTGCCTGTCTGCCCCAGGTTCTGTTTTATATATTTATTGTCTGGGGACAGAAAAGGCTACTGGCTGTG#((((((((((((((((((((#)))))))))))))........................)))))....................	2951	3020	0.98077	-27	1	0.0009377	0.6	0.6	-0.3	-4	22	69	12	3UTR
mmu-miR-214-3p	NM_010700	Ldlr	ACAGCAGGCACAGACAGGCAGT#GCCTGTCCTGTGCGTTGCTT#..((((((((((((((((...#))))))).))))).))))).	4422	4442	0.92308	-28	1	0.0299477	0.5	-0	0.43	-5	17	20	7	3UTR
mmu-miR-214-3p	NM_010700	Ldlr	ACAGCAGGCACAGACAGGCAGT#TTGCCAGTACCTGTGCCTGCC#...((((((((((((.(((((.#))))).))..)))))))))).	2232	2253	1	-27	0	0.0007182	0.3	3.6	2.01	-7	17	21	10	CDS
mmu-miR-214-3p	NM_010718	Limk2	ACAGCAGGCACAGACAGGCAGT#CTGCCATGGGTGCTCCCTGCTGA#.(((((((..((..(((((((.#))))).))..))...))))))).	389	412	1	-24	1	1.62E-06	0.4	-0	-0	-8	16	23	7	CDS
mmu-miR-214-3p	NM_010763	Man1a2	ACAGCAGGCACAGACAGGCAGT#CTGCCTCGTCTGTTTTTGCTTAGCTGTG#(((((((((((((((((((((.#)))))).))))))....)))).))))).	6119	6147	0.80769	-29	1	0.0181973	0.5	4	3.86	-4	21	28	6	3UTR
mmu-miR-214-3p	NM_010763	Man1a2	ACAGCAGGCACAGACAGGCAGT#GCCTGTCGGTGTCGTCCAGCTGG#.((((.(((((.(((((((...#))))))).))))).....)))).	5199	5222	0.84615	-26	0	0.000359	0.4	0.6	1.73	-9	16	23	7	3UTR
mmu-miR-214-3p	NM_010780	Cma1	ACAGCAGGCACAGACAGGCAGT#GCAGGTTTTGTGTGCCATGCGATCTGGCCTGTCTGTAGTTCCTGCTGA#.(((((((((((((((((((((.#.))).))))))))).)..))))))).	934	982	1	-31	1	0.0020812	0.4	0.7	0.34	-4	20	26	9	3UTR

mmu-miR-214-3p	NM_010804	Mllt10	ACAGCAGGCACAGACAGGCAGT#CTGCTGTTCTGTGCTTCACTGGCTGCTGC#.(((((((((((((..(((((.#)))))..)))))))........)))))).	3371	3400	1	-27	1	2.01E-05	0.5	1.4	1.58	-5	18	29	7	3UTR
mmu-miR-214-3p	NM_010804	Mllt10	ACAGCAGGCACAGACAGGCAGT#GCTGCGTCATCTGTAACCCCTGCTGC#.(((((((.(((((.(.(((((#))))).)..)))))....))))))).	1974	2000	0.92308	-24	1	0.0222278	0.5	1.5	2.14	-6	18	26	7	CDS
mmu-miR-214-3p	NM_010804	Mllt10	ACAGCAGGCACAGACAGGCAGT#CTGTCTTCTGTGACACTGCA#...(((((((((((.((((((.#)))))))))))).).))))).	4544	4564	1	-23	0	0.0004269	0.5	2.7	3.16	-7	17	20	12	3UTR
mmu-miR-214-3p	NM_010806	Afdn	ACAGCAGGCACAGACAGGCAGT#ACTGTGTTACTGTGCCTCCTCTGA#.(((.(((((((..(.(((((#))))).)..))))))))...))).	6482	6506	0.92308	-23	0	0.0328463	0.6	-0	0.48	-10	17	24	8	3UTR
mmu-miR-214-3p	NM_010886	Ndufa4	ACAGCAGGCACAGACAGGCAGT#CTGCGTGTAGGTAGGCCTGTGCCGCA#...((.((((((((((.((((.#)))).)))........))))))))).	56	82	0.92308	-23	0	6.42E-06	0.4	0.8	1.55	-8	16	26	9	5UTR
mmu-miR-214-3p	NM_010899	Nfatc2	ACAGCAGGCACAGACAGGCAGT#GCTGCCCTCTACCAGAGAAGCAAGAGCCTGAGTCCCGGCCTGCTGG#.((((((((...((((((((...#.))))..)))...)))))))).	2424	2470	1	-26	1	2.90E-06	0.4	0.5	0.02	-11	15	22	8	CDS
mmu-miR-214-3p	NM_010914	Nfyb	ACAGCAGGCACAGACAGGCAGT#ACTGTCTGTGGCTGTTCAGGCAGCTTCAGTTACTATCACGCCTGTTA#..((((((((((((((((((((#))))))))..)))).......................))))))).	1478	1525	0.92308	-23	1	0.0567644	0.5	3.7	3.3	-5	20	47	9	3UTR
mmu-miR-214-3p	NM_010914	Nfyb	ACAGCAGGCACAGACAGGCAGT#GCCTTCTGGTTTGCTGTC#((((((((((.((((.((((...#))))))))))))))))).	1748	1766	0.92308	-25	1	1.91E-07	0.5	3.7	3.67	-6	17	18	17	3UTR
mmu-miR-214-3p	NM_010928	Notch2	ACAGCAGGCACAGACAGGCAGT#CTGCCTGTGTCCGGAGGGAAAGGCGGGTCTCCTGTGCCATCTGG#.(((..((((((((((((((.#))))))))......................)))))))..))).	1243	1287	0.84615	-27	0	0.0037389	0.4	3.2	3.36	-10	18	44	8	CDS
mmu-miR-214-3p	NM_010928	Notch2	ACAGCAGGCACAGACAGGCAGT#GCTGCTTGTGTGCTCCTGGCTGG#.(((((((..((.(((((((((#))))))))).))..)))).))).	2733	2756	1	-27	1	0.0004657	0.5	2.2	3.02	-7	18	23	9	CDS
mmu-miR-214-3p	NM_010928	Notch2	ACAGCAGGCACAGACAGGCAGT#GCTTCACCTGTCTGTGCATGCC#...(((.(((((((((((....#.))))))))))).))).	1590	1612	0.92308	-26	0	0.0001207	0.5	2.4	3.37	-8	14	17	11	CDS

mmu-miR-214-3p	NM_010937	Nras	ACAGCAGGCACAGACAGGCAGT#CTGCTTACAGACTTTTGCCTGATGCCTGCTGTT#((((((((((....(((((((.#.))))))).)))))))))).	2064	2097	1	-28	1	0.0023665	0.6	0.8	0.36	-12	17	20	10	3UTR
mmu-miR-214-3p	NM_010937	Nras	ACAGCAGGCACAGACAGGCAGT#TGTGTGTGTGTGTGTGCA#...(((.(((((.(((.(((..#))).))).))))).))).	3077	3095	0.84615	-23	0	0.0034139	0.5	-0	0.14	-9	14	18	5	3UTR
mmu-miR-214-3p	NM_011014	Sigmar1	ACAGCAGGCACAGACAGGCAGT#GCCTTTAGTGTGTGTGTGCA#...(((.(((((.((((((...#))))...)).))))).))).	1539	1559	0.87179	-22	0	6.49E-06	0.5	0.1	0.29	-9	14	20	5	3UTR
mmu-miR-214-3p	NM_011028	P2rx6	ACAGCAGGCACAGACAGGCAGT#CTGCCCCATGTCTGCTGCTGCTGG#.(((((.((((((((((((((.#)))))...)))))).)))))))).	1360	1384	0.92308	-28	0	0.0001158	0.4	-0	-0.3	-5	19	24	8	3UTR
mmu-miR-214-3p	NM_011028	P2rx6	ACAGCAGGCACAGACAGGCAGT#CCTTCCTCTGTGACCTGCTGC#.((((((((((((.(((....#)))...)))))).))))))).	1189	1210	1	-25	1	9.83E-08	0.4	2.5	1.07	-5	16	21	7	CDS
mmu-miR-214-3p	NM_011062	Pdpk1	ACAGCAGGCACAGACAGGCAGT#CTGTGTTGGGTGGGGCCTGCTGG#.(((((((.((..((.((((.#))))..))..))..)))))))).	5917	5940	0.84615	-24	1	1.94E-06	0.5	2.5	3.38	-7	16	23	8	3UTR
mmu-miR-214-3p	NM_011067	Per3	ACAGCAGGCACAGACAGGCAGT#GCTGCCTGTGACATTTCTTGGAGCTTTCTGGTATTGAGCAGTTCCTGACACGTGTTCCTGCTGTG#((((((((..((((((((((((#))))))))).................))))........................)))))))).	5234	5299	0.92308	-26	1	0.0001763	0.5	0.4	1.71	-6	20	65	8	3UTR
mmu-miR-214-3p	NM_011137	Pou2f1	ACAGCAGGCACAGACAGGCAGT#CTCCCTGTCTGTGGCTGTC#...((((.((((((((((.((.#)).)))))))))))).)))).	8041	8060	1	-27	0	9.14E-05	0.4	0.4	0.94	-9	16	19	10	3UTR
mmu-miR-214-3p	NM_011137	Pou2f1	ACAGCAGGCACAGACAGGCAGT#GCTGCCCTAGTGTGCAGGAGACAGTGTCCTGCTGG#.(((((((((((....((((((#))))))....)))))............)))))).	6689	6724	0.92308	-25	1	2.93E-05	0.4	0.2	0.48	-7	17	35	6	3UTR
mmu-miR-214-3p	NM_011137	Pou2f1	ACAGCAGGCACAGACAGGCAGT#GCTTCCTTCTACTTCCCATGGGAACAGCTGTGCCTGTTGC#.((((((((((((..(((.(((#))).)))..................)))))))))))).	3404	3444	0.92308	-24	1	0.0002413	0.5	-0	0.04	-5	18	40	12	3UTR
mmu-miR-214-3p	NM_011137	Pou2f1	ACAGCAGGCACAGACAGGCAGT#ACTGCTTGGAAGTGGCTGTCCTGTT#(((((((.(((...((((((((#))))))))...))).)))..)))).	11457	####	1	-23	1	6.72E-05	0.5	3.3	3.11	-12	18	25	8	3UTR

mmu-miR-214-3p	NM_011216	Ptpro	ACAGCAGGCACAGACAGGCAGT#CTGCCTCCAGGAGATATCTACAATCTGTCTGTCACGGCCTGCA#...((((((...(((((((((.#.)))))))))...)))))).	2493	2536	0.98077	-28	0	0.0031247	0.4	2.8	3.73	-14	15	20	9	CDS
mmu-miR-214-3p	NM_011216	Ptpro	ACAGCAGGCACAGACAGGCAGT#ACTGCTGTCGTGCTGAGCTGG#.((((.(((((.((((.(((((#))))))))))))))..)))).	2046	2067	0.92308	-26	0	8.52E-05	0.4	2	3.58	-9	18	21	14	CDS
mmu-miR-214-3p	NM_011221	Purb	ACAGCAGGCACAGACAGGCAGT#CCTGTCCTGTCCTGTTGG#.(((((((.(((((((((....#)))))).)))))))))).	6185	6203	1	-24	1	0.0004208	0.6	0.6	1.48	-5	16	18	10	3UTR
mmu-miR-214-3p	NM_011221	Purb	ACAGCAGGCACAGACAGGCAGT#GCTGTTTTCAATAATGTCCTGCTGTA#((((((((.(((((.(((((((#))))))))).....))))))))))).	3332	3358	0.92308	-21	1	0.0457951	0.6	1.1	1.52	-6	20	26	11	3UTR
mmu-miR-214-3p	NM_011277	Rnf2	ACAGCAGGCACAGACAGGCAGT#GCTAGCCTGGAACTTGCTGA#.(((((((......((((((((#))).)))))...))))))).	1644	1664	0.92308	-20	1	0.0005177	0.5	0.9	0.5	-14	15	20	7	3UTR
mmu-miR-214-3p	NM_011277	Rnf2	ACAGCAGGCACAGACAGGCAGT#TTGGTAATCTGTGCCA#......((((((((..(.(((.#))).)..)))))))).	2875	2891	0.84615	-17	0	0.0001597	0.7	4	3.35	-17	12	16	8	3UTR
mmu-miR-214-3p	NM_011354	Serf2	ACAGCAGGCACAGACAGGCAGT#GCTTCGTGTCCAACCCTCTTGCCCTCCGCCTGTGTGCCTGGAGC#.(..(((((((..((((((...#.)))))))))))))..).	424	468	0.84615	-24	0	2.28E-05	0.4	2.1	2.64	-13	14	18	13	3UTR
mmu-miR-214-3p	NM_011354	Serf2	ACAGCAGGCACAGACAGGCAGT#CTGCCCTGGTTTGTTCTGTGGTC#((.(((((.((((((.(((((.#)))))...))))))))))).)).	1210	1233	0.92308	-20	0	0.0083991	0.4	0.6	0.73	-7	18	23	11	3UTR
mmu-miR-214-3p	NM_011374	St8sia1	ACAGCAGGCACAGACAGGCAGT#ACTGCTCCTGTGCTCTGTC#...((((((((((...((((((#)))))).)))))).)))).	4205	4224	0.84615	-23	0	4.38E-05	0.5	0.3	-0.2	-8	16	19	6	3UTR
mmu-miR-214-3p	NM_011406	Slc8a1	ACAGCAGGCACAGACAGGCAGT#TGTCTGTCTGTCCTCTGG#.(((.(((.(((((((((((..#))))))))))))))))).	15137	####	1	-26	0	0.0009438	0.6	0.5	0.42	-9	17	18	17	3UTR
mmu-miR-214-3p	NM_011406	Slc8a1	ACAGCAGGCACAGACAGGCAGT#ACTGCCGTTGTGTTTGTTGC#.(((((((((((.(((.((((((#)))))))))))))))))))).	2606	2626	1	-25	1	0.0018778	0.5	0.7	0.42	-5	19	20	19	CDS
mmu-miR-214-3p	NM_011406	Slc8a1	ACAGCAGGCACAGACAGGCAGT#GCTGCTTGTCCATTTGCTCCGGCTGG#.((((.((((..((((((((((#))))))))))....))))...)))).	15273	####	0.92308	-25	0	0.0001054	0.4	-0	0.62	-12	18	26	10	3UTR

mmu-miR-214-3p	NM_011406	Slc8a1	ACAGCAGGCACAGACAGGCAGT#TTGTCTGTTGTGTGATGCTGTG#((((((.(((((.((((((((.#))))))))))))))..)))))).	6239	6261	0.88462	-25	0	0.0037416	0.5	0.1	0.02	-7	19	22	13	3UTR
mmu-miR-214-3p	NM_011447	Sox8	ACAGCAGGCACAGACAGGCAGT#ACTCCGTCAAGTGCCTGCTGA#.((((((((((.(((.((.(((#))))))))..)))))))))).	1645	1666	1	-26	1	5.79E-05	0.4	1.6	1.65	-7	18	21	10	3UTR
mmu-miR-214-3p	NM_011447	Sox8	ACAGCAGGCACAGACAGGCAGT#CTGCCTGCGTCGCTGTGTCGAGTGTA#(((...(((((((.(((((((.#))))))).....)))))))...))).	2614	2640	0.92308	-24	0	8.88E-05	0.5	3.3	2.63	-14	17	26	7	3UTR
mmu-miR-214-3p	NM_011469	Sprr2b	ACAGCAGGCACAGACAGGCAGT#ACTGTGTGCTTTCAGAGGAGCCTTCTCAGCCTGCCTGTT#(((((((((..(((.((((...#.)))))))..)))))).))).	621	660	0.92308	-22	1	0.0014076	0.5	-0	-0.3	-9	16	21	7	3UTR
mmu-miR-214-3p	NM_011471	Sprr2e	ACAGCAGGCACAGACAGGCAGT#GCTGTCTATCCTGTGCACCCAGAAGATGTTTTCCAAAACTGTCAATTGTTGTG#((((((((((((((.(((((((#))))))).)).)))))....................	545	598	0.84615	-21	1	0.0049475	0.6	-0	-0.2	-5	21	53	7	3UTR
mmu-miR-214-3p	NM_011499	Strap	ACAGCAGGCACAGACAGGCAGT#AGTGCCATAGCCTGCTGTT#(((((((((.......((((.(#).))))...))))))))).	1538	1557	1	-20	1	0.0067002	0.5	1.5	0.96	-14	14	19	9	3UTR
mmu-miR-214-3p	NM_011513	Med22	ACAGCAGGCACAGACAGGCAGT#CCTGTCCACTGCCTGCTGC#.(((((((((..((((((....#))))))...))))))))).	3308	3327	1	-27	1	0.004589	0.5	-0	-0.6	-10	15	19	9	3UTR
mmu-miR-214-3p	NM_011513	Med22	ACAGCAGGCACAGACAGGCAGT#GCTGCCTGCTGCCTGCC#...((((((..(((.((((((((#))))))))))))))))).	670	687	0.84615	-27	0	6.39E-06	0.3	1.5	2.11	-11	16	17	16	3UTR
mmu-miR-214-3p	NM_011546	Zeb1	ACAGCAGGCACAGACAGGCAGT#TTGCCTTCTGTGTGAGGACTGCC#...((((((((((((.((((((.#)))))))))))))).....)))).	1579	1602	1	-22	0	7.81E-05	0.5	2.8	2.07	-7	17	23	13	CDS
mmu-miR-214-3p	NM_011575	Tff3	ACAGCAGGCACAGACAGGCAGT#CCTGAAGCTTGCCTGCTGC#.(((((((((.((.((((....#))))...))))))))))).	15	34	1	-22	1	0.0006072	0.4	0.1	0.03	-7	15	19	11	5UTR
mmu-miR-214-3p	NM_011586	Myo18a	ACAGCAGGCACAGACAGGCAGT#TGCCTCCCCTCTGCTGGGGTGAGGGCTTTCTCTTCTCAGTGCCTGCTGTC#(((((((((((.((..(((((.#.)))))...................)).))))))))))).	6574	6624	0.84615	-26	1	0.0078529	0.6	1.9	1.02	-8	18	41	11	3UTR

mmu-miR-214-3p	NM_011586	Myo18a	ACAGCAGGCACAGACAGGCAGT#CTGCTTGTCCTTAGCACCCGAGGGGCTCCTGCTGTG#((((((((....(((((((((.#))))))))).................)))))))).	1404	1440	0.92308	-25	1	9.43E-06	0.5	2.8	3.33	-12	17	36	9	CDS
mmu-miR-214-3p	NM_011586	Myo18a	ACAGCAGGCACAGACAGGCAGT#GCTGCTGTCTGGGCCTAAGCCGG#.(.((((((.((((((.(((((#))))))))))).)))))..)).).	3553	3576	0.84615	-24	0	6.93E-06	0.4	2.5	3.37	-7	18	23	11	CDS
mmu-miR-214-3p	NM_011632	Traf3	ACAGCAGGCACAGACAGGCAGT#GCTGCCATCTGTCCTGGGTGTGCCTTCGCCCCAGTGCCTGCTGG#.((((((((((.((((((((((#))))))...))))...................)))))))))).	3903	3947	0.84615	-30	1	4.61E-06	0.3	1.5	1.46	-7	20	44	10	3UTR
mmu-miR-214-3p	NM_011699	Lin7c	ACAGCAGGCACAGACAGGCAGT#TCTGTCCTGCTGTT#((((((((.((((((........#)))))))))))))).	813	827	0.92308	-20	1	1.02E-05	0.6	1.7	1.96	-6	13	14	13	3UTR
mmu-miR-214-3p	NM_011719	Wnt9b	ACAGCAGGCACAGACAGGCAGT#CTGCCTGCTGGTGCTGCCTGCC#...((((((((((.((((((((.#))))))))))....)))))))).	87	109	0.84615	-29	0	4.07E-06	0.2	-0	-0.1	-7	17	22	10	CDS
mmu-miR-214-3p	NM_011740	Ywhaz	ACAGCAGGCACAGACAGGCAGT#TGCGTGTGGTGCCCCTGCTGC#.((((((((((..(((.(((..#))).))).)))))..))))).	409	430	0.84615	-24	1	0.0001542	0.4	0.4	1.01	-8	16	21	5	5UTR
mmu-miR-214-3p	NM_011740	Ywhaz	ACAGCAGGCACAGACAGGCAGT#TTGCTTGGTGTGCCCGGCTC#..(((.((((((..((((((((.#)))))))).))))))..))).	2038	2058	0.92308	-23	0	3.22E-05	0.5	1.5	1.14	-9	16	20	7	3UTR
mmu-miR-214-3p	NM_011785	Akt3	ACAGCAGGCACAGACAGGCAGT#GCTGCTACCGTCTGTACTGTCTACATCACGGTTCCCTTAGCTTGCTC#..(((((((((((((.((((((#))))))...))))))......................))))))).	4298	4345	0.84615	-23	1	0.0021892	0.5	2	1.88	-5	19	47	7	3UTR
mmu-miR-214-3p	NM_011785	Akt3	ACAGCAGGCACAGACAGGCAGT#GCTGCTGGCAGTGGCTGGCCGCTGG#.((((.(((.(((.((((((((#))))))).....)).)))))))))).	3286	3311	0.84615	-22	0	7.72E-07	0.4	0.7	0.63	-8	18	25	10	3UTR
mmu-miR-214-3p	NM_011785	Akt3	ACAGCAGGCACAGACAGGCAGT#GCTGCTTCTCTGTTTGTTGTTGTTGG#.(((((((..(((((((((((((#)))))....))))))))..)))))).	4732	4758	1	-22	1	0.0133866	0.6	1	0.19	-7	19	26	8	3UTR
mmu-miR-214-3p	NM_011794	Bpnt1	ACAGCAGGCACAGACAGGCAGT#ACTGTCCAAGTCATTTGTGCAGTCAACATTCCTGCTGA#.((((((((((((((.((((((#))))))...)))...)))))...........)))))).	1879	1917	0.84615	-22	1	0.0025219	0.5	0.5	0.35	-4	20	38	6	3UTR

mmu-miR-214-3p	NM_011812	Fbln5	ACAGCAGGCACAGACAGGCAGT#ACCGCTGTATGTGTCCTGCTGA#.(((((((((((.(((.((.((#)).))))).)))).))))))).	1357	1379	1	-23	1	0.0059948	0.5	0	-0.2	-5	18	22	7	CDS
mmu-miR-214-3p	NM_011812	Fbln5	ACAGCAGGCACAGACAGGCAGT#ACTGTGTGTGTGTGTTTTGTTT#..((((((((((.(((.(((((#))))).))).))))))).))).	3378	3400	1	-22	1	0.0529113	0.7	1.3	0.86	-6	18	22	7	3UTR
mmu-miR-214-3p	NM_011812	Fbln5	ACAGCAGGCACAGACAGGCAGT#GCTGCTTGTTTTACCCACTTGTTGG#.(((((((...((((((((((#))))))))))......))))))).	4351	4376	0.84615	-21	1	0.005225	0.6	3.5	3.92	-9	18	25	11	3UTR
mmu-miR-214-3p	NM_011828	Hs2st1	ACAGCAGGCACAGACAGGCAGT#GCTGCGTGTCCTCGTGGAACGTTTTCTTCTGCTGC#.((((((((((.((((.(((((#))))).))))...)))..........))))))).	5659	5694	0.84615	-25	1	0.0339263	0.5	2.7	3.11	-7	19	35	7	3UTR
mmu-miR-214-3p	NM_011828	Hs2st1	ACAGCAGGCACAGACAGGCAGT#CTGCTGTCACCTTCCTGCTGTG#((((((((....((((.((((.#))))))))).....)))))))).	2629	2651	0.92308	-24	1	0.0181166	0.4	0	0.16	-12	16	22	8	3UTR
mmu-miR-214-3p	NM_011828	Hs2st1	ACAGCAGGCACAGACAGGCAGT#GCTGCTTCTGTGTCCCCTGA#.(((..((((((((..((((((#))))))))))))))..))).	4475	4495	0.92308	-23	0	0.0001012	0.5	0.8	0.82	-11	17	20	14	3UTR
mmu-miR-214-3p	NM_011865	Pcbp1	ACAGCAGGCACAGACAGGCAGT#CTGCCTGGTCATGCTGG#.((((((((.....(((((((.#))))))))))).))))).	759	776	0.84615	-21	1	1.18E-05	0.4	2.9	2.71	-13	15	17	10	CDS
mmu-miR-214-3p	NM_011881	Grk1	ACAGCAGGCACAGACAGGCAGT#GCTGCCCTGTGCCTGGTTGTA#(((((((((((((...((((((#))))))))))))))).))))).	3989	4010	0.92308	-29	1	0.001217	0.4	0.1	0.01	-6	19	21	15	3UTR
mmu-miR-214-3p	NM_011881	Grk1	ACAGCAGGCACAGACAGGCAGT#CTGTCTGTCTGTCTTGCTT#..((((((.((((((((((((.#))))))))))))))))))).	6766	6785	1	-29	1	0.0601508	0.5	0.2	-0	-6	18	19	18	3UTR
mmu-miR-214-3p	NM_011881	Grk1	ACAGCAGGCACAGACAGGCAGT#CTGCCTGTCAGATGAAGGCGACTGGCAAGATGTATGCCTGCA#...((((((((.(((((((((.#))))))))).)......................))))))).	650	692	1	-27	0	0.0017167	0.5	1.9	3.69	-10	17	42	9	CDS
mmu-miR-214-3p	NM_011898	Spry4	ACAGCAGGCACAGACAGGCAGT#GCTGCCCTGTCTGCTGTGCTACCTGCCGG#.(((..((((((((.(((((((.#.))))))))))))))..))).	959	988	0.84615	-27	0	2.30E-06	0.3	1.3	0.08	-11	17	21	14	CDS
mmu-miR-214-3p	NM_011898	Spry4	ACAGCAGGCACAGACAGGCAGT#GCCGCCTCCGTCCATGCCTGTTGG#.((((((((((..((((((((.((#)).))))..)))..))))))))).	1551	1575	1	-26	1	0.0008034	0.4	0.3	0.19	-10	18	24	9	3UTR

mmu-miR-214-3p	NM_011898	Spry4	ACAGCAGGCACAGACAGGCAGT#GGTGGCTGTCAGTTGTGCCTGGAGC#.(..(((((((((((((.((.(#).)).)))))...))))))))..).	3810	3835	1	-24	0	2.77E-05	0.5	3.6	4.15	-8	17	25	8	3UTR
mmu-miR-214-3p	NM_011906	Tpra1	ACAGCAGGCACAGACAGGCAGT#GCCCCACCAGTGCCTGCTGC#.((((((((((.....(((...#)))......))))))))))).	494	514	0.92308	-25	1	0.0002779	0.5	2.4	2.36	-10	13	20	10	CDS
mmu-miR-214-3p	NM_011908	Ubl3	ACAGCAGGCACAGACAGGCAGT#CTGCACGTGTCTGTGGCGCCCAGTTCTCCTGTGTGTA#((((((((((((((((((((.#)))).).))))))))).........)))))).))).	1527	1564	0.92308	-23	1	6.25E-05	0.4	4.1	3.07	-4	21	37	8	3UTR
mmu-miR-214-3p	NM_011914	Nelfa	ACAGCAGGCACAGACAGGCAGT#AGTGCCATCTCTGCTGCTGA#.(((((.(((.(((..((((.(#).)))).))).))))))))).	1628	1648	0.92308	-19	0	0.0052231	0.5	3.8	2.91	-9	16	20	8	CDS
mmu-miR-214-3p	NM_011932	Dapp1	ACAGCAGGCACAGACAGGCAGT#ACAAATCAAAAGTAGATCGTGGTTGGGAGCTGTCTGCTGTCTGCTGA#.((((((..((((.((((((((#.)))))))))))))))))).	1064	1111	1	-26	1	1.82E-06	0.5	-0	-0	-8	18	20	18	3UTR
mmu-miR-214-3p	NM_011935	Esrrg	ACAGCAGGCACAGACAGGCAGT#GCTGCCTGTATACAAACTCTGTCTCAGAAGGAGCTGTG#((((((((((.(((((((((((#)))))))))......)).)))))).......))))).	2931	2969	1	-23	1	0.0002872	0.5	1.1	1.24	-7	21	38	9	3UTR
mmu-miR-214-3p	NM_011935	Esrrg	ACAGCAGGCACAGACAGGCAGT#ACTGTCGTCTTGTTCTCGTGGTGTTTGGATTCTTGTGGTGTGTGCTT#..((((.(((((((((.((((((#))))))))))).........................)))).))))).	3479	3526	0.92308	-20	0	0.0236703	0.6	3.3	3.95	-7	18	47	10	3UTR
mmu-miR-214-3p	NM_011942	Lypla2	ACAGCAGGCACAGACAGGCAGT#CTGCAGTGTATGTGTGGTAACACCATGTCTGTGCCCCTGCTC#..(((((((((((((((((....#.))).)))))))))))..))))).	96	138	1	-25	1	0.0048319	0.4	0.7	0.95	-5	16	21	10	5UTR
mmu-miR-214-3p	NM_011948	Map3k4	ACAGCAGGCACAGACAGGCAGT#GCTGCTCCTGTCGCCTGCTGA#.(((((((((((((..((((((#)))))).)))).))))))))).	4969	4990	0.92308	-29	1	0.000279	0.4	1.4	2.22	-6	18	21	8	3UTR
mmu-miR-214-3p	NM_011948	Map3k4	ACAGCAGGCACAGACAGGCAGT#GGTGCTCTGTCGGGTCCTGCTGA#.(((((((.(((.((((((((.(#).))).)))))..))))))))).	1855	1878	0.84615	-26	1	0.0002391	0.4	4.1	3.97	-8	18	23	9	CDS
mmu-miR-214-3p	NM_011948	Map3k4	ACAGCAGGCACAGACAGGCAGT#GCTGGTTGTCATGGAGTCTGCTC#..((((((((.((((((((.((((#)))).)))))).))..))))))).	2842	2865	0.92308	-24	1	0.0001485	0.5	4.1	3.57	-6	18	23	7	CDS

mmu-miR-214-3p	NM_011977	Slc27a1	ACAGCAGGCACAGACAGGCAGT#TTGGCTGCTGTGTCCTGCA#...((((((((((.(((.(((.#))).)))))))))).))))).	2433	2452	0.84615	-25	0	3.19E-07	0.4	-0	-0.3	-7	16	19	8	3UTR
mmu-miR-214-3p	NM_011977	Slc27a1	ACAGCAGGCACAGACAGGCAGT#CTGTGGAGCAAGTGGGGGCCCACATAGCTGCTGTCCCTGCTGA#.(((((((....((((.(((((#.)))))))))))))))).	2548	2591	0.84615	-23	1	3.94E-06	0.4	-0	-0.2	-12	16	18	16	3UTR
mmu-miR-214-3p	NM_012024	Ppp2r5e	ACAGCAGGCACAGACAGGCAGT#CCTGTCTGACCTCCCTGGGCAATCCTGCTGTC#((((((((..((((((((....#))))))))...............))	4374	4406	0.84615	-23	1	0.0003598	0.5	4.4	4.72	-6	16	32	8	3UTR
mmu-miR-214-3p	NM_012024	Ppp2r5e	ACAGCAGGCACAGACAGGCAGT#GCTTGTCTTTCTGCTAAGCTGTT#(((((.((((.((((((((...#))))))))...))))..))))).	2382	2405	0.92308	-23	0	0.0007739	0.7	0.9	1.01	-9	17	23	8	3UTR
mmu-miR-214-3p	NM_012024	Ppp2r5e	ACAGCAGGCACAGACAGGCAGT#TTGCTTGCTGTGGTCCCTCTGG#.(((.((((((((.((((((((.#))))))))))))))...)))))).	4021	4043	1	-22	0	1.47E-05	0.5	4.2	2.61	-9	18	22	12	3UTR
mmu-miR-214-3p	NM_012065	Pde6g	ACAGCAGGCACAGACAGGCAGT#TCTGTCCAGTGCTTGCCTGC#.((((((((((.((((((....#))))))..))))))))).)).	72	92	0.92308	-21	1	0.0001032	0.4	-0	-0.3	-7	16	20	8	5UTR
mmu-miR-214-3p	NM_013458	Add2	ACAGCAGGCACAGACAGGCAGT#CTGCCGGTCTCCCATAATGCCCTGCTGG#.(((((((((.((((.(((((.#))))).))))).......)))).))))).	932	960	0.84615	-25	1	0.000647	0.4	3.5	3.46	-7	18	28	5	CDS
mmu-miR-214-3p	NM_013458	Add2	ACAGCAGGCACAGACAGGCAGT#CTGTCTGTATTGTGCTTCTCACAGGCTGA#.(((((((((((((((((((((.#)))))))))).))))))))).......)))).	3097	3126	1	-25	1	0.000123	0.6	0.4	0.26	-4	20	29	8	3UTR
mmu-miR-214-3p	NM_013458	Add2	ACAGCAGGCACAGACAGGCAGT#CTGCTTGTGGGTTTCTCTGTGTGTGCCGTT#((.(((.((((((((((((((.#))))))))........))))))).))).)	7928	7958	1	-25	0	0.0626007	0.5	1	1.09	-7	19	30	7	3UTR
mmu-miR-214-3p	NM_013491	Clcn1	ACAGCAGGCACAGACAGGCAGT#ACGCCTGCTGCACTGCTTGCTA#..((((((((((((.(((((.((#)))))))))))...)))))))).	2406	2428	0.92308	-23	1	0.0003609	0.5	0.9	0.71	-6	18	22	10	CDS
mmu-miR-214-3p	NM_013543	H2-Ke6	ACAGCAGGCACAGACAGGCAGT#ACTGCCTGATGGAAGCCGGACTCTGCTCACTCCCCCACCACTGCCTGGCCCTCCTGCTGA#.(((((((......((((((((#.))))))))).....)))))	787	847	1	-27	1	0.0005815	0.4	0.2	1.09	-14	15	22	8	CDS

mmu-miR-214-3p	NM_013646	Rora	ACAGCAGGCACAGACAGGCAGT#CTGTCTGTCTGTGTGAATTCTTCACCGTCTCATCTTGTTGA#.(((((((((((((((((((.#)))))))))))))...................))))))).	6352	6393	1	-27	1	0.0019717	0.6	0.7	1.11	-4	20	41	13	3UTR
mmu-miR-214-3p	NM_013646	Rora	ACAGCAGGCACAGACAGGCAGT#TGTGTGTGTGTGTGTGCG#...(((.(((((.(((.(((..#))).))).))))).))).	7410	7428	0.84615	-23	0	0.0050871	0.4	0.4	0.09	-9	14	18	5	3UTR
mmu-miR-214-3p	NM_013660	Sema4d	ACAGCAGGCACAGACAGGCAGT#GCTCCAGTCTGTGCTGCTGG#.(((((.((((((((.((.(((#))))).))))))))))))).	345	365	0.96154	-27	0	2.56E-05	0.4	0	0	-6	18	20	13	5UTR
mmu-miR-214-3p	NM_013660	Sema4d	ACAGCAGGCACAGACAGGCAGT#ACTGTGTGTTAGCACGGGACCCCTACTGTGCCTGGAGC#.(..(((((((((((.(((((#))))).)))...............))))))))..).	1995	2033	0.92308	-24	0	0.0003036	0.4	0	0	-8	18	38	9	CDS
mmu-miR-214-3p	NM_013673	Sp100	ACAGCAGGCACAGACAGGCAGT#GCTGCCTGTTGTGAGCG#...((...((((.(((((((((#))))))))))))).)).	2397	2414	0.92308	-21	0	0.0001195	0.5	0.1	0.14	-14	15	17	13	3UTR
mmu-miR-214-3p	NM_013703	Vldlr	ACAGCAGGCACAGACAGGCAGT#TGTCTGTCTTTGCTCCTGCTA#..((((((((.((((((((((..#))))))))).)))..))))).	7262	7283	1	-24	1	0.0004164	0.6	0	0.11	-8	17	21	9	3UTR
mmu-miR-214-3p	NM_013703	Vldlr	ACAGCAGGCACAGACAGGCAGT#GCTGTCTTAGTACTGTCACTGTTGTA#(((((((..(((((((((((((#)))))))..)).))))..))))))).	8173	8199	0.92308	-23	1	0.0338413	0.5	0.2	0.24	-7	20	26	7	3UTR
mmu-miR-214-3p	NM_013703	Vldlr	ACAGCAGGCACAGACAGGCAGT#GCTGCCATGGTCTAGGGTGAGGGTGCTGTC#((((((..(((((((.((((((#))))))...))))...)))....))	7505	7535	0.92308	-23	0	0.0004878	0.5	0.2	0.02	-8	19	30	6	3UTR
mmu-miR-214-3p	NM_013718	Trappc3	ACAGCAGGCACAGACAGGCAGT#GCAGCCAAGAGGATGCCTGCTGG#.((((((((((.....(((.((#)).))).....).))))))))).	651	674	0.84615	-23	1	8.24E-06	0.4	0.4	1.71	-10	15	23	9	3UTR
mmu-miR-214-3p	NM_013757	Sytl4	ACAGCAGGCACAGACAGGCAGT#GCAGAGAAGAAATGCCTGTCAGTGACATCCAGCTGC#.((((.(((((.((((((((..#.)))))))).)))....)).))))).	3495	3531	1	-25	0	0.0005731	0.5	1.8	2.19	-9	17	25	8	3UTR
mmu-miR-214-3p	NM_013757	Sytl4	ACAGCAGGCACAGACAGGCAGT#TGTCTGGGTGTGCTGCTA#..((((.(((((..((((((..#))))))..))))))))).	3146	3164	0.92308	-23	0	0.0010361	0.6	1.5	2.93	-9	15	18	9	3UTR

mmu-miR-214-3p	NM_013790	Abcc5	ACAGCAGGCACAGACAGGCAGT#GCTTTCCTGTCTTGGTGTCAGCTGCTGTT#((((((((((((((((((.(((#)))..))))))))..))))...))))))).	5138	5167	0.84615	-25	1	0.0011847	0.4	3.2	2.99	-4	21	29	7	3UTR
mmu-miR-214-3p	NM_013814	Galnt1	ACAGCAGGCACAGACAGGCAGT#CTGCTGAATGTGCCTTGTGA#.((..(((((((....(((((.#)))))...)))))))..)).	2362	2382	1	-22	0	0.0073382	0.5	1.2	1.71	-14	14	20	7	3UTR
mmu-miR-214-3p	NM_013814	Galnt1	ACAGCAGGCACAGACAGGCAGT#CTGTCTGTCAGGTGATACTTGATGG#.((.((((((.(((((((((.#)))))))))..)))...)))).)).	3447	3472	0.84615	-21	0	3.65E-06	0.6	0.4	0.76	-10	18	25	9	3UTR
mmu-miR-214-3p	NM_013814	Galnt1	ACAGCAGGCACAGACAGGCAGT#ACTCTTGGATATGTTCCTGCTGC#.(((((((.(((..((((.(((#)))))))....))).))))))).	388	411	0.92308	-21	1	0.0007721	0.6	3.9	4.92	-7	17	23	7	CDS
mmu-miR-214-3p	NM_013814	Galnt1	ACAGCAGGCACAGACAGGCAGT#CTGCCTCGCCCTGCCTCCCTCCCTCCACACAGTTTCTTAACATGCTGTC#((((((((((..(..((((((.#))))))..)..)))))........................))))).	2884	2933	0.84615	-20	1	0.0343363	0.5	0.3	1.34	-12	17	49	6	3UTR
mmu-miR-214-3p	NM_013860	Limd1	ACAGCAGGCACAGACAGGCAGT#CTGGGAGGCTGGGTCTCCCTGCTGTGCCTCTGC#.(((.((((((((.((((((((#.))))......)))))))	3135	3168	0.84615	-26	0	0.0049847	0.4	0.2	-0	-9	19	27	15	3UTR
mmu-miR-214-3p	NM_013860	Limd1	ACAGCAGGCACAGACAGGCAGT#GCTGCCCGGTGTGTTTGCA#...(((((((((..(.((((((#)))))).).))))))))).	147	166	0.92308	-23	0	0.0080855	0.4	-0	-0.2	-9	16	19	9	5UTR
mmu-miR-214-3p	NM_013905	Heyl	ACAGCAGGCACAGACAGGCAGT#GCTGCCTGGTTTGCATCCGGCTCCCAGAGCCCAGACTGCTGC#.(((((((((....((((((((#))))))))...)))....................)))))).	1946	1988	0.84615	-23	1	3.61E-06	0.4	1	0.84	-12	17	42	8	3UTR
mmu-miR-214-3p	NM_013905	Heyl	ACAGCAGGCACAGACAGGCAGT#GCTTCTTGTTTCCTGCTGTG#((((((((...(((((((.(((#))).)))))))))))))))).	3798	3818	0.92308	-22	1	0.0005919	0.4	-0	0.02	-9	18	20	15	3UTR
mmu-miR-214-3p	NM_013905	Heyl	ACAGCAGGCACAGACAGGCAGT#GCCTGAGAGCCTGCTC#..(((((((.(...(((((...#))))).).))))))).	2081	2097	1	-22	1	2.77E-06	0.4	0.4	0.42	-12	13	16	7	3UTR
mmu-miR-214-3p	NM_015749	Tcn2	ACAGCAGGCACAGACAGGCAGT#ACTGCCAGGCATCCAGGGTCATGTTGGTGCCAGCTGTT#(((((.(((((.((((((((((#))))))..............)))).))))).))))).	1131	1169	0.84615	-27	0	0.0006268	0.4	1.7	0.96	-9	20	38	6	CDS

mmu-miR-214-3p	NM_015749	Tcn2	ACAGCAGGCACAGACAGGCAGT#ATGGCCTGCTGTGGCTCTGA#.(((.((.(((((.(((((.((#)).)))))))))))).))))).	1815	1835	0.84615	-23	0	1.90E-05	0.4	1.3	0.82	-11	17	20	10	3UTR
mmu-miR-214-3p	NM_015759	Fgd3	ACAGCAGGCACAGACAGGCAGT#GCTGTCACTCTGGGAAAGGCAGAGCTGTCTGTGGCCTGA#....((((((((((((((((((#)))))..................))))))))).)))).	2765	2804	0.96154	-24	0	7.32E-05	0.4	0.2	-0.3	-10	18	39	9	3UTR
mmu-miR-214-3p	NM_015774	Ero1a	ACAGCAGGCACAGACAGGCAGT#CTGTCTCTCTGCTGTGCTCACTTGGCTGCCTGTTGTC#(((((((((((((.((((((.#)))))).))))..............)))))))))).	3569	3606	0.84615	-26	1	0.0011739	0.5	4	3.19	-5	20	37	10	3UTR
mmu-miR-214-3p	NM_015780	Cfhr1	ACAGCAGGCACAGACAGGCAGT#GCTTTAGAAGATGGGGTTCTGTCGCCTGTTGC#.((((((((((((((((((...#)))).........)).))))).)	54	86	0.92308	-21	1	0.0032653	0.5	0.1	-0.1	-4	18	32	8	5UTR
mmu-miR-214-3p	NM_015790	Icosl	ACAGCAGGCACAGACAGGCAGT#GCCTGTCTGCTATGTGGCTGG#.((((..((((((((((((...#)))))))))...))).))))).	1983	2004	0.84615	-26	0	0.0001783	0.5	0.1	0.08	-7	16	21	9	3UTR
mmu-miR-214-3p	NM_015790	Icosl	ACAGCAGGCACAGACAGGCAGT#CTGGTCTTGGTCTGTTCTTGCTGC#.(((((((.((((((((((((.#))).)))..)))))).))))))).	242	266	0.92308	-23	1	0.0008557	0.5	-0	0.02	-5	19	24	7	CDS
mmu-miR-214-3p	NM_015790	Icosl	ACAGCAGGCACAGACAGGCAGT#GCTGCCTCTGCAGAGACTGAAGTCGGTGCAATGGTGG#.((.((.((((.((((((((((#)))))))).............))).)))))..)).)).	283	320	0.84615	-22	0	7.88E-07	0.4	-1	0.29	-12	18	37	7	CDS
mmu-miR-214-3p	NM_016700	Mapk8	ACAGCAGGCACAGACAGGCAGT#CTGCCTGGACTGTGATGTCTGTT#((((((..(((((.((((((((.#))))))))..))))).)).))))).	5437	5460	0.84615	-25	0	0.0002166	0.6	5.5	4.4	-9	18	23	7	3UTR
mmu-miR-214-3p	NM_016700	Mapk8	ACAGCAGGCACAGACAGGCAGT#GCTGGACCTCTGGGCTGCTGTA#((((((((.(.((((....((((#))))....))))).)))))))).	1453	1475	0.92308	-23	1	4.87E-08	0.4	0.8	-0	-8	16	22	7	3UTR
mmu-miR-214-3p	NM_016700	Mapk8	ACAGCAGGCACAGACAGGCAGT#CTGTCCTGGCTACGAGCCTGCTT#..((((((((..((.((((((((.#))).)))).))....)))))))).	2317	2340	1	-22	1	0.0001554	0.5	1.6	0.25	-10	16	23	7	3UTR
mmu-miR-214-3p	NM_016707	Bcl11a	ACAGCAGGCACAGACAGGCAGT#CTGCCGAGTCCTCCCAGCCTGGGCCTGCAGTG#((.((((((.(((((.(((((.#)))))..))).........)).))))))).)).	2776	2808	0.84615	-23	0	4.79E-05	0.6	2.5	2.25	-7	18	32	6	3UTR

mmu-miR-214-3p	NM_016713	Mink1	ACAGCAGGCACAGACAGGCAGT#GCTGCTTGCTGATAGCAATGGCTACACAAACCTGCCTGATGTG#(((.(((((((((.((((((((#)))))))))))....................)))))).)))).	2786	2829	1	-24	0	0.0011015	0.5	3.5	2.98	-8	20	43	11	CDS
mmu-miR-214-3p	NM_016713	Mink1	ACAGCAGGCACAGACAGGCAGT#GCTGCCTTCCCAGCCTCCCACGACCCTGACCCTGCTGC#.(((((((..(((..(((((((#)))))))...................)))..))))))).	2025	2063	0.84615	-23	1	0.1190285	0.3	2.9	2.39	-8	17	38	7	CDS
mmu-miR-214-3p	NM_016745	Atp2a3	ACAGCAGGCACAGACAGGCAGT#GCTGTGGCCCTGGCTGTGGCTGCTA#..((((((.(((((.((((((((#)))).....)))).))))).))))).	1079	1104	0.84615	-26	0	4.46E-06	0.4	3.3	3.94	-8	18	25	5	CDS
mmu-miR-214-3p	NM_016745	Atp2a3	ACAGCAGGCACAGACAGGCAGT#CTGCCTGTCATCCTGCTC#..((((((....(((((((((.#)))))))))..))))))).	3107	3125	0.92308	-26	1	0.0004777	0.4	3.4	2.93	-13	15	18	9	CDS
mmu-miR-214-3p	NM_016775	Dnajc5	ACAGCAGGCACAGACAGGCAGT#GCTGTTTGTTGTTTGTGGCCTCCTCACCTGCTGC#.((((((((((((((((((((#)))))...)))))))))))...........))))))).	547	581	1	-25	1	0.0004061	0.5	3	4.32	-4	21	34	9	CDS
mmu-miR-214-3p	NM_016782	Cntnap1	ACAGCAGGCACAGACAGGCAGT#GCTGCCTGTCTGGTGTCCGGTTC#..(((.(((((((((((((((#))))))))))))).)))))..))).	3766	3789	0.96154	-28	0	0.0003543	0.5	4.1	2.47	-7	19	23	12	CDS
mmu-miR-214-3p	NM_016789	Nptx2	ACAGCAGGCACAGACAGGCAGT#CTGCCTGTTGTTTTGGATCCTTGGTGTCTCATGTGTACTTCCTGTCTGTC#((((((((((((((((((((.#)))))))))).......................)))).....)))).))	1759	1809	0.92308	-24	1	0.0103161	0.5	0	0.22	-4	21	50	9	3UTR
mmu-miR-214-3p	NM_016804	Mtx2	ACAGCAGGCACAGACAGGCAGT#CTGCCTGTCAAAGTGGTGTGTA#...(((.(((((.(((((((((.#)))))))))...))).).))).	348	370	0.84615	-23	0	0.0039202	0.6	2.4	3.55	-11	16	22	9	CDS
mmu-miR-214-3p	NM_016805	Hnrnpu	ACAGCAGGCACAGACAGGCAGT#TGCCTGTTTGCAGGCTTCCAGCGGAAAGCTGTT#(((((((((.((((((((((..#))))))))))...)))))..........))))).	1951	1984	1	-23	1	2.63E-05	0.6	3	3.06	-5	19	33	10	CDS
mmu-miR-214-3p	NM_016805	Hnrnpu	ACAGCAGGCACAGACAGGCAGT#CTGCCATAACTGTGCAGTTGA#.((((..(((((((...((((((.#)))))).....)))))).)))).	1518	1539	0.92308	-22	0	0.0053882	0.6	4.3	2.6	-9	15	21	6	CDS
mmu-miR-214-3p	NM_016810	Gosr1	ACAGCAGGCACAGACAGGCAGT#ACTGTCCTGTCTTACTGCTGA#.((((((....((((((((((((#)))).)))))))..)))))).	3535	3556	1	-26	1	0.0132466	0.4	4.1	2.91	-11	17	21	7	3UTR

mmu-miR-214-3p	NM_016810	Gosr1	ACAGCAGGCACAGACAGGCAGT#TCTGTGCTCTGCTGG#.(((((((((((((........#))))))).))))))).	1947	1962	0.92308	-23	1	0.0003525	0.5	1.1	1.52	-5	13	15	7	3UTR
mmu-miR-214-3p	NM_016810	Gosr1	ACAGCAGGCACAGACAGGCAGT#CTGTCTGTCAGTGTGGTGCCGCCTGG#.(((..(((((.(((((((((.#)))))))))......)))))..))).	1662	1688	0.92308	-25	0	0.000248	0.4	1.3	0.71	-13	17	26	9	3UTR
mmu-miR-214-3p	NM_016860	Actr1a	ACAGCAGGCACAGACAGGCAGT#ACTGCCTGTGTGTGTGAATGA#.((....(((((.(((((((((#)))))))))).)))))...)).	1273	1294	0.97436	-26	0	0.059774	0.5	1	0.73	-16	16	21	9	3UTR
mmu-miR-214-3p	NM_016895	Ak2	ACAGCAGGCACAGACAGGCAGT#ACTGAACAAGGCCTAGGAGTGTAGCTCAGTGGCGTGTGTGTGCCTGGCTCTGTA#((((((((((((.(((.((...#.)).))).)))))))))...)))).	1518	1572	0.84615	-28	1	0.0212714	0.4	0	0.14	-5	17	24	8	3UTR
mmu-miR-214-3p	NM_016895	Ak2	ACAGCAGGCACAGACAGGCAGT#GCTGCCATGTTTGGCCCCTGA#.(((..(((.((((((((((((#))))))).)))))))))).))).	1733	1754	1	-25	0	2.77E-05	0.7	1.7	1.91	-11	18	21	9	3UTR
mmu-miR-214-3p	NM_016920	Atp6v0a1	ACAGCAGGCACAGACAGGCAGT#CTGCCCGTCTCCACAGTGATCAGCTGTGTCTCTCTTGCCTGCTGG#.(((((((((.((((.(((((.#)))))).))))........................))))))))).	2739	2784	0.92308	-28	1	0.0048566	0.4	0.3	0.06	-7	18	45	9	3UTR
mmu-miR-214-3p	NM_016920	Atp6v0a1	ACAGCAGGCACAGACAGGCAGT#TGCCTGGGCCTTTGTGTCGCCCTGCAACCCCTTGTCCCTGCTGTG#(((((((((((((((((((..#))))))....))))))...................)))))))).	2798	2843	0.84615	-25	1	0.031657	0.4	0.3	0.1	-4	20	45	8	3UTR
mmu-miR-214-3p	NM_016920	Atp6v0a1	ACAGCAGGCACAGACAGGCAGT#GCTGAATGACTGTGCCTGA#....(((((((((.((..((((#))))..)).))))))))).	2903	2922	0.80769	-25	0	0.0353039	0.5	-0	-0.1	-10	15	19	9	3UTR
mmu-miR-214-3p	NM_016961	Mapk9	ACAGCAGGCACAGACAGGCAGT#CTGTGCCTGTTGG#.(((((((((((((.........#))))))))))))).	2667	2680	0.92308	-23	1	0.0004919	0.5	-0	-0.1	-6	12	13	12	3UTR
mmu-miR-214-3p	NM_016965	Nckap1	ACAGCAGGCACAGACAGGCAGT#CCATGTCTGTGACTTGCTGA#.((((((((((((((((....#)).))))))))).))))))).	531	551	1	-26	1	0.0006263	0.6	1.5	2.02	-4	17	20	8	CDS
mmu-miR-214-3p	NM_016965	Nckap1	ACAGCAGGCACAGACAGGCAGT#GCTGCCCGGCTGGCATCTGCACTGCCTTTACCCTGCTGTA#((((((((..(((.(.((((((#)))))).).))))...................)))))))).	4213	4253	0.84615	-24	1	0.0688861	0.5	-0	0.28	-7	18	40	8	3UTR

mmu-miR-214-3p	NM_016965	Nckap1	ACAGCAGGCACAGACAGGCAGT#CTGGAATCTGCTGTGAAATTCATAGTCAGAAAATTTCCTGCTGTA#((((((((((((.(((((((.#)))...)))))))))....................)))))))).	373	418	0.92308	-23	1	0.0009636	0.6	2.5	3.08	-5	20	45	9	CDS
mmu-miR-214-3p	NM_017368	Celf1	ACAGCAGGCACAGACAGGCAGT#GCTGCCTTTCTGATTTCCAGCTGC#.((((.((..((((.(((((((#))))))).))))....)).))))).	4160	4184	0.92308	-24	0	0.0028507	0.6	3.2	3.8	-9	17	24	7	3UTR
mmu-miR-214-3p	NM_017368	Celf1	ACAGCAGGCACAGACAGGCAGT#GCCTGCTGTGTGCCCACAGTCTGACTGC#.((((((((((((.(((((...#)))))))))))).........))).)))).	3911	3939	0.92308	-23	1	0.0007356	0.4	1.9	0.84	-5	17	28	11	3UTR
mmu-miR-214-3p	NM_017368	Celf1	ACAGCAGGCACAGACAGGCAGT#CTGCCTGCCTGCGCTTT#.....((((.(((.(((((((.#))))))).))).)))).	3235	3252	1	-22	0	4.61E-05	0.5	1	1.05	-16	14	17	7	3UTR
mmu-miR-214-3p	NM_017462	Polg	ACAGCAGGCACAGACAGGCAGT#GCTGGTTCTCTGTCCTGCTGTG#((((((((.((((((.((.((((#)))).)).))))))))))))).	4298	4320	1	-27	1	0.0027767	0.5	1.5	2.95	-6	19	22	13	3UTR
mmu-miR-214-3p	NM_017462	Polg	ACAGCAGGCACAGACAGGCAGT#GCTGCCTGCCACAGAGCTTGCTA#..(((((((.(.(.((((((((#)))))))).)...).))))))).	4993	5016	0.84615	-23	1	3.09E-05	0.5	2	3.15	-10	17	23	8	3UTR
mmu-miR-214-3p	NM_018732	Scn3a	ACAGCAGGCACAGACAGGCAGT#ACTCACTGTTTGTGCCGCA#...((.((((((((((((..(((#)))..))))))))))))).	2102	2121	0.92308	-24	0	1.45E-07	0.4	1.1	1.32	-8	16	19	13	CDS
mmu-miR-214-3p	NM_018740	Elp5	ACAGCAGGCACAGACAGGCAGT#GCTGGCTGCTGTGTCA#......(((((((.(((.((((#)))).))))))))))).	663	679	0.92308	-22	0	9.39E-06	0.5	-0	0.73	-16	14	16	10	CDS
mmu-miR-214-3p	NM_018750	Rassf5	ACAGCAGGCACAGACAGGCAGT#GCTGTCTGTGGCCTACGCCTGCA#...((((((..((((((((((#)))))))))...))..)))))).	2092	2115	0.84615	-24	0	2.07E-05	0.4	-0	1.76	-11	17	23	9	3UTR
mmu-miR-214-3p	NM_018768	Stx8	ACAGCAGGCACAGACAGGCAGT#AGTGACCTGCTGTGAGCATTCTGGAACCAGTGACCTGCTGTG#(((((((((((((.((((((.(#).)).))))))))))....................)))))))).	3449	3491	0.84615	-25	1	0.0006646	0.5	-0	-0.1	-5	20	42	9	3UTR
mmu-miR-214-3p	NM_018770	Cadm1	ACAGCAGGCACAGACAGGCAGT#GCTGCCCATGTCTTAAATACGTAGAGCAGCGAAGACTAGAAACGTAGACTGTGACCCAGACGCTGTCGTCTGCTGTG#((((((((...((((((((((#.)))))..........)))))))))))))).	2061	2138	0.84615	-23	1	0.0002798	0.5	1.5	1.26	-11	19	31	14	3UTR

mmu-miR-214-3p	NM_018773	Skap2	ACAGCAGGCACAGACAGGCAGT#TGTCTGTCTGCAAGTGCCTTGGAACATGGAATGCCTGAGTGCCTGCTC#..(((((((((...((((((..#.)))))).))))))))).	1243	1291	0.84615	-28	1	6.29E-06	0.4	1.3	2.02	-10	15	18	9	3UTR
mmu-miR-214-3p	NM_018789	Foxo4	ACAGCAGGCACAGACAGGCAGT#GCCGTCTGTCCTGCC#...(((((.((((((.(((...#)))))))))))))).	937	952	1	-24	0	0.0230179	0.4	0.9	2.06	-8	14	15	14	CDS
mmu-miR-214-3p	NM_018789	Foxo4	ACAGCAGGCACAGACAGGCAGT#CTGCCCTTCTTCCCTGTTGTG#((((((((...(((..(((((.#)))))..)))..)))))))).	2789	2810	0.92308	-22	1	0.0150102	0.4	0.6	1.23	-10	16	21	8	3UTR
mmu-miR-214-3p	NM_018790	Arc	ACAGCAGGCACAGACAGGCAGT#GCCTGTCTTTGCCGCTGC#.((((.((((.((((((((...#)))))))).)))))))).	474	492	0.84615	-29	0	0.0002203	0.4	0	0.13	-9	16	18	8	CDS
mmu-miR-214-3p	NM_018790	Arc	ACAGCAGGCACAGACAGGCAGT#CTGCCTGGTGATTGCCCAGCTGA#.((((.((((....(((((((.#))))))).....))))..)))).	2639	2662	0.92308	-24	0	0.0002145	0.4	1.7	2.03	-14	15	23	7	3UTR
mmu-miR-214-3p	NM_018807	Plagl2	ACAGCAGGCACAGACAGGCAGT#TTGGCTGTTGAGTCCCTGCTGTG#((((((((.((.(((((.(((.#))).)))))..)).)))))))).	2102	2125	0.92308	-25	1	4.08E-05	0.5	2.1	1.38	-8	18	23	8	3UTR
mmu-miR-214-3p	NM_018807	Plagl2	ACAGCAGGCACAGACAGGCAGT#CTCCTGGAGCTCTGAACCTGCTGTG#((((((((..((((((((.((.#))))))....))))..)))))))).	4899	4924	0.88462	-24	1	0.0020075	0.4	3.4	4.04	-6	18	25	8	3UTR
mmu-miR-214-3p	NM_018824	Slc23a2	ACAGCAGGCACAGACAGGCAGT#GCCTGCCTGGGGCTCTGCTGA#.((((((((.(((.(((((...#))))).)))..)).))))))).	935	956	0.92308	-27	1	7.56E-05	0.4	0.3	0.79	-6	16	21	6	CDS
mmu-miR-214-3p	NM_018824	Slc23a2	ACAGCAGGCACAGACAGGCAGT#CCTGTTTGTGCTTGG#....((((((((((((((....#)))))))))))))).	1785	1800	1	-24	0	0.0016398	0.6	-0	-0.2	-10	14	15	14	CDS
mmu-miR-214-3p	NM_018824	Slc23a2	ACAGCAGGCACAGACAGGCAGT#GCTGTCGTGGCTGTGCTTTGTACTGCTC#..(((((((((((.((((((((#)))))).)).))))))......))))).	3177	3205	1	-23	1	0.0025013	0.5	1.4	1.55	-6	19	28	6	3UTR
mmu-miR-214-3p	NM_018875	Snx12	ACAGCAGGCACAGACAGGCAGT#ACTGCCTGGGAAAGCCTTGAAGCG#...((((((.....((((((((#)))))))).....))))....)).	412	436	0.92308	-21	0	0.0002748	0.4	2.9	3.04	-16	14	24	8	CDS
mmu-miR-214-3p	NM_018875	Snx12	ACAGCAGGCACAGACAGGCAGT#GCCCCTTTCCCACCTCGCCAGTGTCGGTGTCCTTGCTGC#.((((((((((.(((((((((.#.)).)))..)))).))).))).)))).	767	806	0.84615	-23	1	0.0027797	0.5	3.8	3.32	-7	19	27	4	3UTR

mmu-miR-214-3p	NM_019405	Cetn2	ACAGCAGGCACAGACAGGCAGT#AGTGCTTGCTGTGGCATTTGG#(((((((((((...........#.))))))))))))).	970	991	0.92308	-20	1	1.02E-05	0.6	3.7	3.31	-10	11	13	11	3UTR
mmu-miR-214-3p	NM_019457	Lrrc6	ACAGCAGGCACAGACAGGCAGT#CTGCCGGTCTCCCTGTTT#..((((((...((((.((((((.#))))).)))).)))))).	81	99	0.92308	-21	1	0.0001149	0.4	-0	0.16	-10	15	18	6	5UTR
mmu-miR-214-3p	NM_019487	Hebp2	ACAGCAGGCACAGACAGGCAGT#CTGTCCAACGGTGGGCTGCTGC#.((((((.(((.....(((((.#)))))).....)))..)))))).	1528	1550	0.84615	-19	1	0.0011179	0.5	1	0.9	-13	14	22	6	3UTR
mmu-miR-214-3p	NM_019585	Espn	ACAGCAGGCACAGACAGGCAGT#GCTGGAGTGCTCTCGCAAACCCCTGCTGTCGCCTGG#....(((((((((.((((....#.)))))))).))))	1056	1092	0.84615	-21	0	0.0024151	0.4	0.5	0.56	-10	13	16	8	3UTR
mmu-miR-214-3p	NM_019586	Ube2j1	ACAGCAGGCACAGACAGGCAGT#CTGCCTGTTTTGCTTGTA#...(((((((.((((((((((.#))))))))))))))))).	3406	3424	1	-26	0	0.0003137	0.5	0.2	0.86	-9	17	18	17	3UTR
mmu-miR-214-3p	NM_019586	Ube2j1	ACAGCAGGCACAGACAGGCAGT#ACTCCTGTCATGTGATGTTGTG#((((((..(((((((((((.(((#)))))))))).)))).))))))).	2512	2534	0.84615	-23	0	0.0006187	0.6	0.4	0.69	-8	19	22	9	3UTR
mmu-miR-214-3p	NM_019586	Ube2j1	ACAGCAGGCACAGACAGGCAGT#TGCTGGTTTGTGACTTAAGAGCTGTG#((((((((((((((.((((..#)))).))))))).)))....))))).	1163	1189	1	-21	1	7.94E-05	0.6	1.6	3.1	-4	19	26	7	CDS
mmu-miR-214-3p	NM_019586	Ube2j1	ACAGCAGGCACAGACAGGCAGT#CTGCCATGAAGGATGTCCTGTTGC#.((((((((.((((..(((((((.#)))))).)).....)))))))))).	666	690	0.92308	-21	1	0.0018185	0.5	1.8	3.19	-7	17	24	10	CDS
mmu-miR-214-3p	NM_019651	Ptpn9	ACAGCAGGCACAGACAGGCAGT#CTGCCTGCTAGCCTGCC#...((((((..(((.((((((((.#))))))))).)))))).	3586	3603	0.97802	-26	0	0.0015855	0.4	0.3	0.22	-11	15	17	9	3UTR
mmu-miR-214-3p	NM_019651	Ptpn9	ACAGCAGGCACAGACAGGCAGT#CTGTCATTCTGTGTGTGTGTGTG#(((((((.(((((((..((((((.#))))))..))))))).))).))).	2895	2918	0.92308	-23	0	0.0220343	0.6	0.9	1.21	-6	18	23	7	3UTR
mmu-miR-214-3p	NM_019661	Ykt6	ACAGCAGGCACAGACAGGCAGT#CTGATCCCTACCCTGAGCCTGCTGA#.((((((((.((((..((((((.#)))...)))...))).)))))))).	1941	1966	0.84615	-23	1	0.0072095	0.4	0.4	-0.3	-7	17	25	8	3UTR
mmu-miR-214-3p	NM_019681	Ncs1	ACAGCAGGCACAGACAGGCAGT#GCTGCCTGTCCATCCCTGTGGTG#((.((((((....((((((((((#))))))))))....))))).)).	2669	2692	0.84615	-27	0	1.63E-05	0.5	-1	-0.6	-14	17	23	10	3UTR

mmu-miR-214-3p	NM_019702	Hbs1l	ACAGCAGGCACAGACAGGCAGT#CCTGTGCTGTTACACTACCAAACCGTCAGTGAGCCTGCTGTT#(((((((((((((((((....#))))).)))).....................)))))))))).	1943	1985	0.84615	-25	1	0.0015975	0.6	4.4	3.82	-4	18	42	9	CDS
mmu-miR-214-3p	NM_019702	Hbs1l	ACAGCAGGCACAGACAGGCAGT#GCTGTTCTGTTTCCATTCTCCTGCTGTG#((((((((...(((((((((((#))))).))))))).......)))))))).	2516	2544	1	-23	1	0.0598518	0.6	0.7	0.74	-9	19	28	8	3UTR
mmu-miR-214-3p	NM_019722	Arl2	ACAGCAGGCACAGACAGGCAGT#CTGCCTGGAGCACTGTCCTGTA#...((((((.((((.((((((((.#))))))))).....))))))))).	420	442	0.92308	-23	0	2.75E-05	0.4	1.6	3.49	-8	16	22	9	CDS
mmu-miR-214-3p	NM_019741	Slc2a5	ACAGCAGGCACAGACAGGCAGT#CTGCCTGCTTTTGTGCTTTCCTGCTGTC#((((((((((((((((((((((.#))))))))..))))))))....)))))))).	2306	2334	0.84615	-30	1	0.0001177	0.5	-0	-0.2	-4	21	28	7	3UTR
mmu-miR-214-3p	NM_019753	Cdh17	ACAGCAGGCACAGACAGGCAGT#ACAGCTTCTCTGTGCTGTTAATGTTGTT#((((((((((((((.((((.((#)).)))).)))))))))).....))))))).	3172	3200	1	-21	1	9.97E-05	0.5	0.2	-0.2	-5	20	28	8	3UTR
mmu-miR-214-3p	NM_019758	Mtch2	ACAGCAGGCACAGACAGGCAGT#ACCAGAGGGCAGCAGAACTTTGGTTGTGCCTGTGCATCTGGCCTGCTGA#.((((((((.((((((((((..#.)))))))....)))))))))))).	2506	2555	0.92308	-31	1	0.0005011	0.5	1.4	-0	-5	18	24	12	3UTR
mmu-miR-214-3p	NM_019778	Zbtb20	ACAGCAGGCACAGACAGGCAGT#ACCACCAGTCTGTTGCCTGTTGC#.((((((((((((((.(((..((#))..))).))))))).)))))))).	190	213	1	-26	1	0.0002084	0.4	0.8	1.42	-4	18	23	8	5UTR
mmu-miR-214-3p	NM_019802	Ggcx	ACAGCAGGCACAGACAGGCAGT#ACTGGTCTGTGGATGGCTTGCTGA#.((((((((....(((((((((#)))).))))).....)))))))).	634	658	1	-23	1	0.0001191	0.5	4.1	2.89	-12	17	24	8	CDS
mmu-miR-214-3p	NM_019802	Ggcx	ACAGCAGGCACAGACAGGCAGT#CTGGCTGTCTTTCGTTTTCTCTTTGCTTTCTTGATGCTGCTGG#.(((((.((((((((((.(((.#))).)))))))......................).)))))))).	288	331	0.92308	-21	0	0.0055842	0.5	3.1	2.83	-5	18	43	8	CDS
mmu-miR-214-3p	NM_019806	Vapb	ACAGCAGGCACAGACAGGCAGT#CTGCCTGCATCCTGATGAGCCTCTGTAGCCATCAGGGCTGCTGTG#(((((((((((((((((((((.#))))))))..............))))).))))........)))))).	5714	5759	0.92308	-27	1	1.28E-07	0.4	-0	0.1	-4	21	45	7	3UTR
mmu-miR-214-3p	NM_019806	Vapb	ACAGCAGGCACAGACAGGCAGT#ACTGCTGCTGTGCCTGAGTGG#.((.((((((((((.((.((((((#))))))))))))))))))..)).	2318	2339	0.92308	-26	0	1.49E-05	0.3	0	-0.5	-8	18	21	16	3UTR

mmu-miR-214-3p	NM_019806	Vapb	ACAGCAGGCACAGACAGGCAGT#ACTGCCCATGTGCCTGG#....((((((((....((((((#))))))..)))))))).	4793	4810	0.92308	-24	0	2.59E-05	0.6	0.3	0.12	-13	14	17	8	3UTR
mmu-miR-214-3p	NM_019808	Pdlim5	ACAGCAGGCACAGACAGGCAGT#ACTGTCTCTCTAATTGTTCGGTCTGACTGCTGCCTGTTGA#.((((((((((((.(((((...#.))))).))).)))))))))).	4405	4445	1	-27	1	0.0010799	0.6	3.4	2.11	-5	17	21	9	3UTR
mmu-miR-214-3p	NM_019808	Pdlim5	ACAGCAGGCACAGACAGGCAGT#ACTGCATGTTAGTGCCAATCTTAGTGCTGA#.((((((((((.((((.(((((#))))).)))).)))))........)))	686	716	0.84615	-24	1	0.0012114	0.5	0.2	0.37	-7	19	30	5	CDS
mmu-miR-214-3p	NM_019809	Pdlim5	ACAGCAGGCACAGACAGGCAGT#ACTGCATGTTAGTGCCAATCTTAGTGCTGA#.((((((((((.((((.(((((#))))).)))).)))))........)))	686	716	0.84615	-24	1	0.0012114	0.5	0.2	0.12	-7	19	30	5	CDS
mmu-miR-214-3p	NM_019809	Pdlim5	ACAGCAGGCACAGACAGGCAGT#CTGTGCCCATTACATCTCCTGCTGTA#((((((((((((.........#)))))))............)))))).	469	495	1	-19	1	0.0100852	0.5	0.5	0.3	-6	13	26	7	CDS
mmu-miR-214-3p	NM_019815	Cldn18	ACAGCAGGCACAGACAGGCAGT#ATTGGTGTGTCTGTGTTTGCC#...((((((((((((.(((((#)))).).))))))))))))).	514	535	0.92308	-24	0	0.0006784	0.5	0.2	-0.1	-7	18	21	13	CDS
mmu-miR-214-3p	NM_019815	Cldn18	ACAGCAGGCACAGACAGGCAGT#CTGTCCCTCCTGGGTCTGGCCGGCTGC#.((((.(((.((((((((.((.#.)))))..)))))))).)))).	151	178	0.92308	-23	0	6.64E-07	0.4	0.1	0.18	-7	17	22	8	CDS
mmu-miR-214-3p	NM_019815	Cldn18	ACAGCAGGCACAGACAGGCAGT#CTGTGTGTCTGGCATAGCTA#..(((..((.((((((.((((.#)))).))))))))...))).	1964	1984	0.84615	-21	0	9.42E-05	0.5	1.1	1.76	-10	15	20	8	3UTR
mmu-miR-214-3p	NM_019913	Txn2	ACAGCAGGCACAGACAGGCAGT#GCTGATTGGCTGACGAGCAGAGAGGAGGCCTGACTACCCTTGCCTGCTGG#.(((((((((.((.(((((...#.))))).))).....))))))))).	576	626	0.92308	-26	1	0.0001674	0.4	-0	0.02	-7	16	24	9	CDS
mmu-miR-214-3p	NM_019926	Mtm1	ACAGCAGGCACAGACAGGCAGT#ACTGTGCGAGGAGCTGGCTGGCTTCTGCTGC#.(((((((...((.(((.((((#.)))).))).))))))))).	2647	2678	0.92308	-21	1	2.26E-05	0.5	-0	0.14	-10	16	20	9	3UTR
mmu-miR-214-3p	NM_019933	Ptpn4	ACAGCAGGCACAGACAGGCAGT#ACTGCCTGTGGATGCAGCACATCTTCCCTGCCATCCCAATAGGGACCTGCTGTT#((((((((..(((((((((((#)))))))))).................)))).............))))))).	3427	3481	0.84615	-24	1	8.16E-05	0.5	4.1	3.72	-6	20	54	9	3UTR

mmu-miR-214-3p	NM_019933	Ptpn4	ACAGCAGGCACAGACAGGCAGT#TGCCTGTAATTGTTTCCCGGGTAGCACCAGGAACACCTGCTGA#.(((((((.(((((((((((..#)))))))..))))......................))))))).	1805	1848	1	-22	1	0.0001052	0.5	4	3.85	-5	18	43	7	CDS
mmu-miR-214-3p	NM_019933	Ptpn4	ACAGCAGGCACAGACAGGCAGT#CTGCTGAGAGTGCATCTGCTGC#.(((((((((.....(((((.#)))))....))))..))))))).	3909	3931	1	-21	1	8.01E-05	0.5	3.6	3.96	-10	15	22	6	3UTR
mmu-miR-214-3p	NM_019935	Ovol1	ACAGCAGGCACAGACAGGCAGT#ACTGGTGTGTCTGGGTATCCTGCTGA#.(((((((..((((((.(((((#)))).).))))))).....))))))).	2336	2362	0.84615	-25	1	0.0002345	0.5	0.5	1.6	-6	18	26	7	3UTR
mmu-miR-214-3p	NM_019935	Ovol1	ACAGCAGGCACAGACAGGCAGT#GCTGATCTGAAGGCTTGCTTCACTTTTCCTTATCGTGTCTGGGCTGCTGC#.((((((.(.((((((.(....#.).))))))).)))))).	2602	2652	1	-23	1	0.0011062	0.5	2.6	1.41	-7	14	18	7	3UTR
mmu-miR-214-3p	NM_019978	Dclk1	ACAGCAGGCACAGACAGGCAGT#ATTGTCTCTCTGTGTCTGTCTGTC#((((((((((((((.(((((((#))))))).)))))))))).)))).	6316	6340	0.84615	-28	1	0.1847508	0.6	0.1	-0.1	-5	21	24	10	3UTR
mmu-miR-214-3p	NM_019978	Dclk1	ACAGCAGGCACAGACAGGCAGT#GCTGCTTGCCCATTACCTGCTGC#.(((((((......((((((((#))))))))).......))))))).	3403	3426	0.92308	-23	1	0.0017245	0.4	2.4	0.39	-14	15	23	8	3UTR
mmu-miR-214-3p	NM_020048	Med20	ACAGCAGGCACAGACAGGCAGT#CTTCCTGCTGTGGCTCAGTTATCCGGTTGCTCCTGTTGC#.((((((((((((.((((.((.#)).))))))))).....................))))))).	1758	1797	0.92308	-21	1	0.0014228	0.5	-0	0.2	-5	18	39	9	3UTR
mmu-miR-214-3p	NM_020048	Med20	ACAGCAGGCACAGACAGGCAGT#ACTGGCAGCTGCCCACCTGCTGA#.(((((((..(((.(.(.((((#)))).).).))))....))))))).	735	758	0.82051	-20	1	0.0018935	0.4	0.3	1.57	-7	16	23	7	3UTR
mmu-miR-214-3p	NM_020260	Arhgap31	ACAGCAGGCACAGACAGGCAGT#GCCGTGTCTGTGCCA#......(((((((((((((...#))).)))))))))).	1796	1811	0.92308	-26	0	0.0005581	0.4	0.4	0.34	-16	13	15	10	CDS
mmu-miR-214-3p	NM_020260	Arhgap31	ACAGCAGGCACAGACAGGCAGT#CTGGACACAAGCAGATGCAGTATACCTGTCTGATGTCCCTTACACCTGCTGG#.((((((((((((((((....#.)))))))).))..........))))))).	5795	5847	0.92308	-26	1	0.0007996	0.5	1.4	1.72	-4	17	29	8	3UTR
mmu-miR-214-3p	NM_020286	Tspan32	ACAGCAGGCACAGACAGGCAGT#CTGTGTGTAGTACACTATCTGTGAGTGCCTGCC#...((((((((..(((((.(((#.))).)))))..))))))))	51	84	0.84615	-25	0	0.0012969	0.5	-0	0.11	-11	16	21	8	5UTR

mmu-miR-214-3p	NM_020295	Lmbr1	ACAGCAGGCACAGACAGGCAGT#ACTCCACTCTGTGCTTGTTGTA#(((((((((((((..(((.(((#)))))..))))))))))))))).	2360	2382	1	-27	1	0.002946	0.6	0.3	0.27	-5	19	22	14	3UTR
mmu-miR-214-3p	NM_020295	Lmbr1	ACAGCAGGCACAGACAGGCAGT#GCGTGCAATATTCTTTGCCTGCTGG#.((((((((((.(((...(((((#)).))).....))).))))))))).	1134	1159	0.84615	-23	1	3.49E-06	0.5	-1	-0.3	-8	17	25	9	CDS
mmu-miR-214-3p	NM_020329	Dolpp1	ACAGCAGGCACAGACAGGCAGT#GCTGAGTCAGGTCCTCAGCCAGGACCCTGGGAGGGCCTGCTGTG#(((((((((.....((((....#.)))).....))))))))).	1068	1112	0.92308	-24	1	1.61E-07	0.4	1.2	1.25	-13	13	20	9	3UTR
mmu-miR-214-3p	NM_020497	Zfp276	ACAGCAGGCACAGACAGGCAGT#CTGCCTCAGTCTGTCTGCAAGAACTGCTA#..(((((((((((((((((((.#))))))..))))))..)).....))))).	914	943	0.92308	-23	1	0.0025156	0.4	2.9	2.81	-5	19	29	6	CDS
mmu-miR-214-3p	NM_020604	Jph1	ACAGCAGGCACAGACAGGCAGT#ACCGTCACTCAGCAGTCTTCTGCTGTGACCTGCTGC#.((((((((((((.((((....#.)))))))))).)))	2961	2997	1	-26	1	0.0044784	0.5	1.4	1.26	-5	16	19	9	3UTR
mmu-miR-214-3p	NM_021284	Kras	ACAGCAGGCACAGACAGGCAGT#ACTGGATGTCTGTCTGATTTTAAGCAAATCACTGTCTGCTT#..((((((((((((((((((..#.))))))))))................)))))))).	2171	2212	0.92308	-25	1	0.0039511	0.6	0.4	0.24	-5	18	36	10	3UTR
mmu-miR-214-3p	NM_021284	Kras	ACAGCAGGCACAGACAGGCAGT#GCTTTCAGCCTGTCTCTCACCTGCC#...(((((...((((((((...#.))))))))....))))).	2422	2447	0.84615	-24	0	0.0546249	0.6	0.1	0.77	-12	13	19	8	3UTR
mmu-miR-214-3p	NM_021287	Sptbn2	ACAGCAGGCACAGACAGGCAGT#ACTGCCTGCTGCTAGGGTCTGCTGC#.((((((((.(((.((((((((#))))))))))).....)))))))).	7713	7738	1	-29	1	3.61E-05	0.4	1.5	1.11	-6	19	25	11	3UTR
mmu-miR-214-3p	NM_021287	Sptbn2	ACAGCAGGCACAGACAGGCAGT#GCTGACCTGCTGGAGCTGCTGG#.((((((...(((.((((((((#)))).)))))))...)))))).	5808	5830	0.84615	-26	1	8.10E-05	0.4	5	3.26	-9	17	22	7	CDS
mmu-miR-214-3p	NM_021362	Pappa	ACAGCAGGCACAGACAGGCAGT#TTGCCTGTCAAGCTGCCGACTGC#.(((..(((((.(((((((((.#)))))))))..).))))..))).	3981	4004	0.92308	-25	0	0.0007079	0.4	2.7	3.38	-13	17	23	9	CDS
mmu-miR-214-3p	NM_021362	Pappa	ACAGCAGGCACAGACAGGCAGT#GCTGCTACTCTTCCTGCTGC#.(((((((...(((..((((((#))))))..))).))))))).	6067	6087	1	-23	1	0.0014385	0.5	2.6	1.45	-10	16	20	7	3UTR

mmu-miR-214-3p	NM_021377	Sorcs1	ACAGCAGGCACAGACAGGCAGT#CTGGTGTATCCCAACGATCAGTATGAACCCTGTCTGTGAATGGCTGA#.((((...((((((((((....#.))))))))))....)))).	5706	5753	0.84615	-25	0	3.84E-05	0.5	3.2	3.66	-10	14	20	10	3UTR
mmu-miR-214-3p	NM_021383	Cnot9	ACAGCAGGCACAGACAGGCAGT#GCCAAGCAGGAACTCTGTGCCTGCTC#..((((((((((((........#.)))))))))))).	1954	1980	1	-24	1	0.0028803	0.5	0.9	0.51	-6	12	14	12	3UTR
mmu-miR-214-3p	NM_021399	Bcl11b	ACAGCAGGCACAGACAGGCAGT#ATTGCTTGGTATTTGTGCTCTGCTT#..((((((((((((((((((((#))))))))...))))))).))))).	7416	7441	1	-25	1	0.0005827	0.7	4.9	3.29	-5	20	25	8	3UTR
mmu-miR-214-3p	NM_021399	Bcl11b	ACAGCAGGCACAGACAGGCAGT#GGTGCTTTCTGTGTCTGCC#...(((((((((((.(((((.(#).)))))))))))))))).	7248	7267	0.84615	-25	0	0.013824	0.5	5.7	3.58	-7	17	19	16	3UTR
mmu-miR-214-3p	NM_021399	Bcl11b	ACAGCAGGCACAGACAGGCAGT#GCCTAACCTGTGTCTGCG#...(((((((((..((((...#))))...))))))))).	2833	2851	0.92308	-25	0	0.000411	0.4	0.6	3.74	-8	14	18	10	3UTR
mmu-miR-214-3p	NM_021423	Shank3	ACAGCAGGCACAGACAGGCAGT#ACTGGGTGTGGCTGAGCGCATGGGTGCCCTGTGTGTGCTTGACTGTG#(((((((((((.((((((((.(#.).)))).))).))))))))).)))).	6394	6441	1	-29	1	0.0003017	0.4	0	0.19	-5	20	26	8	3UTR
mmu-miR-214-3p	NM_021423	Shank3	ACAGCAGGCACAGACAGGCAGT#GCCTGGGCAGCCTGCTGG#.((((((((.....(((((...#)))))....)))))))).	4801	4819	0.92308	-27	1	9.42E-06	0.4	1.8	3.49	-13	13	18	8	CDS
mmu-miR-214-3p	NM_021433	Stx6	ACAGCAGGCACAGACAGGCAGT#CTGCCTCTCTGTGCA#.......(((((((.((((((.#))))))).))))))).	2281	2296	0.92308	-23	0	0.0031793	0.5	0.4	0.11	-18	13	15	7	3UTR
mmu-miR-214-3p	NM_021442	Mecom	ACAGCAGGCACAGACAGGCAGT#AGTGTGTGCGTGTGTGTGCGTGTG#...(((.(((((.(((.(((..#.))).))).))))).))).	37	61	0.84615	-24	0	0.0012254	0.5	0.2	0.25	-9	14	19	5	5UTR
mmu-miR-214-3p	NM_021544	Scn5a	ACAGCAGGCACAGACAGGCAGT#GCTGGGCCTGTCTCGCATGGGCAACCTGTCTGTGCTGCGTTCCTTCCGTCTGCTGC#.(((((((((((((((((..((#.))..)))))))))))))...........	2606	2662	0.92308	-29	1	0.0048814	0.4	3.2	4.19	-4	19	37	11	CDS
mmu-miR-214-3p	NM_021547	Stard3	ACAGCAGGCACAGACAGGCAGT#CTGCCCTGTCCTGCTGA#.((((((((.((((...((((((.#)))))))))))))))))).	1059	1076	1	-25	1	0.0034254	0.5	1.1	4	-7	16	17	16	CDS

mmu-miR-214-3p	NM_021567	Pcbp4	ACAGCAGGCACAGACAGGCAGT#GCTGCCGTCTTCCACGCGGTCTCCATGATTGCCTTCAAGCTGG#.(((((((((.((((.((((((#))))))))))...................)))))....)))).	441	484	0.92308	-22	1	0.0022987	0.5	2.3	1.97	-7	19	43	10	CDS
mmu-miR-214-3p	NM_021607	Ncstn	ACAGCAGGCACAGACAGGCAGT#ACTGTCTCTGGGCCTGCTT#..(((((((.((((..((((((#)))))))))).))))))).	2281	2300	0.84615	-25	1	0.0003158	0.5	0.9	2.45	-7	17	19	10	3UTR
mmu-miR-214-3p	NM_021607	Ncstn	ACAGCAGGCACAGACAGGCAGT#CTGCCTGCTCTGAACTTTACTTCCCAGACCAGGTGTCCGGCTGG#.((((.((((((((((((((.#))))))).))).....................)))))..)))).	2212	2256	0.84615	-25	0	1.93E-07	0.5	2.6	1.97	-6	19	44	7	3UTR
mmu-miR-214-3p	NM_021607	Ncstn	ACAGCAGGCACAGACAGGCAGT#GCTCCTTGTGTCCGCCTGCTC#..(((((((..(.(((((.(((#))).))))).)..))))))).	205	226	0.92308	-21	1	0.0492913	0.5	0.6	0.2	-10	16	21	7	CDS
mmu-miR-214-3p	NM_021609	Ackr2	ACAGCAGGCACAGACAGGCAGT#GCTTGTGTGTGGAAAGCCTTTGGAAAGCTGCTGTG#(((((((.((((.((((((...#)))))).))))................))))))).	2231	2266	0.92308	-23	1	0.0037857	0.5	0	0.17	-8	17	35	7	3UTR
mmu-miR-214-3p	NM_021609	Ackr2	ACAGCAGGCACAGACAGGCAGT#CTCCTGTCGTGCTCCCTGCA#...((((((((.(((((((.(((.#))))))))))))...)))).	2122	2142	0.84615	-22	0	1.06E-05	0.4	-0	0.11	-10	16	20	12	3UTR
mmu-miR-214-3p	NM_021609	Ackr2	ACAGCAGGCACAGACAGGCAGT#CTGAAGGCATTTCTGTCTGTGATGTTGA#.(((((..((((((((((....#.)))))))))).))))).	1352	1380	0.92308	-21	0	0.0173136	0.4	0.5	0.87	-8	15	18	10	CDS
mmu-miR-214-3p	NM_021610	Gpa33	ACAGCAGGCACAGACAGGCAGT#CTGCCTGTCAAGGGCCTGCCTGTC#(((((((((...(((((((((.#)))))))))....)))))).))).	1931	1955	0.84615	-31	1	5.08E-05	0.5	0.4	-0.2	-11	18	24	9	3UTR
mmu-miR-214-3p	NM_021716	Fign	ACAGCAGGCACAGACAGGCAGT#CTGCCCCTCTGCTCCTGCTT#..((((((..((((..(((((.#)))))..))))..)))))).	4286	4306	0.84615	-24	1	0.0079537	0.5	1	0.93	-8	15	20	6	3UTR
mmu-miR-214-3p	NM_021716	Fign	ACAGCAGGCACAGACAGGCAGT#GCTGTTCTCGAGATTTAGTTGCTGTCAAGTGCCTGGAGTG#((..(((((((.((((((((((#))))).................)))))..)))))))..)).	2949	2989	0.84615	-23	0	9.61E-06	0.6	0.7	1.49	-12	19	40	7	3UTR
mmu-miR-214-3p	NM_021716	Fign	ACAGCAGGCACAGACAGGCAGT#TGTGTGTGTGTGTGTGTGTGTG#((((((.((((((.(((.(((..#))).))).))))).))).))).	6610	6632	0.84615	-22	0	0.2326371	0.6	4	3.62	-7	17	22	5	3UTR

mmu-miR-214-3p	NM_021877	Hr	ACAGCAGGCACAGACAGGCAGT#ACCGGCTGTGTGTAGCCTGTGGTC#((.((((((((((.((((.(.((#)).).)))).))).)))))).)).	2534	2558	0.84615	-23	0	1.84E-06	0.4	2	1.38	-7	18	24	6	CDS
mmu-miR-214-3p	NM_021881	Qk	ACAGCAGGCACAGACAGGCAGT#ACTGTTTTACCAGACAGAATTTGAAACTTTTTTGTGTATGACCTTGGTGCTGTGTGCCTGCTGTT#((((((((((..(((.(((.(#.).).))))))	1580	1645	1	-26	1	0.0164085	0.6	0.8	1.47	-9	18	21	17	3UTR
mmu-miR-214-3p	NM_021885	Tub	ACAGCAGGCACAGACAGGCAGT#CTGTGTGTGTGTGTCTGTCTGTG#((((((((((((.(((.((((.#)))).))).)))))))).)))).	4315	4338	0.92308	-30	1	0.1816823	0.5	0.6	1.2	-5	19	23	8	3UTR
mmu-miR-214-3p	NM_021885	Tub	ACAGCAGGCACAGACAGGCAGT#GCCTGTCAGAGCTTGCTGTA#((((((((.(.(((((((...#))))))).).))))))))).	5331	5351	1	-30	1	0.0058914	0.5	0.2	0.3	-9	17	20	9	3UTR
mmu-miR-214-3p	NM_021885	Tub	ACAGCAGGCACAGACAGGCAGT#ACTGGCTGCTCTGCCTCTGCTGC#.(((((((..(((((((.((((#)))).))).)))))..))))))).	1859	1882	0.84615	-26	1	1.97E-05	0.4	0.6	0.39	-6	18	23	7	3UTR
mmu-miR-214-3p	NM_022408	Ess2	ACAGCAGGCACAGACAGGCAGT#GCTGCCTCCCTGTCCTCCTCTCTGCTTGACCCACTGTTCTTCAGCTCTGCCTGCTGG#.((((((((((((.(((((((#)))))))............))))......................))))))))).	2048	2105	0.92308	-26	1	9.70E-06	0.5	3.6	3.06	-5	20	57	9	3UTR
mmu-miR-214-3p	NM_022408	Ess2	ACAGCAGGCACAGACAGGCAGT#GCTACGCCTGTTAGCCTGTGCCTACAGAGAGCTGA#.((((((((((((((((((((#)))..))))))....))))))))).......)))).	2239	2274	0.84615	-27	1	0.0001486	0.4	2.9	3.2	-4	21	35	8	3UTR
mmu-miR-214-3p	NM_022408	Ess2	ACAGCAGGCACAGACAGGCAGT#CTGGAACTGTCTGGCCAGGCTGC#.((((.(((.(((((((.(((.#)))...)))))))))))..)))).	1754	1777	1	-25	0	0.0007536	0.4	2.6	3.51	-7	17	23	10	3UTR
mmu-miR-214-3p	NM_022554	Pdlim5	ACAGCAGGCACAGACAGGCAGT#GCTTGGAAAGAGTTGTAATAAAGTTGTCTGTACCGTTGC#.((((.((.((((((((.....#.)))))))	1738	1777	0.92308	-17	0	0.0230703	0.6	2.4	3	-7	14	17	8	3UTR
mmu-miR-214-3p	NM_023055	Slc9a3r2	ACAGCAGGCACAGACAGGCAGT#CTGCCATCCTGCCTCGGGCCTGCTGA#.(((((((.(....((((((.#.))))))..))))))))).	1463	1489	1	-26	1	0.0008569	0.5	4	2.36	-11	15	19	9	3UTR
mmu-miR-214-3p	NM_023055	Slc9a3r2	ACAGCAGGCACAGACAGGCAGT#GCCAGCTGTGCCTGGAGGAGCTGTC#(((((((((((((.(.(((...#))).))))))))))......))))).	1703	1728	1	-25	1	1.84E-06	0.4	1.5	2.22	-5	17	25	9	3UTR

mmu-miR-214-3p	NM_023172	Ndufb9	ACAGCAGGCACAGACAGGCAGT#TTGCCCTAATAAAAGGAACTAGACACGTTGGCTGTGCCTGAAGTG#((..(((((((((.(((((((.#)))))......................)).)))))))))..)).	633	678	0.84615	-22	0	2.64E-06	0.7	-0	0.5	-9	18	45	9	3UTR
mmu-miR-214-3p	NM_023196	Pla2g12a	ACAGCAGGCACAGACAGGCAGT#ACGCCGCGCTGGACCTGCTGG#.(((((((..(((...(((.((#)))))...)))..))))))).	270	291	0.84615	-24	1	2.82E-05	0.4	3.6	3.13	-8	15	21	7	CDS
mmu-miR-214-3p	NM_023214	Slc30a7	ACAGCAGGCACAGACAGGCAGT#GGTGTGTGTCTGTGTCTGTG#...((((((((((((.(((.(#).))).)))))))))))).	43	63	0.92308	-27	0	0.1070615	0.3	1.1	1.05	-7	17	20	13	5UTR
mmu-miR-214-3p	NM_023214	Slc30a7	ACAGCAGGCACAGACAGGCAGT#CTGCCCAGACTGCTGCCTGTGTG#(((.(((((((((...(((((.#)))))....))).))))))))).	1493	1516	1	-23	0	0.0023644	0.4	0.1	0.16	-9	17	23	9	3UTR
mmu-miR-214-3p	NM_023214	Slc30a7	ACAGCAGGCACAGACAGGCAGT#CTGTCTGTCTGTCTTTCTGG#.(((.(((.((((((((((((.#))))))))))))))).))).	5970	5990	1	-26	0	0.0021594	0.5	0.2	0.27	-9	18	20	15	3UTR
mmu-miR-214-3p	NM_023232	Diablo	ACAGCAGGCACAGACAGGCAGT#TTGTCTGCTCACACCCTCTGTGCCTGGTT#..(.((((((((((((((((.#))))))).........)))))))))).).	901	930	0.92308	-25	0	0.0017872	0.4	-0	0.35	-8	18	29	10	3UTR
mmu-miR-214-3p	NM_023232	Diablo	ACAGCAGGCACAGACAGGCAGT#CTGCCATTGACAGTCAGACCAGAGCCTTCTGTGAGCTGTGCCTGG#....(((((((((.(..((((.#.))))..)))))))))).	852	897	0.92308	-23	0	1.37E-06	0.4	0.7	0.35	-11	14	18	10	3UTR
mmu-miR-214-3p	NM_023268	Qsox1	ACAGCAGGCACAGACAGGCAGT#GCTGTCCTGGACTGTGCTGAGGAGACCAACAGTGCTGTC#(((((((((((((.((((((((#)))).))))..)))))))).............)))))).	397	436	0.84615	-27	1	0.0001913	0.4	0.1	0.09	-5	21	39	7	CDS
mmu-miR-214-3p	NM_023314	Eif4e2	ACAGCAGGCACAGACAGGCAGT#TGTGTGTGTGTGTCTGTGTGTG#(((((((((((.(((.(((..#))).))).))))))))).))).	2401	2423	1	-25	1	0.9117894	0.5	0.1	-0	-5	18	22	9	3UTR
mmu-miR-214-3p	NM_023397	Mdp1	ACAGCAGGCACAGACAGGCAGT#GCCTTGGCGTGCCTGTCGCCGCTGC#.((((.(((...((((((((((#.)).))))))))))))))).	255	280	0.84615	-25	0	0.0014096	0.4	-0	-0.1	-13	17	20	15	CDS
mmu-miR-214-3p	NM_023403	Mesd	ACAGCAGGCACAGACAGGCAGT#GCTGTCCTGTTTGAGGGTGCTGC#.(((((....((((((((((((#)))).))))))))....))))).	2060	2083	0.82692	-24	0	1.89E-05	0.5	0	0	-10	17	23	8	3UTR

mmu-miR-214-3p	NM_023403	Mesd	ACAGCAGGCACAGACAGGCAGT#GCTGCTGTTCACATCTGTTTCTGCTGTT#((((((((.(((((..((((((#))))))......))))).)))))))).	1598	1626	0.92308	-24	1	0.0008967	0.6	0	0	-6	19	28	8	3UTR
mmu-miR-214-3p	NM_023403	Mesd	ACAGCAGGCACAGACAGGCAGT#GCTGTGGTCCCATGGCCTGTTGTA#(((((((((.(((((..(((((#))))).)))...)))))))))))).	1014	1038	1	-24	1	0.0026421	0.5	0	0	-5	19	24	11	3UTR
mmu-miR-214-3p	NM_023420	Cert1	ACAGCAGGCACAGACAGGCAGT#GCTTCTTTTTACAGGCCTACAGTCTGTGTCTGTG#...((((((((((((((((.((#.))..))))...))))))))	2750	2784	1	-26	0	0.0001878	0.6	1.2	1.57	-7	18	25	12	3UTR
mmu-miR-214-3p	NM_023449	Slc9a3r2	ACAGCAGGCACAGACAGGCAGT#CTGCCATCCTGCCTCGGGCCTGCTGA#.((((((((.(....((((((.#.))))))..))))))))).	1033	1059	1	-26	1	0.0008569	0.5	1.9	2.18	-11	15	19	9	3UTR
mmu-miR-214-3p	NM_023449	Slc9a3r2	ACAGCAGGCACAGACAGGCAGT#GCCAGCTGTGCCTGGAGGAGCTGTC#(((((((((((((.(.(((...#))).)))))))))......))))).	1273	1298	0.92308	-25	1	1.84E-06	0.4	3	1.95	-5	17	25	9	3UTR
mmu-miR-214-3p	NM_023508	Pdcl2	ACAGCAGGCACAGACAGGCAGT#CTGTGGGTTGTAATTCATCTCTATAGATCAAGTGTCCCAATGTGCTTGGTGG#.((.((((((((....((((.(#.).))))....)))))))).)).	468	520	0.92308	-20	0	3.83E-05	0.6	3.3	2.62	-10	15	23	8	CDS
mmu-miR-214-3p	NM_023508	Pdcl2	ACAGCAGGCACAGACAGGCAGT#TGCCTTATTTCCTGCTGA#.(((((((.......(((((..#))))).....))))))).	899	917	0.92308	-19	1	0.0069884	0.6	3.1	2.83	-15	12	18	7	3UTR
mmu-miR-214-3p	NM_023566	Muc2	ACAGCAGGCACAGACAGGCAGT#CTGCCTGAAGACTGTCGTGCTGTT#((((((.(.((((.(((((((.#)))))))....))))).))))))).	1348	1372	0.92308	-26	0	3.29E-05	0.5	0	0	-7	18	24	7	CDS
mmu-miR-214-3p	NM_023566	Muc2	ACAGCAGGCACAGACAGGCAGT#GCTTGCCTGTTTGATAGCTGCTATGTGCCTGGCTC#..((((((((((((((((((((#))).))))))).............)))))))).)).	12365	####	0.92308	-26	1	0.0012745	0.5	0	0	-5	20	35	8	CDS
mmu-miR-214-3p	NM_023624	Lrat	ACAGCAGGCACAGACAGGCAGT#GCTCCAGACCTGTGTGGGCTGCAGCACAATACTGTCTGCTGTC#((((((((((((.((.((((.#.)))).))..)))..........))))))))))).	2270	2313	1	-24	1	7.57E-05	0.5	0.4	0.11	-5	19	35	10	3UTR
mmu-miR-214-3p	NM_023624	Lrat	ACAGCAGGCACAGACAGGCAGT#CTCCTGTCATCATCCTGCTGTT#((((((((....((((((.((.#))))))))).....)))))))).	1293	1315	1	-23	1	0.0079594	0.6	0	-0.1	-12	16	22	8	3UTR

mmu-miR-214-3p	NM_023625	Plbd2	ACAGCAGGCACAGACAGGCAGT#ACACCCTGTCTGTCCATGGCTGTC#(((((.((.(((((((((..((#))..)))))))))))...))))).	2415	2439	1	-24	0	0.0025113	0.4	4.3	2.01	-7	18	24	11	3UTR
mmu-miR-214-3p	NM_023625	Plbd2	ACAGCAGGCACAGACAGGCAGT#GCTGGCCTGTGGGCCCATCGCCTCAGGAGGCAGCCCTGTGCCCTTCTGG#.(((..((((((((((((((#)))).))))).......................)))))))..	2135	2184	0.84615	-23	0	0.0001059	0.4	2.5	2.95	-10	19	49	7	3UTR
mmu-miR-214-3p	NM_023653	Wnt2	ACAGCAGGCACAGACAGGCAGT#ACTGGTGCTGTGCCGTGCGCTGTC#(((((.(((((((.((..((((#)))).)))))))))....))))).	1143	1167	0.82051	-22	0	0.0001282	0.4	4.4	3.53	-7	18	24	9	CDS
mmu-miR-214-3p	NM_023663	Ripk4	ACAGCAGGCACAGACAGGCAGT#GTGTGTCAAGTGGCTGCTGC#.((((((.(((.((((.(((...#)).))))..))).))))))).	1431	1451	0.84615	-25	1	0.0001043	0.4	2.9	1.8	-9	15	20	6	CDS
mmu-miR-214-3p	NM_023671	Clns1a	ACAGCAGGCACAGACAGGCAGT#CTGCCTGGCTCTGCTCTCCTACTGCTGG#.(((((((((.((.(((((((.#))))))).)).)))).......)))))).	2188	2216	0.92308	-26	1	0.0054727	0.5	0	0	-7	18	28	7	3UTR
mmu-miR-214-3p	NM_023671	Clns1a	ACAGCAGGCACAGACAGGCAGT#CTGCTTCTGTGTTGGGCAGCTCACAGCTGCTATATGCTGCTGG#.(((((((((((((..(((((.#))))))))))))).......................)))))).	1776	1819	0.89744	-23	1	1.21E-05	0.5	0	0.01	-5	18	43	12	3UTR
mmu-miR-214-3p	NM_023671	Clns1a	ACAGCAGGCACAGACAGGCAGT#TGTGTGTTTTGCCTGCG#...(((((((.(((((.(((..#))).)))))))))))).	1552	1569	1	-23	0	9.91E-05	0.6	0.2	0.06	-9	15	17	12	3UTR
mmu-miR-214-3p	NM_023727	Rd3	ACAGCAGGCACAGACAGGCAGT#GCTGCTACTGCTGTCCCTGCC#...(((((.((((.(((((((#)))))))..))))).))))).	2840	2861	0.84615	-24	0	0.0021808	0.4	-0	-0	-8	17	21	7	3UTR
mmu-miR-214-3p	NM_023727	Rd3	ACAGCAGGCACAGACAGGCAGT#CTGTCTGTCTGTCTGTC#...((((..(((((((((((.#))))))))))))))).	3276	3293	1	-24	0	0.015112	0.5	0.3	0.2	-10	16	17	16	3UTR
mmu-miR-214-3p	NM_023727	Rd3	ACAGCAGGCACAGACAGGCAGT#GCCTGCGAACCTGCTGC#.(((((((....(.(((((...#))))))...))))))).	1585	1602	1	-23	1	0.0005707	0.4	0.3	0	-13	13	17	7	3UTR
mmu-miR-214-3p	NM_023750	Zfp84	ACAGCAGGCACAGACAGGCAGT#ACTTTATTCATGACATCGCCTGTCAGTGCTTGTC#...((((((((.(((((((.((#.)).))))))).))))))))	2554	2588	0.9359	-26	0	0.0048233	0.5	0.1	0.1	-10	17	21	8	3UTR
mmu-miR-214-3p	NM_023792	Pank1	ACAGCAGGCACAGACAGGCAGT#TGTCATCTGTGTTTGTTGTT#((((((((((((((..((((..#)))).)))))))))))))).	4251	4271	1	-23	1	0.178895	0.8	0.9	0.38	-5	18	20	14	3UTR

mmu-miR-214-3p	NM_023792	Pank1	ACAGCAGGCACAGACAGGCAGT#GCCTATGTTGCTTGCTGA#.((((((((((...((((...#)))).))).))))))))).	862	880	1	-22	1	0.0095672	0.5	0.2	0.18	-7	15	18	8	CDS
mmu-miR-214-3p	NM_023792	Pank1	ACAGCAGGCACAGACAGGCAGT#GCCACTATGGTGGATGAACCTGCTGTA#((((((((..((..(((((...#)))........))..))..)))))))).	1403	1430	1	-21	1	0.0093265	0.6	0.2	-0	-8	15	27	8	3UTR
mmu-miR-214-3p	NM_023805	Slc38a3	ACAGCAGGCACAGACAGGCAGT#ACTGGCCTGCTTACGTGCATCAATCTGCTGG#.((((((((((((.((((((((#)))).))))))...))))......)	1549	1580	0.84615	-24	1	0.0012247	0.5	0.3	1.96	-5	20	31	6	CDS
mmu-miR-214-3p	NM_023805	Slc38a3	ACAGCAGGCACAGACAGGCAGT#ACTGCTGCTGAGGCCTTCTGTA#((((.(((((.(((.(((.(((((#))))))))))..)))).))))).	1120	1142	0.84615	-23	0	5.77E-05	0.4	4.1	3.19	-10	18	22	10	CDS
mmu-miR-214-3p	NM_024170	Rtl8a	ACAGCAGGCACAGACAGGCAGT#CTGCTGCTGTCCCTGCTGG#.(((((((.((((.(((.((((.#)))))))))).))))))).	711	730	0.84615	-26	1	7.86E-06	0.3	-0	-0	-6	17	19	10	3UTR
mmu-miR-214-3p	NM_024189	Yaf2	ACAGCAGGCACAGACAGGCAGT#ACTGGGACTGCAGTGTCTGCA#...((((((((.(.(((.((((#))))...)))).)))))))).	217	238	0.92308	-20	0	7.41E-06	0.4	1	1.03	-11	16	21	8	CDS
mmu-miR-214-3p	NM_024209	Ppp6c	ACAGCAGGCACAGACAGGCAGT#GCTATGTGACTATGTTTGTGATCTGCTC#..((((((((((((((((...#)))..........)))))))).))))))).	185	213	0.92308	-20	1	0.0053169	0.5	1.8	1.81	-5	17	28	8	CDS
mmu-miR-214-3p	NM_024233	Rexo2	ACAGCAGGCACAGACAGGCAGT#GCGTCTCTCTCTCCTGCTGA#.(((((((.(.((((.((((.((#)))))).))).)))))))).	817	837	0.84615	-18	1	0.0055829	0.5	4.8	4.05	-8	17	20	8	3UTR
mmu-miR-214-3p	NM_024452	Luzp1	ACAGCAGGCACAGACAGGCAGT#GCTGCCTGTCTGCATGTATCCTTTGGCTGTA#(((((((((((((((((((((#)))))))))))))..))...)))...)	7436	7467	1	-29	1	0.0001369	0.5	1.6	1.69	-4	22	31	12	3UTR
mmu-miR-214-3p	NM_025301	Mrpl17	ACAGCAGGCACAGACAGGCAGT#TGCCATGGCCTGTGTCTGTTA#..(((((((((((.((((((..#)))).))..))))))))))).	5890	5911	1	-24	1	0.0031114	0.5	0.6	0.41	-6	17	21	11	3UTR
mmu-miR-214-3p	NM_025301	Mrpl17	ACAGCAGGCACAGACAGGCAGT#TTGCTTGTTTGTTGGAGGGGAGGGGATCTTGCTGTG#((((((((.((((((((((((.#)))))))))))))...............)))))))).	927	963	0.96154	-23	1	9.96E-05	0.5	0.1	-0	-5	20	36	12	3UTR
mmu-miR-214-3p	NM_025301	Mrpl17	ACAGCAGGCACAGACAGGCAGT#CTGCTTACAGGAGCATGTGTGTGTCTTACCAATGGCTGTA#(((((((((((((.((((((((.#))))))..........))).)))))))........))))).	1509	1549	0.84615	-23	1	0.0013	0.5	-0	-0.1	-5	20	40	7	3UTR

mmu-miR-214-3p	NM_025301	Mrpl17	ACAGCAGGCACAGACAGGCAGT#TGCCTGCTGTGTTTATGTA#(((..((((((((.((((((..#))))))))))))))).))).	3670	3689	1	-23	0	0.0011408	0.7	-0	-0.1	-12	17	19	14	3UTR
mmu-miR-214-3p	NM_025325	Haao	ACAGCAGGCACAGACAGGCAGT#GCCCCAGATGACAGCCTGCTGGTGCCTGCTGG#.((((((((((((.(((((.((#.)).)))))))).))))))	818	850	0.84615	-32	1	1.07E-06	0.4	1.6	2.28	-5	19	23	9	CDS
mmu-miR-214-3p	NM_025359	Tspan13	ACAGCAGGCACAGACAGGCAGT#GCTCAACCTGCTGTATACCCTGGTCAGCCTGCTGC#.((((((((((((.((((.(((#)))...))))))))..........)))))))).	209	244	0.84615	-25	1	0.0001088	0.4	0.2	0.39	-5	19	35	8	CDS
mmu-miR-214-3p	NM_025374	Glo1	ACAGCAGGCACAGACAGGCAGT#ATTGCCGTTCCTGATGTCTACAGTGCCTGTA#...(((((((((((((((((((#))))))........)))))...)))	495	526	1	-24	0	0.0002171	0.6	0.2	0.02	-7	19	31	8	CDS
mmu-miR-214-3p	NM_025374	Glo1	ACAGCAGGCACAGACAGGCAGT#ACTGCCTGTCTCTTAAAGACTGCTC#..(((((....(((((((((((#)))))))))))........))))).	967	992	0.92308	-24	0	5.96E-05	0.6	0.3	1.01	-12	16	25	11	3UTR
mmu-miR-214-3p	NM_025376	Mymk	ACAGCAGGCACAGACAGGCAGT#CTGCCTGGCAGTCCTGCTGTC#((((((((.((...(((((((.#)))))))...)))))))))).	855	876	1	-28	1	6.88E-06	0.4	3.2	2.97	-10	17	21	10	3UTR
mmu-miR-214-3p	NM_025376	Mymk	ACAGCAGGCACAGACAGGCAGT#CTGCCTGTCAGAATCGTCTGCTT#..(((((((...(((((((((.#)))))))))......))))))).	1140	1163	0.92308	-26	1	0.0099818	0.5	3.2	3.67	-12	16	23	9	3UTR
mmu-miR-214-3p	NM_025376	Mymk	ACAGCAGGCACAGACAGGCAGT#CTGGCCATGTCCTTTGTCCTGCTGC#.((((((((.((((((((((((.#))).)).))))...)))))))))).	704	729	1	-24	1	0.0251558	0.4	3.4	3.52	-5	19	25	10	CDS
mmu-miR-214-3p	NM_025402	Ogfod3	ACAGCAGGCACAGACAGGCAGT#TGCCTTATGAATTATCCCGAGTCTCAACCTGCTGA#.(((((((...(((((((((..#)))))).............))))...))))))).	1142	1177	0.84615	-21	1	0.0387147	0.6	1.1	0.09	-9	16	35	7	3UTR
mmu-miR-214-3p	NM_025402	Ogfod3	ACAGCAGGCACAGACAGGCAGT#CTGCACTCTGGAGCCTTGTCTGTTGG#.(((((((((((((...((((.#))))..))))......))))))))).	512	538	1	-20	1	0.001611	0.5	3	2.68	-5	17	26	9	CDS
mmu-miR-214-3p	NM_025407	Uqcrc1	ACAGCAGGCACAGACAGGCAGT#CTGCCGAGCGGCCTGCTC#..(((((((.(.(...(((((.#)))))...))))))))).	88	106	0.84615	-22	1	9.26E-06	0.3	0	0.95	-11	14	18	9	CDS

mmu-miR-214-3p	NM_025451	Camk2n1	ACAGCAGGCACAGACAGGCAGT#CTGCCTGATCTGCAGAGACCGATGAACTCAGCTGCTGC#.((((((...(((((((((((.#))))))).))))...................)))))).	1953	1991	0.84615	-24	1	0.0211074	0.5	-0	-0.2	-8	17	38	7	3UTR
mmu-miR-214-3p	NM_025451	Camk2n1	ACAGCAGGCACAGACAGGCAGT#ACTGCCTGGAGCTTTTAATGTGTTTGTTT#..((((((((((..((((((((#))))))))..........)))))))))).	2424	2453	1	-24	1	0.0103464	0.5	2.1	0.96	-7	18	29	10	3UTR
mmu-miR-214-3p	NM_025451	Camk2n1	ACAGCAGGCACAGACAGGCAGT#GCTGCCTGTTTTGCATGGTA#..(.((.(((.((((((((((((#)))))))))))))))).)).).	1405	1425	0.92308	-22	0	7.46E-05	0.5	1.6	2.24	-12	17	20	14	3UTR
mmu-miR-214-3p	NM_025566	Tnfaip8l1	ACAGCAGGCACAGACAGGCAGT#TGCCTGGGGTAGTCTCTCCTCTGTTTCTCCTGCTGTA#((((((((.(((((((((((..#)))))))............)))))....)))))))).	748	785	1	-25	1	0.1299608	0.5	-1	-0.1	-5	19	37	8	3UTR
mmu-miR-214-3p	NM_025566	Tnfaip8l1	ACAGCAGGCACAGACAGGCAGT#CTGCAGCCTCTACTATCTGAGTGTGCCTGTGGTC#((.(((((((((..((((.(((#.))).)))))..)))))))	1814	1848	1	-24	0	0.0001459	0.5	0.2	0.06	-8	18	24	9	3UTR
mmu-miR-214-3p	NM_025590	Acot11	ACAGCAGGCACAGACAGGCAGT#GCTGCTTTTGGACTTGCTGG#.(((((((..((((..((((((#)))))))))))..)))))))).	11	31	0.92308	-23	1	0.0001858	0.4	0.2	0.33	-7	17	20	10	5UTR
mmu-miR-214-3p	NM_025590	Acot11	ACAGCAGGCACAGACAGGCAGT#GCTGGCTGTCCCTGCG#...(((((....(((((.((((#)))).))))))))))).	373	389	1	-22	0	6.28E-06	0.4	0.5	-0.2	-15	14	16	10	CDS
mmu-miR-214-3p	NM_025590	Acot11	ACAGCAGGCACAGACAGGCAGT#GCTTCTGTCTGTGGCGTGAGGG#.(..((.((((((((((((.(((#))))))))))))))).).))..).	1706	1728	0.84615	-22	0	1.03E-06	0.5	-0	-0.1	-10	17	22	13	CDS
mmu-miR-214-3p	NM_025590	Acot11	ACAGCAGGCACAGACAGGCAGT#TGTGTGTGTGTGTGTGTGTGTG#((((((.((((((.(((.(((..#))).))).))))).))).))).	3713	3735	0.84615	-22	0	0.6352039	0.6	-0	0.59	-7	17	22	5	3UTR
mmu-miR-214-3p	NM_025613	Eid1	ACAGCAGGCACAGACAGGCAGT#TTGCTGTGTGTGCCACGCC#...((.((((((.(((.((((.#))))))).))))))..)).	1734	1753	0.92308	-21	0	0.0004318	0.6	-0	0.13	-10	15	19	7	3UTR
mmu-miR-214-3p	NM_025649	Mad2l1bp	ACAGCAGGCACAGACAGGCAGT#CTGTCCTGGCCATGTGCCTTCTCTCCCCAGACTTCCTGCTGA#.(((((((((((..(((((((.#))).))))....)))))))..................)))).	964	1006	0.92308	-24	1	0.0031608	0.4	3.2	2.94	-6	18	42	7	3UTR

mmu-miR-214-3p	NM_025670	Cdip1	ACAGCAGGCACAGACAGGCAGT#TGCCTGTGTGGACGCTGTATCCTGCTGC#.(((((((.(((((((((((..#))))))))).......))))..))))))).	1896	1924	1	-26	1	0.0008632	0.5	2	1.38	-5	18	28	7	3UTR
mmu-miR-214-3p	NM_025670	Cdip1	ACAGCAGGCACAGACAGGCAGT#CTGCTGTCTTGCCTGGCTT#..((((((((.(((((.((((.#)))))))))))))))).)).	2035	2054	0.92308	-23	1	0.0057751	0.4	0.2	0.4	-8	17	19	15	3UTR
mmu-miR-214-3p	NM_025695	Smc6	ACAGCAGGCACAGACAGGCAGT#GCGCCTGTCTGCAGGGCTCCCGCCTGCA#...((((((.(((((((((.((#))))))))))).........)))))).	252	280	0.89744	-26	0	1.10E-10	0.3	0.6	0.19	-8	17	28	11	5UTR
mmu-miR-214-3p	NM_025695	Smc6	ACAGCAGGCACAGACAGGCAGT#GCGTCTGTCTGCGCTTGA#....(((((.((((((((((.((#))))))))))).))))).	2486	2504	0.84615	-23	0	2.92E-06	0.5	1.1	1.59	-11	16	18	11	CDS
mmu-miR-214-3p	NM_025725	Ccdc96	ACAGCAGGCACAGACAGGCAGT#TTGCGCTATCTGGCCATGCTGG#.((((((((.((((.((((((.#)))).)).))))))).))))).	1003	1025	1	-24	1	4.74E-05	0.5	0.8	0.63	-6	18	22	7	CDS
mmu-miR-214-3p	NM_025725	Ccdc96	ACAGCAGGCACAGACAGGCAGT#ACATGCCGTACTTTGTTTGCTGA#.(((((((((.((((.((((((#)).)))))).)).))))))))).	2574	2597	1	-21	1	0.0232574	0.6	0.3	0.23	-7	19	23	9	3UTR
mmu-miR-214-3p	NM_025725	Ccdc96	ACAGCAGGCACAGACAGGCAGT#GTCTGTCTCCCTGTA#...(((((...((((((((...#))))))))).))))).	3511	3526	0.92308	-20	0	0.0155603	0.6	0.2	0.09	-12	13	15	8	3UTR
mmu-miR-214-3p	NM_025729	Tab3	ACAGCAGGCACAGACAGGCAGT#ACTGCCTGTGTTGCAGCACTACTGTATCTTTGTTGAGCGACCTAACAGTATTTCCCTGTTGTG#((((((((.((((((((((((#)))))))))).............))))...........................)))))))).	5861	5924	1	-23	1	0.001684	0.6	1.8	1.15	-5	21	63	9	3UTR
mmu-miR-214-3p	NM_025729	Tab3	ACAGCAGGCACAGACAGGCAGT#GCTCCCTGTGCCTCTTCCTGCTGC#.((((((((.....(((((.(((#))).))))).......))))))).	2863	2887	0.92308	-21	1	0.0011591	0.5	-0	1.18	-14	15	24	7	3UTR
mmu-miR-214-3p	NM_025729	Tab3	ACAGCAGGCACAGACAGGCAGT#TTGCAGTGTGTGTGTCTGTA#...(((((((((.((..(((((.#)))).)).))))))))).	5784	5802	0.96154	-22	0	0.0002653	0.6	1.1	0.89	-8	15	18	9	3UTR
mmu-miR-214-3p	NM_025768	Grtp1	ACAGCAGGCACAGACAGGCAGT#CTGCCTGTTTGTGGACATCCTACCTGTG#...(((((((((((((((((.#)))))))))))))).........))))).	819	847	1	-27	0	0.0014729	0.4	2.9	2.01	-7	18	28	13	CDS
mmu-miR-214-3p	NM_025770	Atg10	ACAGCAGGCACAGACAGGCAGT#TGTGTGTGTGTGTGTGTGTGTG#((((((.((((((.(((.(((..#))).))).))))).))).))).	1183	1205	0.84615	-22	0	0.28599	0.5	2.2	2.2	-7	17	22	5	3UTR

mmu-miR-214-3p	NM_025799	Fuca2	ACAGCAGGCACAGACAGGCAGT#CTGCCGCTGCTGCTGCTGC#.(((((.((((((.(.(((((.#))))))))).)))))))).	164	183	0.84615	-23	0	3.91E-05	0.3	1.1	0	-6	17	19	9	CDS
mmu-miR-214-3p	NM_025799	Fuca2	ACAGCAGGCACAGACAGGCAGT#GCCTAGGTCTGGCCCATGCTT#..(((((((.(((((((((...#))))..))))))))..)))).	5074	5095	0.84615	-23	1	7.58E-05	0.4	0.1	0.1	-6	16	21	8	3UTR
mmu-miR-214-3p	NM_025808	Lztr1	ACAGCAGGCACAGACAGGCAGT#GCCAGGGTCTGCTGGCAGATTTTCTAGCCTGCTGA#.((((((((.(((((.(((...#)))...)))))...............)))))))).	3119	3154	0.84615	-24	1	4.04E-05	0.5	0.1	0.26	-5	16	35	8	3UTR
mmu-miR-214-3p	NM_025812	Hmg20a	ACAGCAGGCACAGACAGGCAGT#GCTGCCCTAAAGGACAATGCTCCCTCCATCCTTCCTGGTCCTGCCTGCTGA#.(((((((((....((((.((.#.)).))))....))))))))).	2018	2069	1	-25	1	0.0143434	0.5	0.5	0.68	-12	15	22	9	3UTR
mmu-miR-214-3p	NM_025842	Vps28	ACAGCAGGCACAGACAGGCAGT#GCTGTATAGCTGCCTGGGCCTGCA#...((((((.(...((((((((#.)))))))))))))))).	105	129	0.92308	-25	0	1.12E-06	0.4	-0	0.17	-13	15	17	15	5UTR
mmu-miR-214-3p	NM_025852	Rexo1	ACAGCAGGCACAGACAGGCAGT#CTGCCCAACGTGACATCACCTCCTTTCCCAGTCCTGTCCCCTGGAGCCTGCTGC#.((((((((.((((((((....#.))))))...))..)))))))).	3989	4043	0.84615	-26	1	4.13E-05	0.3	1.1	0.07	-5	16	23	8	3UTR
mmu-miR-214-3p	NM_025852	Rexo1	ACAGCAGGCACAGACAGGCAGT#GCTGTGGGAACTCTGTGAGACAGCCCCAAGCTCCGGGTAGGCTGTGCCTGCTA#..((((((((((((.((.(((#.)))))..))...))))))))))).	4659	4712	0.92308	-25	1	2.72E-06	0.4	1.6	0.89	-5	18	25	11	3UTR
mmu-miR-214-3p	NM_025885	Med19	ACAGCAGGCACAGACAGGCAGT#ACTGGACTTGGCCAAGGATGGCTTTCCTGTGATACTGAACCTGCTGA#.(((((((..((((((((.(((#.)))..)))))....)))..))))))).	785	832	1	-22	1	0.1437259	0.5	0.3	1.81	-6	18	28	7	3UTR
mmu-miR-214-3p	NM_025900	Dek	ACAGCAGGCACAGACAGGCAGT#GCTGCCTGATAAAGTGACACTAGATGTACCAGCTGA#.((((.((.(((..((((((((#))))))))).................))).)).)))).	2381	2417	1	-21	0	0.0104964	0.6	0.6	0.36	-9	17	36	8	3UTR
mmu-miR-214-3p	NM_025929	Ly6m	ACAGCAGGCACAGACAGGCAGT#ATTGCATCTGTACCTGCTGA#.(((((((.(((((...(((((#))))).))))).))))))).	903	923	0.92308	-24	1	0.0013833	0.5	0.1	0.11	-6	17	20	7	3UTR

mmu-miR-214-3p	NM_025929	Ly6m	ACAGCAGGCACAGACAGGCAGT#ACTCTTGTCTGCAAGCTGGTCATCTGCTGG#.((((((((.((((((((.(((#)))))))))))...)).......))))	710	740	0.84615	-22	1	0.0001108	0.5	0.1	0.14	-5	19	30	11	3UTR
mmu-miR-214-3p	NM_025980	Nrarp	ACAGCAGGCACAGACAGGCAGT#GCCCTTTGTGCCGCCTGG#.(((..((((((((..(((...#))).))))))))..))).	2240	2258	1	-21	0	2.01E-05	0.5	1.5	1.33	-11	14	18	8	3UTR
mmu-miR-214-3p	NM_025980	Nrarp	ACAGCAGGCACAGACAGGCAGT#GGCTGCTGTGCTCTTTCTGCC#...((((((((((.(((.(...#).)))))))))).....)))).	1902	1923	0.92308	-20	0	0.0113443	0.4	-0	0.91	-7	14	21	9	3UTR
mmu-miR-214-3p	NM_025984	Lce1a1	ACAGCAGGCACAGACAGGCAGT#GCTGCCTTTTGTGTCCTGGGCTCAAAGATGCTGTA#((((((((((((((.(((((((#))))))))))))))).............)))))).	587	622	0.92308	-27	1	7.19E-05	0.6	1.5	0.28	-5	21	35	15	3UTR
mmu-miR-214-3p	NM_026079	Elp1	ACAGCAGGCACAGACAGGCAGT#GCCAGTGGAAGCTGAGCCTGCTGG#.((((((((.(((((.(((...#))).)).....))).)))))))).	4159	4183	0.84615	-25	1	0.0003115	0.4	1.4	1.72	-5	16	24	8	CDS
mmu-miR-214-3p	NM_026079	Elp1	ACAGCAGGCACAGACAGGCAGT#ACAGCACTGTTTCTGCCTGCA#...(((((((.((((((((.((#)).)).)))))).))))))).	5844	5865	1	-24	0	0.0004321	0.5	1.7	1.5	-9	17	21	7	3UTR
mmu-miR-214-3p	NM_026122	Hmgn3	ACAGCAGGCACAGACAGGCAGT#GGTGCTTTTGTGCCTA#.....(((((((((..((((.(#).))))))))))))).	802	818	0.84615	-17	0	2.07E-05	0.7	1.4	1.02	-15	14	16	13	3UTR
mmu-miR-214-3p	NM_026146	Eps8l1	ACAGCAGGCACAGACAGGCAGT#GCTCAGCCCGCGCTCTGAGGCCTCGGTGGTCCGTGCCTGGCTGC#.((((((((((.(((((((...#.)))).....))).))))))).))).	1928	1972	0.84615	-26	1	2.53E-06	0.3	0.6	1.27	-7	17	26	7	CDS
mmu-miR-214-3p	NM_026153	Ankrd33b	ACAGCAGGCACAGACAGGCAGT#GCCACAGTCTCGTGCTTTACTGA#.(((.((((((((((.(((...#)))...)))).))))))..))).	1017	1040	0.84615	-20	0	0.0011602	0.5	-0	-0.3	-8	16	23	6	3UTR
mmu-miR-214-3p	NM_026162	Plxdc2	ACAGCAGGCACAGACAGGCAGT#TTGCTAAGAAATTCTAGTTCTGTTTGTGCTTCTGG#.(((.(((((((((((((....#.))))))))))))))))	4816	4851	1	-23	0	0.0437957	0.7	0.1	0.07	-8	16	18	16	3UTR
mmu-miR-214-3p	NM_026162	Plxdc2	ACAGCAGGCACAGACAGGCAGT#TTGCCTGGACTCTCTCTTATTTGGAGTCACTTTGTGCCTCTT#..((.((((((((((((((((.#))))))).......................))))))))))).	6012	6054	0.84615	-21	0	0.0219147	0.5	-0	-0.2	-9	18	42	11	3UTR

mmu-miR-214-3p	NM_026177	Gpalpp1	ACAGCAGGCACAGACAGGCAGT#GCCTTGTGCTGCTGTA#((((((.(((((...((((...#))))))))))))))).	5153	5169	0.84615	-22	0	1.61E-06	0.5	1.1	1.63	-8	15	16	15	3UTR
mmu-miR-214-3p	NM_026217	Atg12	ACAGCAGGCACAGACAGGCAGT#CTGCCTAGCCTGGAACTTGCAGAGATCAGTGTGTCTGTCTATGCCTCCAAAGTGCTGA#.(((((((((.(((((((((..#.))))))))).))))))....	1704	1762	0.92308	-26	1	0.0002394	0.5	0.1	0.08	-7	18	28	9	3UTR
mmu-miR-214-3p	NM_026235	Larp6	ACAGCAGGCACAGACAGGCAGT#ACTGTCTGGTGGGCCATGGGTTTAGGATCGTCTTCTGGCTGGGGCTGTT#(((((.((((.(((((((((((#)))))))))).......................)))))))...))))	1719	1768	0.84615	-22	0	4.47E-06	0.4	0.2	0.83	-7	20	49	8	3UTR
mmu-miR-214-3p	NM_026235	Larp6	ACAGCAGGCACAGACAGGCAGT#ACTGAGTGTGTCTGTTCCCTGTC#...(((((.(((((((..((((#))))....)))))))..))))).	1994	2017	0.92308	-20	0	0.0012982	0.6	0.5	0.53	-8	16	23	7	3UTR
mmu-miR-214-3p	NM_026241	Ankrd39	ACAGCAGGCACAGACAGGCAGT#GCCTGTGGGACCTGCTGG#.(((((((..(..((((((...#)))))).)..))))))).	593	611	0.92308	-26	1	3.78E-05	0.3	0	-0	-11	14	18	7	CDS
mmu-miR-214-3p	NM_026241	Ankrd39	ACAGCAGGCACAGACAGGCAGT#GCCGTCCGGGATCGCAAAGCACGCCTAGCATGTGACCTGCTGC#.(((((((((((..(((((.((#.)))))).)..)))).))))))).	538	581	0.92308	-25	1	7.54E-05	0.4	-0	-0.1	-6	18	24	7	CDS
mmu-miR-214-3p	NM_026252	Cpeb4	ACAGCAGGCACAGACAGGCAGT#ACTGCTTGAGAACTAGCTTTGAGTAATACTTTTCCCTTTGTACACGGCCTGCTGA#.((((((((((((((((((((#))))))))))..........................)))))).....)))))))).	5491	5546	0.84615	-25	1	0.011255	0.6	4.5	4.26	-4	21	55	8	3UTR
mmu-miR-214-3p	NM_026252	Cpeb4	ACAGCAGGCACAGACAGGCAGT#GCTTGGGGTGTGCTATTGTGCTGG#.((((((((((((..(((((...#)))))...))))))....))))).	5425	5449	1	-23	1	0.000947	0.5	2.7	2.63	-6	16	24	6	3UTR
mmu-miR-214-3p	NM_026275	Ube2r2	ACAGCAGGCACAGACAGGCAGT#TTGTGCTGTTTGCCTGGTGTG#(((.((((((..(((((((((.#)))).))))))))))).))).	2859	2880	0.92308	-22	0	0.0006655	0.5	0.7	0.29	-13	18	21	11	3UTR
mmu-miR-214-3p	NM_026390	Ubxn4	ACAGCAGGCACAGACAGGCAGT#GCTGCCAAAGCTGCTGCCTTGCTGA#.(((((((((((...((((((#))))))....))).))))).)))).	1201	1226	1	-27	1	7.50E-06	0.5	1.9	2.43	-6	18	25	6	CDS
mmu-miR-214-3p	NM_026390	Ubxn4	ACAGCAGGCACAGACAGGCAGT#TGCCTTCTGTGGATGTAATTCCTGTTGA#.(((((((((((((.(((((..#))))))))))))).........))))))).	3029	3057	0.92308	-24	1	0.0005405	0.6	0.4	0.41	-5	18	28	11	3UTR

mmu-miR-214-3p	NM_026390	Ubxn4	ACAGCAGGCACAGACAGGCAGT#GCTGCCTGATGGTTCTTCCTTTACAAACCAGTTTCCTTCTGATGCTCCTCTAGAAGAAGCGAGGCAGTTTGCTGC#.(((((((((((((((((((((#)))))))))............................)))).)))).........................))))).	1302	1377	0.92308	-23	1	3.21E-05	0.5	3.5	2.77	-4	21	75	8	CDS
mmu-miR-214-3p	NM_026404	Slc35a4	ACAGCAGGCACAGACAGGCAGT#GCCTGTCCTTGAAGATCCCTGCCTTGTCCTGCATGTA#((((((((.((((((((((...#))))))))..................)))))))).)))).	2329	2366	0.92308	-24	1	0.0108874	0.5	-0	-0	-5	18	37	8	3UTR
mmu-miR-214-3p	NM_026422	Mrrf	ACAGCAGGCACAGACAGGCAGT#GCCTGCTGCACCCTGCTT#..((((((..(((.(((((...#))))))))...)))))).	355	373	1	-23	1	0.0051256	0.5	1.1	1.09	-8	14	18	8	CDS
mmu-miR-214-3p	NM_026422	Mrrf	ACAGCAGGCACAGACAGGCAGT#CCGAGTCCTGTGTCTGCTC#..((((((((((((.((....#))..))).)))))))))).	1142	1161	1	-22	1	0.0003361	0.4	-0	0.92	-5	15	19	10	3UTR
mmu-miR-214-3p	NM_026431	Cfap410	ACAGCAGGCACAGACAGGCAGT#GGTGCTCTGGCTGGCCGAGAACCCGTGCTGTG#(((((((((.(((.((((((.(#).))).))).))))))).........)))))).	324	356	0.92308	-22	1	0.0018425	0.3	4.3	2.6	-6	19	32	6	CDS
mmu-miR-214-3p	NM_026431	Cfap410	ACAGCAGGCACAGACAGGCAGT#ACATGCCAAAGAAGCCTGCTGG#.((((((((.......((((((#)).))))......)))))))).	1544	1566	0.92308	-21	1	8.98E-05	0.5	0.4	0.22	-14	14	22	8	3UTR
mmu-miR-214-3p	NM_026432	Saraf	ACAGCAGGCACAGACAGGCAGT#GCTGTTGCTCCTGCTGTCACTCCTGCTGG#.(((((((.((((.((((.(((#.)))))))))))))....))))))).	196	225	0.84615	-25	1	0.0030637	0.3	-0	0.3	-6	18	24	11	CDS
mmu-miR-214-3p	NM_026433	Tmem100	ACAGCAGGCACAGACAGGCAGT#ACTGCCTTTCTGTGAGCTTGCA#...((((((((((.(((((((#))))))).)))))..)))))).	518	540	1	-27	0	0.018533	0.5	0.4	0.31	-7	18	22	7	5UTR
mmu-miR-214-3p	NM_026451	1600012H06Rik	ACAGCAGGCACAGACAGGCAGT#GCTGCAGTCTGTCACTTGCG#...(((((.((((((..(((((#))))).))))))..))))).	442	462	0.84615	-22	0	0.0005046	0.4	2	2.11	-8	16	20	6	CDS
mmu-miR-214-3p	NM_026453	Mak16	ACAGCAGGCACAGACAGGCAGT#ACTGGCAGCTGTGCTCTTGTGTG#(((..(((((((((.(.(.((((#)))).).))))))).))..))).	947	970	1	-20	0	0.0018102	0.6	3.2	1.38	-12	17	23	7	3UTR

mmu-miR-214-3p	NM_026487	Atad1	ACAGCAGGCACAGACAGGCAGT#GCGGAGGAAACTGAGTGCAGCCTGAGTGTGGGTGCTTGTCTGACCTTTTTAGCCATATGCTGTT#(((((((((.((((((((((.(#.).)))))))))))).........)))...)))))).	1259	1323	1	-25	1	0.0050192	0.6	0.7	1.08	-5	20	35	10	3UTR
mmu-miR-214-3p	NM_026495	Nacc2	ACAGCAGGCACAGACAGGCAGT#GCTGCCTGTCTGCCCGGCTGTC#(((((.((..(((((((((((#))))))))))))).)).))))).	4967	4989	1	-32	0	0.0039843	0.5	3.1	3.79	-8	19	22	12	3UTR
mmu-miR-214-3p	NM_026499	Srsf6	ACAGCAGGCACAGACAGGCAGT#ACTGCATGCAACTGCTGGGCTTGCC#...(((((((.(((.((((((((#)))))......)))))).)))))).	2946	2971	1	-21	0	0.0002738	0.5	-0	0.23	-9	17	25	6	3UTR
mmu-miR-214-3p	NM_026499	Srsf6	ACAGCAGGCACAGACAGGCAGT#GCCTAGGTAGGTGTCTGTAGCTGG#.((((((((((..((((((...#)))))..))..))))))...)))).	2567	2591	1	-21	1	0.0004144	0.5	0.1	-0	-8	16	24	6	3UTR
mmu-miR-214-3p	NM_026501	Retreg3	ACAGCAGGCACAGACAGGCAGT#GCTGCCTTCTGTCCTCAGCTGG#.(((((((.(((((.((((((((#))))))))))))))))..)))).	854	876	1	-27	1	0.0003608	0.5	1.4	0.19	-6	19	22	15	CDS
mmu-miR-214-3p	NM_026501	Retreg3	ACAGCAGGCACAGACAGGCAGT#CTGCCTGCTGAGCTGTGGGGTACTGACCTTTTTGGCCATGTTGG#.((((((((.(((.((((((((.#)))))))))).......................))).)))))).	568	612	0.92308	-23	1	4.50E-05	0.4	0.7	0.65	-6	18	44	10	CDS
mmu-miR-214-3p	NM_026514	Cdc42ep3	ACAGCAGGCACAGACAGGCAGT#CTGCCTCCTGTGAGCTTGTG#...((((((((((..((((((.#)))))).)))))..)))))).	1124	1144	0.92308	-20	0	0.0062008	0.5	0.8	2.14	-8	16	20	6	CDS
mmu-miR-214-3p	NM_026522	Chid1	ACAGCAGGCACAGACAGGCAGT#ACTACTCACTGCTGCCTGCTGTG#(((((((((((((..((..(((#))).))..))).)))))))))).	1179	1202	0.84615	-23	1	6.57E-06	0.4	0.1	0.11	-6	18	23	10	3UTR
mmu-miR-214-3p	NM_026522	Chid1	ACAGCAGGCACAGACAGGCAGT#GCCTGGATTACTTCTATGACCTGCTGTA#((((((((((.((((((((...#)))))).......))).)).)))))))).	995	1023	0.84615	-25	1	0.0032276	0.4	-0	-0.1	-7	18	28	8	CDS
mmu-miR-214-3p	NM_026522	Chid1	ACAGCAGGCACAGACAGGCAGT#GCTTGCTCTGGTCTGTGGCTCTGTT#((((.((.((((((((((((((#))).)).))).)))))).)))))).	126	151	0.84615	-24	0	0.0008687	0.5	-1	-0.3	-10	20	25	6	CDS
mmu-miR-214-3p	NM_026523	Nmb	ACAGCAGGCACAGACAGGCAGT#GCCTGTTTGGCACAGCC#...((.(((.((((((((((...#)))))))))).).)).	68	85	0.92308	-21	0	6.76E-05	0.4	0	0.84	-10	14	17	11	5UTR

mmu-miR-214-3p	NM_026523	Nmb	ACAGCAGGCACAGACAGGCAGT#GTCTTTCCTGCTGTA#((((((((.(.((((.......#)))).))))))))).	624	639	0.92308	-19	1	0.0004857	0.6	-0	0.1	-8	13	15	9	3UTR
mmu-miR-214-3p	NM_026542	Slc25a39	ACAGCAGGCACAGACAGGCAGT#GCTGGCGCTCTCTGTGGCTGGGCTGG#.(((((((.((((((..(.((((#)))).)....)))))).))..)))).	774	800	0.92308	-23	1	8.33E-06	0.4	0.1	2.38	-7	17	26	6	CDS
mmu-miR-214-3p	NM_026542	Slc25a39	ACAGCAGGCACAGACAGGCAGT#ATGGTCTTCTGTGCACAGCTGTG#(((((.((((((((.((((.((#)).))))))))))).).))))).	1484	1507	1	-22	0	0.0002853	0.5	2.1	1.41	-7	19	23	11	3UTR
mmu-miR-214-3p	NM_026547	Lurap1	ACAGCAGGCACAGACAGGCAGT#ACTCTAGAGGCCTAGCCTGTGGCTCCTGTTGTA#(((((((((((((.(((((...#.)))).).)))))...)))))	1049	1082	1	-24	1	0.0031289	0.5	3.3	2.89	-5	18	25	8	3UTR
mmu-miR-214-3p	NM_026547	Lurap1	ACAGCAGGCACAGACAGGCAGT#ATTGCCTGTCATGATACATGAAGTGCTTCCTCTGTA#((((.((((((.((((((((((#))))))))))............))))))...)))).	1316	1352	0.92308	-23	0	0.0021024	0.5	0.2	1.01	-11	20	36	10	3UTR
mmu-miR-214-3p	NM_026609	Leprotl1	ACAGCAGGCACAGACAGGCAGT#ACTGCTTTCCATGGTGTCCTGCTGA#.((((((((((....(((((((#)))))))......))).))))))).	650	675	1	-24	1	0.0065869	0.6	0.9	0.63	-10	17	25	7	3UTR
mmu-miR-214-3p	NM_026609	Leprotl1	ACAGCAGGCACAGACAGGCAGT#ACTCCCTGTTGTGTTTGCC#...(((((((((.(((((.(((#))).)))))))))))))).	287	306	0.94359	-22	0	0.0003591	0.5	0.4	0.04	-8	17	19	14	CDS
mmu-miR-214-3p	NM_026609	Leprotl1	ACAGCAGGCACAGACAGGCAGT#TGCTTGGATGTGCCC#......((((((..((((((..#))))))..)))))).	103	118	0.92308	-22	0	0.0007469	0.5	1.4	1.34	-18	12	15	6	CDS
mmu-miR-214-3p	NM_026615	Urm1	ACAGCAGGCACAGACAGGCAGT#GCTGCCTCCTTCCCTGCTGTA#((((((((...((..(((((((#))))))).))..)))))))).	409	430	0.92308	-24	1	0.128754	0.4	0.6	1.35	-11	17	21	8	3UTR
mmu-miR-214-3p	NM_026615	Urm1	ACAGCAGGCACAGACAGGCAGT#GCTGCTTGCCACTCATGTGTCTTGGTGTA#(((.((((((((((((((((#))))))))....)).))))))).).)))	624	653	1	-24	0	0.0004854	0.5	0.1	0.06	-7	21	29	8	3UTR
mmu-miR-214-3p	NM_026615	Urm1	ACAGCAGGCACAGACAGGCAGT#GCCTAAGTAAGCCTGCTGA#.((((((((......((((...#))))......)))))))).	1502	1521	0.84615	-23	1	0.0038377	0.5	0.1	0.32	-14	12	19	8	3UTR
mmu-miR-214-3p	NM_026638	Cdipt	ACAGCAGGCACAGACAGGCAGT#CTGTCTGGGAACCCTGTGCTTCGGATCTACTATACCTCTAGGCCTGCTC#..((((((((.(((((((((((.#)))))))..........................)))..)))))))	678	727	0.84615	-22	1	0.0596888	0.5	0	0	-9	17	49	7	CDS

mmu-miR-214-3p	NM_026638	Cdipt	ACAGCAGGCACAGACAGGCAGT#ACTAGCCTGGCATGTGGTAGCTGC#.((((.(.((((..((((((((#))).)))))...)))).).)))).	1719	1743	0.84615	-22	0	0.0001282	0.6	0	0	-10	17	24	5	3UTR
mmu-miR-214-3p	NM_026656	Mcoln2	ACAGCAGGCACAGACAGGCAGT#ACTGCCCAAAGTTCTCCGGTTCTGTGCTTGCG#...(((((((((((..((((((#))))))).............))))	1335	1367	0.92308	-26	0	5.13E-05	0.4	4.3	4.01	-7	17	32	11	CDS
mmu-miR-214-3p	NM_026656	Mcoln2	ACAGCAGGCACAGACAGGCAGT#GCTGTCCTGCCTCTGCTGTCTGAGGA#.(..(((((((((((((((((((#)))).)))))..)))).)))))))..).	1734	1760	0.92308	-23	0	7.46E-06	0.5	1.5	0.98	-8	19	26	6	CDS
mmu-miR-214-3p	NM_026661	Aar2	ACAGCAGGCACAGACAGGCAGT#TTGCCTTTGTGTGCTTCTTGCTGG#.(((((((((((.(.((((((.#)))))).).))))))...))))).	1121	1145	1	-25	1	4.55E-05	0.5	3.7	3.32	-6	18	24	6	CDS
mmu-miR-214-3p	NM_026661	Aar2	ACAGCAGGCACAGACAGGCAGT#CTGCCTGCTCCTGGTTGGATGGAAGGAAACCTGCTGG#.(((((((..(((.((((((((.#))))))))...)))...............))))))).	1934	1971	1	-25	1	0.0024715	0.4	0.4	0.02	-7	17	37	7	3UTR
mmu-miR-214-3p	NM_026674	Aph1c	ACAGCAGGCACAGACAGGCAGT#GCTGCTGTGTGTGCTGTCTGA#.(((..(((((((.(((.(((((#)))))))))).)))))))..))).	1652	1673	0.84615	-25	0	0.0015389	0.5	-0	0.23	-11	17	21	8	3UTR
mmu-miR-214-3p	NM_026674	Aph1c	ACAGCAGGCACAGACAGGCAGT#GTCATGTCAGTGCCTCTGCTGG#.((((((((((.(((((((...#))).))))).)))))))..)))).	2455	2477	0.84615	-25	1	9.93E-05	0.6	-0	0	-7	17	22	6	3UTR
mmu-miR-214-3p	NM_026674	Aph1c	ACAGCAGGCACAGACAGGCAGT#GCTGCCGAAGCCTGAAATTCTGCCTGCTC#..((((((((((((...((((((#)))))))).....)))......))))))))).	811	840	0.84615	-24	1	0.0015989	0.5	0	0	-7	17	29	8	CDS
mmu-miR-214-3p	NM_026697	Rab14	ACAGCAGGCACAGACAGGCAGT#ACTGCCACCCGAGTGTCTGCA#...(((((((((.(...((((((#))))))...)..))))))))).	201	222	0.84615	-23	0	0.0183985	0.2	0.6	1.43	-12	15	21	8	5UTR
mmu-miR-214-3p	NM_026697	Rab14	ACAGCAGGCACAGACAGGCAGT#TGTCTGTTGTAGCCAGTAAGTTCATTGCTGTG#((((((((((((.((((((((..#)))))))))))).))).........	1418	1450	1	-22	1	8.97E-05	0.6	1.7	2.97	-5	19	32	10	3UTR
mmu-miR-214-3p	NM_026697	Rab14	ACAGCAGGCACAGACAGGCAGT#TTGCACTGTGGCATACCTGCTGA#.(((((((.....((((((((.#)))).))))......))))))).	1963	1986	1	-21	1	0.0013099	0.6	4.1	2.32	-14	15	23	7	3UTR
mmu-miR-214-3p	NM_026721	Slc39a13	ACAGCAGGCACAGACAGGCAGT#GCTGCCTGTCGCCTGG#....(((((...(((((((((((#)))))))))))))))).	298	314	0.92308	-27	0	0.0006521	0.4	0.2	-0.1	-17	15	16	15	CDS

mmu-miR-214-3p	NM_026721	Slc39a13	ACAGCAGGCACAGACAGGCAGT#GCATGCCTGGATGTCCCTGCC#...(((((.(((..((((((((#)).))))))..))).))))).	167	188	0.92308	-25	0	0.0006005	0.3	2.1	0.96	-10	16	21	6	5UTR
mmu-miR-214-3p	NM_026721	Slc39a13	ACAGCAGGCACAGACAGGCAGT#GCCTTCTGGGGGCCTGCTT#..(((((((.((((.((((...#))))))))...))))))).	1028	1047	1	-24	1	8.15E-08	0.4	0.3	0.25	-7	15	19	8	CDS
mmu-miR-214-3p	NM_026743	Tspan11	ACAGCAGGCACAGACAGGCAGT#GCCTGTCTTCAGATCTGTGGGATGGTCCTCACCTGCTGC#.(((((((((((((((((...#))))))))........)))............))))))).	958	997	0.92308	-25	1	7.83E-06	0.5	3	1.72	-4	18	39	8	CDS
mmu-miR-214-3p	NM_026756	Nfic	ACAGCAGGCACAGACAGGCAGT#GCCTGAGCACCATTGACTGCCTGCTGTT#((((((((((((.(((((...#)))))).......)))..)))))))))).	6062	6090	0.92308	-24	1	0.0361053	0.5	3.8	4.11	-5	18	28	10	3UTR
mmu-miR-214-3p	NM_026782	Ccdc167	ACAGCAGGCACAGACAGGCAGT#TGCAGCTGGGAGGGCAAGCTCTGTGCCTGCC#...(((((((((((..((...#.))..).))))))))))).	534	565	0.92308	-25	0	0.0002929	0.3	-0	-0.6	-7	14	19	11	3UTR
mmu-miR-214-3p	NM_026782	Ccdc167	ACAGCAGGCACAGACAGGCAGT#GCCTGCCCTGGCCTGTG#...((((((.(((.(((((...#)))))..))))))))).	578	595	1	-23	0	0.0010223	0.4	0.2	0.27	-9	14	17	9	3UTR
mmu-miR-214-3p	NM_026787	Eola1	ACAGCAGGCACAGACAGGCAGT#TTGCCTGAGGTTCACTTTACTCCTGCTGTT#((((((((...(((((((((.#)))))))........)))...)))))))	988	1018	0.84615	-21	1	0.0887605	0.7	-0	0.14	-9	18	30	8	3UTR
mmu-miR-214-3p	NM_026886	Srrm4	ACAGCAGGCACAGACAGGCAGT#ACGGGTGTCTGTGCCCCACCCTGGTTTTCTCTCTGCTGA#.(((((((((((((((......#.))))))))))).................))))).	6752	6791	0.92308	-23	1	0.0023324	0.6	2	1.87	-4	15	35	10	3UTR
mmu-miR-214-3p	NM_026965	Comtd1	ACAGCAGGCACAGACAGGCAGT#GCCTGGTGCTTTGCTGC#.((((((((((...(((((...#)))))))))))).))))).	1417	1434	0.84615	-23	1	3.05E-06	0.6	2.9	0.85	-9	15	17	11	3UTR
mmu-miR-214-3p	NM_026965	Comtd1	ACAGCAGGCACAGACAGGCAGT#ACTGTACCGCCTACTACGAGCGCTGTCTGCAGCTCCTACGTCCCGGAGGCGTGCTCGCTGTA#(((((.(((((((.((((((((#.))))))))..
.))................))))).))))).	606	668	0.84615	-23	0	4.46E-05	0.4	0.1	-0.1	-7	20	42	8	CDS
mmu-miR-214-3p	NM_027010	Crygf	ACAGCAGGCACAGACAGGCAGT#GCTGCAACTCTGTGCGAGTGGACAGTGGCTGCTGG#.(((((((((((((...(((((#)))))...)))))))............)))))).	130	165	0.84615	-26	1	3.13E-06	0.4	2.7	2.07	-5	18	35	7	CDS

mmu-miR-214-3p	NM_027011	Krt5	ACAGCAGGCACAGACAGGCAGT#CCTTTTTCTGGAGAGTAGTCTAGACCATGCCTGCTGC#.((((((((..(.(((((...#.)))).).)..)	1926	1963	1	-21	1	0.0148266	0.5	1.7	0.96	-11	15	21	9	3UTR
mmu-miR-214-3p	NM_027081	Dennd6b	ACAGCAGGCACAGACAGGCAGT#GCTTGTCCTGGTTACTGGTAGAGTATACCGCCTGCCTGCTGG#.(((((((((((((((((...#))))))).))......................))))))))).	4102	4144	1	-27	1	2.96E-05	0.4	3.4	2.82	-4	18	42	9	3UTR
mmu-miR-214-3p	NM_027081	Dennd6b	ACAGCAGGCACAGACAGGCAGT#GCTGCCAAGGGCTACTCCAGCTTTTCCCCCTGTGTCTGCC#...((((((((((...((((((#))))))......................)))))))))).	2543	2583	0.86538	-23	0	0.0370138	0.4	0.1	0.4	-8	16	40	10	3UTR
mmu-miR-214-3p	NM_027102	Esam	ACAGCAGGCACAGACAGGCAGT#GCTCTGAGCCGCATGGGTGCTGTGCCTGTGATGGTGCCTGCA#...((((((((..(((((((..#.)))))))....)))))))).	1248	1290	0.84615	-29	0	0.0004019	0.4	2.2	1.26	-11	15	21	8	CDS
mmu-miR-214-3p	NM_027102	Esam	ACAGCAGGCACAGACAGGCAGT#ATTGCCATGTCTGGAGTCTATGTCTGCA#...(((((((((((((((((((#)))))).))))))).......))))))).	786	814	0.88462	-26	0	0.0007653	0.5	1.7	1.28	-7	19	28	7	CDS
mmu-miR-214-3p	NM_027133	Lnpk	ACAGCAGGCACAGACAGGCAGT#CTGACCTTTGTGTGCCTGCTGTG#((((((((((((.(.((((((.#))).))).).)))))))))))).	6731	6754	1	-32	1	0.0030709	0.4	0.8	1.2	-6	19	23	12	3UTR
mmu-miR-214-3p	NM_027133	Lnpk	ACAGCAGGCACAGACAGGCAGT#CTGTCTGTATGTGGAGTGCCTGCTC#..((((((((((..((((((((.#))))))))).......)))))))))).	4276	4301	1	-27	1	0.0001787	0.6	0.8	-0	-10	17	25	9	3UTR
mmu-miR-214-3p	NM_027133	Lnpk	ACAGCAGGCACAGACAGGCAGT#GCCTGAGTTTGTGTCTGCA#...(((((((((((((((...#)))))..)))))))))))).	6866	6885	1	-27	0	0.000724	0.5	1.1	0.67	-7	16	19	12	3UTR
mmu-miR-214-3p	NM_027136	Ovca2	ACAGCAGGCACAGACAGGCAGT#GCTGCTTTCTGACTTCTGCCTCTGA#.((((.((((((((((.(((((((#))))))))))))).....)))))))).	1571	1596	0.92308	-22	0	0.0025518	0.6	-0	-0.1	-9	19	25	11	3UTR
mmu-miR-214-3p	NM_027136	Ovca2	ACAGCAGGCACAGACAGGCAGT#TGCCTTTCTCCTGCTGC#.(((((((...(((.(((((..#))))).)))))))))).	889	906	1	-22	1	0.0728361	0.5	0.1	0.04	-10	15	17	10	3UTR
mmu-miR-214-3p	NM_027144	Arhgef12	ACAGCAGGCACAGACAGGCAGT#GCCGCCTGCTTGAGCTTGCTCTGTGCCTGCTT#..(((((((((((((((((...#.))))).)))))))))))).	9761	9793	1	-31	1	0.0021418	0.4	4.2	4.23	-5	17	20	12	3UTR

mmu-miR-214-3p	NM_027144	Arhgef12	ACAGCAGGCACAGACAGGCAGT#CTGTCTGTCTGGTGCCAGGACTGG#.((((.(((((((((((((((.#)))))))))))).)))))..).))).	6386	6410	0.92308	-29	0	4.82E-07	0.4	1.7	1.54	-6	19	24	11	3UTR
mmu-miR-214-3p	NM_027192	Ttl	ACAGCAGGCACAGACAGGCAGT#AGTGCCTTAGTCTGTGCATGGTGC#.((.((.(((((((((((((.(#).)))))..)))))))).)).)).	1704	1728	0.84615	-26	0	2.36E-06	0.5	2	0.1	-8	18	24	8	3UTR
mmu-miR-214-3p	NM_027238	Ttc39b	ACAGCAGGCACAGACAGGCAGT#ACTACATTGTCCCTTTCAGCCTGTGAGCATGTTGGGCCTGCTGTT#(((((((((.((((((((((...#.)))))).......))).))))))))).	5551	5596	0.92308	-27	1	0.0002502	0.5	0.1	0.1	-5	18	28	9	3UTR
mmu-miR-214-3p	NM_027238	Ttc39b	ACAGCAGGCACAGACAGGCAGT#ACTGCGTGTCACTGGCTCGTGCTGTA#(((((((((.((((((.(((((#))))).)))))..)))))..)))))).	7647	7673	0.97436	-25	1	0.0008066	0.5	3.2	2.94	-5	20	26	6	3UTR
mmu-miR-214-3p	NM_027238	Ttc39b	ACAGCAGGCACAGACAGGCAGT#ACTGTCTGTAACTCCAGAAGAGCTGACACTCGCTCTGGCCTCTGTA#((((.((((.((((((((((((#))))))))).........................))))))))))))).	4143	4189	0.86538	-24	0	0.000551	0.5	0.7	0.31	-9	20	46	12	3UTR
mmu-miR-214-3p	NM_027257	Nabp2	ACAGCAGGCACAGACAGGCAGT#CTGCGGGTGGCTCGGCGGTGTGCACAGTCCGGCCTGCTGG#.((((((((.(.(((.((((..#.))).).))).))))))))).	71	111	0.84615	-24	1	4.12E-07	0.3	0.1	0.1	-9	16	21	9	5UTR
mmu-miR-214-3p	NM_027271	D3Ertd751e	ACAGCAGGCACAGACAGGCAGT#GGTGCCGGTCTAGGTGTCTGCC#...((((((((((((.((((.(#).)))).))))..)))))))).	2792	2814	0.92308	-25	0	2.04E-05	0.5	0	0.01	-7	17	22	8	3UTR
mmu-miR-214-3p	NM_027271	D3Ertd751e	ACAGCAGGCACAGACAGGCAGT#GCCAGTGTGTGTCAGGCTGA#.((((.((((((.((.(((...#))).)).))))))..)))).	239	259	1	-24	0	0.0001777	0.4	0.9	2.18	-7	15	20	6	CDS
mmu-miR-214-3p	NM_027355	Rnf168	ACAGCAGGCACAGACAGGCAGT#TGCCCTCTTCTGGCCTGTCTGA#.((((((((.((((..((((..#))))....))))))))).))).	2927	2949	0.92308	-23	1	4.95E-05	0.6	0	-0	-6	16	22	9	3UTR
mmu-miR-214-3p	NM_027375	Gcc2	ACAGCAGGCACAGACAGGCAGT#CTGCCTGTGATAGACACCATGCTGC#.(((((((.....((((((((.#))))))))........)).))))).	4942	4967	0.92308	-22	1	0.0193368	0.5	3.2	3.41	-14	15	25	8	CDS
mmu-miR-214-3p	NM_027402	Fndc5	ACAGCAGGCACAGACAGGCAGT#ACTATCTGGAACTGTGTCTGTC#...(((((((((((.((((.(((#))).))))...)))))))))).	1639	1661	0.84615	-21	0	2.13E-05	0.5	1	0.73	-7	17	22	10	3UTR

mmu-miR-214-3p	NM_027402	Fndc5	ACAGCAGGCACAGACAGGCAGT#CTGCCAAGGCCTCCATGTCTGCTTGCA#...((((((..((((((((((.#)))))..........))))))))))).	1852	1879	0.84615	-21	0	0.0029268	0.5	-0	0.51	-11	16	27	11	3UTR
mmu-miR-214-3p	NM_027413	Lrrc28	ACAGCAGGCACAGACAGGCAGT#GCCAATGCTGTGCCA#......(((((((.(((((...#)))..))))))))).	1236	1251	1	-19	0	0.0026706	0.6	0.5	0.53	-16	12	15	9	3UTR
mmu-miR-214-3p	NM_027453	Btf3l4	ACAGCAGGCACAGACAGGCAGT#ACTGCCTGCAACTCAAGCTCCAGGCGTCTGACATACTCTGCTGG#.(((((((..((((((((((((#)))))))).................)))))......))))))).	2900	2944	0.92308	-23	1	0.0241793	0.5	4.9	4.56	-6	19	44	7	3UTR
mmu-miR-214-3p	NM_027492	Naa30	ACAGCAGGCACAGACAGGCAGT#GCGTCTGCCAGTGCCTGTGTA#(((.(((((((.(.(((((.((#)))))))).).)))))))))).	1784	1805	0.84615	-21	0	0.0004244	0.6	3.4	2.51	-11	18	21	10	3UTR
mmu-miR-214-3p	NM_027496	Ankrd33b	ACAGCAGGCACAGACAGGCAGT#ACTGCCTGGATCATGGTCTGTGCTGCC#...(((.(((((((((((((((#))))))))........))))))))))).	5582	5609	0.88462	-27	0	0.0026289	0.4	-0	-0.7	-8	18	27	11	3UTR
mmu-miR-214-3p	NM_027496	Ankrd33b	ACAGCAGGCACAGACAGGCAGT#CTGGCTGTGGTGTGATTCCTGTTGTT#((((((((((((.((((.(((.#))).))))..))))...)))))))).	6569	6595	1	-25	1	0.0126794	0.6	2.8	3.38	-5	19	26	8	3UTR
mmu-miR-214-3p	NM_027496	Ankrd33b	ACAGCAGGCACAGACAGGCAGT#ACTTGGCCCATCATGTCCTTGATGGGCAGTGTGCTTGCTGC#.(((((((((((((((((((((#)))..))).....))))............))))))))))).	5076	5117	0.84615	-23	1	4.08E-07	0.4	-0	-0.2	-4	21	41	11	3UTR
mmu-miR-214-3p	NM_027498	Sik3	ACAGCAGGCACAGACAGGCAGT#ACAGCCTGCTGTGCGATCGACACAAGAAACATAAAACTCTGCG#...((((((((((.(((((.((#)).)))))))))))))......................)))).	1173	1216	1	-22	0	0.0624992	0.4	3.4	2.95	-7	17	43	11	CDS
mmu-miR-214-3p	NM_027498	Sik3	ACAGCAGGCACAGACAGGCAGT#ACTGTGCTGTTTGTGTGATCTCCATTCTTATTCTCTACTGCG#...((((((((((((((((((#))))).))))))))))).....................)))).	4961	5003	0.84615	-22	0	0.0636651	0.6	0.6	0.23	-7	19	42	10	3UTR
mmu-miR-214-3p	NM_027498	Sik3	ACAGCAGGCACAGACAGGCAGT#GCTGCCTGGCTTTCCTGGAGTA#((..((((.(.((.((((((((#)))))))).)).)))))..)).	1495	1517	0.92308	-22	0	0.0010677	0.5	2.3	3.06	-13	17	22	8	CDS
mmu-miR-214-3p	NM_027530	Rufy3	ACAGCAGGCACAGACAGGCAGT#ACTGTCTGTGTGCTTGCG#...((((((((..((((((((#)))))))))))))))).	2362	2380	0.92308	-26	0	0.0007071	0.4	0.3	0.28	-11	17	18	17	3UTR

mmu-miR-214-3p	NM_027530	Rufy3	ACAGCAGGCACAGACAGGCAGT#TGTGTGTGTGTGTGTTGTG#((((((.(((((.(((.(((..#))).))).))))).)))))).	589	610	0.92308	-26	0	0.0913976	0.5	2.5	2.67	-7	17	21	6	5UTR
mmu-miR-214-3p	NM_027530	Rufy3	ACAGCAGGCACAGACAGGCAGT#GCTGCTCCTGCGTGCTCCTGCTGTA#((((((((..((..((((((((#)))))..)))..))..)))))))).	3886	3911	0.84615	-25	1	1.19E-06	0.4	1.1	0.67	-8	18	25	8	3UTR
mmu-miR-214-3p	NM_027530	Rufy3	ACAGCAGGCACAGACAGGCAGT#CTGCCCTGCAGTCCTGCTGG#.(((((((.((.(.(((((((.#))))).))).))))))))).	2833	2853	1	-24	1	6.63E-05	0.4	0.5	0.42	-9	17	20	9	3UTR
mmu-miR-214-3p	NM_027604	Usp15	ACAGCAGGCACAGACAGGCAGT#GCTGCCTGTCTGCGCCATCCGGGTACTTAGCTGCTGTG#((((((((.((((((((((((#)))))))))))).)))..............)))))).	3473	3511	1	-35	1	0.0168787	0.4	0.1	0.06	-5	21	38	12	3UTR
mmu-miR-214-3p	NM_027642	Phf6	ACAGCAGGCACAGACAGGCAGT#ATTTCAGGTCTGTGTCTGCTT#..((((((((((((..(.(((#))).)..)))))))))))).	4396	4417	1	-24	1	0.0066615	0.6	0	0	-5	17	21	13	3UTR
mmu-miR-214-3p	NM_027712	Dlgap1	ACAGCAGGCACAGACAGGCAGT#CTGCTCTGGGACTTGCTGAATGTCACCTGAAGGCCCCTGTGTCTGCTT#..((((((((((...(((...#.))).))))))))))).	11	59	1	-24	1	0.0005504	0.5	0.3	0.48	-7	14	17	11	5UTR
mmu-miR-214-3p	NM_027712	Dlgap1	ACAGCAGGCACAGACAGGCAGT#GCTGCTGCATTCAGTCCTGCTGTT#((((((((.((.((..((((((#))))))....)).))))))))).	2970	2994	0.92308	-23	1	0.0018917	0.5	1.5	2.67	-9	18	24	10	CDS
mmu-miR-214-3p	NM_027808	Alpk1	ACAGCAGGCACAGACAGGCAGT#GCTGCCTTCAGTGCCTACACACCCCTCTTTGTGCTCACGGCTGTG#(((((((((((.((.(((((((#)))))))))).))))))).......................))))).	1332	1377	0.92308	-26	1	3.52E-06	0.5	1.3	2.39	-8	20	45	9	CDS
mmu-miR-214-3p	NM_027827	Afmid	ACAGCAGGCACAGACAGGCAGT#GCTGCCATGTGTGTGTATCTTAGCATACACACCACACATACATGATGATGGTGATAAAAAGAGTCCATGTTGTGCCTGCTGTA#(((((((((((((.(((((....#.)).))))))))))))))).	1697	1780	0.92308	-27	1	0.0006826	0.6	-0	0.03	-5	17	20	15	3UTR
mmu-miR-214-3p	NM_027854	Tmem248	ACAGCAGGCACAGACAGGCAGT#CTGTCTCAGTGTGTGCTTGCA#...((((((((((...((((((.#))))))...))))))))).	1691	1710	1	-24	0	0.0050568	0.7	3.1	0.98	-9	15	19	9	3UTR

mmu-miR-214-3p	NM_027889	Vps11	ACAGCAGGCACAGACAGGCAGT#GCTTTTCCTGCCTCCTCCTTTTGCCAGCCTGCTGTCCCTCCTGCTGA#(((((((((.((((.((((((.#.))))))......))))...))))))))).	2986	3033	1	-25	1	0.0002267	0.5	4.1	3.88	-6	19	30	9	3UTR
mmu-miR-214-3p	NM_027889	Vps11	ACAGCAGGCACAGACAGGCAGT#TTGCCTGTGGTCTTCCTGTCTGG#.(((((((.((..((((((((.#))))))))).))...)))).))).	3409	3432	0.84615	-22	1	0.0126491	0.5	1	2.15	-9	17	23	8	3UTR
mmu-miR-214-3p	NM_027892	Ppp1r12a	ACAGCAGGCACAGACAGGCAGT#AGTGCTGTCTGAAACTGTTGTG#(((((((...((((((.(((.(#).)))))))))...))))))).	4543	4565	1	-22	1	0.0037834	0.6	4	2.57	-9	17	22	9	3UTR
mmu-miR-214-3p	NM_027892	Ppp1r12a	ACAGCAGGCACAGACAGGCAGT#CTGTGAAGTCTGGATGCCTGA#....((((((((((((..((((.#))))...)))))..)))))).	5287	5308	0.87179	-20	0	0.0141159	0.6	3.1	2.62	-10	15	21	6	3UTR
mmu-miR-214-3p	NM_027931	Tars2	ACAGCAGGCACAGACAGGCAGT#ACTGGCTGATACTGCAGTGGCTGCTGA#.((((((.(((((.(((.((((#)))).)))...))...))).)))))).	423	450	0.84615	-24	1	0.0002132	0.5	0.5	1.69	-7	18	27	6	CDS
mmu-miR-214-3p	NM_027931	Tars2	ACAGCAGGCACAGACAGGCAGT#ACTGCCTGTGCGACTGGCTGATTTCGGAGCCCTGCA#...(((((..(((((((((((#))))))))))....)))..............))))).	1428	1464	0.84615	-22	0	0.0001458	0.4	2.9	3.12	-9	17	36	9	CDS
mmu-miR-214-3p	NM_027984	Epn3	ACAGCAGGCACAGACAGGCAGT#GCAGACCTGCCTGGCCAGCTGA#.((((.(((.....(((((((.#.)))))))))).)))).	4	26	0.87179	-23	0	6.17E-07	0.4	0	0	-15	14	17	10	5UTR
mmu-miR-214-3p	NM_028010	Apol6	ACAGCAGGCACAGACAGGCAGT#CTGCGTTCTTTCTTCTCAGGCTGTCCAGGTGCTGGCTGTC#(((((.(((((.(((((.(...#.).))))))...))))).))))).	1840	1880	0.92308	-24	0	1.40E-05	0.4	0.2	-0.1	-9	16	23	5	3UTR
mmu-miR-214-3p	NM_028013	Endod1	ACAGCAGGCACAGACAGGCAGT#GCTGTCTGCTGAAGGCTGTCTGCC#...((((((((((.((((((((#))))))))))).....))))))).	1524	1548	0.84615	-24	0	2.12E-05	0.4	0.1	0.12	-7	18	24	11	CDS
mmu-miR-214-3p	NM_028013	Endod1	ACAGCAGGCACAGACAGGCAGT#ATTCGTCTGGCTGGCAGCTTGCTGTG#(((((((((.(((.((((((((#))).))))).)))...)))))))).	922	948	0.84615	-24	1	6.21E-05	0.4	0.2	-0.1	-6	20	26	9	CDS
mmu-miR-214-3p	NM_028082	Cnot2	ACAGCAGGCACAGACAGGCAGT#TTGGAAAATGTTTGCCCTGCTGTA#(((((((..((((((......#.)))))).)))))))).	2380	2404	1	-20	1	0.0123811	0.6	4	4.09	-6	14	17	8	3UTR

mmu-miR-214-3p	NM_028108	Naa50	ACAGCAGGCACAGACAGGCAGT#GCTTCCACTCTGTGCCTACAATGTTGA#.(((((((((((((..((.(((#))).))..))))))))).....)))).	1954	1981	0.84615	-22	1	0.0282815	0.6	0.7	0.95	-5	18	27	9	3UTR
mmu-miR-214-3p	NM_028108	Naa50	ACAGCAGGCACAGACAGGCAGT#CTGTGGTACTTTGAGTTATACAGACTGTCTGTGGGGTGCTA#......(((((..(((((((((#.)))))))))...))))).	1232	1273	1	-21	0	0.0001437	0.5	1.3	1.06	-20	14	19	9	3UTR
mmu-miR-214-3p	NM_028108	Naa50	ACAGCAGGCACAGACAGGCAGT#AGTGATCTGTCTGTAACTGTC#...((((..((((((((((((.(#).)).)))))))))..)))).	3636	3657	1	-20	0	0.0056227	0.7	1.1	1.3	-10	16	21	9	3UTR
mmu-miR-214-3p	NM_028130	Zfp157	ACAGCAGGCACAGACAGGCAGT#TGTGTGTGTGTGTGTGTGTGTG#((((((.(((((.(((.(((..#))).))).))))).))).))).	4147	4169	0.84615	-22	0	0.0398472	0.6	0.2	0.26	-7	17	22	5	3UTR
mmu-miR-214-3p	NM_028149	Fbxl20	ACAGCAGGCACAGACAGGCAGT#CTGCCTGTCTGAGTTACTTGAGTTACTTGCTTTGCAGCAGTGCTGG#.((((((((((((((((((((.#))))))))))))................))))..........))))).	2836	2882	0.92308	-28	1	6.62E-06	0.4	0.1	0.49	-4	20	46	11	3UTR
mmu-miR-214-3p	NM_028149	Fbxl20	ACAGCAGGCACAGACAGGCAGT#CTGCCTGAATCTTTGCCAGTCTGG#.((((.((((.((((((((((.#))))))))..))).)))).).))).	2477	2501	0.92308	-24	0	0.0063664	0.6	0.4	0.2	-9	18	24	7	3UTR
mmu-miR-214-3p	NM_028149	Fbxl20	ACAGCAGGCACAGACAGGCAGT#ACTGGCTGCTGTAACCCTGCCTTATTCTTTTTACCTGCTGG#.(((((((.((((.(((.((((#))))).)))))))....................))))))).	6745	6786	0.92308	-24	1	0.0103252	0.5	0.9	1.2	-6	18	41	7	3UTR
mmu-miR-214-3p	NM_028149	Fbxl20	ACAGCAGGCACAGACAGGCAGT#GCTGTCTGTCGCCATCTGGGTGTCCATAGGAAGGCTTGCTC#..(((((((...((((((((((#))))))))))......................))))))).	7645	7686	0.84615	-24	1	0.000726	0.6	4.4	3.98	-12	17	41	10	3UTR
mmu-miR-214-3p	NM_028162	Tbc1d5	ACAGCAGGCACAGACAGGCAGT#ACTGTCTCCACTCTATGCAATGTATAAGTCTGTGTCTGA#....((((((((((((((((((#))))))))....................)))))))))).	331	370	0.84615	-24	0	0.0002919	0.6	-0	-0.2	-10	18	39	11	5UTR
mmu-miR-214-3p	NM_028162	Tbc1d5	ACAGCAGGCACAGACAGGCAGT#ACTGATCCATCCAGGTCTGGGACAGTGCCGGCCATGTCTGTCACTGCTGA#.((((((..(((((((((.((#.))..))).)))))))..)))))).	4256	4306	0.80769	-27	1	0.0003899	0.4	4.2	4.2	-7	18	25	7	3UTR

mmu-miR-214-3p	NM_028166	1600014C10Rik	ACAGCAGGCACAGACAGGCAGT#GCTGTGTGTGCTATGGAAGGGTACAGGCGTGCCTGCTGTA#(((((((((((((((.(((((#)))))).)))).))...............)))))))))).	734	774	0.92308	-30	1	6.31E-07	0.4	0	0	-4	21	40	11	3UTR
mmu-miR-214-3p	NM_028166	1600014C10Rik	ACAGCAGGCACAGACAGGCAGT#GCCTGTTAGGTGCCTGG#....(((((((.(((((((...#))))))))..))))))).	299	316	0.89744	-27	0	1.84E-05	0.4	0	0	-13	14	17	7	CDS
mmu-miR-214-3p	NM_028166	1600014C10Rik	ACAGCAGGCACAGACAGGCAGT#CTCCTGTCTGCTGGCTGCA#...((((.((((((((((.((.#)))))))))).)).)))).	1391	1410	0.92308	-25	0	0.0031527	0.6	0	0	-9	16	19	10	3UTR
mmu-miR-214-3p	NM_028181	Ccpg1	ACAGCAGGCACAGACAGGCAGT#GCTGGCTTGTCTTGTGAACTCTCTTGCA#...((((((((((((((((((#)))).))))))).)))......))))).	2657	2685	1	-21	0	0.0078911	0.6	2.1	1.78	-7	19	28	7	3UTR
mmu-miR-214-3p	NM_028203	Wdr89	ACAGCAGGCACAGACAGGCAGT#ATTGCTCAGGGTGTTGGCAAGCTGGCCAGTGTGCCTGCTGG#.(((((((((((....((((((#.)))).))..)))))))))).	2418	2459	1	-29	1	1.30E-06	0.4	1.4	2.95	-7	17	22	11	3UTR
mmu-miR-214-3p	NM_028203	Wdr89	ACAGCAGGCACAGACAGGCAGT#ACTGCCTCTGTGTGTGTGTGTGTGTG#((((((.(((((.(((((((((#)))))))..)).))))).))).))).	2758	2784	1	-26	0	0.1819393	0.5	1.1	2.1	-7	20	26	7	3UTR
mmu-miR-214-3p	NM_028216	Psca	ACAGCAGGCACAGACAGGCAGT#GCTGACCGTGCTCTGCAGCCTGTTGC#.((((((((.((((((((((((#)))).)).)).))))..)))))))).	339	365	0.92308	-25	1	6.55E-05	0.3	-0	0.31	-5	20	26	8	CDS
mmu-miR-214-3p	NM_028292	Ppme1	ACAGCAGGCACAGACAGGCAGT#AGTGTGTGTTTCCTGGCTGCTAGTGACCTGCTGTC#(((((((((((((.(((.(((.#.))).)))))).))	1211	1246	0.96154	-28	1	0.0031237	0.5	4.5	4.41	-5	19	24	8	CDS
mmu-miR-214-3p	NM_028310	2810006K23Rik	ACAGCAGGCACAGACAGGCAGT#GTCTCCTTGTAGCCTGCTGC#.((((((((((((..((((...#))))..)))).)))))))).	682	702	0.97436	-23	1	0.0052862	0.4	0	0	-5	16	20	8	3UTR
mmu-miR-214-3p	NM_028310	2810006K23Rik	ACAGCAGGCACAGACAGGCAGT#CTGCCCAGTCTCAGGAGCCTGGTGC#.((.(((((.(((((.(((((.#)))))..))))..)..))))).)).	601	626	0.92308	-23	0	0.0001117	0.5	0.2	-0.1	-8	17	25	5	3UTR
mmu-miR-214-3p	NM_028328	Rprd2	ACAGCAGGCACAGACAGGCAGT#CTGATCCTGTCGTCCTGCTGC#.(((((((.(((.((((((((((.#)))..)))))))))))))))).	3244	3265	1	-26	1	0.0033513	0.4	2.1	2.45	-8	18	21	15	3UTR

mmu-miR-214-3p	NM_028328	Rprd2	ACAGCAGGCACAGACAGGCAGT#ATTGCCAGCACCCACACAACAACCAGGCTTAGCTGTGTGTGCCTGTA#...(((((((((.((((..(((#.)))...)))).))))))))).	2181	2228	1	-25	0	0.0014227	0.5	2.9	2.46	-8	16	22	9	3UTR
mmu-miR-214-3p	NM_028328	Rprd2	ACAGCAGGCACAGACAGGCAGT#GCCTGTGGAGAAACCTGCTGTC#((((((((.....((((((...#))))))........)))))))).	1374	1396	1	-24	1	0.0001424	0.5	0.4	-0.1	-14	14	22	8	CDS
mmu-miR-214-3p	NM_028355	Ndc1	ACAGCAGGCACAGACAGGCAGT#GCCTGGCTAGATAACAGAATTAAAGCCATAGGCCTTTTGTGTTTGCTGG#.(((((((((((((.((((...#.))))))))))))))))).	3522	3571	1	-26	1	0.0050775	0.5	0	0	-5	17	19	17	3UTR
mmu-miR-214-3p	NM_028355	Ndc1	ACAGCAGGCACAGACAGGCAGT#CCATGTCTGCCTGCTGG#.((((((((..(((((((....#)).))))))))))))).	3801	3818	0.92308	-24	1	5.64E-05	0.4	0	0	-8	15	17	13	3UTR
mmu-miR-214-3p	NM_028376	Pfn4	ACAGCAGGCACAGACAGGCAGT#TGCCAGTCTCATGCCTCCTT#..((.(((((.((((.((((..#)))).)))))..))))).)).	1727	1747	0.84615	-19	0	0.0390663	0.5	0.2	0.13	-12	15	20	5	3UTR
mmu-miR-214-3p	NM_028385	Setd5	ACAGCAGGCACAGACAGGCAGT#AGTGCATTGTGCCTGTTGTA#((((((((((((.....(((.(#).)))..)))))))))))).	5601	5621	0.92308	-23	1	0.0006783	0.4	1.8	1.49	-7	16	20	12	3UTR
mmu-miR-214-3p	NM_028462	Fezf1	ACAGCAGGCACAGACAGGCAGT#CTGCCGTCTCAAAATGCCTGCTGA#.(((((((((.((((.(((((.#))))))))))).....))))))))).	2036	2060	0.92308	-29	1	0.019224	0.5	1.7	2.9	-7	18	24	9	3UTR
mmu-miR-214-3p	NM_028466	C9orf72	ACAGCAGGCACAGACAGGCAGT#CTGCCCCTCTGTGTTCTGTGGTA#((.((((((((((((..(((((.#)))))..))))))).)))).)).	1442	1465	0.92308	-25	0	0.0022271	0.5	0.5	0.61	-6	18	23	7	3UTR
mmu-miR-214-3p	NM_028466	C9orf72	ACAGCAGGCACAGACAGGCAGT#CTGCCTGTACTCTGGTGTTTCCCGTGTCACCTGCA#...((((((((((((((((((.#))))))))).))...)))............))))).	2014	2049	0.84615	-22	0	0.0004624	0.5	3.2	3.32	-7	18	35	8	3UTR
mmu-miR-214-3p	NM_028475	Ubap2l	ACAGCAGGCACAGACAGGCAGT#CTTCCAGTATGGGCCTGCTGTG#(((((((((.((.((.((.((.#)).)).)).)).))))))))).	2959	2981	0.92308	-23	1	5.14E-06	0.4	3.4	3.17	-7	17	22	9	CDS
mmu-miR-214-3p	NM_028572	Vgll3	ACAGCAGGCACAGACAGGCAGT#GTCTGTGATGCGCTGTGTCTGTG#...((((((((((((((((...#))))))......)))))))))).	1970	1993	0.92308	-22	0	6.03E-05	0.5	1.3	1.84	-7	16	23	10	3UTR

mmu-miR-214-3p	NM_028597	Thoc3	ACAGCAGGCACAGACAGGCAGT#ACTGCCAGCGTGTTCTTGCTGG#.(((((((.((((..(.((((((#)))))).)..))).))))))).	264	286	0.92308	-25	1	2.02E-05	0.4	0.1	0.1	-7	17	22	7	CDS
mmu-miR-214-3p	NM_028597	Thoc3	ACAGCAGGCACAGACAGGCAGT#CTGCCTAAGAACAGAACTGATGTGGAACATCTGTTTGTCTGTGTATATATCTGTG#((((...((((((((((((((.#.))))))))))))))......)))).	1621	1676	1	-25	0	0.0312101	0.6	4.3	4.45	-12	18	26	14	3UTR
mmu-miR-214-3p	NM_028614	Ppp2r1b	ACAGCAGGCACAGACAGGCAGT#TGCACTGTCTGAAGCCTGTGGTA#((.((((((.((((((((((..#))).)))))))..)))))).)).	1614	1637	1	-26	0	4.26E-05	0.5	3.5	3.95	-7	18	23	7	CDS
mmu-miR-214-3p	NM_028614	Ppp2r1b	ACAGCAGGCACAGACAGGCAGT#GCTGCTGGCTGGCCAGCTGG#.((((.(((.(((.(.((((((#)))))).))))))).)))).	1380	1400	0.84615	-25	0	8.14E-07	0.5	2.4	4.37	-8	17	20	7	CDS
mmu-miR-214-3p	NM_028623	Cst6	ACAGCAGGCACAGACAGGCAGT#GGTGTCTGTGTGTAGCTCTGGCTGTT#((((((((((((.(((((((.(#).))))))).))).)).))).)))).	3643	3669	0.92308	-25	1	5.41E-05	0.5	0.9	-0.2	-5	20	26	7	3UTR
mmu-miR-214-3p	NM_028623	Cst6	ACAGCAGGCACAGACAGGCAGT#GCTGCCTGGAACAAACCAATGGTGTGTGTGGGTGTTGG#.(((((.(((((..((((((((#))))))))................)))))...))))).	2905	2943	0.92308	-22	0	5.29E-06	0.5	0	-0.4	-7	18	38	8	3UTR
mmu-miR-214-3p	NM_028625	Lce1a2	ACAGCAGGCACAGACAGGCAGT#AGTGCCCCCCTGTGTCTTCCTGCTGTA#((((((((((((...((((.(#).))))...)))))....)))))))).	180	207	1	-25	1	0.0275118	0.4	0.5	-0.4	-6	18	27	8	CDS
mmu-miR-214-3p	NM_028625	Lce1a2	ACAGCAGGCACAGACAGGCAGT#GCTGCTCTCTGTGTCCTGGGCTCAAAGATGCTGTA#((((((((((((((..((((((#)))))).)))))))).............)))))).	604	639	0.92308	-25	1	2.47E-06	0.5	1.5	0.25	-5	20	35	8	3UTR
mmu-miR-214-3p	NM_028667	D3Ertd751e	ACAGCAGGCACAGACAGGCAGT#GGTGCCGGTCTAGGTGTCTGCC#...((((((((((((.((((.(#).)))).))))..)))))))).	3044	3066	0.92308	-25	0	2.04E-05	0.5	0	0.01	-7	17	22	8	3UTR
mmu-miR-214-3p	NM_028667	D3Ertd751e	ACAGCAGGCACAGACAGGCAGT#GCCAGTGTGTGTCAGGCTGA#.((((.((((((.((.(((...#))).)).))))))..)))).	239	259	1	-24	0	0.0001777	0.4	0.9	2.18	-7	15	20	6	CDS
mmu-miR-214-3p	NM_028677	Ppih	ACAGCAGGCACAGACAGGCAGT#GTCTGACAAGCTGGACTGGCCTCTGTGTTTGCTT#..(((((((((((((((.((((#.))))..)))...))))))	638	672	0.84615	-24	1	0.0021521	0.4	3.5	3.13	-5	19	26	12	3UTR

mmu-miR-214-3p	NM_028709	Btbd11	ACAGCAGGCACAGACAGGCAGT#CTGTCTGTCTGTACTGGGCCAAGGAGCTGC#.((((.((((((((((((((.#)))))))))))))).....))).....))	3935	3965	0.92308	-26	0	3.40E-05	0.4	0.7	0.41	-6	19	30	12	3UTR
mmu-miR-214-3p	NM_028715	Fcho1	ACAGCAGGCACAGACAGGCAGT#GCGCCACGCTGTCTGGCCTGGACCTGG#....((((..(((.((((((((#.))))))))..)))..)))).	2634	2661	0.84615	-23	0	0.000171	0.3	2.8	2.03	-13	15	21	8	CDS
mmu-miR-214-3p	NM_028715	Fcho1	ACAGCAGGCACAGACAGGCAGT#GCTGCCTATTCCTGCTGA#.(((((((....((.(((((((#))))))).)))))))))).	2999	3017	0.92308	-22	1	0.0006446	0.4	2.8	2.59	-13	16	18	9	3UTR
mmu-miR-214-3p	NM_028718	Traf3ip1	ACAGCAGGCACAGACAGGCAGT#ACCGCCCCTCTGAGCCCCAGTCTGCTGAGAAATGGCCTGCTGTG#(((((((((.(((((.(((...#.)))...)))))..........))))))))).	2031	2075	0.84615	-26	1	0.0005687	0.4	-1	-0.6	-5	17	32	9	3UTR
mmu-miR-214-3p	NM_028816	Xpo6	ACAGCAGGCACAGACAGGCAGT#AGTGCCTGCTTGCCTTCCTGGCTGCTCACTGCCCCAGGGATGTGCCTGCTGC#.((((((((((((..(.((((((#.))))))....)..)))))))))))).	3976	4028	0.92308	-32	1	1.05E-05	0.4	1	1.62	-6	18	26	11	3UTR
mmu-miR-214-3p	NM_028846	Usp20	ACAGCAGGCACAGACAGGCAGT#GCTGCCCGTGTTTGTTCTGTTGA#.(((((((.(((((((((((((#))))))..)))))))))))))).	3481	3504	0.92308	-26	1	0.043367	0.5	-0	-0	-5	20	23	14	3UTR
mmu-miR-214-3p	NM_028846	Usp20	ACAGCAGGCACAGACAGGCAGT#GTCTGTGTCCTGGACCTGCTGA#.(((((((..((((((((...#))))))...)))..))))))).	3761	3783	0.92308	-26	1	0.0003924	0.5	-0	-0.1	-6	16	22	7	3UTR
mmu-miR-214-3p	NM_028860	Mtmr3	ACAGCAGGCACAGACAGGCAGT#GCTGCCTGAGTGGGCACATGTCTGACTCCTGTT#(((((((((((((..((((((((#))))))))..))......))))))....)))).	4746	4779	0.92308	-23	1	0.0103716	0.5	4.3	4.09	-6	20	33	8	3UTR
mmu-miR-214-3p	NM_028860	Mtmr3	ACAGCAGGCACAGACAGGCAGT#GCGCGCTCTGTGTGGCCGCTGC#.((((.((((.((.((((((.((#))..)).)))).))))))))).	14	36	0.84615	-22	0	6.04E-07	0.4	2.3	2.2	-8	17	22	9	5UTR
mmu-miR-214-3p	NM_028862	Rnf145	ACAGCAGGCACAGACAGGCAGT#CTGCCCTCTGTGTCACTGCC#...((((((((((((..((((((.#)))))).))))))))..)))).	2060	2080	0.92308	-23	0	0.0919778	0.5	3.9	2.86	-7	16	20	7	CDS
mmu-miR-214-3p	NM_028862	Rnf145	ACAGCAGGCACAGACAGGCAGT#CTGCTTGTTCCTCCTCGTGTTCCCTGCG#...((((((((((((((((((.#)))))))).....)).)))...))))).	1505	1533	0.84615	-20	0	0.0166945	0.5	3.6	4.82	-7	18	28	8	CDS

mmu-miR-214-3p	NM_028862	Rnf145	ACAGCAGGCACAGACAGGCAGT#ACTTATCGTCTGCTGGAGTTTGTTGTG#(((((((((.((((.((((((((#)))....))))))))..))))))))).	1719	1746	0.88462	-20	1	0.0070121	0.4	4.2	4.3	-6	20	27	9	CDS
mmu-miR-214-3p	NM_028933	Retreg3	ACAGCAGGCACAGACAGGCAGT#GCTGCCTTCTGTCCTCAGCTGG#.(((((((.(((((.((((((((#))))))))))))))))..)))).	727	749	1	-27	1	0.0003608	0.5	3.3	0.91	-6	19	22	15	CDS
mmu-miR-214-3p	NM_028933	Retreg3	ACAGCAGGCACAGACAGGCAGT#CTGCCTGCTGAGCTGTGGGGTACTGACCTTTTTGGCCATGTTGG#.((((((((.(((.((((((((.#)))))))))))......................))).))))).	441	485	0.92308	-23	1	0.0004771	0.4	0.4	0.43	-6	18	44	10	5UTR
mmu-miR-214-3p	NM_028964	Snx29	ACAGCAGGCACAGACAGGCAGT#GGTGACCTGTCTGTGTGCC#...((..(((((((((((((.(#).)).))))))))))))).	190	209	0.92308	-22	0	1.13E-05	0.4	3.7	3.06	-10	16	19	13	CDS
mmu-miR-214-3p	NM_028964	Snx29	ACAGCAGGCACAGACAGGCAGT#CTGCCTCAGTGCCTGA#....(((((((....((((((.#))))))..))))))).	1446	1462	0.92308	-22	0	0.0002215	0.4	1.5	2.18	-16	13	16	7	CDS
mmu-miR-214-3p	NM_028973	Lrrc15	ACAGCAGGCACAGACAGGCAGT#GCTGCCTGTATCTGCTGCTGCTGCTGC#.((((((((((((((((((((#))))))))..)))).)))..)))))).	1749	1776	0.92308	-30	1	8.94E-06	0.4	-0	-0.3	-4	21	27	8	CDS
mmu-miR-214-3p	NM_028973	Lrrc15	ACAGCAGGCACAGACAGGCAGT#CTGCTGGCTGTGGCTTGCTGG#.((((((((((((.(.(((((.#))))).)))))).))))))).	3952	3973	0.92308	-27	1	5.75E-06	0.4	1.8	2.05	-5	18	21	7	3UTR
mmu-miR-214-3p	NM_028975	Tmem33	ACAGCAGGCACAGACAGGCAGT#CTGCACCATAACAGAGAAGTCTGTCTGCGTCTTATGACCTGTGAAGCTGTT#(((((((((.(((((((((...#.))))))))).))))............))))).	1512	1563	0.92308	-25	1	0.0038065	0.5	0.3	0.54	-5	18	34	9	3UTR
mmu-miR-214-3p	NM_028975	Tmem33	ACAGCAGGCACAGACAGGCAGT#GCTGCCACTACCTGCTGTA#((((((((...((...((((((#)))))).)).)))))))).	541	560	1	-23	1	0.018122	0.5	3.4	4.02	-11	16	19	8	CDS
mmu-miR-214-3p	NM_028975	Tmem33	ACAGCAGGCACAGACAGGCAGT#GCTGCTTTCTAGGGCAGGTGTGTTTAGCCTGCA#...(((((((((.(((((((((#)))))))..........)).)	1484	1517	0.92308	-22	0	8.39E-05	0.5	0.9	0.56	-8	18	33	7	3UTR
mmu-miR-214-3p	NM_028999	Ppp6r3	ACAGCAGGCACAGACAGGCAGT#ATTGCCCTGTTTGAAGCATGTTGTA#((((((.((.(((((((((((((#)))))).))))))..)).)))))).	2054	2079	0.92308	-22	0	0.0026079	0.6	1.2	1.07	-7	20	25	6	CDS
mmu-miR-214-3p	NM_029004	Rasgef1c	ACAGCAGGCACAGACAGGCAGT#CTTGTTGAGTGCCATGTGCTGTG#(((((((((((.((((((....#))))))..)))))...)))))).	1873	1896	1	-22	1	0.0003247	0.5	-0	-0	-7	17	23	6	3UTR

mmu-miR-214-3p	NM_029037	Pomk	ACAGCAGGCACAGACAGGCAGT#GTCTGCTCTGGGCTTGCATGC#.((((((((.(((((((((...#))))).)))).)))))).)).	2604	2625	1	-23	1	1.32E-05	0.5	0.5	3.11	-5	17	21	6	3UTR
mmu-miR-214-3p	NM_029037	Pomk	ACAGCAGGCACAGACAGGCAGT#GCTGCTGCTGCTGCTGCTGC#.(((((.((((((.((.(((((#)))))))))).)))))))).	2953	2973	1	-23	0	5.66E-05	0.5	3.1	2.14	-6	18	20	10	3UTR
mmu-miR-214-3p	NM_029037	Pomk	ACAGCAGGCACAGACAGGCAGT#ACTGTCTGCTGCTGGCAAAACTACACCCCTGCTA#..(((((((((((.((((((((#)))))))))))).))..............)))))).	2281	2315	0.92308	-22	1	0.0032123	0.5	1.1	1.19	-6	19	34	11	3UTR
mmu-miR-214-3p	NM_029094	Pik3cb	ACAGCAGGCACAGACAGGCAGT#CTGCCGCACTTGCCTGTTCGTT#((((((((((.((...(((((.#)))))...)))))))))).)).	3556	3578	1	-22	1	0.0200788	0.5	3.5	2.34	-9	17	22	10	3UTR
mmu-miR-214-3p	NM_029094	Pik3cb	ACAGCAGGCACAGACAGGCAGT#TGTTTGCATGTGTGAATCAAACTGCTGTA#(((((((((((((..((((((..#))))))..)))))........))))))).	565	594	0.92308	-21	1	0.0115582	0.6	0.9	1.21	-6	18	29	7	CDS
mmu-miR-214-3p	NM_029128	Qtrt2	ACAGCAGGCACAGACAGGCAGT#ACTGTCCTGCTGTGTCTGTA#...((((((((((.((((((((#)))).)))))))))))))).	1807	1827	0.84615	-28	0	0.0002148	0.5	2.7	3.13	-7	18	20	14	3UTR
mmu-miR-214-3p	NM_029172	Zc2hc1b	ACAGCAGGCACAGACAGGCAGT#CTGACCACTGCGCTTGCTC#..(((((((.(((...(((((.#))).)).))).))))))).	831	850	1	-20	1	0.1051651	0.5	1.9	1.34	-8	15	19	7	3UTR
mmu-miR-214-3p	NM_029216	Chd5	ACAGCAGGCACAGACAGGCAGT#GCTGCAACCATCCGTACCTCTTCCCTGTGGCTGCTGTG#(((((((.(((((....(((((#)))))...................))))).))))))).	3281	3319	0.84615	-25	1	0.0008816	0.4	2.6	3.72	-8	17	38	7	CDS
mmu-miR-214-3p	NM_029216	Chd5	ACAGCAGGCACAGACAGGCAGT#CTGCCAATGCTGTCCTGCA#...(((((.((((.(((((((.#)))))..))))))))))).	5738	5757	0.84615	-23	0	0.0009532	0.4	4.5	4.43	-8	16	19	11	CDS
mmu-miR-214-3p	NM_029332	Akap13	ACAGCAGGCACAGACAGGCAGT#ACTGCCTGTCCTAGATGCCTGTCTGC#.(((((((((((.((((((((((#))))))))))...).)))))).))).	9295	9321	0.86813	-31	1	0.0006084	0.5	-0	-0.1	-7	20	26	10	3UTR
mmu-miR-214-3p	NM_029332	Akap13	ACAGCAGGCACAGACAGGCAGT#GCTGCCTGCTGGCCAGGTGGAGAAATGCAAAGTATTCTAATGCTGC#.((((((((.(((.((((((((#))))))))))))))).........................))))).	11883	####	1	-27	1	0.002657	0.5	0.9	0.49	-6	19	46	14	3UTR

mmu-miR-214-3p	NM_029332	Akap13	ACAGCAGGCACAGACAGGCAGT#CTGCCTGTCTGAAAACAGAAACTAAAGACATCAAGGAAGTGGCCCCACAGGTCTCCCTGCTGA#.((((((((((((((((((((.#)))))))))).............................)))..............))))))).	3137	3200	0.84615	-27	1	4.73E-05	0.4	1.1	0.53	-4	20	63	10	CDS
mmu-miR-214-3p	NM_029362	Chmp4b	ACAGCAGGCACAGACAGGCAGT#GCTGTGTTTCTGTCTGTCACCTGTGTA#(((.((((.(((((((((((((#)))))....))))))))..))))))).	1308	1335	0.84615	-23	0	0.1172551	0.5	1.2	1.09	-8	20	27	8	3UTR
mmu-miR-214-3p	NM_029362	Chmp4b	ACAGCAGGCACAGACAGGCAGT#GCGGGGGGGAGGTGCCTGCTGTT#(((((((((((...........#.))))))))))).	1022	1045	1	-22	1	0.0003943	0.5	0.7	2.05	-10	11	13	11	3UTR
mmu-miR-214-3p	NM_029391	Rab4b	ACAGCAGGCACAGACAGGCAGT#GCCGTGGCCCCTCAGCCCTGTGGCTGCTGA#.((((((.(((((...(((...#.)))))))).)))))).	753	783	0.92308	-25	1	1.11E-05	0.4	3.8	3.6	-8	14	17	8	CDS
mmu-miR-214-3p	NM_029456	Ppp6r3	ACAGCAGGCACAGACAGGCAGT#ATTGCCCTGTTTGAAGCATGTTGTA#((((((.((.((((((((((((#)))))).))))))..)).)))))).	1967	1992	0.84615	-22	0	0.0026079	0.6	2.9	1.7	-7	20	25	6	CDS
mmu-miR-214-3p	NM_029512	Ttpal	ACAGCAGGCACAGACAGGCAGT#ACTGCCTCCTGGAACGCCGTGCTGC#.((((((((.(((..(((((((#))))))).)))....))).))))).	968	993	0.92308	-24	1	0.0062847	0.4	1.9	2.85	-7	18	25	7	CDS
mmu-miR-214-3p	NM_029512	Ttpal	ACAGCAGGCACAGACAGGCAGT#CTGCCAGCCCATGCCTGCCTGC#.(((((((((..(.(.(((((.#))))).).)..))))))).)).	1024	1046	1	-22	1	0.0101874	0.4	0.4	2.08	-11	16	22	7	CDS
mmu-miR-214-3p	NM_029512	Ttpal	ACAGCAGGCACAGACAGGCAGT#ACTGCAGTCTGTTCACTTTGTAAACCTGCTT#..((((((.((((((..(((((#))))).))))))...........)))	3960	3991	0.92308	-22	1	0.0108516	0.5	0	-0	-6	17	31	6	3UTR
mmu-miR-214-3p	NM_029626	Glt8d1	ACAGCAGGCACAGACAGGCAGT#GCGCCTGCTGCGCCTGGCTT#..(((((((.(((.(((((.((#)))))))))).))))).)).	226	246	0.92308	-24	1	3.11E-06	0.3	-0	-0.1	-7	17	20	10	5UTR
mmu-miR-214-3p	NM_029665	Ipo11	ACAGCAGGCACAGACAGGCAGT#GCCTGCCATCCATTTATGAGCCTGCTGA#.((((((((.((..(((((...#)))))...........)).)))))))).	3184	3212	0.84615	-24	1	0.0078296	0.6	3.8	3.92	-7	15	28	8	3UTR
mmu-miR-214-3p	NM_029665	Ipo11	ACAGCAGGCACAGACAGGCAGT#ACTGTGACAGTCGTGCTTGGCTGTG#(((((((((((.(((..(((((#)))))....)))))))))).)))).	3892	3917	0.90385	-23	1	3.36E-06	0.5	4.2	3.54	-8	19	25	10	3UTR
mmu-miR-214-3p	NM_029669	Dnajc18	ACAGCAGGCACAGACAGGCAGT#CTGCCTGCCTCTGCTTCCTGA#.(((.(((((.(((.((((((.#))))))).)).))))).))).	3340	3361	1	-24	0	0.0002392	0.5	0.1	0.29	-12	17	21	7	3UTR

mmu-miR-214-3p	NM_029669	Dnajc18	ACAGCAGGCACAGACAGGCAGT#ACTGTTGCACCTATTGTCTGTTCTTACAGCCTGGTGG#.((.((((((((((((((((((#)))))).......)))))))).......))))).)).	4101	4138	1	-23	0	4.47E-05	0.6	3.1	3.66	-7	20	37	8	3UTR
mmu-miR-214-3p	NM_029709	Clasp1	ACAGCAGGCACAGACAGGCAGT#CCTGTCTGGCCCAGGTGCTGC#.((((((((.((((((((....#)))))))))))....))))).	767	788	1	-26	1	3.77E-06	0.4	2.6	2.95	-5	16	21	11	CDS
mmu-miR-214-3p	NM_029709	Clasp1	ACAGCAGGCACAGACAGGCAGT#TTGCACGTGTCTGCACCACCTGCTGA#.(((((((..((((((((((((.#)))).).)))))).....))))))).	5327	5353	0.84615	-23	1	0.0005851	0.5	0.6	1.25	-6	18	26	7	3UTR
mmu-miR-214-3p	NM_029749	Usp42	ACAGCAGGCACAGACAGGCAGT#CTGCCAGCTGGGGCGCTGTGTCTTCAATAAGTGATGTCTCAAGTCACACACTTCCATTAGGGCCAGTGCCTGGTGC#.((.((((((((((((((((((.#))))))...........................))))).........................)))))).)).	227	303	0.81538	-23	0	2.83E-07	0.5	3	2.6	-7	19	76	7	CDS
mmu-miR-214-3p	NM_029793	Golga1	ACAGCAGGCACAGACAGGCAGT#TTGCCTTCTGTGGTACCTGCTC#..((((((((((((.((((((.#))))))))))))...)))))).	3428	3450	1	-25	1	0.0034663	0.6	1.1	0.76	-6	18	22	12	3UTR
mmu-miR-214-3p	NM_029793	Golga1	ACAGCAGGCACAGACAGGCAGT#CTGCCTCTGCCTCTGCCTCCAAGTGCTGA#.(((((((((((((..(((((.#))))))))))....)))))......)))).	4710	4739	1	-21	1	0.0002933	0.5	0.4	0.7	-5	18	29	9	3UTR
mmu-miR-214-3p	NM_029839	Trub1	ACAGCAGGCACAGACAGGCAGT#AGTGCCTTCTCTGCCTCTGCTGTC#((((((((((.(((.(((((.(#).)))))))).)))))..))))).	3102	3126	1	-24	1	0.001759	0.5	0.1	0.11	-7	19	24	8	3UTR
mmu-miR-214-3p	NM_029846	Atg16l1	ACAGCAGGCACAGACAGGCAGT#GCTCCCTGTCTGGCAGTAATGCTGG#.(((((.(((.((((((((.(((#))).))))))))))).....))))).	1267	1292	0.92308	-24	0	2.71E-06	0.6	1.9	3.62	-7	18	25	10	CDS
mmu-miR-214-3p	NM_029846	Atg16l1	ACAGCAGGCACAGACAGGCAGT#ACTGCCTGGACCTGCGCACCAAACTGCA#...((((((.(((.((((((((#))))))))...))).))......)))).	619	647	1	-23	0	0.0002909	0.4	2.2	2.86	-9	17	28	8	CDS
mmu-miR-214-3p	NM_029862	Cacfd1	ACAGCAGGCACAGACAGGCAGT#GCCCAGACTAGGCCTCATTCTTGTCTGTGTCTGGGTGTC#(((.((((((((((((((.(((#.))))))))))))))))..))).	1529	1568	0.88462	-24	0	2.34E-06	0.4	-1	-0.6	-7	20	24	17	3UTR

mmu-miR-214-3p	NM_029896	Wdr82	ACAGCAGGCACAGACAGGCAGT#CTGCACCTGTGTATGTGTGACAGTCACTTTTGCATGGCTTCTGTGCCTGGCTTGTG#((((((((((((((.((.((((#.)).)).))))))))))))).)).)).	2605	2661	0.92308	-24	1	2.86E-07	0.5	0.8	1.05	-5	20	26	12	3UTR
mmu-miR-214-3p	NM_029896	Wdr82	ACAGCAGGCACAGACAGGCAGT#TGCTCTGTCTGCACCTGTGTA#(((.((((..(((((((((..#))).)))))))..))))))).	2597	2618	1	-24	0	0.0033111	0.6	1.1	1.24	-9	17	21	7	3UTR
mmu-miR-214-3p	NM_029896	Wdr82	ACAGCAGGCACAGACAGGCAGT#ACTGTCACCTGCTGTGTCTACACTGTT#((((.((((((((.((((((((#))))...)))))))))))))...)))).	2729	2756	0.92308	-23	0	0.0141299	0.5	0.1	0.47	-9	20	27	12	3UTR
mmu-miR-214-3p	NM_029920	Mtus2	ACAGCAGGCACAGACAGGCAGT#CTGCCTGTCTTTGAGCATGCC#...(((.((..((((((((((.#))))))))))....)).))).	6494	6515	0.92308	-23	0	2.54E-05	0.5	0.1	0.19	-12	15	21	10	3UTR
mmu-miR-214-3p	NM_029920	Mtus2	ACAGCAGGCACAGACAGGCAGT#CCTGCTGGCCTGCA#...((((((.(((.((((....#))))))))))))).	7337	7351	0.92308	-23	0	9.73E-07	0.4	0.3	-0.3	-9	13	14	13	3UTR
mmu-miR-214-3p	NM_029936	Ddx10	ACAGCAGGCACAGACAGGCAGT#ACCTATTACTGAATATGCACTGTCTCTTGGTCTTGCTGTG#((((((((.((((((((((((..#.))).)))))))....)))))))))).	1590	1630	1	-24	1	0.0010584	0.6	1	0.74	-5	19	26	10	CDS
mmu-miR-214-3p	NM_029936	Ddx10	ACAGCAGGCACAGACAGGCAGT#TCTGTGCCTTCCTGTA#((((.(((((((((........#))))))))).)))).	2743	2759	1	-19	0	0.0125096	0.6	4.8	3.9	-9	13	16	9	3UTR
mmu-miR-214-3p	NM_029972	Ermn	ACAGCAGGCACAGACAGGCAGT#CTGATACCTGTTGGAGATTATGTGACTGCTGG#.((((((.((((((((((((((.#)))...))))))........)))	3272	3304	0.92308	-22	1	0.0130107	0.6	0.1	0.83	-6	19	32	6	3UTR
mmu-miR-214-3p	NM_029972	Ermn	ACAGCAGGCACAGACAGGCAGT#ACAACCTGACCATGTAAAAGATCTGTTACCCCTAGAGTGTTGAGCCTGCTGA#.((((((((...(((((((((.#.)))))..........))))..)))))))).	2486	2538	0.92308	-21	1	0.0227681	0.6	0.9	0.49	-11	17	31	8	3UTR
mmu-miR-214-3p	NM_029992	Tchp	ACAGCAGGCACAGACAGGCAGT#GCCTGTTTCATTTCCTGCTGA#.((((((((...(((((((((...#))))))))).....))))))).	2740	2761	0.92308	-25	1	0.0001669	0.4	0.5	0.05	-9	15	21	8	3UTR
mmu-miR-214-3p	NM_030100	Pym1	ACAGCAGGCACAGACAGGCAGT#GCTGCTGATCTGGGTGTCTTGTTGA#.((((((((((((((..(((((((#)))))))..)))..)))))).)))).	940	965	0.92308	-23	1	0.003142	0.4	1.5	1	-5	19	25	6	3UTR

mmu-miR-214-3p	NM_030108	Tmem33	ACAGCAGGCACAGACAGGCAGT#GCTGCCACTACCTGCTGTA#((((((((...((...((((((#)))))).)).)))))))).	541	560	1	-23	1	0.018122	0.5	3.4	4.02	-11	16	19	8	CDS
mmu-miR-214-3p	NM_030108	Tmem33	ACAGCAGGCACAGACAGGCAGT#CTCCTGTCTCTGCCTC#.....(((((.(((((((.((.#)))))))))).))))).	4443	4459	1	-21	0	0.0614719	0.5	0.2	-0.1	-16	14	16	9	3UTR
mmu-miR-214-3p	NM_030108	Tmem33	ACAGCAGGCACAGACAGGCAGT#TGTCGTGTGTGCGCTGTG#(((((..(((((.((.((((..#)))))).)))))))))).	2048	2066	1	-21	0	0.0007998	0.6	-0	0.2	-9	16	18	10	3UTR
mmu-miR-214-3p	NM_030112	Rtf1	ACAGCAGGCACAGACAGGCAGT#CTGCCTGTCTGCTGATTCCAGTCAACATTCAGCACCTACCTGCA#...((((((((((((((((.#)))))))))).)).......................))))).	4256	4300	0.92308	-28	0	0.0013529	0.4	0.1	0.48	-7	18	44	11	3UTR
mmu-miR-214-3p	NM_030112	Rtf1	ACAGCAGGCACAGACAGGCAGT#TGCCTATGTGTGTCTCTGTG#((((.(((((((.(.(((((..#))))).).))))))))))).	3286	3306	1	-24	0	0.0893184	0.6	-0	0.98	-10	17	20	11	3UTR
mmu-miR-214-3p	NM_030112	Rtf1	ACAGCAGGCACAGACAGGCAGT#GCTGTCTGTAGATCTGTTGTCTGGTTTCTGA#.((((((((((((((((((#))))))))....))))).)))))...	4531	4562	0.92308	-23	1	0.0011646	0.6	0.7	0.63	-4	21	31	8	3UTR
mmu-miR-214-3p	NM_030185	Zcchc4	ACAGCAGGCACAGACAGGCAGT#CTGCCCCGCTGTGCCCGCA#...((.(((((((...(((((.#)))))...))))))).)).	139	158	0.84615	-26	0	2.59E-05	0.4	1.2	1.64	-10	14	19	7	CDS
mmu-miR-214-3p	NM_030185	Zcchc4	ACAGCAGGCACAGACAGGCAGT#TTGCCTGTACCTATCATGTATGTGATCATGTCCCTGCCTGATGTCTGCA#...(((((((((((((((((((.#)))))))))...........................))).))))))	3747	3796	0.92308	-23	0	0.0003469	0.5	0.1	0.18	-7	18	49	8	3UTR
mmu-miR-214-3p	NM_030197	Cacul1	ACAGCAGGCACAGACAGGCAGT#GCTCCCTCTCTGGTCCCCTGCTGC#.(((((((..((((.(((.(((#))).))).))))....))))))).	5064	5088	1	-23	1	0.0092343	0.4	3.5	3.18	-7	17	24	7	3UTR
mmu-miR-214-3p	NM_030197	Cacul1	ACAGCAGGCACAGACAGGCAGT#ACTGACCTGCTGTCATAACTGCA#...((((..((((.((((((((#)))).)))))))).....)))).	3904	3927	0.84615	-20	0	0.0022807	0.6	1.3	1.23	-11	16	23	8	3UTR
mmu-miR-214-3p	NM_030228	Gas2l1	ACAGCAGGCACAGACAGGCAGT#GCTGCCTGTGCCTAGGCCCGGCTC#..((((.(((..(((((((((((#))))))))))..))..)))..))).	1529	1553	0.84615	-24	0	3.11E-05	0.4	-1	-0.2	-11	17	24	9	3UTR

mmu-miR-214-3p	NM_030235	Avl9	ACAGCAGGCACAGACAGGCAGT#GCTGCTTGGTTTTCTGCGGTGTAATGCTCCCTCCCCGTCTGCTGC#.((((((((.((((((((((((#))))))))....)))).................)))))))).	2186	2231	0.84615	-23	1	0.0183932	0.5	0.2	0.53	-5	20	45	8	3UTR
mmu-miR-214-3p	NM_030257	Lysmd3	ACAGCAGGCACAGACAGGCAGT#GCAAGACATTGTGCCAGCTGA#.((((.(((((((.(..((...#))..)...))))))).)))).	560	581	0.92308	-19	0	0.0109676	0.5	0.6	1.6	-7	14	21	7	CDS
mmu-miR-214-3p	NM_030260	Zxdc	ACAGCAGGCACAGACAGGCAGT#GCTGCACAGTCTGTCTGTCCATTGCTGG#.(((((((.(((((((((((((#)))))).....))))))))))..))))).	3096	3124	0.84615	-27	1	0.0013727	0.4	0	-0.3	-5	20	28	10	3UTR
mmu-miR-214-3p	NM_030260	Zxdc	ACAGCAGGCACAGACAGGCAGT#GCTGCCTGGTGGCTCTGCCTGTG#(((((((((.((..((((((((#)))))))).)))).)))).))).	1996	2019	0.84615	-26	1	5.18E-06	0.5	1.2	1.27	-7	19	23	8	CDS
mmu-miR-214-3p	NM_030260	Zxdc	ACAGCAGGCACAGACAGGCAGT#GCGCCGTCGCCTGCTGC#.((((((((...(((.(((.((#)))))))))))))))).	275	292	1	-23	1	3.09E-05	0.2	0.5	0.02	-11	16	17	16	CDS
mmu-miR-214-3p	NM_030689	Nptxr	ACAGCAGGCACAGACAGGCAGT#ACTGCCTGTCCTTGAGCCCTGCTT#..((((((.((((((((((((#))))))))))..)).))).)))).	2803	2827	0.84615	-28	1	0.0013231	0.4	0.7	0.01	-6	19	24	10	3UTR
mmu-miR-214-3p	NM_030719	Castor2	ACAGCAGGCACAGACAGGCAGT#GCTGCGCCGGGCGCCGCGCTCAGCCGGGCGCTGTTTGCTTGTCCACCCCGTAGCTTGCTGC#.((((((((((.(((((((((.#.)))))))))).....)).)))))))).	4	65	0.84615	-24	1	0.0015573	0.3	0.8	0.98	-7	19	28	9	5UTR
mmu-miR-214-3p	NM_030728	Cemip	ACAGCAGGCACAGACAGGCAGT#GCTGCTGGGATGTGTCTGTGGG#.(.(((((((((..(.((((((#)))))).)..))))))))).).	6359	6381	0.92308	-25	0	4.63E-06	0.5	3.7	2.5	-8	17	22	9	3UTR
mmu-miR-214-3p	NM_030728	Cemip	ACAGCAGGCACAGACAGGCAGT#ACTGTGCTTTGCTCTGTGCCTGGAGC#.(..(((((((((((((((((#)))))...))).))))))))))..).	4933	4959	0.84615	-24	0	2.11E-05	0.6	2.6	2.36	-8	19	26	10	3UTR
mmu-miR-214-3p	NM_030728	Cemip	ACAGCAGGCACAGACAGGCAGT#TGCCAATGTGCTTCTGCTGG#.(((((((((((....((((..#))))..)))))..)))))).	6019	6039	0.92308	-23	1	0.0006157	0.5	2.6	2.44	-7	15	20	6	3UTR
mmu-miR-214-3p	NM_030729	Nckipsd	ACAGCAGGCACAGACAGGCAGT#CTGCTGTCTGGTCTGA#....(((((.((((((.((((.#)))))))))))))))).	2584	2600	0.92308	-22	0	2.09E-05	0.4	0	0.68	-11	15	16	15	3UTR

mmu-miR-214-3p	NM_030732	Tbl1xr1	ACAGCAGGCACAGACAGGCAGT#ACTGTGTGCTTTGCTGCTGCTCTCACTGTCCCTGTAGCCTGCTGA#.((((((((((((((((((((#.))))).....)))))..))).)))))))).	4789	4834	1	-29	1	0.0037274	0.4	1.5	1.24	-4	21	31	8	3UTR
mmu-miR-214-3p	NM_030732	Tbl1xr1	ACAGCAGGCACAGACAGGCAGT#GCTCATTTGTGCCTGGCTGG#.((((((((((((..(((...#)))..))))))))))).))).	53	73	0.84615	-25	1	0.0002479	0.2	0.3	0.49	-5	16	20	10	5UTR
mmu-miR-214-3p	NM_030733	Gpr63	ACAGCAGGCACAGACAGGCAGT#CTGCCGGTTGTCGGCTATGTTTTTCTGGTTGTTTGTGATAGAGGGAGTAGCCATCCTGCTC#..((((((((((((((.(((.#.))).)))))))))).................)))))).	890	951	1	-23	1	0.0001136	0.6	0	0	-5	18	38	9	CDS
mmu-miR-214-3p	NM_030733	Gpr63	ACAGCAGGCACAGACAGGCAGT#TTGCCGTTTTCATTGTCTGCTGG#.(((((((((..((..(((((.#)))))....))..))))))))).	1390	1413	0.92308	-23	1	0.0051212	0.5	0	0	-11	16	23	9	CDS
mmu-miR-214-3p	NM_030733	Gpr63	ACAGCAGGCACAGACAGGCAGT#CTGGCTACTCTGGCTCTGCTA#..(((((((.((((.((.(((.#))).))..)))))).))))).	1493	1514	0.92308	-20	1	0.0018619	0.5	0	0	-7	16	21	6	CDS
mmu-miR-214-3p	NM_031162	Cd247	ACAGCAGGCACAGACAGGCAGT#GCTGCCTCTGCCTCTGCCTCTGG#.(((.((((((((..((((((#))))))))))....)))))))).	102	125	0.84615	-23	0	1.38E-05	0.4	0.2	0.12	-9	18	23	10	5UTR
mmu-miR-214-3p	NM_031162	Cd247	ACAGCAGGCACAGACAGGCAGT#AGTGGAAAGTGTCTGTTCTCGCCTGCA#...((((((...((((((((.(#.).))))))))...)))))).	152	179	0.84615	-23	0	0.0001108	0.4	0.2	0.36	-14	15	21	8	CDS
mmu-miR-214-3p	NM_031184	Glis2	ACAGCAGGCACAGACAGGCAGT#CTGCCTGCTGCTATGACAAGGTGCTTCTCTGCTTGCTT#..(((((((((((.((((((((.#))))))))))))..................)))))))).	2354	2392	0.84615	-25	1	0.0026073	0.4	0.5	0.38	-6	18	38	10	3UTR
mmu-miR-214-3p	NM_031408	Gigyf1	ACAGCAGGCACAGACAGGCAGT#GCTGCCTTGCCTGTTGC#.(((((((((.....(((((((#)))))))))))))))).	4462	4479	0.92308	-25	1	0.0014868	0.3	-0	0.32	-13	16	17	16	3UTR
mmu-miR-214-3p	NM_031842	Smarcd1	ACAGCAGGCACAGACAGGCAGT#GCTATCCCTGTCTGAGCCTCAGGCCTAGGGCAGTCTGTAAGCTAGCTGA#.(((((((.((((((((.(((#)))...)))))))).))))........................)	1972	2021	0.92308	-24	1	0.0002455	0.4	0.2	0.8	-5	19	49	8	3UTR
mmu-miR-214-3p	NM_031842	Smarcd1	ACAGCAGGCACAGACAGGCAGT#GCTGCCTGCCTCAGTGTACCTGTC#...(((((((((((.((((((((#)))))))).))..)))..))))).	1629	1653	0.92308	-23	0	0.0024661	0.4	0.8	0.72	-7	18	24	8	3UTR

mmu-miR-214-3p	NM_032000	Trps1	ACAGCAGGCACAGACAGGCAGT#TTGCTTGTAGAAAAAGTAATTGTTCCCACTGTGCACTGTTGC#.((((((((((((((((((((.#))))))))...................)))))).))))))).	2193	2235	1	-25	1	0.0003906	0.5	1.8	1.95	-4	20	42	8	CDS
mmu-miR-214-3p	NM_032000	Trps1	ACAGCAGGCACAGACAGGCAGT#CTGTTTTAATCTCACCTGTCTGTGGCATAGCTC#..(((.(.((((((((((....#.)))))))))).)...))).	6615	6648	0.92308	-23	0	0.0005859	0.6	3.5	3.94	-9	14	20	10	3UTR
mmu-miR-214-3p	NM_032000	Trps1	ACAGCAGGCACAGACAGGCAGT#TGCTTTCTGGCGTGCTGC#.(((((.((.((((.(((((..#))))))))))).))))).	1296	1314	1	-23	0	1.08E-05	0.5	0.7	0.33	-7	16	18	11	CDS
mmu-miR-214-3p	NM_033354	Sec16b	ACAGCAGGCACAGACAGGCAGT#ACTAGGGGCCTGGTGCCTGCC#...((((((((...(((((...#.))))))))))))).	76	97	0.92308	-25	0	1.29E-05	0.5	0.1	0.6	-12	13	15	13	5UTR
mmu-miR-214-3p	NM_033354	Sec16b	ACAGCAGGCACAGACAGGCAGT#CTGCTTCTGTGTCTGG#....((((((((((..(((((.#)))))))))))))))).	909	925	0.92308	-23	0	3.35E-07	0.5	0.4	0.46	-11	15	16	15	CDS
mmu-miR-214-3p	NM_033354	Sec16b	ACAGCAGGCACAGACAGGCAGT#CTTGTTGTGAGCCTGCTGC#.((((((((.((((((((....#)))))).)).)))))))).	3661	3680	1	-22	1	0.0003458	0.4	-0	-0.1	-5	16	19	8	3UTR
mmu-miR-214-3p	NM_033474	Arvcf	ACAGCAGGCACAGACAGGCAGT#ACTGCCGGCAGCAGTGGGGGGAACTGCTGTTCAGTGCCTGCTGTT#(((((((((((.((..((((((#.))))))..)).))))))))))).	3842	3887	0.92308	-32	1	4.43E-05	0.5	-0	0.57	-8	19	24	11	3UTR
mmu-miR-214-3p	NM_033474	Arvcf	ACAGCAGGCACAGACAGGCAGT#ACTGCCTGTCTTCTGCCTTGGCTC#..((((((((.((((((((((((#))))))))))))..)))))..))).	4004	4028	1	-29	1	0.0003356	0.5	-0	0.53	-8	19	24	11	3UTR
mmu-miR-214-3p	NM_033623	Dcun1d1	ACAGCAGGCACAGACAGGCAGT#CTGCTTGTGTGTTTACGTGTGTTGC#.((((((.(((((.((((((((.#)))))))).)))....)).))))).	3944	3969	1	-24	0	0.0421748	0.5	3.8	3.97	-7	18	25	8	3UTR
mmu-miR-214-3p	NM_033623	Dcun1d1	ACAGCAGGCACAGACAGGCAGT#TGCCTTGCTCTCCTGCTGA#.(((((((...((((((((..#))))).).)))))))))).	2223	2242	1	-21	1	0.0100913	0.6	0	0.95	-9	16	19	10	3UTR
mmu-miR-214-3p	NM_033623	Dcun1d1	ACAGCAGGCACAGACAGGCAGT#GGTTGTGCTTTGCCTGGTGA#.((.((((((.((((((.(...#).)))).)).)))))).)).	48	68	0.92308	-20	0	6.44E-05	0.6	0.8	1.01	-10	15	20	6	5UTR
mmu-miR-214-3p	NM_033623	Dcun1d1	ACAGCAGGCACAGACAGGCAGT#GCCATGTTGGCTGCTGA#.((((((.((...((((((...#))).))))).)))))).	1986	2003	0.92308	-20	1	7.09E-05	0.5	0.6	0.63	-14	14	17	6	3UTR

mmu-miR-214-3p	NM_053089	Naa15	ACAGCAGGCACAGACAGGCAGT#GGTGTGTGTGTGTGTGTGTGTGTG#((((((.(((((.(((.(((.(#).))).))).))))).))).))).	4848	4872	0.84615	-22	0	0.0001257	0.4	-0	1.3	-7	18	24	5	3UTR
mmu-miR-214-3p	NM_053089	Naa15	ACAGCAGGCACAGACAGGCAGT#CTGTCTGTTTGTCTTGA#....((((.(((((((((((((.#))))))))))))))))).	4930	4947	1	-22	0	0.0065954	0.7	1.7	1.5	-11	16	17	16	3UTR
mmu-miR-214-3p	NM_053089	Naa15	ACAGCAGGCACAGACAGGCAGT#GCTGTTGTCTAAGCTTTGCTGA#.((((((((..((((((.(((((#))))))))))..)))).)))).	5937	5959	0.84615	-21	1	0.0001989	0.6	0.2	0.04	-8	18	22	10	3UTR
mmu-miR-214-3p	NM_053169	Trim16	ACAGCAGGCACAGACAGGCAGT#CTGTTTGTTTGTGCTAAGTTGA#.((((.((((((((((((((((.#))))))))))))))))..)))).	2838	2860	0.84615	-26	0	0.0041643	0.5	-0	-0.3	-6	19	22	15	3UTR
mmu-miR-214-3p	NM_053169	Trim16	ACAGCAGGCACAGACAGGCAGT#AGTGAAATCCTGTCTGACCTGCATGG#.(((((((..((((((((....#.)))))))).))))).)).	491	517	0.84615	-23	1	1.01E-05	0.4	1.7	2.2	-6	15	19	8	CDS
mmu-miR-214-3p	NM_053169	Trim16	ACAGCAGGCACAGACAGGCAGT#GCGTGTTTCCCTGGCCTGCC#...((((((.((((((.((...#)).)))....))))))))).	3659	3679	1	-22	0	0.0226532	0.6	-0	0.02	-8	14	20	9	3UTR
mmu-miR-214-3p	NM_053170	Trim33	ACAGCAGGCACAGACAGGCAGT#ACTGCTTCGAGTCTGTTGCCTAGTA#...((((((((((((((((((#)))))))...)))))).)))).)).	7720	7745	0.92308	-24	0	0.0060507	0.6	2.2	1.36	-7	19	25	7	3UTR
mmu-miR-214-3p	NM_053170	Trim33	ACAGCAGGCACAGACAGGCAGT#CTGCTTGACTGAGTGAACAAACTGCTGTA#(((((((((.(((.(((((((.#))))))).))).)).......))))))).	6678	6707	1	-23	1	0.0005271	0.5	0.5	0.23	-6	19	29	7	3UTR
mmu-miR-214-3p	NM_053196	Sfxn2	ACAGCAGGCACAGACAGGCAGT#CTGCCTGTTAGGTCACATACCAAAGATTGCTGAGTGCTGG#.(((((..(((((((((((((.#))))))))).................)).))..))))).	2968	3008	0.84615	-22	0	0.0021637	0.5	0.7	0.4	-8	18	40	9	3UTR
mmu-miR-214-3p	NM_053198	Sfxn4	ACAGCAGGCACAGACAGGCAGT#CTGCCTGTGACAGCACCCATGGTATTTCTGTTGA#.((((((((....((((((((.#))))))))....)).............)))))).	358	392	0.84615	-21	1	0.0008143	0.5	0.3	0.06	-12	16	34	8	CDS
mmu-miR-214-3p	NM_053246	Dok4	ACAGCAGGCACAGACAGGCAGT#AGTGCCTGTCCTCAGTGATACAACTCTCCTGCTGC#.((((((((((.((((((((.(#).))))))))....)))..........))))))).	1653	1688	1	-26	1	0.0071879	0.4	3.7	4.06	-7	19	35	8	3UTR

mmu-miR-214-3p	NM_054043	Msi2	ACAGCAGGCACAGACAGGCAGT#GCCTGGATGTGGGGCTGG#.((((...((((..(((((...#)))))..))))..)))).	4653	4671	0.84615	-24	0	2.07E-07	0.4	1.3	1.64	-12	13	18	5	3UTR
mmu-miR-214-3p	NM_054043	Msi2	ACAGCAGGCACAGACAGGCAGT#GCCTCTCTGTGCACAGCTT#..(((.((((((((.((((...#)))).))))))).).))).	2280	2299	0.84615	-23	0	0.0060659	0.5	4.9	3.46	-8	15	19	7	3UTR
mmu-miR-214-3p	NM_054046	Def8	ACAGCAGGCACAGACAGGCAGT#CTGCCCCTTTGCTCCTTGTCTGTGAATTGCTGTT#(((((((.((((((((((((.#)))))..........))))))))..))))))).	2638	2672	0.84615	-27	1	0.0068241	0.5	-0	-0.4	-6	20	34	8	3UTR
mmu-miR-214-3p	NM_054046	Def8	ACAGCAGGCACAGACAGGCAGT#GGTGCCTGCTGGCCTTGCC#...((((((.((((.((((((.(#).)))))))))))).)).	1494	1513	0.84615	-24	0	4.00E-06	0.4	-0	0.04	-9	16	19	13	3UTR
mmu-miR-214-3p	NM_054054	Brdt	ACAGCAGGCACAGACAGGCAGT#ACTCCAGAAGTGTGTCTTCATGCCTGGCTGAGTTGTTAACTTTTCAAAGAGATATGTGTCTGCTGTC#((((((((((((..((((((..#.))))))..........................)))))))))))).	3680	3747	0.92308	-28	1	0.0024337	0.7	0.6	0.2	-6	18	48	12	3UTR
mmu-miR-214-3p	NM_054054	Brdt	ACAGCAGGCACAGACAGGCAGT#CTGGCCTGTCTGAGGAAGAGGTCGCTGA#.((((.(((.(((((((((((.#))).)))))))))........))))))).	2008	2036	0.92308	-23	0	0.0005413	0.5	1.2	2.24	-7	18	28	8	CDS
mmu-miR-214-3p	NM_054054	Brdt	ACAGCAGGCACAGACAGGCAGT#CTGCTGTCCAGACAGGGCAGTGCACACCTGCTGTA#((((((((((.((((.((((.#))))))))))...........))))....))))))).	4418	4453	0.84615	-22	1	0.0001415	0.4	0.3	0.04	-7	19	35	8	3UTR
mmu-miR-214-3p	NM_054055	Slc13a3	ACAGCAGGCACAGACAGGCAGT#ACTAGCTGTCTGTGGCCTGTA#...((((((((((((((..(((#)))..)))))))))).))))).	2061	2082	0.92949	-27	0	0.0001314	0.5	2.9	3.77	-7	17	21	9	3UTR
mmu-miR-214-3p	NM_054078	Baz2a	ACAGCAGGCACAGACAGGCAGT#CTGCCTGGTCTGCCGGAAGGGCGATAATGATGAGTTTCTCCTGCTGTG#((((((((..((((((((((((.#))))))).)))).........................))))))))	5140	5188	0.92308	-27	1	0.0088492	0.5	2.6	3.83	-6	19	48	8	CDS
mmu-miR-214-3p	NM_054078	Baz2a	ACAGCAGGCACAGACAGGCAGT#GCTGTCTGTCTGTCCCAGCA#...((.((.((((((((((((((#))))))))))))).)).)).	5267	5287	0.92308	-27	0	6.60E-05	0.5	2	2.48	-9	17	20	13	CDS
mmu-miR-214-3p	NM_054078	Baz2a	ACAGCAGGCACAGACAGGCAGT#TGTCTGTCTGACCACCAGCCCCTGCCTGACTT#..((((((((((((((((((..#))))))))))..........))	2603	2635	1	-25	1	0.0013977	0.4	3.3	2.85	-5	18	32	10	CDS

mmu-miR-214-3p	NM_054078	Baz2a	ACAGCAGGCACAGACAGGCAGT#ACTGCCCTGTGTGTGACTGA#.(((((.((((((...((((((#)))))))))))).)).))).	7220	7240	0.92308	-24	0	3.71E-05	0.4	0.9	0.89	-7	17	20	12	3UTR
mmu-miR-214-3p	NM_054087	Slc19a2	ACAGCAGGCACAGACAGGCAGT#GCATGTGTGTGTGTGTCTGCA#...(((((((((.(((.(((((#)).))).))).))))))))).	2154	2175	1	-26	0	0.0009372	0.5	0.6	0.35	-8	17	21	9	3UTR
mmu-miR-214-3p	NM_054087	Slc19a2	ACAGCAGGCACAGACAGGCAGT#ACTACCTCCGGTACAAGCCTGTCATCCTGCTGC#.(((((((....(((((((...#.)))))))..))))))).	484	517	0.92308	-26	1	0.0010439	0.4	3.8	3.22	-12	14	18	7	CDS
mmu-miR-214-3p	NM_054087	Slc19a2	ACAGCAGGCACAGACAGGCAGT#CTGTGTTTCTGTGGCTTTTGCTGTG#((((((.((((((.(.((((.#)))).).)))))).))...))))).	794	819	0.84615	-24	1	0.0002105	0.5	2.6	2.57	-7	18	25	6	CDS
mmu-miR-214-3p	NM_080555	Plpp3	ACAGCAGGCACAGACAGGCAGT#TGTCCTGGCAGGATTTGCCCAAGGAGCTCTGGTGGCCTGCTGC#.((((((((.((..(((((...#.)).))).)))))))))).	1245	1288	0.92308	-24	1	0.0006967	0.4	3	3.54	-7	15	19	10	CDS
