# Supplementary material for: Bioinformatic prediction, qCLASH, and AlphaFold3 reveal non-overlapping layers of the miR-214-3p–mRNA interactome
Source: Front Genet. 2026 Sep 30;17:1924314. doi: 10.3389/fgene.2026.1924314 (PMC13661022; doi:10.3389/fgene.2026.1924314)
Supplement: Supplementary file 6 [file DataSheet4.pdf]

Note: This sheet contains the 95 genes identified as the common intersection of TargetScan, miRDB, and miRWalk after Ensembl release 116 Gene ID harmonization. The ‘gene_symbol’ column reflects the current Ensembl official symbol; the input columns show each tool’s original gene symbol. Five cross-method symbol pairs were resolved

gene_id	gene_symbol	TargetScan_input_gene_symbol	miRDB_input_gene_symbol	miRWalk_input_gene_symbol
ENSMUSG0000000108	Luzp1	Luzp1	Luzp1	Luzp1
ENSMUSG0000000248	Tchp	Tchp	Tchp	Tchp
ENSMUSG0000000489	Hdgf	Hdgf	Hdgf	Hdgf
ENSMUSG0000000576	Cd247	Cd247	Cd247	Cd247
ENSMUSG0000000615	Eps8l1	Eps8l1	Eps8l1	Eps8l1
ENSMUSG0000000692	Ezh1	Ezh1	Ezh1	Ezh1
ENSMUSG0000001079	Wnt2	Wnt2	Wnt2	Wnt2
ENSMUSG0000001100	Mcoln2	Mcoln2	Mcoln2	Mcoln2
ENSMUSG0000001771	Afmid	Afmid	Afmid	Afmid
ENSMUSG0000002004	Abtb3	Btbd11	Btbd11	Btbd11
ENSMUSG0000002025	Glyctk	Glyctk	Glyctk	Glyctk
ENSMUSG0000002037	Rasgef1c	Rasgef1c	Rasgef1c	Rasgef1c
ENSMUSG0000002092	Lsm12	Lsm12	Lsm12	Lsm12
ENSMUSG0000002118	Fbln5	Fbln5	Fbln5	Fbln5
ENSMUSG0000002145	Sema4d	Sema4d	Sema4d	Sema4d
ENSMUSG0000002175	Plpp1	Ppap2a	Plpp1	Plpp1
ENSMUSG0000002225	Nadk2	Nadk2	Nadk2	Nadk2
ENSMUSG0000002250	Hapstr1	1810013L24Rik	1810013L24Rik	1810013L24Rik
ENSMUSG0000002282	Abcc5	Abcc5	Abcc5	Abcc5
ENSMUSG0000002290	Kpna1	Kpna1	Kpna1	Kpna1
ENSMUSG0000002301	Smarcd1	Smarcd1	Smarcd1	Smarcd1
ENSMUSG0000002349	Pcbp4	Pcbp4	Pcbp4	Pcbp4
ENSMUSG0000002392	Tbc1d5	Tbc1d5	Tbc1d5	Tbc1d5
ENSMUSG0000002401	Pim1	Pim1	Pim1	Pim1
ENSMUSG0000002453	Psmg2	Psmg2	Psmg2	Psmg2
ENSMUSG0000002490	Peli3	Peli3	Peli3	Peli3
ENSMUSG0000002494	Arl2	Arl2	Arl2	Arl2
ENSMUSG0000002557	Cbx2	Cbx2	Cbx2	Cbx2
ENSMUSG0000002572	Nmb	Nmb	Nmb	Nmb
ENSMUSG0000002595	Creb1	Creb1	Creb1	Creb1
ENSMUSG0000002673	Pter	Pter	Pter	Pter
ENSMUSG0000002685	Usp20	Usp20	Usp20	Usp20
ENSMUSG0000002685	Dolpp1	Dolpp1	Dolpp1	Dolpp1
ENSMUSG0000002687	Rab14	Rab14	Rab14	Rab14
ENSMUSG0000002708	Med19	Med19	Med19	Med19
ENSMUSG0000002738	Bcl2l11	Bcl2l11	Bcl2l11	Bcl2l11
ENSMUSG0000002747	Pdrg1	Pdrg1	Pdrg1	Pdrg1
ENSMUSG0000002754	Sall4	Sall4	Sall4	Sall4
ENSMUSG0000002765	Ralgapb	Ralgapb	Ralgapb	Ralgapb
ENSMUSG0000002770	Dcun1d1	Dcun1d1	Dcun1d1	Dcun1d1
ENSMUSG0000002830	C9orf72	3110043O21Rik	C9orf72	C9orf72
ENSMUSG0000002861	Ndc1	Ndc1	Ndc1	Ndc1
ENSMUSG0000002894	Espn	Espn	Espn	Espn
ENSMUSG0000002923	Pdcl2	Pdcl2	Pdcl2	Pdcl2
ENSMUSG0000002962	Cpsf4	Cpsf4	Cpsf4	Cpsf4
ENSMUSG0000002978	Tmem209	Tmem209	Tmem209	Tmem209
ENSMUSG0000003035	Tspan11	Tspan11	Tspan11	Tspan11
ENSMUSG0000003068	Cdipt	Cdipt	Cdipt	Cdipt
ENSMUSG0000003113	Vgll1	Vgll1	Vgll1	Vgll1
ENSMUSG0000003134	Cetn2	Cetn2	Cetn2	Cetn2
ENSMUSG0000003160	Galnt7	Galnt7	Galnt7	Galnt7

ENSMUSG0000003202	Rexo2	Rexo2	Rexo2	Rexo2
ENSMUSG0000003232	Hmg20a	Hmg20a	Hmg20a	Hmg20a
ENSMUSG0000003235	Lrrc1	Lrrc1	Lrrc1	Lrrc1
ENSMUSG0000003290	Slc16a1	Slc16a1	Slc16a1	Slc16a1
ENSMUSG0000003306	Gas7	Gas7	Gas7	Gas7
ENSMUSG0000003403	Fgd5	Fgd5	Fgd5	Fgd5
ENSMUSG0000003562	Rubcn	1700021K19Rik	Rubcn	Rubcn
ENSMUSG0000003576	Tmem161b	Tmem161b	Tmem161b	Tmem161b
ENSMUSG0000003587	Hykk	Hykk	Hykk	Hykk
ENSMUSG0000003607	Sigmar1	Sigmar1	Sigmar1	Sigmar1
ENSMUSG0000003628	Naa30	Naa30	Naa30	Naa30
ENSMUSG0000003698	Pml	Pml	Pml	Pml
ENSMUSG0000003762	Wdtc1	Wdtc1	Wdtc1	Wdtc1
ENSMUSG0000003802	Rapgefl1	Rapgefl1	Rapgefl1	Rapgefl1
ENSMUSG0000003936	Sec24c	Sec24c	Sec24c	Sec24c
ENSMUSG0000004179	Myrip	Myrip	Myrip	Myrip
ENSMUSG0000004191	Ammecr1l	Ammecr1l	Ammecr1l	Ammecr1l
ENSMUSG0000004240	Crtac1	Crtac1	Crtac1	Crtac1
ENSMUSG0000004259	Ipo11	Ipo11	Ipo11	Ipo11
ENSMUSG0000004338	Tmem130	Tmem130	Tmem130	Tmem130
ENSMUSG0000004346	Elfn2	Elfn2	Elfn2	Elfn2
ENSMUSG0000004447	Zfand3	Zfand3	Zfand3	Zfand3
ENSMUSG0000004453	Ackr2	Ackr2	Ackr2	Ackr2
ENSMUSG0000004461	Zbtb39	Zbtb39	Zbtb39	Zbtb39
ENSMUSG0000004523	Eola1	1110012L19Rik	1110012L19Rik	Eola1
ENSMUSG0000004591	Tmem268	6330416G13Rik	Tmem268	Tmem268
ENSMUSG0000004741	Rexo1	Rexo1	Rexo1	Rexo1
ENSMUSG0000004848	Zbtb8b	Zbtb8b	Zbtb8b	Zbtb8b
ENSMUSG0000004881	Lonrf2	Lonrf2	Lonrf2	Lonrf2
ENSMUSG0000004889	Zfp710	Zfp710	Zfp710	Zfp710
ENSMUSG0000004917	Myoz3	Myoz3	Myoz3	Myoz3
ENSMUSG0000005038	Kif7	Kif7	Kif7	Kif7
ENSMUSG0000005067	Ccdc96	Ccdc96	Ccdc96	Ccdc96
ENSMUSG0000005141	Plagl2	Plagl2	Plagl2	Plagl2
ENSMUSG0000005309	Tmem248	Tmem248	Tmem248	Tmem248
ENSMUSG0000005548	Mtcl2	Soga1	Soga1	Soga1
ENSMUSG0000005641	Adap1	Adap1	Adap1	Adap1
ENSMUSG0000005642	Tgoln1	Tgoln1	Tgoln1	Tgoln1
ENSMUSG0000005761	Ldoc1	Ldoc1	Ldoc1	Ldoc1
ENSMUSG0000006145	Nol10	Nol10	Nol10	Nol10
ENSMUSG0000006258	Cnr2	Cnr2	Cnr2	Cnr2
ENSMUSG0000006327	Naa15	Naa15	Naa15	Naa15
ENSMUSG0000007915	Nalf1	Fam155a	Fam155a	Fam155a
ENSMUSG0000007967	Vwde	Vwde	Vwde	Vwde
