# Supplementary material for: Bioinformatic prediction, qCLASH, and AlphaFold3 reveal non-overlapping layers of the miR-214-3p–mRNA interactome
Source: Front Genet. 2026 Sep 30;17:1924314. doi: 10.3389/fgene.2026.1924314 (PMC13661022; doi:10.3389/fgene.2026.1924314)
Supplement: Supplementary file 7 [file DataSheet5.pdf]

mirnaid	refseqid	genesymbol	duplex	binding_region_length	longest_consecutive_pairing	position
mmu-miR-214-3p	NM_001037726	Creb1	ACAGCAGGCACAGACAGGCAGT#TTGTCCTGCTGAGCTTGCTGG#.((((((((.((((.((((((.#))).))))))).)))))))).	21	8	3UTR
mmu-miR-214-3p	NM_001037726	Creb1	ACAGCAGGCACAGACAGGCAGT#GCTGCCTGTGGCCCGGGCGGCTGG#.((((.(.(....(((((((((#)))))))))......).).)))).	24	9	5UTR
mmu-miR-214-3p	NM_001037726	Creb1	ACAGCAGGCACAGACAGGCAGT#CTGTCTGTGCTAGCA#...((.(((((((((((.....#))))))))))).)).	15	11	3UTR
mmu-miR-214-3p	NM_001039586	Glyctk	ACAGCAGGCACAGACAGGCAGT#CTGATCATGGTTTCTCCTCTCCTGCTGTTGCCTGACTGG#.((((((((((((.((((.((.#.)))))))))).))))).))).	22	10	3UTR
mmu-miR-214-3p	NM_001039586	Glyctk	ACAGCAGGCACAGACAGGCAGT#ACTGTCACCATTGCCTGCTGA#.(((((((((......((((((#)))))).....))))))))).	21	9	3UTR
mmu-miR-214-3p	NM_001080925	Rapgefl1	ACAGCAGGCACAGACAGGCAGT#GCGTGTCTCGCCTGCTGC#.((((((((..(((((.((...#)).))))).)))))))).	18	8	CDS
mmu-miR-214-3p	NM_001081394	Tmem248	ACAGCAGGCACAGACAGGCAGT#CTGTCTCAGTGTGCTTGCA#...(((((((((...((((((.#))))))...))))))))).	19	9	3UTR
mmu-miR-214-3p	NM_001081400	1810013L24Rik	ACAGCAGGCACAGACAGGCAGT#ACTGCCGCTCCTGTCCCTTCCTCCCTGGCCTGCC#...((((((((((...((((((#))))))....))))............)))))).	34	6	3UTR
mmu-miR-214-3p	NM_001145999	Zfp710	ACAGCAGGCACAGACAGGCAGT#GCTGCCATGTCTGTGTCGAATGCG#...(((((((((((((((((#))))))).)))))))...))).	24	10	CDS
mmu-miR-214-3p	NM_001145999	Zfp710	ACAGCAGGCACAGACAGGCAGT#ATGAGGTGTCTGTGCCTGGTGA#.((.((((((((((((......#.)))))))))))).)).	17	12	CDS
mmu-miR-214-3p	NM_001146000	Zfp710	ACAGCAGGCACAGACAGGCAGT#GCTGCCATGTCTGTGTCGAATGCG#...(((((((((((((((((#))))))).)))))))...))).	24	10	CDS

mmu-miR-214-3p	NM_001146000	Zfp710	ACAGCAGGCACAGACAGGCAGT#ATGAGGTGTCTGTGCCTGGTGA#.((.((((((((((((......#.)))))))))))).)).	17	12	CDS
mmu-miR-214-3p	NM_001291016	Bcl2l11	ACAGCAGGCACAGACAGGCAGT#TTGCCCCTGATTCTGTCCTGCA#...(((((.((((((((((((.#))))))..))..))))))))).	22	10	3UTR
mmu-miR-214-3p	NM_001291222	Kif7	ACAGCAGGCACAGACAGGCAGT#ACTGCCTGTTTTGCTTCTA#..((.(((((.(((((((((((#)))))))))))))))))).	19	18	3UTR
mmu-miR-214-3p	NM_001291222	Kif7	ACAGCAGGCACAGACAGGCAGT#TGACCTGCTGGACTGCCTGGTGC#.((.((((((((((.((((((..#)).)))))))...)))))).)).	23	7	CDS
mmu-miR-214-3p	NM_001291248	Cpsf4	ACAGCAGGCACAGACAGGCAGT#GCTGCTTGTCTGGGCTTGCTTGTG#(((((((((.((((((((((((#)))))))))))).))))))).)).	24	12	3UTR
mmu-miR-214-3p	NM_001291249	Cpsf4	ACAGCAGGCACAGACAGGCAGT#GCTGCTTGTCTGGGCTTGCTTGTG#(((((((((.((((((((((((#)))))))))))).))))))).)).	24	12	3UTR
mmu-miR-214-3p	NM_001356265	Zfand3	ACAGCAGGCACAGACAGGCAGT#GCTGCCCACGTCTGGCTGCTGA#.(((((.((.(((((.((((((#))))))...)))))))))))).	22	12	3UTR
mmu-miR-214-3p	NM_001356265	Zfand3	ACAGCAGGCACAGACAGGCAGT#CTGGCTGGTTACTAAGGTGCCTGCTA#..(((((((((...(((.(((.#))).)))..........))))))))).	26	9	3UTR
mmu-miR-214-3p	NM_001356265	Zfand3	ACAGCAGGCACAGACAGGCAGT#ACATGCCTGTCACTGCTGG#.((((((.....((((((((((#)).)))))))).)))))).	19	8	3UTR
mmu-miR-214-3p	NM_133828	Creb1	ACAGCAGGCACAGACAGGCAGT#TTGTCCTGCTGAGCTTGCTGG#.((((((((.(((.(((((((.#))).))))))).)))))))).	21	8	3UTR
mmu-miR-214-3p	NM_133828	Creb1	ACAGCAGGCACAGACAGGCAGT#GCTGCCTGTGGCCCGGGCGGCTGG#.((((.(.(....((((((((((#))))))))))......).).)))).	24	9	5UTR
mmu-miR-214-3p	NM_133828	Creb1	ACAGCAGGCACAGACAGGCAGT#CTGTCTGTGCTAGCA#...((.(((((((((((.....#))))))))))).)).	15	11	3UTR

mmu-miR-214-3p	NM_148926	Zfand3	ACAGCAGGCACAGACAGGCAGT#GCTGCCCACGTCTGGCTGCTGA#.(((((.((.(((((.((((((#))))))...)))))))))))).	22	12	3UTR
mmu-miR-214-3p	NM_148926	Zfand3	ACAGCAGGCACAGACAGGCAGT#CTGGCTGGTTACTAAGGTGCCTGCTA#..(((((((((...(((.(((.#))).))).........))))))))).	26	9	3UTR
mmu-miR-214-3p	NM_148926	Zfand3	ACAGCAGGCACAGACAGGCAGT#ACATGCCTGTCACTGCTGG#.((((((.....((((((((((#)).)))))))).)))))).	19	8	3UTR
mmu-miR-214-3p	NM_172947	Lsm12	ACAGCAGGCACAGACAGGCAGT#CTGTGACTTTCCTTCCCTAGTCTGTGTCTGCC#...((((((((((((((....#.))).))))))))))).	18	12	3UTR
mmu-miR-214-3p	NM_174846	Glyctk	ACAGCAGGCACAGACAGGCAGT#CTGATCATGGTTTCTCCTCTCCTGCTGTTGCCTGACTGG#.((((((((((((.((((.((.#.)))))))))).))))).))).	22	10	3UTR
mmu-miR-214-3p	NM_174846	Glyctk	ACAGCAGGCACAGACAGGCAGT#ACTGTCACCATTGCCTGCTGA#.(((((((((......((((((#)))))).....))))))))).	21	9	3UTR
mmu-miR-214-3p	NM_175187	Tmem161b	ACAGCAGGCACAGACAGGCAGT#ACTGTCTGTCCCACGACCTGTTC#..(((((((...((((((((((#))))))))))....).)))))).	23	10	5UTR
mmu-miR-214-3p	NM_175187	Tmem161b	ACAGCAGGCACAGACAGGCAGT#ACTGTTAGTTTTGGGCCTTGCTGTA#(((((((((.(((((.((((((#)))))).)))).).)))).))))).	25	6	3UTR
mmu-miR-214-3p	NM_175303	Sall4	ACAGCAGGCACAGACAGGCAGT#GCTGCCTGTGTCGCTGGGGGCCAGCTC#..(((.(((.(((((((((((((#))))))))))....)))...))).))).	27	9	CDS
mmu-miR-214-3p	NM_175303	Sall4	ACAGCAGGCACAGACAGGCAGT#CCTGGAGCAGATCCTGTGCCTGCA#...((((((((((....((...#.)).....)))))))))).	19	10	CDS
mmu-miR-214-3p	NM_175433	Zfp710	ACAGCAGGCACAGACAGGCAGT#GCTGCCATGTCTGTGTCGAATGCG#...((((((((((((((((((((#))))))).))))))))))...))).	24	10	CDS
mmu-miR-214-3p	NM_175433	Zfp710	ACAGCAGGCACAGACAGGCAGT#ATGAGGTGTCTGTGCCTGGTGA#.((.((((((((((((......#.)))))))))))).)).	17	12	CDS

mmu-miR-214-3p	NM_178576	Cpsf4	ACAGCAGGCACAGACAGGCAGT#GCTGCTTGTCTGGGCTTGCTTGTG#(((((((((.((((((((((((#)))))))))))).))))))).)).	24	12	3UTR
mmu-miR-214-3p	NM_201395	Sall4	ACAGCAGGCACAGACAGGCAGT#GCTGCCTGTGTCGCTGGGGGCCAGCTC#..(((.(((.((((((((((((#)))))))))....)))...))).))).	27	9	CDS
mmu-miR-214-3p	NM_201395	Sall4	ACAGCAGGCACAGACAGGCAGT#CCTGGAGCAGATCCTGTGCCTGCA#...((((((((((....((...#.)).....)))))))))).	19	10	CDS
mmu-miR-214-3p	NM_201396	Sall4	ACAGCAGGCACAGACAGGCAGT#GCTGCCTGTGTCGCTGGGGGCCAGCTC#..(((.(((.((((((((((((#)))))))))....)))...))).))).	27	9	CDS
mmu-miR-214-3p	NM_201396	Sall4	ACAGCAGGCACAGACAGGCAGT#ACTGCCTGTTTCCTAGTGG#.((..(((...(((((((((((#))))))))))))))..)).	19	14	3UTR
mmu-miR-214-3p	NM_207680	Bcl2l11	ACAGCAGGCACAGACAGGCAGT#ACTGCGGTGGCTGTGTTTGCC#...((((((((((.((.(((((#)))))..)).)))))))))).	21	10	3UTR
mmu-miR-214-3p	NM_207681	Bcl2l11	ACAGCAGGCACAGACAGGCAGT#ACTGCGGTGGCTGTGTTTGCC#...((((((((((.((.(((((#)))))..)).)))))))))).	21	10	3UTR
mmu-miR-214-3p	XM_006495650	Creb1	ACAGCAGGCACAGACAGGCAGT#TTGTCCTGCTGAGCTTGCTGG#.((((((((.(((.(((((((.#))).))))))).)))))))).	21	8	3UTR
mmu-miR-214-3p	XM_006495650	Creb1	ACAGCAGGCACAGACAGGCAGT#GCTGGCCTGTCCCACTGCTGC#.((((((.....((((((((((#)))).))))))...)))))).	21	6	3UTR
mmu-miR-214-3p	XM_006495650	Creb1	ACAGCAGGCACAGACAGGCAGT#CTGTCTGTGCTAGCA#...((.(((((((((((.....#))))))))))).)).	15	11	3UTR
mmu-miR-214-3p	XM_006495651	Creb1	ACAGCAGGCACAGACAGGCAGT#TTGTCCTGCTGAGCTTGCTGG#.((((((((.(((.(((((((.#))).))))))).)))))))).	21	8	3UTR
mmu-miR-214-3p	XM_006495651	Creb1	ACAGCAGGCACAGACAGGCAGT#GCTGGCCTGTCCCACTGCTGC#.((((((.....((((((((((#)))).))))))...)))))).	21	6	3UTR

mmu-miR-214-3p	XM_006495651	Creb1	ACAGCAGGCACAGACAGGCAGT#CTGTCTGTGCTAGCA#...((.(((((((((((.....#)))))))))))).)).	15	11	3UTR
mmu-miR-214-3p	XM_006540668	Kif7	ACAGCAGGCACAGACAGGCAGT#ACTGCCTGTTTTGCTTCTA#..((.(((((.(((((((((((#)))))))))))))))))).	19	18	3UTR
mmu-miR-214-3p	XM_006540668	Kif7	ACAGCAGGCACAGACAGGCAGT#TGACCTGCTGGACTGCCTGGTGC#.((.((((((((((.((((((..#)).)))))))...)))))).)).	23	7	CDS
mmu-miR-214-3p	XM_030244128	Pml	ACAGCAGGCACAGACAGGCAGT#GCTGCCTTGCCTGCA#...(((((((.....(((((((#))))))))))))))).	15	14	CDS
mmu-miR-214-3p	NM_001361909	Tmem161b	ACAGCAGGCACAGACAGGCAGT#ACTGTCTGTCCCACGACCTGTTC#..((((((((...((((((((((#))))))))))....).)))))).	23	10	5UTR
mmu-miR-214-3p	NM_001361909	Tmem161b	ACAGCAGGCACAGACAGGCAGT#ACTGTTAGTTTTGGGCCTTGCTGTA#(((((((((.(((((.((((((#)))))).)))).).)))).))))).	25	6	3UTR
mmu-miR-214-3p	NM_001361987	Fbln5	ACAGCAGGCACAGACAGGCAGT#GCTGTGAGACAAGCAGAAGTCTCCGAACACTTCTGTCTGCGTTTGCTC#..(((((((.((((((((.(((#.))))))))))).))))))).	21	11	5UTR
mmu-miR-214-3p	NM_001361987	Fbln5	ACAGCAGGCACAGACAGGCAGT#ACCGCTGTATGTGTCCTGCTGA#.(((((((((((.(((.((.((#)).))))).)))).))))))).	22	7	CDS
mmu-miR-214-3p	NM_001374716	Cpsf4	ACAGCAGGCACAGACAGGCAGT#GCTGCTTGTCTGGGCTTGCTTGTG#(((((((((.((((((((((((#)))))))))))).))))))).)).	24	12	3UTR
mmu-miR-214-3p	NM_008465	Kpna1	ACAGCAGGCACAGACAGGCAGT#ACTCCCGTCTGTCCTGTTGTT#((((((((.((((((.((.(((#))))).)))))))))))))).	21	14	3UTR
mmu-miR-214-3p	NM_008465	Kpna1	ACAGCAGGCACAGACAGGCAGT#CTGCTCTGGGATGGATGCTGTCTGATGTCTGTTA#..((((((((((((((((((.#))))............))))))).))))))).	34	8	3UTR
mmu-miR-214-3p	NM_009443	Tgoln1	ACAGCAGGCACAGACAGGCAGT#GTTTGTTTTCCTGCTGTG#((((((((...((((((((...#)))))))).)))))))).	18	8	3UTR

mmu-miR-214-3p	NM_009754	Bcl2l11	ACAGCAGGCACAGACAGGCAGT#ACTGCGGTGGCTGTGTTTGCC#...((((((((((.((.(((((#)))))..)).)))))))))))).	21	10	3UTR
mmu-miR-214-3p	NM_009952	Creb1	ACAGCAGGCACAGACAGGCAGT#TTGTCCTGCTGAGCTTGCTGG#.((((((((.((((.(((((((.#))).))))))).)))))))).	21	8	3UTR
mmu-miR-214-3p	NM_009952	Creb1	ACAGCAGGCACAGACAGGCAGT#GCTGCCTGTGGCCCGGGCGGCTGG#.((((.(.(....(((((((((#)))))))))......).).)))).	24	9	5UTR
mmu-miR-214-3p	NM_009952	Creb1	ACAGCAGGCACAGACAGGCAGT#CTGTCTGTGCTAGCA#...((.(((((((((((.....#)))))))))))).)).	15	11	3UTR
mmu-miR-214-3p	NM_010626	Kif7	ACAGCAGGCACAGACAGGCAGT#ACTGCCTGTTTTGCTTCTA#..((.(((((.(((((((((((#))))))))))))))))))).	19	18	3UTR
mmu-miR-214-3p	NM_010626	Kif7	ACAGCAGGCACAGACAGGCAGT#TGACCTGCTGGACTGCCTGGTGC#.((.(((((((((.((((((..#)).)))))))...)))))).)).	23	7	CDS
mmu-miR-214-3p	NM_011812	Fbln5	ACAGCAGGCACAGACAGGCAGT#ACCGCTGTATGTGTCCTGCTGA#.(((((((((((.(((.((.((#)).))))).)))).))))))).	22	7	CDS
mmu-miR-214-3p	NM_011812	Fbln5	ACAGCAGGCACAGACAGGCAGT#ACTGTGTGTGTGTGTTTTGTTT#..((((((((((.(((.(((((#))))).))).))))))).))).	22	7	3UTR
mmu-miR-214-3p	NM_011812	Fbln5	ACAGCAGGCACAGACAGGCAGT#GCTGCTTGTTTTACCCACTTGTTGG#.(((((((...(((((((((((#)))))))))))......))))))).	25	11	3UTR
mmu-miR-214-3p	NM_019405	Cetn2	ACAGCAGGCACAGACAGGCAGT#AGTGCTTGCTGTGGCATTTGG#(((((((((((..........#.))))))))))).	13	11	3UTR
mmu-miR-214-3p	NM_025885	Med19	ACAGCAGGCACAGACAGGCAGT#ACTGGACTTGGCCAAGGATGGCTTTCCTGTGATACTGAACCTGCTGA#.(((((((..((((((((.(((#.)))..)))))....)))..))))))).	28	7	3UTR
mmu-miR-214-3p	NM_027854	Tmem248	ACAGCAGGCACAGACAGGCAGT#CTGTCTCAGTGTGCTTGCA#...(((((((((...((((((.#))))))...))))))))).	19	9	3UTR

mmu-miR-214-3p	NM_028355	Ndc1	ACAGCAGGCACAGACAGGCAGT#GCCTGGCTAGATAACAGAATTAAAGCCATAGGCCTTTTGTGTTTGCTGG#.(((((((((((((.((((...#.))))))))))))))))).	19	17	3UTR
mmu-miR-214-3p	NM_028355	Ndc1	ACAGCAGGCACAGACAGGCAGT#CCATGTCTGCCTGCTGG#.((((((((..(((((((....#)).))))))))))))).	17	13	3UTR
mmu-miR-214-3p	NM_053089	Naa15	ACAGCAGGCACAGACAGGCAGT#GGTGTGTGTGTGTGTGTGTGTGTG#((((((.(((((.(((.(((.(#).))).))).))))).))).))).	24	5	3UTR
mmu-miR-214-3p	NM_053089	Naa15	ACAGCAGGCACAGACAGGCAGT#CTGTCTGTTTGTCTTGA#....((((.((((((((((((.#)))))))))))))))).	17	16	3UTR
mmu-miR-214-3p	NM_053089	Naa15	ACAGCAGGCACAGACAGGCAGT#GCTGTTGTCTAAGCTTTGCTGA#.((((((((..(((((.(((((#))))))))))..)))).))))).	22	10	3UTR
