# Supplementary material for: Bioinformatic prediction, qCLASH, and AlphaFold3 reveal non-overlapping layers of the miR-214-3p–mRNA interactome
Source: Front Genet. 2026 Sep 30;17:1924314. doi: 10.3389/fgene.2026.1924314 (PMC13661022; doi:10.3389/fgene.2026.1924314)
Supplement: Supplementary file 8 [file DataSheet6.pdf]

Appendix Table 1. Complementarity and minimum free energy (MFE) analysis of miR-214-3p–mRNA interactions detected via qCLASH

miR-214-3p—mRNA[1] hybrids (5'→3')	Counts	Base complementary pairing between miR-214-3p and target mRNA		Site region	MFE[2]	Expression levels in mouse lung
		mRNA Name	Reverse base complementary pairing			
ACAGCAGGCACAGACAGGCAGTATATT**TTGCTGCTGCTGCCGCTGCTGC**CTTGTCTTCTGCTCCTG	7	Adgra2	target 5' U G C GC C 3'<br>UGCU CUG UGCC UGCUG<br>ACGG GAC ACGG ACGAC<br>miRNA 3' G ACA A 5'	CDS	-27.0	High
ACAGCAGGCACAGACAGGCAGTGTATAG**ACTGCCTGACATCTGCTGGTGG**CTCT	6	Rhob	target 5' A A A G 3'<br>CUGCCUG C UCUGCUG<br>GACGGAC G GGACGAC<br>miRNA 3' A ACAC A 5'	3'UTR	-26.9	-
ACAGCAGGCACAGACAGGCAGTAAA**GTGCCTGTTCAAAGTCCTGCA**TAAGGGGATCCCTGT	5	Ccnd2	target 5' G CAAA A 3'<br>UGCCUGUU GU CCUGC<br>ACGGACAG CA GGACG<br>miRNA 3' G A C ACA 5'	3'UTR	-27.7	Medium
ACAGCAGGCACAGACAGGCAGTT**CGCCTGCTGCCTGCC**TCCTTCCCCTG	4	Oplah	target 5' C C 3'<br>GCCUG CU GCCUGC<br>CGGAC GA CGGACG<br>miRNA 3' GA A CA ACA 5'	CDS	-27.1	Medium
ACAGCAGGCACAGACAGGCAGTAA**CTCTGTCATGTCCTGCTGA**TCG	4	Sec24c	target 5' C A A 3'<br>UCUGUC UGU CCUGCUG<br>GGACAG ACA GGACGAC<br>miRNA 3' GAC C A 5'	3'UTR	-28.8	High
TACAGCAGGCACAGACAGGCAGGCCGCT**CCTGCCTGTCACTGCTGC**GGCGCTG	1	Cd47	target 5' C A C 3'<br>CUGCCUGUC CUGCUG<br>GACGGACAG GACGAC<br>miRNA 3' ACACG A 5'	5'UTR	-30.6	High
ACAGCAGGCACAGACAGGCAG**TACTGGGACCTGCTAACGCTGCTGG**AAGGGGCT	3	Notch4	target 5' A GGA AACG G 3'<br>CUG CCUG CU CUGCUG<br>GAC GGAC GA GACGAC<br>miRNA 3' A CACG A 5'	CDS	-21.3	High
ACAGCAGGCACAGACAGGCAGTACCTGGTAC**CGCCTGACAACTGCTGCCTCCCTCCTGG**AT	2	SUN2	target 5' C ACAA C CCCUC G 3'<br>GCCUG CUG UGCCU CUG<br>CGGAC GAC ACGGA GAC<br>miRNA 3' GA A C A 5'	CDS	-25.8	High

ACAGCAGGCACAGACAGGCAGTATA**AGCCTGTCATCCTGCTGC**AGGGACTC	2	Slc19a2	target 5' A AU C 3'<br>GCCUGUC CCUGCUG<br>CGGACAG GGACGAC<br>miRNA 3' GA ACAC A 5'	CDS	-30.8	Low
ACAGCAGGCACAGACAGGCAGTATACCTGTTCTTTTTCAGGGAAC**ACTGCCTGTCTGTCT GCT**TGGTTCTCTCTCTG	2	Tppp	target 5' A 3'<br>CUGCCUGUCUGU CUGCCU<br>GACGGACAGACA GACGA<br>miRNA 3' CG CA 5'	3'UTR	-33.8	Medium
ACAGCAGGCACAGACAGGCAGACAT**CCCTGAAGCCTGCTGA**GATCCTCT	1	Mtmr14	target 5' C AA A 3'<br>CCUG GCCUGCUG<br>GGAC CGGACGAC<br>miRNA 3' GAC AGACA A 5	CDS	-25.2	Medium
ACAGCAGGCACAGACAGGCAGA**ATGACTGGCCGGCCTGCTGA**AACCAGGAG	1	Inmt	target 5' A A GCCG A 3'<br>UG CUG GCCUGCUG<br>AC GAC CGGACGAC<br>miRNA 3' G G AGACA A 5'	3'UTR	-27.1	Extremely High
ACAGCAGGCACAGACAGGCAGTAAA**CCCTGTTGAGCTTGACTC**	11	unannotated gene 1[3]	target 5' C GA A C 3'<br>CCUGUU GCUUG CU<br>GGACAG CGGAC GA<br>miRNA 3' GAC ACA CA 5'	unknown	-20.3	low
ACAGCAGGCACAGACAGGCAGTAAAGCATCGCGAAGGCCCGCGGCGGGTGTTGACGCGAT GTGATT**TCTGCCCAGTGCTCTGAATGT**	8	unannotated gene 2[4]	target 5' U CA U AA 3'<br>CUGCC GUGC CUG UGU<br>GACGG CACG GAC ACA<br>miRNA 3' ACAGA G 5'	unknown	-21.1	Extremely low
ACAGCAGGCACAGACAGGCAGTAGGACTCTCGATTCAGCAAGATCATGTGGAACT**TCTGC TTCGGTGCCTTGGGGC**ACAAATC	8	Map3k6	target 5' U G UGGG A 3'<br>CUGCU UC GUGCCU GC<br>GACGG AG CACGGA CG<br>miRNA 3' AC A ACA 5'	CDS	-24.3	Medium
ACAGCAGGCACAGACAGGCAGTAA**GCTGCCGGTTCTGTTGGTCCTGTTGG**TGCTGTTG GTC	4	Col1a2	target 5' G G U UG U G 3'<br>CUGCC G UCUGU G CCUGUUG<br>GACGG C AGACA C GGACGAC<br>miRNA 3' A A 5'	CDS	-29.7	High
ACAGCAGGCACAGACAGGCAGTAGGACTCTCGATTCAGCAAGATCATGTGGAACT**TCTGC TTCGGTGCCTTGGGGCA**CACATC	1	Map3k6	target 5' U G UGGG A 3'<br>CUGCU UC GUGCCU GC<br>GACGG AG CACGGA CG<br>miRNA 3' AC A ACA 5'	CDS	-24.3	Medium

ACAGCAGGCACAGACAGGCAGT**ACCTGTCTTGGGCCTGCTGG**GC	80	unannotated gene 3[5]	target 5' A UGG G 3'<br>CCUGUCU GCCUGCUG<br>GGACAGA CGGACGAC<br>miRNA 3' GAC CA A 5'	unknown	-33.7	Extremely High
ACAGCAGGCACAGACAGGCAGTTTTGTTC**GCTGCCTGCTGCTGCTGTC**GCCGTGCCGCGGGT	47	Mcam	target 5' G C 3'<br>CUGCCUG CUG CUGCUGU<br>GACGGAC GAC GACGACA<br>miRNA 3' A ACG 5'	CDS	-30.0	Extremely High
ACAGCAGGCACAGACAGGCAGT**ACCTGTCTTGGGCCTGCCGG**GC	2	unannotated gene 3[5]	target 5' A UGG C G 3'<br>CCUGUCU GCCUGC G<br>GGACAGA CGGACG C<br>miRNA 3' GAC CA A A 5'	unknown	-29.2	Extremely High
ACAGCAGGCACAGACAGGCAGT**ACCGGTCTTGGGCCTGCTGG**GC	1	unannotated gene 3[5]	target 5' A G UGG G 3'<br>CC GUCU GCCUGCUG<br>GG CAGA CGGACGAC<br>miRNA 3' GAC A CA A 5	unknown	-29.6	Extremely High

Note:

[1]: The entire mRNA sequence is shown uniformly in black, with the miRNA-complementary target site in bold black (miRNA is shown in red).

[2]: refers to the minimum free energy in kcal/mol.

[3]: unannotated gene 1: Mouse DNA sequence from clone RP23-74C21 on chromosome 17, complete sequence

[4]: unannotated gene 2: Mus musculus, clone IMAGE:3499050, mRNA

[5]: unannotated gene 3: Mus musculus BAC clone RP24-571D21 from chromosome 12, complete sequence
