# Supplementary material for: Bioinformatic prediction, qCLASH, and AlphaFold3 reveal non-overlapping layers of the miR-214-3p–mRNA interactome
Source: Front Genet. 2026 Sep 30;17:1924314. doi: 10.3389/fgene.2026.1924314 (PMC13661022; doi:10.3389/fgene.2026.1924314)
Supplement: Supplementary file 9 [file DataSheet7.pdf]

Appendix Table 2. AlphaFold3 models of the miR-214-3p miRISC with consensus target mRNAs from three bioinformatics methods.

Number	Target genes	miR-214-3p#target mRNA sequences	ipTM	pTM
1	Zfand3	ACAGCAGGCACAGACAGGCAGT#AC ATGCCTGTCACTGCTGG	0.79	0.9
2	Fbln5	ACAGCAGGCACAGACAGGCAGT#AC CGCTGTATGTGTCCTGCTGA	0.54	0.88
3	Kpna1	ACAGCAGGCACAGACAGGCAGT#AC TCCCGTCTGTCCTGTTGTT	0.54	0.88
4	1810013L24Rik	ACAGCAGGCACAGACAGGCAGT#AC TGCCGCTCCTGTCCCTTCCTCCCTGGC CTGCC	0.62	0.86
5	Sall4	ACAGCAGGCACAGACAGGCAGT#AC TGCCTGTTTCCTAGTGG	0.82	0.9
6	Kif7	ACAGCAGGCACAGACAGGCAGT#AC TGCCTGTTTTGCTTCTA	0.71	0.89
7	Bcl2l11	ACAGCAGGCACAGACAGGCAGT#AC TGCGGTGGCTGTGTTTGCC	0.57	0.87
8	Med19	ACAGCAGGCACAGACAGGCAGT#AC TGGACTTGGCCAAGGATGGCTTTCCT GTGATACTGAACCTGCTGA	0.75	0.87
9	Glyctk	ACAGCAGGCACAGACAGGCAGT#AC TGTCACCATTGCCTGCTGA	0.71	0.88
10	Tmem161b	ACAGCAGGCACAGACAGGCAGT#AC TGTCTGTCCCACGACCTGTTC	0.63	0.89
11	Fbln5	ACAGCAGGCACAGACAGGCAGT#AC TGTGTGTGTGTGTTTTGTTT	0.65	0.89
12	Tmem161b	ACAGCAGGCACAGACAGGCAGT#AC TGTTAGTTTTGGGCCTTGCTGTA	0.59	0.89
13	Cetn2	ACAGCAGGCACAGACAGGCAGT#AG TGCTTGCTGTGGCATTTGG	0.74	0.88
14	Zfp710	ACAGCAGGCACAGACAGGCAGT#AT GAGGTGTCTGTGCCTGGTGA	0.62	0.87
15	Ndc1	ACAGCAGGCACAGACAGGCAGT#CC ATGTCTGCCTGCTGG	0.73	0.89
16	Sall4	ACAGCAGGCACAGACAGGCAGT#CC TGGAGCAGATCCTGTGCCTGCA	0.71	0.88
17	Glyctk	ACAGCAGGCACAGACAGGCAGT#CT GATCATGGTTTCTCCTCTCCTGCTGTT GCCTGACTGG	0.57	0.86
18	Kpna1	ACAGCAGGCACAGACAGGCAGT#CT GCTCTGGGATGGATGCTGTCTGATGT CTGTTA	0.5	0.86

19	Zfand3	ACAGCAGGCACAGACAGGCAGT#CTGGCTGGTTACTAAGGTGCCTGCTA	0.71	0.88
20	Tmem248	ACAGCAGGCACAGACAGGCAGT#CTGTCTCAGTGTGCTTGCA	0.51	0.88
21	Creb1	ACAGCAGGCACAGACAGGCAGT#CTGTCTGTGCTAGCA	0.76	0.87
22	Naa15	ACAGCAGGCACAGACAGGCAGT#CTGTCTGTTTGTCTTGA	0.8	0.89
23	Lsm12	ACAGCAGGCACAGACAGGCAGT#CTGTGACTTTCCTTCCCTAGTCTGTGTCTGCC	0.74	0.88
24	Ndc1	ACAGCAGGCACAGACAGGCAGT#GCCTGGCTAGATAACAGAATTAAAGCCATAGGCCTTTTGTGTTTGCTGG	0.58	0.85
25	Rapgefl1	ACAGCAGGCACAGACAGGCAGT#GCGTGTCTCGCCTGCTGC	0.69	0.89
26	Zfp710	ACAGCAGGCACAGACAGGCAGT#GCTGCCATGTCTGTGTCGAATGCG	0.61	0.87
27	Zfand3	ACAGCAGGCACAGACAGGCAGT#GCTGCCCACGTCTGGCTGCTGA	0.76	0.89
28	Creb1	ACAGCAGGCACAGACAGGCAGT#GCTGCCTGTGGCCCGGGCGGCTGG	0.66	0.73
29	Sall4	ACAGCAGGCACAGACAGGCAGT#GCTGCCTGTGTCGCTGGGGGCCAGCTC	0.63	0.71
30	Pml	ACAGCAGGCACAGACAGGCAGT#GCTGCCTTGCCTGCA	0.87	0.9
31	Cpsf4	ACAGCAGGCACAGACAGGCAGT#GCTGCTTGTCTGGGCTTGCTTGTG	0.49	0.87
32	Fbln5	ACAGCAGGCACAGACAGGCAGT#GCTGCTTGTTTTACCCACTTGTTGG	0.66	0.73
33	Creb1	ACAGCAGGCACAGACAGGCAGT#GCTGGCCTGTCCCACTGCTGC	0.77	0.89
34	Fbln5	ACAGCAGGCACAGACAGGCAGT#GCTGTGAGACAAGCAGAAGTCTCCGAACACTTCTGTCTGCGTTTGCTC	0.4	0.84
35	Naa15	ACAGCAGGCACAGACAGGCAGT#GCTGTTGTCTAAGCTTTGCTGA	0.59	0.88
36	Naa15	ACAGCAGGCACAGACAGGCAGT#GGTGTGTGTGTGTGTGTGTGTGTG	0.54	0.89
37	Tgoln1	ACAGCAGGCACAGACAGGCAGT#GTTTGTTTTCCTGCTGTG	0.88	0.9
38	Kif7	ACAGCAGGCACAGACAGGCAGT#TGACCTGCTGGACTGCCTGGTGC	0.64	0.88

39	Bcl2l11	ACAGCAGGCACAGACAGGCAGT#TT GCCCCTGATTCTGTCCTGCA	0.74	0.89
40	Creb1	ACAGCAGGCACAGACAGGCAGT#TT GTCCTGCTGAGCTTGCTGG	0.47	0.89

Note: Consensus targets were identified using TargetScan 8.0, miRDB, and miRWalk 3.0.
