# Supplementary material for: Bioinformatic prediction, qCLASH, and AlphaFold3 reveal non-overlapping layers of the miR-214-3p–mRNA interactome
Source: Front Genet. 2026 Sep 30;17:1924314. doi: 10.3389/fgene.2026.1924314 (PMC13661022; doi:10.3389/fgene.2026.1924314)
Supplement: Supplementary file 10 [file DataSheet8.pdf]

Appendix Table 3. AlphaFold3 models of the miR-214-3p/AGO2 miRISC with qCLASH-identified target mRNAs.

Number	Target genes	Sequence	ipTM	pTM
1	Adgra2	ACAGCAGGCACAGACAGGCAGTATATT**TTGCTGCTGCTGCCGCTGCTGC**CTTGTCTTCTGCTCCTG	0.49	0.86
2	Rhob	ACAGCAGGCACAGACAGGCAGTGTATAG**ACTGCCTGACATCTGCTGGTGG**CTCT	0.76	0.9
3	Ccnd2	ACAGCAGGCACAGACAGGCAGTAAA**GTGCCTGTTCAAAGTCCTGCA**TAAGGGGATCCCTGT	0.86	0.89
4	Oplah	ACAGCAGGCACAGACAGGCAGTT**CGCCTGCTGCCTGCC**TCCTTCCCCTG	0.88	0.9
5	Sec24c	ACAGCAGGCACAGACAGGCAGTAA**CTCTGTCATGTCCTGCTGA**TCG	0.7	0.89
6	Cd47	TACAGCAGGCACAGACAGGCAGGCCGCT**CCTGCCTGTCACTGCTGC**GGCGCTG	0.79	0.9
7	Notch4	ACAGCAGGCACAGACAGGCAGT**ACTGGGACCTGCTAACGCTGCTGG**AAGGGGCT	0.66	0.87
8	SUN2	ACAGCAGGCACAGACAGGCAGTACCTGGTAC**CGCCTGACAACTGCTGCCTCCCTCCTGG**AT	0.86	0.9
9	Slc19a2	ACAGCAGGCACAGACAGGCAGTATA**AGCCTGTCATCCTGCTGC**AGGGACTC	0.76	0.9
10	Tppp	ACAGCAGGCACAGACAGGCAGTATACCTGTTCTTTTTCAGGGAAC**ACTGCCTGTCTGTCTGCT**TGGTTCTCTCTCTG	0.75	0.9
11	Mtmr14	ACAGCAGGCACAGACAGGCAGACAT**CCCTGAAGCCTGCTGA**GATCCTCT	0.87	0.91
12	Inmt	ACAGCAGGCACAGACAGGCAGA**ATGACTGGCCGGCCTGCTGA**AACCAGGAGG	0.57	0.89
13	unannotated gene 1	ACAGCAGGCACAGACAGGCAGTAAA**CCCTGTTGAGCTTGACTC**	0.66	0.9
14	unannotated gene 2	ACAGCAGGCACAGACAGGCAGTAAAGCATCGCGAAGGCCCGCGGCGGGTGTTGACGCGATGTGATT**TCTGCCCAGTGCTCTGAATGT**	0.69	0.9

15	Map3k6	ACAGCAGGCACAGACAGGCAGTAGGACTCTCGATTCAGCAAGATCATGTGGAACT**TCTGCTTCGGTGCCTTGGGGC**ACAAATC	0.82	0.9
16	Col1a2	ACAGCAGGCACAGACAGGCAGTAA**GCTGCCGGTTCTGTTGGTCCTGTTGG**TGCTGTTGGTC	0.6	0.88
17	Map3k6	ACAGCAGGCACAGACAGGCAGTAGGACTCTCGATTCAGCAAGATCATGTGGAACT**TCTGCTTCGGTGCCTTGGGGCA**CACATC	0.75	0.9
18	unannotated gene 3	ACAGCAGGCACAGACAGGCAGT**ACCTGTCTTGGGCCTGCTGG**GC	0.6	0.89
19	Mcam	ACAGCAGGCACAGACAGGCAGTTTTGTTC**GCTGCCTGCTGCTGCTGTC**GCCGTGCCGCGGGT	0.55	0.89
20	unannotated gene 3	ACAGCAGGCACAGACAGGCAGT**ACCTGTCTTGGGCCTGCCGG**GC	0.58	0.88
21	unannotated gene 3	ACAGCAGGCACAGACAGGCAGT**ACCGGTCTTGGGCCTGCTGG**GC	0.61	0.89

Note: Data source from qCLASH.
