# Supplementary material for: Bioinformatic prediction, qCLASH, and AlphaFold3 reveal non-overlapping layers of the miR-214-3p–mRNA interactome
Source: Front Genet. 2026 Sep 30;17:1924314. doi: 10.3389/fgene.2026.1924314 (PMC13661022; doi:10.3389/fgene.2026.1924314)
Supplement: Supplementary file 11 [file DataSheet9.pdf]

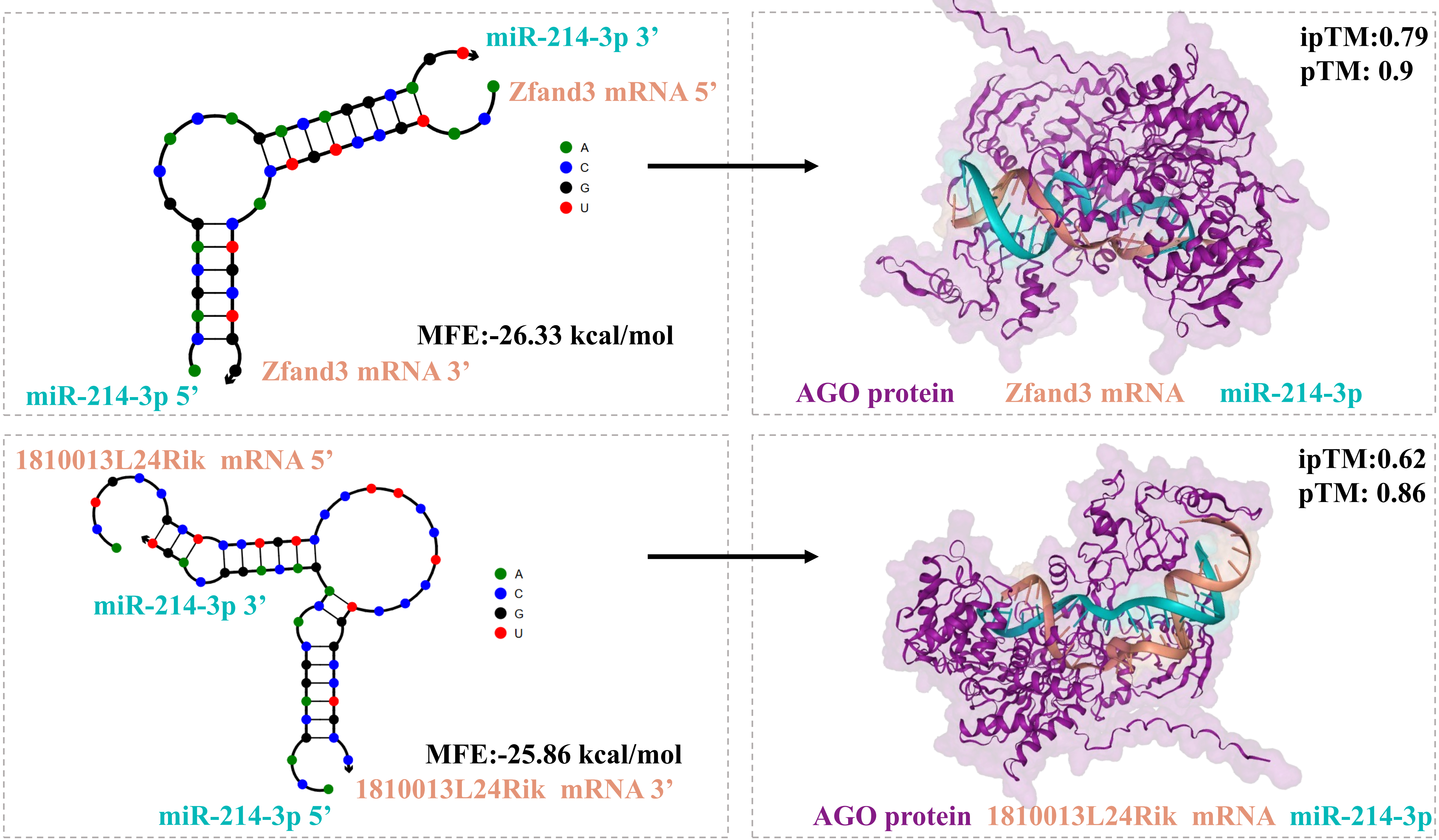

miR-214-3p 3'
Zfand3 mRNA 5'
A
C
G
U
MFE:-26.33 kcal/mol
Zfand3 mRNA 3'
miR-214-3p 5'
ipTM:0.79
pTM: 0.9
AGO protein
Zfand3 mRNA
miR-214-3p
1810013L24Rik mRNA 5'
miR-214-3p 3'
A
C
G
U
MFE:-25.86 kcal/mol
1810013L24Rik mRNA 3'
miR-214-3p 5'
ipTM:0.62
pTM: 0.86
AGO protein
1810013L24Rik mRNA
miR-214-3p

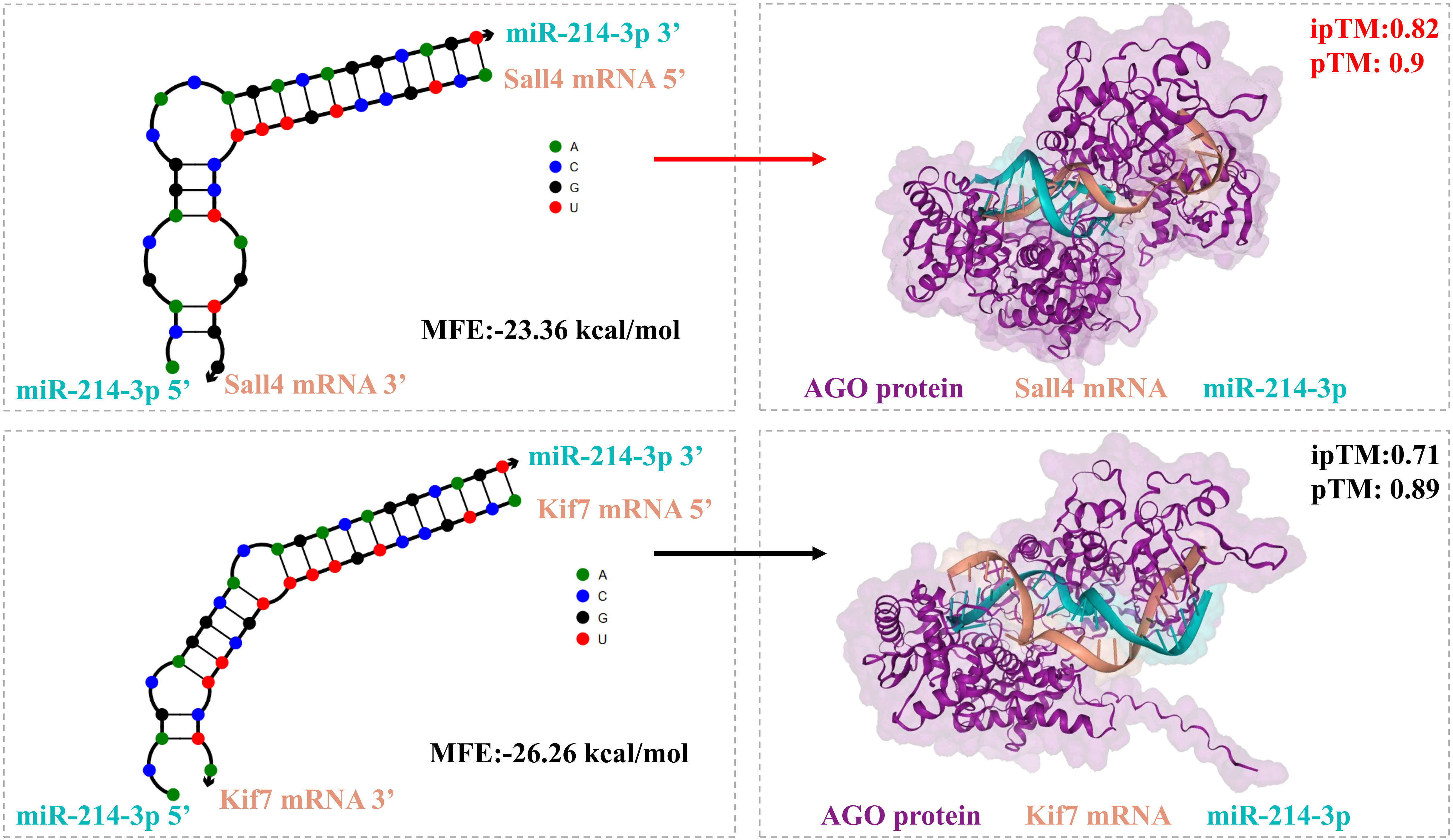

miR-214-3p 3'
Sall4 mRNA 5'
A
C
G
U
MFE:-23.36 kcal/mol
miR-214-3p 5'
Sall4 mRNA 3'
ipTM:0.82
pTM: 0.9
AGO protein
Sall4 mRNA
miR-214-3p
miR-214-3p 3'
Kif7 mRNA 5'
A
C
G
U
MFE:-26.26 kcal/mol
miR-214-3p 5'
Kif7 mRNA 3'
ipTM:0.71
pTM: 0.89
AGO protein
Kif7 mRNA
miR-214-3p

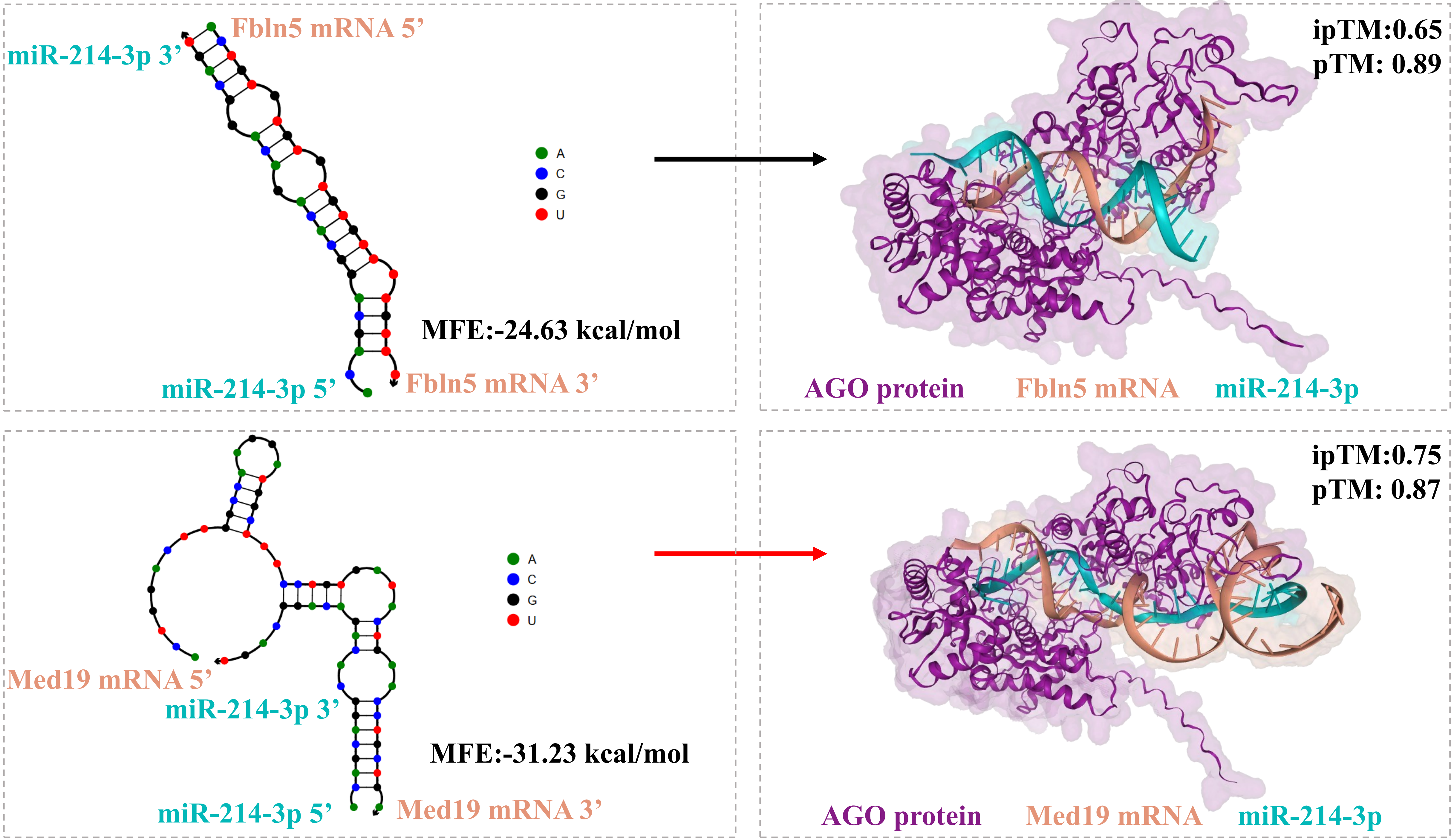
Fbln5 mRNA 5'
miR-214-3p 3'
A
C
G
U
MFE:-24.63 kcal/mol
miR-214-3p 5'
Fbln5 mRNA 3'
ipTM:0.65
pTM: 0.89
AGO protein
Fbln5 mRNA
miR-214-3p
A
C
G
U
Med19 mRNA 5'
miR-214-3p 3'
MFE:-31.23 kcal/mol
miR-214-3p 5'
Med19 mRNA 3'
ipTM:0.75
pTM: 0.87
AGO protein
Med19 mRNA
miR-214-3p

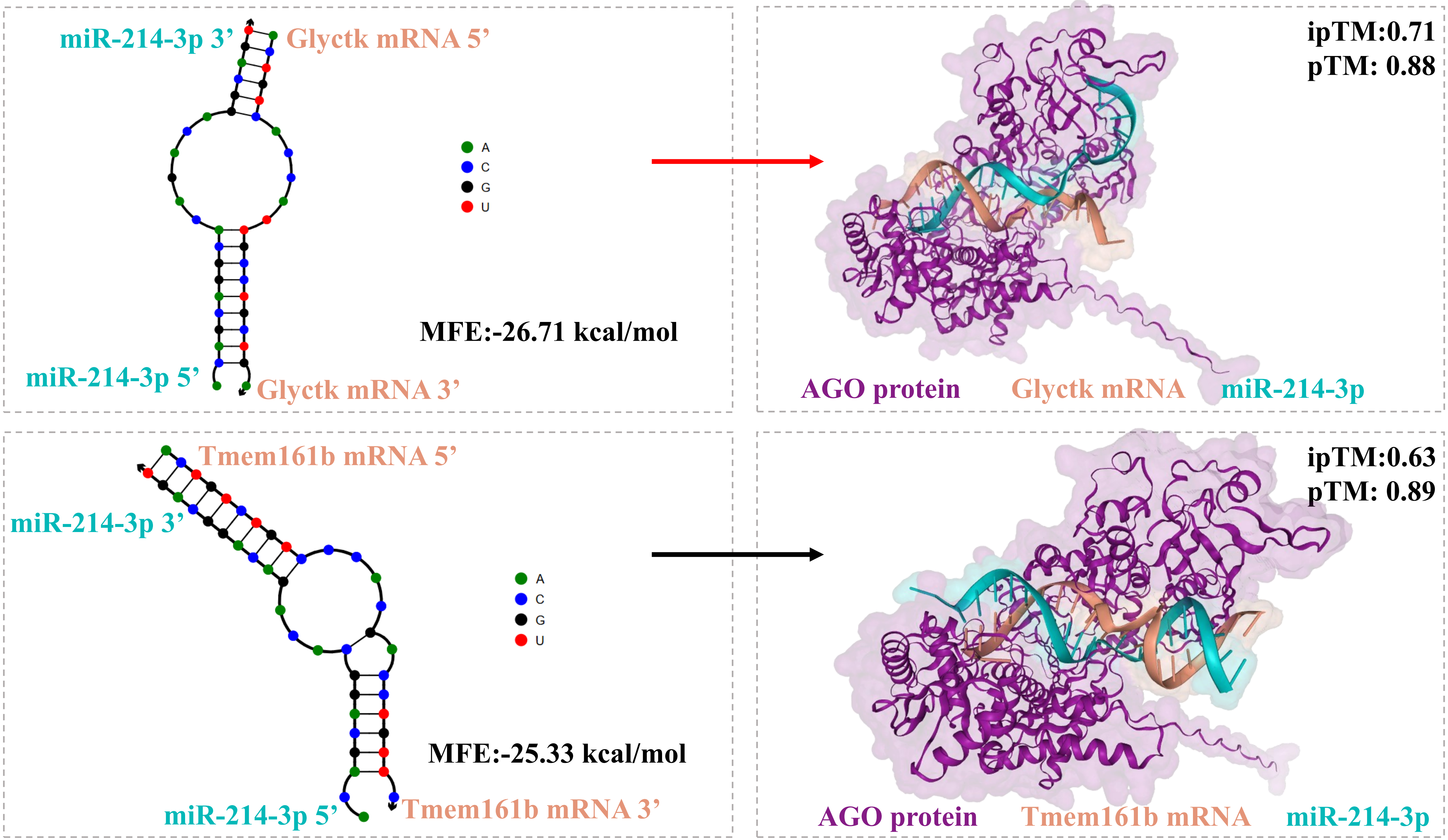
miR-214-3p 3'
Glyctk mRNA 5'
A
C
G
U
MFE:-26.71 kcal/mol
miR-214-3p 5'
Glyctk mRNA 3'
ipTM:0.71
pTM: 0.88
AGO protein
Glyctk mRNA
miR-214-3p
Tmem161b mRNA 5'
miR-214-3p 3'
A
C
G
U
MFE:-25.33 kcal/mol
miR-214-3p 5'
Tmem161b mRNA 3'
ipTM:0.63
pTM: 0.89
AGO protein
Tmem161b mRNA
miR-214-3p

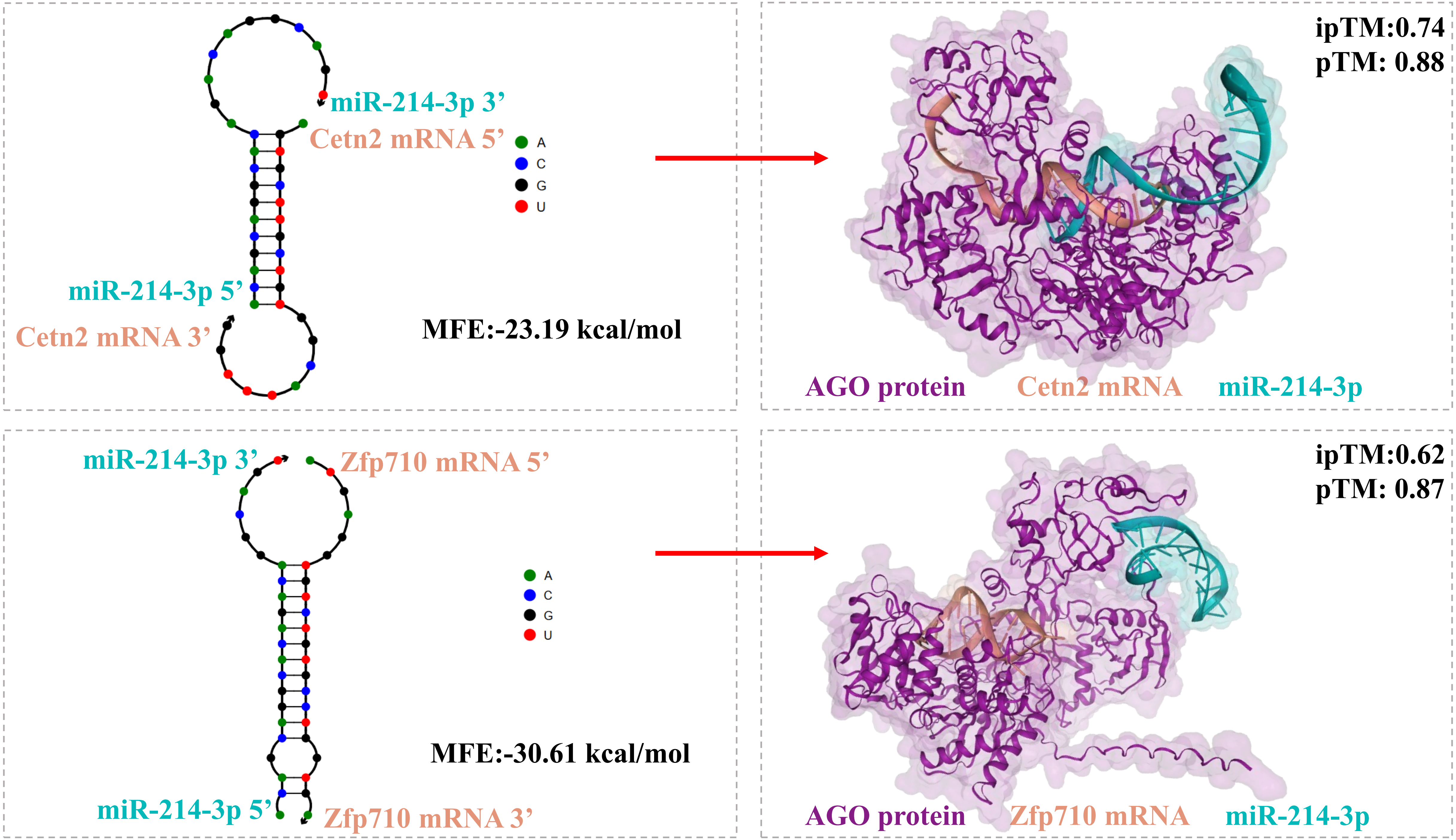
miR-214-3p 3'
Cetn2 mRNA 5'
A
C
G
U
miR-214-3p 5'
Cetn2 mRNA 3'
MFE:-23.19 kcal/mol
ipTM:0.74
pTM: 0.88
AGO protein
Cetn2 mRNA
miR-214-3p
miR-214-3p 3'
Zfp710 mRNA 5'
A
C
G
U
MFE:-30.61 kcal/mol
miR-214-3p 5'
Zfp710 mRNA 3'
ipTM:0.62
pTM: 0.87
AGO protein
Zfp710 mRNA
miR-214-3p

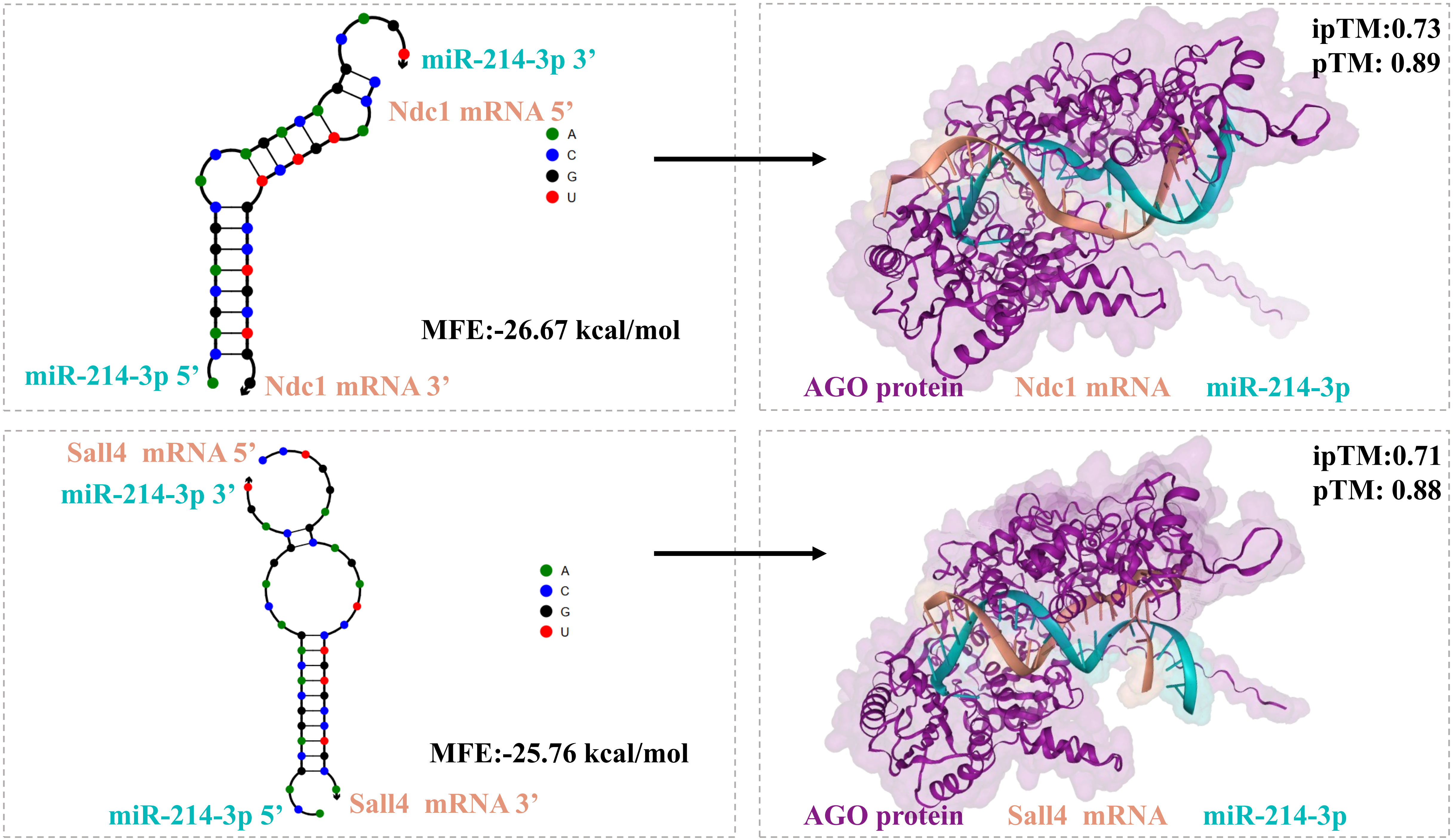
miR-214-3p 3'
Ndc1 mRNA 5'
A
C
G
U
MFE:-26.67 kcal/mol
miR-214-3p 5'
Ndc1 mRNA 3'
ipTM:0.73
pTM: 0.89
AGO protein
Ndc1 mRNA
miR-214-3p
Sall4 mRNA 5'
miR-214-3p 3'
A
C
G
U
MFE:-25.76 kcal/mol
miR-214-3p 5'
Sall4 mRNA 3'
ipTM:0.71
pTM: 0.88
AGO protein
Sall4 mRNA
miR-214-3p

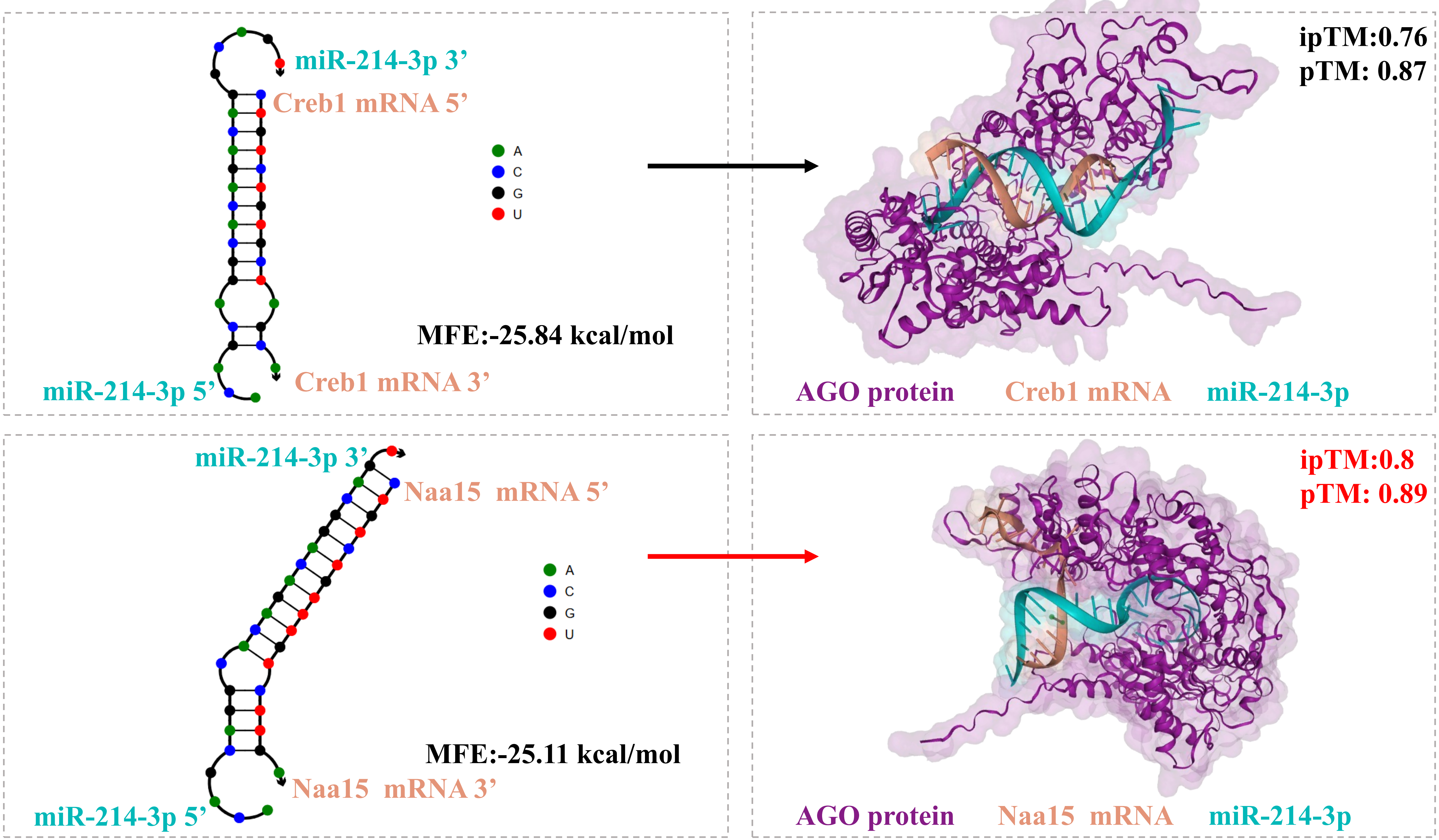
miR-214-3p 3'
Creb1 mRNA 5'
A
C
G
U
MFE:-25.84 kcal/mol
miR-214-3p 5'
Creb1 mRNA 3'
ipTM:0.76
pTM: 0.87
AGO protein
Creb1 mRNA
miR-214-3p
miR-214-3p 3'
Naa15 mRNA 5'
A
C
G
U
MFE:-25.11 kcal/mol
Naa15 mRNA 3'
miR-214-3p 5'
ipTM:0.8
pTM: 0.89
AGO protein
Naa15 mRNA
miR-214-3p

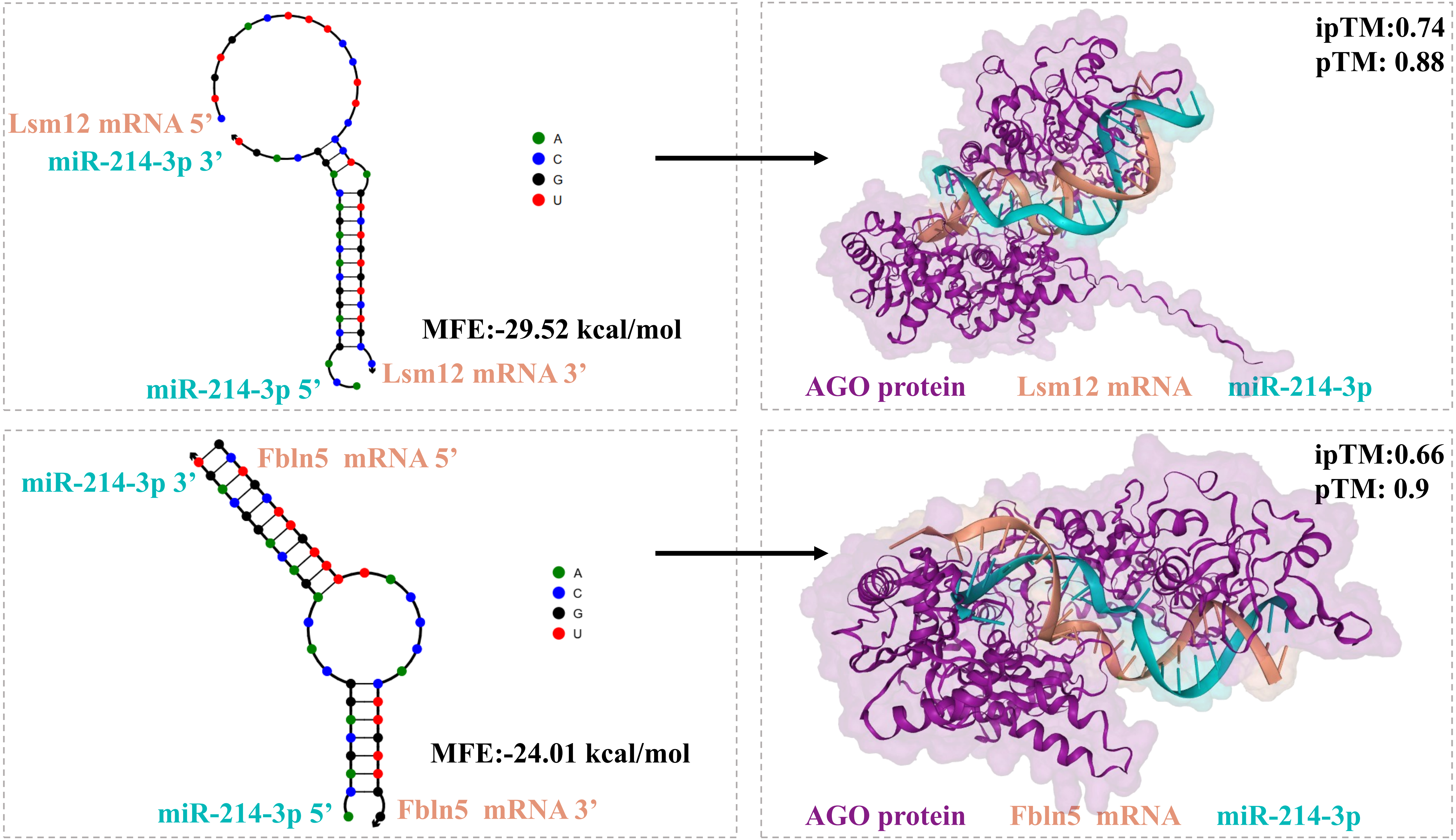

Lsm12 mRNA 5'
miR-214-3p 3'
A
C
G
U
MFE:-29.52 kcal/mol
miR-214-3p 5'
Lsm12 mRNA 3'
ipTM:0.74
pTM: 0.88
AGO protein
Lsm12 mRNA
miR-214-3p
Fbln5 mRNA 5'
miR-214-3p 3'
A
C
G
U
MFE:-24.01 kcal/mol
miR-214-3p 5'
Fbln5 mRNA 3'
ipTM:0.66
pTM: 0.9
AGO protein
Fbln5 mRNA
miR-214-3p

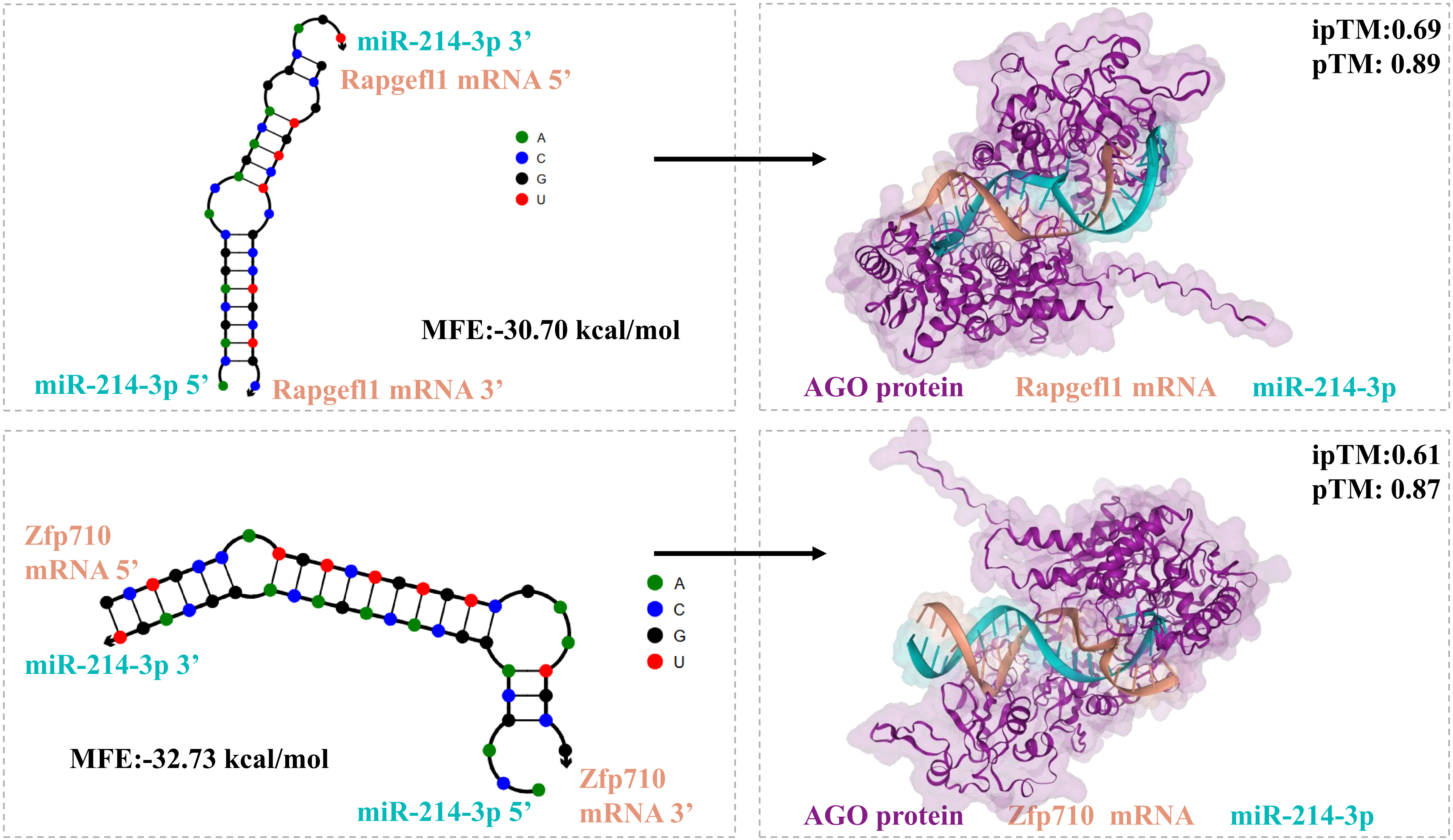

miR-214-3p 3'
Rapgefl1 mRNA 5'
A
C
G
U
MFE:-30.70 kcal/mol
miR-214-3p 5'
Rapgefl1 mRNA 3'
ipTM:0.69
pTM: 0.89
AGO protein
Rapgefl1 mRNA
miR-214-3p
Zfp710
mRNA 5'
miR-214-3p 3'
A
C
G
U
MFE:-32.73 kcal/mol
miR-214-3p 5'
Zfp710
mRNA 3'
ipTM:0.61
pTM: 0.87
AGO protein
Zfp710 mRNA
miR-214-3p

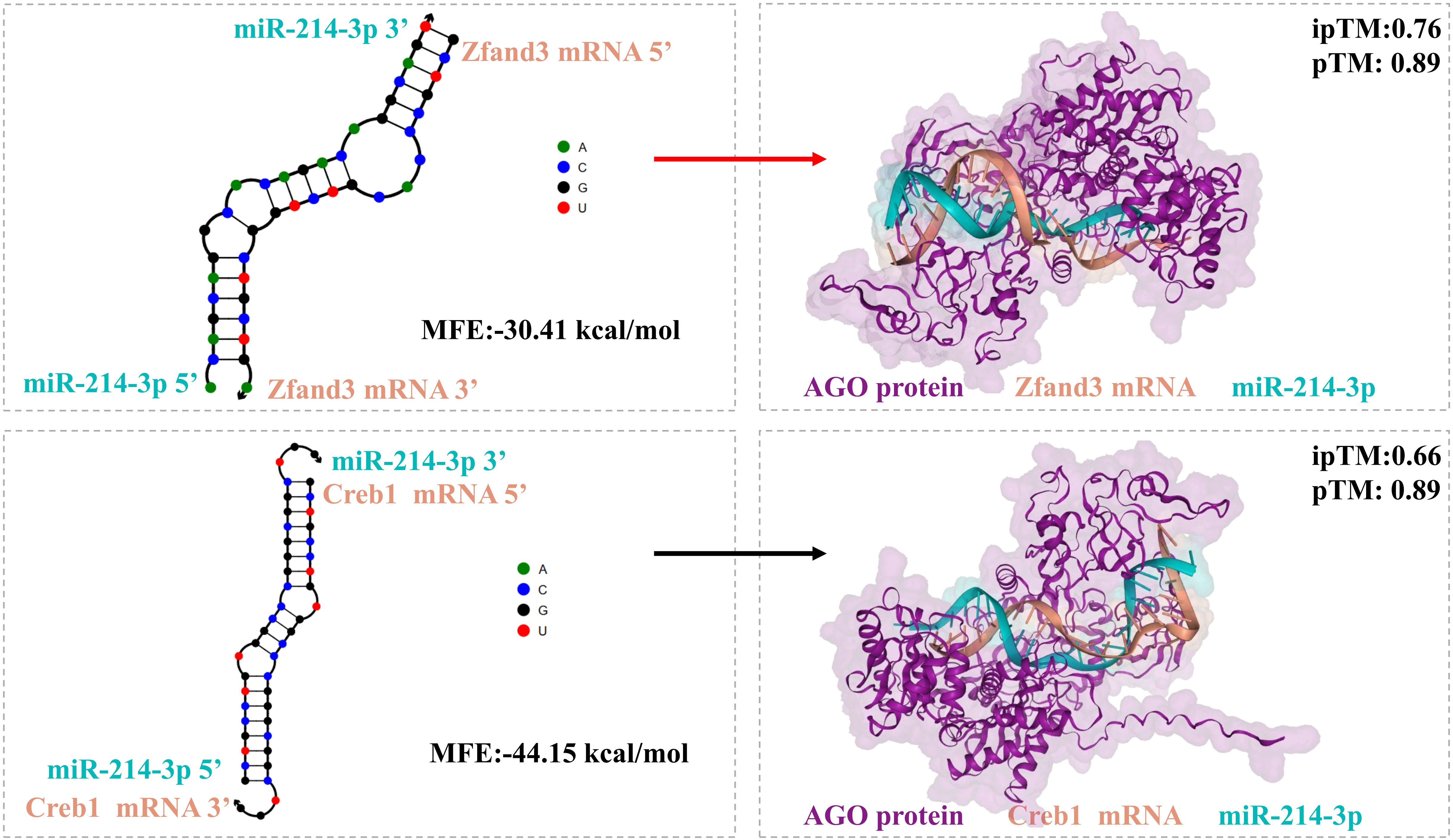

miR-214-3p 3’
Zfand3 mRNA 5’
A
C
G
U
MFE:-30.41 kcal/mol
miR-214-3p 5’
Zfand3 mRNA 3’
ipTM:0.76
pTM: 0.89
AGO protein
Zfand3 mRNA
miR-214-3p
miR-214-3p 3’
Creb1 mRNA 5’
A
C
G
U
MFE:-44.15 kcal/mol
miR-214-3p 5’
Creb1 mRNA 3’
ipTM:0.66
pTM: 0.89
AGO protein
Creb1 mRNA
miR-214-3p

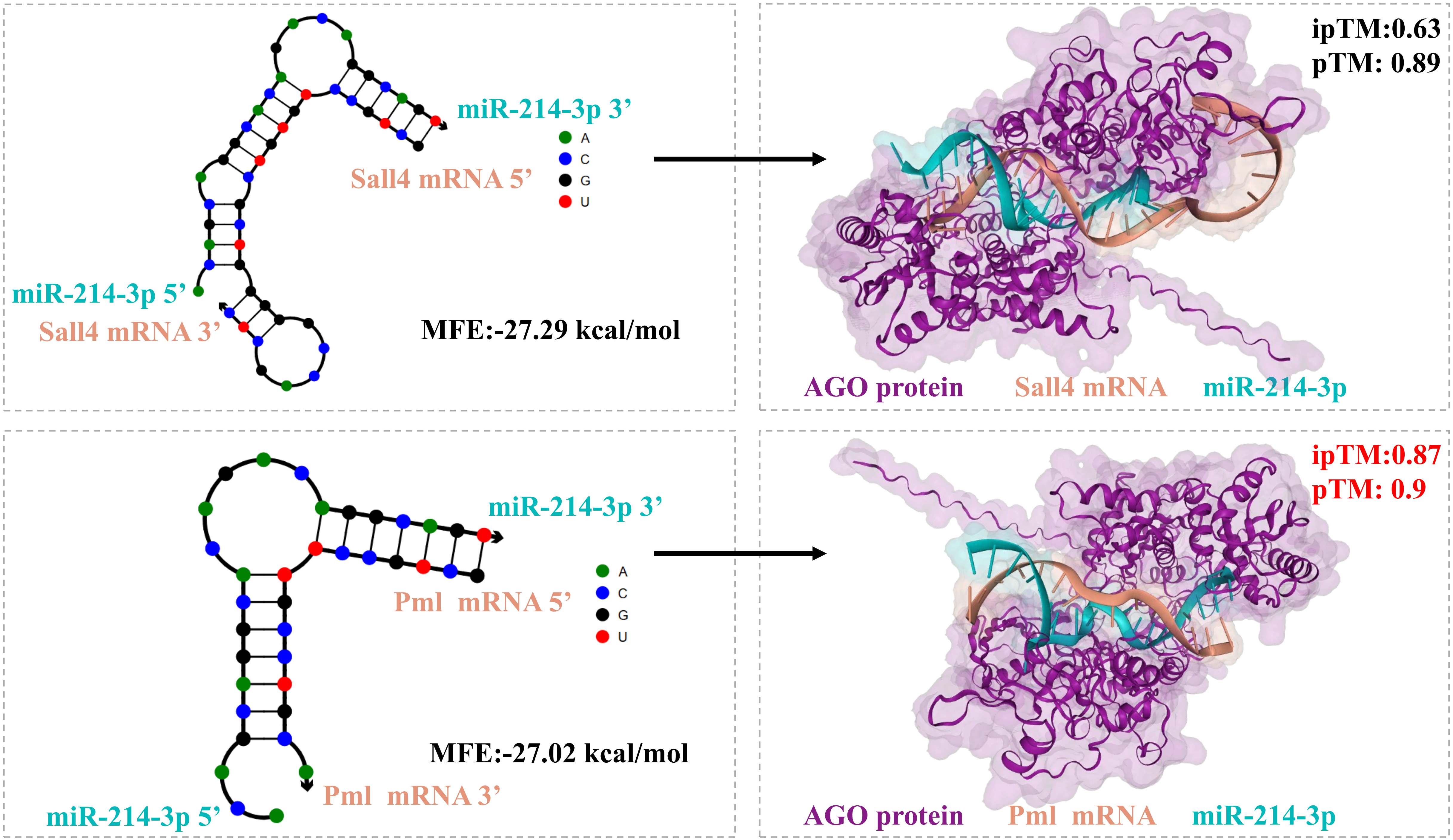
miR-214-3p 3'
Sall4 mRNA 5'
A
C
G
U
miR-214-3p 5'
Sall4 mRNA 3'
MFE:-27.29 kcal/mol
ipTM:0.63
pTM: 0.89
AGO protein
Sall4 mRNA
miR-214-3p
miR-214-3p 3'
Pml mRNA 5'
A
C
G
U
MFE:-27.02 kcal/mol
Pml mRNA 3'
miR-214-3p 5'
ipTM:0.87
pTM: 0.9
AGO protein
Pml mRNA
miR-214-3p

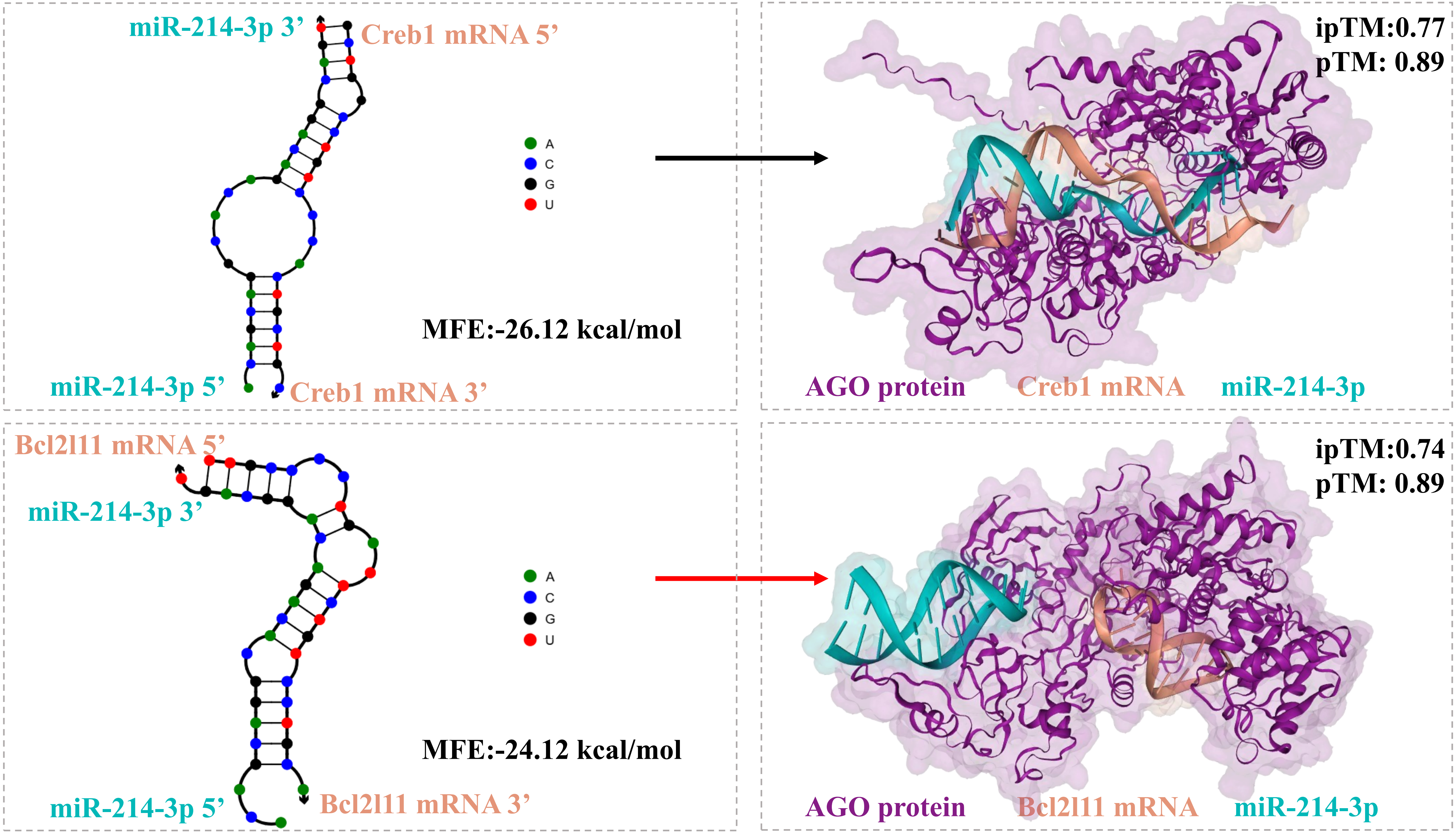
miR-214-3p 3'
Creb1 mRNA 5'
A
C
G
U
MFE:-26.12 kcal/mol
miR-214-3p 5'
Creb1 mRNA 3'
ipTM:0.77
pTM: 0.89
AGO protein
Creb1 mRNA
miR-214-3p
Bcl2l11 mRNA 5'
miR-214-3p 3'
A
C
G
U
MFE:-24.12 kcal/mol
miR-214-3p 5'
Bcl2l11 mRNA 3'
ipTM:0.74
pTM: 0.89
AGO protein
Bcl2l11 mRNA
miR-214-3p

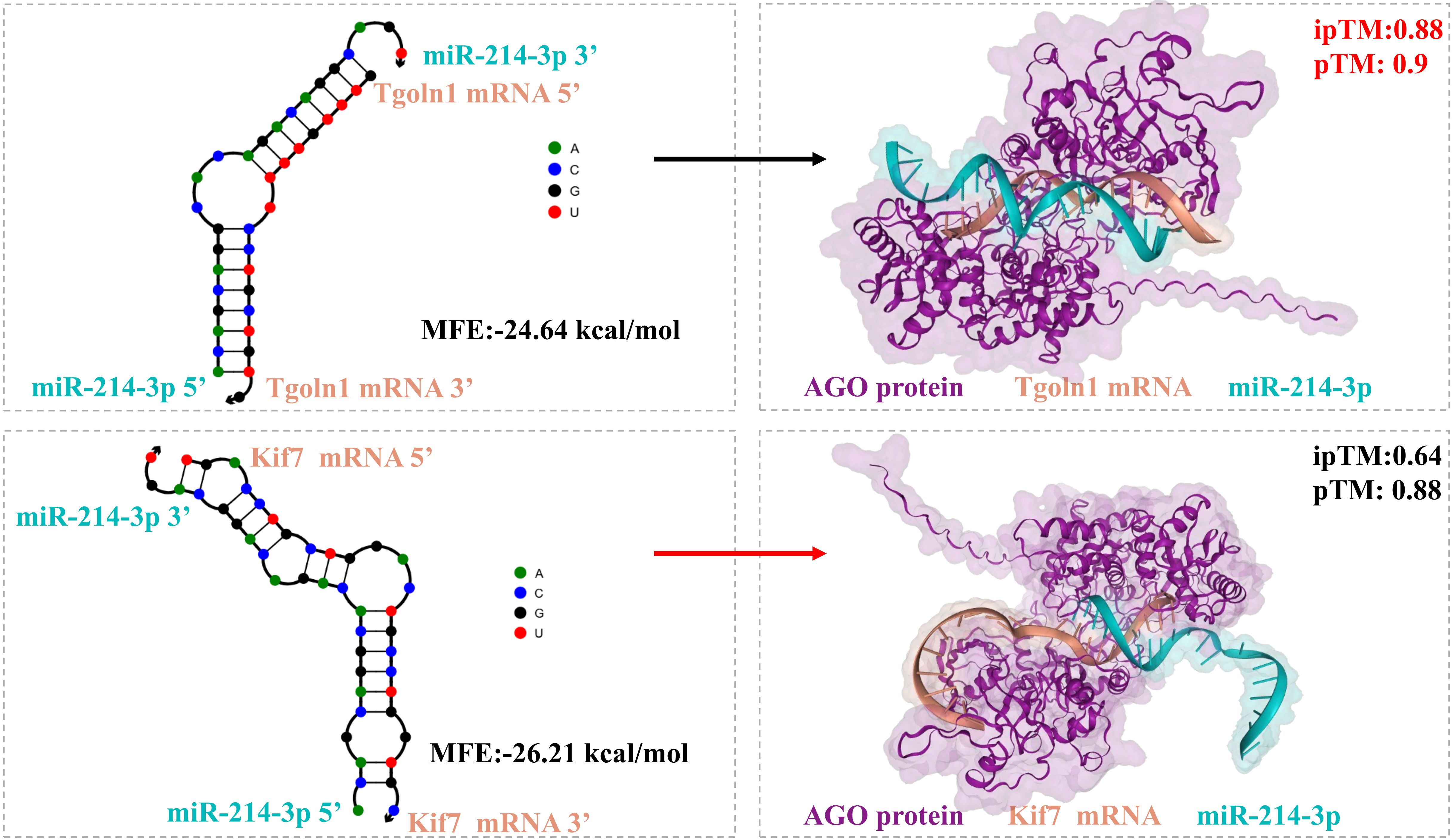

miR-214-3p 3'
Tgoln1 mRNA 5'
A
C
G
U
MFE:-24.64 kcal/mol
miR-214-3p 5'
Tgoln1 mRNA 3'
ipTM:0.88
pTM: 0.9
AGO protein
Tgoln1 mRNA
miR-214-3p
Kif7 mRNA 5'
miR-214-3p 3'
A
C
G
U
MFE:-26.21 kcal/mol
miR-214-3p 5'
Kif7 mRNA 3'
ipTM:0.64
pTM: 0.88
AGO protein
Kif7 mRNA
miR-214-3p

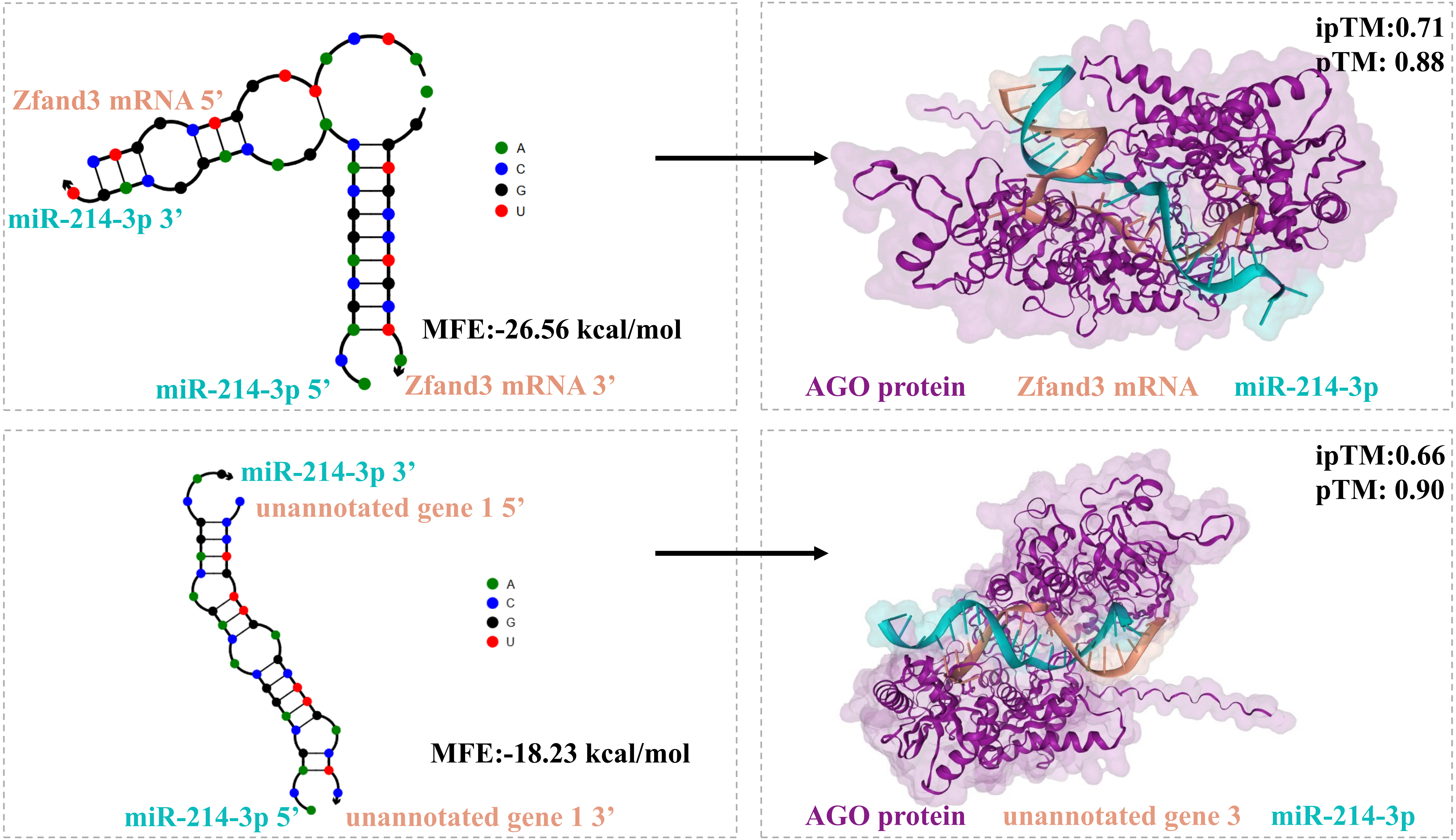
Zfand3 mRNA 5’
miR-214-3p 3’
A
C
G
U
MFE:-26.56 kcal/mol
miR-214-3p 5’
Zfand3 mRNA 3’
ipTM:0.71
pTM: 0.88
AGO protein
Zfand3 mRNA
miR-214-3p
miR-214-3p 3’
unannotated gene 1 5’
A
C
G
U
MFE:-18.23 kcal/mol
miR-214-3p 5’
unannotated gene 1 3’
ipTM:0.66
pTM: 0.90
AGO protein
unannotated gene 3
miR-214-3p

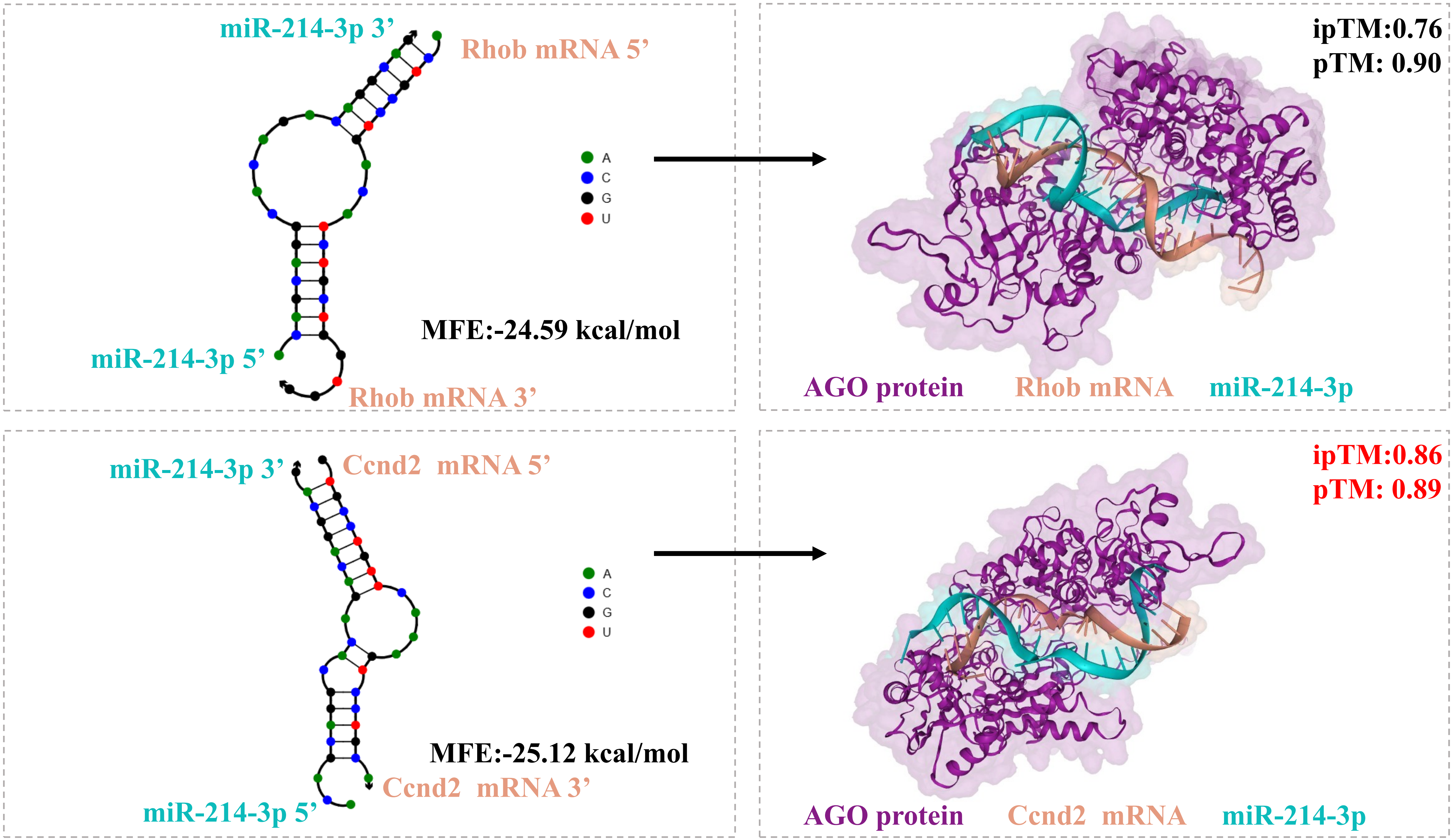

miR-214-3p 3'
Rhob mRNA 5'
A
C
G
U
MFE:-24.59 kcal/mol
miR-214-3p 5'
Rhob mRNA 3'
ipTM:0.76
pTM: 0.90
AGO protein
Rhob mRNA
miR-214-3p
miR-214-3p 3'
Ccnd2 mRNA 5'
A
C
G
U
MFE:-25.12 kcal/mol
Ccnd2 mRNA 3'
miR-214-3p 5'
ipTM:0.86
pTM: 0.89
AGO protein
Ccnd2 mRNA
miR-214-3p

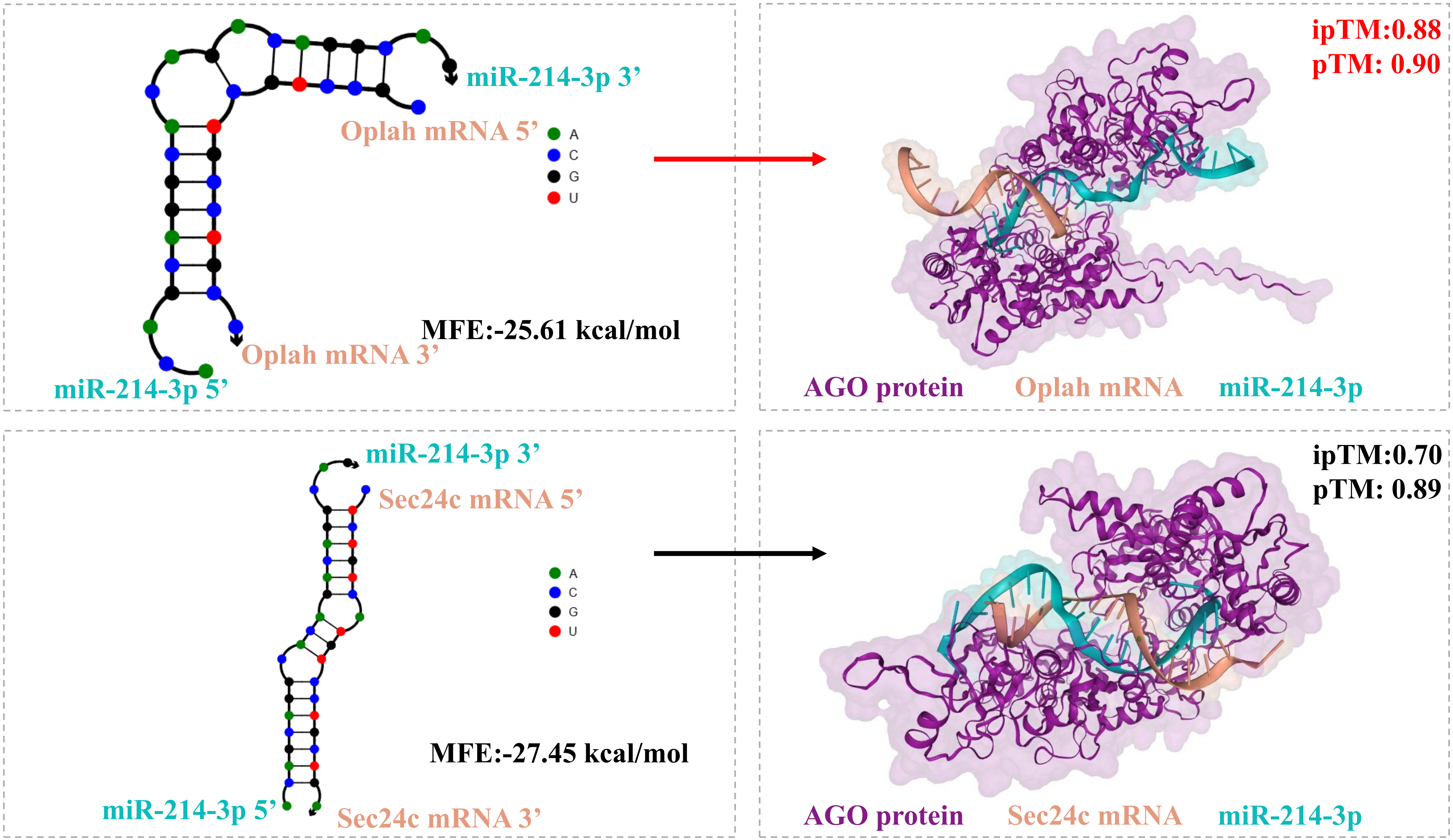
miR-214-3p 3'
Oplah mRNA 5'
A
C
G
U
MFE:-25.61 kcal/mol
Oplah mRNA 3'
miR-214-3p 5'
ipTM:0.88
pTM: 0.90
AGO protein
Oplah mRNA
miR-214-3p
miR-214-3p 3'
Sec24c mRNA 5'
A
C
G
U
MFE:-27.45 kcal/mol
miR-214-3p 5'
Sec24c mRNA 3'
ipTM:0.70
pTM: 0.89
AGO protein
Sec24c mRNA
miR-214-3p

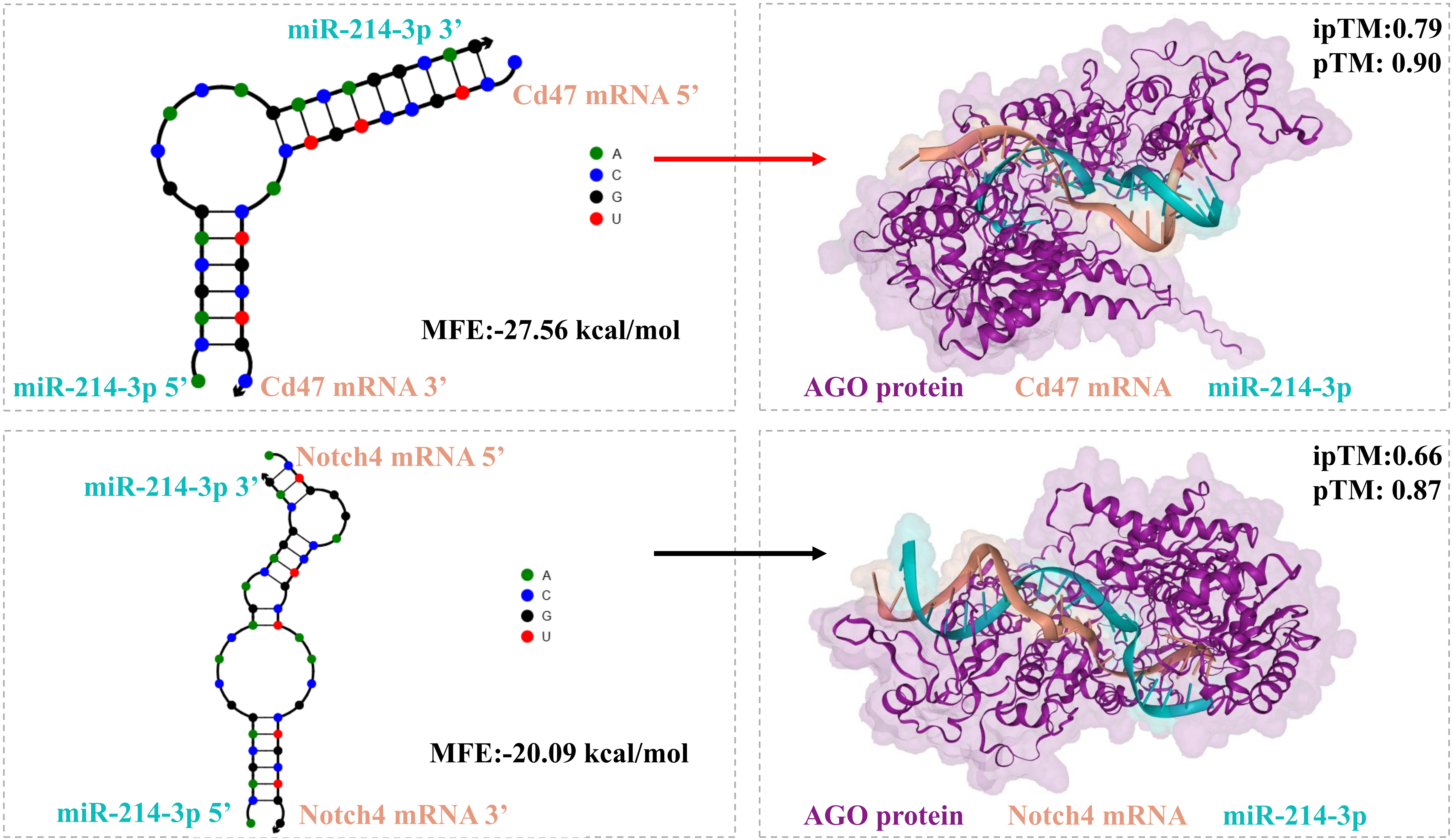

miR-214-3p 3'
Cd47 mRNA 5'
A
C
G
U
MFE:-27.56 kcal/mol
miR-214-3p 5'
Cd47 mRNA 3'
ipTM:0.79
pTM: 0.90
AGO protein
Cd47 mRNA
miR-214-3p
Notch4 mRNA 5'
miR-214-3p 3'
A
C
G
U
MFE:-20.09 kcal/mol
miR-214-3p 5'
Notch4 mRNA 3'
ipTM:0.66
pTM: 0.87
AGO protein
Notch4 mRNA
miR-214-3p

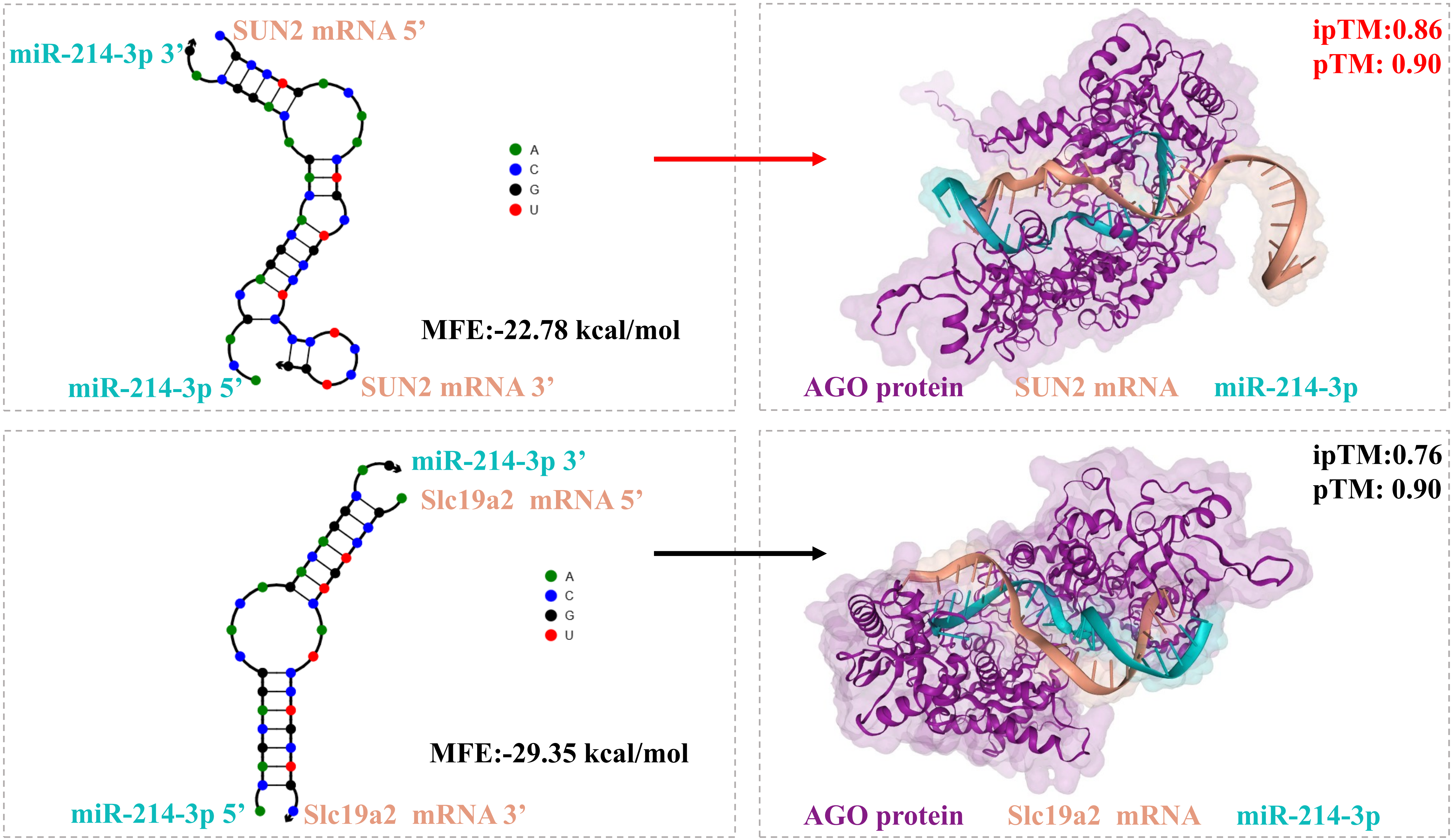
SUN2 mRNA 5'
miR-214-3p 3'
A
C
G
U
MFE:-22.78 kcal/mol
miR-214-3p 5'
SUN2 mRNA 3'
ipTM:0.86
pTM: 0.90
AGO protein
SUN2 mRNA
miR-214-3p
miR-214-3p 3'
Slc19a2 mRNA 5'
A
C
G
U
MFE:-29.35 kcal/mol
miR-214-3p 5'
Slc19a2 mRNA 3'
ipTM:0.76
pTM: 0.90
AGO protein
Slc19a2 mRNA
miR-214-3p

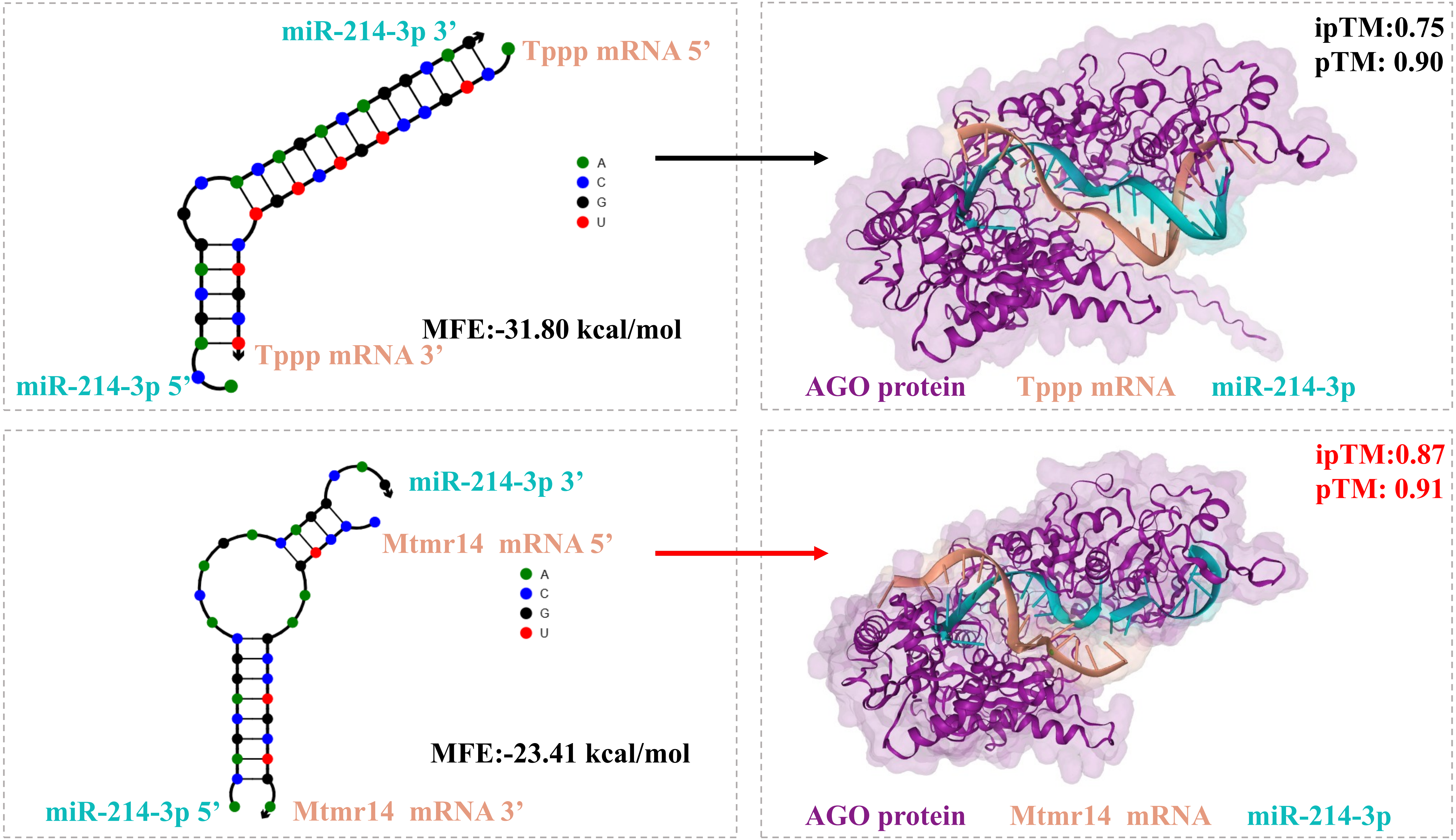
miR-214-3p 3'
Tppp mRNA 5'
A
C
G
U
MFE:-31.80 kcal/mol
Tppp mRNA 3'
miR-214-3p 5'
ipTM:0.75
pTM: 0.90
AGO protein
Tppp mRNA
miR-214-3p
miR-214-3p 3'
Mtmr14 mRNA 5'
A
C
G
U
MFE:-23.41 kcal/mol
miR-214-3p 5'
Mtmr14 mRNA 3'
ipTM:0.87
pTM: 0.91
AGO protein
Mtmr14 mRNA
miR-214-3p

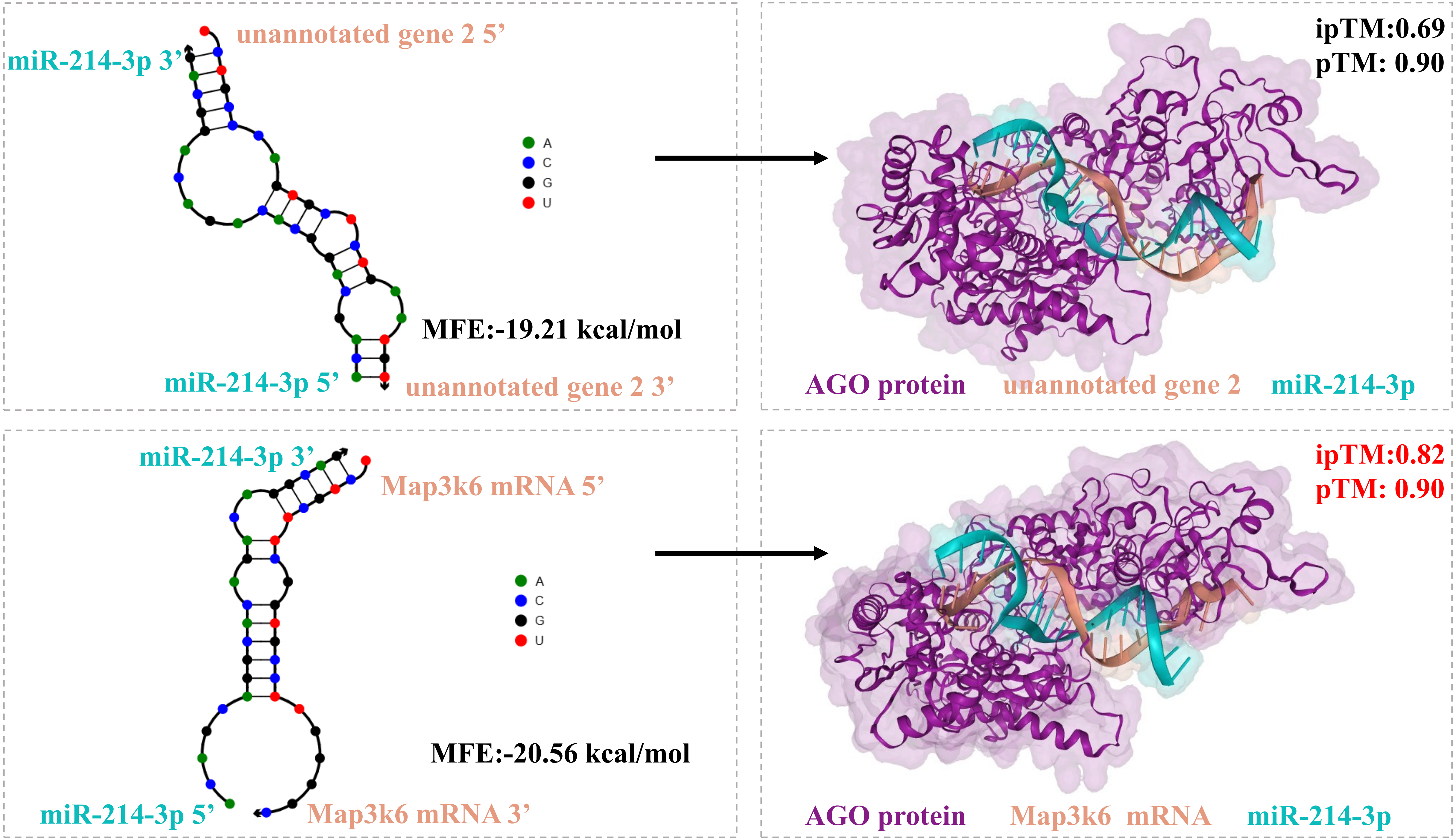
unannotated gene 2 5'
miR-214-3p 3'
A
C
G
U
MFE:-19.21 kcal/mol
miR-214-3p 5'
unannotated gene 2 3'
ipTM:0.69
pTM: 0.90
AGO protein
unannotated gene 2
miR-214-3p
miR-214-3p 3'
Map3k6 mRNA 5'
A
C
G
U
MFE:-20.56 kcal/mol
miR-214-3p 5'
Map3k6 mRNA 3'
ipTM:0.82
pTM: 0.90
AGO protein
Map3k6 mRNA
miR-214-3p

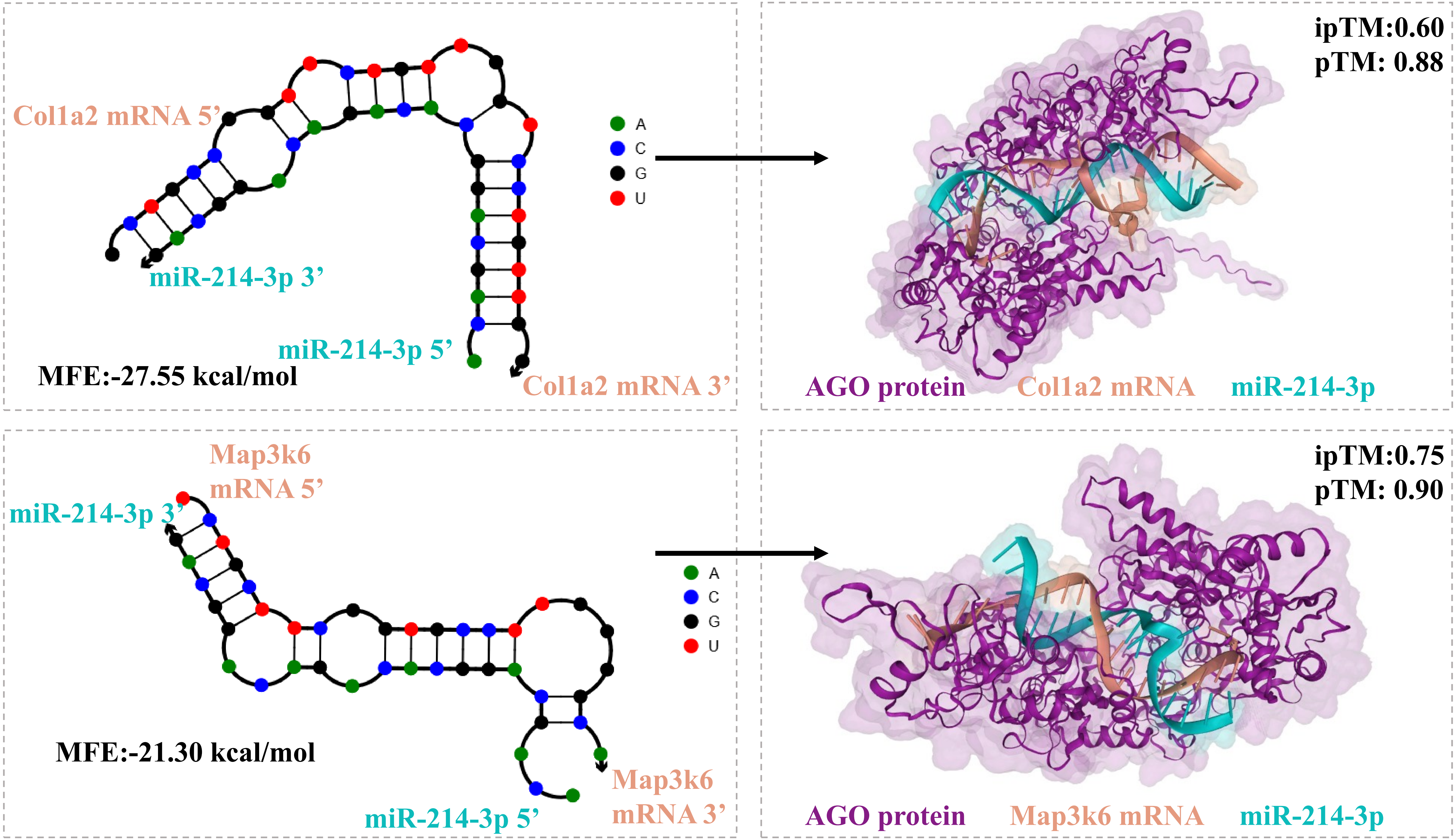

Col1a2 mRNA 5'
miR-214-3p 3'
A
C
G
U
miR-214-3p 5'
MFE:-27.55 kcal/mol
Col1a2 mRNA 3'
ipTM:0.60
pTM: 0.88
AGO protein
Col1a2 mRNA
miR-214-3p
Map3k6
mRNA 5'
miR-214-3p 3'
A
C
G
U
MFE:-21.30 kcal/mol
Map3k6
mRNA 3'
miR-214-3p 5'
ipTM:0.75
pTM: 0.90
AGO protein
Map3k6 mRNA
miR-214-3p

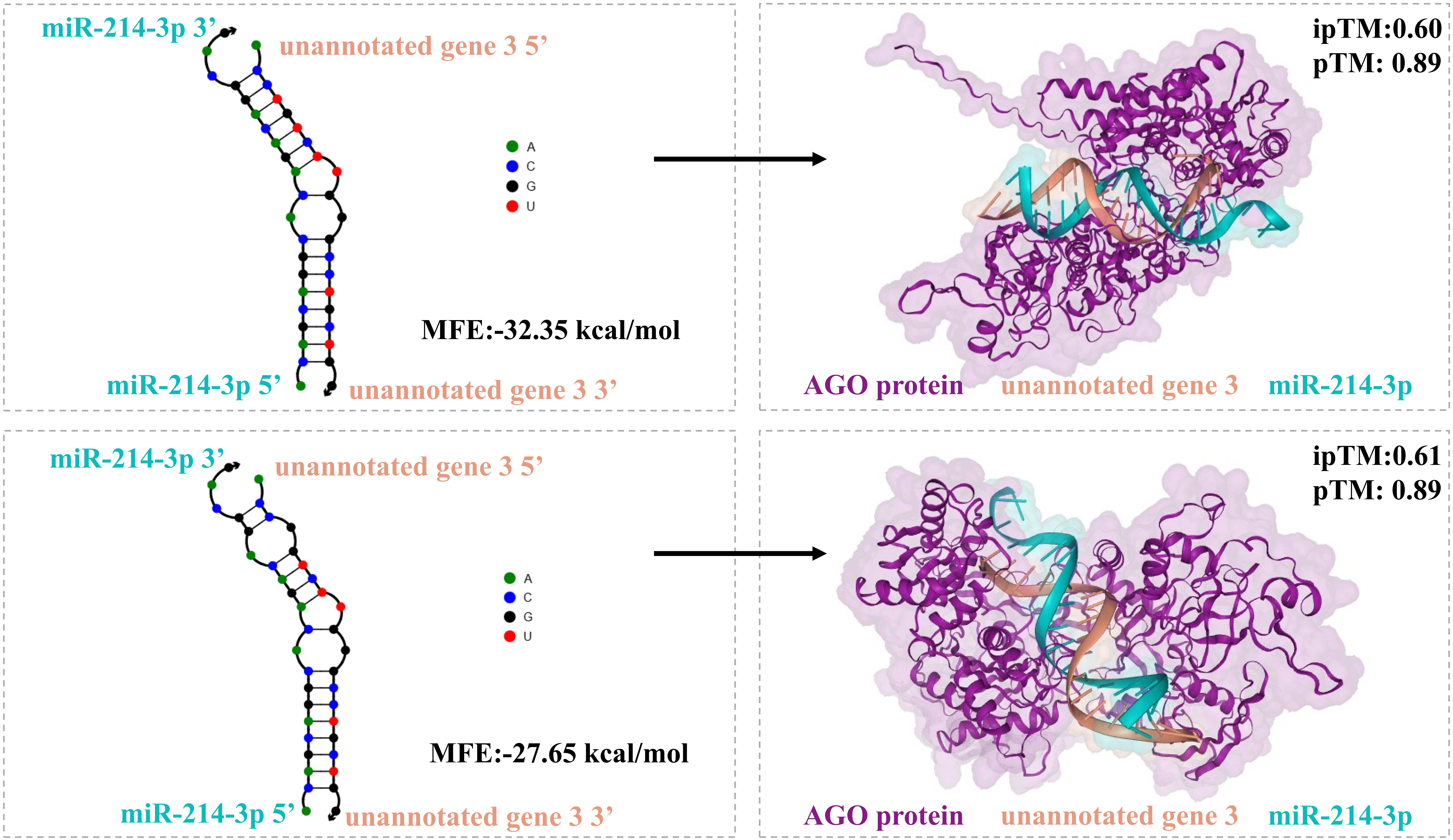
miR-214-3p 3'
unannotated gene 3 5'
A
C
G
U
MFE:-32.35 kcal/mol
miR-214-3p 5'
unannotated gene 3 3'
ipTM:0.60
pTM: 0.89
AGO protein
unannotated gene 3
miR-214-3p
miR-214-3p 3'
unannotated gene 3 5'
A
C
G
U
MFE:-27.65 kcal/mol
miR-214-3p 5'
unannotated gene 3 3'
ipTM:0.61
pTM: 0.89
AGO protein
unannotated gene 3
miR-214-3p
